# LexiRumah: An online lexical database of the Lesser Sunda Islands

**DOI:** 10.1371/journal.pone.0205250

**Published:** 2018-10-17

**Authors:** Gereon A. Kaiping, Marian Klamer

**Affiliations:** Leiden University Centre for Linguistics, Universiteit Leiden, Leiden, The Netherlands; Universita degli Studi di Modena e Reggio Emilia, ITALY

## Abstract

The Lesser Sunda Islands in eastern Indonesia cover a longitudinal distance of some 600 kilometres. They are the westernmost place where languages of the Austronesian family come into contact with a family of Papuan languages and constitute an area of high linguistic diversity. Despite its diversity, the Lesser Sundas are little studied and for most of the region, written historical records, as well as archaeological and ethnographic data are lacking. In such circumstances the study of relationships between languages through their lexicon is a unique tool for making inferences about human (pre-)history and tracing population movements. However, the lack of a collective body of lexical data has severely limited our understanding of the history of the languages and peoples in the Lesser Sundas. The *LexiRumah* database fills this gap by assembling lexicons of Lesser Sunda languages from published and unpublished sources, and making those lexicons available online in a consistent format. This database makes it possible for researchers to explore the linguistic data collated from different primary sources, to formulate hypotheses on how the languages of the two families might be internally related and to compare competing hypotheses about subgroupings and language contact in the region. In this article, we present observations from aggregating lexical data from sources of different type and quality, including fieldwork, and generalize our lessons learned towards practical guidelines for creating a consistent database of comparable lexical items, derived from the design and development of LexiRumah. Databases like this are instrumental in developing theories of language evolution and change in understudied regions where small-scale, pre-industrial, pre-literate societies are the majority. It is therefore vital to follow reliable design choices when creating such databases, as described in this paper.

## 1 Introduction

**Fig 1:**
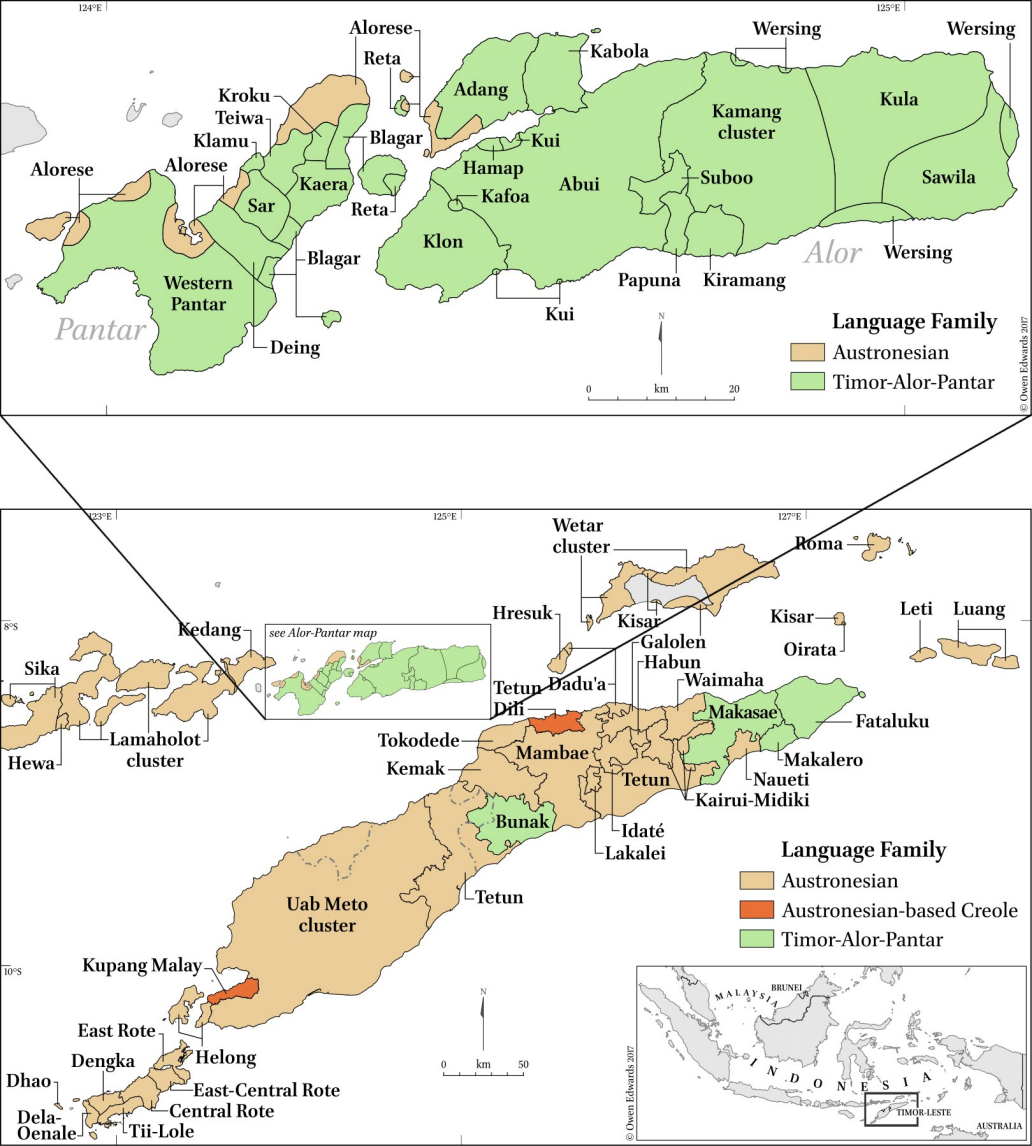
Linguistic map of the Lesser Sunda region. Members of the Austronesian family are shown in brown, Timor-Alor-Pantar languages in green.

The Lesser Sunda Islands in eastern Indonesia are an area of high linguistic diversity where several hundreds of often vastly different language varieties are spoken. The area covers a longitudinal distance of some 600 kilometres, including a multitude of islands, major ones of which are Flores, Lembata, Adonara, Solor, Pantar, Alor and Timor. This insular region constitutes the westernmost place where languages of the Austronesian family come into contact with Papuan languages of the Timor-Alor-Pantar (TAP) family in eastern Indonesia, as shown in Fig 1.

Despite its linguistic diversity, the Lesser Sunda Islands can also be characterized as little studied: for most of the region, written historical records, as well as archaeological and ethnographic data are sparse or absent. In such circumstances, language relationships have been very important tools for making inferences about human (pre-)history and tracing population movements. However, few linguists work in the Lesser Sundas, and their primary linguistic data is often unpublished or scattered across various (including “grey”, that is, unpublished but partly circulated) sources. The lack of a collective body of data collected from primary sources has severely limited our understanding of the history of the languages in the Lesser Sundas and the history of their people.

Very little is yet known about the populations and their movements in this area. The Austronesians are commonly assumed to have arrived in the Lesser Sunda area ~3,000 years Before Present (BP) [1,2], but the origin and age of the Papuan family spoken in the Lesser Sunda Islands (the Timor-Alor-Pantar (TAP) family) is less clear. One hypothesis holds that the speakers are descendants of immigrants from New Guinea who arrived in the Lesser Sundas 4,500–4,000 BP [1,3,4], and that their languages are part of the Trans New Guinea family (cf. [4,5]). However, lexical evidence to support such an affiliation is lacking [6,7]. Another hypothesis holds that the Papuans in the Lesser Sundas descend from arrivals 20,000 BP [8]. While this possibility cannot be excluded, the currently known level of lexical and grammatical similarity in the TAP family does not seem to support a family age of more than a few millennia [9]. In addition, ancient Austronesian loans, e.g. ‘betel nut’ [10], are found across the Alor-Pantar (AP) subgroup following regular sound changes, and suggest a higher order family split-up after contact with the Austronesian languages in the area, which would give the AP branch a maximum age of ~3,000 years. Work on the historical reconstruction of the TAP family has only just begun, and very little is yet known about the internal structure of low-level groups of the Austronesian languages in this area–let alone their higher order affiliations and external relations [7]. With languages of two families coexisting for millennia in a relatively small geographical space, the Lesser Sunda region is also a hotspot of language contact and a laboratory for studying the role of contact in the evolution of languages.

The aim of the LexiRumah database is to provide easy online access to large amounts of lexical data for the wider scientific community–including linguists, historians, and ethnographers. This database makes it possible for researchers to explore the linguistic data collected from primary sources, to formulate hypotheses on how the languages of the two families might be internally related and to compare competing hypotheses about subgroupings in the region [11–13]. It also allows researchers to study how and when languages might have borrowed particular words, both within and across the language family boundaries. LexiRumah can thus be instrumental in developing theories of language evolution and change in a hitherto understudied region where small-scale, pre-industrial societies are still the majority. And as the database includes geographical data, it also enables the study of relationships between language and space, in order to generate and evaluate hypotheses relating to geographic dispersals of speaker groups within and between the Lesser Sunda Islands. Finally, large-scale lexical databases like LexiRumah allow testing of automatic and quantitative reconstruction methods, which have been shown to be powerful tools for making inferences about human prehistory [14], and dating population expansions [15,16].

LexiRumah is a Cross-Linguistic Linked Database (http://clld.org/), based on the open source Lexibank (http://github.com/clld/lexibank/ [17]) and CLLD [18] software projects originally initiated by Martin Haspelmath at the Max Planck Institute for Evolutionary Anthropology, Leipzig, and further developed by the Max Planck Institute for the Science of Human History, Jena. The data of LexiRumah is published under the Open-Access CC-BY-4.0 license and available online under http://model-ling.eu/LexiRumah/, while supporting software (in the shape of the pylexirumah Python package, https://pypi.org/project/pylexirumah/) and the CLDF conform dataset ([19], http://cldf.clld.org) is available from https://doi.org/10.5281/zenodo.1164782.

LexiRumah is designed as a tool to investigate the linguistic history of the Lesser Sunda Islands and contains the lexicon of 101 varieties from two language families spoken in the region: Austronesian (Malayo-Polynesian) and Timor-Alor-Pantar. The database contains words for 596 concepts covering both basic and non-basic concepts, including culture-specific notions (e.g. ‘betel nut’), concepts for words that have been reconstructed to proto-Malayo-Polynesian (e.g. ‘canoe’) and concepts known to be highly borrowable (e.g. ‘to worship’) [20].

The development of this database is part of a general development in linguistics. Lexical materials are increasingly stored in structured formats which are machine readable, and are made available online. Examples of such databases include the ASJP database [21], which aims to contain 40-item word lists of all the world’s languages and currently covers ca. 2/3 of all ISO 639–3 codes; the Intercontinental Dictionary Series (IDS) [22], a database with lexical material from 329 languages across the world; and the Chirila database of lexical data of Australian indigenous languages [23]. For Austronesian and Papuan languages, there is the Austronesian Basic Vocabulary Database (ABVD) [24] and the TransNewGuinea.org database of languages of New Guinea [25]. Some of these databases are designed to be used for comparative phylogenetic research, and therefore they focus on basic vocabulary. Chirila and TransNewGuinea.org, however, were designed as ways to curate, compile and disseminate lexical data from old and recent sources, including dictionaries, and as such they also contain long word lists of particular languages.

### 1.1 Overview of the paper

In this paper, we first explain the aims of LexiRumah (Section 2). We then explain the database structure and user interface (Section 3), the data content (Section 4), the workflow and challenges of collecting the data and building the database (Section 5), and give a short example of how the database can be used in historical linguistics (Section 6). We conclude with a general discussion of experiences and lessons learned (Section 7) and an outlook towards plans for the future (Section 8).

## 2 Aims of LexiRumah

### 2.1 Academic aims

LexiRumah is designed as a tool to study the history of the Lesser Sunda Islands in eastern Indonesia. Classical methods in historical comparative linguistics largely focus on vertical transmission and internally motivated changes, aiming to reconstruct the common ancestor and mutual relationships within groups of related languages. Language contact studies, by contrast, focus on patterns and constraints in externally motivated changes. Truly unravelling the linguistic history of a region requires an approach which combines historical reconstruction with the study of language contact [26], investigating both vertical and horizontal transmission. Within the lexicon, items have different retention rates [27–29]. The general assumption is that basic vocabulary is less easily replaced than non-basic lexicon; for example, numerals are typically quite stable, while words expressing certain particular activities (e.g. ‘to squeeze’) show more variation over time [30]. Words also have different borrowability characteristics, e.g., nouns are more easily borrowed than verbs [31,32]. Loan words may be datable by their spread through a group of languages and level of integration into individual languages and may reflect pre-existing social networks. LexiRumah therefore comprises both basic vocabulary and borrowable vocabulary of various categories, including a range of region-specific vocabulary which could signal contact in particular socio-cultural domains like kinship, religion, politics, or technology at specific moments in time.

### 2.2 Other aims

LexiRumah also provides an accessible repository of lexical data to the general public in Indonesia and elsewhere in the world. It serves as a record of the cultural and linguistic identities and heritages of the minority groups who speak those languages. It makes the linguistic diversity in the Lesser Sundas even more visible for governmental and educational institutions in Indonesia. Educational institutions may decide to use the word lists to make informed decisions on the development of orthographies for local varieties. Internet access is cheap in Indonesia, and increasingly available in more remote locations.

### 2.3 Language collection policies

**Fig 2:**
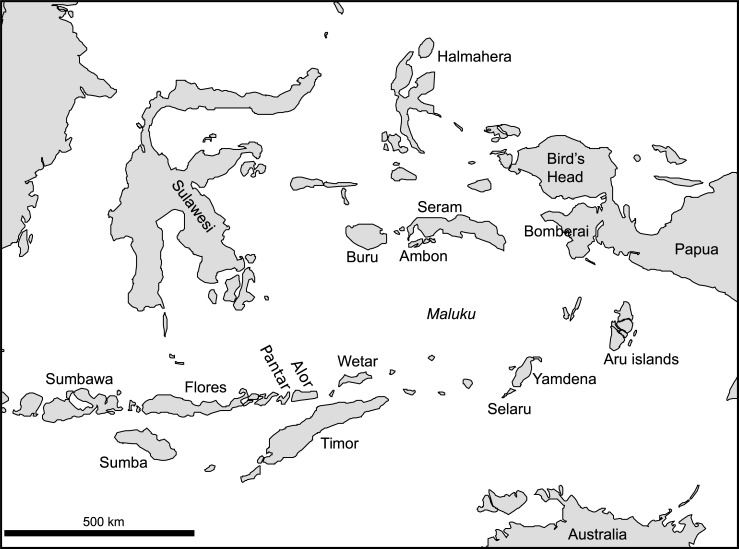
Map of Indonesia, with the locations mentioned in the text.

In compiling the materials for the database, we started with a focus on the languages of the Lesser Sunda Islands, where TAP languages form a Papuan family surrounded by Austronesian (Malayo-Polynesian, MP) languages. For this reason, we have included data on languages within and across the TAP family borders on Alor, Pantar and in eastern Timor, as well as data on MP languages to the west of Pantar, from the islands of Lembata, Adonara, Solor, Flores, and western Timor. More languages from these areas, from the islands north and south of Timor, and from Sumba, are currently being prepared for inclusion into the next version of LexiRumah. Genealogical affiliations between the MP languages in this region are as yet uncertain and understudied. Where known, the reconstructed forms from proto-languages are included in the database (see Section 4). Earlier studies [7] have suggested that there may be a lexical connection between TAP and languages of the Bomberai peninsula in west Papua. In order to further investigate this eastern connection, we plan to include Bomberai languages as well as lexicons from the languages of Maluku spoken on the islands of Wetar, Ambon, Seram, Aru, Kei, Yamdena, Selaru, and also Halmahera in the future, see Fig 2.

The database is not restricted to a single word list for each language. We have cases in LexiRumah of multiple dialects from the same language, for instance for Lamaholot, Alorese and Abui. The observation of micro-variation is useful for both the academic and the non-academic aims of the database. In fact, in several cases, for example for Lamaholot (ISO-693-3: slp, Glottocode: lama1277), it is not clear whether these different varieties should be classified as the same or different languages. In order to not take a stance on this, we will use the term ‘lect’ throughout this paper to refer to a language, dialect or other variety included in the database. Using the formal terminology defined by Good and Cysouw [33], our notion of ‘lect’ corresponds to ‘languoid composed of all those doculects with both broadly homologous glossonyms and matching location data’.

The data we include are from both published and unpublished sources, as well as from fieldwork, including fieldwork done specificially with the goal of gathering data for this database, see Section 5.1. However, as LexiRumah is not a primary but a secondary source, the originals of unpublished manuscripts and fieldwork data must be publically accessible, either online (e.g. in electronic archives) or in printed form (e.g. grammars, dictionaries). A large number of primary sources are already available online, but linking them to the database is an ongoing effort.

LexiRumah consists of not only borrow-resistant, stable core vocabulary (e.g. ‘sky’, ‘house’, ‘child’), but also culture specific words (‘bride price’, ‘betel nut’) as well as highly borrowable, lexically unstable concepts (see Section 4). Since the 1960’s, Indonesian has increasingly been used in education and the public domain of the region, and in most areas (a local variety of) Indonesian has become the lingua franca between speakers of different local languages. Due to this influence we expect that particularly the non-core lexicon of local languages recorded in the 2000’s is rather different from those recorded in the early 1900’s. Therefore we applied an additional policy for the sampling of data in the initial phase of our project: to collect data from comparable time-depths, recorded from speakers of maximally two generations difference. As most of our initial data concerns field data collected over the last decade, we decided for the time being to include only data from published sources from the year 1975 or later. There are a few published sources with lexical data of the region collected in the first half of the 20th Century (e.g. [34]), and data from these sources is currently collected to be included as part of the next phase of the project. Note that data presented in older published sources often has limited metadata; for example not including details about the individual speakers who provided the data, the location they originated from, the dialect and the year of recording, and so on.

In sum, the current version of LexiRumah contains data from published and unpublished sources, including fieldwork data; with comparable time-depths; sampled from lects fanning out from the Lesser Sunda’s. A next version is planned to include data from a wider geographical scope with a deeper time-depth.

## 3 Database structure and user interface

LexiRumah is available in two formats: For computational work with the data, the entire dataset can be downloaded from http://www.model-ling.eu/lexirumah/download or from the DOI https://doi.org/10.5281/zenodo.1164782 in Cross-Linguistic Data (CLDF) format, which builds on the CSV format while formally specifying the relations between table entries. A web application is served under http://model-ling.eu/lexirumah/, which makes browsing and exploring the data more convenient. The web interface exposes the complete data set, and makes the connections between various types of items (e.g. between a lect and its forms) available as internal hyperlinks. External connections to other databases (Glottolog, Concepticon, Ethnologue; see below) are presented as external links online. Similar to other existing cross-linguistic linked database applications, the map is interactive and provides contextual information on interaction with the mouse cursor, and tables as well as individual entries with their metadata can be downloaded in various formats (e.g. HTML, JSON or XSL).

**Fig 3:**
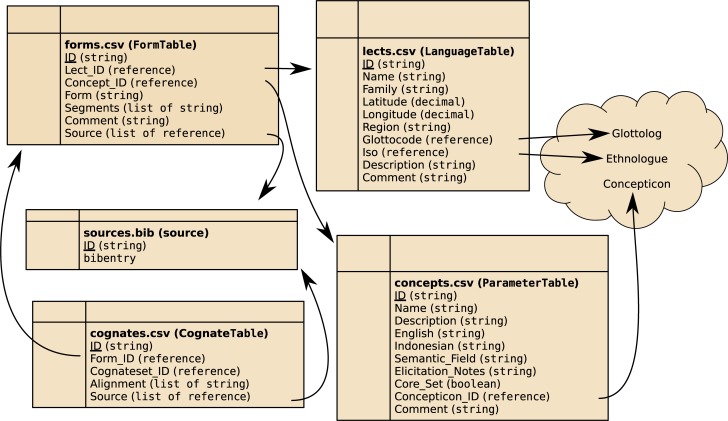
The structure of LexiRumah. → denotes the target of a reference field (e.g. the Lect_ID reference in forms.csv points to an entry in lects.csv).

In the following, we describe the overall logical structure of the LexiRumah database. A visualization is given in Fig 3. The database implements the CLDF-1.0 module Wordlist [19]; the formal description according to the CLDF standard [19] can be found in the Supplementary Material.

### 3.1 Word form table

The central table of the database is the ‘word form’ table. It lists all forms, together with a unique numerical ID, a free-text comment field and the database-internal references to the lect they come from, the concept they express, and the source that provided the form. Metadata concerning lects, concepts and references are listed in separate tables.

Sources for forms are either fieldwork data, or written (un-)published sources. For data collected in fieldwork, the word form is the utterance that the speaker provided as response to the Indonesian prompt, represented in broad IPA transcription (see Section 5.2), that is, a phonetic transcription not expected to convey more detail than available using any basic IPA letter (vowels and consonants), plus the symbols for “long” (:), “primary stress” (‘), “nasalized” (~) and “syllable break” (.). When the word form has a published source, it is cited as it is presented in that source; a segmented translation into broad IPA is stored separately, to account for cases where the source uses a different orthography.

Word forms may be polymorphemic: nominal word forms may carry a possessive affix (e.g. when speakers do not separate inalienable possession affixes from nominal roots) and verbs may have subject or object agreement marker(s). Other word forms may contain derivational morphemes, consist of compounds, be serial verbs, or form a phrase; for example, when the concepts ‘cheap’ and ‘lake’ are translated as, respectively, ‘price light’ and ‘pool still’. Where known, the glosses of polymorphemic word forms are given in the Comments field.

### 3.2 Concept table

**Fig 4:**
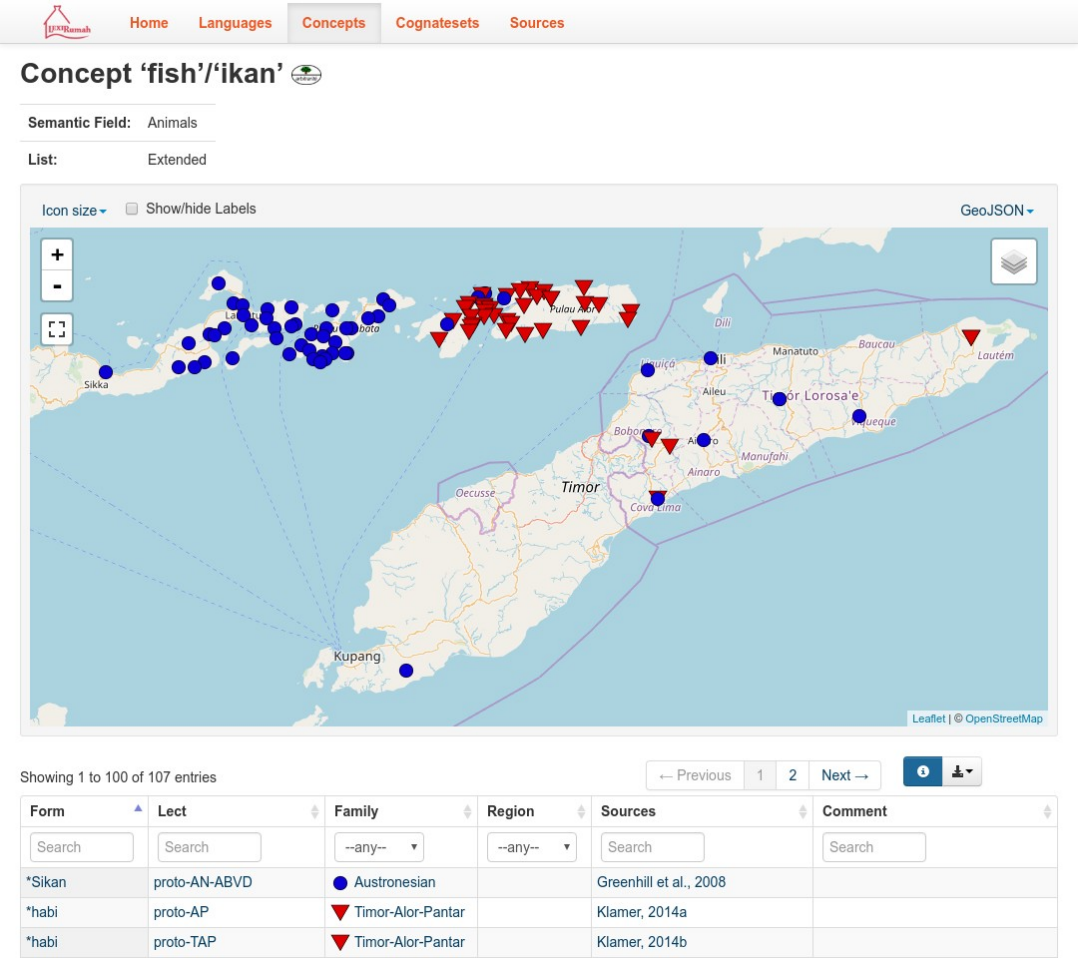
The concept ‘fish’/‘ikan’ in LexiRumah online.

The table of all concepts (‘parameters’ in the more general CLDF framework) lists all concepts in the database. It is indexed by the English glosses in a simplified format. The simplified glosses use only RFC 3986 [35] unreserved characters, eg. replacing spaces with underscores and removing brackets, so they can be part of a URI without re-encoding. Each concept is listed with an English and Indonesian expression for the meaning as used in fieldwork elicitation or given in the written source, as well as a normalized English gloss which Concepticon [36] uses for this or the closest related meaning. Free text fields are provided for elicitation notes and further comments. For example, for the concept ‘(fireplace) ash’, which is elicited with Indonesian *abu*, a note is given: “NOT ‘debu’ (dust, after you have been sweeping)” as some speakers of Lesser Sunda languages interpret Indonesian *abu* as (also) meaning ‘dust’. Fig 4 illustrates the concept ‘fish’/ ‘ikan’.

To enable easy searching for groups of words belonging to particular semantic fields, each concept is assigned to a Semantic Field. For example, the concept ‘sky’ is assigned to the field ‘The physical world’, the concept ‘tomorrow’ to the semantic field ‘Time’, the concept ‘father’ to the field ‘Kinship’. The choice and names of the semantic fields are taken from the World Loanword Database (WOLD), [37].

Each Concept also has a link to Concepticon [36]. Concepticon is an extensive concept lexicon [38] which links a large amount of different concept labels to concept sets. The concept labels come from different concept lists. Those concept lists present in Concepticon are all used in the linguistic literature and range from Swadesh lists as used in historical linguistics to words for naming tests in clinical studies and psycholinguistics. By linking a concept in LexiRumah to its equivalent in Concepticon, the concept can be studied in relation to other concepts, in other languages, as well as their sources. As a rich reference for new and existing databases in diachronic and synchronic linguistics, the link to Concepticon enables users of LexiRumah to have quick access to studies on semantic change, cross-linguistic polysemies, and semantic associations.

### 3.3 Lect table

**Fig 5:**
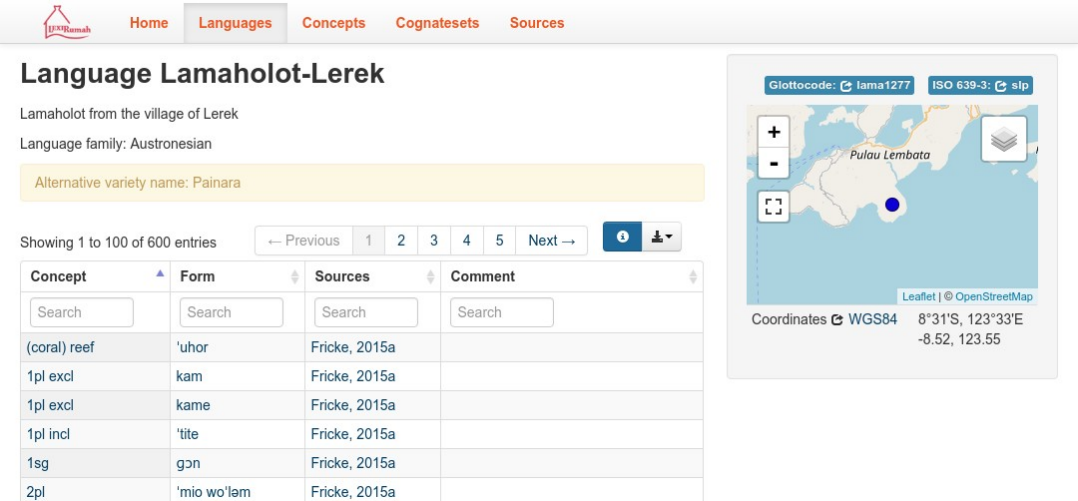
The Lamaholot-Lerek lect in LexiRumah online.

Each word form comes from a particular lect or linguistic variety. LexiRumah documents language varieties, and whether the varieties are considered dialects or languages is a matter we do not take a stance on. The data about all lects is stored in a separate table, which is referenced by the Form table. This table, named ‘Language Table’ following the CLDF standard for data of this type, stores for each lect its name, a description and possible comments in free-text fields. An example is given in Fig 5.

Location is stored as geo-coordinates for latitude and longitude. The coordinates refer to the location where the list was compiled and recorded, or, in case the recording was made in a location different from where the lect is spoken, the place of origin of the speaker that was recorded. Sometimes this information is not available in a (published) source; in such cases, the reference is put in the approximate middle of the known range of the variety, or of the village name associated with the lect. Geo-coordinates have been mapped to region-level administrative districts using the Google Geocoding API.

All Lects table entries also list their higher-order family association–‘Austronesian’ or ‘Timor-Alor-Pantar’–, an ISO 639–3 (three letter) language code and a Glottolog 2.7 identifier [39]. While some of our lects have individual glottocodes already, not all of them do. Integration of our lects and their classification into Glottolog is ongoing.

In the online interface of the database, the geo-coordinates permit us to plot lect locations on a map. The glottocodes and ISO codes are used to provide links to Glottolog and Ethnologue, to make it easier to aggregate data about these lects from different sources.

### 3.4 Source table

The Sources from which the word forms are taken are listed in a separate, bibliography-like structure in BibTeX format. It is indexed by a short internal reference, often consisting of a name and date. In case of a published source, this structure contains the bibliographical information about the data provenience. In case of unpublished written work, the structure contains the reference to the URL and/or online archive where it can be found. In case of a fieldwork recording, the structure contains the metadata of the recording. This includes (where it is known) the names of the speaker(s) on the recording, their place of origin, its geographical coordinates and name(s), any places of residence of the speakers, and name(s) and date(s) of the recorder(s) and transcriber(s).

### 3.5 Cognate table

Apart from the lexical data, the database also contains data on observed similarities between different forms in the Cognate table. Currently, the similarity data is generated using an automatic algorithm (LexStat) described in Section 5.8. The table will also be able to store cognate judgements from different sources, indeed conflicting judgements for the same form. The aim of these similarity codes is to provide data for research into the lexical similarity of the lects involved and to serve as a basis for systematic comparative work doing bottom-up reconstruction of the linguistic history, by identifying potential cognate clusters and making them available in a consistent format.

This data is stored in a separate table. Each entry of that table associates a word form (identified by its numerical ID) with a similarity class (identified by an arbitrary simple string, generally a numerical for now, but able to accommodate postulated proto-forms once clearly identified) and an alignment of the sound segments of that form to other forms in that class. The table of aligned cognate forms gives an implicit description of which sounds correspond to each other, and in which positions a sound segment might have been gained or lost (marked by “-”). The table also provides columns to list the sources which propose that a form be assigned to that particular class or alignment.

The similarity coding and alignments published in the current release of the database were generated using the open-source historical linguistics software package LingPy [40,41]. The process to generate them is described in the next section.

## 4 Data content

Recent and not so recent surveys of underexplored areas of the Lesser Sundas resulted in a collection of lexical materials from languages in Timor, Alor and Pantar in spreadsheets. These sheets were translated into CLDF and aggregated into a single dataset. Additional data were compiled from paper manuscripts, published and unpublished word lists, published dictionaries, and lexicons built during fieldwork, using the software packages Toolbox (https://software.sil.org/toolbox/) or Fieldworks (https://software.sil.org/fieldworks/).

Currently, LexiRumah contains data on 101 lects of which 59 are Austronesian (Malayo-Polynesian) and 42 Timor-Alor-Pantar. The lects represent to 32 different ISO 639–3 codes in total, giving us a lower bound on the number of different languages contained. In some cases, for example for Lamaholot (ISO 639–3: slp), it is doubtful that all lects listed under that ISO code should be classified as dialects of the same language, so the actual number of languages reflected in LexiRumah is likely higher. However, we have a few clear cases in LexiRumah of multiple dialects from the same language, such as for Alorese and Abui.

The database contains expressions (‘counterparts’) for 596 different concepts. These include basic vocabulary, region-specific vocabulary, and highly borrowable vocabulary. The vocabulary items for basic concepts are roughly the 200-item Swadesh list. In addition, various researchers who did surveys in the region over the last decade have added region/culture-specific (basic) concepts to this list, including ‘betelnut’, ‘betelvine’, ‘to chew betelnut’, ‘lime’, ‘corn’, ‘sweet potato’, ‘taro’, ‘rice field’, ‘rice grains’, ‘rice plant’, ‘to plant (rice)’, ‘uncooked rice’, ‘cooked rice’, ‘sago’, ‘bride price’, ‘dowry’, ‘machete’, ‘canoe’, ‘sail’, ‘(coral) rock coral (reef)’, ‘fishing hook’, ‘mosquito’, ‘scabies’. As we wanted to be able to also investigate lexical borrowing, we added meanings from semantic fields that are known to be highly borrowable [20,37]. The following ten semantic fields rank the highest on a borrowability scale: Religion and belief, Clothing and grooming, The house, Law, Social and political relations, Agriculture and vegetation, Food and drink, Warfare and hunting, Possession, and Animals. For some of these fields (Animals, Agriculture and vegetation) our list already had more than 40 concepts, and we added concepts for each of the other eight fields so that each of them contained at least 15 concepts. The borrowable concepts that were added were selected from [37,42,43], giving preference to concepts that occur in at least two of these sources, meanings that are commonly used in the area of research, and meanings that are culturally relevant.

**Fig 6:**
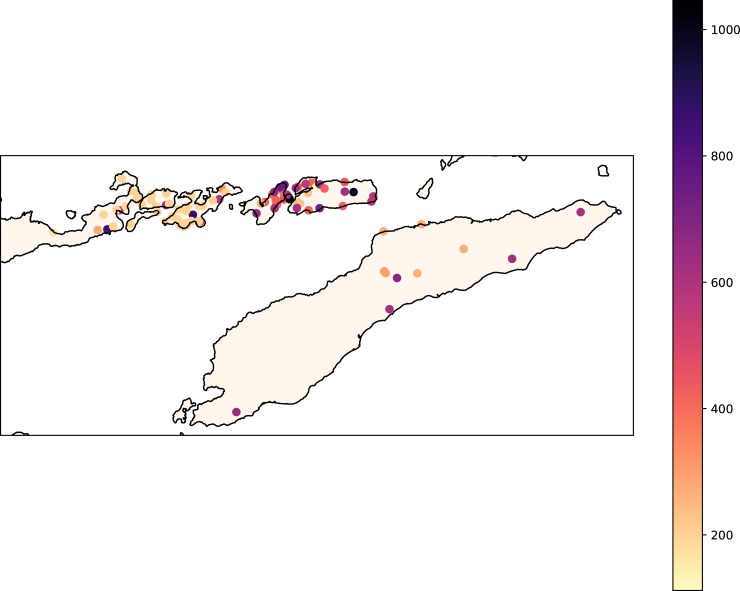
Map showing the locations of lects currently in LexiRumah. Lects are color-coded according to the number of lexical items present.

Of the resulting list of 596 concepts, 569 items are attested for more than a quarter of the languages of the database. Only 8 of the 596 concepts are available for every lect in LexiRumah, because the concept lists used by different sources differ. For example, the lists of Lamaholot dialects collected by Keraf [44] are limited to ~200 items, but while Keraf’s concept list [44] largely overlaps with the Swadesh list as we used it to sample in our fieldwork, they are not completely identical. Currently the database has a total of 38,912 word forms, from lects located as indicated in Fig 6. On average, we have 337 different concepts attested for each language.

In addition to spoken languages, LexiRumah also includes lexical reconstructions of proto-languages, to enable easy comparison between words in modern languages and their hypothetical ancestors. Reconstructions are provided for proto-Austronesian, proto-MP and proto-CMP, taken from the online etymological Austronesian Comparative Dictionary [45,46] and from the Austronesian Basic Vocabulary Database [24]. Lexical reconstructions of proto-AP and proto-TAP were taken from published sources [7,47]. The reconstructions in these sources are all based on applying the standard comparative method of historical linguistics to lexical data that was available to their authors, historical linguists, and as such are the most reliable sources we have to date.

### 4.1 Differences and overlaps with other online lexical databases

Here we list some overlaps in language coverage between LexiRumah and the other online lexical resources which contain data from the Lesser Sunda languages, and point out the differences between the word lists in these resources. Table 1 shows the numbers of concept-form pairs (‘words’) of the compared databases; if the number of lects is more than one it is given in parentheses.

**Table 1:** Number of counterparts for Lesser Sunda lects included in databases covering the region. Numbers in the table denote the number of concept-form pairs given in a database for a given language or dialect cluster. Where a database has multiple sources for the same lect, this has been reported as the size of the largest source with the number of sources (“src”), except in the case of ASJP, which has a much more uniform wordlist length and many more different lects overall.

Lects	LexiRumah	TNG.org	ABVD	ASJP
Fataluku	620	218	218	36
Klon	852 (2 lects)		284	140 (4 lects)
Makasae	615	100	195	34
Teiwa	665	357 (3 lects)		180 (5 lects)
W Pantar	658	357 (3 lects)		176 (5 lects)
Nedebang	406	116		73 (1 lect)
Blagar	3076 (7 lects)	729 (6 lects)		169 (5 lects)
Adang	1000 (2 lects)			78 (2 lects)
Kafoa	235	116		39
Kabola	409	351 (3 lects)		140 (4 lects)
Hamap	231			38
Kui	412	233 (2 lects)		77 (1 lect)
Klon	852 (2 lects)	403 (2 lects)		140 (3 lects)
Abui	2256 (5 lects)			191 (5 lects)
Wersing	1031 (2 lects)			70 (1 lect)
Kamang	620	699 (6 lects)		242 (5 lects)
Sawila	580			74 (1 lect)
Kula	> 582 (2 src)	118		34
Bunak	1641 (3 lects)	101		27
Kiramang	424			
Reta	1047 (2 lects)			66 (2 lects)
Deing	418	117		33
Kaera	616			84 (1 lect)
Oirata		233	232	32
Lamaholot	9516 (36 lects)		414 (2 lects)	72 (2 lects)
Sika (incl. Hewa)	1288 (3 lects)		473 (2 lects)	131 (2 lects)
Mambae	268		180	34
Kemak	300		176	31
Tokodede	276		185	40
Tetun-Dili	268			34
Tetun-Terik	1229 (2 lects)		>224 (2 src)	30
Kedang	910 (3 lects)		215	33
Alorese	2409 (4 lects)	235 (2 lects)		102 (3 lects)
Lakalei	276			
Idate	269			

In both LexiRumah and Transnewguinea.org ([25], TNG.org in the table) we find data from Fataluku and Klon. In both cases, the LexiRumah dataset is at least twice as large. Other word lists in the overlap between Transnewguinea.org and LexiRumah (Teiwa, Deing, West Pantar, Blagar) come from a single publication before our cut-off date. The TAP language Oirata, spoken on Kisar in the adjacent province of Maluku, is represented in Transnewguinea.org with 233 counterparts, but is not included in LexiRumah yet.

LexiRumah and the ABVD [24] overlap in that both contain Lamaholot data. However, the data in the ABVD consists of two Swadesh lists from the Lamalera and Ile Mandiri dialects, while LexiRumah contains data from 36 other Lamaholot lects. Lamaholot is in fact an extensive dialect chain containing varieties at the edges that are mutually unintelligible. Most of the Lamaholot data in LexiRumah is restricted to 200-item Swadesh lists taken from [44], but for a number of lects a list containing close to 600 items has been collected. These include Lamaholot-Lewoingu (on Flores Island), Lamaholot-Adonara (on Adonara Island), Lamaholot-Kalikasa and Lamaholot-Lerek (both on Lembata Island). Both the ABVD and LexiRumah contain Sika word lists, but the ABVD uses data from an 80-year old manuscript [48] while LexiRumah contains modern data from the Sika dialects of Maumere [49] and Tana Ai [50]. The ABVD also has data from the East Timor languages Mambae [51], Kemak [52], and Tokodede (undated fieldnotes by A. Schapper). These three sets are approximately equal in size but their sources and source locations are unspecified and the times when they were collected are old or unknown. The LexiRumah data from East Timor languages are more homogeneous, as they were all collected in one month (May 2002) by the same linguist and include metadata on source speakers and locations. For Tetun-Dili and Tetun-Terik, as well as for the non-Austronesian languages Klon, Fataluku and Makasae, datasets are found in both ABVD and LexiRumah, but the LexiRumah dataset is two or three times larger, comprising about 600 items. The ABVD also contains the same Oirata word list as Transnewguinea.org.

Turning now to overlaps between LexiRumah and ASJP [21]SJP has 40 items for each language, LexiRumah has about 600, fifteen times more. For most languages contained in LexiRumah, ASJP has at least one, often several, word lists. Languages included in LexiRumah but not in ASJP are the TAP language Kiramang and the Austronesian languages Lakalei and Idate. In cases where the data in LexiRumah and ASJP are from the same collector/source and not identical, the ASJP data stems from earlier, less detailed surveys which took place in the early 2000’s. The Intercontinental Dictionary Series (IDS) [22] has 1310 lexical entries for 329 languages in the world, but lacks coverage of Island SE Asia or New Guinea.

Austronesian lects which are covered by LexiRumah but in no other online database are Lakalei, Idate, various Alorese dialects, and the eastern Lamaholot lects of Adonara, Kalikasa and Lerek. The TAP language Kiramang is found only in LexiRumah.

In sum, a subset of the lects in LexiRumah is also found in other online lexical databases, but where this is the case the dataset per lect in LexiRumah is often fifteen times bigger, more contemporary and/or more homogeneous.

## 5 Workflow and challenges

In this section, we describe the general procedure of going from primary sources (fieldwork, in particular fieldwork with the specific goal of expanding this database, or publications by researchers inside or outside the project) to a validated entry in the database. We highlight challenges encountered in the process and give suggestions for best practices for future database collections of a similar nature.

Entries in LexiRumah may be either based on published or as of yet unpublished linguistic sources, or on survey work in the field, executed by the authors’ project members or close collaborators. Both types of data require different work flows to be included in our database and present different hurdles. Those steps are described below.

### 5.1 Compiling word lists through fieldwork

The word lists in LexiRumah have been collected through fieldwork in two different ways. One data collection method involved a longer fieldwork visit, studying one language in particular, and using frequent consultations with different native speakers to obtain an in-depth knowledge of the language. This method was used by researchers who spent several months in the field studying one language in detail. As one task of many, they also collected word lists in a few of the dialects of the language they were studying. The other collection method was through survey work. In survey work, a fieldworker, possibly with their assistant(s), visits individual language communities for a relatively short stay, typically one week, maybe two, to collect “shallow” linguistic data which does not require in-depth knowledge of the language. The aim of survey work is to sample data from various points in a region to study the linguistic diversity of that region. It also provides initial data about unstudied lects that are possibly relevant for further study. We use the term “shallow” here in contrast with the method of deeper investigation mentioned above, which would allow the researcher to also draw conclusions about the morphology and syntax of the target language, which is hardly possible from word list data collected through survey work. Survey work to elicit word lists is fraught with problems, especially in its main use case when there is limited survey time available per language, and the researcher collecting the data does not speak the target language, so that another language must be used as an intermediate language. The risk of collecting bad or noisy data further increases when only one speaker is consulted, and it can become very high when this speaker has lived away from the place where the variety is originally spoken and has not used it for extensive periods.

#### (a) Best practices for linguistic survey work eliciting word lists

For these reasons, we apply the following best practices, where possible. Eliciting word lists through survey work should (i) take place in the location where the variety is spoken; (ii) involve a small group (e.g. 3–6) of native speakers who feel confident about their language and speak the same lect with each other on a daily basis; (iii) involve native speakers who have sufficient time for compiling a word list which they consider to be representative of the consensus forms used in their local lect; (iv) involve a linguist who has in-depth knowledge of at least two languages that are spoken in the region covered by the survey, but which are genealogically and/or geographically remote from each other. Furthermore, (v) the elicitation word list should not provide only a single word in a gloss language, but give a clear definition of the meaning to be elicited, and (vi) specify criteria which word(s) to include if multiple words can be used to express that meaning, either because they are synonyms, or because each of them is more specific than the meaning requested.

#### (b) Materials and protocol for lexical survey work

For lexical survey work, we use the following materials and protocol. Apart from the use of Indonesian as gloss language, this procedure is not specific to the Lesser Sunda Islands and should serve to obtain high quality transcribed word lists where lexical survey work using a *lingua franca* is possible.

Elicitation is in Indonesian, the national language of education, media and government, and spoken by virtually everyone as a second language. An original word list compiled in English is translated into Indonesian by a native speaker of Indonesian. A “notes” column on that list provides extra information for the linguist about Indonesian prompts that often raise questions and/or need some extra clarification.

Before the first compilation stage, the linguist goes through the word list and the notes to familiarize him/herself with what is going to be asked. Then several speakers of the local variety are invited who are willing and able to translate the Indonesian words in their own language, and have sufficient time for the task. The linguist and the speakers work through the list. When the speakers have reached a consensus about which word is the best translational equivalent of the Indonesian prompt, the linguist repeats this word until his/her pronunciation of it is accepted by the speakers, and writes it down in International Phonetic Alphabet (IPA). In the Lesser Sunda region, where we did dozens of lexical surveys in remote field sites, we worked with fluent native speakers who live in a stable social context with few distractions. In such situations the elicitation of a list of 600 words (using Indonesian prompts) takes at least half a day, but can also last one or two days. The speed of collection might differ depending on the region where a survey takes place, the fluency of the speakers, and/or their cultural context.

After the first compilation stage is finished, the linguist fills in a new (blank) list with the local words, now using Indonesian orthography (not IPA). This is done so that a local speaker will be able to read the word that has been written down. A second appointment is made to audio/video record the word list.

On this second appointment, the copy of the word list with the words in Indonesian script is given to one native speaker who is willing to be recorded. An informed consent form is filled in. The linguist/assistant keeps the list with the IPA transcriptions. On the recording, the linguist / assistant reads out the Indonesian word once, and the speaker repeats this word twice in his local language. The speaker has the written word list in front of him/her as a reminder, but if s/he feels uncomfortable about reading the words of his language, the linguist/assistant sits next to him/her to assist when necessary. The linguist/assistant checks if the response uttered on the recording is the same as the word that was written on the list. If there is a difference, the speaker is invited to comment on the difference. Usually the difference is due to a transcription error by the linguist, or an erratic choice of words by the recorded speaker (e.g. because of the pressure felt by being recorded). Speakers who are comfortable reading their language will often note small errors in the way the linguist captured the words in written form (e.g. an [n] should be [ŋ], a final glottal stop should be an unreleased [k], etc.). These transcription errors are corrected during the recording. In this way, the recording session provides a recording of the word list as well as a transcribed word list that has been double-checked by a speaker. In our region, none of the lects under consideration have a standardized written variety, so it is unlikely that speakers correct natural to more literary forms, a problem that might occur for languages with established prescriptive traditions.

Immediately after the recording is finished, backup copies are made and put in different places. At a later stage, the recording is transcribed. For the final transcription, the linguist listens back to the recording and transcribes what is heard, whilst consulting the list that was already written in IPA in the compilation stage and had been corrected during the recording. This third check is to ensure that the words that are transcribed to be entered into the database indeed reflect what has been discussed and recorded.

#### (c) Issues encountered in lexical survey work

The following are some issues that arise in lexical survey work. Being aware of these issues means they can be addressed in the elicitation process or at least annotated in the database. If these issues are not addressed, the data will make it harder to find cognate forms necessary for historical reconstruction, such that the lects under consideration appear less related than they actually are.

First, speakers may translate the Indonesian word differently in their own language, because the Indonesian word is already polysemous. For example, Indonesian *susu* refers to ‘milk’ or ‘breast’; and the word *sempit* ‘narrow’ in Indonesian can mean ‘narrow’ (road), ‘crowded’ (house), or ‘tight’ (clothes). In elicitation it must thus be specified what the target is.

There may also be words in Indonesian with a meaning that is too generic to be translatable. For example, the Indonesian general preposition *di* ‘at, in’ often does not have an equally general counterpart in the target language, so speakers will provide a semantically more specific adposition; or, in case the language does not have adpositions, give an expression that contains a locational verb (‘be at’).

Third, the target language may have more than one translational equivalent for the Indonesian prompt, e.g. *pukul* ‘to hit’ may render different lexemes for e.g. ‘hit (a drum)’ and ‘hit (a dog)’.

Finally, not all Indonesian words have a translational equivalent in the target language. For example, causal conjunctions such as Indonesian *karena* ‘because’ are not directly translatable into Alor-Pantar languages because causal relations between clauses are not expressed with conjunctions in these languages. Or languages lack a word for a particular concept, e.g. *murah* ‘cheap’ may be translated with various expressions such as ‘low price’, ‘short price’, ‘light price’ or ‘price go down’.

Issues of translational non-equivalence and polysemy will always cause word lists to have unclear or incomparable data. We tried to minimize the amount of such “noise” by applying best practices and a uniform protocol described above.

While the lects under investigation may have a higher morphological complexity than the Indonesian used for elicitation, this does not constitute a problem of lexical survey work. Just like English verbs might include the particle *to* in their citation form, eg. Abui inalienable nouns in our word lists are cited with possessor prefixes. The choice of citation form for various word classes appears consistent within each word list and such morphemes are marked where known.

### 5.2 Transcription

Transcription of the recordings of the word lists are all in broad International Phonetic Alphabet (IPA). ‘Broad’ IPA transcription uses all basic IPA vowel and consonant symbols, and indicates (primary) stress, nasality and segment length. It does not mark aspiration, centralization, dentality, roundedness, and breathiness with diacritics. Transcriptions are phonemic for the languages for which we know the phoneme inventory; otherwise they are in broad IPA but phonetic. The optimal situation is such that the linguist who transcribed the recording was also present at the compilation and recording of the word list, and has made written notes of the earlier sessions. In a few cases, for reasons of time constraints, survey work and/or transcriptions were done by relatively less experienced students. Their transcriptions have been checked against the original recordings and, where necessary, corrected by a linguist with significant experience in fieldwork and the phonological typology of the region. Of 59 fieldwork sources, 5 were checked or transcribed by a different person than the collector and marked as such in their metadata.

### 5.3 Extraction from published sources

In contrast to survey work, where word forms are extracted orally and then written down, manuscripts and published sources require a first step to extract word forms from an already written source. Some publications, such as [44] and the word lists in the Comparative Austronesian Dictionary [53] are not available in digital form, so the word lists need to be manually converted into a computer-readable format. Other publications, such as the Austronesian Comparative Dictionary [46], are digital in principle, but are not presented in a format that makes extracting word lists (or even navigating the data) a simple, automatizable process. Collecting word lists from such sources is also a diligent, potentially error-prone procedure.

Judging from our experience with these tasks, we suggest that the extraction of word lists from existing sources works best when the person doing the work is known to be very meticulous, and also has some background knowledge of the languages spoken in the area.

Where a source cites multiple variants, synonyms, or ambiguous translations (e.g. a separate word for ‘small frog’ vs. ‘large frog’), each word form is in a separate row of the Form table. The comment column may give additional information about the relationship between the forms.

### 5.4 IPA cleanup

Both transcriptions from published sources as well as survey data collected by fieldworkers may not always closely follow IPA. An often occurring point of deviation, in particular among European, American and Indonesian fieldworkers, is the use of <j> for the voiced palato-alveolar affricate [d͡ʒ] or palatal voiced stop [ɟ], and the use of <y> for the palatal glide [j]. When this is done consistently (which can be seen from the absence of <ɟ>, <d͡ʒ> and variants thereof in the transcriptions, and from the use of <y> exactly where a consonant [j] would be expected), a simple two-step string replacement can resolve the issue. If audio recordings of the collection sessions are available, they can also shed light on the issue.

There are therefore separate columns in the CLDF wordlist for forms as given in the source, and for segmented data. The sound segments are given in CLPA normalization (https://github.com/clpn/clpa)–CLPA describes a normalization of IPA strings, which can be understood by other programs also subscribing to that standard.

### 5.5 Checks by native speakers

The NorthEuraLex database [54], which is of similar size, but aggregates data based on published dictionaries, includes a step having many entries routine-checked by a native speaker, and additional checks for forms flagged as surprising by non-native speakers. A step like this is not feasible for our database in general, especially when it concerns data that was compiled from published sources or was done by fieldworkers.

However, recall from the discussion above that the methodology of our own surveys involved two cycles designed to incorporate feedback from, and control by, native speakers. In a few cases, remaining suspicious forms could also be corrected by consultants the fieldworkers had maintained contact with. As an online publication with a public versioning history, it will also be easy to transparently incorporate future feedback from native speakers into later releases of LexiRumah.

### 5.6 Translation into Cross-Linguistic Data Format (CLDF)

The first word lists, originating from previous projects of the research group, were maintained as unversioned Microsoft Excel files. In order to conform to emerging standards for lexical data and to make version control and tracking changes of the whole project manageable, the data was translated into comma-separated Cross-Linguistic Data Format (CLDF, http://cldf.clld.org/) and placed in a Git (https://git-scm.com/) version control repository mirrored on Github (https://github.com/). This translation was done using a custom-written Python script, which was simplified and its input format standardized. The current input template and translation script are available through the LexiRumah Git repository. A project member checked the resulting CLDF files (the editing steps are reflected in the Git repository) and manually converted the previously unstructured source and collection metadata into the BibTeX format with additional standardized fields for fieldwork metadata before further processing.

As described in Section 3, the data is split into multiple tables to avoid redundancy. Data about the lects is stored in a central table, as are the meanings. It should be noted that the concepts in different source word lists are not completely identical. Where synonyms from different sources were merged, an entry in the comment field of the concepts table lists the original gloss from the deviating source. The link to Concepticon enforces the disambiguating comments for elicitation given in the annotation task and makes the specific concept referred to transparent for external users of the data.

### 5.7 Editing CLDF using other software

The CLDF standard is based on delimiter-separated files (CSV or TSV), which are easily read and written by a variety of data analysis tools, from spreadsheet applications (e.g. LibreOffice Calc) and text editors (e.g. Notepad++) to programming languages (Python, R, Matlab) and statistical tools (SPSS, Weka). In order to make editing CLDF datasets easier, the CLDF developers have also published a set of command line tools packed with the pycldf (https://github.com/glottobank/pycldf) Python library.

Unfortunately, the most widespread spreadsheet application, Microsoft Excel, is itself incapable of supporting standard text-based data exchange formats (in particular, it does support comma-separated files, but has problems with Unicode UTF-8 encoding, which is the standard of CLDF and other data guidelines, such as the W3C’s tabular data vocabulary, http://w3c.github.io/csvw/metadata/#tables). In order to edit CLDF files directly using Excel, it would therefore be necessary to provide a separate copy of the repository, which uses automatic conversion between CLDF and a format which MS Excel can natively read and write. Other office packages, such as LibreOffice, do not have this problem.

Using a very simple conversion script (included in the Git repository), which aggregates the data concerning each word form from the different CLDF tables, it is easy to edit CLDF data using standard tools for computational historical linguistics, such as the LingPy [41] suite for historical linguistics data operations (http://lingpy.org), or the Edictor [55] web-application for cognate coding (http://edictor.digling.org).

### 5.8 Similarity coding

In order to provide initial groupings for seeding bottom-up historical reconstruction work, we used automatic lexical comparison approaches [56] to group the forms in the database into lexical similarity classes. We used the LexStat [57] method with its default sound class model (known as “SCA”) for examining the similarities between word forms and Infomap [58] for selecting groups of forms with high similarity scores between them, with automatically inferred threshold. These state-of-the-art algorithms are available in the LingPy [40,41,59] open-source software package. The forms within each cognate class were then aligned using the multi-alignment procedure, also from LingPy [41,60].

LingPy is a very convenient software package which provides state-of-the-art algorithms for computational historical linguistics. The current version (LingPy 2.6.3) is able to parse a small subset of CLDF, but has very strong assumptions about the types of files it can read and the possible operations to be executed on data. It is therefore necessary to convert our CLDF to make it accessible through LingPy, execute the clustering algorithms, and merge the results back into our CLDF. The output file generated by LingPy contains several columns with annotations of the data (e.g. sound classes and scores for the importance of phonetic segment). However, access to other intermediate results of the algorithm, such as the similarity ratings between different phonetic segments or the graph that underlies the reported cognate clusters, is very complicated. Yet such access would be useful to obtain and check the algorithm’s estimations of which sounds are similar, and construct the alignments on that same empirical basis.

### 5.9 Conversion into SQLite for the online interface

The web interface of LexiRumah, developed on the basis of the open source LexiBank CLLD application (https://github.com/clld/lexibank), serves the data from an SQLite relational database (https://sqlite.org/). This database is generated from the CLDF data using a custom script, which is included in pylexirumah.

### 5.10 External access

Our data is served through a CLLD web application. The web application always serves the most current release (with a new release published about twice a year) of the data, both in the shape of an easy-to-navigate web interface and as downloadable source of the entire data in CLDF format. The data is published under an open Creative Commons 4.0 Attribution license, and old versions, including a history of all gradual changes that happened between releases, are available in CLDF format through Github.

### 5.11 Versioning and backups

In order to enable scrutiny of the processes that led to the data presented in the end, and to make older versions of the data available eg. for reproduction studies, most guidelines for data management (e.g. [61], which provides a good starting point for the topic in general) suggest using back-ups and, where possible, dedicated version control software. The CLDF is specifically designed around text formats, among other reasons to support easy versioning. Consequently, the data is maintained in a distributed version control repository using Git. One copy of the repository is stored on the project’s institutional network storage, which is professionally maintained and routinely backed-up by the university’s IT services. A central copy is kept and updated with the Github repository hosting provider, which consequently also functions as incremental off-site backup.

In addition, the current release of the data has been archived at Zenodo (DOI 10.5281/zenodo.1164783), the open science data archive provided and funded by CERN, OpenAIRE and the EU’s Horizon 2020 programme. Future data releases will also be archived on Zenodo, the process is requires very little manual work due to the excellent integration between Github and Zenodo. The most current release will always be available through the DOI 10.5281/zenodo.1164782.

### 5.12 Infrastructure for incorporating future contributions, changes, and corrections

We welcome future additions of data sets on the Lesser Sunda languages and their neighbours, with their metadata. As LexiRumah is a secondary source, the originals of unpublished manuscripts and fieldwork data included in it must be publicly accessible, either online (e.g. in electronic archives) or in printed form (e.g. grammars, dictionaries).

Using existing scripts in the creation of the current dataset, as well as standard tools to deal with wordlist-structured data, new sources can be easily integrated into the database, provided they use a compatible way of identifying the concepts they encode.

All corrections, contributions and other changes will be immediately visible in the Git version history of the repository. New releases, which will be reflected in the online database, will occur at least twice a year while the database is actively funded and curated, with additional releases after significant changes or additions of new lects.

## 6 Example use case

Using the language of Alorese as an example, we shall now illustrate of how this database can be used in historical linguistics to study the historical signals of borrowing and retention as attested in the lexicon of an individual language. Alorese (AL) has some 25,000 speakers living in pockets along the coasts of the islands of Pantar and Alor as well as on the islets in their vicinity [62–64]. It is currently in contact with the TAP languages surrounding it. AL is a variety of the Lamaholot (LH) language/dialect chain, which is spoken by some 180,000 people [64] living on the eastern tip of Flores and neighbouring islands (Fig 1), including Solor, Lembata and Adonara. Within the LH clade, AL is generally grouped together with the lects of Western LH, cf. http://glottolog.org/resource/languoid/id/alor1247 in Glottolog [39]. Together with its sister languages Sika and Kedang, the LH cluster forms the Flores-Lembata (FL) clade.

It is known from oral history that speakers of a LH variety migrated eastwards to Alor and Pantar around 1300–1350 CE [62,65]. Investigating the lexicon of AL for historical traces is interesting because it is spoken in areas non-contiguous with its closest relatives, and it is the only Lamaholotic language currently in contact with Timor-Alor-Pantar (TAP) languages.

Here we study the structure of the AL lexicon on the basis of the following research questions: (i) What is the influence of TAP languages in the AL lexicon? (ii) To what extent does the (non-TAP) lexicon of AL look like an Austronesian (Malayo-Polynesian, MP) language? and (iii) Which part of the lexicon is unique for AL?

We show, using an interactive IPython notebook (which can be found in Supplementary Material 2), how the pylexirumah Python package, and the automatic similarity codes described in Section 5.8, can be used to study these questions.

**Fig 7:**
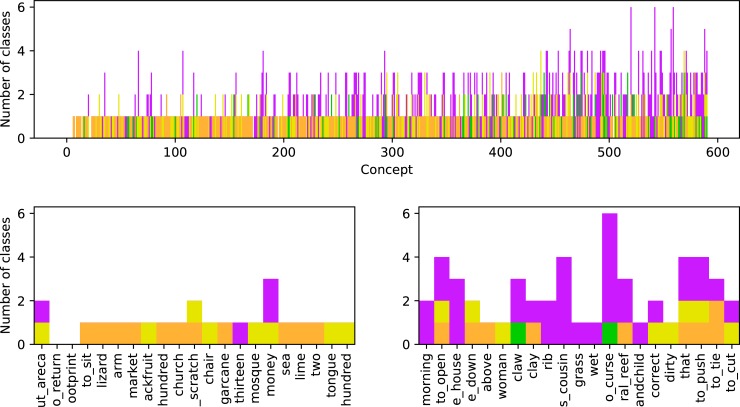
Vocabulary pattern plots for the Alorese lects in LexiRumah. **(A) All 596 concepts in the database, sorted along the *x*-axis by their number of cognate classes across all lects. (B) Concepts 20–30 and (C) Concepts 530–550 from (A).** Every unit bar represents one similarity class in the database attested in at least one AL lect. Similarity classes are coloured according to their distribution in different language groups: similarity classes shared only with TAP languages are green, concepts distributed as if they have a hierarchical history of inheritance are orange, and concepts with classes that are unique for AL are purple. Classes marked yellow have a complicated distribution. Close-ups are provided of concepts 20–30 from the more stable half of the plot (Fig 7B) and of number 530–550 from the less stable half of the plot (Fig 7C).

For each similarity class in LexiRumah, we aggregate for five groups of lects whether the cognate class is attested in any lect of that group. The groups are (1) AL, (2) the LH lects except for AL, (3) the FL languages (Sika, Kedang) except for the LH lects, (4) the Austronesian (Malayo-Polynesian) lects that are not FL languages, and (5) the TAP lects. We can then visualize the composition of the aggregate vocabularies of these lects, similar to admixture plots in genetics, as shown in Fig 7. These plots were generated in the IPython notebook, and can be adapted to other language groups and cognate class patterns of interest.

Fig 7A shows the composition of the AL vocabulary; the *x*-axis corresponds to the 596 concepts in the database, sorted by their number of cognate classes across all lects, which we take as an approximation of lexical stability–words with fewer different cognate classes are presumably more stable.

In Fig 7, concepts with similarity classes that are shared with TAP lects, but not with Austronesian lects are green, classes that are unique for AL are purple. The remaining classes are coloured according to whether their distribution looks like they have a hierarchical history of inheritance: orange is used for classes that do, because they are (a) present in LH and AL, but in no other lects; or (b) present in at least some wider FL lects and in at least some LH lects and in AL, but nowhere else; or (c) present in other Austronesian lects, as well as in FL languages, LH and AL–independent of whether they are found in TAP lects or not. An example of (c) is the “hierarchically inherited” similarity class (orange) of ‘lizard’: all our attested forms for ‘lizard’ in AL (AL-Pandai [taˈkːe], AL-Munaseli [təˈkːɛʔ] and AL-Alor-Besar [taˈkːe]) are similar, and similar forms are also attested in, for example, LH-Lerek [təˈkɛk], Sika [təkeː], and proto-MP *təktək, as well as in most TAP languages (eg. Abui-Fuimelang [tekok]). Presumably, the proto-MP form got borrowed into proto-TAP and was inherited by daughter languages in each family.

Note that the orange class is only an approximation of “hierarchically inherited”: a recently introduced concept like ‘church’ also shows the distribution of an inherited word, because forms like AL-Alor-Besar [gaˈrɛd͡ʒa], borrowed from Indonesian *gereja*, are widespread across all groups.

Yellow is used for all other similarity classes, indicating a complex history of inheritance and/or borrowing. This includes all forms that “skipped” a clade in the inhertance hierarchy (and thus do not have the “hierarchical inheritance” pattern shown by the colour orange). For example, this includes classes found in AL and other Austronesian lects which are not attested in any of the LH lects. (This would not be immediately expected under the hypothesis that AL is a daughter, not a sister, of LH). It also applies to forms found in TAP, but which among our Austronesian languages are attested only in the wider FL languages.

The latter, cross-family, distribution cannot be explained by the two contact situations known to us, where AL borrows from TAP languages and proto-TAP borrowed forms from proto-MP. A more in-depth analysis of these forms is necessary to distinguish between (i) TAP forms borrowed into FL languages, (ii) forms that originated in FL languages but have been borrowed into TAP lects possibly through the mediation of AL, and (iii) recent loans that spread through the Flores-Lembata-Alor region, but not attested in our other Austronesian languages. We present an example containing such an analysis after discussion of Fig 7.

The height of the bars in Fig 7 indicates the number of similarity classes in AL. For example, in Fig 7B, for the concepts ‘footprint’ and ‘to return’, we lack data in AL, so no bars are shown. All other concepts in that subfigure, which have a bar height of 1 or more, are attested in AL. An example of a bar height of 1 is the concept ‘lizard’ mentioned above, where all our attested forms in AL (AL-Pandai [taˈkːe], AL-Munaseli [təˈkːɛʔ] and AL-Alor-Besar [taˈkːe]) are similar. An example of a bar height of 3 is the concept ‘claw’ in Fig 7C. The similarity of AL-Pandai [kˈrako] and even more AL-Alor-Besar [gaˈraku] with Bunak-Bobonaro [galakha] places this similarity class in the green category of forms possibly borrowed from TAP languages, while the other two classes (AL-Munaseli [kɛla ˈleiŋ] and AL-Baranusa [limɑŋ taˈnuŋgul]) are not similar to any other forms in the database and therefore unique to AL, and purple.

Overall, Fig 7A shows that all four of these categories (purple, green, orange, yellow) are present throughout the entire lexicon, and that innovations unique to AL (purple) make up a far larger part of the lexicon than apparent borrowings (green), although examples like ‘church’ mentioned above show that this overall picture reflects general trends, but is misleading in the details.

The IPython notebook also shows how to systematically compare lexicons on a more detailed level. This is relevant, because the plots do not give us detailed insight into inheritance patterns, as when they collapse hierarchically inherited forms with wide-spread borrowings (orange, cf. ‘church’) or when they collapse different reasons for “skipping” a clade in the inhertance hierarchy (yellow); ‘blood’ discussed below is an example of the latter. We systematically compare the lexicon of AL and LH, focussing on the most stable vocabulary where differences are most relevant to the history of AL.

The first most stable 25 concepts (cf. Fig 7A) where AL differs from LH are ‘roof rafter’, ‘some’, ‘dew’, ‘fist’, dibble stick’, ‘betel vine’, ‘stem’, ‘corn/maize’, ‘hand’, ‘ten’, ‘betel nut/areca’, ‘other’, ‘money’, ‘to scratch’, ‘thirteen’, ‘to return’, ‘jackfruit’, ‘mosque’, ‘footprint’, ‘chair’, ‘seventeen’, ‘two’, ‘sea’, ‘fifteen’, ‘blood’.

For some of these very stable concepts (e.g. ‘roof rafter’, ‘some’, ‘dew’), we have word forms only in LH but not in AL. This does not allow us to say anything on the history of these forms. For the other concepts, which have different word forms in AL and LH, we find various explanations for why they are different.

For ‘hand’, most LH lects, including AL, have forms similar to [ˈlimaŋ], a form which is widely shared also outside the group and goes back to proto-Malayo-Polynesian *qalima. However, LH-Adonara has a form [ˈnaiʔjũ] that is not similar to any other counterpart for ‘hand’ in LexiRumah, suggestive of a lexical innovation. A similar situation occurs for ‘betel vine’ and ‘sea’.

For the concepts ‘ten’, ‘thirteen’, ‘seventeen’ and ‘fifteen’, the AL and LH forms are dissimilar, but the AL forms show similarity with the forms of the neighbouring Timor-Alor-Pantar languages, having apparently borrowed a reflex of Proto-TAP ‘ten’ *qar-. The forms for ‘jackfruit’ also show a split where the AL lects adopted a loan from TAP languages.

In the forms for ‘betel nut’, an accidental split in classes has happened due to apparent metathesis: in some LH lects (LH-Lewoingu, LH-Adonara), words for ‘betel nut’ are [wua], reflecting ancestral proto-Malayo-Polynesian *buaq ‘fruit’ (in reconstructed proto-MP forms, <q> stands for a glottal stop /ʔ/). AL-Baranusa [ʔufa] and Kedang [uwe] on the other hand, are apparently metathesized and put in a different class from the unmetathesized forms in LH.

The words for ‘money’, a relatively recent concept, show a range of different forms of apparently different origins: in LH the form [doi(t/r)] is used, from 17th Century Dutch *duit* ‘coin worth 1/8 of a *stuiver* (penny)’. In AL-Alor Besar and AL-Pandai the word for money it [sɛŋ], from Dutch *cent* ‘coin worth 1/100 of a guilder’. AL-Munaseli has two forms for ‘money’, both of them apparently innovated. Similarly, the forms for ‘chair’ have different donor languages: *kursi* from Indonesian, *cadeira* from Portuguese.

For ‘to scratch’, the similarity classes suggest that AL and a few LH lects (LH-Pukaunu, LH-Waiwadan, LH-Ile Ape, LH-Watan–which is not a recognizable geographic or phylogenetic cluster) reflect ancestral proto-MP *kaRaw (e.g. [garu] in LH-Watan), while all other LH lects are in a group of [ragu] forms, suggesting metathesis.

The words for ‘mosque’ are two arbitrary split classes, both similar to [mɛs'ɟid]; and the fact that they are split into two classes is an artefact of the clustering algorithm we used. The same happened for the concept ‘two’, with forms [ʤua] and [rua] in different classes.

Lastly, for the concept ‘blood’, all LH lects and the neighboring Austronesian languages of Sika and Kedang have forms like [mei] (which the algorithm grouped with [wei] forms in TAP languages, possibly for their only superficial similarity), whereas AL [ra] appears to reflect proto-MP *daRaq, shared with other Austronesian languages of the region, but not with any TAP languages. This is a lexical retention in AL which needs to be explained in the context of the AL emigration from LH-speaking areas in the 14th Century. It suggests that the ancestor language of both AL and LH contained a reflex of proto-MP *daRaq which was retained in AL but replaced with [mei] forms in all LH lects.

In sum, using even a very basic quantitative analysis of some data available in LexiRumah with its noisy automatic similarity codings has nonetheless enabled us to uncover several instances of lexical innovation, possible loans, metathesis, and a surprising case of lexical retention. The findings suggest that the history of LH has some complexities that are not trivially congruent with the grouping of AL originating as a Western LH dialect. AL has undergone unique changes after the split, but has also retained ancestral material that is apparently not retained in any other LH dialects. We therefore expect that a more sophisticated study using LexiRumah would help to uncover relevant facts relating to the history of the region.

## 7 Discussion

Creating a database such as LexiRumah has become significantly easier in the last decade, and hardly comparable to the first computer-readable lexical databases such as the Indo-European database on punched cards generated by Dyen in the 1960s.

Nonetheless, creating a lexical database that is easy to re-use, inspect, and browse, for the lexicon of language diversity in under-studied areas of the world, currently still requires linguistic as well as computational expertise. In the coming years, we expect the threshold of necessary programming skills to go down, and the computing literacy in the linguistic community to go up, such that it will become ever easier to construct and combine lexical databases.

Given this development, it is useful to consider the state of the art and look at the lessons learned from building, maintaining, and using existing databases, such as LexiRumah.

When building a lexical database, it is important to establish protocols for collecting and handling the data during its journey of getting from a speaker’s mind or an external author’s publication into the form that will enter the database. We have outlined our protocols and procedures above. Our most important takeaway messages are listed below.

### 7.1 Do not remove data

Data and metadata may be hidden if it appears no longer useful, but should never be completely unavailable. This includes an obligation to archive recordings from fieldwork, intermediate worksheets, and to keep a history of changes that lead to the version of data that is ultimately published. This allows anyone using the data to re-visit earlier decisions transparently. If data is of severely questionable quality and would have been removed in times when computer memory space was more scarce, we suggest to not remove it but instead marking it as ‘unreliable’.

### 7.2 Keep thinking linguistically

Once data is in a computer-readable format, it may be tempting to some to hand the data to more computationally-minded collaborators and have them apply their methods. It is to be expected, however, that the raw data collected will contain several issues that will still need linguistic consideration. This can relate to matters of orthography, informant sociolinguistics, decisions on synonyms in glosses and target languages, how to deal with recurring morphemes (e.g. the systematic prefix *ta-* to mark possessors of inalienable nouns in one of the languages, Abui), or a multitude of other matters.

Some of these issues may only show up while trying to analyse the data. It is therefore important to continue the qualitative study of the data even when it is being used for quantitative purposes.

### 7.3 Use appropriate protocols of data collection, tools, procedures and standards

Because the field of linguistic databases is still very young and the methods used for linguistic data collection are not always explicit, it is often unclear how to evaluate the quality of the data found in these databases. Explicit descriptions of methods and protocols, as well as a sufficient amount of metadata will increase the usefulness of such databases. New tools have been created (and sometimes discarded) quite frequently in the last years. Recently, new databases and tools are more and more converging towards a consistent structure of delimiter-structured tabular text data, which can be easily manipulated using both generic data manipulation software (including many spreadsheet applications) as well as specific tools for working with linguistic data. The CLDF standard is used for a wide range of other important databases ranging from lexical (e.g. NorthEuraLex [54], WOLD [37], and ASJP [21]) to structural (e.g. WALS [66]) and further afield (e.g. PHOIBLE [67]). Some of these databases were originally using other data formats, which shows that this standard is not crystallizing for lack of alternatives, but because it has proven useful for a variety of editing and analysis needs.

### 7.4 Ensure credit where credit is due

If databases are not seen as scientific contributions of their own right, or if databases neglect to make their sources visible and easy to cite, there will be disincentives for creating and contributing to databases. If it is however easy to re-use the data *and* cite the database as well as the individual sources that contributed to the results of an analysis, the ecosystem of data collection, aggregation and analysis will be able to flourish and grow, without leaving any of the contributors behind.

## 8 Outlook

If the lessons we learned from constructing our database can be transferred to other, similar efforts, we are looking forward to a strong academic ecosystem of open-access databases and tools. This is sure to give an exciting boost to research in the history and dispersal of languages, peoples and cultures. We will continue to use our own database in this context, improving the cognate annotation using tools like LingPy [41] and Edictor [55] and feeding into computational analyses using Beastling [68] and BEAST [69].

To ensure the re-usability of our data also in other contexts, we need to take our own advice to heart and continue our efforts to make all our sources, including fieldwork recordings, available through accessible archives. Many of the original recordings are archived in The Language Archive (http://hdl.handle.net/1839/00-0000-0000-0018-CB72-4@view), and we strive to have every individual form in LexiRumah linked to a media file or place in a publication which serves as its reference. Other recordings are going to be archived and linked in the near future. While all metadata is currently kept together with the data, making it available for further analyses will require better structuring of the data, e.g. for connecting speakers’ sociolinguistic profiles to the sources they appear in.

To capture the fact that not all words in the lists are equally reliable, we will provide reliability scores for each of the lexical items. For example, words for concepts like ‘breast’ or ‘narrow’ which are polysemous in Indonesian (*susu* ‘milk, breast’; *sempit* ‘narrowcrowded tight’) would get a lower reliability scale than translations of words that are not polysemous in Indonesian, e.g. *laut* ‘sea’ or *langit* ‘sky’. Further factors influencing the reliability of lexical items are language external. For instance, because of time and funding constraints some of the survey work was done by less-experienced students, and in some cases, the only native speakers we could consult had been living away from their original home town for a certain period. Such factors also lower the reliability score of the word form collected.

However, for the purpose of unravelling the history of the Lesser Sunda region, the goal of our project, our dataset needs to also grow in breadth. Our data content concerning the island of Timor and its surrounding islands is still incomplete, and data from lects of neighbouring islands East of Timor and West of Central Flores are yet missing. We welcome additional data sets on the languages of the region.

In order to assemble a cleaner picture of the history of the region, we have also begun assembling structured data of the same languages that is not lexical. Typological features collected from grammars published about the languages of the region will form the content of the GramRumah database, while cultural features, collected from local informants in a structured questionnaire, are used for CultureRumah. In the construction of these two databases, we will in turn learn from the challenges of other typological databases currently in construction (e.g. GramBank [70], D-PLACE [71]). Together with an expanded LexiRumah, and knowledge gained from the analysis of other linguistic databases describing other regions of the world, we are confident that qualitative and quantitative analyses of this data will provide us with a clearer picture of the contact-rich history of Papuan and Austronesian peoples settling the Lesser Sunda region.

## Supporting information

S1 File Data archive. ZIP-Archive containing CLDF dataset and pylexirumah software package.
(ZIP)

S2 File Program code. ZIP-Archive containing the IPython notebook used for the analysis in section 6, both as executable notebook and as static HTML file.
(ZIP)
