# Supplementary material for: LexiRumah: An online lexical database of the Lesser Sunda Islands
Source: PLoS One. 2018 Oct 17;13(10):e0205250. doi: 10.1371/journal.pone.0205250 (PMC6192618; doi:10.1371/journal.pone.0205250)
Supplement: S2 File — ZIP-Archive containing the IPython notebook used for the analysis in section 6, both as executable notebook and as static HTML file. (ZIP) [file pone.0205250.s002.zip › AnalyzeAlorese.html]

AnalyzeAlorese


# Lexical particularities of Alorese¶

This notebook shows how to use LexiRumah to investigate the lexical differences between
two groups of languages in LexiRumah. As example, compare the lexicon of Alorese lects (alor1247) with the lexicon of all other lects of Lamaholot (lama1292) lects.

First we import a variety of necessary modules and define constants.

In [1]:

```
# Import generally useful modules
%matplotlib inline
from matplotlib import pyplot
import numpy
import itertools
from bisect import bisect
from collections import defaultdict, OrderedDict
```

In [2]:

```
# Import pylexirumah and related objects
from clldutils.path import Path
import pylexirumah
from pylexirumah.util import glottolog_clade, get_dataset, lexirumah_glottocodes

# Load the LexiRumah database
lexirumah = get_dataset()
```

We define the language groups: `alorese` will be all alorese lects in LexiRumah, `lamaholot` will contain all sedentary Lamaholot lects (spoken on Solor, Flores, Lembata etc., but not the Alorese lects of Alor and Pantar). In order to check for inheritance and cross-family borrowings, we define `an` and `tap` to be the groups of all other lects in the database, by family.

In [3]:

```
# This may take some time, our implementation of `glottolog_clade` is not built for speed.

alorese = glottolog_clade("alor1247", lexirumah)
print(alorese)

lamaholot = glottolog_clade("lama1292", lexirumah) - alorese
print(lamaholot)

floreslembata = glottolog_clade("flor1239", lexirumah) - alorese - lamaholot

an = glottolog_clade("aust1307", lexirumah) - alorese - lamaholot - floreslembata
tap = glottolog_clade("timo1261", lexirumah)

rest = set(lexirumah_glottocodes(lexirumah)) - alorese - lamaholot - floreslembata - an - tap
# There should not be any languages in the rest
assert not rest

groups = [alorese, lamaholot, floreslembata, an, tap]
```

```
{'alor1247-besar', 'alor1247-munas', 'alor1247-baran', 'alor1247-marisa', 'alor1247-pandai'}
{'lama1277-baipi', 'lama1277-lewok', 'lama1277-lewuk', 'lama1277-lerek', 'lama1277-lewop', 'lama1277-lamah', 'lama1277-kiwan', 'lama1277-ileap', 'lama1277-waiwa', 'lama1277-minga', 'lama1277-lewoi', 'lama1277-botun', 'lama1277-lewoe', 'lama1277-tanju', 'lama1277-pukau', 'lama1277-watan', 'lama1277-waiba', 'lama1277-wuake', 'lama1277-adona', 'lama1277-lewot', 'lama1277-imulo', 'lama1277-merde', 'lama1277-lamak', 'lama1277-dulhi', 'lama1277-lewob', 'lama1277-paina', 'lama1277-mulan', 'lama1277-ritae', 'lama1277-lewom', 'lama1277-belan', 'lama1277-lewog', 'lama1277-kalik', 'lama1277-horow', 'lama1277-bama', 'lama1277-lamal', 'lama1277-lamat'}
```

The LexiRumah database contains a `CognateTable`, which associates forms with cognate classes. In principle, different sources can assign one form to different classes. This is not the case at the moment where all forms are automatically generated. Going forwards, it will be a useful assumption that the newer cognate judgements occur later in the file, which is the semantics the by the following code block takes into account.

Explicitly prioritizing or ignoring a specific source can still be done by adding a filtering `if` statement to the following `for` loop.

In [4]:

```
# Aggregate cognate data from the Form and Cognate table.

# First, load the relevant content of the CognateTable into memory as dictionary.
cognateclass_by_form = {}
for cognate in lexirumah["CognateTable"].iterdicts():
    cognateclass_by_form[cognate["Form_ID"]] = cognate["Cognateset_ID"]

# Then, generate a sequence of all cognate classes, storing associations from the
# cognate class ID to the set of (concept, lect, form) tuples representing all forms
# belonging to that class.
cognates_by_class = {}
for form in lexirumah["FormTable"].iterdicts():
    concept = form["Concept_ID"]
    lect = form["Lect_ID"]
    cognates_by_class.setdefault(
        cognateclass_by_form.get(form["ID"]), set()).add(
        (concept, lect, form["Form"]))
```

In order to compare the lexical material of the two language groups, we now check what cognate classes are attested in which of the language groups. For now, all cognate sets in LexiRumah are restricted to one concept each, which simplifies the following calculation and interpretation.

In [5]:

```
# attested_cognate_sets is a dictionary, which associates concepts to a list of sets.
# The list contains one set for each language group defined above, and the set is a
# set of all cognate set IDs that express that concept in at least one lect of that
# group.
attested_cognate_sets = defaultdict(lambda: [set() for g_ in groups])
for name, cognates in cognates_by_class.items():
    lects = {lect for (concept, lect, form) in cognates}
    concept, _, _ = next(iter(cognates))
    
    for g, group in enumerate(groups):
        if lects & group:
            attested_cognate_sets[concept][g].add(name)
```

Now we can compare the lexicon. Starting with the most ‘stable’ concepts (we don't have a direct measure of stability, but we can assume that concepts with fewer cognate classes are more stable), we list those concepts where Alorese and Lamaholot have different cognate classes.

In [6]:

```
# Sort concepts by stability, by running an insertion sort with the help of bisect.
concepts = []
concept_stability = []
for concept, concept_classes in attested_cognate_sets.items():
    order = len(
        set.union(*concept_classes))
    i = bisect(concept_stability, order)
    concepts.insert(i, concept)
    concept_stability.insert(i, order)
# concepts is now a list of concept IDs, in order of stability.

cognateset_pattern_counts = numpy.zeros((2,) * len(groups) + (len(concepts),))

# Count the number of cognate classes for each pattern.
for c, concept in enumerate(concepts):
    attested_in_groups = attested_cognate_sets[concept]

    for pattern in itertools.product([0, 1], repeat=len(groups)):
        cognate_classes_for_pattern = set.union(*attested_in_groups)
        for present, classes in zip(pattern, attested_in_groups):
            if present:
                cognate_classes_for_pattern &= classes
            else:
                cognate_classes_for_pattern -= classes
        
        cognateset_pattern_counts[pattern][c] = len(cognate_classes_for_pattern)
```

# Genome-style plots¶

Every vertical line in the following bar plots corresponds to a concept; stable concepts on the left, unstable concepts on the right. Inside each bar, different types of cognate classes are plotted – every cognate class attested in that group is plotted with the same percentage, with colors corresponding to the apparent origin of the form.

In [7]:

```
def genomestyle(group_index=None, order=None):
    # Prepare the figure
    pyplot.gcf().set_size_inches((14, 3))
    #pyplot.axis('off')
    #pyplot.gca().get_xaxis().set_visible(False)
    #pyplot.gca().get_yaxis().set_visible(False)

    # Only count cognate classes present in group `group_index` – set all counts where
    # `group_index` is absent to 0.
    counts = cognateset_pattern_counts.copy()
    if group_index is not None:
        counts.swapaxes(group_index, 0)[0] = 0

    # Where `normalize` is 0, no forms for that concept are given. Ignore.
    b = numpy.zeros((len(concepts), len(colorize)))
    
    for c, (color, cells) in enumerate(colorize.items()):
        bar_height = 0
        for cell in cells:
            bar_height = counts[cell] + bar_height
        b[:, c] = bar_height

    # This complex expression sorts the bars as we want them, but keeps the data
    # structure an array for easier calculations.
    order = order or sorted(
        range(len(b)),
        key=lambda x: tuple(
            sum(b[x][n:]) for n in range(len(b[x]))))
    sorted_bars = b[order]

    bottom = 0
    for color, bars in zip(colorize, sorted_bars.T):
        pyplot.bar(
            range(len(bars)),
            bars,
            1.0,
            bottom = bottom,
            color = color)
        bottom = bottom + bars
    
    pyplot.xlabel("Concept")
    pyplot.ylabel("Number of classes")
    return order
```

In [8]:

```
colorize = OrderedDict()
```

Some cognate classes are present in Alorese and other AN languages (including the provided reconstructions of pMP and pAN). If they follow the hierarchy (see below), we give them all the same orange color. (In addition, this color is given to all hierarchically inherited forms that did not make it into Alorese, but this is only for completion's sake.)

That is, all similarity classes found in every group for one of the following diagrams is colored orange.

```
(TAP < ) AN       (TAP < ) AN                 (TAP < ) AN             (TAP < ) AN
                            \                           \                       \
             FL             FL     FL                   FL        FL            FL
                                    \                    \         \             \
                LH                  LH      LH           LH        LH            LH
                                             \                      \             \
                                             AL                     AL            AL
```

As an exception from that hierarchical inheritance color, Forms found in Alorese which are judged dissimilar to any other forms get the color purple marking them as “Alorese-specific”.

In [9]:

```
# Hierarchical inheritance
colorize[(1, 0.7, 0.2)] = [
    (1, 1, 1, 1, 1),
    (0, 1, 1, 1, 1),
    (0, 0, 1, 1, 1),
    (0, 0, 0, 1, 1),
    (1, 1, 1, 1, 0),
    (0, 1, 1, 1, 0),
    (0, 0, 1, 1, 0),
    (0, 0, 0, 1, 0),
    (1, 1, 1, 0, 0),
    (0, 1, 1, 0, 0), 
    (0, 0, 1, 0, 0),
    (1, 1, 0, 0, 0),
    (0, 1, 0, 0, 0)]
```

Similarity classes that are found in TAP, and outside TAP only in Alorese, are a priori easily explained as TAP loans, so they will all be colored green. (In the complete listing, that color is also chosen for all classes found in TAP, but not outside that family.)

In [10]:

```
# TAP forms
colorize[(0.0, 0.8, 0.0)] = [
     (1, 0, 0, 0, 1),
     (0, 0, 0, 0, 1)]
```

If a cognate class is attested in Alorese and other Flores-Lembata languages, but not in any Lamaholot dialect, that is slightly unexpected under the hypothesis that Alorese is a daughter, not a sister, of Lamaholot. We color those similarity classes in yellow.

Similarly, we assign the same yellow color to all classes that have an unexpected distribution. This includes

1. All forms that “skipped” a clade in the inhertance hierarchy, eg. similarity classes found in AN, Alorese, and other Flores-Lembata languages, but not in any Lamaholot dialect;
2. Similarity classes found in Timor-Alor-Pantar languages, but which in the Austronesian languages are found only in the wider Flores-Lembata languages.

In [11]:

```
# Unexpected distribution
colorize[(0.9, 0.9, 0.0)] = [
    (1, 0, 1, 1, 1),
    (1, 0, 1, 1, 0),
    (1, 0, 1, 0, 0),
    (1, 0, 0, 1, 1),
    (1, 1, 0, 1, 1),
    (0, 1, 0, 1, 1), 
    (1, 0, 0, 1, 0),
    (1, 1, 0, 1, 0),
    (0, 1, 0, 1, 0),
    (1, 1, 1, 0, 1),
    (0, 1, 1, 0, 1),
    (0, 0, 1, 0, 1),
    (1, 1, 0, 0, 1),
    (0, 1, 0, 0, 1),
    (1, 0, 1, 0, 1)]
```

In [12]:

```
# Alorese forms
colorize[(0.8, 0.1, 1.0)] = [
    (1, 0, 0, 0, 0)]
```

In [13]:

```
# This should be all combinations.
all_patterns = [(0,) * len(groups)]
for patterns in colorize.values():
    all_patterns.extend(patterns)
for i, j in zip(sorted(all_patterns), sorted(itertools.product([0, 1], repeat=len(groups)))):
    if i!=j:
        print("expected:", j, "but found:", i)
```

# Genome-style plot of the entire database¶

The plots below sort concepts by their local stability. If you want a specific order, eg. order by global stability, you have to calculate that order and pass it to those plotting functions as argument. Here, the output is the distribution of these classes over the entire database.

In [14]:

```
order = genomestyle(None, order=None)
```

## TAP forms¶

In [15]:

```
_ = genomestyle(4, order=None)
```

## Alorese forms¶

In [16]:

```
_ = genomestyle(0, order=None)
```

In [17]:

```
_ = genomestyle(0, order=order)
```

In [18]:

```
concepts_in_order = [concepts[o] for o in order]

pyplot.subplot(1,2,1)
_ = genomestyle(0, order=order)
pyplot.xticks(range(len(concepts)), concepts_in_order, rotation='vertical')
pyplot.xlim(19.5, 40.5)
pyplot.subplot(1,2,2)
_ = genomestyle(0, order=order)
pyplot.xticks(range(len(concepts)), concepts_in_order, rotation='vertical')
pyplot.xlim(529.5, 550.5)
pyplot.ylabel("")
```

Out[18]:

```
Text(0,0.5,'')
```

In [19]:

```
_ = genomestyle(0, order=order)
pyplot.xticks(range(len(concepts)), concepts_in_order, rotation='vertical')
pyplot.xlim(19.5, 80.5)
```

Out[19]:

```
(19.5, 80.5)
```

## Examples: a few of the concepts seen in the figures above¶

In [20]:

```
for c in attested_cognate_sets["lizard"][0]:
    for group, lects in zip(("AL", "LH", "FL", "AN", "TAP"), groups):
        print(group)
        for concept, lect, form in cognates_by_class[c]:
            if lect in lects:
                print(lect, form)
```

```
AL
alor1247-munas tə'kːɛʔ
alor1247-pandai ta'kːe
alor1247-besar ta'kːe
LH
lama1277-kalik təˈkɛk
lama1277-adona ˈtɜke
lama1277-lerek təˈkɛk
lama1277-lewoi ˈtəkɛʔ
FL
sika1262-hewa təke:
keda1252-leuba tɛ'kɛʔ
AN
tetu1245-suai toko
p-mala1545-acd *tektek
tetu1245-vique toko
koto1251 ʔteke
TAP
dein1238 teih
sarr1247-adiab teh
lamm1241-westp tak:a
kula1280-lanto tə’ko
kama1365 takːeː
adan1251-otvai ’tɛko
buna1278-suai thɔkɔh
kuii1253 takók
buna1278-bobon thɔkɔh
sarr1247-nule taqo
baka1276 tekek
teiw1235 taqoq
pura1258 teke
abui1241-fuime tekok
blag1240-bama texe
maka1316 toke
sawi1256 ta'kɔ
blag1240-kulij tekeʔ
blag1240-warsa texe
kula1280-lanto tko
rett1240 tek
adan1251-lawah teko
fata1247 tɔkɛ
blag1240-tuntu texe
abui1241-takal te̞̞qɔq
kaer1234 tɛk
abui1241-takal te̞̞qɔɁ
rett1240 tɛk
kira1248 taˈkok
abui1241-ulaga taŋkok
atim1239 tekok
kabo1247 teko
blag1240-nule te'ke
kelo1247-hopte ta'kɛk
```

In [21]:

```
for c in attested_cognate_sets["thirteen"][0]:
    for group, lects in zip(("AL", "LH", "FL", "AN", "TAP"), groups):
        print(group)
        for concept, lect, form in cognates_by_class[c]:
            if lect in lects:
                print(lect, form)
```

```
AL
alor1247-pandai kə'rtou 'ilak 'talːɔ
alor1247-besar kartou 'ilaka 'talːɔ
alor1247-munas kər'tou 'ilak tə'lːɔ
LH
FL
AN
TAP
```

In [22]:

```
for c in attested_cognate_sets["tongue"][0]:
    for group, lects in zip(("AL", "LH", "FL", "AN", "TAP"), groups):
        print(group)
        for concept, lect, form in cognates_by_class[c]:
            if lect in lects:
                print(lect, form)
```

```
AL
alor1247-munas wɛwɛl
alor1247-pandai wɛwɛl
alor1247-baran fɛfɛl
alor1247-besar fɛ'fɛlɛŋ
LH
lama1277-belan ewel
lama1277-lewok ewel
lama1277-lerek eˈwel
lama1277-waiba wewel
lama1277-lewuk ewel
lama1277-lewog wewel
lama1277-botun wewe
lama1277-lewoi ˈweweˈləŋ
lama1277-lamal efel
lama1277-dulhi wewen
lama1277-paina eweləg
lama1277-kiwan weweyək
lama1277-baipi wewe
lama1277-lewom wewe
lama1277-lewop efel
lama1277-imulo efeləs
lama1277-lewot efel
lama1277-ritae wewel
lama1277-waiwa wewer
lama1277-pukau wewel
lama1277-lewob wewe
lama1277-horow wewer
lama1277-lamah ewel
lama1277-tanju wewel
lama1277-wuake ewel
lama1277-lamak ewel
lama1277-watan wewer
lama1277-mulan ewel
lama1277-merde ewelə
lama1277-adona ˈɜwɜˈrɜt
lama1277-kalik eˈvɛl
lama1277-ileap ewel
lama1277-kalik ewel
lama1277-bama wewel
lama1277-minga efel
FL
AN
TAP
dein1238 nawas:aŋ
sarr1247-adiab pe'fal
maka1316 ifi'leru
```

In [23]:

```
for c in attested_cognate_sets["church"][0]:
    for group, lects in zip(("AL", "LH", "FL", "AN", "TAP"), groups):
        print(group)
        for concept, lect, form in cognates_by_class[c]:
            if lect in lects:
                print(lect, form)
```

```
AL
alor1247-besar ga'rɛʤa
alor1247-munas gər'dʒa
alor1247-pandai uma gə'rɛdʒa
LH
lama1277-lewoi ɡeˈrɛʤa
lama1277-adona ɡɜˈrɛdʒa
FL
sika1262-hewa gereʤa
keda1252-leuba ɡə'rɛdʒa
AN
tetu1245-vique uma 'kreda
tetu1245-suai uma 'kreda
TAP
pura1258 ga'rija
rett1240 garaʤa
kelo1247-hopte gə'reɟ
teiw1235 gə'reja
adan1251-otvai ga’rɛɟa
```

In [24]:

```
for c in attested_cognate_sets["claw"][0]:
    for group, lects in zip(("AL", "LH", "FL", "AN", "TAP"), groups):
        print(group)
        for concept, lect, form in cognates_by_class[c]:
            if lect in lects:
                print(lect, form)
    print()

print()
print("For context, the forms in Lamaholot:")
for c in attested_cognate_sets["claw"][1]:
    for group, lects in zip(("AL", "LH", "FL", "AN", "TAP"), groups):
        print(group)
        for concept, lect, form in cognates_by_class[c]:
            if lect in lects:
                print(lect, form)
```

```
AL
alor1247-baran limɑŋ ta'nuŋgul
LH
FL
AN
TAP

AL
alor1247-munas kɛla 'leiŋ
LH
FL
AN
TAP

AL
alor1247-besar ga'raku
alor1247-pandai k'rako
LH
FL
AN
TAP
buna1278-bobon galakha
sawi1256 kə'riki


For context, the forms in Lamaholot:
AL
LH
lama1277-adona kɜˈmukure
FL
AN
p-mala1545-acd *kuhkuh
TAP
kelo1247-bring gɛkuh
AL
LH
lama1277-lewoi təˈnuʔi
FL
AN
TAP
AL
LH
lama1277-lerek kabəˈlən
FL
AN
TAP
```

In [25]:

```
for concept in concepts:
    by_group = attested_cognate_sets[concept]
    if by_group[0] != by_group[1]:
        if by_group[0] and by_group[1]:
            fl_skipping_l = (by_group[0] & by_group[2]) - by_group[1]
            an_skipping_l = (by_group[0] & by_group[3]) - by_group[1]
            skipping = fl_skipping_l | an_skipping_l
            a_but_not_l = by_group[0] - by_group[1]
            l_skipping_a = by_group[1] - by_group[0]

            print("Concept ‘{:}’ is different between Alorese and Lamaholot.".format(
                concept))
            print()
            if (a_but_not_l - skipping) - by_group[4]:
                print("", "Alorese innovated the following forms:")
                for cognateclass in a_but_not_l - skipping:
                    print("", cognates_by_class[cognateclass])
            if (a_but_not_l - skipping) & by_group[4]:
                print("", "Alorese shares the following forms with TAP languages:")
                for cognateclass in a_but_not_l - skipping:
                    print("", cognates_by_class[cognateclass])
            if skipping:
                print("", "Alorese shares the following forms with other AN languanges, but not Lamaholot:")
                for cognateclass in skipping:
                    print("", cognates_by_class[cognateclass])
            if l_skipping_a:
                print("", "One or more Lamaholot dialects innovated forms not found in Alorese:")
                for cognateclass in l_skipping_a:
                    print("", cognates_by_class[cognateclass])
            if by_group[1] & by_group[0]:
                print()
                print(" ", "Just for clarity:")
                print(" ", "There are also the following similarity classes shared between Alorese and LH.")
                for cognateclass in by_group[1] & by_group[0]:
                    print(" ", cognates_by_class[cognateclass])
        elif by_group[0]:
            print("Concept {:} is attested only in Alorese".format(concept))
        elif by_group[1]:
            print("Concept {:} is attested only in Lamaholot".format(concept))
        print("\n\n")
```

```
Concept roof_rafter is attested only in Lamaholot


Concept some is attested only in Lamaholot


Concept dew is attested only in Lamaholot


Concept fist is attested only in Lamaholot


Concept dibble_stick is attested only in Lamaholot


Concept ‘hand’ is different between Alorese and Lamaholot.

 One or more Lamaholot dialects innovated forms not found in Alorese:
 {('hand', 'lama1277-adona', 'ˈnaiʔjũ')}

  Just for clarity:
  There are also the following similarity classes shared between Alorese and LH.
  {('hand', 'kema1243', 'limɑr'), ('hand', 'lama1277-wuake', 'limak'), ('hand', 'alor1247-besar', 'limaŋ'), ('hand', 'lama1277-kalik', 'limaga'), ('hand', 'lama1277-mulan', 'lima'), ('hand', 'lama1277-bama', 'lima'), ('hand', 'sika1262-tanai', 'lima-ŋ'), ('hand', 'lama1277-baipi', 'lima'), ('hand', 'lama1277-merde', 'limak'), ('hand', 'lama1277-tanju', 'lima'), ('hand', 'p-cent2245-abvd', '*lima'), ('hand', 'lama1277-imulo', 'limasa'), ('hand', 'koto1251', 'ʔnima-f'), ('hand', 'lama1277-lewoe', 'lima'), ('hand', 'lama1277-minga', 'limasa'), ('hand', 'p-aust1307-abvd', '*(qa)lima'), ('hand', 'lama1277-belan', 'limak'), ('hand', 'p-mala1545-acd', '*qalima'), ('hand', 'tetu1246', "limɑn 'tanɛn"), ('hand', 'lama1277-dulhi', 'lima'), ('hand', 'lama1277-lerek', 'liˈma'), ('hand', 'lama1277-pukau', 'lima'), ('hand', 'alor1247-munas', 'limaŋ'), ('hand', 'idat1237', 'lima'), ('hand', 'lama1277-paina', 'limag'), ('hand', 'tetu1245-vique', 'liman'), ('hand', 'lama1277-botun', 'lima'), ('hand', 'lama1277-waiwa', 'limak'), ('hand', 'lama1277-lamah', 'limak'), ('hand', 'lama1277-lewot', 'limaha'), ('hand', 'lama1277-lewob', 'lima'), ('hand', 'alor1247-pandai', 'limaŋ'), ('hand', 'keda1252', 'liŋ'), ('hand', 'laka1255', 'limɑn'), ('hand', 'tuku1254', "limu 'tane"), ('hand', 'keda1252-leuwa', 'liŋ'), ('hand', 'lama1277-lewom', 'lima'), ('hand', 'lama1277-lewop', 'limaha'), ('hand', 'lama1277-ritae', 'lima'), ('hand', 'p-mala1545-acd', '*kamay'), ('hand', 'sika1262-maume', 'liman'), ('hand', 'tetu1245-suai', 'liman'), ('hand', 'p-mala1545-abvd', '*[qa]lima'), ('hand', 'lama1277-horow', 'limak'), ('hand', 'lama1277-lewog', 'lima'), ('hand', 'lama1277-ileap', 'limak'), ('hand', 'alor1247-baran', "limaŋ ka'lumak"), ('hand', 'lama1277-lamak', 'limak'), ('hand', 'lama1277-kiwan', 'limak'), ('hand', 'keda1252-leuba', 'liŋ'), ('hand', 'lama1277-waiba', 'lima'), ('hand', 'mamb1306', 'limɑ'), ('hand', 'lama1277-lewoi', 'ˈlimaŋ'), ('hand', 'lama1277-lamat', 'lima'), ('hand', 'lama1277-watan', 'limã'), ('hand', 'lama1277-lewuk', 'limaga'), ('hand', 'lama1277-lamal', 'limate'), ('hand', 'lama1277-kalik', 'liˈma'), ('hand', 'sika1262-hewa', 'lima'), ('hand', 'lama1277-lewok', 'limaga'), ('hand', 'alor1247-munas', 'limaʔ')}


Concept stem is attested only in Lamaholot


Concept ‘betel_vine’ is different between Alorese and Lamaholot.

 One or more Lamaholot dialects innovated forms not found in Alorese:
 {('betel_vine', 'lama1277-lewoi', 'ˈlɔlɔŋ')}

  Just for clarity:
  There are also the following similarity classes shared between Alorese and LH.
  {('betel_vine', 'laka1255', 'malus'), ('betel_vine', 'tetu1246', 'mɑlus'), ('betel_vine', 'maka1316', 'malu'), ('betel_vine', 'keda1252-leuba', 'mal'), ('betel_vine', 'lama1277-adona', 'ˈmaluʔ'), ('betel_vine', 'alor1247-munas', 'malu'), ('betel_vine', 'koto1251', 'manus'), ('betel_vine', 'lama1277-kalik', 'maˈlu'), ('betel_vine', 'alor1247-baran', 'malu'), ('betel_vine', 'buna1278-suai', 'molo'), ('betel_vine', 'alor1247-pandai', 'malu'), ('betel_vine', 'fata1247', 'malu'), ('betel_vine', 'mamb1306', 'ma:l'), ('betel_vine', 'buna1278-bobon', 'molo'), ('betel_vine', 'tuku1254', 'malu'), ('betel_vine', 'idat1237', "ma'lus"), ('betel_vine', 'lama1277-lerek', 'ˈmalor'), ('betel_vine', 'buna1278-malia', 'mɔlɔ'), ('betel_vine', 'lama1277-kalik', 'maˈlɔr'), ('betel_vine', 'buna1278-suai', 'moro'), ('betel_vine', 'alor1247-besar', 'malu')}


Concept ‘betel_nut_areca’ is different between Alorese and Lamaholot.

 Alorese innovated the following forms:
 {('betel_nut_areca', 'alor1247-baran', 'ʔufa')}
 Alorese shares the following forms with other AN languanges, but not Lamaholot:
 {('betel_nut_areca', 'alor1247-besar', 'ua'), ('betel_nut_areca', 'alor1247-munas', 'ua'), ('betel_nut_areca', 'alor1247-pandai', 'ua'), ('betel_nut_areca', 'keda1252-leuba', 'uwe')}
 One or more Lamaholot dialects innovated forms not found in Alorese:
 {('betel_nut_areca', 'lama1277-lerek', 'ˈkleruk'), ('betel_nut_areca', 'lama1277-kalik', 'kleˈruk')}
 {('betel_nut_areca', 'lama1277-adona', 'ˈwuaʔ'), ('betel_nut_areca', 'abui1241-ulaga', 'wuu'), ('betel_nut_areca', 'sika1262-hewa', 'ʋua'), ('betel_nut_areca', 'lama1277-lewoi', 'ˈwua')}


Concept other is attested only in Lamaholot


Concept ‘ten’ is different between Alorese and Lamaholot.

 Alorese shares the following forms with TAP languages:
 {('ten', 'hama1240', 'airnu'), ('ten', 'blag1240-warsa', 'xar nuk'), ('ten', 'p-alor1249', '*qar-'), ('ten', 'rett1240', 'karanu'), ('ten', 'adan1251-lawah', 'airnu'), ('ten', 'abui1241-takal', "kär.'nu.ku"), ('ten', 'sarr1247-nule', 'qarnuk'), ('ten', 'nede1245', 'ka nuk'), ('ten', 'alor1247-besar', 'kartou'), ('ten', 'abui1241-ulaga', 'karnuku'), ('ten', 'adan1251-otvai', '’ʔernu'), ('ten', 'dein1238', 'qarnuk'), ('ten', 'alor1247-baran', 'kartɔ'), ('ten', 'p-alor1249', '*qar nuk'), ('ten', 'atim1239', 'kar nuku'), ('ten', 'kelo1247-hopte', "kar'nuk"), ('ten', 'kaer1234', "xar nu'ko"), ('ten', 'lamm1241-westp', 'keanuku'), ('ten', 'kira1248', 'karnuku'), ('ten', 'kafo1240', 'kɑrnuku'), ('ten', 'abui1241-petle', "kar.'nu.ku"), ('ten', 'baka1276', 'arnu'), ('ten', 'blag1240-tuntu', 'qarnuk'), ('ten', 'p-timo1261', '*qar-'), ('ten', 'teiw1235', 'qa:r nuk'), ('ten', 'kabo1247', 'karnu'), ('ten', 'sarr1247-adiab', 'qarnuk'), ('ten', 'pura1258', "a'rinu"), ('ten', 'kelo1247-bring', 'kɑrənʊk'), ('ten', 'blag1240-nule', 'arnu'), ('ten', 'kama1365', 'kɑrnɔk'), ('ten', 'blag1240-bama', 'qarnuku'), ('ten', 'alor1247-munas', "kər'tou"), ('ten', 'abui1241-fuime', 'kar nuku'), ('ten', 'blag1240-kulij', 'ar nu'), ('ten', 'kuii1253', 'karnuku'), ('ten', 'alor1247-pandai', "kə'rtou")}
 One or more Lamaholot dialects innovated forms not found in Alorese:
 {('ten', 'buna1278-bobon', 'sogo'), ('ten', 'lama1277-lerek', 'heˈpulo'), ('ten', 'buna1278-malia', 'sɔgɔ'), ('ten', 'lama1277-adona', 'puloː'), ('ten', 'sika1262-hewa', 'pulu'), ('ten', 'laka1255', "sa'kulu"), ('ten', 'tetu1245-suai', 'sanulu'), ('ten', 'p-mala1545-acd', '*sa-ŋa-puluq'), ('ten', 'lama1277-lewoi', 'ˈpulɔ'), ('ten', 'lama1277-kalik', 'ˈspulo'), ('ten', 'keda1252-leuba', 'pulu'), ('ten', 'mamb1306', "sa'gul"), ('ten', 'tuku1254', "sɑ'gulu"), ('ten', 'kema1243', ",sa'pulu"), ('ten', 'tetu1245-vique', 'sanulu'), ('ten', 'buna1278-suai', 'sogo'), ('ten', 'tetu1246', "sa'nulu")}


Concept ‘corn_maize’ is different between Alorese and Lamaholot.

 One or more Lamaholot dialects innovated forms not found in Alorese:
 {('corn_maize', 'lama1277-kalik', 'ˈkvaru'), ('corn_maize', 'lama1277-lerek', 'ˈkəvaror'), ('corn_maize', 'lama1277-kalik', 'kvaˈrɔr')}

  Just for clarity:
  There are also the following similarity classes shared between Alorese and LH.
  {('corn_maize', 'kaer1234', "ba'tar"), ('corn_maize', 'blag1240-nule', 'batar'), ('corn_maize', 'adan1251-otvai', '’bate'), ('corn_maize', 'abui1241-fuime', 'fa:ti'), ('corn_maize', 'pura1258', 'batar'), ('cooked_rice', 'alor1247-munas', 'wata'), ('corn_maize', 'kabo1247', 'botiʔ'), ('cooked_corn', 'sika1262-hewa', 'ʋatɑr bəŋɛŋ'), ('cooked_rice', 'alor1247-munas', "wata 'tahak"), ('corn_maize', 'blag1240-tuntu', "ba'tar"), ('corn_maize', 'blag1240-bama', 'batar'), ('corn_maize', 'wers1238-taram', "pe'ter"), ('corn_maize', 'idat1237', "pɑ'ta:r"), ('corn_maize', 'alor1247-munas', 'wata'), ('corn_maize', 'sarr1247-nule', "ba'tar"), ('corn_maize', 'keda1252-leuba', 'wɑtɑr'), ('cooked_rice', 'alor1247-baran', 'fata'), ('corn_maize', 'nede1245', 'ba:ta'), ('corn_maize', 'baka1276', 'batar'), ('corn_maize', 'mamb1306', "bɑ'tar"), ('cooked_rice', 'alor1247-pandai', "wata 'tahak"), ('corn_maize', 'alor1247-pandai', 'wata'), ('corn_maize', 'sawi1256', 'pata'), ('corn_maize', 'kama1365', 'patei'), ('corn_maize', 'kuii1253', 'batar'), ('corn_maize', 'tetu1245-suai', 'batar'), ('corn_maize', 'kelo1247-hopte', 'bat'), ('corn_maize', 'dein1238', 'batr'), ('corn_maize', 'wers1238-marit', "pɛ'tɛr"), ('corn_maize', 'lamm1241-westp', 'bat:e'), ('corn_maize', 'rett1240', 'batal'), ('corn_maize', 'abui1241-takal', 'fä:t'), ('cooked_rice', 'alor1247-besar', 'fata'), ('corn_maize', 'laka1255', "bɑ'tar"), ('corn_maize', 'alor1247-besar', 'fata'), ('corn_maize', 'kira1248', 'bati'), ('corn_maize', 'adan1251-lawah', 'batiʔ'), ('cooked_corn', 'alor1247-munas', "wata 'punaʔ"), ('cooked_corn', 'alor1247-besar', "fata pu'nakaŋ"), ('corn_maize', 'sarr1247-adiab', "bə'tar"), ('corn_maize', 'tetu1245-vique', 'batar'), ('corn_maize', 'blag1240-kulij', 'batar'), ('corn_maize', 'kula1280-lanto', 'pte'), ('corn_maize', 'lama1277-lewoi', 'ˈwata'), ('corn_maize', 'atim1239', 'fati'), ('cooked_corn', 'alor1247-pandai', "wata 'puna"), ('corn_maize', 'tetu1246', 'bɑtɑr'), ('corn_maize', 'teiw1235', "ba'tar"), ('corn_maize', 'sika1262-hewa', 'ʋatar'), ('cooked_corn', 'lama1277-adona', 'wataʔ ˈmalune'), ('corn_maize', 'abui1241-ulaga', 'fa:t'), ('corn_maize', 'kula1280-lanto', 'pə’te'), ('corn_maize', 'lama1277-adona', 'ˈwataʔ'), ('corn_maize', 'blag1240-warsa', 'batar')}


Concept ‘chair’ is different between Alorese and Lamaholot.

 One or more Lamaholot dialects innovated forms not found in Alorese:
 {('chair', 'lama1277-adona', 'nɔbɔ')}
 {('chair', 'abui1241-ulaga', 'kursi'), ('chair', 'keda1252-leuba', 'kursi'), ('chair', 'lama1277-adona', 'kursi'), ('chair', 'lama1277-lewoi', 'ˈkursi'), ('chair', 'sika1262-hewa', 'kursi')}

  Just for clarity:
  There are also the following similarity classes shared between Alorese and LH.
  {('chair', 'wers1238-marit', 'kander'), ('chair', 'adan1251-otvai', 'ka’dere'), ('chair', 'teiw1235', "kade'ra"), ('chair', 'kelo1247-hopte', "kə'dɛ:r"), ('chair', 'tetu1245-suai', "ka'deira"), ('chair', 'rett1240', 'kadɛra'), ('chair', 'lamm1241-westp', 'kadera'), ('chair', 'sawi1256', 'kander'), ('chair', 'kula1280-lanto', 'kan’dera'), ('chair', 'fata1247', "ka'dɛra"), ('chair', 'pura1258', "ka'dera"), ('chair', 'lama1277-lerek', 'kəˈdera'), ('chair', 'tetu1245-vique', "ka'deira"), ('chair', 'alor1247-besar', "ka'dera"), ('chair', 'alor1247-pandai', "k'dera"), ('chair', 'kaer1234', "kede'ra"), ('chair', 'maka1316', "ka'dera"), ('chair', 'alor1247-munas', "kan'dɛra"), ('chair', 'kama1365', 'kadera')}


Concept ‘to_scratch’ is different between Alorese and Lamaholot.

 Alorese shares the following forms with other AN languanges, but not Lamaholot:
 {('to_scratch', 'mamb1306', 'koi'), ('to_scratch', 'alor1247-besar', 'gou'), ('to_scratch', 'kema1243', 'koʔi'), ('to_scratch', 'buna1278-bobon', 'kɔɁi'), ('to_scratch', 'tetu1245-vique', 'kɔɁi'), ('to_scratch', 'buna1278-malia', 'khɔʔi'), ('to_scratch', 'tetu1245-suai', 'kɔɁi'), ('to_scratch', 'tetu1246', 'koi'), ('to_scratch', 'buna1278-suai', 'kɔɁi')}
 One or more Lamaholot dialects innovated forms not found in Alorese:
 {('to_scratch', 'lama1277-lewoi', 'ˈraɡuʔ'), ('to_scratch', 'lama1277-adona', 'ˈraɡuʔ'), ('to_scratch', 'lama1277-dulhi', 'ragu'), ('to_scratch', 'lama1277-lewoe', 'ragu'), ('to_scratch', 'lama1277-tanju', 'ragu'), ('to_scratch', 'lama1277-kiwan', 'ragu'), ('to_scratch', 'lama1277-lewok', 'kəragum'), ('to_scratch', 'lama1277-botun', 'ragu'), ('to_scratch', 'lama1277-baipi', 'ragu'), ('to_scratch', 'lama1277-lewuk', 'ragu'), ('to_scratch', 'lama1277-bama', 'ragu'), ('to_scratch', 'lama1277-merde', 'ragu'), ('to_scratch', 'lama1277-kalik', 'kəˈraɡu'), ('to_scratch', 'lama1277-horow', 'ragu'), ('to_scratch', 'lama1277-lewop', 'ragu'), ('to_scratch', 'lama1277-lamal', 'rago'), ('to_scratch', 'lama1277-lerek', 'kəˈraɡum'), ('to_scratch', 'lama1277-imulo', 'rag'), ('to_scratch', 'lama1277-lewob', 'ragu'), ('to_scratch', 'lama1277-lewom', 'ragu'), ('to_scratch', 'lama1277-ritae', 'ragu'), ('to_scratch', 'kama1365', 'fakiː'), ('to_scratch', 'lama1277-kalik', 'kəragu'), ('to_scratch', 'lama1277-lewot', 'ragu'), ('to_scratch', 'lama1277-minga', 'ragu'), ('to_scratch', 'lama1277-lewog', 'ragu'), ('to_scratch', 'lama1277-belan', 'ragu'), ('to_scratch', 'lama1277-lamak', 'ragu'), ('to_scratch', 'lama1277-mulan', 'ragu'), ('to_scratch', 'lama1277-wuake', 'ragu'), ('to_scratch', 'lama1277-lamah', 'ragu'), ('to_scratch', 'lama1277-lamat', 'ragu'), ('to_scratch', 'lama1277-waiba', 'ragu')}

  Just for clarity:
  There are also the following similarity classes shared between Alorese and LH.
  {('to_scratch', 'alor1247-pandai', 'gau'), ('to_scratch', 'tuku1254', 'krui'), ('to_scratch', 'p-mala1545-abvd', '*kaɾaw'), ('to_scratch', 'sika1262-hewa', 'ʔaro'), ('to_scratch', 'p-aust1307-abvd', '*kaʀaw'), ('to_scratch', 'pura1258', "ka'rabi"), ('to_scratch', 'sika1262-hewa', 'garu'), ('to_scratch', 'fata1247', 'kaurɛ'), ('to_scratch', 'lamm1241-westp', 'karasi'), ('to_scratch', 'p-timo1261', '*karab'), ('to_scratch', 'lama1277-ileap', 'gau'), ('to_scratch', 'dein1238', 'krab'), ('to_scratch', 'p-mala1545-acd', '*garus'), ('to_scratch', 'sarr1247-nule', 'kəra:b'), ('to_scratch', 'koto1251', 'n-kai'), ('to_scratch', 'maka1316', "ka'uru"), ('to_scratch', 'sika1262-maume', 'ʔaro'), ('to_scratch', 'lama1277-watan', 'garu'), ('to_scratch', 'p-cent2245-abvd', '*kaʀaw'), ('to_scratch', 'keda1252-leuba', 'karɔ'), ('to_scratch', 'sika1262-tanai', 'kəru'), ('to_scratch', 'idat1237', "karu'i"), ('to_scratch', 'kaer1234', 'krabis'), ('to_scratch', 'laka1255', 'krui'), ('to_scratch', 'lama1277-waiwa', 'garuk'), ('to_scratch', 'alor1247-munas', 'gau'), ('to_scratch', 'p-mala1545-acd', '*garut'), ('to_scratch', 'p-mala1545-acd', '*g<um>arut'), ('to_scratch', 'keda1252', 'karo'), ('to_scratch', 'lama1277-pukau', 'gaʔu'), ('to_scratch', 'p-alor1249', '*karab'), ('to_scratch', 'sarr1247-adiab', 'kəra:b'), ('to_scratch', 'keda1252-leuwa', 'karɔ'), ('to_scratch', 'lama1277-paina', 'kərag'), ('to_scratch', 'alor1247-baran', 'gaɔ')}


Concept ‘thirteen’ is different between Alorese and Lamaholot.

 Alorese innovated the following forms:
 {('thirteen', 'alor1247-pandai', "kə'rtou 'ilak 'talːɔ"), ('thirteen', 'alor1247-besar', "kartou 'ilaka 'talːɔ"), ('thirteen', 'alor1247-munas', "kər'tou 'ilak tə'lːɔ")}
 One or more Lamaholot dialects innovated forms not found in Alorese:
 {('thirteen', 'tetu1245-suai', 'sanulu resin tolu'), ('thirteen', 'sika1262-hewa', 'pulu ʋot təlu'), ('thirteen', 'lama1277-kalik', 'ˈspulo no təˈlu'), ('thirteen', 'koto1251', 'boʔ es am tenu'), ('thirteen', 'lama1277-lerek', 'heˈpulo no təl'), ('thirteen', 'lama1277-adona', 'pulok tɜlo'), ('thirteen', 'lama1277-lewoi', 'pulo noʔoŋ ˈtɛlːo'), ('thirteen', 'tetu1245-vique', 'sanulu resin tolu'), ('thirteen', 'keda1252-leuba', "pulaʔ 'tɛlu")}


Concept to_return is attested only in Lamaholot


Concept footprint is attested only in Lamaholot


Concept ‘jackfruit’ is different between Alorese and Lamaholot.

 Alorese shares the following forms with other AN languanges, but not Lamaholot:
 {('jackfruit', 'wers1238-marit', 'naŋ'), ('jackfruit', 'rett1240', 'naŋka'), ('jackfruit', 'kaer1234', "na'ka"), ('jackfruit', 'blag1240-warsa', 'naka'), ('jackfruit', 'kuii1253', 'naŋka'), ('jackfruit', 'alor1247-pandai', 'naka'), ('jackfruit', 'baka1276', 'naŋka'), ('jackfruit', 'blag1240-nule', 'naŋka'), ('jackfruit', 'blag1240-kulij', 'naŋka'), ('jackfruit', 'blag1240-tuntu', 'naka'), ('jackfruit', 'alor1247-munas', 'nakaŋ'), ('jackfruit', 'teiw1235', 'nak'), ('jackfruit', 'sika1262-hewa', 'nakat'), ('jackfruit', 'alor1247-besar', 'naŋka')}
 One or more Lamaholot dialects innovated forms not found in Alorese:
 {('jackfruit', 'lama1277-kalik', 'kveˈrak'), ('jackfruit', 'keda1252-leuba', "wɛ'raʔ"), ('jackfruit', 'lama1277-lerek', 'kəˈwerak'), ('jackfruit', 'lama1277-lewoi', 'kəˈwɛra'), ('jackfruit', 'lama1277-adona', 'ˈkwɛrak')}


Concept ‘mosque’ is different between Alorese and Lamaholot.

 One or more Lamaholot dialects innovated forms not found in Alorese:
 {('mosque', 'sika1262-hewa', 'mesʤid'), ('mosque', 'buna1278-suai', 'mɛdZit'), ('mosque', 'buna1278-bobon', 'mɛdZit'), ('mosque', 'lama1277-lewoi', 'mesˈʤit')}

  Just for clarity:
  There are also the following similarity classes shared between Alorese and LH.
  {('mosque', 'adan1251-otvai', 'ʔuma ’sigi'), ('mosque', 'abui1241-ulaga', 'mesɟid'), ('mosque', 'kaer1234', 'misikit'), ('mosque', 'alor1247-besar', "uma 'sigi"), ('mosque', 'teiw1235', 'mesɟid'), ('mosque', 'keda1252-leuba', "ma'siŋki"), ('mosque', 'rett1240', 'umasigi'), ('mosque', 'maka1316', 'mezid'), ('mosque', 'kelo1247-hopte', "mɛs'ɟid"), ('mosque', 'lama1277-adona', 'mɛˈsjit'), ('mosque', 'pura1258', "ma'sigit"), ('mosque', 'pura1258', "uma 'sigit"), ('mosque', 'alor1247-munas', "uma 'sigi"), ('mosque', 'alor1247-pandai', "uma 'sigi"), ('mosque', 'sawi1256', 'mɛsɟid')}


Concept ‘money’ is different between Alorese and Lamaholot.

 Alorese innovated the following forms:
 {('money', 'alor1247-munas', "k'uar")}
 {('money', 'alor1247-munas', 'bintis')}
 Alorese shares the following forms with other AN languanges, but not Lamaholot:
 {('money', 'alor1247-besar', 'sɛŋ'), ('money', 'kula1280-lanto', '’sena'), ('money', 'pura1258', 'sɛŋ'), ('money', 'rett1240', 'sɛŋ'), ('money', 'alor1247-pandai', 'sɛŋ'), ('money', 'wers1238-marit', 'sɛŋ'), ('money', 'kama1365', 'seŋ'), ('money', 'kaer1234', 'seŋ'), ('money', 'adan1251-otvai', '’sɛŋ'), ('money', 'sawi1256', 'sɛna'), ('money', 'rett1240', 'hoaŋ'), ('money', 'tetu1245-vique', 'ɔsaŋ'), ('money', 'tetu1245-suai', 'osan'), ('money', 'abui1241-ulaga', 'seŋ'), ('money', 'teiw1235', 'sen'), ('money', 'abui1241-takal', 'se̞äŋ')}
 One or more Lamaholot dialects innovated forms not found in Alorese:
 {('money', 'sika1262-hewa', 'do:i'), ('money', 'lama1277-adona', 'ˈdoi'), ('money', 'kaer1234', 'doi'), ('money', 'lamm1241-westp', 'doi'), ('money', 'lama1277-lerek', 'ˈdoit'), ('money', 'lama1277-kalik', 'doi'), ('money', 'lama1277-lewoi', 'ˈdoi'), ('money', 'lama1277-kalik', 'doer'), ('money', 'kelo1247-hopte', 'du:i'), ('money', 'keda1252-leuba', 'duiʔ')}


Concept ‘thread’ is different between Alorese and Lamaholot.

 Alorese shares the following forms with other AN languanges, but not Lamaholot:
 {('thread', 'keda1252-leuba', "bə'naŋ"), ('thread', 'alor1247-besar', "ba'nːaŋ"), ('thread', 'sika1262-hewa', 'bənaŋ')}
 One or more Lamaholot dialects innovated forms not found in Alorese:
 {('thread', 'lama1277-lewoi', 'ˈmale')}
 {('thread', 'lama1277-adona', 'bɜˈʔuwa')}

  Just for clarity:
  There are also the following similarity classes shared between Alorese and LH.
  {('thread', 'koto1251', 'abas'), ('thread', 'pura1258', 'api'), ('thread', 'lama1277-lerek', 'ˈkapha'), ('thread', 'tetu1245-suai', 'kabas'), ('thread', 'adan1251-otvai', '’ʔab'), ('thread', 'kelo1247-hopte', 'kap'), ('thread', 'alor1247-pandai', 'kapo'), ('thread', 'lamm1241-westp', 'kap:as'), ('thread', 'kaer1234', "qa'pis"), ('thread', 'lama1277-kalik', 'kaˈpək'), ('thread', 'rett1240', 'kapa'), ('thread', 'alor1247-munas', 'kapɔ'), ('thread', 'tetu1245-vique', 'kabas'), ('thread', 'abui1241-takal', "kä.'päi"), ('thread', 'teiw1235', "qa'pas")}


Concept ‘two’ is different between Alorese and Lamaholot.

 One or more Lamaholot dialects innovated forms not found in Alorese:
 {('two', 'p-aust1307-abvd', '*duSa'), ('two', 'p-mala1545-abvd', '*duha'), ('two', 'p-cent2245-abvd', '*dua'), ('two', 'lama1277-lerek', 'ˈdʒ͡ua'), ('two', 'lama1277-kalik', 'ˈʤua'), ('two', 'p-mala1545-acd', '*duha')}

  Just for clarity:
  There are also the following similarity classes shared between Alorese and LH.
  {('two', 'tetu1245-vique', 'rua'), ('two', 'baka1276', 'aru'), ('two', 'alor1247-munas', 'rua'), ('two', 'kama1365', 'aruk'), ('two', 'teiw1235', 'raq'), ('two', 'tetu1245-suai', 'rua'), ('two', 'lama1277-lewoi', 'ˈrua'), ('two', 'kelo1247-bring', 'ərʊk'), ('two', 'kelo1247-hopte', "ʔə'ruk"), ('two', 'dein1238', 'raq'), ('two', 'alor1247-besar', 'rua'), ('two', 'alor1247-pandai', 'rua'), ('two', 'alor1247-baran', 'rua'), ('two', 'tetu1246', 'rua'), ('two', 'mamb1306', 'rua'), ('two', 'lama1277-adona', 'ˈrua'), ('two', 'laka1255', 'rua'), ('two', 'tuku1254', 'ru:'), ('two', 'pura1258', 'aru'), ('two', 'sika1262-hewa', 'rua'), ('two', 'blag1240-nule', 'aru'), ('two', 'sika1262-tanai', 'rua'), ('two', 'sarr1247-adiab', 'raq'), ('two', 'kema1243', "hu'rua"), ('two', 'sarr1247-nule', 'raq')}


Concept ‘tongue’ is different between Alorese and Lamaholot.

 One or more Lamaholot dialects innovated forms not found in Alorese:
 {('tongue', 'keda1252-leuwa', 'æbæl'), ('tongue', 'lama1277-lamat', 'eblə'), ('tongue', 'fata1247', 'ipil'), ('tongue', 'lama1277-lewoe', 'ebe'), ('tongue', 'keda1252-leuba', 'ebel'), ('tongue', 'keda1252', 'ebel')}

  Just for clarity:
  There are also the following similarity classes shared between Alorese and LH.
  {('tongue', 'lama1277-belan', 'ewel'), ('tongue', 'lama1277-lewok', 'ewel'), ('tongue', 'alor1247-munas', 'wɛwɛl'), ('tongue', 'lama1277-lerek', 'eˈwel'), ('tongue', 'lama1277-waiba', 'wewel'), ('tongue', 'lama1277-lewuk', 'ewel'), ('tongue', 'alor1247-pandai', 'wɛwɛl'), ('tongue', 'lama1277-lewog', 'wewel'), ('tongue', 'lama1277-botun', 'wewe'), ('tongue', 'lama1277-lewoi', 'ˈweweˈləŋ'), ('tongue', 'lama1277-lamal', 'efel'), ('tongue', 'lama1277-dulhi', 'wewen'), ('tongue', 'lama1277-paina', 'eweləg'), ('tongue', 'lama1277-kiwan', 'weweyək'), ('tongue', 'lama1277-baipi', 'wewe'), ('tongue', 'lama1277-lewom', 'wewe'), ('tongue', 'lama1277-lewop', 'efel'), ('tongue', 'lama1277-imulo', 'efeləs'), ('tongue', 'lama1277-lewot', 'efel'), ('tongue', 'lama1277-ritae', 'wewel'), ('tongue', 'alor1247-baran', 'fɛfɛl'), ('tongue', 'lama1277-waiwa', 'wewer'), ('tongue', 'dein1238', 'nawas:aŋ'), ('tongue', 'lama1277-pukau', 'wewel'), ('tongue', 'lama1277-lewob', 'wewe'), ('tongue', 'lama1277-horow', 'wewer'), ('tongue', 'lama1277-lamah', 'ewel'), ('tongue', 'lama1277-tanju', 'wewel'), ('tongue', 'lama1277-wuake', 'ewel'), ('tongue', 'lama1277-lamak', 'ewel'), ('tongue', 'lama1277-watan', 'wewer'), ('tongue', 'lama1277-mulan', 'ewel'), ('tongue', 'lama1277-merde', 'ewelə'), ('tongue', 'alor1247-besar', "fɛ'fɛlɛŋ"), ('tongue', 'lama1277-adona', 'ˈɜwɜˈrɜt'), ('tongue', 'sarr1247-adiab', "pe'fal"), ('tongue', 'lama1277-kalik', 'eˈvɛl'), ('tongue', 'lama1277-ileap', 'ewel'), ('tongue', 'lama1277-kalik', 'ewel'), ('tongue', 'lama1277-bama', 'wewel'), ('tongue', 'lama1277-minga', 'efel'), ('tongue', 'maka1316', "ifi'leru")}


Concept ‘fifteen’ is different between Alorese and Lamaholot.

 Alorese innovated the following forms:
 {('fifteen', 'alor1247-pandai', "kə'rtou 'ilak 'lɛma"), ('fifteen', 'alor1247-besar', "kartou 'ilaka 'lɛma"), ('fifteen', 'alor1247-munas', "kər'tou 'ilak 'lɛma")}
 One or more Lamaholot dialects innovated forms not found in Alorese:
 {('fifteen', 'lama1277-adona', 'pulok lɛma'), ('fifteen', 'lama1277-lewoi', 'pulo noʔoŋ ˈlɛma'), ('fifteen', 'lama1277-kalik', 'ˈspulo no lɛm'), ('fifteen', 'lama1277-lerek', 'heˈpulo no ˈlɛma'), ('fifteen', 'sika1262-hewa', 'pulu ʋot lima'), ('fifteen', 'keda1252-leuba', "pulaʔ 'lɛmɛ")}


Concept ‘blood’ is different between Alorese and Lamaholot.

 Alorese shares the following forms with other AN languanges, but not Lamaholot:
 {('blood', 'alor1247-munas', 'ra'), ('blood', 'tetu1246', 'ra:n'), ('blood', 'tetu1245-vique', 'ra:'), ('blood', 'alor1247-baran', 'ra:'), ('blood', 'laka1255', 'ra:n'), ('blood', 'idat1237', 'ra:n'), ('blood', 'alor1247-pandai', 'ra'), ('blood', 'alor1247-munas', 'raŋ'), ('blood', 'alor1247-besar', 'ra'), ('blood', 'tetu1245-suai', 'ra:'), ('blood', 'kema1243', 'ra:r'), ('blood', 'tuku1254', 'ra:')}
 One or more Lamaholot dialects innovated forms not found in Alorese:
 {('blood', 'lama1277-dulhi', 'mei'), ('blood', 'lama1277-adona', 'mei'), ('blood', 'lama1277-lewuk', 'smei'), ('blood', 'sika1262-maume', 'mei'), ('blood', 'lama1277-lerek', 'həˈmei'), ('blood', 'lama1277-lewom', 'mei'), ('blood', 'lama1277-ritae', 'mei'), ('blood', 'lama1277-waiwa', 'mei'), ('blood', 'lama1277-watan', 'mei'), ('blood', 'lama1277-baipi', 'mei'), ('blood', 'lama1277-minga', 'səmei'), ('blood', 'lama1277-imulo', 'səmeisa'), ('blood', 'lama1277-botun', 'mei'), ('blood', 'lama1277-horow', 'mei'), ('blood', 'lama1277-merde', 'mei'), ('blood', 'lama1277-bama', 'mei'), ('blood', 'lama1277-mulan', 'mehi'), ('blood', 'lama1277-lewop', 'səmei'), ('blood', 'sika1262-hewa', 'mei'), ('blood', 'lama1277-kalik', 'ˈsmein'), ('blood', 'lama1277-lamal', 'mei'), ('blood', 'lama1277-kiwan', 'mei'), ('blood', 'lama1277-lewog', 'meiʔ'), ('blood', 'lama1277-tanju', 'mei'), ('blood', 'lama1277-lewoi', 'ˈmei'), ('blood', 'lama1277-waiba', 'mei'), ('blood', 'lama1277-lewoe', 'meʔi'), ('blood', 'lama1277-lamat', 'meʔi'), ('blood', 'lama1277-wuake', 'səmei'), ('blood', 'lama1277-paina', 'həmein'), ('blood', 'lama1277-lewob', 'mei'), ('blood', 'lama1277-lewok', 'səmei'), ('blood', 'lama1277-ileap', 'mei'), ('blood', 'sika1262-tanai', 'mei̥-ŋ'), ('blood', 'lama1277-belan', 'mei'), ('blood', 'lama1277-lamah', 'mei'), ('blood', 'lama1277-lamak', 'mei'), ('blood', 'lama1277-lewot', 'həmeiha'), ('blood', 'sika1262-tanai', 'mei'), ('blood', 'lama1277-pukau', 'mei'), ('blood', 'lama1277-kalik', 'səmei')}


Concept ‘sea’ is different between Alorese and Lamaholot.

 One or more Lamaholot dialects innovated forms not found in Alorese:
 {('sea', 'lama1277-lamal', 'lefa'), ('sea', 'lama1277-adona', 'lewaʔ'), ('sea', 'lama1277-lewop', 'lefar'), ('sea', 'lama1277-lewot', 'lefa'), ('sea', 'lama1277-imulo', 'lefar'), ('sea', 'lama1277-minga', 'lewa'), ('sea', 'keda1252-leuwa', 'lεva'), ('sea', 'lama1277-mulan', 'lewa')}
 {('sea', 'lama1277-belan', 'ojok'), ('sea', 'lama1277-lewok', 'loyoru'), ('sea', 'lama1277-lerek', 'ˈlojor'), ('sea', 'lama1277-kalik', 'loˈʤɔr'), ('sea', 'lama1277-paina', 'loyor'), ('sea', 'lama1277-lewuk', 'lojor'), ('sea', 'lama1277-wuake', 'ojok'), ('sea', 'lama1277-kalik', 'lojor')}

  Just for clarity:
  There are also the following similarity classes shared between Alorese and LH.
  {('sea', 'keda1252', 'tahi'), ('sea', 'lama1277-lamat', 'tahi'), ('sea', 'sika1262-maume', 't̪ahi'), ('sea', 'lama1277-horow', 'tahik'), ('sea', 'alor1247-munas', 'tahiʔ'), ('sea', 'lama1277-lamah', 'tahi'), ('sea', 'keda1252-leuwa', 'tahiʔ a̘ŋin'), ('sea', 'p-mala1545-abvd', '*tasik'), ('sea', 'lama1277-lewom', 'tahik'), ('sea', 'sika1262-tanai', 'tahi'), ('sea', 'idat1237', 'tɑsi'), ('sea', 'lama1277-ritae', 'tahik'), ('sea', 'lama1277-lewoi', 'tahi'), ('sea', 'lama1277-lewob', 'tahi'), ('sea', 'p-cent2245-abvd', '*tasik'), ('sea', 'sika1262-hewa', 'tahi'), ('sea', 'lama1277-tanju', 'tahik'), ('sea', 'lama1277-pukau', 'tahik'), ('sea', 'lama1277-dulhi', 'tahik'), ('sea', 'keda1252-leuba', "ta'hiʔ"), ('sea', 'tetu1245-vique', 'tasi'), ('sea', 'alor1247-besar', 'tahi'), ('sea', 'alor1247-pandai', 'tahi'), ('sea', 'lama1277-merde', 'tahi'), ('sea', 'lama1277-ileap', 'tahi'), ('sea', 'lama1277-watan', 'tahik'), ('sea', 'p-mala1545-acd', '*tasik'), ('sea', 'lama1277-waiwa', 'tahik'), ('sea', 'lama1277-lamak', 'tahik'), ('sea', 'laka1255', 'tɑsi'), ('sea', 'koto1251', 'tasi'), ('sea', 'tetu1245-suai', 'tasi'), ('sea', 'fata1247', 'tahi'), ('sea', 'lama1277-bama', 'tahik'), ('sea', 'tuku1254', 'tɑsi'), ('sea', 'lama1277-kiwan', 'tahi'), ('sea', 'lama1277-lewog', 'tahik'), ('sea', 'lama1277-baipi', 'tahik'), ('sea', 'fata1247', 'tahi ira'), ('sea', 'teiw1235', 'taɁ'), ('sea', 'mamb1306', 'tas'), ('sea', 'lama1277-botun', 'tahik'), ('sea', 'kema1243', 'tasi'), ('sea', 'lama1277-lewoe', 'tahi'), ('sea', 'tetu1246', "tɑ'si"), ('sea', 'sika1262-hewa', 'tahiʔ'), ('sea', 'lama1277-waiba', 'tahik')}


Concept ‘wrong’ is different between Alorese and Lamaholot.

 One or more Lamaholot dialects innovated forms not found in Alorese:
 {('wrong', 'lama1277-lerek', 'bənələˈŋən')}

  Just for clarity:
  There are also the following similarity classes shared between Alorese and LH.
  {('wrong', 'p-mala1545-acd', '*salaq'), ('wrong', 'baka1276', 'hala'), ('wrong', 'fata1247', 'sala'), ('wrong', 'maka1316', 'sala'), ('wrong', 'buna1278-bobon', 'sal'), ('wrong', 'alor1247-pandai', 'halaŋ'), ('wrong', 'atim1239', 'hasala'), ('wrong', 'sika1262-hewa', 'hala'), ('wrong', 'tetu1245-vique', 'sala'), ('wrong', 'keda1252-leuba', 'kehe'), ('wrong', 'tetu1245-suai', 'sala'), ('wrong', 'lama1277-kalik', 'snaˈlak'), ('wrong', 'blag1240-bama', 'gasaliŋ'), ('wrong', 'abui1241-fuime', 'hasala'), ('wrong', 'alor1247-besar', 'hala'), ('wrong', 'lama1277-lewoi', 'ˈnalaŋ'), ('wrong', 'pura1258', "a'hala"), ('wrong', 'kelo1247-hopte', "gə'ha:l"), ('wrong', 'lama1277-adona', 'ˈnalã'), ('wrong', 'koto1251', 'n-sana'), ('wrong', 'adan1251-lawah', 'ʔahalaʔ'), ('wrong', 'blag1240-nule', 'hala'), ('wrong', 'adan1251-otvai', 'a’hal'), ('wrong', 'blag1240-warsa', 'gasaliŋ'), ('wrong', 'kabo1247', 'asala'), ('wrong', 'rett1240', 'gahala'), ('wrong', 'buna1278-suai', 'sal'), ('wrong', 'alor1247-munas', 'halaŋ'), ('wrong', 'blag1240-kulij', 'hala'), ('wrong', 'alor1247-munas', 'hala'), ('wrong', 'blag1240-tuntu', 'gasali'), ('wrong', 'teiw1235', "gə'saj")}


Concept ‘seventeen’ is different between Alorese and Lamaholot.

 Alorese innovated the following forms:
 {('seventeen', 'alor1247-pandai', "kə'rtou 'ilak 'pito"), ('seventeen', 'alor1247-besar', "kartou 'ilaka 'pito"), ('seventeen', 'alor1247-munas', "kər'tou 'ilak 'pito")}
 One or more Lamaholot dialects innovated forms not found in Alorese:
 {('seventeen', 'lama1277-kalik', 'ˈspulo no ˈpito'), ('seventeen', 'koto1251', 'boʔ es am hitu'), ('seventeen', 'lama1277-adona', 'pulok pito'), ('seventeen', 'lama1277-lewoi', 'pulo noʔoŋ ˈpitɔ'), ('seventeen', 'keda1252-leuba', "pulaʔ 'pitu"), ('seventeen', 'lama1277-lerek', 'heˈpulo no ˈpito'), ('seventeen', 'sika1262-hewa', 'pulu ʋot pitu')}


Concept ‘window’ is different between Alorese and Lamaholot.

 Alorese innovated the following forms:
 {('window', 'alor1247-munas', "pitu 'aŋiŋ"), ('window', 'alor1247-munas', "pitu 'anaŋ")}

  Just for clarity:
  There are also the following similarity classes shared between Alorese and LH.
  {('window', 'teiw1235', 'ʤandela'), ('window', 'buna1278-bobon', 'zanɛla'), ('window', 'tetu1245-vique', "ʤa'nela"), ('window', 'tetu1245-suai', "ʤa'nela"), ('window', 'kula1280-lanto', '’diŋgel'), ('window', 'keda1252-leuba', "dʒən'dela"), ('window', 'lama1277-lewoi', 'ʤənˈdela'), ('window', 'lama1277-adona', 'ɡɜˈnela'), ('window', 'alor1247-besar', "ʤa'nela"), ('window', 'rett1240', 'ʤanɛla'), ('window', 'sawi1256', "di'ŋela"), ('window', 'adan1251-otvai', 'sa’nɛlɛ'), ('window', 'wers1238-marit', 'ʤɛndɛl'), ('window', 'abui1241-ulaga', 'ɟendela'), ('window', 'buna1278-suai', 'zanɛla'), ('window', 'alor1247-pandai', "dʒ'nɛla"), ('window', 'sika1262-hewa', 'ʤendela')}


Concept ‘forty’ is different between Alorese and Lamaholot.

 Alorese shares the following forms with TAP languages:
 {('forty', 'teiw1235', 'qa:r ut'), ('forty', 'alor1247-pandai', "kar'pa"), ('forty', 'alor1247-besar', "kar'pa"), ('forty', 'adan1251-otvai', '’ʔer ’ut'), ('forty', 'pura1258', "ari'buta"), ('forty', 'abui1241-takal', "kär.'bu.ti"), ('forty', 'kelo1247-hopte', "kar 'ʔut"), ('forty', 'abui1241-ulaga', 'kar buti'), ('forty', 'rett1240', 'karɓuta'), ('forty', 'kaer1234', 'xar ut'), ('forty', 'adan1251-lawah', 'air wut'), ('forty', 'alor1247-munas', "kar'paʔ")}
 One or more Lamaholot dialects innovated forms not found in Alorese:
 {('forty', 'lama1277-kalik', 'pul pat'), ('forty', 'lama1277-adona', 'pulu paːt'), ('forty', 'lama1277-lerek', 'pul ˈpata'), ('forty', 'sika1262-hewa', 'pulu hutu'), ('forty', 'lama1277-lewoi', 'pulu ˈpaːt'), ('forty', 'keda1252-leuba', 'purun apaʔ')}


Concept snow is attested only in Alorese


Concept ‘shirt’ is different between Alorese and Lamaholot.

 Alorese shares the following forms with TAP languages:
 {('shirt', 'pura1258', 'konde'), ('shirt', 'teiw1235', 'kon'), ('shirt', 'adan1251-otvai', '’kɔd'), ('shirt', 'kaer1234', "ko'no"), ('shirt', 'alor1247-munas', 'kɔndɔ'), ('shirt', 'alor1247-besar', 'kɔnʤɔ'), ('shirt', 'lamm1241-westp', 'kunda'), ('shirt', 'rett1240', 'kɔndɔ'), ('shirt', 'alor1247-pandai', 'kɔndɔ')}
 One or more Lamaholot dialects innovated forms not found in Alorese:
 {('shirt', 'keda1252-leuba', 'labor'), ('shirt', 'lama1277-kalik', 'laˈbur'), ('shirt', 'lama1277-lerek', 'laˈbur'), ('shirt', 'sika1262-hewa', 'lɑbur'), ('shirt', 'lama1277-lewoi', 'ˈlabu'), ('shirt', 'lama1277-adona', 'ˈlabu')}


Concept ‘thirty’ is different between Alorese and Lamaholot.

 Alorese shares the following forms with TAP languages:
 {('thirty', 'kelo1247-hopte', "kar 'tɔŋ"), ('thirty', 'kama1365', 'ataːk su'), ('thirty', 'rett1240', 'karatoga'), ('thirty', 'dein1238', 'qaratig'), ('thirty', 'alor1247-besar', "kar'talːɔ"), ('thirty', 'kaer1234', 'xar tug'), ('thirty', 'baka1276', 'ari tue'), ('thirty', 'rett1240', 'karatɔga'), ('thirty', 'adan1251-lawah', 'air towoʔ'), ('thirty', 'adan1251-otvai', '’ʔer ’tou'), ('thirty', 'sarr1247-adiab', 'qar tig'), ('thirty', 'alor1247-munas', "kartə'lːɔ"), ('thirty', 'blag1240-tuntu', 'qar tuge'), ('thirty', 'abui1241-fuime', 'kar sua'), ('thirty', 'pura1258', "ari'tue"), ('thirty', 'kira1248', 'karsuwa'), ('thirty', 'kuii1253', 'karsiwa'), ('thirty', 'sarr1247-nule', 'qar tig'), ('thirty', 'abui1241-takal', "kär.'su.ä"), ('thirty', 'alor1247-pandai', "kar'talːɔ"), ('thirty', 'blag1240-bama', 'qar tuge'), ('thirty', 'teiw1235', 'qa:r jerig'), ('thirty', 'abui1241-ulaga', 'kar sua'), ('thirty', 'lamm1241-westp', 'keatiga'), ('thirty', 'kabo1247', "kar 'towo"), ('thirty', 'blag1240-nule', 'ar tue'), ('thirty', 'atim1239', 'kar suo')}
 One or more Lamaholot dialects innovated forms not found in Alorese:
 {('thirty', 'sika1262-hewa', 'pulu təlu'), ('thirty', 'lama1277-lerek', 'pul təl'), ('thirty', 'lama1277-kalik', 'pul təˈlu'), ('thirty', 'keda1252-leuba', 'purun tɛlu'), ('thirty', 'lama1277-lewoi', 'pulu ˈtɛlːo'), ('thirty', 'lama1277-adona', 'pulu ˈtɜlo')}


Concept ‘to_slap’ is different between Alorese and Lamaholot.

 Alorese shares the following forms with other AN languanges, but not Lamaholot:
 {('to_slap', 'alor1247-besar', 'lapa'), ('to_slap', 'kaer1234', "la'pa"), ('to_slap', 'keda1252-leuba', "le'paʔ"), ('to_slap', 'lamm1241-westp', 'lopa'), ('to_slap', 'alor1247-pandai', "lə'pːa"), ('to_slap', 'alor1247-munas', "lə'pa"), ('to_slap', 'alor1247-pandai', "lə'pːa 'kapuŋ"), ('to_slap', 'adan1251-otvai', '’baʔ')}
 One or more Lamaholot dialects innovated forms not found in Alorese:
 {('to_slap', 'buna1278-bobon', 'thas'), ('to_slap', 'buna1278-suai', 'thas'), ('to_slap', 'lama1277-lewoi', 'ˈtəpa'), ('to_slap', 'p-mala1545-acd', '*bebak'), ('to_slap', 'lama1277-kalik', 'təˈpa'), ('to_slap', 'fata1247', "taɁal-ci'palɛ"), ('to_slap', 'lama1277-adona', 'ˈtɜpɑk'), ('to_slap', 'maka1316', "ti'bala"), ('to_slap', 'p-mala1545-acd', '*tepak'), ('to_slap', 'lama1277-adona', 'ˈtɜpɔ̃'), ('to_slap', 'lama1277-lerek', 'təˈpa')}


Concept ‘twelve’ is different between Alorese and Lamaholot.

 Alorese innovated the following forms:
 {('twelve', 'alor1247-munas', "kər'tou 'ilak 'rua"), ('twelve', 'alor1247-besar', "kartou 'ilaka 'rua"), ('twelve', 'alor1247-pandai', "kə'rtou 'ilak 'rua")}
 One or more Lamaholot dialects innovated forms not found in Alorese:
 {('twelve', 'sika1262-hewa', 'pulu ʋot rua'), ('twelve', 'tetu1245-suai', 'sanulu resin rua'), ('twelve', 'lama1277-lerek', 'heˈpulo no ˈdʒ͡ua'), ('twelve', 'lama1277-adona', 'pulok rua'), ('twelve', 'tetu1245-vique', 'sanulu resin rua'), ('twelve', 'lama1277-lewoi', 'pulo noʔoŋ ˈrua'), ('twelve', 'lama1277-kalik', 'ˈspulo no ˈʤua'), ('twelve', 'keda1252-leuba', "pulaʔ su'we")}


Concept ‘fourteen’ is different between Alorese and Lamaholot.

 Alorese innovated the following forms:
 {('fourteen', 'alor1247-besar', "kartou 'ilaka 'pa"), ('fourteen', 'alor1247-pandai', "kə'rtou 'ilak 'pa"), ('fourteen', 'alor1247-munas', "kər'tou 'ilak 'paʔ")}
 One or more Lamaholot dialects innovated forms not found in Alorese:
 {('fourteen', 'keda1252-leuba', "pulaʔ 'apaʔ"), ('fourteen', 'lama1277-lewoi', 'pulo noʔoŋ ˈpaː'), ('fourteen', 'lama1277-adona', 'pulok paːt'), ('fourteen', 'lama1277-kalik', 'ˈspulo no pat'), ('fourteen', 'lama1277-lerek', 'heˈpulo no ˈpatə')}


Concept ice is attested only in Alorese


Concept ‘lamp’ is different between Alorese and Lamaholot.

 Alorese shares the following forms with TAP languages:
 {('lamp', 'fata1247', 'lampu'), ('lamp', 'alor1247-besar', 'lampu'), ('lamp', 'alor1247-pandai', 'lampo'), ('lamp', 'alor1247-munas', 'lampɔʔ'), ('lamp', 'maka1316', 'lambu')}
 One or more Lamaholot dialects innovated forms not found in Alorese:
 {('lamp', 'wers1238-marit', 'lampur'), ('lamp', 'sawi1256', 'lampur'), ('lamp', 'kula1280-lanto', '’lampu'), ('lamp', 'sika1262-hewa', 'lampu'), ('lamp', 'lama1277-adona', 'lampu'), ('lamp', 'kama1365', 'laŋpu'), ('lamp', 'pura1258', 'lampu'), ('lamp', 'adan1251-otvai', '’lampu'), ('lamp', 'lama1277-lewoi', 'ˈlampu')}
 {('lamp', 'lama1277-adona', 'pɜlita'), ('lamp', 'lamm1241-westp', 'pelita'), ('lamp', 'lama1277-lerek', 'pəˈlita'), ('lamp', 'keda1252-leuba', "pə'lita")}


Concept ‘cuscus’ is different between Alorese and Lamaholot.

 Alorese innovated the following forms:
 {('cuscus', 'alor1247-pandai', 'kuskus')}
 One or more Lamaholot dialects innovated forms not found in Alorese:
 {('cuscus', 'lama1277-lewoi', 'kəbeˈheʔ')}


Concept ‘guilty’ is different between Alorese and Lamaholot.

 Alorese innovated the following forms:
 {('guilty', 'alor1247-munas', "mariŋ 'go 'hala")}

  Just for clarity:
  There are also the following similarity classes shared between Alorese and LH.
  {('guilty', 'koto1251', 'n-sana'), ('guilty', 'buna1278-suai', 'sal'), ('guilty', 'lama1277-lerek', 'hənaləˈkən'), ('guilty', 'lama1277-kalik', 'snaˈlak'), ('guilty', 'fata1247', 'sala'), ('guilty', 'tetu1245-suai', 'sa:la'), ('guilty', 'lama1277-lewoi', 'ˈnalɑŋ'), ('guilty', 'lama1277-adona', 'ˈnalã'), ('guilty', 'alor1247-besar', 'hala'), ('guilty', 'maka1316', 'sala'), ('guilty', 'buna1278-bobon', 'sal'), ('guilty', 'tetu1245-vique', 'sa:la'), ('guilty', 'alor1247-pandai', 'hala'), ('guilty', 'sika1262-hewa', "dəna 'hala")}


Concept ‘to_die’ is different between Alorese and Lamaholot.

 One or more Lamaholot dialects innovated forms not found in Alorese:
 {('to_die', 'lama1277-lewok', 'belu')}

  Just for clarity:
  There are also the following similarity classes shared between Alorese and LH.
  {('to_die', 'lama1277-horow', 'mata'), ('to_die', 'lama1277-mulan', 'mate'), ('to_die', 'alor1247-pandai', 'mate'), ('to_die', 'lama1277-baipi', 'mata'), ('to_die', 'alor1247-besar', 'mati'), ('to_die', 'mamb1306', 'maɛt'), ('to_die', 'p-cent2245-abvd', '*matay'), ('to_die', 'alor1247-munas', 'mate'), ('to_die', 'lama1277-lewom', 'mata'), ('to_die', 'lama1277-ritae', 'mata'), ('to_die', 'idat1237', 'mɑte'), ('to_die', 'lama1277-dulhi', 'matẽ'), ('to_die', 'lama1277-lewop', 'mataj'), ('to_die', 'lama1277-pukau', 'matĩ'), ('to_die', 'lama1277-merde', 'mataya'), ('to_die', 'tetu1245-suai', 'matɛ'), ('to_die', 'lama1277-lewog', 'mata'), ('to_die', 'lama1277-lamak', 'mata'), ('to_die', 'sika1262-maume', 'mat̪en'), ('to_die', 'lama1277-lamah', 'mataj'), ('to_die', 'p-aust1307-abvd', '*m-aCay'), ('to_die', 'sika1262-tanai', 'mate'), ('to_die', 'lama1277-wuake', 'mataj'), ('to_die', 'kema1243', 'mate'), ('to_die', 'lama1277-kalik', 'matek'), ('to_die', 'keda1252', 'mate'), ('to_die', 'lama1277-botun', 'mate'), ('to_die', 'lama1277-lamat', 'mata'), ('to_die', 'laka1255', 'matɛ'), ('to_die', 'lama1277-lewoe', 'mata'), ('to_die', 'tuku1254', 'mate'), ('to_die', 'p-mala1545-acd', '*m-atay'), ('to_die', 'lama1277-belan', 'mate'), ('to_die', 'lama1277-kalik', 'mataj'), ('to_die', 'lama1277-minga', 'mataj'), ('to_die', 'tetu1245-vique', 'matɛ'), ('to_die', 'lama1277-waiwa', 'mate'), ('to_die', 'tetu1246', 'mɑtɛ'), ('to_die', 'lama1277-lamal', 'mata'), ('to_die', 'lama1277-adona', 'ˈmataː'), ('to_die', 'lama1277-ileap', 'mataj'), ('to_die', 'lama1277-lewuk', 'mataj'), ('to_die', 'lama1277-waiba', 'mata'), ('to_die', 'mamb1306', 'mat'), ('to_die', 'lama1277-tanju', 'mata'), ('to_die', 'lama1277-lewoi', 'ˈmata'), ('to_die', 'lama1277-kiwan', 'mata'), ('to_die', 'alor1247-baran', 'matɛ'), ('to_die', 'koto1251', 'n-mate'), ('to_die', 'lama1277-lerek', 'maˈtei'), ('to_die', 'p-mala1545-abvd', '*m-atay'), ('to_die', 'keda1252-leuba', 'mate'), ('to_die', 'lama1277-bama', 'mata'), ('to_die', 'lama1277-lewot', 'mataj'), ('to_die', 'sika1262-hewa', 'mate'), ('to_die', 'lama1277-lewob', 'mata'), ('to_die', 'lama1277-paina', 'mateyəg'), ('to_die', 'lama1277-watan', 'mata'), ('to_die', 'lama1277-imulo', 'mataja')}


Concept ‘nineteen’ is different between Alorese and Lamaholot.

 Alorese innovated the following forms:
 {('nineteen', 'alor1247-pandai', "kə'rtou 'ilak 'hiwa"), ('nineteen', 'alor1247-munas', "kər'tou 'ilak 'hiwa"), ('nineteen', 'alor1247-besar', "kartou 'ilaka 'hifa")}
 One or more Lamaholot dialects innovated forms not found in Alorese:
 {('nineteen', 'lama1277-lerek', 'heˈpulo no ˈhiva'), ('nineteen', 'lama1277-lewoi', 'pulo noʔoŋ ˈhiwa'), ('nineteen', 'sika1262-hewa', 'pulu ʋot hiʋa'), ('nineteen', 'lama1277-kalik', 'ˈspulo no ˈsiva'), ('nineteen', 'lama1277-adona', 'pulok hiwa')}


Concept ‘candle’ is different between Alorese and Lamaholot.

 Alorese innovated the following forms:
 {('candle', 'alor1247-pandai', "padu 'nomal")}
 {('candle', 'alor1247-munas', 'tapal')}
 {('candle', 'wers1238-marit', "du'paiŋ"), ('candle', 'kula1280-lanto', 'dəp’wan'), ('candle', 'adan1251-otvai', '’pa:n'), ('candle', 'alor1247-besar', 'panʤu'), ('candle', 'kelo1247-hopte', 'pa:n'), ('candle', 'sawi1256', "idu'pani"), ('candle', 'abui1241-ulaga', 'pa:n')}
 Alorese shares the following forms with TAP languages:
 {('candle', 'alor1247-pandai', "padu 'nomal")}
 {('candle', 'alor1247-munas', 'tapal')}
 {('candle', 'wers1238-marit', "du'paiŋ"), ('candle', 'kula1280-lanto', 'dəp’wan'), ('candle', 'adan1251-otvai', '’pa:n'), ('candle', 'alor1247-besar', 'panʤu'), ('candle', 'kelo1247-hopte', 'pa:n'), ('candle', 'sawi1256', "idu'pani"), ('candle', 'abui1241-ulaga', 'pa:n')}
 One or more Lamaholot dialects innovated forms not found in Alorese:
 {('candle', 'lama1277-lerek', 'ˈudor')}
 {('candle', 'sika1262-hewa', 'taru'), ('candle', 'lama1277-lewoi', 'təˈnarɔʔ'), ('candle', 'lama1277-adona', 'tɜˈnaro')}
 {('candle', 'teiw1235', 'lilin'), ('candle', 'keda1252-leuba', 'lilin'), ('candle', 'lama1277-lerek', 'lilin'), ('candle', 'buna1278-suai', 'lilin'), ('candle', 'tetu1245-suai', 'lɪlɪŋ'), ('candle', 'abui1241-ulaga', 'lilin'), ('candle', 'abui1241-takal', 'li.lin'), ('candle', 'tetu1245-vique', 'lɪlɪŋ'), ('candle', 'buna1278-bobon', 'lilin'), ('candle', 'kaer1234', 'lilin')}


Concept ‘2pl’ is different between Alorese and Lamaholot.

 Alorese innovated the following forms:
 {('2pl', 'alor1247-baran', ",mi sa'kali")}
 One or more Lamaholot dialects innovated forms not found in Alorese:
 {('2pl', 'lama1277-lerek', 'ˈmio woˈləm')}

  Just for clarity:
  There are also the following similarity classes shared between Alorese and LH.
  {('2pl', 'p-mala1545-acd', '*amu'), ('2pl', 'laka1255', 'ɪme'), ('2pl', 'lama1277-adona', 'ˈmio'), ('2pl', 'sika1262-tanai', 'miu'), ('2pl', 'idat1237', 'ʔemi'), ('2pl', 'alor1247-besar', 'mi'), ('2pl', 'p-mala1545-acd', '*kamu'), ('2pl', 'alor1247-pandai', 'mi'), ('2pl', 'alor1247-munas', 'mi'), ('2pl', 'lama1277-lewoi', 'ˈmio'), ('2pl', 'sika1262-maume', 'miu'), ('2pl', 'tetu1246', 'ʔimi'), ('2pl', 'tetu1245-suai', 'imi'), ('2pl', 'sika1262-hewa', 'miu'), ('2pl', 'tetu1245-vique', 'imi'), ('2pl', 'keda1252-leuba', 'meː'), ('2pl', 'tuku1254', 'kimi'), ('2pl', 'keda1252-leuwa', 'mε'), ('2pl', 'kema1243', 'ʔimi'), ('2pl', 'p-aust1307-abvd', '*i-kamu'), ('2pl', 'lama1277-kalik', 'ˈmio')}


Concept ‘rain’ is different between Alorese and Lamaholot.

 One or more Lamaholot dialects innovated forms not found in Alorese:
 {('rain', 'abui1241-ulaga', 'anui'), ('rain', 'blag1240-bama', 'onar'), ('rain', 'adan1251-otvai', '’nɔi'), ('rain', 'abui1241-takal', 'ä.nui'), ('rain', 'buna1278-malia', "ʔi'nɛl"), ('rain', 'kabo1247', 'nui'), ('rain', 'buna1278-suai', 'inɛl'), ('rain', 'lama1277-lewok', 'apunu'), ('rain', 'p-timo1261', '*anu(r, R)'), ('rain', 'p-alor1249', '*anur'), ('rain', 'buna1278-bobon', 'inɛl'), ('rain', 'kelo1247-hopte', "ʔə'nu:r"), ('rain', 'kuii1253', 'anor'), ('rain', 'kira1248', 'aˈnor'), ('rain', 'blag1240-tuntu', 'onor'), ('rain', 'abui1241-fuime', 'anui'), ('rain', 'blag1240-warsa', 'onor'), ('rain', 'blag1240-kulij', 'nuwar'), ('rain', 'adan1251-lawah', 'nui'), ('rain', 'atim1239', 'anui'), ('rain', 'baka1276', 'noar'), ('rain', 'p-east2519', '*ine(r, R)'), ('rain', 'blag1240-nule', 'nuwar')}

  Just for clarity:
  There are also the following similarity classes shared between Alorese and LH.
  {('rain', 'lama1277-bama', 'urã'), ('rain', 'lama1277-botun', 'urã'), ('rain', 'lama1277-lamah', 'urã'), ('rain', 'lama1277-horow', 'urã'), ('rain', 'lama1277-kalik', 'uˈʤan'), ('rain', 'keda1252-leuwa', 'u̘ja'), ('rain', 'lama1277-kalik', 'ujan'), ('rain', 'lama1277-ileap', 'uran'), ('rain', 'lama1277-paina', 'ujan'), ('rain', 'lama1277-lewop', 'ujan'), ('rain', 'lama1277-minga', 'ujau'), ('rain', 'lama1277-pukau', 'urã'), ('rain', 'sika1262-hewa', 'uran'), ('rain', 'koto1251', 'uran'), ('rain', 'lama1277-lewog', 'urã'), ('rain', 'lama1277-dulhi', 'urut'), ('rain', 'alor1247-pandai', 'uraŋ'), ('rain', 'lama1277-lerek', 'ˈudʒ͡an'), ('rain', 'keda1252-leuba', "u'ja"), ('rain', 'lama1277-belan', 'ura'), ('rain', 'p-cent2245-abvd', '*quzan'), ('rain', 'tetu1245-vique', 'udaŋ'), ('rain', 'lama1277-watan', 'urã'), ('rain', 'lama1277-merde', 'urã'), ('rain', 'tetu1245-suai', 'udaŋ'), ('rain', 'lama1277-lewuk', 'ujan'), ('rain', 'tuku1254', 'ʔura'), ('rain', 'lama1277-waiwa', 'urã'), ('rain', 'sika1262-tanai', 'ura-n'), ('rain', 'p-mala1545-abvd', '*quzan'), ('rain', 'lama1277-baipi', 'urã'), ('rain', 'lama1277-lamat', 'urã'), ('rain', 'alor1247-munas', "uraŋ 'tuŋ"), ('rain', 'lama1277-mulan', 'urɤ'), ('rain', 'alor1247-besar', 'uraŋ'), ('rain', 'p-mala1545-acd', '*quzan'), ('rain', 'keda1252', 'uya'), ('rain', 'lama1277-lamak', 'urã'), ('rain', 'alor1247-munas', 'uraŋ'), ('rain', 'sika1262-maume', 'uran'), ('rain', 'lama1277-lewoe', 'urã'), ('rain', 'lama1277-lewob', 'urã'), ('rain', 'lama1277-tanju', 'urã'), ('rain', 'lama1277-imulo', 'ujan'), ('rain', 'lama1277-lewoi', 'ˈuraŋ'), ('rain', 'lama1277-lamal', 'urã'), ('rain', 'lama1277-lewot', 'ujan'), ('rain', 'p-aust1307-abvd', '*quzaN'), ('rain', 'lama1277-kiwan', 'urut'), ('rain', 'lama1277-wuake', 'ura'), ('rain', 'lama1277-adona', 'ˈʔurɑŋ'), ('rain', 'lama1277-ritae', 'urã'), ('rain', 'lama1277-waiba', 'urã'), ('rain', 'lama1277-lewom', 'urã')}


Concept ‘sun’ is different between Alorese and Lamaholot.

 One or more Lamaholot dialects innovated forms not found in Alorese:
 {('sun', 'lama1277-lerek', 'ˈluvak ləˈd͡ʒau'), ('sun', 'lama1277-paina', 'luwak'), ('sun', 'lama1277-lamat', 'luwa'), ('sun', 'lama1277-lewuk', 'luwak'), ('sun', 'lama1277-kalik', 'luˈvak'), ('sun', 'lama1277-kalik', 'luwak')}

  Just for clarity:
  There are also the following similarity classes shared between Alorese and LH.
  {('sun', 'lama1277-lewom', 'rəra'), ('sun', 'sika1262-hewa', 'ləro'), ('sun', 'lama1277-lewog', 'ləra'), ('sun', 'alor1247-besar', "la'ra"), ('sun', 'lama1277-merde', 'ləra'), ('sun', 'lama1277-pukau', 'ləra'), ('sun', 'lama1277-dulhi', 'ləra'), ('sun', 'lama1277-ritae', 'ləra'), ('sun', 'alor1247-pandai', "lə'rːa"), ('sun', 'lama1277-adona', 'ˈrɜra'), ('sun', 'lama1277-lewop', 'ləja'), ('sun', 'tuku1254', 'lɛlɔ'), ('sun', 'lama1277-horow', 'ləra'), ('sun', 'alor1247-baran', "la'ra"), ('sun', 'lama1277-bama', 'ləra'), ('sun', 'lama1277-lewoe', 'ləra'), ('sun', 'lama1277-waiwa', 'ləra'), ('sun', 'lama1277-wuake', 'ləra'), ('sun', 'sika1262-tanai', 'ləro'), ('sun', 'lama1277-mulan', 'ləra'), ('sun', 'lama1277-waiba', 'ləra'), ('sun', 'lama1277-lamal', 'ləra'), ('sun', 'lama1277-tanju', 'ləra'), ('sun', 'lama1277-watan', 'rəra'), ('sun', 'lama1277-lewob', 'ləra'), ('sun', 'lama1277-kiwan', 'rəra'), ('sun', 'lama1277-imulo', 'ləjaf'), ('sun', 'lama1277-lewot', 'ləjaf'), ('sun', 'lama1277-lamah', 'ləra'), ('sun', 'keda1252-leuba', "lɔ'jɔ"), ('sun', 'lamm1241-westp', 'marari'), ('sun', 'lama1277-belan', 'ləra'), ('sun', 'lama1277-lewok', 'ləjawu'), ('sun', 'alor1247-munas', "lə'rːa"), ('sun', 'lama1277-lewoi', 'ˈləraː'), ('sun', 'lama1277-lamak', 'rəra'), ('sun', 'lama1277-ileap', 'ləra'), ('sun', 'lama1277-botun', 'rəra'), ('sun', 'lama1277-baipi', 'rəra'), ('sun', 'lama1277-minga', 'ləja')}


Concept ‘father’ is different between Alorese and Lamaholot.

 One or more Lamaholot dialects innovated forms not found in Alorese:
 {('father', 'lama1277-lewuk', 'bapa'), ('father', 'lama1277-waiba', 'bapa'), ('father', 'lama1277-lewob', 'bapa'), ('father', 'lama1277-lewom', 'baba'), ('father', 'lama1277-lamak', 'bapa'), ('father', 'maka1316', 'baba'), ('father', 'p-mala1545-acd', '*baba'), ('father', 'lama1277-ritae', 'bapa'), ('father', 'lama1277-lamal', 'bapa'), ('father', 'lama1277-baipi', 'bapaʔ'), ('father', 'lama1277-watan', 'bapa'), ('father', 'lama1277-pukau', 'baʔ'), ('father', 'p-mala1545-acd', '*aba'), ('father', 'lama1277-mulan', 'bapa'), ('father', 'lama1277-lewog', 'bapa'), ('father', 'lama1277-bama', 'bapa'), ('father', 'lamm1241-westp', 'baba'), ('father', 'tuku1254', "a'pã:"), ('father', 'lama1277-tanju', 'bapa'), ('father', 'lama1277-lewoi', 'ˈbapaʔ'), ('father', 'lama1277-botun', 'bapa')}

  Just for clarity:
  There are also the following similarity classes shared between Alorese and LH.
  {('father', 'p-aust1307-abvd', '*t-ama'), ('father', 'keda1252', 'ame'), ('father', 'lama1277-merde', 'ama'), ('father', 'lama1277-adona', 'ˈʔama'), ('father', 'lama1277-lerek', 'ama'), ('father', 'lama1277-lamat', 'ama'), ('father', 'lama1277-paina', 'amay'), ('father', 'lama1277-wuake', 'ama'), ('father', 'alor1247-munas', 'mama'), ('father', 'lama1277-kalik', 'ama'), ('father', 'idat1237', 'ʔamɑn'), ('father', 'atim1239', 'mama'), ('father', 'p-cent2245-abvd', '*ama'), ('father', 'lama1277-imulo', 'ama'), ('father', 'lama1277-lewot', 'ama'), ('father', 'alor1247-munas', 'ama'), ('father', 'abui1241-takal', "mä:.'mä"), ('father', 'keda1252-leuwa', 'a̘mɔ'), ('father', 'lama1277-lewoe', 'ama'), ('father', 'tetu1245-suai', 'ama'), ('father', 'sika1262-tanai', 'ama'), ('father', 'abui1241-ulaga', 'ama ɣema'), ('father', 'lama1277-lewop', 'ma'), ('father', 'lama1277-belan', 'ama'), ('father', 'lama1277-kalik', 'ˈama'), ('father', 'alor1247-besar', 'amã'), ('father', 'koto1251', 'amaf'), ('father', 'mamb1306', 'ʔama'), ('father', 'buna1278-suai', 'ama'), ('father', 'p-alor1249', '*-mam'), ('father', 'lama1277-horow', 'ama'), ('father', 'kema1243', "a'mɑr"), ('father', 'p-mala1545-acd', '*ama'), ('father', 'buna1278-malia', 'ʔamɑ'), ('father', 'keda1252-leuba', 'ʔame'), ('father', 'lama1277-ileap', 'ama'), ('father', 'abui1241-fuime', 'mama'), ('father', 'lama1277-waiwa', 'ama'), ('father', 'alor1247-baran', 'ʔama'), ('father', 'buna1278-bobon', 'ama'), ('father', 'laka1255', 'amɑn'), ('father', 'alor1247-munas', 'amaŋ'), ('father', 'lama1277-lewok', 'ama'), ('father', 'lama1277-kiwan', 'ama'), ('father', 'lama1277-dulhi', 'ama'), ('father', 'alor1247-pandai', 'mama'), ('father', 'sika1262-hewa', 'ama'), ('father', 'keda1252-leuwa', 'amε'), ('father', 'sika1262-maume', 'ama'), ('father', 'p-mala1545-abvd', '*t-ama'), ('father', 'lama1277-lamah', 'amakə'), ('father', 'lama1277-minga', 'ama'), ('father', 'tetu1246', 'ʔɑmɑn')}


Concept ‘fingernail’ is different between Alorese and Lamaholot.

 Alorese shares the following forms with other AN languanges, but not Lamaholot:
 {('fingernail', 'alor1247-pandai', 'nuŋgul'), ('fingernail', 'p-mala1545-acd', '*k<an>uhkuh'), ('fingernail', 'p-mala1545-acd', '*kuhkuh'), ('fingernail', 'maka1316', 'tana kuli'), ('fingernail', 'alor1247-munas', 'nugul'), ('fingernail', 'alor1247-baran', "ta'nuŋgul"), ('fingernail', 'alor1247-besar', "tanaŋ'guluŋ")}
 One or more Lamaholot dialects innovated forms not found in Alorese:
 {('fingernail', 'lama1277-lerek', 'kaˈtau')}
 {('fingernail', 'lama1277-adona', 'kɜˈmukurɜt')}
 {('fingernail', 'lama1277-lewoi', 'tənuˈʔi'), ('fingernail', 'lama1277-kalik', 'tənuˈmək')}


Concept ‘twenty’ is different between Alorese and Lamaholot.

 Alorese shares the following forms with other AN languanges, but not Lamaholot:
 {('twenty', 'hama1240', 'air alo'), ('twenty', 'baka1276', 'ar iaru'), ('twenty', 'adan1251-otvai', '’ʔera’lo'), ('twenty', 'sarr1247-adiab', 'qaraq'), ('twenty', 'kaer1234', "xar 'raxo"), ('twenty', 'abui1241-fuime', 'kar ajoku'), ('twenty', 'rett1240', 'karalɔ'), ('twenty', 'adan1251-lawah', 'airialu'), ('twenty', 'blag1240-tuntu', 'qar akur'), ('twenty', 'alor1247-munas', 'karua'), ('twenty', 'blag1240-kulij', 'ari aru'), ('twenty', 'blag1240-warsa', 'xar akur'), ('twenty', 'kira1248', 'karaijoku'), ('twenty', 'kuii1253', 'kararoku'), ('twenty', 'kabo1247', 'kar ʤolo'), ('twenty', 'teiw1235', 'qa:r raq'), ('twenty', 'abui1241-takal', "kär.'ä.jo.qu"), ('twenty', 'rett1240', 'karalo'), ('twenty', 'kelo1247-hopte', "kar ʔə'ruk"), ('twenty', 'sarr1247-nule', 'qar rag'), ('twenty', 'alor1247-besar', 'karua'), ('twenty', 'blag1240-bama', 'qar akur'), ('twenty', 'nede1245', 'kar:aku'), ('twenty', 'kelo1247-bring', 'kɑrərok'), ('twenty', 'blag1240-nule', 'ariaru'), ('twenty', 'abui1241-ulaga', 'kar a:ki'), ('twenty', 'alor1247-baran', "ka:'rua"), ('twenty', 'kafo1240', 'kɑr.iʤɑk.u'), ('twenty', 'tuku1254', "sɑku'ru:"), ('twenty', 'dein1238', 'qar:aq'), ('twenty', 'alor1247-pandai', 'karua'), ('twenty', 'atim1239', 'kar ajoku'), ('twenty', 'pura1258', "ari'a ru"), ('twenty', 'lamm1241-westp', 'kealaku')}
 One or more Lamaholot dialects innovated forms not found in Alorese:
 {('twenty', 'sika1262-hewa', 'pulu rua'), ('twenty', 'lama1277-kalik', 'ˈpuluˈʤua'), ('twenty', 'lama1277-adona', 'pulu rua'), ('twenty', 'lama1277-lewoi', 'pulu ˈrua'), ('twenty', 'keda1252-leuba', "purun 'suwe"), ('twenty', 'lama1277-lerek', 'pul ˈdʒ͡ua'), ('twenty', 'kema1243', ",gulu 'rua")}


Concept ‘sixteen’ is different between Alorese and Lamaholot.

 Alorese innovated the following forms:
 {('sixteen', 'alor1247-besar', "kartou 'ilaka 'namuŋ"), ('sixteen', 'alor1247-munas', "kər'tou 'ilak nə'mu"), ('sixteen', 'alor1247-pandai', "kə'rtou 'ilak na'mːu")}
 One or more Lamaholot dialects innovated forms not found in Alorese:
 {('sixteen', 'keda1252-leuba', "pulaʔ 'ɛnɛŋ"), ('sixteen', 'lama1277-lerek', 'heˈpulo no əˈnam'), ('sixteen', 'sika1262-hewa', 'pulu ʋot əna'), ('sixteen', 'lama1277-kalik', 'ˈspulo no əˈnəm'), ('sixteen', 'lama1277-adona', 'pulok nɜmu'), ('sixteen', 'lama1277-lewoi', 'pulo noʔoŋ ˈnəmuŋ')}


Concept ‘sixty’ is different between Alorese and Lamaholot.

 Alorese shares the following forms with TAP languages:
 {('sixty', 'teiw1235', 'qa:r tia:m'), ('sixty', 'abui1241-takal', "kär.'tä.lä.mä"), ('sixty', 'alor1247-pandai', "karna'mːu"), ('sixty', 'kelo1247-hopte', "kar tə'la:n"), ('sixty', 'kaer1234', "xar ti'am"), ('sixty', 'abui1241-ulaga', 'kar talama'), ('sixty', 'alor1247-munas', "karnə'mu"), ('sixty', 'alor1247-besar', "kar'namuŋ"), ('sixty', 'adan1251-otvai', '’ʔer ta’la:ŋ'), ('sixty', 'adan1251-lawah', 'air talaŋ'), ('sixty', 'rett1240', 'kartalauŋ'), ('sixty', 'pura1258', "ari'talIŋ")}
 One or more Lamaholot dialects innovated forms not found in Alorese:
 {('sixty', 'lama1277-lerek', 'pul əˈnəm'), ('sixty', 'lama1277-lewoi', 'pulu ˈnəmuŋ'), ('sixty', 'keda1252-leuba', 'purun ɛnɛŋ'), ('sixty', 'lama1277-adona', 'pulu nɜmu'), ('sixty', 'sika1262-hewa', 'pulu əna'), ('sixty', 'lama1277-kalik', 'pul ˈənəm')}


Concept ‘year’ is different between Alorese and Lamaholot.

 Alorese innovated the following forms:
 {('year', 'alor1247-munas', "nuŋ-'nuŋ"), ('year', 'alor1247-munas', 'nuŋ')}
 One or more Lamaholot dialects innovated forms not found in Alorese:
 {('year', 'lama1277-lewom', 'sũ'), ('year', 'lama1277-baipi', 'sũ'), ('year', 'lama1277-pukau', 'sũ'), ('year', 'lama1277-lewoi', 'ˈsuːŋ'), ('year', 'lama1277-tanju', 'sũ'), ('year', 'lama1277-bama', 'sũ'), ('year', 'lama1277-lewog', 'sũ'), ('year', 'lama1277-lewob', 'sũ'), ('year', 'lama1277-waiba', 'sũ')}

  Just for clarity:
  There are also the following similarity classes shared between Alorese and LH.
  {('year', 'tetu1245-vique', 'tɪnaŋ'), ('year', 'buna1278-malia', 'thɔ'), ('year', 'p-mala1545-abvd', '*taqun'), ('year', 'lama1277-imulo', 'tunən'), ('year', 'alor1247-baran', 'tu:ŋ'), ('year', 'tetu1245-suai', 'tɪnaŋ'), ('year', 'lama1277-kalik', 'tunən'), ('year', 'alor1247-besar', 'tuŋ'), ('year', 'lama1277-botun', 'tun'), ('year', 'lama1277-wuake', 'tuŋ'), ('year', 'buna1278-bobon', 'thɔ:'), ('year', 'lama1277-watan', 'tun'), ('year', 'rett1240', 'tuŋ'), ('year', 'lama1277-ileap', 'tun'), ('year', 'abui1241-petle', 'tuŋ'), ('year', 'mamb1306', 'tɔ:n'), ('year', 'lama1277-lewuk', 'tunən'), ('year', 'kema1243', 'tɔnɑn'), ('year', 'lama1277-dulhi', 'tuŋ'), ('year', 'lama1277-kiwan', 'tũn'), ('year', 'kaer1234', 'tuŋ'), ('year', 'lama1277-mulan', 'tu'), ('year', 'laka1255', 'tinɑn'), ('year', 'abui1241-ulaga', 'tuŋ'), ('year', 'lama1277-lamak', 'tuŋ'), ('year', 'lama1277-minga', 'tunɤ'), ('year', 'idat1237', "ti'nan"), ('year', 'buna1278-suai', 'thɔ:'), ('year', 'lama1277-lewok', 'tunən'), ('year', 'lama1277-lewoe', 'tuŋ'), ('year', 'lamm1241-westp', 'tun:u'), ('year', 'tetu1246', "ti'nɑn"), ('year', 'teiw1235', 'tun'), ('year', 'lama1277-belan', 'tuŋ'), ('year', 'lama1277-paina', 'tunanən'), ('year', 'tuku1254', 'tɔ:'), ('year', 'adan1251-otvai', '’tun'), ('year', 'lama1277-lamah', 'tunə'), ('year', 'lama1277-ritae', 'tũ'), ('year', 'alor1247-munas', 'tuŋ'), ('year', 'lama1277-adona', 'tũː'), ('year', 'p-mala1545-acd', '*taqun'), ('year', 'keda1252-leuwa', 'tun'), ('year', 'keda1252-leuba', 'tun'), ('year', 'lama1277-lerek', 'ˈtunan'), ('year', 'lama1277-merde', 'tuŋ'), ('year', 'lama1277-horow', 'tuŋ'), ('year', 'lama1277-kalik', 'tun'), ('year', 'lama1277-lewot', 'tun'), ('year', 'keda1252', 'tun'), ('year', 'lama1277-lewop', 'tun'), ('year', 'koto1251', 'toon'), ('year', 'kama1365', 'tuŋ'), ('year', 'alor1247-pandai', 'tuŋ'), ('year', 'p-cent2245-abvd', '*taqun'), ('year', 'lama1277-lamal', 'tuŋ'), ('year', 'abui1241-takal', 'tu:ŋ'), ('year', 'lama1277-lamat', 'tũ'), ('year', 'kelo1247-hopte', 'tun'), ('year', 'lama1277-waiwa', 'tũ')}


Concept ‘mother’ is different between Alorese and Lamaholot.

 One or more Lamaholot dialects innovated forms not found in Alorese:
 {('mother', 'lama1277-lewom', 'əma'), ('mother', 'lama1277-lamal', 'əma'), ('mother', 'tuku1254', "a'mã:"), ('mother', 'lama1277-botun', 'əma'), ('mother', 'lama1277-ritae', 'əma'), ('mother', 'lama1277-waiba', 'əmaʔ'), ('mother', 'maka1316', 'mama'), ('mother', 'lama1277-lewuk', 'əma'), ('mother', 'lama1277-lewoi', 'ˈəˈmaʔ'), ('mother', 'lama1277-tanju', 'əma'), ('mother', 'lama1277-lamak', 'əma'), ('mother', 'buna1278-suai', 'ɛmɛ'), ('mother', 'lama1277-lewog', 'əmaʔ'), ('mother', 'lama1277-baipi', 'əmaʔ'), ('mother', 'lama1277-pukau', 'əmaʔ'), ('mother', 'buna1278-malia', 'ʔɛmɛ'), ('mother', 'sika1262-hewa', 'əma'), ('mother', 'buna1278-bobon', 'ɛmɛ'), ('mother', 'lama1277-lewob', 'əma'), ('mother', 'lama1277-bama', 'əma'), ('mother', 'lama1277-watan', 'əma')}

  Just for clarity:
  There are also the following similarity classes shared between Alorese and LH.
  {('mother', 'lama1277-minga', 'ina'), ('mother', 'koto1251', 'ainaf'), ('mother', 'p-aust1307-abvd', '*t-ina'), ('mother', 'sika1262-hewa', 'ina'), ('mother', 'kema1243', "i'nɑr"), ('mother', 'lama1277-dulhi', 'ina'), ('mother', 'lama1277-lerek', 'ina'), ('mother', 'alor1247-munas', 'ina'), ('mother', 'lama1277-kalik', 'ina'), ('mother', 'lama1277-mulan', 'ina'), ('mother', 'lama1277-lewop', 'ina'), ('mother', 'tetu1245-vique', 'naj'), ('mother', 'mamb1306', 'ʔina'), ('mother', 'p-mala1545-abvd', '*t-ina'), ('mother', 'keda1252', 'ine'), ('mother', 'alor1247-besar', 'inaŋ'), ('mother', 'alor1247-munas', 'inaŋ'), ('mother', 'p-cent2245-abvd', '*ina'), ('mother', 'laka1255', 'inɑn'), ('mother', 'lama1277-imulo', 'ina'), ('mother', 'lama1277-lewot', 'ina'), ('mother', 'lama1277-lamat', 'ina'), ('mother', 'lama1277-lewoe', 'ina'), ('mother', 'lama1277-kalik', 'ˈina'), ('mother', 'lama1277-waiwa', 'inaʔ'), ('mother', 'p-mala1545-acd', '*ina'), ('mother', 'lama1277-belan', 'ina'), ('mother', 'lama1277-lewok', 'ina'), ('mother', 'lama1277-kiwan', 'ina'), ('mother', 'lama1277-merde', 'ina'), ('mother', 'tetu1246', 'ʔinɑn'), ('mother', 'keda1252-leuba', 'ʔine'), ('mother', 'lama1277-paina', 'inan'), ('mother', 'lama1277-adona', 'ˈʔina'), ('mother', 'lama1277-horow', 'ina'), ('mother', 'alor1247-pandai', 'nina'), ('mother', 'alor1247-munas', 'nina'), ('mother', 'idat1237', 'ʔinak'), ('mother', 'sika1262-maume', 'ina'), ('mother', 'lama1277-ileap', 'ina'), ('mother', 'lama1277-wuake', 'ina'), ('mother', 'alor1247-baran', 'ʔina'), ('mother', 'tetu1245-suai', 'ina'), ('mother', 'sika1262-tanai', 'ina'), ('mother', 'lama1277-lamah', 'inakə')}


Concept ‘scorpion’ is different between Alorese and Lamaholot.

 Alorese shares the following forms with other AN languanges, but not Lamaholot:
 {('scorpion', 'alor1247-besar', 'ala'), ('scorpion', 'buna1278-bobon', 'ɛlɛ'), ('scorpion', 'buna1278-suai', 'uɛlɛ'), ('scorpion', 'alor1247-pandai', 'ala'), ('scorpion', 'alor1247-munas', 'ala'), ('scorpion', 'keda1252-leuba', 'ela')}
 One or more Lamaholot dialects innovated forms not found in Alorese:
 {('scorpion', 'lama1277-kalik', 'iˈlɔt'), ('scorpion', 'lama1277-lerek', 'iˈlot')}
 {('scorpion', 'lama1277-lewoi', 'kəˈmɛko'), ('scorpion', 'lama1277-adona', 'ˈmɛkɔt'), ('scorpion', 'sika1262-hewa', 'mekot')}


Concept ‘eighteen’ is different between Alorese and Lamaholot.

 Alorese innovated the following forms:
 {('eighteen', 'alor1247-pandai', "kə'rtou 'ilak 'buto"), ('eighteen', 'alor1247-munas', "kər'tou 'ilak 'buto"), ('eighteen', 'alor1247-besar', "kartou 'ilaka 'buto")}
 One or more Lamaholot dialects innovated forms not found in Alorese:
 {('eighteen', 'lama1277-adona', 'pulok buto'), ('eighteen', 'lama1277-kalik', 'ˈspulo no ˈbuto'), ('eighteen', 'lama1277-lerek', 'heˈpulo no ˈbuto'), ('eighteen', 'keda1252-leuba', "pulaʔ butu 'rai"), ('eighteen', 'lama1277-lewoi', 'pulo noʔoŋ ˈbutɔ')}


Concept ‘one_hundred_thousand’ is different between Alorese and Lamaholot.

 Alorese shares the following forms with other AN languanges, but not Lamaholot:
 {('one_hundred_thousand', 'alor1247-munas', "ratu 'ribu"), ('one_hundred_thousand', 'alor1247-pandai', 'ratu'), ('one_hundred_thousand', 'alor1247-besar', 'ratu'), ('one_hundred_thousand', 'keda1252-leuba', 'seratus ribu')}
 One or more Lamaholot dialects innovated forms not found in Alorese:
 {('one_hundred_thousand', 'teiw1235', 'riba ratu nuk'), ('one_hundred_thousand', 'lama1277-lewoi', 'ribu ˈtəratu'), ('one_hundred_thousand', 'lama1277-adona', 'ribhũ tɜˈratu'), ('one_hundred_thousand', 'lama1277-kalik', 'ˈribu ˈratu  tu'), ('one_hundred_thousand', 'lama1277-lerek', 'rib rat tu'), ('one_hundred_thousand', 'pura1258', "ribu ra'tunu"), ('one_hundred_thousand', 'adan1251-lawah', 'rib ratnu'), ('one_hundred_thousand', 'rett1240', 'ribu ratu anu'), ('one_hundred_thousand', 'adan1251-otvai', '’rib ’rat ’nu'), ('one_hundred_thousand', 'kaer1234', "ra'tu nu'ko")}


Concept ‘palm_of_hand’ is different between Alorese and Lamaholot.

 Alorese innovated the following forms:
 {('palm_of_hand', 'alor1247-pandai', "limaŋ p'lawak 'lɔlɔŋ")}
 One or more Lamaholot dialects innovated forms not found in Alorese:
 {('palm_of_hand', 'sika1262-hewa', 'lima əpak'), ('palm_of_hand', 'lama1277-kalik', 'liˈma əˈpak')}

  Just for clarity:
  There are also the following similarity classes shared between Alorese and LH.
  {('palm_of_hand', 'alor1247-pandai', "limaŋ 'ɔnɔŋ"), ('palm_of_hand', 'alor1247-besar', "limaŋ 'ɔnɔŋ"), ('palm_of_hand', 'tetu1245-suai', "lima'tanen"), ('palm_of_hand', 'lama1277-adona', 'ˈlimakɜt ˈʔonʔeʔ'), ('palm_of_hand', 'lama1277-lewoi', 'limaŋ ˈɔnəʔəŋ'), ('palm_of_hand', 'alor1247-munas', "limaŋ 'ɔnɔŋ"), ('palm_of_hand', 'alor1247-munas', "lima 'ɔnɔŋ"), ('palm_of_hand', 'lama1277-lerek', 'liˈma koˈnok'), ('palm_of_hand', 'tetu1245-vique', "lima'tanen")}


Concept ‘dog’ is different between Alorese and Lamaholot.

 One or more Lamaholot dialects innovated forms not found in Alorese:
 {('dog', 'keda1252-leuba', 'au'), ('dog', 'lama1277-lamal', 'ao'), ('dog', 'keda1252', 'au'), ('dog', 'lama1277-kalik', 'au'), ('dog', 'lama1277-belan', 'ao')}
 {('dog', 'lama1277-lewuk', 'aor'), ('dog', 'lama1277-kalik', 'aˈɔr'), ('dog', 'lama1277-imulo', 'aor'), ('dog', 'lama1277-lerek', 'aˈor'), ('dog', 'lama1277-lewot', 'aoru'), ('dog', 'lama1277-minga', 'aoru'), ('dog', 'lama1277-lewok', 'aoru'), ('dog', 'lama1277-lewop', 'aoru')}

  Just for clarity:
  There are also the following similarity classes shared between Alorese and LH.
  {('dog', 'alor1247-besar', 'aho'), ('dog', 'lama1277-wuake', 'aho'), ('dog', 'tuku1254', 'ʔasu'), ('dog', 'alor1247-munas', 'ahɔ'), ('dog', 'lama1277-adona', 'ˈʔaho'), ('dog', 'lama1277-bama', 'aho'), ('dog', 'sika1262-hewa', 'ahu'), ('dog', 'lama1277-mulan', 'aho'), ('dog', 'lama1277-watan', 'aho'), ('dog', 'lama1277-lamak', 'aho'), ('dog', 'sika1262-hewa', 'a̤hu'), ('dog', 'lama1277-tanju', 'aho'), ('dog', 'kema1243', 'ʔasu'), ('dog', 'tetu1245-suai', 'asu'), ('dog', 'lama1277-dulhi', 'aho'), ('dog', 'sika1262-maume', 'ahu'), ('dog', 'lama1277-waiba', 'aho'), ('dog', 'lama1277-botun', 'aho'), ('dog', 'p-mala1545-abvd', '*asu'), ('dog', 'lama1277-lamat', 'aho'), ('dog', 'lama1277-lamah', 'aho'), ('dog', 'sika1262-tanai', 'ahu'), ('dog', 'p-aust1307-abvd', '*asu'), ('dog', 'koto1251', 'asu'), ('dog', 'mamb1306', 'ʔa:s'), ('dog', 'lama1277-waiwa', 'aho'), ('dog', 'lama1277-baipi', 'aho'), ('dog', 'laka1255', "a'su:"), ('dog', 'alor1247-baran', "a'ho"), ('dog', 'lama1277-lewoi', 'ˈahɔʔ'), ('dog', 'idat1237', 'ɑsu'), ('dog', 'tetu1246', 'ʔɑsu'), ('dog', 'lama1277-kiwan', 'aho'), ('dog', 'alor1247-munas', 'hao'), ('dog', 'p-aust1307-abvd', '*wasu'), ('dog', 'lama1277-lewoe', 'aho'), ('dog', 'lama1277-lewog', 'aho'), ('dog', 'lama1277-pukau', 'aho'), ('dog', 'p-mala1545-acd', '*asu'), ('dog', 'lama1277-merde', 'aho'), ('dog', 'lama1277-lewob', 'aho'), ('dog', 'alor1247-pandai', 'aho'), ('dog', 'lama1277-lewom', 'aho'), ('dog', 'lama1277-ritae', 'aho'), ('dog', 'tetu1245-vique', 'asu'), ('dog', 'lama1277-paina', 'aho'), ('dog', 'lama1277-ileap', 'aho'), ('dog', 'lama1277-horow', 'aho'), ('dog', 'p-cent2245-abvd', '*asu')}


Concept ‘six’ is different between Alorese and Lamaholot.

 One or more Lamaholot dialects innovated forms not found in Alorese:
 {('six', 'p-mala1545-acd', '*enem'), ('six', 'kema1243', "hə'nɛm"), ('six', 'lama1277-lerek', 'əˈnam'), ('six', 'sika1262-hewa', 'əna'), ('six', 'lama1277-kalik', 'əˈnəm')}

  Just for clarity:
  There are also the following similarity classes shared between Alorese and LH.
  {('six', 'alor1247-pandai', "na'mːu"), ('six', 'fata1247', 'nɛmɛ'), ('six', 'alor1247-munas', "nə'mu"), ('six', 'lama1277-adona', 'ˈnamu'), ('six', 'lama1277-lewoi', 'ˈnəmuŋ'), ('six', 'alor1247-baran', 'nam:u'), ('six', 'alor1247-besar', 'namuŋ')}


Concept ‘salt’ is different between Alorese and Lamaholot.

 One or more Lamaholot dialects innovated forms not found in Alorese:
 {('salt', 'lama1277-lewop', 'irar'), ('salt', 'lama1277-lamat', 'hira'), ('salt', 'lama1277-kalik', 'irar'), ('salt', 'lama1277-kalik', 'iˈra'), ('salt', 'lama1277-minga', 'iraru'), ('salt', 'lama1277-paina', 'irar'), ('salt', 'lama1277-lewuk', 'irar'), ('salt', 'lama1277-lerek', 'iˈrar'), ('salt', 'lama1277-lewot', 'iraru'), ('salt', 'lama1277-lewok', 'iraru'), ('salt', 'lama1277-imulo', 'irar'), ('salt', 'lama1277-kalik', 'iˈrar')}
 {('salt', 'p-cent2245-abvd', '*tasik'), ('salt', 'keda1252', 'teʔu'), ('salt', 'keda1252-leuwa', 'tæʔu a̘pur'), ('salt', 'keda1252-leuba', "te'ʔu"), ('salt', 'lama1277-lewoe', 'taʔo')}

  Just for clarity:
  There are also the following similarity classes shared between Alorese and LH.
  {('salt', 'blag1240-nule', 'siah'), ('salt', 'lama1277-botun', 'siʔa'), ('salt', 'lama1277-ileap', 'sia'), ('salt', 'lama1277-adona', 'ˈsiʔa'), ('salt', 'lama1277-lewob', 'siʔa'), ('salt', 'mamb1306', 'sia'), ('salt', 'lama1277-belan', 'sia'), ('salt', 'alor1247-pandai', 'sia'), ('salt', 'lama1277-kiwan', 'siʔa'), ('salt', 'lama1277-bama', 'siʔa'), ('salt', 'lama1277-wuake', 'siɁa'), ('salt', 'lama1277-pukau', 'siʔa'), ('salt', 'lama1277-lewog', 'siʔa'), ('salt', 'lama1277-waiwa', 'sia'), ('salt', 'lama1277-lewoi', 'ˈsiʔa'), ('salt', 'lama1277-waiba', 'siʔa'), ('salt', 'pura1258', "si'a"), ('salt', 'lama1277-lamah', 'sia'), ('salt', 'lama1277-merde', 'siʔa'), ('salt', 'lama1277-horow', 'siʔa'), ('salt', 'kema1243', 'sia'), ('salt', 'lama1277-dulhi', 'siʔa'), ('salt', 'buna1278-malia', 'ʔi:'), ('salt', 'alor1247-baran', 'siʔa'), ('salt', 'baka1276', 'sia'), ('salt', 'lama1277-lamak', 'siʔa'), ('salt', 'lama1277-lewom', 'siʔa'), ('salt', 'lama1277-ritae', 'siʔa'), ('salt', 'lama1277-watan', 'siʔa'), ('salt', 'tuku1254', 'sia'), ('salt', 'blag1240-kulij', 'sija'), ('salt', 'alor1247-besar', 'sia'), ('salt', 'lama1277-mulan', 'sia'), ('salt', 'lama1277-baipi', 'siʔa'), ('salt', 'lama1277-tanju', 'siʔa'), ('salt', 'alor1247-munas', 'sia'), ('salt', 'lama1277-lamal', 'sia')}


Concept ‘to_rule’ is different between Alorese and Lamaholot.

 Alorese innovated the following forms:
 {('to_rule', 'alor1247-pandai', "kə'rːa")}
 {('to_rule', 'alor1247-pandai', 'gahiŋ'), ('to_rule', 'alor1247-besar', 'gahiŋ'), ('to_rule', 'pura1258', 'gahiŋ')}
 {('to_rule', 'alor1247-munas', "sɔrak 'sɔgaŋ")}
 Alorese shares the following forms with TAP languages:
 {('to_rule', 'alor1247-pandai', "kə'rːa")}
 {('to_rule', 'alor1247-pandai', 'gahiŋ'), ('to_rule', 'alor1247-besar', 'gahiŋ'), ('to_rule', 'pura1258', 'gahiŋ')}
 {('to_rule', 'alor1247-munas', "sɔrak 'sɔgaŋ")}
 One or more Lamaholot dialects innovated forms not found in Alorese:
 {('to_rule', 'sika1262-hewa', 'plɛta'), ('to_rule', 'lama1277-adona', 'pɜˈreta'), ('to_rule', 'kelo1247-hopte', 'prɛnta'), ('to_rule', 'teiw1235', "sarin'taɁ"), ('to_rule', 'wers1238-marit', 'prɛn'), ('to_rule', 'keda1252-leuba', "pa'rɛnta"), ('to_rule', 'lama1277-lerek', 'ˈprinta'), ('to_rule', 'lamm1241-westp', 'prenta'), ('to_rule', 'sawi1256', "gira pa'renta"), ('to_rule', 'lama1277-kalik', 'preˈta'), ('to_rule', 'lama1277-lewoi', 'pəˈrinta'), ('to_rule', 'kula1280-lanto', 'pa’renta'), ('to_rule', 'adan1251-otvai', 'pa’rɛnta’puɲ')}


Concept ‘ear’ is different between Alorese and Lamaholot.

 One or more Lamaholot dialects innovated forms not found in Alorese:
 {('ear', 'lama1277-kalik', 'kiŋan'), ('ear', 'lama1277-minga', 'kiŋasa'), ('ear', 'lama1277-lewok', 'kiŋa'), ('ear', 'lama1277-imulo', 'kiŋasa'), ('ear', 'mamb1306', 'kikɑ'), ('ear', 'lama1277-lewuk', 'kiŋa'), ('ear', 'lama1277-lewot', 'kinaha'), ('ear', 'lama1277-paina', 'kiŋag'), ('ear', 'lama1277-lerek', 'kiˈŋa'), ('ear', 'lama1277-kalik', 'kiˈŋa'), ('ear', 'lama1277-lewop', 'kiŋa')}

  Just for clarity:
  There are also the following similarity classes shared between Alorese and LH.
  {('ear', 'p-alor1249', '*-uari'), ('ear', 'lama1277-lewoe', 'tilũ'), ('ear', 'alor1247-munas', 'tiluŋ'), ('ear', 'lama1277-tanju', 'tilu'), ('ear', 'tuku1254', "tə'ligi 'rɔa"), ('ear', 'p-mala1545-acd', '*taliŋa'), ('ear', 'laka1255', "tina 'rɔ:"), ('ear', 'lama1277-adona', 'ˈtiluˈkɜt'), ('ear', 'sika1262-maume', 't̪ilun'), ('ear', 'keda1252-leuba', 'til'), ('ear', 'lama1277-lamak', 'tilũ'), ('ear', 'lama1277-lewob', 'tilu'), ('ear', 'lama1277-ritae', 'tilũ'), ('ear', 'lama1277-dulhi', 'tilũ'), ('ear', 'lama1277-botun', 'tilũ'), ('ear', 'keda1252', 'til'), ('ear', 'lama1277-pukau', 'tilu'), ('ear', 'alor1247-baran', 'til:uŋ'), ('ear', 'p-mala1545-abvd', '*taliŋa'), ('ear', 'alor1247-pandai', 'tiluŋ'), ('ear', 'lama1277-ileap', 'tiluk'), ('ear', 'lama1277-lamat', 'tilu'), ('ear', 'keda1252-leuwa', 'til'), ('ear', 'lama1277-lamah', 'tilukə'), ('ear', 'sika1262-tanai', 'tilu-ŋ'), ('ear', 'p-cent2245-abvd', '*taliŋa'), ('ear', 'tetu1245-suai', 'tilun'), ('ear', 'p-aust1307-abvd', '*Caliŋa'), ('ear', 'lama1277-watan', 'tilũ'), ('ear', 'lama1277-wuake', 'tilu'), ('ear', 'alor1247-besar', 'tiluŋ'), ('ear', 'lama1277-lewog', 'tilu'), ('ear', 'lama1277-bama', 'tilu'), ('ear', 'lama1277-belan', 'tiluk'), ('ear', 'lama1277-mulan', 'tilu'), ('ear', 'lama1277-kiwan', 'tiluk'), ('ear', 'lama1277-merde', 'tiluk'), ('ear', 'tetu1246', 'tilun'), ('ear', 'sika1262-hewa', 'tilu'), ('ear', 'p-mala1545-acd', '*taŋila'), ('ear', 'lama1277-lewom', 'tilu'), ('ear', 'lama1277-lamal', 'tilu'), ('ear', 'tetu1245-vique', 'tilun'), ('ear', 'idat1237', "tɑ'lina"), ('ear', 'lama1277-lewoi', 'ˈtiluŋ'), ('ear', 'lama1277-waiba', 'tilu'), ('ear', 'lama1277-baipi', 'tilu'), ('ear', 'lama1277-waiwa', 'tilu'), ('ear', 'sika1262-tanai', 'tilu'), ('ear', 'lama1277-horow', 'tiluk'), ('ear', 'alor1247-munas', 'tiluʔ')}


Concept ‘skin’ is different between Alorese and Lamaholot.

 One or more Lamaholot dialects innovated forms not found in Alorese:
 {('skin', 'idat1237', "li'tɑk"), ('skin', 'lama1277-lewok', 'kuda'), ('skin', 'laka1255', 'litɑn'), ('skin', 'lama1277-kalik', 'kudan'), ('skin', 'lama1277-lewuk', 'kudan'), ('skin', 'lama1277-kalik', 'kuˈda')}

  Just for clarity:
  There are also the following similarity classes shared between Alorese and LH.
  {('skin', 'lama1277-lamal', 'kãmã'), ('skin', 'lama1277-lewom', 'kamaʔ'), ('skin', 'lama1277-ritae', 'kamaʔ'), ('skin', 'lama1277-tanju', 'kama'), ('skin', 'keda1252-leuwa', 'amaʔ'), ('skin', 'lama1277-paina', 'kamak'), ('skin', 'lama1277-bama', 'kama'), ('skin', 'lama1277-mulan', 'kɤmɤ'), ('skin', 'lama1277-minga', 'kamak'), ('skin', 'lama1277-lewot', 'kamakha'), ('skin', 'lama1277-merde', 'kama'), ('skin', 'lama1277-adona', 'ˈkamaˈkɜt'), ('skin', 'lama1277-wuake', 'kama'), ('skin', 'lama1277-dulhi', 'kamʔa'), ('skin', 'lama1277-lerek', 'kaˈmak'), ('skin', 'alor1247-pandai', 'kamak'), ('skin', 'lama1277-lewog', 'kama'), ('skin', 'lama1277-kiwan', 'kamʔa'), ('skin', 'lama1277-belan', 'kama'), ('skin', 'lama1277-botun', 'kamʔã'), ('skin', 'lama1277-ileap', 'kamak'), ('skin', 'keda1252-leuba', "ʔa'maʔ"), ('skin', 'keda1252', 'ama'), ('skin', 'lama1277-waiwa', 'kamʔa'), ('skin', 'lama1277-lamak', 'kamʔa'), ('skin', 'lama1277-lewop', 'kama'), ('skin', 'alor1247-baran', 'kamaŋ'), ('skin', 'lama1277-horow', 'kamakət'), ('skin', 'lama1277-waiba', 'kama'), ('skin', 'lama1277-imulo', 'kamakəs')}
  {('skin', 'tetu1246', 'kulit'), ('skin', 'lama1277-baipi', 'kulit'), ('skin', 'alor1247-pandai', 'kulik'), ('skin', 'tetu1245-vique', "isi'kulit"), ('skin', 'wers1238-marit', "k'lut"), ('skin', 'blag1240-nule', 'piʔulit'), ('skin', 'kira1248', 'kuli'), ('skin', 'lama1277-lewoi', 'ˈkuli'), ('skin', 'lama1277-lamah', 'kulit'), ('skin', 'p-mala1545-abvd', '*kulit'), ('skin', 'lama1277-lewob', 'kuli'), ('skin', 'lama1277-pukau', 'kulit'), ('skin', 'wers1238-taram', 'klut'), ('skin', 'sika1262-maume', 'ulit̪'), ('skin', 'buna1278-malia', 'gurul'), ('skin', 'sika1262-tanai', 'ʔuli-t'), ('skin', 'lama1277-watan', 'kulit'), ('skin', 'lama1277-lamat', 'ulitã'), ('skin', 'sika1262-hewa', 'ʔulit'), ('skin', 'kema1243', "uli'tir"), ('skin', 'sika1262-hewa', 'ulit'), ('skin', 'maka1316', 'uli'), ('skin', 'lama1277-lewoe', 'uli'), ('skin', 'tuku1254', "ku'luta"), ('skin', 'p-mala1545-acd', '*kulit'), ('skin', 'lamm1241-westp', 'kil:i'), ('skin', 'alor1247-munas', 'kulik'), ('skin', 'tetu1245-suai', "isi'kulit"), ('skin', 'alor1247-besar', "ku'likiŋ"), ('skin', 'mamb1306', 'litɑ'), ('skin', 'p-cent2245-abvd', '*kulit'), ('skin', 'baka1276', 'ulit'), ('skin', 'blag1240-kulij', 'piʔulit')}


Concept ‘one_hundred_and_twenty_three’ is different between Alorese and Lamaholot.

 Alorese innovated the following forms:
 {('one_hundred_and_twenty_three', 'alor1247-pandai', "ratu 'karua 'ilak 'talːɔ"), ('one_hundred_and_twenty_three', 'alor1247-besar', "ratu 'karua 'ilaka 'talːɔ"), ('one_hundred_and_twenty_three', 'alor1247-munas', "ratu 'karua 'ilak tə'lːɔ")}
 One or more Lamaholot dialects innovated forms not found in Alorese:
 {('one_hundred_and_twenty_three', 'lama1277-lewoi', 'ˈtəratu pulu ˈrua noʔoŋ ˈtɛlo'), ('one_hundred_and_twenty_three', 'sika1262-hewa', 'ŋasu ha pulu rua ʋot təlu'), ('one_hundred_and_twenty_three', 'lama1277-kalik', 'ˈratu tu no pul ˈʤua no təˈlu'), ('one_hundred_and_twenty_three', 'lama1277-lerek', 'rat tu no pul ˈdʒ͡ua no təl'), ('one_hundred_and_twenty_three', 'tetu1245-suai', 'atus ida rua nulu resin tolu'), ('one_hundred_and_twenty_three', 'tetu1245-vique', 'atus ida rua nulu resin tolu'), ('one_hundred_and_twenty_three', 'lama1277-adona', 'tɜˈratu pulu ˈrua nɔ ˈtɛlo')}


Concept ‘rat’ is different between Alorese and Lamaholot.

 One or more Lamaholot dialects innovated forms not found in Alorese:
 {('rat', 'lama1277-lerek', 'məˈlak')}

  Just for clarity:
  There are also the following similarity classes shared between Alorese and LH.
  {('rat', 'alor1247-pandai', 'mɔre'), ('rat', 'lama1277-kalik', 'kroˈmɛr'), ('rat', 'alor1247-baran', "ka'more"), ('rat', 'alor1247-munas', 'more'), ('rat', 'lama1277-lewoi', 'kəˈrɔme'), ('rat', 'lama1277-adona', 'ˈkromɛ'), ('rat', 'lama1277-kalik', 'kroˈmi'), ('rat', 'alor1247-pandai', "k'mɔre"), ('rat', 'alor1247-besar', "ka'more")}


Concept ‘to_sell’ is different between Alorese and Lamaholot.

 Alorese innovated the following forms:
 {('to_sell', 'alor1247-pandai', "p'nahaŋ"), ('to_sell', 'alor1247-besar', "pa'nahaŋ"), ('to_sell', 'alor1247-munas', "p'nahaŋ")}
 One or more Lamaholot dialects innovated forms not found in Alorese:
 {('to_sell', 'lama1277-adona', 'ˈduʔũ'), ('to_sell', 'lama1277-lewoi', 'ˈduʔuŋ'), ('to_sell', 'kelo1247-hopte', 'ta:n'), ('to_sell', 'lama1277-lerek', 'duˈrum'), ('to_sell', 'lamm1241-westp', 'ariŋ'), ('to_sell', 'lama1277-kalik', 'duˈrum'), ('to_sell', 'keda1252-leuba', "du'ruŋ")}


Concept ‘needle’ is different between Alorese and Lamaholot.

 Alorese shares the following forms with other AN languanges, but not Lamaholot:
 {('needle', 'nede1245', 'batu'), ('needle', 'teiw1235', "ba'ti"), ('needle', 'blag1240-bama', 'batul'), ('needle', 'sarr1247-adiab', "bi'tal"), ('needle', 'baka1276', 'batul'), ('needle', 'alor1247-munas', 'batul'), ('needle', 'alor1247-besar', 'batu'), ('needle', 'blag1240-kulij', 'batul'), ('needle', 'blag1240-nule', 'batul'), ('needle', 'alor1247-pandai', 'batul'), ('needle', 'adan1251-otvai', '’batiŋ'), ('needle', 'pura1258', 'batul'), ('needle', 'blag1240-tuntu', "ba'tul"), ('needle', 'sarr1247-nule', 'bitaj'), ('needle', 'kabo1247', 'bataŋ'), ('needle', 'keda1252-leuba', "ba'tur"), ('needle', 'adan1251-lawah', 'bátuŋ'), ('needle', 'blag1240-warsa', 'batul'), ('needle', 'rett1240', 'batul'), ('needle', 'kaer1234', 'ba:ti')}
 One or more Lamaholot dialects innovated forms not found in Alorese:
 {('needle', 'sika1262-hewa', 'luhir'), ('needle', 'lama1277-kalik', 'ˈlusir'), ('needle', 'lama1277-lewoi', 'ˈluhi'), ('needle', 'lama1277-lerek', 'ˈluhir'), ('needle', 'lama1277-adona', 'ˈluhi')}


Concept ‘twenty_five’ is different between Alorese and Lamaholot.

 Alorese innovated the following forms:
 {('twenty_five', 'alor1247-munas', "karua 'ilak 'lɛma"), ('twenty_five', 'alor1247-pandai', "karua 'ilak 'lɛma"), ('twenty_five', 'alor1247-besar', "karua 'ilaka 'lɛma")}
 One or more Lamaholot dialects innovated forms not found in Alorese:
 {('twenty_five', 'tetu1245-suai', 'rua nulu resin lima'), ('twenty_five', 'maka1316', "ru lo'laɁe resi 'lima"), ('twenty_five', 'lama1277-lerek', 'pul dʒ͡ə no ˈlɛma'), ('twenty_five', 'sika1262-hewa', 'pulu rua ʋot lima'), ('twenty_five', 'lama1277-lewoi', 'pulu rua noʔoŋ ˈlema'), ('twenty_five', 'lama1277-adona', 'pulu rua nɔ ˈlɛma'), ('twenty_five', 'lama1277-kalik', 'ˈpulu ˈʤua no lɛm'), ('twenty_five', 'tetu1245-vique', 'rua nulu resin lima')}


Concept ‘eleven’ is different between Alorese and Lamaholot.

 Alorese innovated the following forms:
 {('eleven', 'alor1247-munas', "kər'tou 'ilak 'tou"), ('eleven', 'alor1247-pandai', "kə'rtou 'ilak 'tou"), ('eleven', 'alor1247-besar', "kartou 'ilaka 'tou")}
 One or more Lamaholot dialects innovated forms not found in Alorese:
 {('eleven', 'sika1262-hewa', 'pulu ʋot ha'), ('eleven', 'lama1277-lerek', 'heˈpulo no tu'), ('eleven', 'lama1277-lewoi', 'ˈpulo noʔoŋ ˈtoʔu'), ('eleven', 'keda1252-leuba', "pulaʔ u'deʔ"), ('eleven', 'lama1277-kalik', 'ˈspulo no tu'), ('eleven', 'lama1277-adona', 'pulok ˈtoʔu')}


Concept ‘bone’ is different between Alorese and Lamaholot.

 Alorese shares the following forms with other AN languanges, but not Lamaholot:
 {('bone', 'tetu1245-suai', 'ruin'), ('bone', 'alor1247-munas', 'ruiŋ'), ('bone', 'alor1247-besar', 'ruĩ'), ('bone', 'alor1247-munas', 'rue'), ('bone', 'tetu1245-vique', 'ruin'), ('bone', 'mamb1306', 'rui'), ('bone', 'tuku1254', 'rui'), ('bone', 'alor1247-pandai', 'ruiŋ'), ('bone', 'alor1247-baran', "ru'ʔiŋ"), ('bone', 'laka1255', 'ruin'), ('bone', 'kema1243', "ru'ir"), ('bone', 'tetu1246', 'ruin')}
 One or more Lamaholot dialects innovated forms not found in Alorese:
 {('bone', 'lama1277-horow', 'riʔuk'), ('bone', 'lama1277-lewok', 'riuk'), ('bone', 'lama1277-waiba', 'riʔuk'), ('bone', 'lama1277-lewoe', 'riʔũ'), ('bone', 'lama1277-paina', 'riuk'), ('bone', 'lama1277-kiwan', 'riʔũ'), ('bone', 'lama1277-lamah', 'riɁu'), ('bone', 'lama1277-belan', 'riuk'), ('bone', 'lama1277-kalik', 'riˈuk'), ('bone', 'lama1277-minga', 'riuk'), ('bone', 'lama1277-botun', 'riʔũ'), ('bone', 'lama1277-lamat', 'riʔũ'), ('bone', 'lama1277-merde', 'riuk'), ('bone', 'lama1277-tanju', 'riʔuk'), ('bone', 'lama1277-pukau', 'riʔuk'), ('bone', 'lama1277-lewob', 'riʔu'), ('bone', 'lama1277-lamal', 'riuk'), ('bone', 'lama1277-lewot', 'riuk'), ('bone', 'lama1277-imulo', 'riukəs'), ('bone', 'lama1277-bama', 'riʔu'), ('bone', 'lama1277-dulhi', 'riʔũ'), ('bone', 'lama1277-baipi', 'riʔuk'), ('bone', 'lama1277-lewoi', 'ˈriʔu'), ('bone', 'lama1277-watan', 'riʔũ'), ('bone', 'lama1277-lewog', 'riʔu'), ('bone', 'lama1277-lewuk', 'riuksa'), ('bone', 'lama1277-lamak', 'rĩu'), ('bone', 'lama1277-lerek', 'ˈriuk'), ('bone', 'lama1277-mulan', 'riuk'), ('bone', 'lama1277-adona', 'ˈriʔũː'), ('bone', 'lama1277-lewop', 'riuk'), ('bone', 'lama1277-lewom', 'riʔuk'), ('bone', 'lama1277-ritae', 'riʔuk'), ('bone', 'lama1277-wuake', 'riɁũ'), ('bone', 'lama1277-waiwa', 'riuk'), ('bone', 'lama1277-kalik', 'riuk'), ('bone', 'lama1277-ileap', 'riuk')}


Concept ‘axe’ is different between Alorese and Lamaholot.

 One or more Lamaholot dialects innovated forms not found in Alorese:
 {('axe', 'sika1262-hewa', 'kapak'), ('axe', 'lama1277-kalik', 'ˈkapak'), ('axe', 'lama1277-adona', "soru 'kɑpa"), ('axe', 'lama1277-kalik', 'ˈkapa'), ('axe', 'lama1277-lewoi', 'ˈsɔru')}

  Just for clarity:
  There are also the following similarity classes shared between Alorese and LH.
  {('axe', 'keda1252-leuba', "ba'liʔ"), ('axe', 'atim1239', 'faliŋ'), ('axe', 'lama1277-lerek', 'ˈbadoŋ'), ('axe', 'kama1365', 'paliŋ'), ('axe', 'abui1241-takal', "fä.'liŋ"), ('axe', 'alor1247-besar', 'bali'), ('axe', 'tetu1245-vique', 'baliu'), ('axe', 'wers1238-taram', 'poloŋ'), ('axe', 'alor1247-pandai', 'bali'), ('axe', 'dein1238', 'baliŋ'), ('axe', 'adan1251-otvai', '’baliŋ'), ('axe', 'kabo1247', 'baliŋ boʔoboj'), ('axe', 'alor1247-baran', 'bali'), ('axe', 'adan1251-lawah', 'baliŋboʔoboi'), ('axe', 'kafo1240', 'fɑlɪŋ'), ('axe', 'sarr1247-nule', 'baliŋ'), ('axe', 'tetu1245-suai', 'baliu'), ('axe', 'adan1251-lawah', 'baliŋ'), ('axe', 'alor1247-munas', 'bali'), ('axe', 'abui1241-petle', "fa.'liŋ"), ('axe', 'abui1241-ulaga', 'valiŋ'), ('axe', 'abui1241-fuime', 'faliŋ'), ('axe', 'sarr1247-adiab', "ba'liŋ"), ('axe', 'abui1241-takal', "fä.'lĩ"), ('axe', 'wers1238-marit', 'pɔlɔŋ'), ('axe', 'lamm1241-westp', 'baliŋ')}


Concept ‘priest_modern’ is different between Alorese and Lamaholot.

 Alorese innovated the following forms:
 {('priest_modern', 'alor1247-besar', 'hatib')}
 {('priest_modern', 'alor1247-munas', "guru 'indʒil")}
 Alorese shares the following forms with other AN languanges, but not Lamaholot:
 {('priest_modern', 'koto1251', 'panri:t'), ('priest_modern', 'kula1280-lanto', '’panit'), ('priest_modern', 'sawi1256', "pa'niti"), ('priest_modern', 'kaer1234', "pen'deta"), ('priest_modern', 'teiw1235', "pan'deta"), ('priest_modern', 'kelo1247-hopte', "pan'dita"), ('priest_modern', 'alor1247-munas', "pan'ditaʔ"), ('priest_modern', 'pura1258', "pa'nita"), ('priest_modern', 'wers1238-marit', "pan'dit")}
 One or more Lamaholot dialects innovated forms not found in Alorese:
 {('priest_modern', 'lama1277-lerek', 'ˈata ˈbudʒ͡ak')}

  Just for clarity:
  There are also the following similarity classes shared between Alorese and LH.
  {('priest_modern', 'sika1262-hewa', 'tuan'), ('priest_modern', 'lama1277-adona', 'ˈtuã'), ('priest_modern', 'abui1241-ulaga', 'tuoŋ'), ('priest_modern', 'lama1277-lerek', 'ˈtuan'), ('priest_modern', 'lama1277-kalik', 'tuˈan'), ('priest_modern', 'abui1241-takal', "tu.'ɔŋ"), ('priest_modern', 'lama1277-lewoi', 'ˈtuaŋ'), ('priest_modern', 'alor1247-besar', 'ʤou')}


Concept ‘clan’ is different between Alorese and Lamaholot.

 Alorese shares the following forms with TAP languages:
 {('clan', 'pura1258', 'lɛlaŋ'), ('clan', 'alor1247-pandai', 'lalːaŋ'), ('clan', 'kaer1234', "le'liŋ"), ('clan', 'lamm1241-westp', 'leŋ'), ('clan', 'alor1247-besar', 'lalaŋ'), ('clan', 'rett1240', 'lɛlaŋ'), ('clan', 'alor1247-munas', 'lɛlaŋ'), ('clan', 'adan1251-otvai', '’lɛlaŋ')}
 One or more Lamaholot dialects innovated forms not found in Alorese:
 {('clan', 'lama1277-lerek', 'huk ˈlamak')}
 {('clan', 'tetu1245-suai', 'suku'), ('clan', 'lama1277-lewoi', 'ˈsuku'), ('clan', 'lama1277-adona', 'ˈsuku'), ('clan', 'tetu1245-vique', 'suku'), ('clan', 'teiw1235', 'suku'), ('clan', 'sika1262-hewa', 'suku'), ('clan', 'maka1316', 'nuku'), ('clan', 'keda1252-leuba', 'suku')}


Concept ‘to_steal’ is different between Alorese and Lamaholot.

 One or more Lamaholot dialects innovated forms not found in Alorese:
 {('to_steal', 'maka1316', 'lia'), ('to_steal', 'lama1277-adona', 'ˈlãː')}
 {('to_steal', 'lama1277-kalik', 'laˈvit')}

  Just for clarity:
  There are also the following similarity classes shared between Alorese and LH.
  {('to_steal', 'blag1240-tuntu', 'taqawi'), ('to_steal', 'alor1247-pandai', "t'maka"), ('to_steal', 'adan1251-lawah', 'taʔau'), ('to_steal', 'sawi1256', "ta'ko"), ('to_steal', 'abui1241-ulaga', 'takau'), ('to_steal', 'blag1240-kulij', 'tavi'), ('to_steal', 'blag1240-bama', 'taxawi'), ('to_steal', 'lama1277-kalik', 'taˈkav'), ('to_steal', 'wers1238-taram', 'tekau'), ('to_steal', 'p-aust1307-abvd', '*Cakaw'), ('to_steal', 'p-mala1545-acd', '*nakaw'), ('to_steal', 'kama1365', 'takaːwa'), ('to_steal', 'kelo1247-hopte', "tə'ka:"), ('to_steal', 'sika1262-hewa', 'toʔi'), ('to_steal', 'teiw1235', "ta'xa:u"), ('to_steal', 'sarr1247-adiab', 'taxa'), ('to_steal', 'p-cent2245-abvd', '*takaw'), ('to_steal', 'kula1280-lanto', 'tako'), ('to_steal', 'abui1241-petle', 'ta.kau'), ('to_steal', 'kaer1234', "ta'qawo"), ('to_steal', 'wers1238-marit', "tə'kau"), ('to_steal', 'lama1277-lerek', 'taˈkav'), ('to_steal', 'adan1251-otvai', '’taʔu'), ('to_steal', 'blag1240-warsa', 'taxavi'), ('to_steal', 'abui1241-fuime', 'takau'), ('to_steal', 'kabo1247', 'nataʔaw'), ('to_steal', 'nede1245', 'taxawa'), ('to_steal', 'p-mala1545-acd', '*takaw'), ('to_steal', 'pura1258', 'taɁavi'), ('to_steal', 'sarr1247-adiab', 'taxawis'), ('to_steal', 'kuii1253', 'takao'), ('to_steal', 'blag1240-kulij', 'tawi'), ('to_steal', 'atim1239', 'takau'), ('to_steal', 'pura1258', 'taɁabi'), ('to_steal', 'sarr1247-nule', 'taxa:'), ('to_steal', 'baka1276', 'tavi'), ('to_steal', 'kira1248', 'takau'), ('to_steal', 'blag1240-nule', 'tavi'), ('to_steal', 'abui1241-takal', "tä.'käi"), ('to_steal', 'p-mala1545-abvd', '*takaw'), ('to_steal', 'kula1280-lanto', '’ta:ko'), ('to_steal', 'dein1238', 'taxau')}
  {('to_steal', 'alor1247-pandai', 'maka'), ('to_steal', 'lama1277-lewoi', 'ˈtəmaka'), ('to_steal', 'alor1247-munas', 'maka'), ('to_steal', 'alor1247-besar', "ta'maku")}


Concept ‘twenty_two’ is different between Alorese and Lamaholot.

 Alorese innovated the following forms:
 {('twenty_two', 'alor1247-munas', "karua 'ilak 'rua"), ('twenty_two', 'alor1247-besar', "karua 'ilaka 'rua"), ('twenty_two', 'alor1247-pandai', "karua 'ilak 'rua")}
 One or more Lamaholot dialects innovated forms not found in Alorese:
 {('twenty_two', 'lama1277-lewoi', 'pulu rua  noʔoŋ ˈrua'), ('twenty_two', 'lama1277-adona', 'pulu rua nɔ ˈrua'), ('twenty_two', 'sika1262-hewa', 'pulu rua ʋot rua'), ('twenty_two', 'tetu1245-vique', 'rua nulu resin rua'), ('twenty_two', 'tetu1245-suai', 'rua nulu resin rua'), ('twenty_two', 'lama1277-kalik', 'ˈpulu ˈʤua no ˈʤua'), ('twenty_two', 'lama1277-lerek', 'pul dʒ͡ə no ˈdʒua')}


Concept ‘to_swim’ is different between Alorese and Lamaholot.

 One or more Lamaholot dialects innovated forms not found in Alorese:
 {('to_swim', 'sarr1247-adiab', "tə'lis"), ('to_swim', 'lama1277-kalik', 'ˈdulɔ'), ('to_swim', 'sarr1247-nule', 'təlis'), ('to_swim', 'atim1239', 'na-teula'), ('to_swim', 'atim1239', 'teula'), ('to_swim', 'lama1277-lewuk', 'dulo'), ('to_swim', 'dein1238', 'talis')}
 {('to_swim', 'lama1277-waiba', 'doŋat')}

  Just for clarity:
  There are also the following similarity classes shared between Alorese and LH.
  {('to_swim', 'lama1277-belan', 'naŋe'), ('to_swim', 'lama1277-ileap', 'naŋe'), ('to_swim', 'kema1243', 'nagi'), ('to_swim', 'buna1278-malia', 'nɑni'), ('to_swim', 'lama1277-pukau', 'naŋe'), ('to_swim', 'lama1277-adona', 'ˈnaŋe'), ('to_swim', 'keda1252-leuba', 'naŋi'), ('to_swim', 'lama1277-tanju', 'naŋe'), ('to_swim', 'alor1247-besar', 'naŋgɛ'), ('to_swim', 'lama1277-watan', 'naŋe'), ('to_swim', 'alor1247-pandai', 'naŋge'), ('to_swim', 'p-mala1545-abvd', '*naŋuy'), ('to_swim', 'p-aust1307-abvd', '*Naŋuy'), ('to_swim', 'lama1277-lewoe', 'naŋi'), ('to_swim', 'lama1277-imulo', 'naŋ'), ('to_swim', 'lama1277-merde', 'naŋe'), ('to_swim', 'sika1262-maume', 'nani'), ('to_swim', 'sika1262-hewa', 'nani'), ('to_swim', 'lama1277-paina', 'naŋ'), ('to_swim', 'lama1277-wuake', 'naŋe'), ('to_swim', 'lama1277-botun', 'naŋe'), ('to_swim', 'keda1252', 'naŋe'), ('to_swim', 'sika1262-tanai', 'nani'), ('to_swim', 'lama1277-lamah', 'naŋe'), ('to_swim', 'lama1277-lerek', 'naŋ'), ('to_swim', 'lama1277-lewoi', 'ˈnaŋe'), ('to_swim', 'p-mala1545-abvd', '*laŋuy'), ('to_swim', 'lama1277-lewop', 'naŋi'), ('to_swim', 'lama1277-minga', 'naŋe'), ('to_swim', 'tetu1245-suai', 'nani'), ('to_swim', 'tetu1245-vique', 'nani'), ('to_swim', 'lama1277-mulan', 'naŋe'), ('to_swim', 'lama1277-bama', 'naŋe'), ('to_swim', 'lama1277-kalik', 'ˈnaŋe'), ('to_swim', 'lama1277-dulhi', 'naŋe'), ('to_swim', 'lama1277-kiwan', 'naŋe'), ('to_swim', 'laka1255', "na'ni"), ('to_swim', 'p-mala1545-acd', '*naŋuy'), ('to_swim', 'lama1277-lamak', 'naŋe'), ('to_swim', 'keda1252-leuwa', 'naŋi ɔ̘jaŋ'), ('to_swim', 'lama1277-lamat', 'naŋi'), ('to_swim', 'buna1278-suai', 'nagi'), ('to_swim', 'lama1277-lewog', 'naŋe'), ('to_swim', 'mamb1306', 'na:n'), ('to_swim', 'buna1278-bobon', 'nagi'), ('to_swim', 'tetu1246', 'nani'), ('to_swim', 'lama1277-kalik', 'naŋe'), ('to_swim', 'lama1277-lewob', 'naŋe'), ('to_swim', 'tuku1254', 'nagi'), ('to_swim', 'alor1247-baran', 'naŋgɛ'), ('to_swim', 'lama1277-lamal', 'naŋe'), ('to_swim', 'lama1277-lewok', 'naŋe'), ('to_swim', 'lama1277-lewom', 'naŋe'), ('to_swim', 'alor1247-munas', 'naŋgɛ'), ('to_swim', 'lama1277-ritae', 'naŋe'), ('to_swim', 'lama1277-baipi', 'naŋe'), ('to_swim', 'lama1277-lewot', 'naŋi'), ('to_swim', 'p-cent2245-abvd', '*naŋuy'), ('to_swim', 'lama1277-horow', 'naŋe'), ('to_swim', 'lama1277-waiwa', 'naŋe')}


Concept ‘twenty_one’ is different between Alorese and Lamaholot.

 Alorese innovated the following forms:
 {('twenty_one', 'alor1247-pandai', "karua 'ilak 'tou"), ('twenty_one', 'alor1247-munas', "karua 'ilak 'tou"), ('twenty_one', 'alor1247-besar', "karua 'ilaka 'tou")}
 One or more Lamaholot dialects innovated forms not found in Alorese:
 {('twenty_one', 'lama1277-kalik', 'ˈpulu ˈʤua no tu'), ('twenty_one', 'lama1277-lerek', 'pul dʒ͡ə no tu'), ('twenty_one', 'lama1277-lewoi', 'pulu rua  noʔoŋ ˈtoʔu'), ('twenty_one', 'sika1262-hewa', 'pulu rua ʋot ha'), ('twenty_one', 'lama1277-adona', 'pulu rua nɔ ˈtoʔu')}


Concept ‘to_work’ is different between Alorese and Lamaholot.

 One or more Lamaholot dialects innovated forms not found in Alorese:
 {('to_work', 'lama1277-waiwa', 'ola'), ('to_work', 'lama1277-horow', 'ola'), ('to_work', 'lama1277-dulhi', 'ola'), ('to_work', 'lama1277-ritae', 'ola'), ('to_work', 'lama1277-botun', 'ola'), ('to_work', 'lama1277-adona', 'ˈʔola')}
 {('to_work', 'lama1277-lewoe', 'ewo eʔo')}

  Just for clarity:
  There are also the following similarity classes shared between Alorese and LH.
  {('to_work', 'lama1277-lamal', 'kariã'), ('to_work', 'alor1247-besar', "kə'raʤaŋ"), ('to_work', 'lama1277-lamah', 'kərja'), ('to_work', 'lama1277-paina', 'kərəja'), ('to_work', 'pura1258', "ka'riɟaŋ"), ('to_work', 'keda1252', 'kareyã'), ('to_work', 'abui1241-ulaga', 'kariaŋ'), ('to_work', 'lama1277-pukau', 'kəriã'), ('to_work', 'lama1277-belan', 'kərja'), ('to_work', 'lama1277-waiba', 'kəria'), ('to_work', 'lama1277-bama', 'kariã'), ('to_work', 'alor1247-baran', "ka'raʤaŋ"), ('to_work', 'lama1277-lewog', 'kəriã'), ('to_work', 'lama1277-lewop', 'kərian'), ('to_work', 'keda1252-leuba', "ka'rejaŋ"), ('to_work', 'lama1277-baipi', 'gəriã'), ('to_work', 'lama1277-watan', 'kərja'), ('to_work', 'sika1262-hewa', 'kərian'), ('to_work', 'lama1277-lewob', 'kəriã'), ('to_work', 'lama1277-lewuk', 'kəria'), ('to_work', 'lama1277-minga', 'kəria'), ('to_work', 'kelo1247-hopte', "kre'jaŋ"), ('to_work', 'lama1277-lamak', 'kərja'), ('to_work', 'lama1277-mulan', 'kəriɤ'), ('to_work', 'lama1277-kiwan', 'kərəya'), ('to_work', 'lama1277-adona', 'krɜja'), ('to_work', 'alor1247-pandai', "k'radʒaŋ"), ('to_work', 'lamm1241-westp', 'kariaŋ'), ('to_work', 'teiw1235', 'krian'), ('to_work', 'lama1277-lerek', 'kəˈradʒ͡a'), ('to_work', 'adan1251-otvai', 'kara’saŋ'), ('to_work', 'lama1277-ileap', 'kərja'), ('to_work', 'lama1277-merde', 'kərja'), ('to_work', 'lama1277-lewoi', 'kəˈriaŋ'), ('to_work', 'lama1277-wuake', 'kərja'), ('to_work', 'alor1247-munas', "kr'dʒaŋ"), ('to_work', 'sika1262-hewa', 'krian'), ('to_work', 'lama1277-imulo', 'kəria'), ('to_work', 'teiw1235', 'krajan'), ('to_work', 'lama1277-lewom', 'gəriã'), ('to_work', 'lama1277-lewot', 'kəria'), ('to_work', 'kaer1234', 'kiriaŋ'), ('to_work', 'lama1277-tanju', 'kəriã'), ('to_work', 'lama1277-kalik', 'kərˈʤan'), ('to_work', 'lama1277-kalik', 'kərja'), ('to_work', 'lama1277-lewok', 'kərja'), ('to_work', 'lama1277-lamat', 'kəria'), ('to_work', 'abui1241-takal', 'kär.je̞äŋ')}


Concept ‘stone’ is different between Alorese and Lamaholot.

 One or more Lamaholot dialects innovated forms not found in Alorese:
 {('stone', 'lama1277-lewot', 'enaj')}

  Just for clarity:
  There are also the following similarity classes shared between Alorese and LH.
  {('stone', 'p-mala1545-abvd', '*batu'), ('stone', 'laka1255', 'hatuk'), ('stone', 'lama1277-pukau', 'wato'), ('stone', 'lama1277-minga', 'wato'), ('stone', 'lama1277-lewuk', 'wator'), ('stone', 'lama1277-kalik', 'ˈvatu'), ('stone', 'sika1262-hewa', 'ʋatu'), ('stone', 'lama1277-belan', 'wato'), ('stone', 'keda1252', 'waʔ'), ('stone', 'lama1277-lamak', 'wato'), ('stone', 'lama1277-kalik', 'vaˈtɔr'), ('stone', 'lama1277-lamal', 'fato'), ('stone', 'alor1247-pandai', 'watɔ'), ('stone', 'lama1277-lewog', 'wato'), ('stone', 'lama1277-waiwa', 'wato'), ('stone', 'lama1277-lerek', 'ˈwator'), ('stone', 'lama1277-lewop', 'fatoru'), ('stone', 'lama1277-lamah', 'wato'), ('stone', 'lama1277-lewom', 'wato'), ('stone', 'lama1277-ritae', 'wato'), ('stone', 'idat1237', 'hatu'), ('stone', 'lama1277-wuake', 'wato'), ('stone', 'lama1277-imulo', 'fator'), ('stone', 'lama1277-mulan', 'wato'), ('stone', 'lama1277-horow', 'wato'), ('stone', 'alor1247-baran', 'fato'), ('stone', 'tuku1254', 'hatu'), ('stone', 'lama1277-baipi', 'wato'), ('stone', 'koto1251', 'fatu'), ('stone', 'mamb1306', 'hat'), ('stone', 'lama1277-lewoi', 'ˈwatɔ'), ('stone', 'lama1277-watan', 'wato'), ('stone', 'lama1277-dulhi', 'wato'), ('stone', 'tetu1245-suai', 'fatuk'), ('stone', 'p-mala1545-acd', '*batu'), ('stone', 'lama1277-bama', 'wato'), ('stone', 'sika1262-hewa', 'watu'), ('stone', 'lama1277-lewob', 'wato'), ('stone', 'lama1277-kalik', 'watu'), ('stone', 'lama1277-lewok', 'watoru'), ('stone', 'kema1243', 'hatu'), ('stone', 'lama1277-botun', 'wato'), ('stone', 'lama1277-merde', 'wato'), ('stone', 'lama1277-kiwan', 'wato'), ('stone', 'lama1277-tanju', 'wato'), ('stone', 'lama1277-paina', 'wator'), ('stone', 'alor1247-munas', 'watɔ'), ('stone', 'alor1247-besar', 'fatɔ'), ('stone', 'p-cent2245-abvd', '*batu'), ('stone', 'p-aust1307-abvd', '*batu'), ('stone', 'tetu1245-vique', 'fatuk'), ('stone', 'fata1247', 'matar'), ('stone', 'tetu1246', "fa'tuk"), ('stone', 'lama1277-waiba', 'wato'), ('stone', 'lama1277-lewoe', 'wato'), ('stone', 'keda1252-leuba', 'waʔ'), ('stone', 'lama1277-ileap', 'wato'), ('stone', 'lama1277-lamat', 'wato'), ('stone', 'sika1262-tanai', 'βatu'), ('stone', 'sika1262-maume', 'vat̪u'), ('stone', 'lama1277-adona', 'watɔ')}


Concept ‘twenty_three’ is different between Alorese and Lamaholot.

 Alorese innovated the following forms:
 {('twenty_three', 'alor1247-besar', "karua 'ilaka 'talːɔ"), ('twenty_three', 'alor1247-munas', "karua 'ilak tə'lːɔ"), ('twenty_three', 'alor1247-pandai', "karua 'ilak 'talːɔ")}
 One or more Lamaholot dialects innovated forms not found in Alorese:
 {('twenty_three', 'lama1277-lerek', 'pul dʒ͡ə no təl'), ('twenty_three', 'keda1252-leuba', 'purun suwe ilaʔ tɛlu'), ('twenty_three', 'lama1277-lewoi', 'pulu rua noʔoŋ ˈtɛlːo'), ('twenty_three', 'lama1277-adona', 'pulu rua nɔ ˈtɛlo'), ('twenty_three', 'tetu1245-suai', 'rua nulu resin tolu'), ('twenty_three', 'tetu1245-vique', 'rua nulu resin tolu'), ('twenty_three', 'sika1262-hewa', 'pulu rua ʋot təlu'), ('twenty_three', 'lama1277-kalik', 'ˈpulu ˈʤua no təˈlu')}


Concept ‘3sg’ is different between Alorese and Lamaholot.

 Alorese innovated the following forms:
 {('3sg', 'alor1247-munas', 'ro'), ('3sg', 'alor1247-pandai', 'ro')}
 One or more Lamaholot dialects innovated forms not found in Alorese:
 {('3sg', 'lama1277-lerek', '(mɔn)'), ('3sg', 'lama1277-lerek', '(mɔˈne)')}

  Just for clarity:
  There are also the following similarity classes shared between Alorese and LH.
  {('3sg', 'tetu1245-vique', 'nia'), ('3sg', 'lama1277-paina', 'nane'), ('3sg', 'lama1277-minga', 'na'), ('3sg', 'lama1277-kalik', 'na'), ('3sg', 'lama1277-kalik', 'nane'), ('3sg', 'maka1316', 'laɁi'), ('3sg', 'lama1277-lewog', 'na'), ('3sg', 'lama1277-lewom', 'naʔe'), ('3sg', 'lama1277-lamak', 'naʔe'), ('3sg', 'lama1277-ritae', 'naʔe'), ('3sg', 'lama1277-lamal', 'nae'), ('3sg', 'sika1262-tanai', 'nimu'), ('3sg', 'lama1277-mulan', 'nae'), ('3sg', 'tetu1245-suai', 'nia'), ('3sg', 'lama1277-horow', 'naʔe'), ('3sg', 'keda1252-leuba', 'nuɔ'), ('3sg', 'lama1277-lewop', 'na'), ('3sg', 'lama1277-baipi', 'naʔe'), ('3sg', 'sika1262-maume', 'miu'), ('3sg', 'lama1277-lewoi', 'naː'), ('3sg', 'laka1255', 'ni mane'), ('3sg', 'idat1237', 'ni:'), ('3sg', 'lama1277-imulo', 'na'), ('3sg', 'sika1262-hewa', 'nimu'), ('3sg', 'lama1277-lamat', 'noʔe'), ('3sg', 'lama1277-dulhi', 'na(e)'), ('3sg', 'lama1277-lewoe', 'noʔe'), ('3sg', 'lama1277-lewot', 'na'), ('3sg', 'lama1277-pukau', 'na'), ('3sg', 'lama1277-belan', 'nae'), ('3sg', 'lama1277-watan', 'naʔe'), ('3sg', 'lama1277-wuake', 'naɁe'), ('3sg', 'lama1277-bama', 'na'), ('3sg', 'lama1277-lewob', 'na'), ('3sg', 'lama1277-waiwa', 'naʔe'), ('3sg', 'tetu1246', 'nia'), ('3sg', 'lama1277-lewok', 'nane'), ('3sg', 'lama1277-merde', 'naʔe'), ('3sg', 'keda1252-leuwa', 'ni'), ('3sg', 'alor1247-baran', 'nɔ:'), ('3sg', 'lama1277-kiwan', 'naʔe'), ('3sg', 'alor1247-besar', 'no'), ('3sg', 'lama1277-tanju', 'naʔe'), ('3sg', 'lama1277-botun', 'naʔe'), ('3sg', 'keda1252-leuwa', 'nuɔ'), ('3sg', 'lama1277-lamah', 'nae'), ('3sg', 'keda1252', 'nuo'), ('3sg', 'lama1277-waiba', 'naʔe'), ('3sg', 'lama1277-adona', 'ˈnaʔɛ'), ('3sg', 'laka1255', 'ni:'), ('3sg', 'lama1277-ileap', 'na'), ('3sg', 'alor1247-munas', 'no')}


Concept ‘to_pound’ is different between Alorese and Lamaholot.

 One or more Lamaholot dialects innovated forms not found in Alorese:
 {('to_pound', 'blag1240-kulij', 'tapaʔ'), ('to_pound', 'rett1240', 'ta'), ('to_pound', 'kula1280-lanto', 'atuba'), ('to_pound', 'kaer1234', "ta'po"), ('to_pound', 'lama1277-adona', 'ˈtubuk'), ('to_pound', 'kama1365', 'tafe'), ('to_pound', 'kelo1247-hopte', "tə'pan"), ('to_pound', 'p-east2519', '*tafa'), ('to_pound', 'kabo1247', 'natapaŋ'), ('to_pound', 'sarr1247-nule', 'ta:p'), ('to_pound', 'maka1316', 'tafa'), ('to_pound', 'atim1239', 'tape'), ('to_pound', 'sarr1247-adiab', 'tap'), ('to_pound', 'blag1240-nule', 'tapa'), ('to_pound', 'p-timo1261', '*tapa(i)'), ('to_pound', 'abui1241-fuime', 'tape'), ('to_pound', 'abui1241-takal', "tä.'pe̞i"), ('to_pound', 'fata1247', 'tafa'), ('to_pound', 'kula1280-lanto', '’taŋ'), ('to_pound', 'adan1251-otvai', 'ta’paŋ'), ('to_pound', 'kira1248', 'tapai'), ('to_pound', 'lamm1241-westp', 'tap:aŋ'), ('to_pound', 'dein1238', 'utap'), ('to_pound', 'p-alor1249', '*tapai'), ('to_pound', 'teiw1235', 'tap'), ('to_pound', 'sawi1256', 'tana'), ('to_pound', 'pura1258', "mod 'tapa"), ('to_pound', 'p-mala1545-acd', '*bukbuk'), ('to_pound', 'adan1251-lawah', 'ta:paŋ'), ('to_pound', 'wers1238-marit', 'ta:'), ('to_pound', 'rett1240', 'ta:')}

  Just for clarity:
  There are also the following similarity classes shared between Alorese and LH.
  {('to_pound', 'p-mala1545-acd', '*bayu'), ('to_pound', 'alor1247-besar', 'baʤɔ '), ('to_pound', 'alor1247-pandai', 'badʒɔ '), ('to_pound', 'lama1277-kalik', 'ˈbaʤu'), ('to_pound', 'sika1262-hewa', 'bai'), ('to_pound', 'lama1277-lerek', 'baˈdʒ͡o'), ('to_pound', 'keda1252-leuba', 'bae'), ('to_pound', 'alor1247-munas', 'badʒɔ '), ('to_pound', 'lama1277-lewoi', 'ˈbajo'), ('to_pound', 'lama1277-adona', 'ˈbadʒo')}


Concept ‘twenty_four’ is different between Alorese and Lamaholot.

 Alorese innovated the following forms:
 {('twenty_four', 'alor1247-pandai', "karua 'ilak 'pa"), ('twenty_four', 'alor1247-munas', "karua 'ilak 'paʔ"), ('twenty_four', 'alor1247-besar', "karua 'ilaka 'pa")}
 One or more Lamaholot dialects innovated forms not found in Alorese:
 {('twenty_four', 'lama1277-lewoi', 'pulu rua noʔoŋ ˈpaː'), ('twenty_four', 'lama1277-kalik', 'ˈpulu ˈʤua no pat'), ('twenty_four', 'lama1277-lerek', 'pul dʒ͡ə no ˈpatə'), ('twenty_four', 'sika1262-hewa', 'pulu rua ʋot hutu'), ('twenty_four', 'lama1277-adona', 'pulu rua nɔ ˈpaːt')}


Concept ‘bitter’ is different between Alorese and Lamaholot.

 One or more Lamaholot dialects innovated forms not found in Alorese:
 {('bitter', 'lama1277-lerek', 'pnetəˈnən')}
 {('bitter', 'lama1277-lerek', 'ˈpeta-ˈpeta')}
 {('bitter', 'sarr1247-nule', 'bene:r'), ('bitter', 'lama1277-kalik', 'pəˈnait'), ('bitter', 'teiw1235', "ba'ne:r")}

  Just for clarity:
  There are also the following similarity classes shared between Alorese and LH.
  {('bitter', 'lama1277-adona', 'ˈpait'), ('bitter', 'lama1277-lewoi', 'ˈpaik'), ('bitter', 'alor1247-besar', 'pei'), ('bitter', 'alor1247-munas', 'paiʔ'), ('bitter', 'alor1247-pandai', 'pai'), ('bitter', 'keda1252-leuba', 'pɛiʔ')}


Concept ‘God’ is different between Alorese and Lamaholot.

 Alorese shares the following forms with TAP languages:
 {('God', 'kula1280-lanto', 'la’tala'), ('God', 'pura1258', "Laha'tala"), ('God', 'abui1241-takal', "lä.hä.'tä.lä"), ('God', 'alor1247-pandai', "a'lapɔ 'laha 'tala"), ('God', 'kaer1234', 'Latala'), ('God', 'teiw1235', 'La:tal'), ('God', 'wers1238-marit', 'latal'), ('God', 'lamm1241-westp', 'Latala'), ('God', 'adan1251-otvai', '’lahtal'), ('God', 'abui1241-ulaga', 'alasala'), ('God', 'sawi1256', "la'tala"), ('God', 'rett1240', 'lahatala'), ('God', 'kelo1247-hopte', "lah'tal")}
 One or more Lamaholot dialects innovated forms not found in Alorese:
 {('God', 'lama1277-adona', 'ˈʔalatɜt')}
 {('God', 'lama1277-lewoi', 'ləra ˈwulaŋ')}

  Just for clarity:
  There are also the following similarity classes shared between Alorese and LH.
  {('God', 'alor1247-munas', "a'lapːɔ"), ('God', 'lama1277-kalik', 'aˈlap-sa'), ('God', 'lama1277-lerek', 'ˈalepəha'), ('God', 'alor1247-besar', "a'lapa")}


Concept ‘rich’ is different between Alorese and Lamaholot.

 Alorese shares the following forms with other AN languanges, but not Lamaholot:
 {('rich', 'sika1262-hewa', 'bisaʔ'), ('rich', 'alor1247-munas', 'bisaŋ')}
 One or more Lamaholot dialects innovated forms not found in Alorese:
 {('rich', 'keda1252-leuba', 'kaja'), ('rich', 'lama1277-lewoi', 'ˈkaja')}

  Just for clarity:
  There are also the following similarity classes shared between Alorese and LH.
  {('rich', 'kelo1247-hopte', "kaka'wa:h"), ('rich', 'alor1247-pandai', "k'wasaŋ"), ('rich', 'adan1251-otvai', 'ka’faɟ'), ('rich', 'lama1277-adona', 'kəˈwasa'), ('rich', 'alor1247-munas', "k'wasaŋ"), ('rich', 'sawi1256', "ka'wasa"), ('rich', 'alor1247-besar', "ka'fasaŋ"), ('rich', 'wers1238-marit', "kə'was"), ('rich', 'pura1258', 'kawasa'), ('rich', 'kama1365', 'kawasa'), ('rich', 'lama1277-kalik', 'kovaˈsan'), ('rich', 'lama1277-lerek', 'kəvaˈhan'), ('rich', 'rett1240', 'kavasa')}


Concept ‘machete’ is different between Alorese and Lamaholot.

 One or more Lamaholot dialects innovated forms not found in Alorese:
 {('machete', 'lama1277-lewoi', 'kəˈnumɛ')}

  Just for clarity:
  There are also the following similarity classes shared between Alorese and LH.
  {('machete', 'kuii1253', 'peda'), ('machete', 'kama1365', 'sapaːra'), ('machete', 'blag1240-kulij', 'peda'), ('machete', 'kelo1247-bring', 'pɛd'), ('machete', 'alor1247-baran', 'pɛda'), ('machete', 'lama1277-adona', 'pɛda'), ('machete', 'kira1248', 'sapada'), ('machete', 'kelo1247-hopte', 'pɛd'), ('machete', 'baka1276', 'peda'), ('machete', 'maka1316', 'sita'), ('machete', 'abui1241-ulaga', 'pedaŋ'), ('machete', 'kaer1234', 'pɛ:d'), ('machete', 'nede1245', 'peda'), ('machete', 'blag1240-tuntu', 'peda'), ('machete', 'teiw1235', 'pɛ:d'), ('machete', 'lama1277-kalik', 'peˈda'), ('machete', 'blag1240-nule', 'peda'), ('machete', 'kira1248', 'peda'), ('machete', 'sawi1256', 'pida'), ('machete', 'hama1240', 'səpad'), ('machete', 'wers1238-marit', 'ped'), ('machete', 'pura1258', "sa'pada"), ('machete', 'alor1247-pandai', 'peda'), ('machete', 'keda1252-leuba', "pɛ'daʔ"), ('machete', 'wers1238-taram', 'pede'), ('machete', 'adan1251-otvai', 'sa’pad'), ('machete', 'rett1240', 'sapada'), ('machete', 'alor1247-besar', "sa'pada"), ('machete', 'kabo1247', 'spada'), ('machete', 'lamm1241-westp', 'pera'), ('machete', 'alor1247-munas', 'pedaʔ'), ('machete', 'lama1277-kalik', 'peˈdar'), ('machete', 'kafo1240', 'pɛdɑŋ'), ('machete', 'abui1241-petle', "sa.'pa.da"), ('machete', 'hama1240', 'sapad'), ('machete', 'blag1240-bama', 'peda'), ('machete', 'blag1240-warsa', 'peda'), ('machete', 'rett1240', "sa'pada"), ('machete', 'lama1277-lerek', 'ˈpeda'), ('machete', 'adan1251-lawah', 'sapada'), ('machete', 'dein1238', 'pe:t'), ('machete', 'sarr1247-nule', 'pe:d'), ('machete', 'sarr1247-adiab', 'pe:d')}


Concept ‘2sg_polite’ is different between Alorese and Lamaholot.

 One or more Lamaholot dialects innovated forms not found in Alorese:
 {('2sg_polite', 'lama1277-lerek', 'kəˈneu')}

  Just for clarity:
  There are also the following similarity classes shared between Alorese and LH.
  {('2sg_polite', 'alor1247-munas', 'mo'), ('2sg_polite', 'alor1247-pandai', 'mo'), ('2sg_polite', 'alor1247-besar', 'mo'), ('2sg_polite', 'lama1277-lewoi', 'moː'), ('2sg_polite', 'alor1247-baran', 'mɔ:')}


Concept ‘rope’ is different between Alorese and Lamaholot.

 One or more Lamaholot dialects innovated forms not found in Alorese:
 {('rope', 'lama1277-lewot', 'fədəku'), ('rope', 'lama1277-lerek', 'vəˈdək'), ('rope', 'lama1277-lewuk', 'wəkat'), ('rope', 'keda1252', 'wade'), ('rope', 'keda1252-leuba', "wa'dɛʔ"), ('rope', 'keda1252-leuwa', 'vadεʔ'), ('rope', 'lama1277-minga', 'fədak'), ('rope', 'lama1277-imulo', 'fədək'), ('rope', 'lama1277-lewop', 'fədek')}

  Just for clarity:
  There are also the following similarity classes shared between Alorese and LH.
  {('rope', 'sika1262-tanai', 'tali'), ('rope', 'lama1277-kalik', 'tale'), ('rope', 'tetu1245-vique', 'tali'), ('rope', 'lama1277-lewoe', 'tale'), ('rope', 'lama1277-baipi', 'tale'), ('rope', 'lama1277-tanju', 'tale'), ('rope', 'lama1277-pukau', 'tale'), ('rope', 'p-mala1545-abvd', '*talih'), ('rope', 'abui1241-ulaga', 'tila'), ('rope', 'lama1277-adona', 'talɛʔ'), ('rope', 'kema1243', 'tali'), ('rope', 'lama1277-lamak', 'tale'), ('rope', 'lama1277-mulan', 'tale'), ('rope', 'koto1251', 'tani'), ('rope', 'alor1247-munas', 'talɛ'), ('rope', 'alor1247-besar', 'talɛ'), ('rope', 'sika1262-maume', 't̪ali'), ('rope', 'lama1277-botun', 'tale'), ('rope', 'lama1277-lewoi', 'talɛ'), ('rope', 'tetu1245-suai', 'tali'), ('rope', 'lama1277-kalik', 'taˈli'), ('rope', 'lama1277-merde', 'tale'), ('rope', 'lama1277-horow', 'tale'), ('rope', 'tetu1246', 'tɑli'), ('rope', 'alor1247-pandai', 'talɛ'), ('rope', 'lama1277-belan', 'tale'), ('rope', 'lama1277-lewob', 'tale'), ('rope', 'lama1277-kalik', 'taˈlɛr'), ('rope', 'alor1247-baran', 'tale'), ('rope', 'lama1277-lewom', 'tale'), ('rope', 'lama1277-ritae', 'tale'), ('rope', 'lama1277-wuake', 'tale'), ('rope', 'p-aust1307-abvd', '*CaliS'), ('rope', 'idat1237', 'tɑli'), ('rope', 'lama1277-waiwa', 'tale'), ('rope', 'maka1316', 'tali'), ('rope', 'abui1241-takal', 'tʰi.lä'), ('rope', 'atim1239', 'ˈtila'), ('rope', 'p-mala1545-acd', '*talih'), ('rope', 'lama1277-lewog', 'tale'), ('rope', 'mamb1306', 'tal'), ('rope', 'lama1277-lamal', 'tale'), ('rope', 'p-cent2245-abvd', '*tali'), ('rope', 'lama1277-kiwan', 'taleʔ'), ('rope', 'lama1277-waiba', 'tale'), ('rope', 'laka1255', 'tali'), ('rope', 'abui1241-fuime', 'tila'), ('rope', 'lama1277-lamah', 'tale'), ('rope', 'lama1277-lewok', 'tali'), ('rope', 'lama1277-ileap', 'tale'), ('rope', 'lama1277-paina', 'tali'), ('rope', 'lama1277-lamat', 'tale'), ('rope', 'tuku1254', 'tali'), ('rope', 'sika1262-hewa', 'tali'), ('rope', 'lama1277-bama', 'tale'), ('rope', 'mamb1306', 'taɛl'), ('rope', 'lama1277-dulhi', 'tale'), ('rope', 'lama1277-watan', 'taleʔ')}


Concept ‘milk’ is different between Alorese and Lamaholot.

 One or more Lamaholot dialects innovated forms not found in Alorese:
 {('milk', 'lama1277-adona', 'ˈtuhɔ'), ('milk', 'lama1277-kalik', 'tuˈsɔr')}

  Just for clarity:
  There are also the following similarity classes shared between Alorese and LH.
  {('milk', 'alor1247-besar', 'susu'), ('milk', 'idat1237', "susu 'wɛ:r"), ('milk', 'kuii1253', 'su'), ('milk', 'sika1262-hewa', 'susu'), ('milk', 'mamb1306', "su'sɛra"), ('milk', 'koto1251', 'susu'), ('milk', 'kira1248', 'su'), ('milk', 'lama1277-kalik', 'tuˈsu'), ('milk', 'adan1251-otvai', '’susu'), ('milk', 'alor1247-baran', 'susu'), ('milk', 'kula1280-lanto', '’susu'), ('milk', 'tuku1254', 'susɛ'), ('milk', 'tetu1245-vique', 'susu wen'), ('milk', 'tetu1246', ",susu'bɛn"), ('milk', 'kema1243', "susu 'biɑn"), ('milk', 'tetu1245-suai', 'susu wen'), ('milk', 'alor1247-munas', 'susu'), ('milk', 'alor1247-pandai', 'susu'), ('milk', 'lama1277-lewoi', 'ˈsusu'), ('milk', 'kula1280-lanto', 'susu'), ('milk', 'lama1277-lerek', 'ˈsusu'), ('milk', 'laka1255', "susu 'wɛ:r"), ('milk', 'pura1258', 'susu'), ('milk', 'abui1241-ulaga', 'susu')}


Concept ‘itchy’ is different between Alorese and Lamaholot.

 One or more Lamaholot dialects innovated forms not found in Alorese:
 {('itchy', 'lama1277-lerek', 'kənəˈha')}

  Just for clarity:
  There are also the following similarity classes shared between Alorese and LH.
  {('itchy', 'p-mala1545-acd', '*atel'), ('itchy', 'alor1247-munas', 'gateʔ'), ('itchy', 'alor1247-pandai', 'gate'), ('itchy', 'p-mala1545-acd', '*gatel'), ('itchy', 'lama1277-lewoi', 'ˈɡatə'), ('itchy', 'alor1247-besar', 'gate'), ('itchy', 'p-mala1545-acd', '*ma-gatel'), ('itchy', 'lama1277-adona', 'ˈɡatɜk'), ('itchy', 'sika1262-hewa', 'ɡatar'), ('itchy', 'lama1277-kalik', 'ɡəˈtək'), ('itchy', 'tetu1245-vique', 'katar'), ('itchy', 'tetu1245-suai', 'katar')}


Concept ‘fishnet’ is different between Alorese and Lamaholot.

 One or more Lamaholot dialects innovated forms not found in Alorese:
 {('fishnet', 'lama1277-lewoi', 'ˈalaʔ'), ('fishnet', 'lama1277-adona', 'ala'), ('fishnet', 'sika1262-hewa', 'a:la')}

  Just for clarity:
  There are also the following similarity classes shared between Alorese and LH.
  {('fishnet', 'adan1251-otvai', '’puko'), ('fishnet', 'alor1247-pandai', 'pukɔ'), ('fishnet', 'teiw1235', "pu'kat"), ('fishnet', 'kaer1234', "pu'kat"), ('fishnet', 'lama1277-lerek', 'ˈpukat'), ('fishnet', 'kama1365', 'bit'), ('fishnet', 'alor1247-munas', 'pukɔʔ'), ('fishnet', 'alor1247-besar', 'pukɔ'), ('fishnet', 'lama1277-kalik', 'ˈpukət'), ('fishnet', 'pura1258', 'pukat'), ('fishnet', 'keda1252-leuba', 'pueʔ'), ('fishnet', 'lama1277-adona', 'ˈpukɜt')}


Concept ‘cheek’ is different between Alorese and Lamaholot.

 One or more Lamaholot dialects innovated forms not found in Alorese:
 {('cheek', 'lama1277-lerek', 'kliˈpi'), ('cheek', 'lama1277-lewoi', 'kəˈlipi')}

  Just for clarity:
  There are also the following similarity classes shared between Alorese and LH.
  {('cheek', 'alor1247-munas', 'pipiŋ'), ('cheek', 'alor1247-munas', 'pipiʔ'), ('cheek', 'p-mala1545-acd', '*pipi'), ('cheek', 'keda1252-leuba', 'pipin'), ('cheek', 'sika1262-hewa', 'pipi'), ('cheek', 'p-mala1545-acd', '*piŋi'), ('cheek', 'lama1277-adona', 'ˈpipiˈkɜt'), ('cheek', 'alor1247-pandai', 'pipiŋ'), ('cheek', 'alor1247-besar', 'pipiŋ'), ('cheek', 'lama1277-kalik', 'piˈpi')}


Concept ‘sarong_women’ is different between Alorese and Lamaholot.

 One or more Lamaholot dialects innovated forms not found in Alorese:
 {('sarong_women', 'lama1277-kalik', 'kreˈɔt'), ('sarong_women', 'lama1277-kalik', 'kreˈmɔt')}
 {('sarong_women', 'lama1277-lerek', 'pəˈtək')}

  Just for clarity:
  There are also the following similarity classes shared between Alorese and LH.
  {('sarong_women', 'lama1277-lewoi', 'kəˈwatə'), ('sarong_women', 'alor1247-munas', "k'wateʔ"), ('sarong_women', 'alor1247-besar', "ka'fate"), ('sarong_women', 'alor1247-pandai', "k'wate"), ('sarong_women', 'lama1277-adona', 'kəˈwata')}


Concept ‘voice’ is different between Alorese and Lamaholot.

 Alorese shares the following forms with other AN languanges, but not Lamaholot:
 {('voice', 'alor1247-munas', 'alaʔ'), ('voice', 'alor1247-munas', 'alaŋ'), ('voice', 'alor1247-pandai', 'alaŋ'), ('voice', 'alor1247-besar', 'alaŋ'), ('voice', 'tetu1245-vique', 'lian'), ('voice', 'tetu1245-suai', 'lian')}
 One or more Lamaholot dialects innovated forms not found in Alorese:
 {('voice', 'lama1277-adona', 'ˈrãː'), ('voice', 'lama1277-lerek', 'ˈraŋa'), ('voice', 'lama1277-lewoi', 'ˈraːŋ'), ('voice', 'lama1277-kalik', 'raŋ')}


Concept ‘papaya’ is different between Alorese and Lamaholot.

 Alorese shares the following forms with TAP languages:
 {('papaya', 'buna1278-suai', 'dila'), ('papaya', 'alor1247-pandai', 'sela'), ('papaya', 'buna1278-bobon', 'dila'), ('papaya', 'alor1247-munas', 'sela'), ('papaya', 'alor1247-pandai', 'tela')}
 {('papaya', 'alor1247-pandai', 'kasi'), ('papaya', 'kaer1234', "ka'si"), ('papaya', 'blag1240-warsa', 'kasi'), ('papaya', 'abui1241-ulaga', 'kasal'), ('papaya', 'sarr1247-nule', 'qaja'), ('papaya', 'baka1276', 'kasi'), ('papaya', 'teiw1235', 'kasi'), ('papaya', 'blag1240-bama', 'kasi'), ('papaya', 'rett1240', 'kasi'), ('papaya', 'blag1240-nule', 'kasi'), ('papaya', 'blag1240-kulij', 'kasi'), ('papaya', 'sarr1247-adiab', "qa'ja"), ('papaya', 'alor1247-besar', 'kasi'), ('papaya', 'pura1258', 'kasi'), ('papaya', 'blag1240-tuntu', 'kasi')}
 One or more Lamaholot dialects innovated forms not found in Alorese:
 {('papaya', 'keda1252-leuba', "wa'ja"), ('papaya', 'lama1277-lewoi', 'wua ˈpajɑŋ'), ('papaya', 'lama1277-adona', 'paˈjã'), ('papaya', 'kelo1247-hopte', "ʔə'temɔl"), ('papaya', 'abui1241-takal', 'bä.tä: mäl'), ('papaya', 'lama1277-kalik', 'paˈʤam'), ('papaya', 'abui1241-fuime', 'bata: mija'), ('papaya', 'atim1239', 'batamia'), ('papaya', 'lama1277-lerek', 'paˈjam')}


Concept ‘body_hair’ is different between Alorese and Lamaholot.

 Alorese shares the following forms with other AN languanges, but not Lamaholot:
 {('body_hair', 'sika1262-hewa', 'ʋuluʔ'), ('body_hair', 'alor1247-munas', 'wuluk'), ('body_hair', 'p-mala1545-acd', '*bulu'), ('body_hair', 'keda1252-leuba', 'wurun'), ('body_hair', 'alor1247-pandai', 'wuluk'), ('body_hair', 'alor1247-besar', "fu'lukuŋ")}
 One or more Lamaholot dialects innovated forms not found in Alorese:
 {('body_hair', 'tetu1245-vique', 'rahuk'), ('body_hair', 'tetu1245-suai', 'rahuk'), ('body_hair', 'lama1277-lewoi', 'rawuˈkəŋ'), ('body_hair', 'lama1277-kalik', 'raˈvʊk'), ('body_hair', 'lama1277-adona', 'ˈrawuˈkɜt')}
 {('body_hair', 'lama1277-lerek', 'riˈau')}


Concept ‘wall’ is different between Alorese and Lamaholot.

 Alorese shares the following forms with other AN languanges, but not Lamaholot:
 {('wall', 'adan1251-otvai', 'ka’bɛʔ'), ('wall', 'alor1247-munas', 'keba'), ('wall', 'alor1247-munas', "gə'bːe"), ('wall', 'sika1262-hewa', 'gəbi')}

  Just for clarity:
  There are also the following similarity classes shared between Alorese and LH.
  {('wall', 'rett1240', 'ganɛbɛ'), ('wall', 'alor1247-besar', "ga'nabɛŋ"), ('wall', 'lama1277-lerek', 'kənaˈber'), ('wall', 'alor1247-pandai', "g'nabːɛŋ"), ('wall', 'lama1277-kalik', 'kənəˈbɛr'), ('wall', 'lama1277-adona', 'ˈkɜnɜˈbiʔ'), ('wall', 'keda1252-leuba', "nɛ'biʔ"), ('wall', 'lama1277-lewoi', 'kəˈnəbi'), ('wall', 'lama1277-kalik', 'kənəˈbin'), ('wall', 'pura1258', "ga'nebe")}


Concept ‘mat’ is different between Alorese and Lamaholot.

 Alorese innovated the following forms:
 {('mat', 'alor1247-baran', "sa'fae")}

  Just for clarity:
  There are also the following similarity classes shared between Alorese and LH.
  {('mat', 'alor1247-munas', "ɔ'haŋ"), ('mat', 'alor1247-munas', 'hoaŋ'), ('mat', 'alor1247-pandai', 'ɔhaŋ'), ('mat', 'lama1277-adona', 'ˈʔɔhãː'), ('mat', 'sika1262-hewa', 'oha?'), ('mat', 'lama1277-kalik', 'oˈsan'), ('mat', 'alor1247-besar', "ɔ'haŋ"), ('mat', 'lama1277-kalik', 'oˈsa'), ('mat', 'lama1277-lewoi', 'ˈɔhɑŋ'), ('mat', 'lama1277-lerek', 'ˈohan')}


Concept ‘excrement’ is different between Alorese and Lamaholot.

 Alorese innovated the following forms:
 {('excrement', 'alor1247-munas', "witi 'taiŋ"), ('excrement', 'alor1247-pandai', "witi 'taiŋ")}

  Just for clarity:
  There are also the following similarity classes shared between Alorese and LH.
  {('excrement', 'koto1251', 'tei'), ('excrement', 'lama1277-kalik', 'taˈi'), ('excrement', 'alor1247-besar', 'taɛ'), ('excrement', 'p-mala1545-acd', '*taqay'), ('excrement', 'alor1247-munas', 'taiŋ'), ('excrement', 'lama1277-adona', 'tae'), ('excrement', 'lama1277-lewoi', 'ˈtɑe'), ('excrement', 'p-mala1545-acd', '*taqi'), ('excrement', 'sika1262-hewa', 'taʔi'), ('excrement', 'alor1247-munas', "sapi 'taiŋ"), ('excrement', 'lama1277-lerek', 'taer'), ('excrement', 'abui1241-takal', 'täɁi'), ('excrement', 'alor1247-pandai', "sapi 'taiŋ"), ('excrement', 'lama1277-kalik', 'taˈɛr')}


Concept ‘bat’ is different between Alorese and Lamaholot.

 Alorese innovated the following forms:
 {('bat', 'alor1247-besar', "la'bɛruŋ")}
 Alorese shares the following forms with other AN languanges, but not Lamaholot:
 {('bat', 'keda1252-leuba', "mi'ʔi"), ('bat', 'alor1247-pandai', 'mike'), ('bat', 'alor1247-munas', 'mikɛ')}
 One or more Lamaholot dialects innovated forms not found in Alorese:
 {('bat', 'lama1277-kalik', 'krɛˈbɛ'), ('bat', 'lama1277-lerek', 'kəreˈbeŋ'), ('bat', 'lama1277-kalik', 'krɛˈbɛŋ')}

  Just for clarity:
  There are also the following similarity classes shared between Alorese and LH.
  {('bat', 'lama1277-adona', 'pɜˈnike'), ('bat', 'sika1262-hewa', 'niʔi'), ('bat', 'alor1247-pandai', "pə'nike"), ('bat', 'lama1277-lewoi', 'pəniˈke'), ('bat', 'tetu1245-vique', 'niki'), ('bat', 'tetu1245-suai', 'niki')}


Concept ‘right_side’ is different between Alorese and Lamaholot.

 Alorese shares the following forms with other AN languanges, but not Lamaholot:
 {('right_side', 'alor1247-besar', 'dikɛ̃'), ('right_side', 'p-mala1545-acd', '*taqu'), ('right_side', 'p-mala1545-acd', '*ma-taqu'), ('right_side', 'alor1247-pandai', 'dikɛŋ'), ('right_side', 'alor1247-munas', 'dikɛŋ'), ('right_side', 'p-mala1545-acd', '*ka-taqu'), ('right_side', 'alor1247-baran', "di'kɛ̃:")}
 One or more Lamaholot dialects innovated forms not found in Alorese:
 {('right_side', 'lama1277-tanju', 'wana'), ('right_side', 'lama1277-lewok', 'wanã'), ('right_side', 'lama1277-lerek', 'vaˈnan'), ('right_side', 'lama1277-minga', 'wana'), ('right_side', 'lama1277-merde', 'wanã'), ('right_side', 'keda1252-leuwa', 'vana'), ('right_side', 'lama1277-kalik', 'vaˈna'), ('right_side', 'tetu1245-suai', 'kwana'), ('right_side', 'lama1277-bama', 'wana'), ('right_side', 'lama1277-lamah', 'wana'), ('right_side', 'lama1277-waiwa', 'wanan'), ('right_side', 'lama1277-lewop', 'fanan'), ('right_side', 'p-mala1545-abvd', '*ka-wanan'), ('right_side', 'sika1262-maume', 'vana'), ('right_side', 'lama1277-imulo', 'fanan'), ('right_side', 'lama1277-lamal', 'fana'), ('right_side', 'lama1277-lewoe', 'wana'), ('right_side', 'keda1252', 'wana'), ('right_side', 'lama1277-lamat', 'wana'), ('right_side', 'lama1277-lewog', 'wanã'), ('right_side', 'lama1277-lewot', 'fanan'), ('right_side', 'lama1277-kalik', 'wana'), ('right_side', 'lama1277-lewob', 'wana'), ('right_side', 'p-aust1307-abvd', '*ka-wanaN'), ('right_side', 'lama1277-watan', 'wanã'), ('right_side', 'lama1277-pukau', 'wana'), ('right_side', 'sika1262-hewa', 'wanan'), ('right_side', 'lama1277-kiwan', 'wana'), ('right_side', 'lama1277-waiba', 'wana'), ('right_side', 'lama1277-horow', 'wanã'), ('right_side', 'lama1277-belan', 'wana'), ('right_side', 'lama1277-lewuk', 'wana'), ('right_side', 'sika1262-tanai', 'βana'), ('right_side', 'keda1252-leuba', 'wana'), ('right_side', 'lama1277-lewoi', 'ˈwanaŋ'), ('right_side', 'lama1277-ileap', 'wanan'), ('right_side', 'sika1262-hewa', 'ʋanan'), ('right_side', 'lama1277-lewom', 'wana'), ('right_side', 'lama1277-ritae', 'wana'), ('right_side', 'lama1277-baipi', 'wana'), ('right_side', 'kema1243', "bɑ'nala"), ('right_side', 'lama1277-dulhi', 'wanã'), ('right_side', 'lama1277-botun', 'wanã'), ('right_side', 'lama1277-wuake', 'wana'), ('right_side', 'lama1277-paina', 'wanan'), ('right_side', 'p-cent2245-abvd', '*ka-wanan'), ('right_side', 'lama1277-mulan', 'wɤnɤ'), ('right_side', 'lama1277-adona', 'ˈwana'), ('right_side', 'lama1277-lamak', 'wanã')}


Concept ‘no_not’ is different between Alorese and Lamaholot.

 Alorese shares the following forms with other AN languanges, but not Lamaholot:
 {('no_not', 'alor1247-munas', 'lahɛ'), ('no_not', 'alor1247-baran', "la'h:ɛ"), ('no_not', 'alor1247-munas', 'lahɛʔ'), ('no_not', 'alor1247-pandai', 'lahɛ'), ('no_not', 'alor1247-besar', 'lahɛ'), ('no_not', 'idat1237', 'lɑek'), ('no_not', 'tetu1246', 'lae'), ('no_not', 'tetu1245-suai', 'laɛ'), ('no_not', 'tetu1245-vique', 'laɛk'), ('no_not', 'tetu1246', 'lɑe')}
 One or more Lamaholot dialects innovated forms not found in Alorese:
 {('no_not', 'lama1277-kalik', 'ˈta'), ('no_not', 'kema1243', 'tai'), ('no_not', 'p-cent2245-abvd', '*ta-i'), ('no_not', 'laka1255', 'tɑi'), ('no_not', 'lama1277-kalik', 'ˈtak'), ('no_not', 'lama1277-lerek', 'taˈken'), ('no_not', 'lama1277-lewoi', 'ˈtake'), ('no_not', 'mamb1306', "tɑi 'sid"), ('no_not', 'p-cent2245-abvd', '*ta'), ('no_not', 'p-mala1545-acd', '*ta'), ('no_not', 'tuku1254', 'tɑʔi'), ('no_not', 'lama1277-kalik', 'ˈtake')}
 {('no_not', 'lama1277-adona', 'ˈhalaʔ')}
 {('no_not', 'p-mala1545-acd', '*dian'), ('no_not', 'p-mala1545-abvd', '*diaq'), ('no_not', 'p-cent2245-abvd', '*diaq'), ('because', 'sika1262-hewa', 'əle'), ('because', 'lama1277-lewoe', 'ʔele'), ('because', 'keda1252-leuba', 'ʔele'), ('no_not', 'p-mala1545-acd', '*adi'), ('because', 'keda1252', 'eleʔ'), ('because', 'keda1252-leuwa', 'εlε')}


Concept ‘tree’ is different between Alorese and Lamaholot.

 One or more Lamaholot dialects innovated forms not found in Alorese:
 {('tree', 'lama1277-lamat', 'əso'), ('tree', 'lama1277-lewoe', 'əso')}

  Just for clarity:
  There are also the following similarity classes shared between Alorese and LH.
  {('tree', 'lama1277-mulan', 'kajo'), ('tree', 'lama1277-botun', 'kayo'), ('tree', 'lama1277-imulo', 'kajor'), ('tree', 'p-aust1307-abvd', '*(kayu)'), ('tree', 'lama1277-belan', 'kajo'), ('tree', 'alor1247-besar', 'kaʤɔ'), ('tree', 'lama1277-kalik', 'ˈkaʤɔr'), ('tree', 'lama1277-horow', 'kayo'), ('tree', 'lama1277-kalik', 'kaju'), ('tree', 'lama1277-lewot', 'kajoru'), ('tree', 'lama1277-lerek', 'ˈkajor'), ('tree', 'lama1277-lewop', 'kaju'), ('tree', 'lama1277-kalik', 'ˈkaʤu'), ('tree', 'lama1277-lewom', 'kajo'), ('tree', 'lama1277-ritae', 'kayo'), ('tree', 'lama1277-pukau', 'kayu'), ('tree', 'lama1277-merde', 'kayo'), ('tree', 'lama1277-lewog', 'kayo'), ('tree', 'lama1277-tanju', 'kajo'), ('tree', 'lama1277-lamak', 'kajo'), ('tree', 'lama1277-waiwa', 'kayo'), ('tree', 'lama1277-ileap', 'kayo'), ('tree', 'lama1277-bama', 'kayo'), ('tree', 'lama1277-baipi', 'kajo'), ('tree', 'lama1277-lewok', 'kayoru'), ('tree', 'lama1277-adona', 'ˈkajo'), ('tree', 'lama1277-paina', 'kayo'), ('tree', 'alor1247-baran', "kaʤu 'lɔlɔŋ"), ('tree', 'lama1277-wuake', 'kajo'), ('tree', 'lama1277-lamah', 'kayo'), ('tree', 'lama1277-lewuk', 'kajor'), ('tree', 'lama1277-waiba', 'kajo'), ('tree', 'lama1277-lamal', 'kajo'), ('tree', 'lama1277-watan', 'kajo'), ('tree', 'lama1277-kiwan', 'kayo'), ('tree', 'lama1277-dulhi', 'karo'), ('tree', 'lama1277-minga', 'kaju')}
  {('tree', 'lama1277-lewob', 'kajopukã'), ('tree', 'alor1247-pandai', "kaʤɔ 'pukɔŋ"), ('tree', 'lama1277-lewoi', 'ˈpukəŋ'), ('tree', 'alor1247-munas', "kaʤɔ 'pukɔŋ")}


Concept ‘cookhouse’ is different between Alorese and Lamaholot.

 Alorese innovated the following forms:
 {('cookhouse', 'alor1247-besar', 'lika')}
 {('cookhouse', 'alor1247-pandai', 'awo'), ('cookhouse', 'alor1247-munas', "pawɔ 'awɔ")}
 One or more Lamaholot dialects innovated forms not found in Alorese:
 {('cookhouse', 'lama1277-kalik', 'lura'), ('cookhouse', 'lama1277-kalik', 'luran'), ('cookhouse', 'lama1277-adona', 'ˈrurã')}
 {('cookhouse', 'abui1241-ulaga', 'dapur'), ('cookhouse', 'lama1277-lewoi', 'ˈdapur'), ('cookhouse', 'maka1316', "da'buru"), ('cookhouse', 'wers1238-marit', 'dapur'), ('cookhouse', 'buna1278-suai', 'daphɔr'), ('cookhouse', 'sawi1256', 'dapuru'), ('cookhouse', 'sika1262-hewa', 'dapur'), ('cookhouse', 'rett1240', 'dampur'), ('cookhouse', 'pura1258', 'dampur'), ('cookhouse', 'fata1247', 'capur'), ('cookhouse', 'adan1251-otvai', '‘’dapur'), ('cookhouse', 'buna1278-bobon', 'daphɔr'), ('cookhouse', 'kula1280-lanto', '’dapur')}


Concept ‘sermon’ is different between Alorese and Lamaholot.

 Alorese innovated the following forms:
 {('sermon', 'alor1247-munas', "tutu 'indʒil"), ('sermon', 'alor1247-besar', 'tutu')}
 {('sermon', 'alor1247-pandai', "s'rama")}
 One or more Lamaholot dialects innovated forms not found in Alorese:
 {('sermon', 'sika1262-hewa', 'kotba'), ('sermon', 'koto1251', 'kotba'), ('sermon', 'lama1277-lewoi', 'ˈkotba'), ('sermon', 'keda1252-leuba', 'xɔtbɑh'), ('sermon', 'teiw1235', 'xotbah')}
 {('sermon', 'lama1277-lewoi', 'ˈtutuʔ ˈmariŋ'), ('sermon', 'lama1277-adona', 'ˈnuã marĩ')}


Concept ‘floor’ is different between Alorese and Lamaholot.

 Alorese innovated the following forms:
 {('floor', 'alor1247-pandai', 'dasar')}
 {('floor', 'alor1247-besar', 'lamiŋ')}
 {('floor', 'alor1247-pandai', 'tana')}

  Just for clarity:
  There are also the following similarity classes shared between Alorese and LH.
  {('floor', 'lama1277-lewoi', 'ˈlɑnte'), ('floor', 'kaer1234', 'lantai'), ('floor', 'keda1252-leuba', 'lante'), ('floor', 'wers1238-marit', 'lante'), ('floor', 'kula1280-lanto', '’lantai'), ('floor', 'lama1277-lerek', 'ˈlante'), ('floor', 'sika1262-hewa', 'lante'), ('floor', 'alor1247-munas', 'lantɛʔ'), ('floor', 'abui1241-ulaga', 'lantai'), ('floor', 'lama1277-kalik', 'ˈlante'), ('floor', 'lama1277-adona', 'ˈlante')}


Concept ‘chew_betel’ is different between Alorese and Lamaholot.

 One or more Lamaholot dialects innovated forms not found in Alorese:
 {('chew_betel', 'lama1277-adona', 'ɡãːˈwuaʔ')}
 {('chew_betel', 'lama1277-lerek', 'ɡa ˈkleruk')}

  Just for clarity:
  There are also the following similarity classes shared between Alorese and LH.
  {('chew_betel', 'lama1277-lewoi', 'ˈtəkɑŋ wua ˈmalu'), ('chew_betel', 'keda1252-leuba', "ka uwe 'mal"), ('chew_betel', 'alor1247-munas', "kaŋ 'malu"), ('chew_betel', 'alor1247-besar', "ta'kːa 'ua 'malu"), ('chew_betel', 'lama1277-kalik', 'ɡa malɔˈlɔ'), ('chew_betel', 'alor1247-munas', "gouŋ 'malu"), ('chew_betel', 'alor1247-pandai', "gouŋ 'ua 'malu")}


Concept ‘sweat’ is different between Alorese and Lamaholot.

 Alorese innovated the following forms:
 {('sweat', 'alor1247-pandai', 'namːɛ'), ('sweat', 'alor1247-besar', "ha'name"), ('sweat', 'alor1247-munas', "nə'mɛk")}
 One or more Lamaholot dialects innovated forms not found in Alorese:
 {('sweat', 'lama1277-lerek', 'kərəˈwun'), ('sweat', 'keda1252-leuba', "re'wun"), ('sweat', 'sika1262-hewa', 'rəʋu'), ('sweat', 'lama1277-adona', 'ˈʔɜwũ'), ('sweat', 'lama1277-kalik', 'krəˈvun'), ('sweat', 'lama1277-lewoi', 'ˈəwuŋ')}


Concept ‘initiation_ceremony’ is different between Alorese and Lamaholot.

 Alorese innovated the following forms:
 {('initiation_ceremony', 'alor1247-pandai', "gə'hiŋ 'gapaŋ")}
 {('initiation_ceremony', 'alor1247-besar', "suna 'kara")}
 {('initiation_ceremony', 'alor1247-munas', "nadʒaŋ 'borɛŋ")}
 One or more Lamaholot dialects innovated forms not found in Alorese:
 {('initiation_ceremony', 'lama1277-lerek', 'ˈtule ˈmakat')}
 {('initiation_ceremony', 'lama1277-adona', 'ˈnuã')}


Concept ‘hour’ is different between Alorese and Lamaholot.

 Alorese shares the following forms with TAP languages:
 {('hour', 'teiw1235', 'dani'), ('hour', 'alor1247-munas', 'dane'), ('hour', 'alor1247-besar', 'dane'), ('hour', 'lamm1241-westp', 'dani')}
 Alorese shares the following forms with other AN languanges, but not Lamaholot:
 {('hour', 'fata1247', 'ɔras'), ('hour', 'alor1247-pandai', 'oras'), ('hour', 'tetu1245-vique', 'ɔras'), ('hour', 'buna1278-suai', 'oras'), ('hour', 'maka1316', 'orasa'), ('hour', 'kaer1234', "o'ras"), ('hour', 'tetu1245-suai', 'ɔras'), ('hour', 'buna1278-bobon', 'oras')}
 One or more Lamaholot dialects innovated forms not found in Alorese:
 {('hour', 'keda1252-leuba', 'dʒam'), ('hour', 'adan1251-otvai', '’ɟam'), ('hour', 'teiw1235', 'ɟam'), ('hour', 'lama1277-lewoi', 'ˈʤam'), ('hour', 'sika1262-hewa', 'jam')}
 {('hour', 'lama1277-adona', 'ˈdahu')}


Concept ‘to_refuse’ is different between Alorese and Lamaholot.

 Alorese shares the following forms with TAP languages:
 {('to_refuse', 'kaer1234', "go'tobuŋ"), ('to_refuse', 'alor1247-besar', 'tobaŋ'), ('to_refuse', 'rett1240', 'gatɔmba'), ('to_refuse', 'lamm1241-westp', 'dub:a'), ('to_refuse', 'pura1258', "a'tobaŋ")}
 One or more Lamaholot dialects innovated forms not found in Alorese:
 {('to_refuse', 'lama1277-kalik', 'oˈdʊk')}

  Just for clarity:
  There are also the following similarity classes shared between Alorese and LH.
  {('to_refuse', 'lama1277-lerek', 'ˈɡehiŋ'), ('to_refuse', 'alor1247-munas', "ɔnɔ 'gɛhiŋ"), ('to_refuse', 'lama1277-adona', 'ɡeˈhik'), ('to_refuse', 'alor1247-pandai', 'gɛhiŋ'), ('to_refuse', 'lama1277-lewoi', 'ˈɡehiʔ')}


Concept ‘bamboo’ is different between Alorese and Lamaholot.

 Alorese shares the following forms with other AN languanges, but not Lamaholot:
 {('bamboo', 'tetu1245-vique', 'au'), ('bamboo', 'alor1247-pandai', 'au'), ('bamboo', 'tetu1245-suai', 'au')}
 One or more Lamaholot dialects innovated forms not found in Alorese:
 {('bamboo', 'lama1277-lerek', 'ˈure'), ('bamboo', 'lama1277-kalik', 'uˈru'), ('bamboo', 'lama1277-kalik', 'uˈrɔr')}
 {('bamboo', 'sika1262-hewa', 'pəri'), ('bamboo', 'lama1277-adona', 'pɜˈrĩ'), ('bamboo', 'lama1277-kalik', 'pəˈri'), ('bamboo', 'keda1252-leuba', "be'riŋ"), ('bamboo', 'lama1277-kalik', 'bəˈlaŋ'), ('bamboo', 'lama1277-kalik', 'pəˈrim')}
 {('bamboo', 'lama1277-lewoi', 'ˈaːu')}

  Just for clarity:
  There are also the following similarity classes shared between Alorese and LH.
  {('bamboo', 'kuii1253', 'pitan'), ('bamboo', 'blag1240-tuntu', 'petuŋ'), ('bamboo', 'blag1240-nule', 'petuŋ vandaŋ'), ('bamboo', 'sarr1247-nule', 'peta'), ('bamboo', 'maka1316', 'betu'), ('bamboo', 'alor1247-besar', "pa'tuŋ"), ('bamboo', 'adan1251-otvai', '’pituŋ'), ('bamboo', 'alor1247-munas', "pə'tuŋ"), ('bamboo', 'rett1240', 'badaŋ pituŋ'), ('bamboo', 'sika1262-hewa', 'pətun'), ('bamboo', 'blag1240-bama', 'petuŋ'), ('bamboo', 'sarr1247-adiab', 'peita '), ('bamboo', 'blag1240-nule', 'petuŋ'), ('bamboo', 'wers1238-marit', "bɛ'tiŋ"), ('bamboo', 'kelo1247-hopte', 'pe:t'), ('bamboo', 'alor1247-pandai', "pa'tːuŋ"), ('bamboo', 'rett1240', 'pe:tuŋ'), ('bamboo', 'sarr1247-adiab', 'ʤa'), ('bamboo', 'wers1238-taram', 'betiŋ'), ('bamboo', 'pura1258', 'pɛtuŋ'), ('bamboo', 'lama1277-kalik', 'pəˈtu'), ('bamboo', 'baka1276', 'petuŋ'), ('bamboo', 'rett1240', 'petuŋ'), ('bamboo', 'kaer1234', "pe'tuŋ"), ('bamboo', 'rett1240', 'pituŋ kaka'), ('bamboo', 'kabo1247', 'peteŋ'), ('bamboo', 'teiw1235', "pe'tan"), ('bamboo', 'lama1277-kalik', 'pəˈtuŋ'), ('bamboo', 'keda1252-leuba', "pe'ruŋ")}


Concept ‘what’ is different between Alorese and Lamaholot.

 One or more Lamaholot dialects innovated forms not found in Alorese:
 {('what', 'lama1277-lewot', 'alaka'), ('what', 'lama1277-waiwa', 'aku'), ('what', 'lama1277-watan', 'aku'), ('what', 'lama1277-lamak', 'aku'), ('what', 'lama1277-ileap', 'aku'), ('what', 'lama1277-adona', 'ˈʔaku'), ('what', 'lama1277-botun', 'aku'), ('what', 'lama1277-dulhi', 'aku'), ('what', 'lama1277-lamal', 'alaka'), ('what', 'lama1277-kiwan', 'aku'), ('what', 'lama1277-kalik', 'naoga'), ('what', 'lama1277-minga', 'alaku'), ('what', 'lama1277-mulan', 'ale'), ('what', 'lama1277-wuake', 'alaku'), ('what', 'lama1277-lewuk', 'aleka'), ('what', 'lama1277-horow', 'aku'), ('what', 'buna1278-bobon', 'negɔ'), ('what', 'buna1278-suai', 'negɔ'), ('what', 'buna1278-malia', 'nɛgo'), ('what', 'lama1277-belan', 'alaku')}
 {('what', 'kula1280-lanto', 'na’dua'), ('what', 'p-aust1307-abvd', '*n-anu'), ('what', 'p-mala1545-abvd', '*-anu'), ('what', 'lama1277-lamat', 'naʔa'), ('what', 'sawi1256', "naga'dua")}
 {('what', 'lama1277-lewob', 'a'), ('what', 'lama1277-kalik', 'an'), ('what', 'lama1277-merde', 'naʔã'), ('what', 'lama1277-baipi', 'a'), ('what', 'lama1277-lewoi', 'ˈaː'), ('what', 'lama1277-lewog', 'a'), ('what', 'lama1277-lewom', 'ã'), ('what', 'lama1277-pukau', 'a'), ('what', 'lama1277-ritae', 'ã'), ('what', 'lama1277-tanju', 'a'), ('what', 'lama1277-bama', 'a'), ('what', 'lama1277-lamah', 'a'), ('what', 'lama1277-waiba', 'a')}
 {('what', 'lama1277-lewok', 'lane')}

  Just for clarity:
  There are also the following similarity classes shared between Alorese and LH.
  {('what', 'lama1277-lewop', 'kapa'), ('what', 'alor1247-besar', 'pai'), ('what', 'sika1262-hewa', 'apar'), ('what', 'alor1247-munas', 'apai'), ('what', 'p-mala1545-abvd', '*apa'), ('what', 'lama1277-imulo', 'kapa'), ('what', 'alor1247-munas', 'pai'), ('what', 'keda1252-leuba', "a'pe"), ('what', 'sika1262-maume', 'apa'), ('what', 'keda1252', 'ape'), ('what', 'alor1247-pandai', 'pai'), ('what', 'lama1277-lewoe', 'ape'), ('what', 'lama1277-lerek', 'aˈpɔi'), ('what', 'p-cent2245-abvd', '*sapa'), ('what', 'keda1252-leuwa', 'a̘pε'), ('what', 'p-mala1545-acd', '*apa'), ('what', 'sika1262-hewa', 'apa'), ('what', 'alor1247-besar', 'paru'), ('what', 'alor1247-baran', 'pei'), ('what', 'lama1277-paina', 'apay'), ('what', 'p-cent2245-abvd', '*apa')}


Concept ‘eye’ is different between Alorese and Lamaholot.

 One or more Lamaholot dialects innovated forms not found in Alorese:
 {('eye', 'lama1277-lamat', 'ulʔũ')}
 {('eye', 'lama1277-lewot', 'kuluk'), ('eye', 'lama1277-imulo', 'kulukəs'), ('eye', 'lama1277-lewoe', 'ulu'), ('eye', 'lama1277-minga', 'kuluk'), ('eye', 'lama1277-lewop', 'kuluka')}

  Just for clarity:
  There are also the following similarity classes shared between Alorese and LH.
  {('eye', 'p-mala1545-acd', '*mata'), ('eye', 'lama1277-pukau', 'mata'), ('eye', 'alor1247-pandai', 'mataŋ'), ('eye', 'lama1277-kalik', 'matan'), ('eye', 'lama1277-waiba', 'mata'), ('eye', 'lama1277-merde', 'matakə'), ('eye', 'lama1277-lewog', 'mata'), ('eye', 'sika1262-tanai', 'mata-ŋ'), ('eye', 'laka1255', 'mata'), ('eye', 'lama1277-wuake', 'mata'), ('eye', 'kema1243', "ma'tɑr"), ('eye', 'laka1255', 'au matak'), ('eye', 'keda1252-leuwa', 'matɔ'), ('eye', 'alor1247-besar', 'mataŋ'), ('eye', 'lama1277-lamak', 'matã'), ('eye', 'lama1277-watan', 'mata'), ('eye', 'lama1277-adona', 'ˈmatak'), ('eye', 'lama1277-lewob', 'mata'), ('eye', 'lama1277-mulan', 'matɤ'), ('eye', 'lama1277-lewok', 'mata'), ('eye', 'lama1277-waiwa', 'mata'), ('eye', 'mamb1306', 'mata'), ('eye', 'tetu1245-suai', 'matan'), ('eye', 'lama1277-ileap', 'matak'), ('eye', 'tuku1254', 'ma:t'), ('eye', 'keda1252', 'mato'), ('eye', 'lama1277-kalik', 'maˈta'), ('eye', 'lama1277-paina', 'matag'), ('eye', 'lama1277-lamah', 'matakə'), ('eye', 'lama1277-baipi', 'mata'), ('eye', 'lama1277-lewom', 'mata'), ('eye', 'lama1277-ritae', 'mata'), ('eye', 'sika1262-hewa', 'mata'), ('eye', 'sika1262-tanai', 'mata'), ('eye', 'p-mala1545-abvd', '*mata'), ('eye', 'lama1277-kiwan', 'mata'), ('eye', 'lama1277-lamal', 'matã'), ('eye', 'p-aust1307-abvd', '*maCA'), ('eye', 'lama1277-tanju', 'mata'), ('eye', 'lama1277-lewoi', 'ˈmata'), ('eye', 'sika1262-maume', 'mat̪an'), ('eye', 'p-cent2245-abvd', '*mata'), ('eye', 'tetu1246', 'mɑtɑn'), ('eye', 'alor1247-munas', 'mataʔ'), ('eye', 'keda1252-leuba', 'mato'), ('eye', 'lama1277-botun', 'mata'), ('eye', 'lama1277-lewuk', 'mata'), ('eye', 'tetu1245-vique', 'matan'), ('eye', 'idat1237', 'mata'), ('eye', 'alor1247-baran', 'mataŋ'), ('eye', 'lama1277-lerek', 'maˈta'), ('eye', 'lama1277-horow', 'matak'), ('eye', 'lama1277-belan', 'matak'), ('eye', 'lama1277-bama', 'mata'), ('eye', 'lama1277-dulhi', 'mata'), ('eye', 'lama1277-adona', 'matakɜt'), ('eye', 'alor1247-munas', 'mataŋ'), ('eye', 'koto1251', 'mata-f')}


Concept ‘bean’ is different between Alorese and Lamaholot.

 Alorese innovated the following forms:
 {('bean', 'alor1247-besar', 'kepa'), ('bean', 'alor1247-pandai', 'kepa'), ('bean', 'alor1247-munas', 'kepa')}
 One or more Lamaholot dialects innovated forms not found in Alorese:
 {('bean', 'kelo1247-hopte', 'ʔut'), ('bean', 'lama1277-lewoi', 'utaŋ'), ('bean', 'fata1247', 'ɔtɛ'), ('bean', 'lama1277-lerek', 'ˈutan'), ('bean', 'lama1277-kalik', 'uˈtan'), ('bean', 'maka1316', 'uta'), ('bean', 'kuii1253', 'utan'), ('bean', 'lama1277-adona', 'ˈʔutã'), ('bean', 'keda1252-leuba', "u'tan"), ('bean', 'kira1248', 'utan'), ('bean', 'adan1251-otvai', '’utaŋ'), ('bean', 'kabo1247', '(h)utu'), ('bean', 'adan1251-lawah', 'ʔutuŋ'), ('bean', 'lama1277-kalik', 'uˈta'), ('bean', 'rett1240', 'ʔutaŋ')}


Concept cooked_corn is attested only in Lamaholot


Concept ‘to_sew’ is different between Alorese and Lamaholot.

 Alorese innovated the following forms:
 {('to_sew', 'alor1247-munas', 'bulaŋ')}
 Alorese shares the following forms with other AN languanges, but not Lamaholot:
 {('to_sew', 'wers1238-taram', 'suai'), ('to_sew', 'koto1251', 'n-so:'), ('to_sew', 'tetu1245-suai', 'suku'), ('to_sew', 'kuii1253', 'serót'), ('to_sew', 'alor1247-pandai', 'horːu'), ('to_sew', 'laka1255', 'suʔu'), ('to_sew', 'mamb1306', 'sɔr'), ('to_sew', 'tetu1245-vique', 'suku'), ('to_sew', 'wers1238-taram', 'sor'), ('to_sew', 'idat1237', 'suʔu'), ('to_sew', 'sawi1256', 'surə'), ('to_sew', 'kula1280-lanto', '’sua'), ('to_sew', 'alor1247-besar', "hɔru 'apa"), ('to_sew', 'kema1243', 'sɔra'), ('to_sew', 'tuku1254', 'sɔr'), ('to_sew', 'wers1238-marit', "sɔr 'burkiŋ"), ('to_sew', 'tetu1246', 'suku')}
 One or more Lamaholot dialects innovated forms not found in Alorese:
 {('to_sew', 'lama1277-tanju', 'dau'), ('to_sew', 'p-mala1545-acd', '*tahiq'), ('to_sew', 'p-aust1307-abvd', '*taSiq'), ('to_sew', 'lama1277-lewoi', 'ˈdauŋ'), ('to_sew', 'p-mala1545-abvd', '*tahiq')}

  Just for clarity:
  There are also the following similarity classes shared between Alorese and LH.
  {('to_sew', 'lama1277-mulan', 'hau'), ('to_sew', 'alor1247-munas', 'haur'), ('to_sew', 'lama1277-baipi', 'hawu'), ('to_sew', 'lama1277-lewot', 'haur'), ('to_sew', 'lama1277-lewog', 'hawurã'), ('to_sew', 'lama1277-dulhi', 'hau'), ('to_sew', 'lama1277-belan', 'haur'), ('to_sew', 'alor1247-pandai', 'haur'), ('to_sew', 'lama1277-adona', 'ˈhɑu'), ('to_sew', 'lama1277-paina', 'hau'), ('to_sew', 'lama1277-lewom', 'hawu'), ('to_sew', 'lama1277-merde', 'haur'), ('to_sew', 'lama1277-ritae', 'hau'), ('to_sew', 'lama1277-watan', 'hau'), ('to_sew', 'lama1277-ileap', 'hau'), ('to_sew', 'lama1277-lamal', 'hau'), ('to_sew', 'lama1277-lamat', 'heu'), ('to_sew', 'lama1277-lewop', 'haur'), ('to_sew', 'keda1252', 'heuŋ'), ('to_sew', 'lama1277-horow', 'hau'), ('to_sew', 'lama1277-wuake', 'haur'), ('to_sew', 'keda1252-leuba', "hɛ'woŋ"), ('to_sew', 'lama1277-lewuk', 'saur'), ('to_sew', 'lama1277-lewok', 'saur'), ('to_sew', 'lama1277-lewoe', 'heu'), ('to_sew', 'lama1277-pukau', 'hau'), ('to_sew', 'lama1277-lamah', 'haur'), ('to_sew', 'lama1277-waiba', 'hau'), ('to_sew', 'lama1277-kiwan', 'hau'), ('to_sew', 'lama1277-minga', 'saur'), ('to_sew', 'lama1277-lerek', 'hauˈrəŋ'), ('to_sew', 'lama1277-bama', 'hãu'), ('to_sew', 'lama1277-imulo', 'saur'), ('to_sew', 'lama1277-lamak', 'hawu'), ('to_sew', 'lama1277-lewob', 'hau'), ('to_sew', 'lama1277-waiwa', 'hau'), ('to_sew', 'lama1277-botun', 'hau'), ('to_sew', 'alor1247-baran', 'haur'), ('to_sew', 'lama1277-kalik', 'saur')}


Concept ‘leaf’ is different between Alorese and Lamaholot.

 One or more Lamaholot dialects innovated forms not found in Alorese:
 {('leaf', 'lama1277-lewoi', 'ˈləpɑŋ'), ('leaf', 'lama1277-baipi', 'ləpa'), ('leaf', 'lama1277-lewob', 'ləpã'), ('leaf', 'keda1252-leuwa', 'lεpaʔ'), ('leaf', 'lama1277-lewog', 'lepaʔã'), ('leaf', 'lama1277-lewom', 'ləpã'), ('leaf', 'lama1277-ritae', 'ləpã')}

  Just for clarity:
  There are also the following similarity classes shared between Alorese and LH.
  {('leaf', 'lama1277-lamat', 'lolõ'), ('leaf', 'lama1277-lewoe', 'lolõ'), ('leaf', 'lama1277-kiwan', 'lolo'), ('leaf', 'lama1277-waiba', 'lolo'), ('leaf', 'lama1277-imulo', 'lolo'), ('leaf', 'lama1277-lamak', 'lolõ'), ('leaf', 'lama1277-lewot', 'lolo'), ('leaf', 'lama1277-tanju', 'lolo'), ('leaf', 'lama1277-lamal', 'lolo'), ('leaf', 'keda1252-leuba', 'lɔlɔn'), ('leaf', 'lama1277-dulhi', 'lolo'), ('leaf', 'lama1277-lerek', 'ˈlolon'), ('leaf', 'lama1277-belan', 'lolo'), ('leaf', 'lama1277-pukau', 'loloŋ'), ('leaf', 'lama1277-ileap', 'lolon'), ('leaf', 'keda1252', 'lolon'), ('leaf', 'lama1277-adona', 'ˈlolõ'), ('leaf', 'lama1277-minga', 'lolo'), ('leaf', 'alor1247-munas', "kaʤɔ 'lɔlɔŋ"), ('leaf', 'lama1277-botun', 'lolo'), ('leaf', 'lama1277-kalik', 'lɔˈlon'), ('leaf', 'lama1277-waiwa', 'lolo'), ('leaf', 'alor1247-baran', 'lɔlɔŋ'), ('leaf', 'lama1277-wuake', 'lolo'), ('leaf', 'lama1277-lewop', 'lolo'), ('leaf', 'lama1277-kalik', 'lolon'), ('leaf', 'lama1277-lewok', 'lolo'), ('leaf', 'lama1277-paina', 'lolon'), ('leaf', 'lama1277-lamah', 'lolõ'), ('leaf', 'alor1247-pandai', 'lɔlɔŋ'), ('leaf', 'lama1277-bama', 'lolõ'), ('leaf', 'lama1277-mulan', 'lolo'), ('leaf', 'keda1252-leuwa', 'lɔlɔn'), ('leaf', 'lama1277-horow', 'lolo'), ('leaf', 'lama1277-lewuk', 'lolon'), ('leaf', 'keda1252-leuwa', 'ai lɔlɔn'), ('leaf', 'alor1247-besar', 'lɔlɔŋ'), ('leaf', 'lama1277-watan', 'lolo'), ('leaf', 'lama1277-merde', 'lolõ')}


Concept ‘yellow’ is different between Alorese and Lamaholot.

 One or more Lamaholot dialects innovated forms not found in Alorese:
 {('yellow', 'buna1278-malia', 'kinul'), ('yellow', 'lama1277-baipi', 'kuniŋ'), ('yellow', 'tetu1245-vique', 'kinur'), ('yellow', 'p-cent2245-abvd', '*kunij'), ('yellow', 'p-mala1545-abvd', '*ma-kunij'), ('yellow', 'tetu1245-suai', 'kinur'), ('yellow', 'buna1278-bobon', 'khinul'), ('yellow', 'buna1278-suai', 'khinur'), ('yellow', 'tetu1246', 'kinur')}
 {('yellow', 'sika1262-hewa', 'heret'), ('yellow', 'lama1277-pukau', 'heret'), ('yellow', 'sika1262-tanai', 'herə-t'), ('yellow', 'sika1262-maume', 'heret̪')}

  Just for clarity:
  There are also the following similarity classes shared between Alorese and LH.
  {('yellow', 'alor1247-pandai', 'kumɔŋ'), ('yellow', 'lama1277-horow', 'kumha'), ('yellow', 'lama1277-tanju', 'kuma'), ('yellow', 'keda1252', 'uman'), ('yellow', 'lama1277-lerek', 'kumaˈhən'), ('yellow', 'lama1277-lewop', 'kənumahan'), ('yellow', 'lama1277-lewog', 'kumã'), ('yellow', 'keda1252-leuba', "ʔu'man"), ('yellow', 'alor1247-baran', 'kumɔ̃:'), ('yellow', 'lama1277-lewob', 'kumã'), ('yellow', 'lama1277-botun', 'kumha'), ('yellow', 'lama1277-waiba', 'kumã'), ('yellow', 'lama1277-kalik', 'kuˈmas'), ('yellow', 'lama1277-lamal', 'kumã'), ('yellow', 'lama1277-wuake', 'kuma'), ('yellow', 'lama1277-paina', 'kumahən'), ('yellow', 'lama1277-watan', 'kumã'), ('yellow', 'lama1277-lewuk', 'kumas'), ('yellow', 'laka1255', 'kɛ:n'), ('yellow', 'alor1247-besar', 'kumɔŋ'), ('yellow', 'lama1277-bama', 'kuma'), ('yellow', 'lama1277-adona', 'ˈkumhã'), ('yellow', 'lama1277-kalik', 'kumas'), ('yellow', 'lama1277-lewok', 'kumas'), ('yellow', 'alor1247-munas', 'kumɔŋ'), ('yellow', 'lama1277-dulhi', 'kuma'), ('yellow', 'lama1277-merde', 'kumhã'), ('yellow', 'lama1277-imulo', 'kumasən'), ('yellow', 'lama1277-lamak', 'kumha'), ('yellow', 'mamb1306', "gə'me:"), ('yellow', 'lama1277-lamat', 'uməhã'), ('yellow', 'lama1277-mulan', 'kumɤ'), ('yellow', 'lama1277-kiwan', 'kumha'), ('yellow', 'lama1277-lewot', 'kuma'), ('yellow', 'lama1277-lewom', 'kumã'), ('yellow', 'lama1277-ritae', 'kumã'), ('yellow', 'lama1277-minga', 'kumas'), ('yellow', 'lama1277-lamah', 'kuma'), ('yellow', 'lama1277-ileap', 'kuma'), ('yellow', 'lama1277-waiwa', 'kuma'), ('yellow', 'lama1277-lewoi', 'ˈkuma'), ('yellow', 'lama1277-lewoe', 'umɔ'), ('yellow', 'lama1277-belan', 'kuma')}


Concept ‘to_run’ is different between Alorese and Lamaholot.

 One or more Lamaholot dialects innovated forms not found in Alorese:
 {('to_run', 'lama1277-lerek', 'kar'), ('to_run', 'lama1277-kalik', 'ˈkari')}

  Just for clarity:
  There are also the following similarity classes shared between Alorese and LH.
  {('to_run', 'alor1247-pandai', "p'lae"), ('to_run', 'alor1247-munas', "p'laeŋ"), ('to_run', 'alor1247-besar', 'palae'), ('to_run', 'lama1277-adona', 'pɑˈlaʔe'), ('to_run', 'sika1262-hewa', 'plari'), ('to_run', 'lama1277-lewoi', 'pəˈlaʔɛ'), ('to_run', 'alor1247-baran', "pa'laɛ")}


Concept ‘rice_field’ is different between Alorese and Lamaholot.

 Alorese innovated the following forms:
 {('rice_field', 'alor1247-pandai', "ɛkaŋ  'buta")}
 Alorese shares the following forms with other AN languanges, but not Lamaholot:
 {('rice_field', 'kula1280-lanto', 'sawa'), ('rice_field', 'keda1252-leuba', 'sawa'), ('rice_field', 'sawi1256', 'sawa'), ('rice_field', 'kula1280-lanto', '’sawa'), ('rice_field', 'alor1247-munas', "sawa 'alɛr"), ('rice_field', 'alor1247-pandai', 'sawa'), ('rice_field', 'abui1241-ulaga', 'sawa'), ('rice_field', 'alor1247-besar', 'sawa'), ('rice_field', 'abui1241-takal', 'sä.wä')}
 One or more Lamaholot dialects innovated forms not found in Alorese:
 {('rice_field', 'kaer1234', "bu'tax"), ('rice_field', 'lama1277-lewoi', 'ˈpɛta'), ('rice_field', 'pura1258', 'buta'), ('rice_field', 'tetu1245-suai', 'kintal'), ('rice_field', 'tetu1245-vique', 'kintal')}
 {('rice_field', 'keda1252-leuba', 'lumɑr'), ('rice_field', 'sika1262-hewa', 'uma'), ('rice_field', 'lama1277-adona', 'mãː')}


Concept ‘waist’ is different between Alorese and Lamaholot.

 One or more Lamaholot dialects innovated forms not found in Alorese:
 {('waist', 'lama1277-adona', 'kɜˈlɜɡɜrɜt')}
 {('waist', 'lama1277-lewoi', 'ˈwekɔl')}

  Just for clarity:
  There are also the following similarity classes shared between Alorese and LH.
  {('waist', 'lama1277-lerek', 'ˈale'), ('waist', 'alor1247-pandai', 'alɛŋ'), ('waist', 'alor1247-munas', 'alɛʔ'), ('waist', 'alor1247-besar', 'alɛ̃'), ('waist', 'lama1277-kalik', 'aˈle'), ('waist', 'alor1247-munas', 'alɛŋ'), ('waist', 'keda1252-leuba', "a'lɛn")}


Concept ‘finger’ is different between Alorese and Lamaholot.

 Alorese innovated the following forms:
 {('finger', 'alor1247-besar', "kalu'makaŋ")}
 One or more Lamaholot dialects innovated forms not found in Alorese:
 {('finger', 'sika1262-hewa', 'kikir'), ('finger', 'lama1277-lerek', 'kiˈkil'), ('finger', 'lama1277-kalik', 'kiˈkil')}

  Just for clarity:
  There are also the following similarity classes shared between Alorese and LH.
  {('finger', 'alor1247-pandai', 'limaŋ numak'), ('finger', 'alor1247-munas', 'numak'), ('finger', 'lama1277-adona', 'ˈlimakɜt ˈʔaːnʔa'), ('finger', 'lama1277-lewoi', 'limaŋ ˈanaŋ')}


Concept ‘sago’ is different between Alorese and Lamaholot.

 Alorese innovated the following forms:
 {('sago', 'alor1247-munas', "mukɔ 'huaʔ")}
 Alorese shares the following forms with other AN languanges, but not Lamaholot:
 {('sago', 'abui1241-takal', 'sä.gu'), ('sago', 'alor1247-pandai', 'sagu'), ('sago', 'keda1252-leuba', 'saɡu'), ('sago', 'alor1247-munas', 'sagu'), ('sago', 'alor1247-besar', 'sagu')}
 One or more Lamaholot dialects innovated forms not found in Alorese:
 {('sago', 'lama1277-lewoi', 'ˈkɛbɔ')}


Concept ‘to_plant_rice’ is different between Alorese and Lamaholot.

 One or more Lamaholot dialects innovated forms not found in Alorese:
 {('to_plant_rice', 'lama1277-adona', 'ˈtubak')}

  Just for clarity:
  There are also the following similarity classes shared between Alorese and LH.
  {('to_plant_rice', 'alor1247-besar', "mula 'pari"), ('to_plant_rice', 'alor1247-pandai', "mula 'parɛ"), ('to_plant_rice', 'lama1277-lewoi', 'mula ˈtahaʔɑŋ'), ('to_plant_rice', 'lama1277-kalik', 'mul (ˈlisɔ)'), ('to_plant_rice', 'lama1277-lerek', 'ˈmula kəˈluok'), ('to_plant_rice', 'lama1277-adona', 'ˈmula'), ('to_plant_rice', 'alor1247-munas', "mula 'pare")}


Concept ‘ritual_mound’ is different between Alorese and Lamaholot.

 Alorese innovated the following forms:
 {('ritual_mound', 'alor1247-pandai', "wato 'ua 'mau 'uliŋ")}
 {('ritual_mound', 'alor1247-pandai', "wato p'lawak")}
 {('ritual_mound', 'alor1247-munas', 'dɔru'), ('ritual_mound', 'adan1251-otvai', '’dɔr'), ('ritual_mound', 'rett1240', 'dɔru'), ('ritual_mound', 'kaer1234', 'dor'), ('ritual_mound', 'alor1247-besar', 'dolu'), ('ritual_mound', 'kelo1247-hopte', 'dur'), ('ritual_mound', 'pura1258', 'doru')}
 Alorese shares the following forms with TAP languages:
 {('ritual_mound', 'alor1247-pandai', "wato 'ua 'mau 'uliŋ")}
 {('ritual_mound', 'alor1247-pandai', "wato p'lawak")}
 {('ritual_mound', 'alor1247-munas', 'dɔru'), ('ritual_mound', 'adan1251-otvai', '’dɔr'), ('ritual_mound', 'rett1240', 'dɔru'), ('ritual_mound', 'kaer1234', 'dor'), ('ritual_mound', 'alor1247-besar', 'dolu'), ('ritual_mound', 'kelo1247-hopte', 'dur'), ('ritual_mound', 'pura1258', 'doru')}
 One or more Lamaholot dialects innovated forms not found in Alorese:
 {('ritual_mound', 'lama1277-adona', 'ˈnubo ˈnara'), ('ritual_mound', 'lama1277-lerek', 'ˈnobeŋ')}
 {('ritual_mound', 'lama1277-lewoi', 'ˈnuba')}


Concept ‘louse_eggs’ is different between Alorese and Lamaholot.

 One or more Lamaholot dialects innovated forms not found in Alorese:
 {('louse_eggs', 'lama1277-kalik', 'ˈkutu kiˈan'), ('louse_eggs', 'keda1252-leuba', "ʔutu 'ʔihɛn"), ('louse_eggs', 'lama1277-lerek', 'kut kiˈan')}
 {('louse_eggs', 'lama1277-lewoi', 'kuto ˈtəluŋ'), ('louse_eggs', 'tetu1245-vique', 'utu tolun'), ('louse_eggs', 'tetu1245-suai', 'utu tolun'), ('louse_eggs', 'sika1262-hewa', 'ʔutu telon')}

  Just for clarity:
  There are also the following similarity classes shared between Alorese and LH.
  {('louse_eggs', 'lama1277-adona', 'kɜˈlɛhɛ̃'), ('louse_eggs', 'alor1247-munas', "k'lɛhɛŋ"), ('louse_eggs', 'alor1247-besar', "kuto ka'lehã"), ('louse_eggs', 'alor1247-pandai', "kuto k'lɛhɛŋ ")}


Concept ‘broom’ is different between Alorese and Lamaholot.

 One or more Lamaholot dialects innovated forms not found in Alorese:
 {('broom', 'tetu1245-suai', 'kna:r'), ('broom', 'tetu1245-vique', 'kna:r'), ('broom', 'lama1277-kalik', 'knoˈrɔt')}
 {('broom', 'lama1277-lewoi', 'kəˈnuru')}

  Just for clarity:
  There are also the following similarity classes shared between Alorese and LH.
  {('broom', 'alor1247-munas', 'namɔ'), ('broom', 'lama1277-lerek', 'bəˈnamur'), ('broom', 'alor1247-pandai', 'namo'), ('broom', 'alor1247-besar', 'namɔ'), ('broom', 'lama1277-adona', 'ˈnamu')}


Concept ‘teeth’ is different between Alorese and Lamaholot.

 Alorese innovated the following forms:
 {('teeth', 'alor1247-baran', 'ʔulɔŋ'), ('teeth', 'alor1247-pandai', 'ulɔŋ'), ('teeth', 'alor1247-munas', 'ulɔʔ'), ('teeth', 'alor1247-besar', 'ulɔŋ'), ('teeth', 'alor1247-munas', 'ulɔŋ')}
 One or more Lamaholot dialects innovated forms not found in Alorese:
 {('teeth', 'lama1277-lewot', 'kiakha'), ('teeth', 'lama1277-lewop', 'kiak'), ('teeth', 'lama1277-imulo', 'kiakəs')}
 {('teeth', 'lama1277-lamah', 'ipəkə'), ('teeth', 'lama1277-ileap', 'ipək'), ('teeth', 'lama1277-lewom', 'ipə'), ('teeth', 'lama1277-bama', 'ipə'), ('teeth', 'lama1277-ritae', 'ipə'), ('teeth', 'lama1277-lewoi', 'ˈipəʔ'), ('teeth', 'lama1277-pukau', 'ipək'), ('teeth', 'lama1277-botun', 'ipə'), ('teeth', 'lama1277-tanju', 'ipə'), ('teeth', 'lama1277-kalik', 'pai'), ('teeth', 'lama1277-belan', 'ipək'), ('teeth', 'lama1277-mulan', 'ipə'), ('teeth', 'lama1277-minga', 'ipã'), ('teeth', 'lama1277-lewoe', 'ihpe'), ('teeth', 'lama1277-lamak', 'ipã'), ('teeth', 'lama1277-horow', 'ipək'), ('teeth', 'lama1277-waiba', 'ipə'), ('teeth', 'lama1277-lewok', 'paiga'), ('teeth', 'lama1277-lerek', 'pəi'), ('teeth', 'lama1277-wuake', 'ipẽ'), ('teeth', 'lama1277-lamat', 'ipe'), ('teeth', 'lama1277-merde', 'ipək'), ('teeth', 'lama1277-kiwan', 'ipək'), ('teeth', 'lama1277-lewuk', 'paisa'), ('teeth', 'lama1277-baipi', 'ipət'), ('teeth', 'lama1277-lewog', 'ipə'), ('teeth', 'lama1277-paina', 'paig'), ('teeth', 'lama1277-kalik', 'paˈi'), ('teeth', 'lama1277-watan', 'ipʔã'), ('teeth', 'lama1277-lamal', 'ipã'), ('teeth', 'lama1277-lewob', 'ipə'), ('teeth', 'lama1277-waiwa', 'ipə'), ('teeth', 'lama1277-dulhi', 'ipɤ (ɤ)'), ('teeth', 'lama1277-adona', 'ˈipɜʔ')}


Concept ‘adultery’ is different between Alorese and Lamaholot.

 Alorese innovated the following forms:
 {('adultery', 'pura1258', 'buha'), ('adultery', 'teiw1235', 'bu:s'), ('adultery', 'alor1247-besar', "buha ma'leku"), ('adultery', 'rett1240', 'buha'), ('adultery', 'kaer1234', 'bus'), ('adultery', 'alor1247-munas', 'buha')}
 {('adultery', 'alor1247-pandai', "laka 'gawe"), ('adultery', 'alor1247-pandai', "gawe 'laka")}
 Alorese shares the following forms with TAP languages:
 {('adultery', 'pura1258', 'buha'), ('adultery', 'teiw1235', 'bu:s'), ('adultery', 'alor1247-besar', "buha ma'leku"), ('adultery', 'rett1240', 'buha'), ('adultery', 'kaer1234', 'bus'), ('adultery', 'alor1247-munas', 'buha')}
 {('adultery', 'alor1247-pandai', "laka 'gawe"), ('adultery', 'alor1247-pandai', "gawe 'laka")}
 One or more Lamaholot dialects innovated forms not found in Alorese:
 {('adultery', 'lama1277-adona', 'ˈbəluwo')}
 {('adultery', 'lama1277-lewoi', 'ˈnalana')}
 {('adultery', 'lama1277-lerek', 'ˈmitə ˈvakim')}


Concept ‘penis’ is different between Alorese and Lamaholot.

 One or more Lamaholot dialects innovated forms not found in Alorese:
 {('penis', 'lama1277-kalik', 'kɔˈlɔŋ')}

  Just for clarity:
  There are also the following similarity classes shared between Alorese and LH.
  {('penis', 'tetu1245-suai', "lasa'kain"), ('penis', 'tetu1245-vique', "lasa'kain"), ('penis', 'alor1247-besar', "la'hakaŋ"), ('penis', 'alor1247-pandai', 'lahak'), ('penis', 'lama1277-lewoi', 'ˈlaha')}
  {('penis', 'lama1277-lerek', 'uˈti'), ('penis', 'lama1277-adona', 'ˈute'), ('penis', 'p-mala1545-acd', '*qutin'), ('penis', 'alor1247-munas', 'utiŋ'), ('penis', 'sika1262-hewa', 'uti'), ('penis', 'keda1252-leuba', "wu'ti"), ('penis', 'lama1277-kalik', 'ˈuti'), ('penis', 'koto1251', 'uti-f')}


Concept ‘fowl’ is different between Alorese and Lamaholot.

 Alorese innovated the following forms:
 {('fowl', 'alor1247-pandai', "apa mə'nakaŋ")}
 {('fowl', 'alor1247-munas', "wuluk 'gapal")}
 One or more Lamaholot dialects innovated forms not found in Alorese:
 {('fowl', 'buna1278-bobon', 'hɔs'), ('fowl', 'lama1277-adona', 'ˈʔɜˈwã'), ('fowl', 'buna1278-suai', 'hɔs')}


Concept millet is attested only in Lamaholot


Concept ‘fruit’ is different between Alorese and Lamaholot.

 Alorese innovated the following forms:
 {('fruit', 'alor1247-besar', "i'hikiŋ"), ('fruit', 'alor1247-munas', "i'hik"), ('fruit', 'alor1247-pandai', 'ihik')}
 {('fruit', 'alor1247-baran', 'kuluŋ')}

  Just for clarity:
  There are also the following similarity classes shared between Alorese and LH.
  {('fruit', 'lama1277-waiba', 'wuã'), ('fruit', 'lama1277-lewob', 'wuã'), ('fruit', 'lama1277-lewot', 'uak'), ('fruit', 'lama1277-lerek', 'ˈvuak'), ('fruit', 'tetu1245-vique', 'Ɂaifuaŋ'), ('fruit', 'tetu1245-suai', 'Ɂaifuaŋ'), ('fruit', 'lama1277-lamah', 'wuã'), ('fruit', 'sika1262-maume', 'vuan'), ('fruit', 'lama1277-lewog', 'wuʔã'), ('fruit', 'lama1277-waiwa', 'wuã'), ('fruit', 'tetu1246', 'fuɑn'), ('fruit', 'lama1277-bama', 'wuã'), ('fruit', 'lama1277-lewuk', 'wuak'), ('fruit', 'lama1277-pukau', 'wuʔã'), ('fruit', 'lama1277-kalik', 'vuˈak'), ('fruit', 'p-cent2245-abvd', '*buaq'), ('fruit', 'lama1277-horow', 'wuã'), ('fruit', 'p-mala1545-abvd', '*buaq'), ('fruit', 'lama1277-wuake', 'wuã'), ('fruit', 'sika1262-hewa', 'ʋuan'), ('fruit', 'lama1277-lamat', 'wuʔã'), ('fruit', 'lama1277-lewom', 'wuã'), ('fruit', 'lama1277-ritae', 'wuã'), ('fruit', 'lama1277-dulhi', 'wuã'), ('fruit', 'lama1277-lewoe', 'wuʔɔ̃'), ('fruit', 'lama1277-baipi', 'wuã'), ('fruit', 'p-mala1545-acd', '*buaq'), ('fruit', 'lama1277-paina', 'wuakən'), ('fruit', 'p-aust1307-abvd', '*buaq'), ('fruit', 'lama1277-minga', 'fuak'), ('fruit', 'keda1252-leuba', 'uan'), ('fruit', 'lama1277-adona', 'kajo ˈwuã'), ('fruit', 'keda1252-leuwa', 'u̘an'), ('fruit', 'sika1262-tanai', 'βua-ŋ'), ('fruit', 'lama1277-lamak', 'wuã'), ('fruit', 'lama1277-tanju', 'wua'), ('fruit', 'koto1251', 'fua-f'), ('fruit', 'lama1277-lewok', 'wuak'), ('fruit', 'lama1277-watan', 'wuã'), ('fruit', 'lama1277-kalik', 'wuak'), ('fruit', 'alor1247-pandai', 'punak'), ('fruit', 'lama1277-lamal', 'fuã'), ('fruit', 'lama1277-ileap', 'wuan'), ('fruit', 'keda1252', 'wuan'), ('fruit', 'lama1277-merde', 'wuã'), ('fruit', 'lama1277-lewop', 'fuak'), ('fruit', 'sika1262-hewa', 'wuan'), ('fruit', 'lama1277-imulo', 'fuak'), ('fruit', 'lama1277-botun', 'wuã'), ('fruit', 'lama1277-mulan', 'wuɤ'), ('fruit', 'lama1277-kiwan', 'wuã'), ('fruit', 'lama1277-belan', 'wua'), ('fruit', 'lama1277-lewoi', 'ˈwuaŋ')}


Concept ‘canoe’ is different between Alorese and Lamaholot.

 One or more Lamaholot dialects innovated forms not found in Alorese:
 {('canoe', 'atim1239', 'pelaŋ'), ('canoe', 'buna1278-suai', 'bɛlɔ'), ('canoe', 'lama1277-lerek', 'bəˈruaŋ'), ('canoe', 'buna1278-bobon', 'bɛlɔ'), ('canoe', 'abui1241-petle', 'pe.laŋ'), ('canoe', 'buna1278-malia', 'bɛrɔ'), ('canoe', 'abui1241-takal', 'pe̞.läŋ'), ('canoe', 'mamb1306', 'bɛro'), ('canoe', 'abui1241-takal', 'pe̞.lã'), ('canoe', 'laka1255', 'bɛrɔ'), ('canoe', 'kema1243', 'bɛrɔ')}

  Just for clarity:
  There are also the following similarity classes shared between Alorese and LH.
  {('canoe', 'lama1277-adona', 'tɛna'), ('canoe', 'lama1277-lewoi', 'ˈtɛna'), ('canoe', 'alor1247-munas', 'tɛna'), ('canoe', 'keda1252-leuba', 'tɛnɛ'), ('canoe', 'sika1262-hewa', 'tena'), ('canoe', 'alor1247-besar', 'tɛna'), ('canoe', 'lama1277-lerek', 'ˈtenar'), ('canoe', 'lama1277-kalik', 'teˈnar'), ('canoe', 'alor1247-pandai', 'tena'), ('canoe', 'lama1277-kalik', 'tena'), ('canoe', 'alor1247-baran', 'tɛna')}


Concept ‘nostril’ is different between Alorese and Lamaholot.

 One or more Lamaholot dialects innovated forms not found in Alorese:
 {('nostril', 'lama1277-adona', 'iruˈnɜt kɜˈlɜŋũ')}
 {('nostril', 'lama1277-lerek', 'nidʒ͡u roˈboŋ')}

  Just for clarity:
  There are also the following similarity classes shared between Alorese and LH.
  {('nostril', 'alor1247-besar', "niruŋ faŋ'gɔkɔŋ"), ('nostril', 'alor1247-pandai', 'iruŋ'), ('nostril', 'lama1277-kalik', 'niˈʤu uˈmaŋ'), ('nostril', 'alor1247-munas', "iru 'wanɔʔ"), ('nostril', 'sika1262-hewa', 'iru boaŋ'), ('nostril', 'alor1247-munas', "iruŋ 'waŋɔʔ"), ('nostril', 'lama1277-lewoi', 'iruŋ uˈmaŋ'), ('nostril', 'alor1247-pandai', "niruŋ 'waŋgɔ")}


Concept ‘to_fly’ is different between Alorese and Lamaholot.

 One or more Lamaholot dialects innovated forms not found in Alorese:
 {('to_fly', 'lama1277-lewoe', 'bɔʔɔ̃')}

  Just for clarity:
  There are also the following similarity classes shared between Alorese and LH.
  {('to_fly', 'lama1277-wuake', 'bəka'), ('to_fly', 'lama1277-botun', 'bəka'), ('to_fly', 'lama1277-lamat', 'baʔã'), ('to_fly', 'alor1247-munas', "bə'kaŋ"), ('to_fly', 'lama1277-waiba', 'bəka'), ('to_fly', 'lama1277-minga', 'bəkɤ'), ('to_fly', 'lama1277-lerek', 'ˈbəkə'), ('to_fly', 'lama1277-lamah', 'bəka'), ('to_fly', 'lama1277-lamal', 'bəka'), ('to_fly', 'lama1277-lewom', 'bəka'), ('to_fly', 'lama1277-ritae', 'bəka'), ('to_fly', 'lama1277-kalik', 'bəˈka'), ('to_fly', 'lama1277-horow', 'bəka'), ('to_fly', 'lama1277-ileap', 'bəka'), ('to_fly', 'lama1277-lewok', 'bəka'), ('to_fly', 'alor1247-besar', 'baka'), ('to_fly', 'alor1247-baran', 'bak:aŋ'), ('to_fly', 'lama1277-bama', 'bəka'), ('to_fly', 'lama1277-adona', 'ˈbɜka'), ('to_fly', 'lama1277-watan', 'bəka'), ('to_fly', 'lama1277-lewop', 'bəkə'), ('to_fly', 'lama1277-waiwa', 'bəka'), ('to_fly', 'lama1277-lewog', 'bəka'), ('to_fly', 'keda1252-leuba', 'baʔa'), ('to_fly', 'lama1277-merde', 'bəka'), ('to_fly', 'lama1277-kiwan', 'bəka'), ('to_fly', 'lama1277-lewuk', 'bəka'), ('to_fly', 'lama1277-baipi', 'bəka'), ('to_fly', 'lama1277-dulhi', 'bəka'), ('to_fly', 'lama1277-mulan', 'bəka'), ('to_fly', 'keda1252', 'baʔa'), ('to_fly', 'lama1277-lewot', 'bək'), ('to_fly', 'lama1277-belan', 'bəka'), ('to_fly', 'lama1277-paina', 'bəka'), ('to_fly', 'lama1277-lewob', 'bəka'), ('to_fly', 'lama1277-pukau', 'bəka'), ('to_fly', 'lama1277-kalik', 'bəka'), ('to_fly', 'lama1277-lewoi', 'ˈbəka'), ('to_fly', 'lama1277-imulo', 'bək'), ('to_fly', 'alor1247-pandai', 'bəkːaŋ'), ('to_fly', 'lama1277-lamak', 'bəka'), ('to_fly', 'lama1277-tanju', 'bəka')}


Concept ‘to_float’ is different between Alorese and Lamaholot.

 One or more Lamaholot dialects innovated forms not found in Alorese:
 {('to_float', 'keda1252-leuba', 'baɔ'), ('to_float', 'lama1277-lewoi', 'ˈbao'), ('to_float', 'lama1277-horow', 'bao'), ('to_float', 'lama1277-lamak', 'bao'), ('to_float', 'lama1277-lerek', 'ˈbao'), ('to_float', 'lama1277-botun', 'bao'), ('to_float', 'lama1277-lewom', 'bao'), ('to_float', 'lama1277-belan', 'bao'), ('to_float', 'lama1277-ritae', 'bao'), ('to_float', 'lama1277-merde', 'bao'), ('to_float', 'lama1277-lewog', 'bao'), ('to_float', 'lama1277-minga', 'bao'), ('to_float', 'lama1277-lewuk', 'bao'), ('to_float', 'lama1277-watan', 'bao'), ('to_float', 'lama1277-lewop', 'baoŋa'), ('to_float', 'lama1277-waiba', 'bao'), ('to_float', 'lama1277-lamah', 'baʔo'), ('to_float', 'lama1277-lewob', 'bao'), ('to_float', 'lama1277-mulan', 'bao'), ('to_float', 'keda1252-leuwa', 'baɔ tata'), ('to_float', 'lama1277-lamal', 'bao'), ('to_float', 'lama1277-adona', 'ˈbaɔʔ'), ('to_float', 'lama1277-lewoe', 'bɔo'), ('to_float', 'lama1277-ileap', 'bao'), ('to_float', 'keda1252', 'bao'), ('to_float', 'lama1277-lamat', 'bao'), ('to_float', 'lama1277-wuake', 'bao'), ('to_float', 'lama1277-waiwa', 'bao'), ('to_float', 'lama1277-paina', 'bao'), ('to_float', 'lama1277-bama', 'bao'), ('to_float', 'lama1277-tanju', 'bao'), ('to_float', 'lama1277-kiwan', 'bao'), ('to_float', 'sika1262-tanai', 'baβak'), ('to_float', 'lama1277-dulhi', 'bao'), ('to_float', 'lama1277-baipi', 'bao'), ('to_float', 'lama1277-pukau', 'bao'), ('to_float', 'lama1277-imulo', 'baoŋa'), ('to_float', 'lama1277-lewot', 'baoŋa')}
 {('to_float', 'pura1258', 'tediŋ'), ('to_float', 'blag1240-tuntu', 'tediŋ'), ('to_float', 'kelo1247-hopte', 'tɛd'), ('to_float', 'blag1240-nule', 'tediŋ'), ('to_float', 'adan1251-lawah', 'tɛdɛʔ'), ('to_float', 'sarr1247-nule', 'tedis'), ('to_float', 'blag1240-kulij', 'tediŋ'), ('to_float', 'dein1238', 'tiadis'), ('to_float', 'rett1240', 'tiada'), ('to_float', 'baka1276', 'tediŋ'), ('to_float', 'kelo1247-hopte', "tə'tɛ:d"), ('to_float', 'adan1251-otvai', '’tɛd'), ('to_float', 'rett1240', 'tiadaŋ'), ('to_float', 'lama1277-kalik', 'ˈteda'), ('to_float', 'blag1240-bama', 'tediŋ'), ('to_float', 'abui1241-fuime', 'ije fauiti'), ('to_float', 'lama1277-kalik', 'teda'), ('to_float', 'sarr1247-adiab', 'tedis'), ('to_float', 'blag1240-warsa', 'tediŋ'), ('to_float', 'hama1240', 'tɛd'), ('to_float', 'atim1239', 'tauiti'), ('to_float', 'kelo1247-bring', 'tətɛd')}

  Just for clarity:
  There are also the following similarity classes shared between Alorese and LH.
  {('to_float', 'alor1247-besar', 'nepi'), ('to_float', 'lama1277-lewok', 'nebon'), ('to_float', 'alor1247-pandai', 'nɛpik'), ('to_float', 'alor1247-baran', 'nɛpi'), ('to_float', 'sika1262-hewa', 'nebon'), ('to_float', 'alor1247-munas', 'nepik'), ('to_float', 'p-mala1545-acd', '*apuŋ')}


Concept ‘monitor_lizard’ is different between Alorese and Lamaholot.

 Alorese innovated the following forms:
 {('monitor_lizard', 'kira1248', 'wurosi'), ('monitor_lizard', 'baka1276', 'rihi'), ('monitor_lizard', 'kelo1247-hopte', "wə'rih"), ('monitor_lizard', 'blag1240-warsa', 'iris'), ('monitor_lizard', 'sarr1247-nule', 'ris'), ('monitor_lizard', 'kuii1253', 'ros'), ('monitor_lizard', 'blag1240-bama', 'iris'), ('monitor_lizard', 'lamm1241-westp', 'hid:is'), ('monitor_lizard', 'sarr1247-adiab', 'jiris'), ('monitor_lizard', 'kaer1234', "tɛ'rɛs"), ('monitor_lizard', 'blag1240-nule', 'ri'), ('monitor_lizard', 'alor1247-besar', 'reha'), ('monitor_lizard', 'kaer1234', "i'ris"), ('monitor_lizard', 'pura1258', 'ari'), ('monitor_lizard', 'dein1238', "je'ris"), ('monitor_lizard', 'blag1240-tuntu', 'iris'), ('monitor_lizard', 'teiw1235', 'ris'), ('monitor_lizard', 'blag1240-kulij', 'rihi')}
 {('monitor_lizard', 'alor1247-munas', "tɛto 'damar"), ('monitor_lizard', 'alor1247-pandai', "təto'damar")}
 Alorese shares the following forms with TAP languages:
 {('monitor_lizard', 'kira1248', 'wurosi'), ('monitor_lizard', 'baka1276', 'rihi'), ('monitor_lizard', 'kelo1247-hopte', "wə'rih"), ('monitor_lizard', 'blag1240-warsa', 'iris'), ('monitor_lizard', 'sarr1247-nule', 'ris'), ('monitor_lizard', 'kuii1253', 'ros'), ('monitor_lizard', 'blag1240-bama', 'iris'), ('monitor_lizard', 'lamm1241-westp', 'hid:is'), ('monitor_lizard', 'sarr1247-adiab', 'jiris'), ('monitor_lizard', 'kaer1234', "tɛ'rɛs"), ('monitor_lizard', 'blag1240-nule', 'ri'), ('monitor_lizard', 'alor1247-besar', 'reha'), ('monitor_lizard', 'kaer1234', "i'ris"), ('monitor_lizard', 'pura1258', 'ari'), ('monitor_lizard', 'dein1238', "je'ris"), ('monitor_lizard', 'blag1240-tuntu', 'iris'), ('monitor_lizard', 'teiw1235', 'ris'), ('monitor_lizard', 'blag1240-kulij', 'rihi')}
 {('monitor_lizard', 'alor1247-munas', "tɛto 'damar"), ('monitor_lizard', 'alor1247-pandai', "təto'damar")}
 One or more Lamaholot dialects innovated forms not found in Alorese:
 {('monitor_lizard', 'lama1277-kalik', 'bəˈpap'), ('monitor_lizard', 'lama1277-lerek', 'ɡəˈpap')}
 {('monitor_lizard', 'lama1277-adona', 'ˈʔoteʔ'), ('monitor_lizard', 'lama1277-lewoi', 'ˈote'), ('monitor_lizard', 'sika1262-hewa', 'oti')}


Concept ‘to_teach’ is different between Alorese and Lamaholot.

 One or more Lamaholot dialects innovated forms not found in Alorese:
 {('to_teach', 'lama1277-lerek', 'ˈnuat ˈnokan')}

  Just for clarity:
  There are also the following similarity classes shared between Alorese and LH.
  {('to_teach', 'alor1247-munas', "nuaŋ 'apa"), ('to_teach', 'lama1277-adona', 'ˈnuã'), ('to_teach', 'alor1247-pandai', 'nuaŋ')}
  {('to_teach', 'alor1247-besar', 'aʤara'), ('to_teach', 'lama1277-lewoi', 'ˈaʤɑr'), ('to_teach', 'sika1262-hewa', 'meŋajar'), ('to_teach', 'keda1252-leuba', 'ʔadʒar'), ('to_teach', 'teiw1235', 'aɟar'), ('to_teach', 'lama1277-kalik', 'ŋaˈʤar')}


Concept ‘to_dwell’ is different between Alorese and Lamaholot.

 Alorese innovated the following forms:
 {('to_dwell', 'alor1247-besar', 'tɔbɔ'), ('to_dwell', 'alor1247-munas', 'tɔbɔ'), ('to_dwell', 'alor1247-pandai', 'tɔbɔ')}
 One or more Lamaholot dialects innovated forms not found in Alorese:
 {('to_dwell', 'lama1277-kalik', "-'ia"), ('to_dwell', 'lama1277-lerek', 'ˈmian')}
 {('to_dwell', 'lama1277-lewoi', 'ˈtei')}
 {('to_dwell', 'lama1277-adona', 'ˈtawãnɜt')}


Concept ‘sour’ is different between Alorese and Lamaholot.

 One or more Lamaholot dialects innovated forms not found in Alorese:
 {('sour', 'lama1277-lerek', 'ˈtobil')}

  Just for clarity:
  There are also the following similarity classes shared between Alorese and LH.
  {('sour', 'lama1277-adona', 'ˈɡilo'), ('sour', 'lama1277-kalik', 'kniˈluk'), ('sour', 'keda1252-leuba', "ki'ru"), ('sour', 'lama1277-lewoi', 'ˈɡilo'), ('sour', 'sika1262-hewa', 'niluk'), ('sour', 'alor1247-munas', 'giloŋ'), ('sour', 'alor1247-besar', 'gilo'), ('sour', 'alor1247-pandai', 'gilo'), ('sour', 'alor1247-pandai', 'gilɔŋ')}


Concept ‘tinea’ is different between Alorese and Lamaholot.

 One or more Lamaholot dialects innovated forms not found in Alorese:
 {('tinea', 'lama1277-lerek', 'bəroŋ')}
 {('tinea', 'lama1277-kalik', 'kraˈposa')}

  Just for clarity:
  There are also the following similarity classes shared between Alorese and LH.
  {('tinea', 'sika1262-hewa', 'na:uʔ'), ('tinea', 'sarr1247-adiab', "mə'nas"), ('tinea', 'alor1247-pandai', "m'nao"), ('tinea', 'sarr1247-nule', 'n-ənaj'), ('tinea', 'blag1240-nule', "mə'naw"), ('tinea', 'teiw1235', "ma'nai"), ('tinea', 'alor1247-munas', 'nao'), ('tinea', 'rett1240', 'manau'), ('tinea', 'alor1247-besar', 'manau'), ('tinea', 'dein1238', 'mna:s'), ('tinea', 'lama1277-adona', 'mɜˈnaɔ'), ('tinea', 'lama1277-lewoi', 'ˈmau'), ('tinea', 'alor1247-pandai', 'nao'), ('tinea', 'nede1245', 'manu')}


Concept ‘salty’ is different between Alorese and Lamaholot.

 One or more Lamaholot dialects innovated forms not found in Alorese:
 {('salty', 'lama1277-kalik', 'pənɛˈʤʊk')}
 {('salty', 'lama1277-lerek', 'mədʒ͡uˈkən')}
 {('salty', 'lama1277-lerek', 'pɔi-pɔi')}

  Just for clarity:
  There are also the following similarity classes shared between Alorese and LH.
  {('salty', 'alor1247-besar', 'parɔ'), ('salty', 'alor1247-munas', "pə'rːrɔ"), ('salty', 'keda1252-leuba', "pɛ'ju"), ('salty', 'lama1277-adona', 'ˈpɜro'), ('salty', 'lama1277-lewoi', 'ˈpəro'), ('salty', 'alor1247-pandai', 'parːɔ'), ('salty', 'sika1262-hewa', 'bəru')}


Concept ‘older_brother’ is different between Alorese and Lamaholot.

 Alorese shares the following forms with other AN languanges, but not Lamaholot:
 {('older_brother', 'alor1247-pandai', "beiŋ k'lake"), ('older_brother', 'alor1247-besar', "kakaŋ ka'lake"), ('older_brother', 'alor1247-pandai', 'tata'), ('older_brother', 'koto1251', 'tata-f'), ('older_brother', 'alor1247-munas', "tata k'lake")}
 One or more Lamaholot dialects innovated forms not found in Alorese:
 {('older_brother', 'lama1277-adona', 'kaka'), ('older_brother', 'maka1316', 'kaka'), ('older_brother', 'fata1247', 'kaka')}
 {('older_brother', 'lama1277-adona', 'ʔariʔ')}
 {('older_brother', 'lama1277-lewoi', 'aʔe'), ('older_brother', 'keda1252-leuba', 'aʔe')}


Concept ‘penalty’ is different between Alorese and Lamaholot.

 Alorese innovated the following forms:
 {('penalty', 'alor1247-pandai', 'pate '), ('penalty', 'alor1247-munas', "pate 'hala"), ('penalty', 'alor1247-besar', "pate 'hala")}

  Just for clarity:
  There are also the following similarity classes shared between Alorese and LH.
  {('penalty', 'lama1277-lewoi', 'ˈdɛnda'), ('penalty', 'teiw1235', "den'daɁ"), ('penalty', 'lama1277-adona', 'ˈnɜda'), ('penalty', 'alor1247-pandai', "dan'da")}


Concept ‘to_learn’ is different between Alorese and Lamaholot.

 Alorese innovated the following forms:
 {('to_learn', 'alor1247-pandai', "ukur 'iŋa")}
 {('to_learn', 'alor1247-munas', "guru 'apa")}
 One or more Lamaholot dialects innovated forms not found in Alorese:
 {('to_learn', 'lama1277-lerek', 'studi')}
 {('to_learn', 'lama1277-adona', 'ɡɜˈnuaʔ')}

  Just for clarity:
  There are also the following similarity classes shared between Alorese and LH.
  {('to_learn', 'keda1252-leuba', 'basa'), ('to_learn', 'abui1241-ulaga', 'vosar'), ('to_learn', 'sika1262-hewa', 'belajar'), ('to_learn', 'alor1247-besar', 'aʤar'), ('to_learn', 'pura1258', "be'laɟar"), ('to_learn', 'lama1277-lerek', 'balajar'), ('to_learn', 'kaer1234', "ba'laʤar"), ('to_learn', 'lama1277-lewoi', 'bəˈlaʤɑr'), ('to_learn', 'teiw1235', "bla'ɟar")}


Concept ‘trousers’ is different between Alorese and Lamaholot.

 One or more Lamaholot dialects innovated forms not found in Alorese:
 {('trousers', 'lama1277-lewoi', 'ˈbru beˈlahaʔaŋ')}
 {('trousers', 'adan1251-otvai', 'deko’lai'), ('trousers', 'lama1277-lerek', 'kəˈdeko ˈdoak'), ('trousers', 'lamm1241-westp', 'deku'), ('trousers', 'lama1277-kalik', 'deko'), ('trousers', 'kelo1247-hopte', "dɛk gi'la:ŋ"), ('trousers', 'lama1277-kalik', 'ˈdeko (doˈak)'), ('trousers', 'lama1277-kalik', 'dekor')}

  Just for clarity:
  There are also the following similarity classes shared between Alorese and LH.
  {('trousers', 'alor1247-besar', "deko 'leiŋ bəla'hakaŋ"), ('trousers', 'lama1277-adona', 'ˈdekoˈblahã'), ('trousers', 'keda1252-leuba', "deko 'lawan"), ('trousers', 'alor1247-munas', "deko 'leiŋ b'lahak"), ('trousers', 'alor1247-pandai', "deko 'leiŋ b'lahak")}


Concept ‘man’ is different between Alorese and Lamaholot.

 One or more Lamaholot dialects innovated forms not found in Alorese:
 {('man', 'rett1240', 'amu'), ('man', 'kama1365', 'lɑmɪ'), ('man', 'lama1277-imulo', 'lamen'), ('man', 'lama1277-lewok', 'lame'), ('man', 'lama1277-lewuk', 'lameja'), ('man', 'lama1277-lewop', 'analame'), ('man', 'wers1238-marit', 'limi'), ('man', 'wers1238-taram', 'lmi'), ('man', 'lamm1241-westp', 'am:u'), ('man', 'lama1277-kalik', 'lamen'), ('man', 'lama1277-lewot', 'ata lamen'), ('man', 'lama1277-kalik', 'laˈmɛn')}
 {('man', 'lama1277-lewoe', 'rəbã'), ('man', 'lama1277-lamat', 'reba')}

  Just for clarity:
  There are also the following similarity classes shared between Alorese and LH.
  {('man', 'lama1277-pukau', 'kake'), ('man', 'lama1277-waiwa', 'amalake'), ('man', 'lama1277-ileap', 'amalake'), ('man', 'lama1277-minga', 'analaki'), ('man', 'lama1277-dulhi', 'amalake'), ('man', 'lama1277-adona', 'ˈʔama lake'), ('man', 'lama1277-tanju', 'amalake'), ('man', 'lama1277-kiwan', 'amalake'), ('man', 'lama1277-waiba', 'bəlaki'), ('man', 'lama1277-belan', 'kəbailake'), ('man', 'alor1247-munas', "k'lake"), ('man', 'lama1277-baipi', 'bəlaki'), ('man', 'sika1262-hewa', 'lai'), ('man', 'lama1277-lewom', 'amalake'), ('man', 'lama1277-lamak', 'bəlakeʔ'), ('man', 'sika1262-hewa', 'laʔit'), ('man', 'lama1277-ritae', 'bəlake'), ('man', 'p-mala1545-abvd', '*laki'), ('man', 'sika1262-tanai', 'ata laʔi'), ('man', 'lama1277-wuake', 'kəlake'), ('man', 'lama1277-horow', 'amalake'), ('man', 'lama1277-botun', 'bəlakĩ'), ('man', 'p-cent2245-abvd', '*laki'), ('man', 'lama1277-lewob', 'bailaki'), ('man', 'lama1277-watan', 'bəlake'), ('man', 'alor1247-baran', "ka'lake"), ('man', 'lama1277-lewog', 'amalake'), ('man', 'lama1277-lamal', 'kəbaelake'), ('man', 'alor1247-pandai', "k'lake"), ('man', 'p-mala1545-acd', '*laki'), ('man', 'lama1277-lamah', 'bəlake'), ('man', 'sika1262-maume', 'laʔi'), ('man', 'lama1277-lewoi', 'ina məlaˈke'), ('man', 'lama1277-bama', 'amalake'), ('man', 'lama1277-mulan', 'kəblake'), ('man', 'lama1277-merde', 'kəbəlake'), ('man', 'lama1277-lerek', 'laˈkin'), ('man', 'lama1277-paina', 'lakin'), ('man', 'alor1247-besar', "ka'lake")}


Concept ‘earring’ is different between Alorese and Lamaholot.

 Alorese innovated the following forms:
 {('earring', 'alor1247-munas', "mɔ'lːiʔ")}
 One or more Lamaholot dialects innovated forms not found in Alorese:
 {('earring', 'lama1277-lerek', 'ˈdʒ͡ivaŋ'), ('earring', 'lama1277-kalik', 'ɡiˈvaŋ')}
 {('earring', 'kula1280-lanto', '’ma:la'), ('earring', 'wers1238-marit', "mə'lauŋ"), ('earring', 'sawi1256', "məla'wana"), ('earring', 'lama1277-lewoi', 'bəˈlaoŋ'), ('earring', 'abui1241-ulaga', 'mala:ŋ')}

  Just for clarity:
  There are also the following similarity classes shared between Alorese and LH.
  {('earring', 'alor1247-besar', 'anti'), ('earring', 'keda1252-leuba', 'anti'), ('earring', 'alor1247-munas', 'anti'), ('earring', 'rett1240', 'antil'), ('earring', 'lama1277-adona', 'ˈʔatĩ'), ('earring', 'alor1247-pandai', "anti-'anti")}


Concept ‘necklace’ is different between Alorese and Lamaholot.

 Alorese innovated the following forms:
 {('necklace', 'alor1247-munas', 'emar')}
 {('necklace', 'alor1247-besar', 'maŋge'), ('necklace', 'alor1247-munas', 'maŋgɛr'), ('necklace', 'alor1247-munas', "maŋgɛr 'bɔta"), ('necklace', 'alor1247-pandai', 'maŋger')}
 One or more Lamaholot dialects innovated forms not found in Alorese:
 {('necklace', 'lama1277-lerek', 'ˈniŋal'), ('necklace', 'lama1277-adona', 'ˈnilɜ'), ('necklace', 'lama1277-kalik', 'niˈləŋ'), ('necklace', 'lama1277-lewoi', 'ˈniləŋ')}


Concept ‘scabies’ is different between Alorese and Lamaholot.

 Alorese innovated the following forms:
 {('scabies', 'alor1247-besar', 'katu'), ('scabies', 'alor1247-munas', "katu wə'ne"), ('scabies', 'alor1247-munas', 'katu '), ('scabies', 'alor1247-pandai', 'katu')}
 One or more Lamaholot dialects innovated forms not found in Alorese:
 {('scabies', 'lama1277-lewoi', 'kəˈbuʔu'), ('scabies', 'lama1277-adona', 'kəˈbuʔu'), ('scabies', 'lama1277-kalik', 'kəˈbura'), ('scabies', 'lama1277-lerek', 'kəpuˈrok'), ('scabies', 'keda1252-leuba', "pu'rɔʔ")}


Concept ‘Earth’ is different between Alorese and Lamaholot.

 Alorese innovated the following forms:
 {('Earth', 'alor1247-besar', 'bumi'), ('Earth', 'alor1247-pandai', 'bumi')}

  Just for clarity:
  There are also the following similarity classes shared between Alorese and LH.
  {('Earth', 'alor1247-munas', 'tana'), ('Earth', 'lama1277-lewoi', 'tana ˈʔɛkɑŋ'), ('Earth', 'lama1277-waiwa', 'tana'), ('Earth', 'lama1277-lewob', 'tana'), ('Earth', 'lama1277-paina', 'ənay'), ('Earth', 'lama1277-belan', 'tana'), ('Earth', 'lama1277-merde', 'tana'), ('Earth', 'wers1238-taram', '(dunia)'), ('Earth', 'lama1277-pukau', 'tana'), ('Earth', 'sika1262-maume', 't̪ana'), ('Earth', 'lama1277-lewuk', 'ənaj'), ('Earth', 'p-mala1545-abvd', '*tanaq'), ('Earth', 'p-cent2245-abvd', '*tanaq'), ('Earth', 'lama1277-lewop', 'enaj'), ('Earth', 'lama1277-adona', "tana 'ʔekã"), ('Earth', 'lama1277-lewog', 'tana'), ('Earth', 'lama1277-kalik', 'ənaj'), ('Earth', 'lama1277-lewok', 'ənaj'), ('Earth', 'lama1277-kiwan', 'tana'), ('Earth', 'alor1247-baran', 'tana'), ('Earth', 'lama1277-watan', 'tana'), ('Earth', 'sika1262-hewa', 'nian tana'), ('Earth', 'lama1277-wuake', 'tana'), ('Earth', 'lama1277-mulan', 'tana'), ('Earth', 'lama1277-bama', 'tanʔa'), ('Earth', 'lama1277-imulo', 'ənay'), ('Earth', 'lama1277-lewom', 'tana'), ('Earth', 'lama1277-ritae', 'tana'), ('Earth', 'lama1277-lerek', 'ˈtana ˈekan'), ('Earth', 'p-mala1545-abvd', '*taneq'), ('Earth', 'sika1262-hewa', 'tana'), ('Earth', 'lama1277-ileap', 'tana'), ('Earth', 'lama1277-botun', 'tana'), ('Earth', 'lama1277-dulhi', 'tana'), ('Earth', 'lama1277-horow', 'tana'), ('Earth', 'lama1277-lamah', 'tana'), ('Earth', 'lama1277-lewot', 'enaj'), ('Earth', 'lama1277-lamak', 'tana'), ('Earth', 'sika1262-tanai', 'tana'), ('Earth', 'lama1277-lamat', 'tana'), ('Earth', 'lama1277-waiba', 'tana'), ('Earth', 'lama1277-kalik', 'ˈtana ˈɛkan'), ('Earth', 'lama1277-lamal', 'tana'), ('Earth', 'kira1248', 'dunia'), ('Earth', 'lama1277-tanju', 'tana'), ('Earth', 'lama1277-baipi', 'tana'), ('Earth', 'lama1277-lewoe', 'tana'), ('Earth', 'lama1277-minga', 'ənaj')}


Concept ‘thatch_for_roofing’ is different between Alorese and Lamaholot.

 Alorese innovated the following forms:
 {('thatch_for_roofing', 'alor1247-pandai', "takar 'luo")}
 One or more Lamaholot dialects innovated forms not found in Alorese:
 {('thatch_for_roofing', 'lama1277-lerek', 'ˈrear')}

  Just for clarity:
  There are also the following similarity classes shared between Alorese and LH.
  {('thatch_for_roofing', 'lama1277-lewoi', 'ˈluʔɔ'), ('thatch_for_roofing', 'alor1247-pandai', 'luo'), ('thatch_for_roofing', 'alor1247-besar', 'luo'), ('thatch_for_roofing', 'tuku1254', 'lei'), ('thatch_for_roofing', 'kema1243', 'lei'), ('thatch_for_roofing', 'lama1277-kalik', 'luˈɔ'), ('thatch_for_roofing', 'alor1247-munas', 'luo'), ('thatch_for_roofing', 'lama1277-kalik', 'luˈɔr'), ('thatch_for_roofing', 'lama1277-adona', 'ˈluʔo')}


Concept ‘gum_tree’ is different between Alorese and Lamaholot.

 Alorese innovated the following forms:
 {('gum_tree', 'alor1247-munas', 'nihɛr'), ('gum_tree', 'alor1247-pandai', 'nihɛr')}
 {('gum_tree', 'alor1247-besar', "a'rːɛŋ")}
 One or more Lamaholot dialects innovated forms not found in Alorese:
 {('gum_tree', 'kuii1253', 'pokoil'), ('gum_tree', 'atim1239', 'puokdai'), ('gum_tree', 'abui1241-takal', "bä.'tä: qul"), ('gum_tree', 'lama1277-lerek', 'ˈpukei'), ('gum_tree', 'kira1248', 'pokul'), ('gum_tree', 'abui1241-ulaga', 'puokal')}

  Just for clarity:
  There are also the following similarity classes shared between Alorese and LH.
  {('gum_tree', 'maka1316', "ate'buti"), ('gum_tree', 'fata1247', "ɛtɛ 'piti"), ('gum_tree', 'lama1277-lewoi', 'kajo ˈburaŋ'), ('gum_tree', 'alor1247-besar', "kaʤɔ bu'rakaŋ"), ('gum_tree', 'lama1277-kalik', 'ˈkaʤu buˈʤak'), ('gum_tree', 'keda1252-leuba', "ʔaju 'bujaʔ")}


Concept ‘flying_fox’ is different between Alorese and Lamaholot.

 Alorese innovated the following forms:
 {('flying_fox', 'alor1247-besar', "la'bɛruŋ")}
 {('flying_fox', 'alor1247-munas', "mi'hua"), ('flying_fox', 'alor1247-pandai', 'mihua')}
 One or more Lamaholot dialects innovated forms not found in Alorese:
 {('flying_fox', 'lama1277-adona', 'ˈʔobɔʔ')}
 {('flying_fox', 'lama1277-lerek', 'miˈker'), ('flying_fox', 'lama1277-kalik', 'kəmiˈki'), ('flying_fox', 'lama1277-kalik', 'kəmiˈkɛr')}
 {('flying_fox', 'lama1277-lewoi', 'ˈbɔkɑŋ')}


Concept ‘treaty’ is different between Alorese and Lamaholot.

 Alorese innovated the following forms:
 {('treaty', 'alor1247-pandai', "li 'uta")}
 Alorese shares the following forms with other AN languanges, but not Lamaholot:
 {('treaty', 'adan1251-otvai', 'bɛl’basa'), ('treaty', 'alor1247-munas', "bela 'badʒa"), ('treaty', 'alor1247-besar', "bela 'baʤa"), ('treaty', 'keda1252-leuba', 'bɛlɑn bajɑn')}
 One or more Lamaholot dialects innovated forms not found in Alorese:
 {('treaty', 'lama1277-lewoi', 'ˈɡɑhiŋ (ˈɡire)')}
 {('treaty', 'lama1277-adona', 'ˈbaja')}


Concept ‘to_sail’ is different between Alorese and Lamaholot.

 One or more Lamaholot dialects innovated forms not found in Alorese:
 {('to_sail', 'lama1277-lerek', 'ˈbua ˈlaja'), ('to_sail', 'sika1262-hewa', 'berlajar')}
 {('to_sail', 'lama1277-kalik', 'ˈleva')}

  Just for clarity:
  There are also the following similarity classes shared between Alorese and LH.
  {('to_sail', 'lama1277-lewoi', 'bua ˈtɛna'), ('to_sail', 'alor1247-munas', 'buaʔ'), ('to_sail', 'alor1247-pandai', 'bua '), ('to_sail', 'alor1247-besar', 'bua'), ('to_sail', 'lama1277-adona', 'buaː')}


Concept ‘thick’ is different between Alorese and Lamaholot.

 One or more Lamaholot dialects innovated forms not found in Alorese:
 {('thick', 'lama1277-lerek', 'prəˈvak'), ('thick', 'lama1277-adona', 'pɜˈwɜrã'), ('thick', 'lama1277-lamal', 'pəfəre'), ('thick', 'lama1277-kalik', 'pərəwak'), ('thick', 'lama1277-kiwan', 'pəwərã'), ('thick', 'lama1277-lamat', 'prəwa'), ('thick', 'lama1277-mulan', 'pərəwɤ'), ('thick', 'lama1277-botun', 'pəwərã'), ('thick', 'lama1277-lewoe', 'wəra'), ('thick', 'lama1277-watan', 'pəwəre'), ('thick', 'lama1277-ileap', 'fəran'), ('thick', 'lama1277-waiwa', 'pəwəre'), ('thick', 'lama1277-lewuk', 'pəfərak'), ('thick', 'lama1277-ritae', 'pəwərẽ'), ('thick', 'lama1277-lewob', 'kəwara'), ('thick', 'lama1277-waiba', 'pəwəre'), ('thick', 'lama1277-paina', 'pərəwakən'), ('thick', 'lama1277-lamah', 'pəwərã'), ('thick', 'lama1277-kalik', 'prəˈvak'), ('thick', 'lama1277-horow', 'pəwərʔe'), ('thick', 'lama1277-dulhi', 'pəwərã'), ('thick', 'lama1277-lamak', 'pəwərʔẽ')}
 {('thick', 'lama1277-lewoi', 'bəˈsiʔ'), ('thick', 'blag1240-warsa', 'pusi'), ('thick', 'baka1276', 'pusi'), ('thick', 'lama1277-lewog', 'bəsiʔ'), ('thick', 'lama1277-tanju', 'bəsiʔ'), ('thick', 'lama1277-bama', 'bəsiʔ'), ('thick', 'blag1240-bama', 'pusi'), ('thick', 'lama1277-lewom', 'bəsĩʔ'), ('thick', 'kama1365', 'kusa'), ('thick', 'kaer1234', 'puso'), ('thick', 'rett1240', 'pusi'), ('thick', 'blag1240-nule', 'pusi'), ('thick', 'blag1240-kulij', 'pusi'), ('thick', 'pura1258', 'pusi'), ('thick', 'blag1240-tuntu', 'pusi')}
 {('thick', 'lama1277-lewok', 'təbalɤ'), ('thick', 'p-cent2245-abvd', '*telu')}
 {('thick', 'lama1277-minga', 'pənəŋe'), ('thick', 'lama1277-lewop', 'pəŋe'), ('thick', 'lama1277-belan', 'pəŋe'), ('thick', 'lama1277-wuake', 'pəŋe'), ('thick', 'lama1277-baipi', 'bate'), ('thick', 'lama1277-imulo', 'pənəŋenən'), ('thick', 'lama1277-merde', 'pənəŋe'), ('thick', 'lama1277-lewot', 'pəŋe')}

  Just for clarity:
  There are also the following similarity classes shared between Alorese and LH.
  {('thick', 'alor1247-baran', 'gapa'), ('thick', 'keda1252-leuwa', 'kapal vahɔʔ'), ('thick', 'keda1252-leuba', 'kapal'), ('thick', 'p-mala1545-abvd', '*ma-kapal'), ('thick', 'keda1252', 'kapal'), ('thick', 'alor1247-munas', 'gapal'), ('thick', 'sika1262-maume', 'apar'), ('thick', 'sika1262-hewa', 'apar'), ('thick', 'sika1262-tanai', 'ʔapar'), ('thick', 'p-mala1545-acd', '*kaS(e)pal'), ('thick', 'alor1247-besar', 'gapa'), ('thick', 'maka1316', 'abaɁa'), ('thick', 'p-aust1307-abvd', '*ma-kaSepal'), ('thick', 'lama1277-pukau', 'təbal'), ('thick', 'alor1247-pandai', 'gapal'), ('thick', 'p-cent2245-abvd', '*kapal')}


Concept ‘crocodile’ is different between Alorese and Lamaholot.

 Alorese shares the following forms with other AN languanges, but not Lamaholot:
 {('crocodile', 'baka1276', 'bapa'), ('crocodile', 'alor1247-pandai', 'bapa'), ('crocodile', 'blag1240-warsa', 'bapa'), ('crocodile', 'blag1240-kulij', 'bapaʔ'), ('crocodile', 'blag1240-tuntu', 'bapa'), ('crocodile', 'alor1247-besar', 'bapa'), ('crocodile', 'alor1247-baran', 'bapa'), ('crocodile', 'pura1258', 'bapa'), ('crocodile', 'blag1240-bama', 'bapa'), ('crocodile', 'blag1240-nule', 'bapa'), ('crocodile', 'rett1240', 'bapa'), ('crocodile', 'alor1247-munas', 'bapa'), ('crocodile', 'keda1252-leuba', "bapa'juː")}
 One or more Lamaholot dialects innovated forms not found in Alorese:
 {('crocodile', 'lama1277-kalik', 'ˈkobu'), ('crocodile', 'lama1277-lewoi', 'ˈkobu'), ('crocodile', 'sika1262-hewa', 'kobu'), ('crocodile', 'lama1277-lerek', 'kɔb'), ('crocodile', 'lama1277-adona', 'ˈkobu')}


Concept ‘to_wake_up’ is different between Alorese and Lamaholot.

 Alorese shares the following forms with TAP languages:
 {('to_wake_up', 'kelo1247-hopte', "mə'tih"), ('to_wake_up', 'kelo1247-bring', 'mətɛh'), ('to_wake_up', 'alor1247-besar', 'tidɛ'), ('to_wake_up', 'rett1240', 'mate:'), ('to_wake_up', 'kafo1240', 'nɑtɛi'), ('to_wake_up', 'abui1241-takal', "dä.'rui.dä"), ('to_wake_up', 'kelo1247-hopte', "hih me'tih"), ('to_wake_up', 'rett1240', 'mate'), ('to_wake_up', 'atim1239', 'na-ruda')}
 One or more Lamaholot dialects innovated forms not found in Alorese:
 {('to_wake_up', 'lama1277-adona', 'ˈhoɡo'), ('to_wake_up', 'lama1277-lewoi', 'ˈhoɡo'), ('to_wake_up', 'keda1252-leuba', 'hɔkɔ'), ('to_wake_up', 'sika1262-hewa', 'hogo')}

  Just for clarity:
  There are also the following similarity classes shared between Alorese and LH.
  {('to_wake_up', 'alor1247-baran', 'baũ'), ('to_wake_up', 'alor1247-pandai', 'bauŋ'), ('to_wake_up', 'alor1247-munas', 'bauŋ'), ('to_wake_up', 'alor1247-munas', 'baŋuŋ'), ('to_wake_up', 'lama1277-lerek', 'baŋ'), ('to_wake_up', 'lama1277-kalik', 'ˈbaŋu')}


Concept ‘horn’ is different between Alorese and Lamaholot.

 Alorese innovated the following forms:
 {('horn', 'alor1247-munas', 'huar'), ('horn', 'alor1247-pandai', 'huar')}
 One or more Lamaholot dialects innovated forms not found in Alorese:
 {('horn', 'lama1277-lerek', 'roˈŋot')}

  Just for clarity:
  There are also the following similarity classes shared between Alorese and LH.
  {('horn', 'alor1247-baran', "ru'ha:"), ('horn', 'alor1247-besar', "u'haraŋ"), ('horn', 'sika1262-tanai', 'tara-ŋ'), ('horn', 'lama1277-lewoi', 'ˈtaraŋ'), ('horn', 'sika1262-hewa', 'taran'), ('horn', 'lama1277-adona', 'taˈrã'), ('horn', 'lama1277-kalik', 'taˈra')}


Concept ‘to_flow’ is different between Alorese and Lamaholot.

 One or more Lamaholot dialects innovated forms not found in Alorese:
 {('to_flow', 'p-mala1545-abvd', '*qaliɾ'), ('to_flow', 'lama1277-belan', 'golo'), ('to_flow', 'lama1277-lamah', 'golo'), ('to_flow', 'lama1277-merde', 'golo'), ('to_flow', 'p-mala1545-acd', '*qaliR'), ('to_flow', 'idat1237', 'nakɑr'), ('to_flow', 'p-mala1545-abvd', '*qaluɾ'), ('to_flow', 'nede1245', 'karári'), ('to_flow', 'p-aust1307-abvd', '*qaluʀ')}
 {('to_flow', 'lama1277-kiwan', 'doro'), ('to_flow', 'kema1243', 'dɔrɔ')}

  Just for clarity:
  There are also the following similarity classes shared between Alorese and LH.
  {('to_flow', 'lama1277-lerek', 'ˈbaŋak'), ('to_flow', 'alor1247-baran', 'pana'), ('to_flow', 'lama1277-bama', 'ba'), ('to_flow', 'alor1247-munas', "wai 'baŋ"), ('to_flow', 'lama1277-ileap', 'baŋ'), ('to_flow', 'lama1277-imulo', 'baŋ'), ('to_flow', 'lama1277-botun', 'baʔ'), ('to_flow', 'lama1277-lewot', 'baŋ'), ('to_flow', 'lama1277-watan', 'ba’ʔ'), ('to_flow', 'lama1277-adona', 'baʔ'), ('to_flow', 'sika1262-tanai', 'ba'), ('to_flow', 'lama1277-lamak', 'ba'), ('to_flow', 'lama1277-lamal', 'bã'), ('to_flow', 'lama1277-lewom', 'ba'), ('to_flow', 'lama1277-kalik', 'baˈŋak'), ('to_flow', 'lama1277-lewob', 'ba'), ('to_flow', 'lama1277-ritae', 'ba'), ('to_flow', 'sika1262-hewa', 'ba'), ('to_flow', 'lama1277-kalik', 'baŋak'), ('to_flow', 'lama1277-minga', 'baŋɤ'), ('to_flow', 'lama1277-lewop', 'baŋ'), ('to_flow', 'lama1277-waiba', 'ba'), ('to_flow', 'lama1277-tanju', 'ba'), ('to_flow', 'lama1277-waiwa', 'ba'), ('to_flow', 'lama1277-lewuk', 'baŋak'), ('to_flow', 'sika1262-maume', 'ba'), ('to_flow', 'lama1277-baipi', 'ba'), ('to_flow', 'lama1277-lewoe', 'bã'), ('to_flow', 'lama1277-mulan', 'bapa'), ('to_flow', 'lama1277-lewoi', 'baː'), ('to_flow', 'alor1247-besar', 'barɛ'), ('to_flow', 'lama1277-lamat', 'bã'), ('to_flow', 'lama1277-pukau', 'ba'), ('to_flow', 'lama1277-lewok', 'baŋak'), ('to_flow', 'alor1247-pandai', "bə'rːɛŋ"), ('to_flow', 'lama1277-wuake', 'baŋak'), ('to_flow', 'lama1277-lewog', 'ba'), ('to_flow', 'lama1277-dulhi', 'baʔ'), ('to_flow', 'lama1277-paina', 'ba'), ('to_flow', 'lama1277-horow', 'baʔ')}


Concept ‘dolphin’ is different between Alorese and Lamaholot.

 Alorese shares the following forms with TAP languages:
 {('dolphin', 'pura1258', 'kuɟa'), ('dolphin', 'teiw1235', "ku'jaɁ"), ('dolphin', 'dein1238', 'kui'), ('dolphin', 'blag1240-tuntu', 'kuʤah'), ('dolphin', 'baka1276', 'kuʤa'), ('dolphin', 'sarr1247-adiab', 'kuja'), ('dolphin', 'alor1247-pandai', 'udʒa'), ('dolphin', 'blag1240-warsa', 'kuʤa'), ('dolphin', 'blag1240-bama', 'kuʤa'), ('dolphin', 'blag1240-nule', 'kuʤa'), ('dolphin', 'alor1247-besar', "ikaŋ 'kuʤae"), ('dolphin', 'blag1240-kulij', 'kuʤa'), ('dolphin', 'kaer1234', 'xuja'), ('dolphin', 'alor1247-munas', 'kudʒa')}
 One or more Lamaholot dialects innovated forms not found in Alorese:
 {('dolphin', 'lama1277-lewoi', 'ˈlumba-ˈlumba'), ('dolphin', 'sika1262-hewa', 'iʔan lumba-lumba'), ('dolphin', 'keda1252-leuba', 'iʔa lumba lumba')}
 {('dolphin', 'lama1277-adona', 'ˈtɜmu')}


Concept ‘with’ is different between Alorese and Lamaholot.

 One or more Lamaholot dialects innovated forms not found in Alorese:
 {('with', 'lama1277-lewoe', 'rua'), ('with', 'lamm1241-westp', '-r'), ('with', 'lamm1241-westp', 'gra')}
 {('with', 'sika1262-hewa', '-ora'), ('with', 'lama1277-lamat', 'ʔo'), ('with', 'idat1237', 'ʔɔrɔ:'), ('with', 'tetu1246', 'hɔ:'), ('with', 'tuku1254', 'ʔɔ:')}
 {('with', 'lama1277-adona', 'ˈtaliʔ')}
 {('with', 'kula1280-lanto', 'wɛ'), ('with', 'abui1241-ulaga', 'fa'), ('with', 'lama1277-lewot', 'fe'), ('with', 'lama1277-minga', 'we'), ('with', 'abui1241-ulaga', 'va'), ('with', 'abui1241-takal', 'fäl')}
 {('with', 'maka1316', 'mai'), ('with', 'lama1277-merde', 'mo'), ('with', 'teiw1235', 'ma'), ('with', 'lama1277-lamat', 'mo'), ('with', 'lama1277-lamah', 'mã'), ('with', 'lama1277-kalik', 'mo')}
 {('with', 'lamm1241-westp', 'gar'), ('with', 'lama1277-lamah', 'kã'), ('with', 'lama1277-merde', 'ko'), ('with', 'lama1277-kalik', 'ko')}

  Just for clarity:
  There are also the following similarity classes shared between Alorese and LH.
  {('with', 'lama1277-lewoi', 'ˈnɔʔɔŋ'), ('with', 'lama1277-dulhi', 'noʔo'), ('with', 'alor1247-munas', 'naŋ'), ('with', 'lama1277-mulan', 'nɤ'), ('with', 'alor1247-pandai', 'naŋ'), ('with', 'kema1243', 'nɔ:'), ('with', 'lama1277-bama', 'no'), ('with', 'tetu1245-suai', 'nɔ:'), ('with', 'lama1277-lewuk', 'no'), ('with', 'kama1365', '-neŋ'), ('with', 'lama1277-horow', 'noʔo'), ('with', 'lama1277-wuake', 'no'), ('with', 'keda1252-leuwa', 'nɔrε'), ('with', 'lama1277-pukau', 'noʔõ'), ('with', 'lama1277-lewob', 'noʔõ'), ('with', 'lama1277-lamal', 'nã'), ('with', 'lama1277-botun', 'noʔo'), ('with', 'laka1255', 'nɔrɔ'), ('with', 'alor1247-besar', 'nɔŋ'), ('with', 'keda1252-leuba', "no're"), ('with', 'lama1277-kalik', 'no'), ('with', 'lama1277-waiba', 'nã'), ('with', 'lama1277-lewok', 'no '), ('with', 'lama1277-paina', 'no'), ('with', 'koto1251', 'n-ok'), ('with', 'p-mala1545-acd', '*deŋan'), ('with', 'tetu1245-vique', 'nɔ:'), ('with', 'lama1277-lewom', 'noʔõ'), ('with', 'lama1277-ritae', 'noʔõ'), ('with', 'lama1277-imulo', 'na'), ('with', 'lama1277-tanju', 'noʔo'), ('with', 'alor1247-baran', 'nɔ̃:'), ('with', 'lama1277-lamak', 'noʔõ'), ('with', 'lama1277-waiwa', 'noʔõ'), ('with', 'mamb1306', 'nɔr'), ('with', 'sika1262-hewa', 'nora'), ('with', 'lama1277-belan', 'no'), ('with', 'lama1277-ileap', 'no'), ('with', 'keda1252', 'nore'), ('with', 'lama1277-lerek', 'no'), ('with', 'lama1277-watan', 'noʔõ'), ('with', 'lama1277-lewog', 'noʔõ'), ('with', 'lama1277-baipi', 'nõ'), ('with', 'lama1277-kiwan', 'noʔõ'), ('with', 'lama1277-lewop', 'no'), ('with', 'lama1277-adona', 'nɔʔ')}


Concept ‘to_worship’ is different between Alorese and Lamaholot.

 Alorese innovated the following forms:
 {('to_worship', 'alor1247-munas', "gamar 'apa")}
 {('to_worship', 'alor1247-munas', "kə'bokuŋ")}
 One or more Lamaholot dialects innovated forms not found in Alorese:
 {('to_worship', 'lama1277-kalik', 'məˈŋaʤi')}

  Just for clarity:
  There are also the following similarity classes shared between Alorese and LH.
  {('to_worship', 'alor1247-pandai', "sə'beaŋ"), ('to_worship', 'alor1247-besar', "sam'beaŋ"), ('to_worship', 'lama1277-lewoi', 'səmˈbajɑŋ'), ('to_worship', 'lama1277-lerek', 'həˈbaja'), ('to_worship', 'alor1247-munas', "sə'beaŋ"), ('to_worship', 'lama1277-adona', 'sɜˈbajã'), ('to_worship', 'pura1258', "sem'baɟaŋ"), ('to_worship', 'pura1258', "sam'baɟaŋ"), ('to_worship', 'sawi1256', 'sambai'), ('to_worship', 'wers1238-marit', "sam'bai"), ('to_worship', 'keda1252-leuba', "sɑm'beʔaŋ")}


Concept ‘younger_brother’ is different between Alorese and Lamaholot.

 Alorese innovated the following forms:
 {('younger_brother', 'alor1247-besar', "ariŋ ka'lake"), ('younger_brother', 'alor1247-munas', "kau k'lake"), ('younger_brother', 'alor1247-pandai', "kau k'lake"), ('younger_brother', 'alor1247-munas', "ulːa k'lake"), ('younger_brother', 'alor1247-pandai', "ula k'lake")}
 {('younger_brother', 'alor1247-pandai', 'naŋ')}
 One or more Lamaholot dialects innovated forms not found in Alorese:
 {('younger_brother', 'lama1277-adona', 'ʔariʔ'), ('younger_brother', 'keda1252-leuba', 'ariʔ'), ('younger_brother', 'lama1277-lewoi', 'ariʔ')}


Concept ‘new’ is different between Alorese and Lamaholot.

 Alorese shares the following forms with other AN languanges, but not Lamaholot:
 {('new', 'alor1247-besar', "uma 'funɔŋ"), ('new', 'tetu1245-vique', "uma 'foun"), ('new', 'tetu1245-suai', "uma 'foun"), ('new', 'alor1247-munas', "uma 'wunɔŋ"), ('new', 'alor1247-pandai', "uma 'wunɔŋ")}
 One or more Lamaholot dialects innovated forms not found in Alorese:
 {('new', 'sika1262-hewa', 'ʋərun'), ('new', 'koto1251', 'feʔu'), ('new', 'lama1277-paina', 'wərun'), ('new', 'keda1252-leuba', "wɛ'run"), ('new', 'lama1277-lewop', 'fərun'), ('new', 'lama1277-kalik', 'vəˈrʊn'), ('new', 'lama1277-kalik', 'wərun'), ('new', 'sika1262-tanai', 'βəru'), ('new', 'sika1262-maume', 'vərun'), ('new', 'lama1277-lerek', 'una vəˈrun'), ('new', 'lama1277-lewot', 'fəruja'), ('new', 'lama1277-lewuk', 'wərun'), ('new', 'keda1252', 'werun'), ('new', 'sika1262-hewa', 'wərun'), ('new', 'keda1252-leuwa', 'vεrun taʔεn'), ('new', 'lama1277-imulo', 'wərun')}
 {('new', 'laka1255', "mo'rin"), ('new', 'idat1237', "mu'rin"), ('new', 'lama1277-lamah', 'mureŋ'), ('new', 'fata1247', '(lɛ) miri')}

  Just for clarity:
  There are also the following similarity classes shared between Alorese and LH.
  {('new', 'lama1277-lewob', 'wuʔũ'), ('new', 'lama1277-lewoe', 'wərũ'), ('new', 'lama1277-horow', 'wuʔũ'), ('new', 'lama1277-merde', 'wuʔũ'), ('new', 'lama1277-mulan', 'wu'), ('new', 'lama1277-lamak', 'wuʔũ'), ('new', 'lama1277-botun', 'wuʔũ'), ('new', 'lama1277-adona', 'ˈwuʔũ'), ('new', 'lama1277-belan', 'wu'), ('new', 'lama1277-baipi', 'wuʔũ'), ('new', 'lama1277-kiwan', 'wuʔũ'), ('new', 'lama1277-tanju', 'wuʔũ'), ('new', 'lama1277-lewok', 'wəru'), ('new', 'lama1277-bama', 'wuʔũ'), ('new', 'alor1247-baran', 'funɔ'), ('new', 'lama1277-dulhi', 'wuʔũ'), ('new', 'lama1277-ileap', 'wun'), ('new', 'lama1277-waiwa', 'wu'), ('new', 'lama1277-lewoi', 'ˈwuʔuŋ'), ('new', 'lama1277-wuake', 'wuɁũ'), ('new', 'lama1277-waiba', 'wuʔũ'), ('new', 'lama1277-lamal', 'fu'), ('new', 'lama1277-minga', 'wəru'), ('new', 'lama1277-ritae', 'wuʔũ'), ('new', 'lama1277-lamat', 'wərũ'), ('new', 'lama1277-lewog', 'wuʔũ'), ('new', 'lama1277-pukau', 'wuʔu'), ('new', 'lama1277-lewom', 'wuʔũ'), ('new', 'lama1277-watan', 'wuʔũ'), ('new', 'alor1247-pandai', 'wunɔŋ')}


Concept ‘to_bless’ is different between Alorese and Lamaholot.

 Alorese innovated the following forms:
 {('to_bless', 'alor1247-pandai', "b'rakːaʔ"), ('to_bless', 'teiw1235', "bra'kaɁ")}
 {('to_bless', 'alor1247-munas', "mama a'lapːɔ k'waraŋ 'tite")}
 {('to_bless', 'alor1247-besar', "basa 'doa")}
 Alorese shares the following forms with TAP languages:
 {('to_bless', 'alor1247-pandai', "b'rakːaʔ"), ('to_bless', 'teiw1235', "bra'kaɁ")}
 {('to_bless', 'alor1247-munas', "mama a'lapːɔ k'waraŋ 'tite")}
 {('to_bless', 'alor1247-besar', "basa 'doa")}
 One or more Lamaholot dialects innovated forms not found in Alorese:
 {('to_bless', 'lama1277-kalik', 'bekurˈnia')}
 {('to_bless', 'lama1277-lerek', 'həbəˈtir')}
 {('to_bless', 'keda1252-leuba', 'bərkat'), ('to_bless', 'lama1277-lewoi', 'ˈbərkat'), ('to_bless', 'lama1277-adona', 'bɜrˈkaːt'), ('to_bless', 'sika1262-hewa', 'bəli bɛrkat')}
 {('to_bless', 'fata1247', 'bɛnsa'), ('to_bless', 'lama1277-kalik', 'ˈbəri  ˈbensa'), ('to_bless', 'tetu1245-suai', 'bɛnsa'), ('to_bless', 'buna1278-suai', 'bensaun'), ('to_bless', 'buna1278-bobon', 'bensaun'), ('to_bless', 'tetu1245-vique', 'bɛnsa'), ('to_bless', 'maka1316', 'bɛ~nsa')}


Concept ‘loom’ is different between Alorese and Lamaholot.

 Alorese innovated the following forms:
 {('loom', 'alor1247-besar', 'fulɔ')}
 {('loom', 'alor1247-munas', "he b'lia"), ('loom', 'alor1247-besar', 'baliaŋ'), ('loom', 'pura1258', "pa'liaŋ"), ('loom', 'alor1247-pandai', 'he'), ('loom', 'alor1247-besar', 'he'), ('loom', 'alor1247-munas', "he 'nəŋ b'lia"), ('loom', 'alor1247-pandai', 'blia')}
 {('loom', 'alor1247-besar', 'opa')}
 Alorese shares the following forms with TAP languages:
 {('loom', 'alor1247-besar', 'fulɔ')}
 {('loom', 'alor1247-munas', "he b'lia"), ('loom', 'alor1247-besar', 'baliaŋ'), ('loom', 'pura1258', "pa'liaŋ"), ('loom', 'alor1247-pandai', 'he'), ('loom', 'alor1247-besar', 'he'), ('loom', 'alor1247-munas', "he 'nəŋ b'lia"), ('loom', 'alor1247-pandai', 'blia')}
 {('loom', 'alor1247-besar', 'opa')}
 One or more Lamaholot dialects innovated forms not found in Alorese:
 {('loom', 'lama1277-kalik', 'knəˈkum')}
 {('loom', 'p-mala1545-acd', '*tenun-an'), ('loom', 'rett1240', 'tananɛŋ'), ('loom', 'lama1277-lewoi', 'təˈnaneʔ'), ('loom', 'lama1277-lerek', 'ˈtənane')}


Concept ‘grains’ is different between Alorese and Lamaholot.

 Alorese shares the following forms with other AN languanges, but not Lamaholot:
 {('grains', 'alor1247-munas', 'pare'), ('grains', 'kama1365', 'bileː'), ('grains', 'tetu1245-suai', 'hare'), ('grains', 'alor1247-besar', 'pari'), ('grains', 'tetu1245-vique', 'hare'), ('grains', 'alor1247-pandai', 'parɛ'), ('grains', 'alor1247-pandai', 'apa')}
 One or more Lamaholot dialects innovated forms not found in Alorese:
 {('grains', 'lama1277-lerek', 'ˈkəluok')}
 {('grains', 'lama1277-adona', 'ˈwahaʔ')}
 {('grains', 'lama1277-kalik', 'liˈsɔ-ʤa')}
 {('grains', 'lama1277-lewoi', 'ˈtahaŋ')}


Concept ‘innocent’ is different between Alorese and Lamaholot.

 One or more Lamaholot dialects innovated forms not found in Alorese:
 {('innocent', 'lama1277-lerek', 'tək no salahi')}
 {('innocent', 'lama1277-lewoi', 'ˈmurəŋ'), ('innocent', 'lama1277-kalik', 'muˈʤək')}

  Just for clarity:
  There are also the following similarity classes shared between Alorese and LH.
  {('innocent', 'tetu1245-suai', "la 'sa:la"), ('innocent', 'alor1247-pandai', "hala 'lahɛ"), ('innocent', 'alor1247-besar', "hala 'lahɛ"), ('innocent', 'lama1277-adona', 'nalã hɑlɑ'), ('innocent', 'alor1247-munas', "hala 'lahɛ"), ('innocent', 'tetu1245-vique', "la 'sa:la")}


Concept ‘younger_sibling’ is different between Alorese and Lamaholot.

 Alorese innovated the following forms:
 {('younger_sibling', 'alor1247-pandai', 'ula'), ('younger_sibling', 'alor1247-munas', 'ulːa')}
 Alorese shares the following forms with other AN languanges, but not Lamaholot:
 {('younger_sibling', 'buna1278-malia', 'khɑu'), ('younger_sibling', 'buna1278-suai', 'khau'), ('younger_sibling', 'tetu1245-suai', 'kau'), ('younger_sibling', 'alor1247-pandai', 'kau'), ('younger_sibling', 'alor1247-munas', 'kau'), ('younger_sibling', 'tetu1245-vique', 'kau'), ('younger_sibling', 'buna1278-bobon', 'khau')}
 One or more Lamaholot dialects innovated forms not found in Alorese:
 {('younger_sibling', 'teiw1235', "gə'i:k"), ('younger_sibling', 'lama1277-tanju', 'adeʔ'), ('younger_sibling', 'lama1277-lewom', 'ade'), ('younger_sibling', 'lama1277-baipi', 'adeʔ'), ('younger_sibling', 'lama1277-lewuk', 'adik'), ('younger_sibling', 'lama1277-waiba', 'adeʔ')}

  Just for clarity:
  There are also the following similarity classes shared between Alorese and LH.
  {('younger_sibling', 'lama1277-minga', 'waji'), ('younger_sibling', 'lama1277-kalik', 'vaˈʤin'), ('younger_sibling', 'lama1277-watan', 'arik'), ('younger_sibling', 'lama1277-lerek', 'vaˈdʒ͡i'), ('younger_sibling', 'lama1277-imulo', 'waji'), ('younger_sibling', 'kelo1247-bring', 'ʊlɪk'), ('younger_sibling', 'lama1277-lamat', 'ari'), ('younger_sibling', 'kema1243', 'alir'), ('younger_sibling', 'lama1277-ileap', 'ari'), ('younger_sibling', 'koto1251', 'orif'), ('younger_sibling', 'lama1277-kalik', 'waji'), ('younger_sibling', 'lama1277-lewop', 'wajin'), ('younger_sibling', 'lama1277-lewob', 'ari'), ('younger_sibling', 'laka1255', 'wali'), ('younger_sibling', 'keda1252-leuba', "ʔa'riʔ"), ('younger_sibling', 'lama1277-paina', 'wajig'), ('younger_sibling', 'lama1277-ritae', 'arĩ'), ('younger_sibling', 'lama1277-merde', 'ari'), ('younger_sibling', 'sika1262-hewa', 'ʋari'), ('younger_sibling', 'lama1277-lamah', 'arikə'), ('younger_sibling', 'lama1277-wuake', 'ari'), ('younger_sibling', 'lama1277-mulan', 'ari'), ('younger_sibling', 'lama1277-lamak', 'arĩ'), ('younger_sibling', 'lama1277-lamal', 'arik'), ('younger_sibling', 'lama1277-waiwa', 'arĩ'), ('younger_sibling', 'lama1277-lewoi', 'ˈariŋ'), ('younger_sibling', 'lama1277-lewot', 'fajin'), ('younger_sibling', 'lama1277-botun', 'arik'), ('younger_sibling', 'mamb1306', 'ʔɑli'), ('younger_sibling', 'lama1277-pukau', 'arĩ'), ('younger_sibling', 'lama1277-horow', 'arik'), ('younger_sibling', 'lama1277-bama', 'ariʔ'), ('younger_sibling', 'alor1247-baran', 'ʔari ̃'), ('younger_sibling', 'lama1277-lewog', 'ariʔ'), ('younger_sibling', 'lama1277-belan', 'ari'), ('younger_sibling', 'alor1247-besar', 'ariŋ'), ('younger_sibling', 'alor1247-munas', 'ariŋ'), ('younger_sibling', 'alor1247-pandai', 'ariŋ'), ('younger_sibling', 'lama1277-lewoe', 'wari'), ('younger_sibling', 'lama1277-adona', 'ˈʔarim'), ('younger_sibling', 'lama1277-kiwan', 'arik'), ('younger_sibling', 'lama1277-lewok', 'wajiga'), ('younger_sibling', 'keda1252', 'ariʔ'), ('younger_sibling', 'lama1277-dulhi', 'arik')}


Concept ‘dry_in_sun’ is different between Alorese and Lamaholot.

 Alorese innovated the following forms:
 {('dry_in_sun', 'alor1247-besar', "peiŋ 'pakiaŋ")}
 {('dry_in_sun', 'alor1247-pandai', "paiŋ apa'hire")}

  Just for clarity:
  There are also the following similarity classes shared between Alorese and LH.
  {('dry_in_sun', 'blag1240-nule', 'poriŋ'), ('dry_in_sun', 'kaer1234', "po'riŋ"), ('dry_in_sun', 'lama1277-adona', 'ˈpahe'), ('dry_in_sun', 'rett1240', 'puali'), ('dry_in_sun', 'blag1240-kulij', 'poriŋ'), ('dry_in_sun', 'kelo1247-hopte', "ʔu'pu:r"), ('dry_in_sun', 'lama1277-adona', 'ˈpaʔĩ'), ('dry_in_sun', 'lama1277-kalik', 'paˈri (məˈka)'), ('dry_in_sun', 'p-mala1545-acd', '*bilaj'), ('dry_in_sun', 'keda1252-leuba', "pa'riː"), ('dry_in_sun', 'lamm1241-westp', 'puariŋ'), ('dry_in_sun', 'alor1247-munas', 'paiŋ '), ('dry_in_sun', 'adan1251-otvai', '’poil'), ('dry_in_sun', 'hama1240', 'pʊil'), ('dry_in_sun', 'lama1277-lewoi', 'paˈʔiŋ'), ('dry_in_sun', 'blag1240-tuntu', 'poriŋ'), ('dry_in_sun', 'p-alor1249', '*por'), ('dry_in_sun', 'lama1277-lerek', 'paˈriŋ'), ('dry_in_sun', 'blag1240-bama', 'poriŋ'), ('dry_in_sun', 'baka1276', 'poriŋ'), ('dry_in_sun', 'rett1240', 'pualiŋ'), ('dry_in_sun', 'kelo1247-bring', 'ʊpur'), ('dry_in_sun', 'blag1240-warsa', 'poriŋ'), ('dry_in_sun', 'alor1247-baran', 'paɛ̃:')}


Concept ‘dry’ is different between Alorese and Lamaholot.

 One or more Lamaholot dialects innovated forms not found in Alorese:
 {('dry', 'abui1241-ulaga', 'takata'), ('dry', 'kafo1240', 'tɑkɑtɑ'), ('dry', 'blag1240-bama', 'taxata'), ('dry', 'blag1240-tuntu', "ta'qata"), ('dry', 'abui1241-fuime', 'takata'), ('dry', 'baka1276', 'tata'), ('dry', 'kelo1247-bring', 'təkɑt'), ('dry', 'blag1240-warsa', 'taxata'), ('dry', 'blag1240-nule', 'tata'), ('dry', 'pura1258', "ta'ata"), ('dry', 'kelo1247-hopte', "tə'kat"), ('dry', 'atim1239', 'takata'), ('dry', 'adan1251-lawah', 'taʔata'), ('dry', 'nede1245', 'ʧiʧi'), ('dry', 'kuii1253', 'takata'), ('dry', 'rett1240', 'tati'), ('dry', 'blag1240-kulij', 'tata'), ('dry', 'lama1277-lamak', 'tuʔuk'), ('dry', 'abui1241-takal', "tä.kä.'tä"), ('dry', 'kira1248', 'takata'), ('dry', 'rett1240', "ta'ti"), ('dry', 'kabo1247', 'taʔata'), ('dry', 'hama1240', 'taʔat')}

  Just for clarity:
  There are also the following similarity classes shared between Alorese and LH.
  {('dry', 'maka1316', 'saɁara'), ('dry', 'sika1262-hewa', 'maran'), ('dry', 'sika1262-tanai', 'mara'), ('dry', 'lama1277-botun', 'marʔã'), ('dry', 'lama1277-lamat', 'marʔã'), ('dry', 'lama1277-pukau', 'maraʔã'), ('dry', 'lama1277-lewok', 'majak'), ('dry', 'p-mala1545-abvd', '*ma-ɾaŋaw'), ('dry', 'lama1277-belan', 'mara'), ('dry', 'lama1277-tanju', 'mara'), ('dry', 'lama1277-lewob', 'marã'), ('dry', 'lama1277-dulhi', 'mara'), ('dry', 'keda1252-leuba', "ma'jaʔ"), ('dry', 'lama1277-waiba', 'mara'), ('dry', 'p-cent2245-abvd', '*ma-ʀaŋaw'), ('dry', 'alor1247-baran', 'mara'), ('dry', 'lama1277-watan', 'mara'), ('dry', 'lama1277-kiwan', 'marʔǎ '), ('dry', 'lama1277-minga', 'majak'), ('dry', 'lama1277-lewoi', 'ˈmara'), ('dry', 'lama1277-horow', 'mara'), ('dry', 'lama1277-adona', 'ˈmaʔrã'), ('dry', 'tetu1246', 'marɑn'), ('dry', 'idat1237', 'marɑn'), ('dry', 'lama1277-wuake', 'mara'), ('dry', 'lama1277-lewuk', 'maj'), ('dry', 'lama1277-bama', 'mara'), ('dry', 'lama1277-lamah', 'mara'), ('dry', 'sika1262-maume', 'maran'), ('dry', 'lama1277-imulo', 'majakən'), ('dry', 'lama1277-lewot', 'majakən'), ('dry', 'lama1277-lewog', 'mara'), ('dry', 'lama1277-baipi', 'mara'), ('dry', 'lama1277-kalik', 'maˈʤak'), ('dry', 'tetu1245-suai', 'maran'), ('dry', 'lama1277-lewom', 'marã'), ('dry', 'lama1277-lewop', 'maja'), ('dry', 'lama1277-ritae', 'marã'), ('dry', 'sika1262-hewa', 'mara'), ('dry', 'tetu1245-vique', 'maran'), ('dry', 'lama1277-lerek', 'majaˈkən'), ('dry', 'lama1277-kalik', 'majak'), ('dry', 'lama1277-lewoe', 'mɔrɔ'), ('dry', 'alor1247-munas', 'marak'), ('dry', 'lama1277-mulan', 'mara'), ('dry', 'alor1247-pandai', 'marak'), ('dry', 'lama1277-ileap', 'mara'), ('dry', 'lama1277-lamal', 'marã'), ('dry', 'lama1277-merde', 'marʔã'), ('dry', 'alor1247-besar', 'mara'), ('dry', 'p-aust1307-abvd', '*ma-qaʀiw'), ('dry', 'lama1277-paina', 'mayakən'), ('dry', 'keda1252', 'mayaʔ'), ('dry', 'lama1277-waiwa', 'mara')}


Concept ‘tear’ is different between Alorese and Lamaholot.

 Alorese shares the following forms with other AN languanges, but not Lamaholot:
 {('tear', 'alor1247-munas', "mataŋ 'louŋ"), ('tear', 'tetu1245-suai', 'mata lun'), ('tear', 'alor1247-pandai', "mataŋ 'louŋ"), ('tear', 'tetu1245-vique', 'mata lun')}
 One or more Lamaholot dialects innovated forms not found in Alorese:
 {('tear', 'lama1277-lerek', 'mat waer'), ('tear', 'lama1277-lewoi', 'mata ˈwaʔiŋ'), ('tear', 'lama1277-kalik', 'ˈmata ˈvaen')}

  Just for clarity:
  There are also the following similarity classes shared between Alorese and LH.
  {('tear', 'lama1277-lewoi', 'ˈlouŋ'), ('tear', 'lama1277-adona', 'ˈlõː'), ('tear', 'sika1262-hewa', 'lu'), ('tear', 'alor1247-munas', 'lou'), ('tear', 'alor1247-besar', 'lou'), ('tear', 'alor1247-munas', 'louŋ'), ('tear', 'keda1252-leuba', 'lun')}


Concept ‘honey’ is different between Alorese and Lamaholot.

 Alorese shares the following forms with other AN languanges, but not Lamaholot:
 {('honey', 'keda1252-leuba', "lani 'wɛrɛŋ"), ('honey', 'alor1247-besar', "fa'lanɛ 'fekiŋ"), ('honey', 'alor1247-pandai', "bə'lane 'waiŋ"), ('honey', 'alor1247-munas', "b'lane 'waiŋ")}
 One or more Lamaholot dialects innovated forms not found in Alorese:
 {('honey', 'lama1277-kalik', 'ˈrua ˈvaen')}
 {('honey', 'maka1316', 'wani'), ('honey', 'nede1245', 'toʔoŋ waŋi'), ('honey', 'lama1277-lewoi', 'ˈwaneː'), ('honey', 'lamm1241-westp', 'wani'), ('honey', 'fata1247', 'wani'), ('honey', 'lama1277-adona', 'waˈneʔ')}
 {('honey', 'lama1277-lerek', 'ˈbliwo')}


Concept ‘star’ is different between Alorese and Lamaholot.

 One or more Lamaholot dialects innovated forms not found in Alorese:
 {('star', 'lama1277-kalik', 'toˈna'), ('star', 'lama1277-kalik', 'tona'), ('star', 'lama1277-lamah', 'tona'), ('star', 'lama1277-lewok', 'tonaru'), ('star', 'lama1277-paina', 'tonar'), ('star', 'lama1277-lerek', 'ˈtonar'), ('star', 'lama1277-lewuk', 'tonar'), ('star', 'lama1277-wuake', 'tona'), ('star', 'lama1277-kalik', 'toˈnar'), ('star', 'lama1277-lamal', 'tona'), ('star', 'lama1277-minga', 'tona'), ('star', 'lama1277-imulo', 'tonar'), ('star', 'lama1277-lewot', 'tonaja'), ('star', 'lama1277-lewop', 'tonaru')}
 {('star', 'lama1277-kiwan', 'e-təp'), ('star', 'lama1277-adona', 'ʔetɜp'), ('star', 'lama1277-merde', 'etəp'), ('star', 'lama1277-dulhi', 'etəp'), ('star', 'lama1277-lamak', 'e təp'), ('star', 'lama1277-watan', 'etəp')}
 {('star', 'lamm1241-westp', 'hib:i'), ('star', 'p-east2519', '*ipi(-bere)'), ('star', 'hama1240', 'ibiŋ'), ('star', 'buna1278-bobon', 'bi:'), ('star', 'buna1278-malia', 'bi:'), ('star', 'buna1278-suai', 'bi:'), ('star', 'buna1278-malia', 'bibɛl'), ('star', 'adan1251-otvai', '’ibiŋ'), ('star', 'lama1277-botun', 'epət')}

  Just for clarity:
  There are also the following similarity classes shared between Alorese and LH.
  {('star', 'lama1277-waiba', 'pətala'), ('star', 'lama1277-lewoe', 'malã'), ('star', 'lama1277-lamat', 'malã'), ('star', 'alor1247-munas', 'mala'), ('star', 'lama1277-horow', 'pətala'), ('star', 'alor1247-besar', "ta'mala"), ('star', 'lama1277-ileap', 'təmala'), ('star', 'lama1277-lewob', 'bəlia'), ('star', 'sika1262-tanai', 'dala'), ('star', 'p-mala1545-acd', '*mantalaq'), ('star', 'lama1277-pukau', 'talaʔ'), ('star', 'lama1277-lewoi', 'ˈpətala'), ('star', 'keda1252-leuwa', 'malæ manuʔ'), ('star', 'alor1247-baran', "ta'mala"), ('star', 'lamm1241-westp', 'tamariŋ'), ('star', 'lama1277-waiwa', 'pətala'), ('star', 'lama1277-tanju', 'kətala'), ('star', 'lama1277-mulan', 'bəlia'), ('star', 'lama1277-baipi', 'pətala'), ('star', 'lama1277-bama', 'pətala'), ('star', 'p-mala1545-acd', '*talaq'), ('star', 'keda1252', 'male'), ('star', 'sika1262-hewa', 'dala'), ('star', 'alor1247-pandai', "tə'mala"), ('star', 'keda1252-leuba', "ma'le"), ('star', 'sika1262-maume', 'dala'), ('star', 'lama1277-lewog', 'pətala'), ('star', 'lama1277-ritae', 'bəlia'), ('star', 'keda1252-leuwa', 'malæ tala'), ('star', 'lama1277-lewom', 'pətala'), ('star', 'lama1277-belan', 'təmala')}


Concept ‘to_give’ is different between Alorese and Lamaholot.

 One or more Lamaholot dialects innovated forms not found in Alorese:
 {('to_give', 'lama1277-kalik', 'noto'), ('to_give', 'lama1277-minga', 'noto')}
 {('to_give', 'lama1277-lamal', 'soro'), ('to_give', 'lama1277-lamah', 'soro'), ('to_give', 'lama1277-merde', 'sorõ'), ('to_give', 'keda1252-leuba', 'sɔrɔŋ'), ('to_give', 'lama1277-belan', 'soro'), ('to_give', 'lama1277-lewoi', 'ˈsɔrɔŋ'), ('to_give', 'lama1277-lewog', 'sorõ'), ('to_give', 'lama1277-ileap', 'soro'), ('to_give', 'lama1277-lamat', 'sorõ'), ('to_give', 'lama1277-lewop', 'soro'), ('to_give', 'lama1277-pukau', 'sorõ'), ('to_give', 'lama1277-wuake', 'sorõ'), ('to_give', 'keda1252-leuwa', 'sɔrɔŋ natεŋ'), ('to_give', 'lama1277-lewoe', 'sorõ'), ('to_give', 'lama1277-lewot', 'soro'), ('to_give', 'keda1252', 'soroŋ'), ('to_give', 'lama1277-ritae', 'sorõ'), ('to_give', 'lama1277-waiwa', 'sorõ'), ('to_give', 'lama1277-bama', 'sotõ'), ('to_give', 'lama1277-lewob', 'sorõ')}
 {('to_give', 'lama1277-lerek', 'bɛŋ'), ('to_give', 'p-mala1545-acd', '*pa-beRay'), ('to_give', 'p-east2519', '*-inV'), ('to_give', 'lama1277-paina', 'beŋ'), ('to_give', 'lama1277-kalik', 'be'), ('to_give', 'sika1262-hewa', 'bəli'), ('to_give', 'sika1262-tanai', 'βəli'), ('to_give', 'p-mala1545-acd', '*beRay'), ('to_give', 'lama1277-imulo', 'beŋa')}

  Just for clarity:
  There are also the following similarity classes shared between Alorese and LH.
  {('to_give', 'lama1277-kiwan', 'nei'), ('to_give', 'lama1277-lewuk', 'ne'), ('to_give', 'lama1277-dulhi', 'nei'), ('to_give', 'alor1247-munas', 'niŋ'), ('to_give', 'lama1277-lamak', 'neʔi'), ('to_give', 'mamb1306', 'ne:'), ('to_give', 'lama1277-horow', 'nei'), ('to_give', 'lama1277-tanju', 'nei'), ('to_give', 'idat1237', 'ne:'), ('to_give', 'alor1247-pandai', 'neiŋ'), ('to_give', 'lama1277-botun', 'nei'), ('to_give', 'kema1243', 'nɛ:'), ('to_give', 'lama1277-waiba', 'neʔi'), ('to_give', 'lama1277-lewoi', 'ˈneiŋ'), ('to_give', 'lama1277-mulan', 'ni'), ('to_give', 'lamm1241-westp', '-nia'), ('to_give', 'alor1247-besar', 'nẽ'), ('to_give', 'lama1277-baipi', 'nei'), ('to_give', 'alor1247-pandai', 'niŋ'), ('to_give', 'tuku1254', 'nɛ:'), ('to_give', 'keda1252-leuwa', 'ni'), ('to_give', 'p-timo1261', '*-(e, i)na'), ('to_give', 'laka1255', 'naʔɛ'), ('to_give', 'alor1247-baran', 'nɛĩ'), ('to_give', 'lama1277-lewom', 'nei'), ('to_give', 'lama1277-adona', 'ˈneĩ'), ('to_give', 'lama1277-lewok', 'ne'), ('to_give', 'lama1277-watan', 'nei')}


Concept ‘to_walk’ is different between Alorese and Lamaholot.

 One or more Lamaholot dialects innovated forms not found in Alorese:
 {('to_walk', 'p-alor1249', '*laka'), ('to_walk', 'abui1241-ulaga', 'la:k'), ('to_walk', 'p-east2519', '*lagar'), ('to_walk', 'p-timo1261', '*lak(Vr)'), ('to_walk', 'kafo1240', 'lɑ:kɑ'), ('to_walk', 'kira1248', 'lak'), ('to_walk', 'kuii1253', 'lak'), ('to_walk', 'abui1241-fuime', 'lak'), ('to_walk', 'atim1239', 'lak'), ('to_walk', 'lama1277-paina', 'wage')}
 {('to_walk', 'tetu1245-vique', 'laɁɔ (ajɪŋ)'), ('to_walk', 'p-timo1261', '*lamV'), ('to_walk', 'lama1277-imulo', 'labi'), ('to_walk', 'tetu1246', 'lɑu'), ('to_walk', 'p-mala1545-acd', '*lampaq'), ('to_walk', 'idat1237', 'lɑu'), ('to_walk', 'lama1277-lewuk', 'labi'), ('to_walk', 'kama1365', 'loː')}

  Just for clarity:
  There are also the following similarity classes shared between Alorese and LH.
  {('to_walk', 'lama1277-belan', 'pana'), ('to_walk', 'lama1277-lewok', 'pana'), ('to_walk', 'lama1277-merde', 'pana'), ('to_walk', 'lama1277-kiwan', 'pana'), ('to_walk', 'alor1247-munas', "pana 'leiŋ"), ('to_walk', 'lama1277-adona', 'ˈpana'), ('to_walk', 'alor1247-pandai', 'pana '), ('to_walk', 'lama1277-waiwa', 'pana'), ('to_walk', 'lama1277-lewom', 'pana'), ('to_walk', 'lama1277-ritae', 'pana'), ('to_walk', 'lama1277-tanju', 'pana'), ('to_walk', 'lama1277-ileap', 'pana'), ('to_walk', 'sawi1256', 'jana'), ('to_walk', 'sika1262-maume', 'pano'), ('to_walk', 'keda1252-leuwa', 'pan lεdɔ'), ('to_walk', 'lama1277-horow', 'pana'), ('to_walk', 'lama1277-pukau', 'pana'), ('to_walk', 'alor1247-baran', 'pana'), ('to_walk', 'lama1277-watan', 'pana'), ('to_walk', 'sika1262-hewa', 'pano'), ('to_walk', 'lama1277-lerek', 'ˈpanə'), ('to_walk', 'alor1247-besar', "pana 'leiŋ "), ('to_walk', 'kula1280-lanto', 'jana'), ('to_walk', 'lama1277-minga', 'pana'), ('to_walk', 'lama1277-bama', 'pana'), ('to_walk', 'lama1277-lewoe', 'pã'), ('to_walk', 'lama1277-botun', 'pana'), ('to_walk', 'lama1277-lamah', 'pana'), ('to_walk', 'lama1277-wuake', 'pana'), ('to_walk', 'keda1252-leuba', 'pɑn (lala)'), ('to_walk', 'lama1277-lamak', 'pana'), ('to_walk', 'lama1277-lewoi', 'ˈpana'), ('to_walk', 'lama1277-lewog', 'pana'), ('to_walk', 'alor1247-munas', "pana 'lei"), ('to_walk', 'lama1277-mulan', 'pana'), ('to_walk', 'lama1277-waiba', 'pana'), ('to_walk', 'lama1277-lewop', 'pana'), ('to_walk', 'lama1277-kalik', 'ˈpana məˈra lim'), ('to_walk', 'lama1277-lamal', 'pana'), ('to_walk', 'lama1277-lewot', 'pana'), ('to_walk', 'sika1262-tanai', 'pano'), ('to_walk', 'lama1277-baipi', 'pana'), ('to_walk', 'lama1277-dulhi', 'pana'), ('to_walk', 'lama1277-lamat', 'pana'), ('to_walk', 'lama1277-kalik', 'pana'), ('to_walk', 'lama1277-lewob', 'pana'), ('to_walk', 'keda1252', 'pan'), ('to_walk', 'p-cent2245-abvd', '*panaw')}


Concept ‘to_skewer’ is different between Alorese and Lamaholot.

 One or more Lamaholot dialects innovated forms not found in Alorese:
 {('to_skewer', 'lama1277-kalik', 'roˈbak')}
 {('to_skewer', 'lama1277-lerek', 'tukuˈraŋ')}
 {('to_skewer', 'lama1277-kalik', 'təˈɡu'), ('to_skewer', 'lama1277-adona', 'ˈtɜɡuʔ')}

  Just for clarity:
  There are also the following similarity classes shared between Alorese and LH.
  {('to_skewer', 'keda1252-leuba', 'tuhuʔ'), ('to_skewer', 'p-mala1545-acd', '*tuduk'), ('to_skewer', 'adan1251-otvai', '’teʔ'), ('to_skewer', 'adan1251-lawah', 'tɛʔɛ'), ('to_skewer', 'sika1262-hewa', 'tuhuk'), ('to_skewer', 'alor1247-pandai', 'tuhu'), ('to_skewer', 'lama1277-adona', 'ˈtuhuk'), ('to_skewer', 'tetu1245-vique', 'tu:'), ('to_skewer', 'tetu1245-suai', 'tu:'), ('to_skewer', 'p-mala1545-acd', '*tusuk'), ('to_skewer', 'lama1277-lewoi', 'təˈluhu'), ('to_skewer', 'alor1247-munas', 'tuhuʔ'), ('to_skewer', 'alor1247-besar', 'tuhu')}


Concept ‘person’ is different between Alorese and Lamaholot.

 Alorese shares the following forms with TAP languages:
 {('person', 'fata1247', 'ma:r louhana'), ('person', 'alor1247-munas', "mə'sia"), ('person', 'fata1247', 'ma:r'), ('person', 'alor1247-pandai', "mə'sia")}
 One or more Lamaholot dialects innovated forms not found in Alorese:
 {('person', 'lama1277-wuake', 'atadikẽ'), ('person', 'lama1277-lamah', 'dikã'), ('person', 'lama1277-ritae', 'atadikã'), ('person', 'lama1277-lewoe', 'atadiyã'), ('person', 'lama1277-kalik', 'ˈata diˈkən'), ('person', 'lama1277-lewob', 'atadikã'), ('person', 'lama1277-minga', 'atadikə'), ('person', 'lama1277-watan', 'atadikã'), ('person', 'lama1277-belan', 'atadik'), ('person', 'lama1277-merde', 'ədikã'), ('person', 'lama1277-mulan', 'atadikɤ'), ('person', 'lama1277-lewog', 'atadikã'), ('person', 'lama1277-lewom', 'atadikẽ'), ('person', 'lama1277-waiba', 'atadikã'), ('person', 'lama1277-lamal', 'atadikã'), ('person', 'lama1277-kalik', 'atadikən'), ('person', 'lama1277-botun', 'atadikə'), ('person', 'lama1277-lamat', 'ədiʔã'), ('person', 'lama1277-ileap', 'atadikən'), ('person', 'keda1252', 'atadiʔen'), ('person', 'lama1277-paina', 'atawiha'), ('person', 'keda1252-leuba', "ate 'diʔen"), ('person', 'lama1277-pukau', 'atadikẽ'), ('person', 'lama1277-lerek', 'ata dikən'), ('person', 'lama1277-imulo', 'atadikəja'), ('person', 'lama1277-lewot', 'atadikəja'), ('person', 'lama1277-baipi', 'atadikə'), ('person', 'lama1277-waiwa', 'atadike'), ('person', 'lama1277-lewop', 'atadikəj'), ('person', 'lama1277-lewoi', 'ata dikəˈʔəŋ'), ('person', 'lama1277-dulhi', 'atadikã'), ('person', 'lama1277-horow', 'atadikã'), ('person', 'lama1277-kiwan', 'atadikʔã'), ('person', 'lama1277-lamak', 'atadikã')}
 {('person', 'lama1277-lewok', 'səlik')}

  Just for clarity:
  There are also the following similarity classes shared between Alorese and LH.
  {('person', 'p-mala1545-acd', '*tau'), ('person', 'lama1277-lewuk', 'ate'), ('person', 'p-aust1307-abvd', '*Cau'), ('person', 'p-cent2245-abvd', '*tau'), ('person', 'p-mala1545-abvd', '*tau'), ('person', 'alor1247-munas', 'ata'), ('person', 'lama1277-bama', 'atã'), ('person', 'alor1247-pandai', 'ata'), ('person', 'tuku1254', 'ʔatu'), ('person', 'lama1277-tanju', 'ata'), ('person', 'sika1262-hewa', 'ata'), ('person', 'lama1277-adona', 'ˈʔata'), ('person', 'alor1247-besar', 'ata'), ('person', 'alor1247-baran', 'ʔata')}


Concept ‘3pl’ is different between Alorese and Lamaholot.

 Alorese innovated the following forms:
 {('3pl', 'alor1247-baran', "fe: sa'kali")}
 {('3pl', 'alor1247-pandai', 'we'), ('3pl', 'alor1247-munas', 'we'), ('3pl', 'alor1247-besar', 'fe')}
 One or more Lamaholot dialects innovated forms not found in Alorese:
 {('3pl', 'lama1277-lewok', 'yane'), ('3pl', 'lama1277-kalik', 'dane'), ('3pl', 'lama1277-lewop', 'daro'), ('3pl', 'lama1277-kalik', 'da'), ('3pl', 'lama1277-paina', 'dane'), ('3pl', 'lama1277-lewuk', 'dan'), ('3pl', 'lama1277-minga', 'daro'), ('3pl', 'lama1277-lewot', 'da'), ('3pl', 'lama1277-imulo', 'daro'), ('3pl', 'lama1277-lerek', 'ˈdane')}
 {('3pl', 'lama1277-watan', 'raʔe'), ('3pl', 'lama1277-lewog', 'ra'), ('3pl', 'lama1277-lewom', 'raʔe'), ('3pl', 'lama1277-ritae', 'raʔe'), ('3pl', 'lama1277-lamal', 'rae'), ('3pl', 'tuku1254', 'rɔʔɔ'), ('3pl', 'lama1277-mulan', 'rae'), ('3pl', 'lama1277-kiwan', 'raʔe'), ('3pl', 'lama1277-pukau', 'ra'), ('3pl', 'lama1277-wuake', 'raɁe'), ('3pl', 'lama1277-lamak', 'raʔe'), ('3pl', 'lama1277-botun', 'raʔe'), ('3pl', 'lama1277-lewoi', 'ˈraː'), ('3pl', 'lama1277-lewob', 'ra'), ('3pl', 'lama1277-merde', 'raʔe'), ('3pl', 'lama1277-lamat', 'roʔe'), ('3pl', 'lama1277-lewoe', 'roʔe'), ('3pl', 'lama1277-tanju', 'ra'), ('3pl', 'lama1277-baipi', 'raʔe'), ('3pl', 'lama1277-waiba', 'raʔe'), ('3pl', 'lama1277-ileap', 'ra'), ('3pl', 'lama1277-adona', 'ˈraʔe'), ('3pl', 'lama1277-lamah', 'raʔe'), ('3pl', 'lama1277-bama', 'ra'), ('3pl', 'lama1277-belan', 'rae'), ('3pl', 'lama1277-horow', 'raʔe'), ('3pl', 'lama1277-dulhi', 'raʔe'), ('3pl', 'lama1277-waiwa', 'rae')}


Concept ‘king_ruler’ is different between Alorese and Lamaholot.

 Alorese innovated the following forms:
 {('king_ruler', 'alor1247-pandai', "paha 'lawo")}
 {('king_ruler', 'alor1247-besar', "ata 'beiŋ"), ('king_ruler', 'alor1247-pandai', "ata 'bea")}
 {('king_ruler', 'alor1247-munas', "peiŋ 'beaʔ")}
 One or more Lamaholot dialects innovated forms not found in Alorese:
 {('king_ruler', 'lama1277-lerek', 'ˈkordes')}
 {('king_ruler', 'kaer1234', 'rai'), ('king_ruler', 'lama1277-lewoi', 'ˈraʤa'), ('king_ruler', 'lamm1241-westp', 'raja'), ('king_ruler', 'sika1262-hewa', 'raʤa'), ('king_ruler', 'teiw1235', 'raj'), ('king_ruler', 'lama1277-adona', 'ˈraja'), ('king_ruler', 'keda1252-leuba', 'radʒa')}


Concept ‘vagina’ is different between Alorese and Lamaholot.

 One or more Lamaholot dialects innovated forms not found in Alorese:
 {('vagina', 'lama1277-kalik', 'məˈna'), ('vagina', 'lama1277-adona', 'ˈmɜna'), ('vagina', 'lama1277-lewoi', 'ˈmena')}
 {('vagina', 'lama1277-kalik', 'vaˈvi')}

  Just for clarity:
  There are also the following similarity classes shared between Alorese and LH.
  {('vagina', 'lama1277-lerek', 'puˈki'), ('vagina', 'alor1247-munas', 'pukiʔ'), ('vagina', 'p-mala1545-acd', '*puki'), ('vagina', 'alor1247-munas', 'utiʔ'), ('vagina', 'alor1247-pandai', 'pukiŋ'), ('vagina', 'alor1247-munas', 'pukiŋ'), ('vagina', 'alor1247-besar', 'pukiŋ')}


Concept ‘to_go’ is different between Alorese and Lamaholot.

 Alorese innovated the following forms:
 {('to_go', 'alor1247-besar', 'mei')}
 {('to_go', 'alor1247-munas', 'kaiʔ')}
 One or more Lamaholot dialects innovated forms not found in Alorese:
 {('to_go', 'lama1277-lerek', 'paˈnav'), ('to_go', 'tetu1245-suai', 'ba:'), ('to_go', 'keda1252-leuba', 'pan'), ('to_go', 'sika1262-hewa', 'pano'), ('to_go', 'lama1277-kalik', 'ˈpana'), ('to_go', 'tetu1245-vique', 'ba:')}

  Just for clarity:
  There are also the following similarity classes shared between Alorese and LH.
  {('to_go', 'lama1277-adona', 'ˈnaʔi'), ('to_go', 'alor1247-pandai', 'rahi'), ('to_go', 'lama1277-lewoi', 'ˈnaʔi')}


Concept ‘judge’ is different between Alorese and Lamaholot.

 Alorese innovated the following forms:
 {('judge', 'alor1247-pandai', "p'resa")}
 {('judge', 'alor1247-besar', "ka'pala")}
 {('judge', 'alor1247-munas', "dewan 'adat")}
 One or more Lamaholot dialects innovated forms not found in Alorese:
 {('judge', 'lama1277-adona', 'ʔolɛ̃ ˈkwadʒũ')}
 {('judge', 'wers1238-marit', 'hakim'), ('judge', 'lama1277-lewoi', 'ˈhakim'), ('judge', 'kaer1234', "ha'kim"), ('judge', 'keda1252-leuba', "ha'kim"), ('judge', 'kelo1247-hopte', 'hakim')}


Concept ‘to_blow’ is different between Alorese and Lamaholot.

 One or more Lamaholot dialects innovated forms not found in Alorese:
 {('to_blow', 'lama1277-lewop', 'təkə')}
 {('to_blow', 'lama1277-pukau', 'dihẽ'), ('to_blow', 'lama1277-imulo', 'die'), ('to_blow', 'lama1277-lewuk', 'diji'), ('to_blow', 'lama1277-lamah', 'dihe'), ('to_blow', 'lama1277-kalik', 'diji'), ('to_blow', 'lama1277-horow', 'dihẽ'), ('to_blow', 'lama1277-lamal', 'die'), ('to_blow', 'lama1277-belan', 'didi'), ('to_blow', 'lama1277-dulhi', 'dihe'), ('to_blow', 'lama1277-lewot', 'dihe'), ('to_blow', 'lama1277-watan', 'dihẽ')}
 {('to_blow', 'lama1277-ritae', 'kəbuha'), ('to_blow', 'rett1240', 'ugapi'), ('to_blow', 'rett1240', 'gapi')}
 {('to_blow', 'lama1277-minga', 'purəs'), ('to_blow', 'wers1238-marit', "u'sas"), ('to_blow', 'wers1238-taram', 'usasi')}

  Just for clarity:
  There are also the following similarity classes shared between Alorese and LH.
  {('to_blow', 'adan1251-lawah', 'apaʔu'), ('to_blow', 'adan1251-otvai', '’apu'), ('to_blow', 'blag1240-nule', "u'pu"), ('to_blow', 'keda1252', 'puiʔ'), ('to_blow', 'lama1277-tanju', 'bu'), ('to_blow', 'sika1262-maume', 'prupi'), ('to_blow', 'keda1252-leuba', 'puiʔ'), ('to_blow', 'lama1277-kalik', 'pur'), ('to_blow', 'lama1277-lewok', 'pur'), ('to_blow', 'p-cent2245-abvd', '*upi'), ('to_blow', 'lama1277-waiba', 'bu'), ('to_blow', 'sarr1247-nule', 'puj'), ('to_blow', 'baka1276', 'opu'), ('to_blow', 'lama1277-ileap', 'puit'), ('to_blow', 'lama1277-wuake', 'puis'), ('to_blow', 'lama1277-lewom', 'bu'), ('to_blow', 'blag1240-kulij', 'up:uʔ'), ('to_blow', 'lama1277-bama', 'bu'), ('to_blow', 'lama1277-lamat', 'pu'), ('to_blow', 'sika1262-hewa', 'pərupi'), ('to_blow', 'sika1262-hewa', 'plupi'), ('to_blow', 'lama1277-lewoe', 'pui'), ('to_blow', 'laka1255', 'fu:'), ('to_blow', 'lama1277-paina', 'pura'), ('to_blow', 'alor1247-besar', 'pui'), ('to_blow', 'mamb1306', 'pua'), ('to_blow', 'nede1245', 'puja'), ('to_blow', 'lama1277-waiwa', 'bu'), ('to_blow', 'lama1277-merde', 'puit'), ('to_blow', 'lama1277-kiwan', 'puit'), ('to_blow', 'hama1240', 'apu'), ('to_blow', 'lama1277-lewog', 'bu'), ('to_blow', 'kafo1240', 'ɰɑpuɑ'), ('to_blow', 'kelo1247-hopte', "mi 'pu"), ('to_blow', 'lama1277-lewoi', 'buː'), ('to_blow', 'teiw1235', 'pui'), ('to_blow', 'lamm1241-westp', 'pusakai'), ('to_blow', 'lama1277-lamak', 'pui'), ('to_blow', 'alor1247-munas', 'puiʔk'), ('to_blow', 'pura1258', 'apu'), ('to_blow', 'alor1247-baran', 'pui'), ('to_blow', 'tuku1254', 'pɔ:'), ('to_blow', 'alor1247-pandai', 'pui'), ('to_blow', 'lama1277-mulan', 'bu'), ('to_blow', 'lama1277-baipi', 'bu'), ('to_blow', 'dein1238', 'pui'), ('to_blow', 'sarr1247-adiab', 'puj'), ('to_blow', 'lama1277-lewob', 'bu'), ('to_blow', 'kema1243', 'pu:'), ('to_blow', 'lamm1241-westp', 'pus:a'), ('to_blow', 'kelo1247-hopte', 'pu'), ('to_blow', 'lama1277-adona', 'ˈpuit'), ('to_blow', 'lamm1241-westp', 'pujaŋ'), ('to_blow', 'lama1277-botun', 'bu'), ('to_blow', 'lama1277-lerek', 'purəˈhəŋ')}


Concept ‘where’ is different between Alorese and Lamaholot.

 Alorese innovated the following forms:
 {('where', 'alor1247-pandai', 'ɔrga'), ('where', 'alor1247-munas', "r'ga"), ('where', 'alor1247-munas', "r'ga 'naŋ")}
 One or more Lamaholot dialects innovated forms not found in Alorese:
 {('where', 'lama1277-lewok', 'maga'), ('where', 'lama1277-lerek', 'tamo nəɡaʔ'), ('where', 'lama1277-lerek', 'mai ɡa?'), ('where', 'lama1277-minga', 'maga')}
 {('where', 'buna1278-bobon', 'tʃiɔ gɛnɛ'), ('where', 'buna1278-bobon', 'tɛro gɛne'), ('where', 'buna1278-bobon', 'tɔ gɛnɛ'), ('where', 'buna1278-suai', 'tɔ gɛnɛ'), ('where', 'lama1277-dulhi', 'takunai'), ('where', 'buna1278-suai', 'tɛro gɛne'), ('where', 'buna1278-suai', 'tʃiɔ gɛnɛ')}
 {('where', 'lama1277-lewog', 'gaʔe'), ('where', 'lama1277-bama', 'gaʔe'), ('where', 'lama1277-lewoi', 'ˈɡaʔɛ'), ('where', 'lama1277-pukau', 'gaʔe'), ('where', 'lama1277-lamat', 'nəgaʔe'), ('where', 'lama1277-kalik', 'ɡane')}

  Just for clarity:
  There are also the following similarity classes shared between Alorese and LH.
  {('where', 'lama1277-lerek', 'no ɡə'), ('where', 'alor1247-besar', "naŋga 'ʤafa"), ('where', 'lama1277-paina', 'nanaga'), ('where', 'lama1277-kalik', 'nagane'), ('where', 'lama1277-lewuk', 'nanaga'), ('where', 'lama1277-mulan', 'nəga'), ('where', 'alor1247-besar', "ɔrɔ 'naŋga"), ('where', 'alor1247-besar', 'naŋga'), ('where', 'alor1247-baran', "na'ŋga 'ɔrɔ")}
  {('where', 'lama1277-ileap', 'təga'), ('where', 'lama1277-imulo', 'kətəga'), ('where', 'lama1277-watan', 'digaku'), ('where', 'lama1277-lewom', 'təga'), ('where', 'lama1277-merde', 'təga'), ('where', 'lama1277-lamal', 'diga'), ('where', 'lama1277-tanju', 'təgaʔe'), ('where', 'lama1277-lewoe', 'deʔea'), ('where', 'lama1277-adona', 'dɜˈɡaku'), ('where', 'lamm1241-westp', 'taga'), ('where', 'lama1277-ritae', 'diga'), ('where', 'lama1277-botun', 'dəga'), ('where', 'lama1277-waiba', 'təga'), ('where', 'lama1277-lewot', 'ketega'), ('where', 'lama1277-lamah', 'təga'), ('where', 'lama1277-wuake', 'dəga'), ('where', 'lama1277-kalik', 'ɡa'), ('where', 'lama1277-kiwan', 'dəga'), ('where', 'alor1247-pandai', "ər'ga"), ('where', 'lama1277-lamak', 'dəga'), ('where', 'lama1277-lewop', 'kətega'), ('where', 'lama1277-belan', 'nəga'), ('where', 'lama1277-baipi', 'təga'), ('where', 'lama1277-lewob', 'tea'), ('where', 'lama1277-horow', 'təga'), ('where', 'lama1277-waiwa', 'təga')}


Concept ‘when’ is different between Alorese and Lamaholot.

 One or more Lamaholot dialects innovated forms not found in Alorese:
 {('when', 'lama1277-paina', 'naoga')}
 {('when', 'lama1277-lewob', 'boʔã')}

  Just for clarity:
  There are also the following similarity classes shared between Alorese and LH.
  {('when', 'lama1277-lewoi', 'ərəŋ ˈpira'), ('when', 'lama1277-botun', 'ər pira'), ('when', 'lama1277-tanju', 'arəmpira'), ('when', 'lama1277-kalik', 'ˈʤəm(a) ˈpira'), ('when', 'idat1237', "a'lira"), ('when', 'tetu1245-vique', "wai'hira"), ('when', 'tetu1245-suai', "wai'hira"), ('when', 'lama1277-ileap', 'arampira'), ('when', 'lama1277-kiwan', 'ər pira'), ('when', 'kema1243', ",bɑi 'pila"), ('when', 'lama1277-mulan', 'arapira'), ('when', 'sika1262-hewa', 'rəmapira'), ('when', 'lama1277-baipi', 'arəmpira'), ('when', 'lama1277-lewuk', 'jəm pira'), ('when', 'idat1237', 'walira'), ('when', 'lama1277-bama', 'arəmpira'), ('when', 'alor1247-munas', "ɛr'pira"), ('when', 'lama1277-kalik', 'jəm pira'), ('when', 'alor1247-baran', "ɛrpɛ'hɛlɛ"), ('when', 'lama1277-lewop', 'jəmpira'), ('when', 'lama1277-watan', 'ərəm pira'), ('when', 'lama1277-lewom', 'arapira'), ('when', 'lama1277-wuake', 'ar pira'), ('when', 'sika1262-hewa', 'rəma pira'), ('when', 'lama1277-lerek', 'ˈdʒ͡əma ˈpira'), ('when', 'laka1255', "wɑr 'ʔila"), ('when', 'lama1277-horow', 'ərə̃m pira'), ('when', 'lama1277-lamah', 'arapira'), ('when', 'lama1277-lamak', 'arəmpira'), ('when', 'tetu1246', "bɑin 'hira"), ('when', 'alor1247-besar', "ɛrə 'pira"), ('when', 'mamb1306', 'ɑrpil'), ('when', 'lama1277-pukau', 'arəmpira'), ('when', 'lama1277-minga', 'jəma pira'), ('when', 'lama1277-lewog', 'arəmpira'), ('when', 'lama1277-lamat', 'arapirə'), ('when', 'lama1277-lewoe', 'erepirə'), ('when', 'lama1277-dulhi', 'ərən pira'), ('when', 'lama1277-merde', 'ar-apira'), ('when', 'lama1277-belan', 'arapira'), ('when', 'lama1277-adona', 'ˈʔɜrɜˈpira'), ('when', 'alor1247-pandai', "ari 'pira"), ('when', 'tuku1254', "ʔɑi 'pil"), ('when', 'lama1277-waiba', 'ərəmpira'), ('when', 'lama1277-lewok', 'jəm pira'), ('when', 'lama1277-ritae', 'ərəmpira'), ('when', 'lama1277-lamal', 'arapira'), ('when', 'sika1262-maume', 'rema pira'), ('when', 'lama1277-lewot', 'jəmapira'), ('when', 'lama1277-imulo', 'jəm pira'), ('when', 'lama1277-waiwa', 'ərəmpira')}


Concept ‘knife’ is different between Alorese and Lamaholot.

 Alorese shares the following forms with other AN languanges, but not Lamaholot:
 {('knife', 'kelo1247-bring', 'dʊ:r'), ('knife', 'adan1251-otvai', '’duir'), ('knife', 'alor1247-baran', "du'ri"), ('knife', 'kafo1240', 'dʊr'), ('knife', 'alor1247-pandai', 'duri'), ('knife', 'alor1247-besar', 'duri'), ('knife', 'kabo1247', 'dur'), ('knife', 'adan1251-otvai', '’dir'), ('knife', 'kuii1253', 'dur'), ('knife', 'alor1247-munas', 'duriʔ'), ('knife', 'hama1240', 'du.ir'), ('knife', 'kelo1247-hopte', 'du:r'), ('knife', 'nede1245', 'ʧija'), ('knife', 'adan1251-lawah', 'duir'), ('knife', 'mamb1306', 'nɪr')}
 One or more Lamaholot dialects innovated forms not found in Alorese:
 {('knife', 'laka1255', 'keru'), ('knife', 'lama1277-lerek', 'ˈɡerut')}
 {('knife', 'keda1252-leuba', 'mɛiʔ'), ('knife', 'lama1277-kalik', 'məˈrit')}
 {('knife', 'lama1277-lewoi', 'ˈhɛpɛʔ'), ('knife', 'lama1277-adona', 'hɛpɛʔ')}


Concept ‘to_pray’ is different between Alorese and Lamaholot.

 Alorese innovated the following forms:
 {('to_pray', 'alor1247-pandai', "pudʒi p'lɛwaŋ"), ('to_pray', 'alor1247-pandai', "pudʒi 'pakaŋ")}
 {('to_pray', 'alor1247-besar', 'doa')}
 {('to_pray', 'alor1247-munas', "gamar 'apa")}
 One or more Lamaholot dialects innovated forms not found in Alorese:
 {('to_pray', 'lama1277-kalik', 'məˈŋaʤi')}
 {('to_pray', 'lama1277-adona', 'sɜˈbajã'), ('to_pray', 'lama1277-lewoi', 'səmˈbajaŋ'), ('to_pray', 'pura1258', "sam'baɟaŋ"), ('to_pray', 'sawi1256', "sam'bai"), ('to_pray', 'pura1258', "sem'baɟaŋ"), ('to_pray', 'wers1238-marit', "sam'bai"), ('to_pray', 'lama1277-lerek', 'həˈbaja')}


Concept ‘husband’ is different between Alorese and Lamaholot.

 One or more Lamaholot dialects innovated forms not found in Alorese:
 {('husband', 'lama1277-lewok', 'awaga lame'), ('husband', 'lama1277-lewot', 'atan lame'), ('husband', 'lama1277-kalik', 'aˈvan laˈme'), ('husband', 'sika1262-tanai', 'ata laʔi')}
 {('husband', 'lama1277-lewop', 'afan'), ('husband', 'p-mala1545-abvd', '*qasawa'), ('husband', 'lama1277-lamat', 'hawã'), ('husband', 'lama1277-lewuk', 'awan'), ('husband', 'p-cent2245-abvd', '*qasawa'), ('husband', 'lama1277-kalik', 'awan')}
 {('husband', 'lama1277-belan', 'ha'), ('husband', 'lama1277-kiwan', 'hak (kalake)'), ('husband', 'lama1277-watan', 'hã'), ('husband', 'lama1277-lamah', 'hakã'), ('husband', 'lama1277-ileap', 'hān')}

  Just for clarity:
  There are also the following similarity classes shared between Alorese and LH.
  {('husband', 'mamb1306', 'lɑi'), ('husband', 'alor1247-munas', "k'lake"), ('husband', 'lama1277-tanju', 'lake'), ('husband', 'lama1277-botun', 'bəlikĩ'), ('husband', 'lama1277-paina', 'lakin'), ('husband', 'lama1277-waiba', 'lakẽ'), ('husband', 'lama1277-lamak', 'bəlakeʔ'), ('husband', 'lama1277-lewoi', 'ˈlake'), ('husband', 'sika1262-hewa', 'laʔi'), ('husband', 'tetu1246', 'lɑin'), ('husband', 'kema1243', 'lair'), ('husband', 'laka1255', 'lain'), ('husband', 'alor1247-pandai', "k'lake"), ('husband', 'lama1277-lamal', 'kəlake'), ('husband', 'lama1277-lewob', 'kəlake'), ('husband', 'lama1277-baipi', 'lakẽ'), ('husband', 'lama1277-bama', 'lake'), ('husband', 'lama1277-mulan', 'kəlake'), ('husband', 'lama1277-kiwan', 'hak (kalake)'), ('husband', 'lama1277-lewog', 'lake'), ('husband', 'lama1277-wuake', 'kəlake'), ('husband', 'lama1277-imulo', 'kəlake'), ('husband', 'lama1277-waiwa', 'kəlake'), ('husband', 'sika1262-hewa', 'lait'), ('husband', 'fata1247', "ɛ'lɛhi"), ('husband', 'alor1247-munas', "k'lake 'nara"), ('husband', 'alor1247-besar', "ka'lake"), ('husband', 'lama1277-lerek', 'lakin'), ('husband', 'sika1262-maume', 'laʔi'), ('husband', 'tuku1254', 'laki'), ('husband', 'lama1277-horow', 'lake'), ('husband', 'lama1277-pukau', 'kakehe'), ('husband', 'lama1277-adona', 'ˈlakhẽ'), ('husband', 'lama1277-lewoe', 'təlaʔe'), ('husband', 'alor1247-baran', "ka'lake"), ('husband', 'lama1277-dulhi', 'kəlake'), ('husband', 'lama1277-lewom', 'lake'), ('husband', 'lama1277-ritae', 'lake'), ('husband', 'lama1277-minga', 'analaki'), ('husband', 'lama1277-merde', 'kəbəlake')}


Concept ‘soil’ is different between Alorese and Lamaholot.

 Alorese innovated the following forms:
 {('soil', 'alor1247-besar', "fa'raha")}

  Just for clarity:
  There are also the following similarity classes shared between Alorese and LH.
  {('soil', 'sika1262-hewa', 'tana'), ('soil', 'lama1277-kalik', 'əˈnan'), ('soil', 'kafo1240', 'anɑi'), ('soil', 'abui1241-takal', "ä.'ne̞i"), ('soil', 'abui1241-ulaga', 'anei'), ('soil', 'lama1277-adona', 'tana'), ('soil', 'lama1277-lewoi', 'ˈtana'), ('soil', 'kama1365', 'dane'), ('soil', 'lama1277-kalik', 'əˈna'), ('soil', 'atim1239', 'anai'), ('soil', 'p-mala1545-acd', '*taneq'), ('soil', 'abui1241-fuime', 'anei'), ('soil', 'alor1247-pandai', 'tana'), ('soil', 'lama1277-lerek', 'ˈanai'), ('soil', 'alor1247-munas', 'tana')}


Concept ‘sugar_palm’ is different between Alorese and Lamaholot.

 One or more Lamaholot dialects innovated forms not found in Alorese:
 {('sugar_palm', 'lama1277-kalik', 'ˈkebɔl'), ('sugar_palm', 'lama1277-lerek', 'ˈkebol'), ('sugar_palm', 'lama1277-lewoi', 'ˈkɛbɔ')}

  Just for clarity:
  There are also the following similarity classes shared between Alorese and LH.
  {('sugar_palm', 'alor1247-pandai', 'pɔla'), ('sugar_palm', 'alor1247-munas', "pɔla 'pukɔŋ"), ('sugar_palm', 'keda1252-leuba', "pɔ'le"), ('sugar_palm', 'lama1277-adona', 'ˈpolaʔ')}


Concept ‘to_fold’ is different between Alorese and Lamaholot.

 Alorese shares the following forms with TAP languages:
 {('to_fold', 'alor1247-pandai', 'lakuk'), ('to_fold', 'kula1280-lanto', 'lakup'), ('to_fold', 'sawi1256', "la'kupi"), ('to_fold', 'kula1280-lanto', 'la’kup')}

  Just for clarity:
  There are also the following similarity classes shared between Alorese and LH.
  {('to_fold', 'alor1247-pandai', "la'pːɛ"), ('to_fold', 'sika1262-hewa', 'ləpet'), ('to_fold', 'lama1277-lewoi', 'ˈləpək'), ('to_fold', 'lama1277-kalik', 'ləˈpət'), ('to_fold', 'alor1247-munas', "lə'pɛʔ"), ('to_fold', 'baka1276', "le'pet"), ('to_fold', 'keda1252-leuba', "lɛ'piʔ"), ('to_fold', 'alor1247-besar', 'lapɛ '), ('to_fold', 'blag1240-kulij', 'lepet'), ('to_fold', 'blag1240-nule', "le'pet"), ('to_fold', 'p-mala1545-acd', '*lipet'), ('to_fold', 'lama1277-adona', 'ˈlɜpɜt'), ('to_fold', 'lama1277-lerek', 'ˈləpət')}


Concept ‘to_command’ is different between Alorese and Lamaholot.

 Alorese innovated the following forms:
 {('to_command', 'alor1247-munas', 'gahiŋ'), ('to_command', 'pura1258', "iŋ'gahiŋ"), ('to_command', 'rett1240', 'gahiŋ'), ('to_command', 'alor1247-besar', 'gahĩŋ'), ('to_command', 'alor1247-pandai', 'gahiŋ')}
 {('to_command', 'alor1247-pandai', "kə'rːa")}
 Alorese shares the following forms with TAP languages:
 {('to_command', 'alor1247-munas', 'gahiŋ'), ('to_command', 'pura1258', "iŋ'gahiŋ"), ('to_command', 'rett1240', 'gahiŋ'), ('to_command', 'alor1247-besar', 'gahĩŋ'), ('to_command', 'alor1247-pandai', 'gahiŋ')}
 {('to_command', 'alor1247-pandai', "kə'rːa")}
 One or more Lamaholot dialects innovated forms not found in Alorese:
 {('to_command', 'lama1277-lerek', 'koˈmando')}
 {('to_command', 'lama1277-adona', 'ˈhudaʔ'), ('to_command', 'lama1277-lewoi', 'ˈhudaʔ')}
 {('to_command', 'sika1262-hewa', 'plɛta'), ('to_command', 'lama1277-kalik', 'ˈpreta'), ('to_command', 'lama1277-lerek', 'pərinta')}


Concept ‘lips’ is different between Alorese and Lamaholot.

 Alorese shares the following forms with other AN languanges, but not Lamaholot:
 {('lips', 'alor1247-munas', 'wiwiʔ'), ('lips', 'alor1247-baran', 'fifiŋ'), ('lips', 'sika1262-hewa', 'ʋiʋir'), ('lips', 'alor1247-besar', 'fifiŋ'), ('lips', 'p-mala1545-acd', '*bibiR'), ('lips', 'alor1247-pandai', 'wiwiŋ'), ('lips', 'alor1247-munas', 'wiwiŋ'), ('lips', 'keda1252-leuba', 'wiwir')}
 One or more Lamaholot dialects innovated forms not found in Alorese:
 {('lips', 'lama1277-adona', 'ˈnuhuˈkɜt'), ('lips', 'tetu1245-vique', "ibun 'suhun"), ('lips', 'tetu1245-suai', "ibun 'suhun"), ('lips', 'lama1277-lewoi', 'ˈnuhuŋ')}
 {('lips', 'lama1277-lerek', 'ˈnuhə ləˈrit')}


Concept ‘battle’ is different between Alorese and Lamaholot.

 Alorese innovated the following forms:
 {('battle', 'alor1247-munas', "nuhɔ 'kataŋ"), ('battle', 'alor1247-pandai', 'nuhɔ')}
 {('battle', 'alor1247-besar', "pə'raŋ")}
 One or more Lamaholot dialects innovated forms not found in Alorese:
 {('battle', 'lama1277-lewoi', 'ˈtupaʔ')}
 {('battle', 'lama1277-adona', 'pɜˈwuno'), ('battle', 'maka1316', 'funu'), ('battle', 'tetu1245-suai', 'funu'), ('battle', 'tetu1245-vique', 'funu')}


Concept ‘body’ is different between Alorese and Lamaholot.

 Alorese innovated the following forms:
 {('body', 'alor1247-munas', 'badaŋ'), ('body', 'alor1247-munas', 'badak'), ('body', 'alor1247-pandai', 'odaŋ')}

  Just for clarity:
  There are also the following similarity classes shared between Alorese and LH.
  {('body', 'lama1277-lewoi', 'ˈwəkiŋ'), ('body', 'lama1277-adona', 'ˈwɜkiˈkɜt'), ('body', 'sika1262-hewa', 'ʋiʔin'), ('body', 'alor1247-pandai', "wə'kːiŋ"), ('body', 'lama1277-kalik', 'vəˈki naˈvak'), ('body', 'alor1247-besar', 'fakiŋ'), ('body', 'lama1277-lerek', 'naˈwak')}


Concept ‘to_whisper’ is different between Alorese and Lamaholot.

 Alorese shares the following forms with TAP languages:
 {('to_whisper', 'adan1251-otvai', '’tamaŋ'), ('to_whisper', 'alor1247-pandai', "t'maga"), ('to_whisper', 'alor1247-munas', 'maga'), ('to_whisper', 'kula1280-lanto', 'ma’gisa'), ('to_whisper', 'fata1247', "ma'kase"), ('to_whisper', 'alor1247-besar', "ta'maga")}
 One or more Lamaholot dialects innovated forms not found in Alorese:
 {('to_whisper', 'lama1277-lerek', 'keˈhakok'), ('to_whisper', 'lama1277-lewoi', 'ˈkəsakɔ')}
 {('to_whisper', 'lama1277-adona', 'hakori')}
 {('to_whisper', 'lama1277-kalik', 'krəˈŋav')}


Concept ‘smooth’ is different between Alorese and Lamaholot.

 Alorese innovated the following forms:
 {('smooth', 'alor1247-pandai', 'mɛmɛŋ')}
 One or more Lamaholot dialects innovated forms not found in Alorese:
 {('smooth', 'lama1277-lerek', 'kəˈmel-kəˈmel')}

  Just for clarity:
  There are also the following similarity classes shared between Alorese and LH.
  {('smooth', 'alor1247-munas', "b'lɔhɔʔ"), ('smooth', 'lama1277-lewoi', 'kəˈlɔhɔ'), ('smooth', 'lama1277-kalik', 'ɡəməˈlɔs')}
  {('smooth', 'lama1277-lerek', 'kəmeluˈtən'), ('smooth', 'sika1262-hewa', 'mɛlur'), ('smooth', 'alor1247-pandai', 'mɛluk'), ('smooth', 'lama1277-adona', 'kɜˈmelut'), ('smooth', 'keda1252-leuba', "mɛ'luʔ"), ('smooth', 'alor1247-munas', 'mɛluk'), ('smooth', 'alor1247-besar', "ha'melu")}


Concept ‘to_plant_yam’ is different between Alorese and Lamaholot.

 One or more Lamaholot dialects innovated forms not found in Alorese:
 {('to_plant_yam', 'lama1277-adona', 'ˈpahaʔ'), ('to_plant_yam', 'sika1262-hewa', 'pahe')}

  Just for clarity:
  There are also the following similarity classes shared between Alorese and LH.
  {('to_plant_yam', 'alor1247-munas', "mula 'kura "), ('to_plant_yam', 'p-aust1307-abvd', '*mula'), ('to_plant_yam', 'alor1247-pandai', "mula 'kura "), ('to_plant_yam', 'sika1262-hewa', 'mula'), ('to_plant_yam', 'lama1277-kalik', 'mul'), ('to_plant_yam', 'p-mala1545-abvd', '*mula'), ('to_plant_yam', 'lama1277-lewoi', 'mula ˈuwe'), ('to_plant_yam', 'alor1247-besar', "mula 'kura 'ʤafa"), ('to_plant_yam', 'lama1277-lerek', 'ˈmula ˈhurar')}


Concept ‘left_side’ is different between Alorese and Lamaholot.

 Alorese innovated the following forms:
 {('left_side', 'alor1247-munas', 'hɛkɛŋ'), ('left_side', 'alor1247-pandai', 'hɛkɛŋ'), ('left_side', 'alor1247-besar', 'hɛkɛ̃')}
 One or more Lamaholot dialects innovated forms not found in Alorese:
 {('left_side', 'sika1262-maume', 'viri'), ('left_side', 'keda1252-leuwa', 'vεri'), ('left_side', 'sarr1247-nule', 'xawar'), ('left_side', 'sika1262-hewa', 'wirin'), ('left_side', 'sika1262-tanai', 'βiri'), ('left_side', 'p-mala1545-acd', '*ka-wiRi'), ('left_side', 'p-mala1545-abvd', '*ka-wiɾi'), ('left_side', 'p-mala1545-acd', '*wiRi'), ('left_side', 'p-aust1307-abvd', '*ka-wiʀi'), ('left_side', 'lama1277-lamat', 'wiri'), ('left_side', 'p-cent2245-abvd', '*ka-wiʀi'), ('left_side', 'lama1277-pukau', 'wiri'), ('left_side', 'keda1252-leuba', 'weri'), ('left_side', 'lama1277-lewoe', 'wirĩ'), ('left_side', 'keda1252', 'weri'), ('left_side', 'sika1262-hewa', 'ʋirin'), ('left_side', 'sarr1247-adiab', "xa'war")}

  Just for clarity:
  There are also the following similarity classes shared between Alorese and LH.
  {('left_side', 'lama1277-mulan', 'neki'), ('left_side', 'lama1277-paina', 'heken'), ('left_side', 'lama1277-tanju', 'nekĩ'), ('left_side', 'lama1277-baipi', 'nekĩ'), ('left_side', 'lama1277-lamah', 'meki'), ('left_side', 'lama1277-lerek', 'hɛˈkɛn'), ('left_side', 'lama1277-adona', 'nekeʔ'), ('left_side', 'alor1247-baran', 'mekiŋ'), ('left_side', 'lama1277-lewoi', 'ˈnɛkiŋ'), ('left_side', 'lama1277-minga', 'səmeki'), ('left_side', 'lama1277-horow', 'nekiŋ'), ('left_side', 'lama1277-belan', 'seki'), ('left_side', 'lama1277-waiba', 'nekĩ'), ('left_side', 'lama1277-lewok', 'seke'), ('left_side', 'lama1277-bama', 'nekĩ'), ('left_side', 'lama1277-lewog', 'nekĩ'), ('left_side', 'lama1277-ileap', 'mekin'), ('left_side', 'lama1277-dulhi', 'nekĩ'), ('left_side', 'lama1277-lewom', 'nekĩ'), ('left_side', 'lama1277-lamak', 'nekĩ'), ('left_side', 'lama1277-lewuk', 'seke'), ('left_side', 'lama1277-ritae', 'nekĩ'), ('left_side', 'lama1277-watan', 'nekĩ'), ('left_side', 'lama1277-kalik', 'seˈke'), ('left_side', 'lama1277-merde', 'meki'), ('left_side', 'lama1277-lewob', 'mekĩ'), ('left_side', 'lama1277-adona', 'ˈnɛkĩ'), ('left_side', 'lama1277-lewop', 'heke'), ('left_side', 'lama1277-lamal', 'meki'), ('left_side', 'lama1277-botun', 'nekĩ'), ('left_side', 'lama1277-imulo', 'səmekiŋən'), ('left_side', 'lama1277-waiwa', 'nekĩ'), ('left_side', 'lama1277-kiwan', 'nekin'), ('left_side', 'lama1277-wuake', 'səmekiŋ'), ('left_side', 'lama1277-lewot', 'həmekiŋən'), ('left_side', 'lama1277-kalik', 'seke')}


Concept ‘fence’ is different between Alorese and Lamaholot.

 Alorese innovated the following forms:
 {('fence', 'alor1247-munas', "laka-'lakaʔ")}
 One or more Lamaholot dialects innovated forms not found in Alorese:
 {('fence', 'lama1277-kalik', 'kaˈdal')}

  Just for clarity:
  There are also the following similarity classes shared between Alorese and LH.
  {('fence', 'lama1277-lerek', 'ˈnihar'), ('fence', 'sika1262-hewa', 'niha'), ('fence', 'keda1252-leuba', "ni'hɛ"), ('fence', 'lama1277-lewoi', 'ˈniha'), ('fence', 'lama1277-adona', 'niha'), ('fence', 'alor1247-pandai', 'niha'), ('fence', 'alor1247-besar', "ni'ha")}


Concept ‘shy_ashamed’ is different between Alorese and Lamaholot.

 One or more Lamaholot dialects innovated forms not found in Alorese:
 {('shy_ashamed', 'lama1277-kalik', 'kruˈit'), ('shy_ashamed', 'lama1277-lerek', 'kruˈit')}

  Just for clarity:
  There are also the following similarity classes shared between Alorese and LH.
  {('shy_ashamed', 'sarr1247-nule', 'menija:ŋ'), ('shy_ashamed', 'rett1240', 'maniaŋ'), ('shy_ashamed', 'alor1247-besar', 'maniaŋ'), ('shy_ashamed', 'teiw1235', "mani'aŋ"), ('shy_ashamed', 'alor1247-munas', "m'niaŋ"), ('shy_ashamed', 'lamm1241-westp', 'maniaŋ'), ('shy_ashamed', 'baka1276', "mi'niaŋ"), ('shy_ashamed', 'blag1240-nule', "mə'nijaŋ"), ('shy_ashamed', 'lama1277-lewoi', 'ˈmia'), ('shy_ashamed', 'blag1240-kulij', 'mənijaŋ'), ('shy_ashamed', 'kuii1253', 'manian'), ('shy_ashamed', 'alor1247-pandai', "m'niaŋ"), ('shy_ashamed', 'lama1277-adona', 'ˈmia')}


Concept ‘empty’ is different between Alorese and Lamaholot.

 One or more Lamaholot dialects innovated forms not found in Alorese:
 {('empty', 'lama1277-kalik', 'məˈliŋ')}

  Just for clarity:
  There are also the following similarity classes shared between Alorese and LH.
  {('empty', 'keda1252-leuba', 'mɛtuŋ'), ('empty', 'tetu1245-vique', 'mamuk'), ('empty', 'lama1277-lewoi', 'aˈmuʔuŋ'), ('empty', 'alor1247-besar', "a'mukuŋ"), ('empty', 'alor1247-munas', 'amuk'), ('empty', 'alor1247-pandai', 'amuk'), ('empty', 'lama1277-adona', 'ˈʔaʔmũ'), ('empty', 'lama1277-lerek', 'amuˈkən'), ('empty', 'tetu1245-suai', 'mamuk')}


Concept ‘spear’ is different between Alorese and Lamaholot.

 Alorese innovated the following forms:
 {('spear', 'adan1251-lawah', 'boko'), ('spear', 'adan1251-otvai', '’bɔk'), ('spear', 'alor1247-besar', 'bɔka')}
 {('spear', 'blag1240-tuntu', 'qaba'), ('spear', 'sarr1247-nule', 'qab'), ('spear', 'p-timo1261', '*qaba(k)'), ('spear', 'kafo1240', 'kɑfɑkɑ'), ('spear', 'blag1240-nule', 'ʔaba'), ('spear', 'sarr1247-adiab', 'qab'), ('spear', 'kabo1247', 'kaba'), ('spear', 'abui1241-takal', "kä.'fäk"), ('spear', 'dein1238', 'qab'), ('spear', 'kama1365', 'kapa'), ('spear', 'blag1240-bama', 'qaba'), ('spear', 'kuii1253', 'kabak'), ('spear', 'kaer1234', "qa'bi"), ('spear', 'alor1247-pandai', 'rapaŋ'), ('spear', 'adan1251-otvai', '’aba'), ('spear', 'blag1240-warsa', 'qaba'), ('spear', 'atim1239', 'kafak'), ('spear', 'kelo1247-hopte', "kə'bak"), ('spear', 'alor1247-munas', 'rapaŋ'), ('spear', 'p-alor1249', '*qaba(k)'), ('spear', 'blag1240-kulij', 'rapaŋ'), ('spear', 'kira1248', 'kaˈbak'), ('spear', 'nede1245', 'kab:a'), ('spear', 'baka1276', 'rapaŋ'), ('spear', 'kelo1247-bring', 'kəbɑk'), ('spear', 'blag1240-warsa', 'xaba'), ('spear', 'lamm1241-westp', 'kab:i'), ('spear', 'hama1240', 'aba'), ('spear', 'teiw1235', 'qab')}
 {('spear', 'alor1247-munas', "kɛ'laleiŋ")}
 {('spear', 'alor1247-pandai', 'turaiŋ'), ('spear', 'alor1247-munas', "tuŋ'raiŋ")}
 Alorese shares the following forms with TAP languages:
 {('spear', 'adan1251-lawah', 'boko'), ('spear', 'adan1251-otvai', '’bɔk'), ('spear', 'alor1247-besar', 'bɔka')}
 {('spear', 'blag1240-tuntu', 'qaba'), ('spear', 'sarr1247-nule', 'qab'), ('spear', 'p-timo1261', '*qaba(k)'), ('spear', 'kafo1240', 'kɑfɑkɑ'), ('spear', 'blag1240-nule', 'ʔaba'), ('spear', 'sarr1247-adiab', 'qab'), ('spear', 'kabo1247', 'kaba'), ('spear', 'abui1241-takal', "kä.'fäk"), ('spear', 'dein1238', 'qab'), ('spear', 'kama1365', 'kapa'), ('spear', 'blag1240-bama', 'qaba'), ('spear', 'kuii1253', 'kabak'), ('spear', 'kaer1234', "qa'bi"), ('spear', 'alor1247-pandai', 'rapaŋ'), ('spear', 'adan1251-otvai', '’aba'), ('spear', 'blag1240-warsa', 'qaba'), ('spear', 'atim1239', 'kafak'), ('spear', 'kelo1247-hopte', "kə'bak"), ('spear', 'alor1247-munas', 'rapaŋ'), ('spear', 'p-alor1249', '*qaba(k)'), ('spear', 'blag1240-kulij', 'rapaŋ'), ('spear', 'kira1248', 'kaˈbak'), ('spear', 'nede1245', 'kab:a'), ('spear', 'baka1276', 'rapaŋ'), ('spear', 'kelo1247-bring', 'kəbɑk'), ('spear', 'blag1240-warsa', 'xaba'), ('spear', 'lamm1241-westp', 'kab:i'), ('spear', 'hama1240', 'aba'), ('spear', 'teiw1235', 'qab')}
 {('spear', 'alor1247-munas', "kɛ'laleiŋ")}
 {('spear', 'alor1247-pandai', 'turaiŋ'), ('spear', 'alor1247-munas', "tuŋ'raiŋ")}
 One or more Lamaholot dialects innovated forms not found in Alorese:
 {('spear', 'lama1277-adona', 'ɡalaʔ'), ('spear', 'lama1277-lerek', 'ˈɡalar'), ('spear', 'lama1277-kalik', 'ɡaˈla'), ('spear', 'lama1277-kalik', 'ɡaˈlar'), ('spear', 'keda1252-leuba', 'kala'), ('spear', 'lama1277-lewoi', 'ˈɡala')}


Concept ‘to_rub’ is different between Alorese and Lamaholot.

 Alorese shares the following forms with other AN languanges, but not Lamaholot:
 {('to_rub', 'alor1247-pandai', 'dɔhɔ'), ('to_rub', 'tetu1245-vique', 'dɔkɔ'), ('to_rub', 'alor1247-besar', 'dɔhɔ'), ('to_rub', 'blag1240-kulij', 'dohoʔ'), ('to_rub', 'baka1276', 'doho'), ('to_rub', 'alor1247-baran', "dɔ'h:ɔ"), ('to_rub', 'alor1247-munas', 'dɔhɔʔ'), ('to_rub', 'tetu1245-suai', 'dɔkɔ')}
 One or more Lamaholot dialects innovated forms not found in Alorese:
 {('to_rub', 'lama1277-minga', 'godo'), ('to_rub', 'lama1277-wuake', 'godo'), ('to_rub', 'lama1277-imulo', 'odo'), ('to_rub', 'lama1277-lewot', 'odo'), ('to_rub', 'lama1277-lewop', 'godoŋ')}
 {('to_rub', 'blag1240-warsa', 'osoŋ'), ('to_rub', 'rett1240', 'osoŋ'), ('to_rub', 'lama1277-waiba', 'rosuk'), ('to_rub', 'blag1240-tuntu', 'osoŋ'), ('to_rub', 'blag1240-bama', 'osoŋ'), ('to_rub', 'kaer1234', "o'soŋ"), ('to_rub', 'lama1277-lamak', 'oso'), ('to_rub', 'lama1277-watan', 'oso'), ('to_rub', 'rett1240', 'ɔsɔŋ'), ('to_rub', 'pura1258', 'osoŋ'), ('to_rub', 'blag1240-nule', 'osoŋ')}
 {('to_rub', 'keda1252-leuba', "pɔ'hɔʔ"), ('to_rub', 'keda1252', 'poho'), ('to_rub', 'sika1262-tanai', 'bloso'), ('to_rub', 'lama1277-ritae', 'puʔũ'), ('to_rub', 'lama1277-lerek', 'pohoˈkəŋ'), ('to_rub', 'sika1262-hewa', 'beroso'), ('to_rub', 'p-mala1545-acd', '*usuq'), ('to_rub', 'sika1262-tanai', 'boho'), ('to_rub', 'lama1277-kiwan', 'pusak'), ('to_rub', 'sika1262-hewa', 'blosok')}
 {('to_rub', 'lamm1241-westp', 'toru'), ('to_rub', 'lama1277-mulan', 'doru'), ('to_rub', 'lama1277-lewoe', 'doru'), ('to_rub', 'lama1277-tanju', 'doru'), ('to_rub', 'lama1277-dulhi', 'doruk'), ('to_rub', 'lama1277-lewok', 'doru'), ('to_rub', 'lama1277-kalik', 'doru'), ('to_rub', 'lama1277-lewob', 'doru'), ('to_rub', 'lama1277-lamat', 'doru'), ('to_rub', 'lama1277-lewuk', 'doru'), ('to_rub', 'lama1277-lamal', 'doru'), ('to_rub', 'lama1277-waiwa', 'doru'), ('to_rub', 'lama1277-merde', 'doru'), ('to_rub', 'lama1277-horow', 'doruk'), ('to_rub', 'lama1277-baipi', 'doru'), ('to_rub', 'lama1277-adona', 'doˈruʔ'), ('to_rub', 'lama1277-belan', 'doru'), ('to_rub', 'lama1277-paina', 'dore'), ('to_rub', 'keda1252-leuwa', 'dɔru'), ('to_rub', 'lama1277-kalik', 'ˈdoru'), ('to_rub', 'lama1277-lewog', 'doruk'), ('to_rub', 'lama1277-pukau', 'doru'), ('to_rub', 'lama1277-lewom', 'doru'), ('to_rub', 'lama1277-botun', 'doruk'), ('to_rub', 'lama1277-ileap', 'doru'), ('to_rub', 'lama1277-lewoi', 'ˈdɔruʔ'), ('to_rub', 'lama1277-bama', 'doru'), ('to_rub', 'lama1277-lamah', 'doru')}


Concept ‘smoke’ is different between Alorese and Lamaholot.

 One or more Lamaholot dialects innovated forms not found in Alorese:
 {('smoke', 'lama1277-lerek', 'ap ˈnunan'), ('smoke', 'lama1277-paina', 'nunan')}
 {('smoke', 'lama1277-lewop', 'negur'), ('smoke', 'lama1277-lewot', 'hənəgur'), ('smoke', 'lama1277-lamal', 'sənəgur'), ('smoke', 'lama1277-kalik', 'sənəgur'), ('smoke', 'lama1277-minga', 'sənəgur'), ('smoke', 'lama1277-belan', 'sənəgur'), ('smoke', 'lama1277-lewuk', 'sənəgur'), ('smoke', 'lama1277-imulo', 'sənəgurən'), ('smoke', 'lama1277-kalik', 'snəˈɡur'), ('smoke', 'lama1277-lewok', 'sənəgur'), ('smoke', 'lama1277-wuake', 'sənəgur')}
 {('smoke', 'kafo1240', 'fonɑ'), ('smoke', 'fata1247', "aca 'tapin"), ('smoke', 'rett1240', 'bɔna'), ('smoke', 'kelo1247-hopte', "a'da 'bɔn"), ('smoke', 'dein1238', 'bu:n'), ('smoke', 'teiw1235', 'bu:n'), ('smoke', 'kelo1247-bring', 'ədɑbon'), ('smoke', 'sarr1247-adiab', '(ar) bu:n'), ('smoke', 'nede1245', 'arabun'), ('smoke', 'sarr1247-nule', 'har gon'), ('smoke', 'lama1277-ileap', 'bənu'), ('smoke', 'kama1365', 'punɑ')}

  Just for clarity:
  There are also the following similarity classes shared between Alorese and LH.
  {('smoke', 'lama1277-botun', 'nuhũ'), ('smoke', 'lama1277-lewoi', 'nuhũŋ'), ('smoke', 'lama1277-lewom', 'huhũ'), ('smoke', 'sika1262-tanai', 'nuhi-n'), ('smoke', 'lama1277-ritae', 'nuhu'), ('smoke', 'lama1277-pukau', 'nuhã'), ('smoke', 'lama1277-lewoe', 'nuhẽ'), ('smoke', 'alor1247-besar', "pa'nuhuŋ"), ('smoke', 'lama1277-horow', 'nuhu'), ('smoke', 'lama1277-mulan', 'nuhu'), ('smoke', 'alor1247-munas', "p'nuhuŋ"), ('smoke', 'lama1277-adona', 'ˈnuhũ'), ('smoke', 'lama1277-lamah', 'nuhu'), ('smoke', 'lama1277-kiwan', 'nuhũ'), ('smoke', 'lama1277-lamat', 'nuhẽ'), ('smoke', 'lama1277-lewog', 'nuhũ'), ('smoke', 'lama1277-waiwa', 'nuhu'), ('smoke', 'alor1247-baran', ",ape 'nɑhiŋ"), ('smoke', 'sika1262-hewa', 'nuhi'), ('smoke', 'lama1277-tanju', 'nuhu'), ('smoke', 'sika1262-maume', 'nuhin'), ('smoke', 'lama1277-watan', 'nuhũ'), ('smoke', 'lama1277-lewob', 'rihũ'), ('smoke', 'lama1277-dulhi', 'nuhũ'), ('smoke', 'lama1277-baipi', 'nuhũ'), ('smoke', 'lama1277-bama', 'nuhu'), ('smoke', 'alor1247-pandai', "pə'nuhuŋ"), ('smoke', 'lama1277-lamak', 'nuhuŋ'), ('smoke', 'lama1277-merde', 'nuhu'), ('smoke', 'lama1277-waiba', 'nuhu'), ('smoke', 'sika1262-hewa', '(api) nuhin')}


Concept ‘branch’ is different between Alorese and Lamaholot.

 Alorese innovated the following forms:
 {('branch', 'alor1247-munas', "kaʤɔ 'limaŋ"), ('branch', 'alor1247-besar', "kaʤɔ 'limaŋ"), ('branch', 'alor1247-pandai', "kaʤɔ 'limaŋ")}
 {('branch', 'blag1240-nule', "te kə'raŋa"), ('branch', 'alor1247-munas', "kaʤɔ k'raŋak"), ('branch', 'alor1247-pandai', "kaʤɔ kə'raŋak")}
 Alorese shares the following forms with TAP languages:
 {('branch', 'alor1247-munas', "kaʤɔ 'limaŋ"), ('branch', 'alor1247-besar', "kaʤɔ 'limaŋ"), ('branch', 'alor1247-pandai', "kaʤɔ 'limaŋ")}
 {('branch', 'blag1240-nule', "te kə'raŋa"), ('branch', 'alor1247-munas', "kaʤɔ k'raŋak"), ('branch', 'alor1247-pandai', "kaʤɔ kə'raŋak")}
 One or more Lamaholot dialects innovated forms not found in Alorese:
 {('branch', 'lama1277-kalik', 'viˈkil'), ('branch', 'lama1277-adona', 'ˈwikʔĩ'), ('branch', 'lama1277-lerek', 'ˈviɡit')}
 {('branch', 'abui1241-takal', "hä.'täŋ"), ('branch', 'teiw1235', 'tei getiŋi:'), ('branch', 'kula1280-lanto', 'a’tən'), ('branch', 'baka1276', 'ataŋ'), ('branch', 'blag1240-kulij', 'tei kraŋga'), ('branch', 'p-mala1545-acd', '*daqan'), ('branch', 'kira1248', '(atei) gawadin'), ('branch', 'kama1365', '-taŋ'), ('branch', 'blag1240-tuntu', 'te geteŋi'), ('branch', 'adan1251-lawah', 'ataŋ'), ('branch', 'pura1258', "te'Ɂataŋ"), ('branch', 'kula1280-lanto', 'geatɨna'), ('branch', 'p-cent2245-abvd', '*daqan'), ('branch', 'kaer1234', "tei gɛ'tɛni"), ('branch', 'abui1241-fuime', 'bata tawakiŋ'), ('branch', 'fata1247', 'kɛtɛ'), ('branch', 'p-mala1545-abvd', '*daqan'), ('branch', 'abui1241-ulaga', 'gawataŋ'), ('branch', 'rett1240', 'te gataŋ'), ('branch', 'blag1240-warsa', 'te gətini'), ('branch', 'rett1240', 'te: gataŋ'), ('branch', 'kelo1247-hopte', "ʔə'te gʔə'tan"), ('branch', 'blag1240-warsa', 'te gataŋ'), ('branch', 'lama1277-lewoi', 'ˈrɛtiŋ'), ('branch', 'blag1240-bama', 'geteni'), ('branch', 'wers1238-marit', "gə'teŋ"), ('branch', 'atim1239', 'ˈbata takawak'), ('branch', 'kabo1247', 'ataŋ'), ('branch', 'kuii1253', 'gatan'), ('branch', 'nede1245', 'gatani')}


Concept ‘to_weave’ is different between Alorese and Lamaholot.

 One or more Lamaholot dialects innovated forms not found in Alorese:
 {('to_weave', 'lama1277-lerek', 'ˈpano tan')}

  Just for clarity:
  There are also the following similarity classes shared between Alorese and LH.
  {('to_weave', 'lama1277-adona', 'tane'), ('to_weave', 'alor1247-munas', 'tane'), ('to_weave', 'lama1277-adona', 'nekɜt tane'), ('to_weave', 'alor1247-pandai', 'tane '), ('to_weave', 'alor1247-besar', "tane 'apa"), ('to_weave', 'lama1277-kalik', 'ˈtani'), ('to_weave', 'lama1277-lewoi', 'ˈtane'), ('to_weave', 'keda1252-leuba', "nekeʔ 'tan")}


Concept ‘clean’ is different between Alorese and Lamaholot.

 One or more Lamaholot dialects innovated forms not found in Alorese:
 {('clean', 'lama1277-lerek', 'bəliˈnau')}

  Just for clarity:
  There are also the following similarity classes shared between Alorese and LH.
  {('clean', 'alor1247-besar', 'lahiŋ'), ('clean', 'alor1247-munas', 'lahɛk'), ('clean', 'lama1277-adona', 'ˈlaɛʔ'), ('clean', 'lama1277-lewoi', 'ˈlaɛŋ'), ('clean', 'alor1247-pandai', 'lahɛŋ'), ('clean', 'lama1277-kalik', 'slaˈɛk'), ('clean', 'alor1247-munas', 'lahɛŋ')}


Concept ‘animal’ is different between Alorese and Lamaholot.

 One or more Lamaholot dialects innovated forms not found in Alorese:
 {('animal', 'lama1277-paina', 'əwaŋ ləku'), ('animal', 'lama1277-lerek', 'əˈvaŋ ˈlakut')}
 {('animal', 'lama1277-botun', 'əwã'), ('animal', 'lama1277-lamah', 'əwã'), ('animal', 'lama1277-waiba', 'əwã'), ('animal', 'keda1252', 'ewaŋ'), ('animal', 'keda1252-leuba', 'ʔewaŋ'), ('animal', 'lama1277-kalik', 'əˈva'), ('animal', 'lama1277-belan', 'əwan nawu'), ('animal', 'lama1277-ritae', 'əwã'), ('animal', 'lama1277-tanju', 'əwã'), ('animal', 'lama1277-lewot', 'əfanŋ'), ('animal', 'lama1277-merde', 'əwã (nawu)'), ('animal', 'lama1277-minga', 'əfau'), ('animal', 'lama1277-horow', 'əwã'), ('animal', 'lama1277-dulhi', 'əwã'), ('animal', 'lama1277-kalik', 'əˈvaŋ'), ('animal', 'lama1277-lamak', 'əwã'), ('animal', 'lama1277-ileap', 'əwaŋnawun'), ('animal', 'lama1277-adona', 'ˈʔɜˈwã'), ('animal', 'lama1277-lewoe', 'əwã(nawu)'), ('animal', 'lama1277-mulan', 'əwə'), ('animal', 'lama1277-kiwan', 'əwã'), ('animal', 'lama1277-lamat', 'əwa ̃(nawu)'), ('animal', 'lama1277-lewuk', 'əwaŋ'), ('animal', 'lama1277-watan', 'əwã'), ('animal', 'lama1277-waiwa', 'əwã'), ('animal', 'lama1277-wuake', 'əwã'), ('animal', 'lama1277-imulo', 'əfaŋ'), ('animal', 'lama1277-lewop', 'əfaŋ'), ('animal', 'lama1277-bama', 'əwã'), ('animal', 'lama1277-kalik', 'əwaŋ'), ('animal', 'lama1277-lamal', 'əfã')}

  Just for clarity:
  There are also the following similarity classes shared between Alorese and LH.
  {('animal', 'alor1247-munas', "bi'nata"), ('animal', 'pura1258', "bi'nanta"), ('animal', 'lama1277-lewoi', 'biˈnataŋ'), ('animal', 'lama1277-lewok', 'binataŋ'), ('animal', 'adan1251-otvai', '’binanta'), ('animal', 'alor1247-besar', "bi'nataŋ"), ('animal', 'rett1240', 'binanta'), ('animal', 'alor1247-pandai', "bi'nata"), ('animal', 'sika1262-hewa', 'binataŋ'), ('animal', 'lama1277-baipi', 'binataŋ'), ('animal', 'kira1248', 'binatan'), ('animal', 'lama1277-lewob', 'binataŋ'), ('animal', 'lama1277-pukau', 'binataŋ'), ('animal', 'kabo1247', "bi'naŋta"), ('animal', 'teiw1235', 'binata'), ('animal', 'wers1238-taram', 'binaʔat'), ('animal', 'wers1238-marit', "bi'nant"), ('animal', 'lama1277-lewog', 'binataŋ'), ('animal', 'sawi1256', "bi'nanta")}


Concept ‘mud’ is different between Alorese and Lamaholot.

 Alorese innovated the following forms:
 {('mud', 'adan1251-lawah', 'parah'), ('mud', 'kabo1247', 'para'), ('mud', 'adan1251-otvai', '’par'), ('mud', 'kama1365', 'paroŋ'), ('mud', 'lamm1241-westp', 'para'), ('mud', 'alor1247-besar', 'para')}
 {('mud', 'alor1247-munas', 'mɔta')}
 Alorese shares the following forms with TAP languages:
 {('mud', 'adan1251-lawah', 'parah'), ('mud', 'kabo1247', 'para'), ('mud', 'adan1251-otvai', '’par'), ('mud', 'kama1365', 'paroŋ'), ('mud', 'lamm1241-westp', 'para'), ('mud', 'alor1247-besar', 'para')}
 {('mud', 'alor1247-munas', 'mɔta')}
 Alorese shares the following forms with other AN languanges, but not Lamaholot:
 {('mud', 'abui1241-takal', "pä.'däq"), ('mud', 'baka1276', 'buta'), ('mud', 'alor1247-pandai', 'buta'), ('mud', 'abui1241-petle', "na.'bak"), ('mud', 'p-mala1545-acd', '*pitek'), ('mud', 'p-mala1545-acd', '*pitak'), ('mud', 'blag1240-bama', 'buta')}
 One or more Lamaholot dialects innovated forms not found in Alorese:
 {('mud', 'lama1277-kalik', 'keˈmat')}
 {('mud', 'lama1277-lerek', 'ˈwalaŋ'), ('mud', 'kaer1234', "li'saq"), ('mud', 'lama1277-adona', 'ˈwalã'), ('mud', 'blag1240-tuntu', "wa'lasa"), ('mud', 'blag1240-warsa', 'alasax'), ('mud', 'lama1277-lewoi', 'ˈwalɑŋ')}


Concept ‘to_let_go’ is different between Alorese and Lamaholot.

 Alorese innovated the following forms:
 {('to_let_go', 'alor1247-besar', "la'pasa")}
 {('to_let_go', 'alor1247-pandai', 'hoe')}
 {('to_let_go', 'alor1247-munas', 'nawuŋ')}
 One or more Lamaholot dialects innovated forms not found in Alorese:
 {('to_let_go', 'lama1277-adona', 'ˈloʔok'), ('to_let_go', 'lama1277-lewoi', 'ˈlio'), ('to_let_go', 'lama1277-lerek', 'ˈlokaŋ'), ('to_let_go', 'lama1277-kalik', 'ˈlɔit'), ('to_let_go', 'lama1277-lewoi', 'ˈloʔo'), ('to_let_go', 'lama1277-kalik', 'ˈlɔka')}


Concept ‘to_grasp’ is different between Alorese and Lamaholot.

 Alorese innovated the following forms:
 {('to_grasp', 'alor1247-munas', "mə'kuŋ"), ('to_grasp', 'alor1247-munas', "kə'kuŋ"), ('to_grasp', 'alor1247-munas', "nə'kuŋ"), ('to_grasp', 'alor1247-munas', "rə'kuŋ"), ('to_grasp', 'alor1247-munas', " tə'kuŋ"), ('to_grasp', 'alor1247-pandai', "p'ragɛŋ")}
 One or more Lamaholot dialects innovated forms not found in Alorese:
 {('to_grasp', 'lama1277-kalik', 'soˈɡa')}

  Just for clarity:
  There are also the following similarity classes shared between Alorese and LH.
  {('to_grasp', 'lama1277-lewoi', 'ˈpɛhɛŋ'), ('to_grasp', 'alor1247-pandai', 'paha'), ('to_grasp', 'lama1277-adona', 'ˈpehẽ'), ('to_grasp', 'alor1247-besar', 'paha'), ('to_grasp', 'lama1277-lerek', 'pəheˈnaŋ')}


Concept ‘green’ is different between Alorese and Lamaholot.

 Alorese shares the following forms with TAP languages:
 {('green', 'adan1251-otvai', '’leu'), ('green', 'adan1251-lawah', 'leu'), ('green', 'alor1247-baran', 'lɔ̃:')}
 One or more Lamaholot dialects innovated forms not found in Alorese:
 {('green', 'lama1277-lewob', 'ijo'), ('green', 'lama1277-wuake', 'hijo'), ('green', 'lama1277-baipi', 'ijon'), ('green', 'lama1277-tanju', 'ijo'), ('green', 'lama1277-lewoi', 'ˈiʤo'), ('green', 'lama1277-lamak', 'ijõ'), ('green', 'lama1277-lewom', 'ijõ'), ('green', 'lama1277-waiba', 'ijo'), ('green', 'lama1277-watan', 'ijõ'), ('green', 'lama1277-botun', 'ijõ')}
 {('green', 'atim1239', 'walaŋai'), ('green', 'kelo1247-hopte', "wə'lɛŋ"), ('green', 'abui1241-takal', "wä.lä.'ŋäi"), ('green', 'abui1241-fuime', 'walaŋai'), ('green', 'abui1241-petle', "wa.la.'ŋai"), ('green', 'kula1280-lanto', '’walaŋ’ka'), ('green', 'sawi1256', "wa'laŋara"), ('green', 'wers1238-taram', 'walar'), ('green', 'kula1280-lanto', 'walaŋka'), ('green', 'kama1365', 'waːi'), ('green', 'lama1277-pukau', 'halaŋ'), ('green', 'abui1241-petle', "wa.la.'ŋɪi"), ('green', 'kelo1247-bring', 'wəwɛlɛŋ')}
 {('green', 'abui1241-ulaga', 'buloŋai'), ('green', 'kira1248', 'balowa'), ('green', 'kafo1240', 'biluɑi'), ('green', 'abui1241-ulaga', 'biloŋai'), ('green', 'lama1277-minga', 'pələm')}
 {('green', 'lama1277-lewot', 'keorən'), ('green', 'lama1277-imulo', 'keorən'), ('green', 'lama1277-horow', 'keorã'), ('green', 'lama1277-lamal', 'keor'), ('green', 'lama1277-adona', 'ˈkeorɛ'), ('green', 'lama1277-mulan', 'keor')}
 {('green', 'lama1277-lewuk', 'kəbəris'), ('green', 'lama1277-lewok', 'pəris'), ('green', 'lama1277-lamah', 'kəpəri')}

  Just for clarity:
  There are also the following similarity classes shared between Alorese and LH.
  {('green', 'keda1252-leuba', "ta'ŋɛn"), ('green', 'keda1252', 'taŋen'), ('green', 'lama1277-merde', 'pətaŋənə'), ('green', 'lama1277-kiwan', 'bət’ã'), ('green', 'lama1277-kalik', 'pətaŋəs'), ('green', 'lama1277-paina', 'pataŋənən'), ('green', 'lama1277-ritae', 'bətɤ'), ('green', 'lama1277-lamat', 'taŋenə'), ('green', 'lama1277-lerek', 'pətaˈŋan'), ('green', 'lama1277-belan', 'pəntaŋəs'), ('green', 'lama1277-lewop', 'pətaŋən'), ('green', 'lama1277-bama', 'bətʔã'), ('green', 'lama1277-dulhi', 'bətʔã'), ('green', 'lama1277-lewoe', 'taŋe'), ('green', 'alor1247-munas', 'taŋaŋ'), ('green', 'keda1252-leuwa', 'taŋε'), ('green', 'keda1252-leuwa', 'taŋεn dɔlɔr'), ('green', 'lama1277-lewog', 'bətʔã'), ('green', 'lama1277-waiwa', 'bətʔã'), ('green', 'hama1240', 'baʔ.oir')}
  {('green', 'sika1262-hewa', 'daʔan'), ('green', 'lama1277-kalik', 'iˈʤoni'), ('green', 'alor1247-besar', 'iʤɔŋ'), ('green', 'alor1247-pandai', 'idʒɔŋ'), ('green', 'sika1262-tanai', 'daʔa-ŋ'), ('green', 'sika1262-maume', 'daʔan'), ('green', 'lama1277-ileap', 'məritən'), ('green', 'rett1240', 'iʤoŋ')}


Concept ‘brother_in_law’ is different between Alorese and Lamaholot.

 Alorese innovated the following forms:
 {('brother_in_law', 'alor1247-munas', 'naraŋ'), ('brother_in_law', 'alor1247-pandai', 'naraŋ')}
 {('brother_in_law', 'alor1247-munas', 'opuʔ'), ('brother_in_law', 'alor1247-pandai', 'ɔpuŋ'), ('brother_in_law', 'alor1247-besar', 'opuŋ'), ('brother_in_law', 'alor1247-munas', 'opuŋ')}
 One or more Lamaholot dialects innovated forms not found in Alorese:
 {('brother_in_law', 'lama1277-lewoi', 'aʔe'), ('brother_in_law', 'keda1252-leuba', 'aʔe')}
 {('brother_in_law', 'lama1277-lewoi', 'ariʔ'), ('brother_in_law', 'keda1252-leuba', 'ariʔ'), ('brother_in_law', 'sika1262-hewa', 'ʋari')}
 {('brother_in_law', 'lama1277-adona', 'bine ʔanaʔ')}


Concept ‘forehead’ is different between Alorese and Lamaholot.

 Alorese innovated the following forms:
 {('forehead', 'alor1247-pandai', "kə'larak"), ('forehead', 'alor1247-munas', "k'larak"), ('forehead', 'alor1247-besar', "kala'rakaŋ")}
 One or more Lamaholot dialects innovated forms not found in Alorese:
 {('forehead', 'lama1277-kalik', 'kəniˈtɔ'), ('forehead', 'lama1277-lewoi', 'kəniˈtɔʔ'), ('forehead', 'lama1277-lerek', 'kəniˈto'), ('forehead', 'lama1277-adona', 'kɜˈniːtoˈkɜt')}


Concept ‘holy’ is different between Alorese and Lamaholot.

 Alorese innovated the following forms:
 {('holy', 'alor1247-munas', "halaŋ 'lahɛ"), ('holy', 'alor1247-besar', "la'hikiŋ"), ('holy', 'alor1247-pandai', 'laheŋ')}
 {('holy', 'alor1247-munas', "mɔlɔŋ 'mɛhɛŋ")}
 One or more Lamaholot dialects innovated forms not found in Alorese:
 {('holy', 'lama1277-lerek', 'ˈorə ˈmulu-mulu')}
 {('holy', 'lama1277-adona', 'kəˈlɔhɔ̃')}
 {('holy', 'sika1262-hewa', 'suʧi'), ('holy', 'keda1252-leuba', 'sutʃi'), ('holy', 'lama1277-lewoi', 'ˈsutʃi')}


Concept ‘lontar_palm’ is different between Alorese and Lamaholot.

 Alorese innovated the following forms:
 {('lontar_palm', 'alor1247-besar', "ka'dafu")}
 One or more Lamaholot dialects innovated forms not found in Alorese:
 {('lontar_palm', 'lama1277-lewoi', 'ˈmara')}
 {('lontar_palm', 'kira1248', 'tuk'), ('lontar_palm', 'blag1240-bama', 'tox'), ('lontar_palm', 'lama1277-adona', 'ˈtuak'), ('lontar_palm', 'blag1240-warsa', 'tox'), ('lontar_palm', 'blag1240-kulij', 'tua'), ('lontar_palm', 'dein1238', 'tuah'), ('lontar_palm', 'adan1251-otvai', '’tɔk'), ('lontar_palm', 'koto1251', 'tuaʔ'), ('lontar_palm', 'sarr1247-adiab', 'tuax')}

  Just for clarity:
  There are also the following similarity classes shared between Alorese and LH.
  {('lontar_palm', 'lama1277-kalik', 'ˈkolɛr'), ('lontar_palm', 'kaer1234', 'ai qoli'), ('lontar_palm', 'pura1258', 'tua kori'), ('lontar_palm', 'rett1240', 'kɔli ɛʤɛl'), ('lontar_palm', 'baka1276', 'tua: ai oli'), ('lontar_palm', 'sika1262-hewa', 'koli'), ('lontar_palm', 'alor1247-munas', "koɔli 'pukɔŋ"), ('lontar_palm', 'kuii1253', 'tu:k yera'), ('lontar_palm', 'blag1240-nule', 'koli'), ('lontar_palm', 'lama1277-lerek', 'koˈler'), ('lontar_palm', 'lama1277-kalik', 'ˈkoli'), ('lontar_palm', 'rett1240', 'enkuali'), ('lontar_palm', 'blag1240-bama', 'tox aikoli'), ('lontar_palm', 'blag1240-tuntu', 'ajkoli'), ('lontar_palm', 'alor1247-pandai', 'kɔli')}


Concept ‘to_divide’ is different between Alorese and Lamaholot.

 Alorese shares the following forms with other AN languanges, but not Lamaholot:
 {('to_divide', 'wers1238-marit', "bə'kar"), ('to_divide', 'baka1276', 'pol'), ('to_divide', 'blag1240-kulij', 'pol'), ('to_divide', 'alor1247-pandai', 'bagɛ'), ('to_divide', 'wers1238-taram', "ba'kar"), ('to_divide', 'kaer1234', "pa'gui"), ('to_divide', 'sawi1256', "ba'kara"), ('to_divide', 'sika1262-hewa', 'bige'), ('to_divide', 'teiw1235', "pa'xai"), ('to_divide', 'rett1240', 'paul'), ('to_divide', 'sarr1247-adiab', 'pahal'), ('to_divide', 'alor1247-munas', 'bagɛ'), ('to_divide', 'blag1240-warsa', 'pagul'), ('to_divide', 'alor1247-besar', 'bagɛ'), ('to_divide', 'sarr1247-nule', 'pahaj'), ('to_divide', 'dein1238', 'paxei'), ('to_divide', 'blag1240-kulij', 'bage'), ('to_divide', 'blag1240-tuntu', 'pagul'), ('to_divide', 'pura1258', 'paul'), ('to_divide', 'blag1240-bama', 'pagul'), ('to_divide', 'p-mala1545-acd', '*baqagi'), ('to_divide', 'keda1252-leuba', 'bɔʔ')}

  Just for clarity:
  There are also the following similarity classes shared between Alorese and LH.
  {('to_divide', 'alor1247-munas', 'wɛkaŋ'), ('to_divide', 'lama1277-lerek', 'veˈkan'), ('to_divide', 'lama1277-adona', 'ˈweka'), ('to_divide', 'lama1277-kalik', 'vɛˈka'), ('to_divide', 'lama1277-lewoi', 'ˈwekɑŋ')}


Concept ‘full’ is different between Alorese and Lamaholot.

 One or more Lamaholot dialects innovated forms not found in Alorese:
 {('full', 'lama1277-kalik', 'məˈnuk'), ('full', 'lama1277-lewoi', 'ˈmənuŋ'), ('full', 'lama1277-lerek', 'mənuˈkən')}

  Just for clarity:
  There are also the following similarity classes shared between Alorese and LH.
  {('full', 'alor1247-pandai', "pa'nːoŋ"), ('full', 'sika1262-hewa', 'bənu'), ('full', 'laka1255', 'benu'), ('full', 'alor1247-munas', "pə'nɔŋ"), ('full', 'alor1247-baran', 'pan:ɔŋ'), ('full', 'keda1252-leuwa', 'pænu mænu'), ('full', 'sika1262-tanai', 'bənu'), ('full', 'maka1316', 'benu'), ('full', 'p-mala1545-acd', '*penuq'), ('full', 'keda1252-leuba', 'pɛnu'), ('full', 'alor1247-besar', 'panːo'), ('full', 'kema1243', 'be:nu'), ('full', 'lama1277-adona', 'ˈpɜnɔ'), ('full', 'tuku1254', 'bɛnu'), ('full', 'mamb1306', 'be:n')}


Concept ‘to_count’ is different between Alorese and Lamaholot.

 Alorese shares the following forms with other AN languanges, but not Lamaholot:
 {('to_count', 'pura1258', 'rɛkɛŋ'), ('to_count', 'alor1247-pandai', 'rɛkɛŋ'), ('to_count', 'blag1240-kulij', 'rekeŋ'), ('to_count', 'alor1247-munas', 'rɛkɛŋ'), ('to_count', 'baka1276', 'rekeŋ'), ('to_count', 'alor1247-baran', 'rɛkiŋ'), ('to_count', 'alor1247-besar', 'rɛkɛŋ'), ('to_count', 'blag1240-nule', 'rekeŋ'), ('to_count', 'sika1262-tanai', 'reke-ŋ')}
 One or more Lamaholot dialects innovated forms not found in Alorese:
 {('to_count', 'sika1262-hewa', 'ɡasik'), ('to_count', 'lama1277-adona', 'ˈɡasik'), ('to_count', 'lama1277-lewoi', 'ˈɡasi'), ('to_count', 'dein1238', 'qiq'), ('to_count', 'lama1277-lerek', 'ˈɡahip'), ('to_count', 'lama1277-kalik', 'ɡaˈsip'), ('to_count', 'sika1262-tanai', 'ɡasik'), ('to_count', 'sika1262-tanai', 'api-ŋ ɡasik')}


Concept ‘to_invite’ is different between Alorese and Lamaholot.

 Alorese innovated the following forms:
 {('to_invite', 'alor1247-besar', "pa'mada")}

  Just for clarity:
  There are also the following similarity classes shared between Alorese and LH.
  {('to_invite', 'lama1277-kalik', 'ˈmali'), ('to_invite', 'lama1277-lewoi', 'ˈmariŋ'), ('to_invite', 'lama1277-adona', 'ˈmarĩ'), ('to_invite', 'adan1251-otvai', '’mariŋ'), ('to_invite', 'alor1247-pandai', "mariŋ 'ata")}
  {('to_invite', 'alor1247-munas', 'ɔndaŋ'), ('to_invite', 'lama1277-lerek', 'ˈundaŋ'), ('to_invite', 'lama1277-kalik', 'sɔˈʤan'), ('to_invite', 'alor1247-pandai', 'ɔnda'), ('to_invite', 'keda1252-leuba', 'ʔundaŋ')}


Concept ‘mosquito’ is different between Alorese and Lamaholot.

 One or more Lamaholot dialects innovated forms not found in Alorese:
 {('mosquito', 'lama1277-adona', 'kɜˈraɡã')}
 {('mosquito', 'lama1277-kalik', 'kəməˈŋɛŋ'), ('mosquito', 'lama1277-kalik', 'kəməˈŋɛ'), ('mosquito', 'lama1277-lerek', 'kəməˈŋeŋ')}

  Just for clarity:
  There are also the following similarity classes shared between Alorese and LH.
  {('mosquito', 'lama1277-lewoi', 'kəˈnəpuŋ'), ('mosquito', 'alor1247-pandai', 'kapːuŋ'), ('mosquito', 'wers1238-marit', 'kubuŋ'), ('mosquito', 'wers1238-taram', 'kubuŋ'), ('mosquito', 'alor1247-besar', 'kapuŋ'), ('mosquito', 'alor1247-munas', 'kapːuŋ')}


Concept ‘arrow’ is different between Alorese and Lamaholot.

 One or more Lamaholot dialects innovated forms not found in Alorese:
 {('arrow', 'lama1277-lerek', 'ˈəmat')}
 {('arrow', 'keda1252-leuba', "na'mɛʔ"), ('arrow', 'lama1277-lewoi', 'ˈamɜ'), ('arrow', 'lama1277-adona', 'ʔamɜt')}

  Just for clarity:
  There are also the following similarity classes shared between Alorese and LH.
  {('arrow', 'alor1247-besar', 'hupɔ'), ('arrow', 'lama1277-kalik', 'ˈsupəl'), ('arrow', 'lama1277-lerek', 'ˈhupəl'), ('arrow', 'alor1247-munas', 'hupɔl'), ('arrow', 'lamm1241-westp', 'upal'), ('arrow', 'alor1247-pandai', 'hupɔl'), ('arrow', 'sika1262-hewa', 'huper')}


Concept ‘murder’ is different between Alorese and Lamaholot.

 Alorese shares the following forms with other AN languanges, but not Lamaholot:
 {('murder', 'alor1247-pandai', "belo 'buno"), ('murder', 'alor1247-besar', 'bunɔ'), ('murder', 'keda1252-leuba', "bɛ'lɔʔ")}
 One or more Lamaholot dialects innovated forms not found in Alorese:
 {('murder', 'lama1277-kalik', 'ˈbelu baˈkat')}

  Just for clarity:
  There are also the following similarity classes shared between Alorese and LH.
  {('murder', 'alor1247-munas', "bunɔ wə'ki"), ('murder', 'lama1277-adona', 'belo ˈwɜkika'), ('murder', 'lama1277-lewoi', 'ˈbelo ˈwəkika'), ('murder', 'lama1277-lerek', 'bel ˈvaki'), ('murder', 'alor1247-pandai', "belo wə'kiŋ")}


Concept ‘cassava’ is different between Alorese and Lamaholot.

 Alorese innovated the following forms:
 {('cassava', 'alor1247-munas', "kura 'ʤawa"), ('cassava', 'alor1247-pandai', 'kura'), ('cassava', 'alor1247-baran', 'kur:a'), ('cassava', 'alor1247-besar', "kura 'ʤafa")}
 One or more Lamaholot dialects innovated forms not found in Alorese:
 {('cassava', 'lama1277-kalik', 'skaˈʤo uˈsər')}
 {('cassava', 'lama1277-lewoi', 'uwe kajɔ'), ('cassava', 'lama1277-adona', 'uwe ˈkaju'), ('cassava', 'lama1277-lerek', 'ˈhure ˈkajor')}


Concept ‘to_help’ is different between Alorese and Lamaholot.

 Alorese innovated the following forms:
 {('to_help', 'alor1247-munas', 'sambɔ'), ('to_help', 'alor1247-pandai', 'sambɔ'), ('to_help', 'alor1247-besar', 'sambɔ')}
 One or more Lamaholot dialects innovated forms not found in Alorese:
 {('to_help', 'kama1365', 'tolon'), ('to_help', 'tetu1245-suai', 'tulun'), ('to_help', 'atim1239', 'netuluŋ'), ('to_help', 'p-mala1545-acd', '*tuluŋ'), ('to_help', 'maka1316', "tu'lunu"), ('to_help', 'sawi1256', "tulu'ana"), ('to_help', 'wers1238-marit', 'tuluŋ'), ('to_help', 'fata1247', 'tɔlunɛ'), ('to_help', 'tetu1245-vique', 'tulun'), ('to_help', 'lama1277-lewoi', 'ˈtuluŋ')}
 {('to_help', 'lama1277-kalik', 'ɡələˈkat')}
 {('to_help', 'lama1277-kalik', 'ˈpɔi'), ('to_help', 'lama1277-lerek', 'ˈpoɛŋ'), ('to_help', 'keda1252-leuba', "po'hiŋ")}
 {('to_help', 'lama1277-adona', 'ɡɜˈmohe')}
 {('to_help', 'lama1277-kalik', 'sɛrˈvi')}


Concept ‘to_trade’ is different between Alorese and Lamaholot.

 Alorese innovated the following forms:
 {('to_trade', 'alor1247-munas', "raŋ 'wu")}
 {('to_trade', 'alor1247-pandai', "p'nahaŋ 'apa")}
 One or more Lamaholot dialects innovated forms not found in Alorese:
 {('to_trade', 'lama1277-lewoi', 'ˈduʔuŋ ˈhope'), ('to_trade', 'lama1277-adona', 'ˈduʔũ ˈhope')}
 {('to_trade', 'adan1251-otvai', '’dagaŋ'), ('to_trade', 'wers1238-marit', 'dagaŋ'), ('to_trade', 'teiw1235', "da'gan"), ('to_trade', 'sawi1256', "dakaŋ ta're"), ('to_trade', 'keda1252-leuba', 'duruŋ'), ('to_trade', 'lama1277-lerek', 'peˈdurum')}

  Just for clarity:
  There are also the following similarity classes shared between Alorese and LH.
  {('to_trade', 'sika1262-hewa', 'pəlele'), ('to_trade', 'lama1277-lerek', 'pəˈlele'), ('to_trade', 'alor1247-besar', "papa 'lɛlɛ")}


Concept ‘light_weight’ is different between Alorese and Lamaholot.

 Alorese innovated the following forms:
 {('light_weight', 'alor1247-besar', 'bɛru'), ('light_weight', 'alor1247-pandai', 'bɛrɛ'), ('light_weight', 'alor1247-munas', 'bɛrɛ')}
 One or more Lamaholot dialects innovated forms not found in Alorese:
 {('light_weight', 'sika1262-hewa', 'heak'), ('light_weight', 'lama1277-lerek', 'kəleaˈŋen'), ('light_weight', 'lama1277-lewoi', 'kəˈlea'), ('light_weight', 'lama1277-kalik', 'kleˈak')}
 {('light_weight', 'lama1277-adona', 'kɜˈliɛʔ')}


Concept ‘to_search_for_to_hunt_for’ is different between Alorese and Lamaholot.

 Alorese innovated the following forms:
 {('to_search_for_to_hunt_for', 'alor1247-munas', 'gɛna'), ('to_search_for_to_hunt_for', 'alor1247-pandai', 'gɛna'), ('to_search_for_to_hunt_for', 'alor1247-besar', 'gɛna'), ('to_search_for_to_hunt_for', 'alor1247-baran', 'gɛna')}
 {('to_search_for_to_hunt_for', 'alor1247-munas', 'gɛna'), ('to_search_for_to_hunt_for', 'alor1247-besar', 'gɛna')}
 One or more Lamaholot dialects innovated forms not found in Alorese:
 {('to_search_for_to_hunt_for', 'lama1277-lewoi', 'ˈsebaʔ'), ('to_search_for_to_hunt_for', 'lama1277-adona', 'ˈsɜbɑʔ')}
 {('to_search_for_to_hunt_for', 'lama1277-lerek', 'doˈriŋ')}
 {('to_search_for_to_hunt_for', 'lama1277-kalik', 'ɡəˈsak')}


Concept ‘mucus’ is different between Alorese and Lamaholot.

 Alorese innovated the following forms:
 {('mucus', 'alor1247-munas', 'sini'), ('mucus', 'alor1247-pandai', 'sini')}
 Alorese shares the following forms with other AN languanges, but not Lamaholot:
 {('mucus', 'keda1252-leuba', "niŋ 'tein"), ('mucus', 'alor1247-besar', "niruŋ 'teiŋ")}
 One or more Lamaholot dialects innovated forms not found in Alorese:
 {('mucus', 'lama1277-adona', 'ˈehuˈkɜt'), ('mucus', 'lama1277-lewoi', 'ˈneʔu'), ('mucus', 'p-mala1545-acd', '*ŋuhuR')}
 {('mucus', 'lama1277-lerek', 'nidʒ͡u koˈtol'), ('mucus', 'lama1277-kalik', 'niˈʤu kɔˈtɔl')}


Concept ‘mountain’ is different between Alorese and Lamaholot.

 Alorese shares the following forms with TAP languages:
 {('mountain', 'baka1276', 'pondo'), ('mountain', 'blag1240-kulij', 'pondo'), ('mountain', 'alor1247-munas', "wɔtɔ b'lɔlɔk"), ('mountain', 'alor1247-pandai', "wɔtɔ 'blɔlɔ"), ('mountain', 'alor1247-besar', "dɔla balɔ'lɔkɔŋ"), ('mountain', 'alor1247-baran', 'foto'), ('mountain', 'blag1240-nule', 'pondo')}
 One or more Lamaholot dialects innovated forms not found in Alorese:
 {('mountain', 'lama1277-kiwan', 'ile'), ('mountain', 'lama1277-watan', 'ile'), ('mountain', 'lama1277-lamal', 'ile'), ('mountain', 'lama1277-lewob', 'ile'), ('mountain', 'lama1277-lewom', 'ile'), ('mountain', 'lama1277-ritae', 'ile'), ('mountain', 'lama1277-waiba', 'ile'), ('mountain', 'lama1277-wuake', 'ile'), ('mountain', 'fata1247', 'ili'), ('mountain', 'lama1277-waiwa', 'ile'), ('mountain', 'lama1277-horow', 'ile'), ('mountain', 'lama1277-lamah', 'ile'), ('mountain', 'lama1277-lerek', 'ˈiler'), ('mountain', 'lama1277-baipi', 'ile'), ('mountain', 'lama1277-paina', 'iler'), ('mountain', 'lama1277-bama', 'ile'), ('mountain', 'lama1277-kalik', 'iˈler'), ('mountain', 'sika1262-hewa', 'ilin'), ('mountain', 'lama1277-minga', 'ileru'), ('mountain', 'keda1252', 'ili'), ('mountain', 'lama1277-lamak', 'ile'), ('mountain', 'lama1277-lewog', 'ile'), ('mountain', 'lama1277-lewot', 'ileru'), ('mountain', 'lama1277-kalik', 'ile'), ('mountain', 'lama1277-dulhi', 'ile'), ('mountain', 'lama1277-pukau', 'ile'), ('mountain', 'lama1277-imulo', 'iler'), ('mountain', 'lama1277-lewoe', 'ile'), ('mountain', 'sika1262-hewa', 'i̤lin'), ('mountain', 'lama1277-lewok', 'ileru'), ('mountain', 'lama1277-lewop', 'ile'), ('mountain', 'lama1277-merde', 'ile'), ('mountain', 'lama1277-kalik', 'iˈli'), ('mountain', 'lama1277-ileap', 'ile'), ('mountain', 'lama1277-botun', 'ile'), ('mountain', 'lama1277-lamat', 'ile'), ('mountain', 'lama1277-adona', 'ʔile'), ('mountain', 'lama1277-lewuk', 'iler'), ('mountain', 'keda1252-leuba', '(j)ili'), ('mountain', 'lama1277-belan', 'ile'), ('mountain', 'lama1277-lewoi', 'ile'), ('mountain', 'keda1252-leuwa', 'i̘li'), ('mountain', 'maka1316', 'ili'), ('mountain', 'p-cent2245-abvd', '*halas'), ('mountain', 'lama1277-mulan', 'ile'), ('mountain', 'sika1262-tanai', 'ili-n'), ('mountain', 'p-mala1545-acd', '*qilih'), ('mountain', 'lama1277-tanju', 'ile')}


Concept ‘to_freeze’ is different between Alorese and Lamaholot.

 Alorese innovated the following forms:
 {('to_freeze', 'alor1247-pandai', "ma'tːɛŋ")}
 One or more Lamaholot dialects innovated forms not found in Alorese:
 {('to_freeze', 'lama1277-lerek', 'nətərˈha')}
 {('to_freeze', 'lama1277-adona', 'ˈmarana')}

  Just for clarity:
  There are also the following similarity classes shared between Alorese and LH.
  {('to_freeze', 'tetu1245-vique', "sai'fatuk"), ('to_freeze', 'tetu1246', "sɑi fɑ'tuk"), ('to_freeze', 'alor1247-baran', 'fatɔ'), ('to_freeze', 'alor1247-besar', 'fatɔnaŋ'), ('to_freeze', 'tetu1245-suai', "sai'fatuk"), ('to_freeze', 'lama1277-kalik', 'vaˈtʊk')}


Concept ‘cheap’ is different between Alorese and Lamaholot.

 Alorese innovated the following forms:
 {('cheap', 'alor1247-pandai', "wɛliŋ 'lahɛ")}
 Alorese shares the following forms with other AN languanges, but not Lamaholot:
 {('cheap', 'keda1252-leuba', 'mura'), ('cheap', 'alor1247-besar', 'mura'), ('cheap', 'sika1262-hewa', 'murah')}
 One or more Lamaholot dialects innovated forms not found in Alorese:
 {('cheap', 'lama1277-adona', 'welĩ ˈtake')}
 {('cheap', 'lama1277-lerek', 'ˈweli ˈlere-ˈlere')}

  Just for clarity:
  There are also the following similarity classes shared between Alorese and LH.
  {('cheap', 'lama1277-lewoi', 'ˈweliŋ ˈbəruwa'), ('cheap', 'alor1247-munas', "weliŋ 'wɛrɔʔ")}


Concept ‘inside’ is different between Alorese and Lamaholot.

 One or more Lamaholot dialects innovated forms not found in Alorese:
 {('inside', 'lama1277-watan', 'onʔã'), ('inside', 'lama1277-lamah', 'onã'), ('inside', 'lama1277-lewog', 'onə'), ('inside', 'lama1277-bama', 'onə'), ('inside', 'lama1277-tanju', 'onə'), ('inside', 'lama1277-dulhi', 'onʔã'), ('inside', 'lama1277-lewob', 'onã'), ('inside', 'lama1277-kiwan', 'dəonã'), ('inside', 'lama1277-ritae', 'onã'), ('inside', 'lama1277-horow', 'onʔã'), ('inside', 'lama1277-belan', 'on'), ('inside', 'lama1277-mulan', 'onɤ'), ('inside', 'lama1277-waiwa', 'onã')}
 {('inside', 'lama1277-adona', 'ˈʔɔnʔeʔ')}
 {('inside', 'lama1277-lewok', 'or')}
 {('inside', 'lama1277-baipi', 'bəlomə'), ('inside', 'keda1252', 'lomo'), ('inside', 'lama1277-ileap', 'lomək'), ('inside', 'lama1277-lewom', 'bəlomo'), ('inside', 'sika1262-hewa', 'ləman'), ('inside', 'lama1277-waiba', 'bəlomə'), ('inside', 'lama1277-botun', 'bəlomã'), ('inside', 'lama1277-lewoe', 'ləmə'), ('inside', 'lama1277-pukau', 'ləmã'), ('inside', 'lama1277-wuake', 'bəlome'), ('inside', 'lama1277-merde', 'bəlomã'), ('inside', 'lama1277-lamal', 'belomã'), ('inside', 'lama1277-lamak', 'bəlomã')}
 {('inside', 'lama1277-lamat', 'huʔa')}
 {('inside', 'lama1277-lewop', 'gom'), ('inside', 'lama1277-kalik', 'gomək'), ('inside', 'lama1277-lewuk', 'gom'), ('inside', 'lama1277-lewot', 'goməkən'), ('inside', 'lama1277-imulo', 'goməkən'), ('inside', 'lama1277-minga', 'gomək'), ('inside', 'lama1277-paina', 'gomakən')}
 {('inside', 'lama1277-lerek', 'dʒ͡e ... ˈora'), ('inside', 'lama1277-kalik', 'ʤe ... or')}

  Just for clarity:
  There are also the following similarity classes shared between Alorese and LH.
  {('inside', 'alor1247-munas', 'ɔnɔŋ'), ('inside', 'sika1262-hewa', 'unen'), ('inside', 'lama1277-lewoi', 'ˈɔnɔ'), ('inside', 'alor1247-besar', 'ɔnɔŋ'), ('inside', 'sika1262-maume', 'une(ŋ)'), ('inside', 'koto1251', 'nana-n'), ('inside', 'alor1247-pandai', 'ɔnɔŋ')}


Concept ‘vein’ is different between Alorese and Lamaholot.

 Alorese innovated the following forms:
 {('vein', 'alor1247-besar', "u'rakaŋ")}
 {('vein', 'alor1247-besar', "u'rakaŋ")}
 One or more Lamaholot dialects innovated forms not found in Alorese:
 {('vein', 'lama1277-kalik', 'aˈlis'), ('vein', 'sika1262-hewa', 'a:li')}

  Just for clarity:
  There are also the following similarity classes shared between Alorese and LH.
  {('vein', 'lama1277-adona', 'a:lˈhĩ'), ('vein', 'lama1277-lerek', 'alhin'), ('vein', 'kama1365', 'aliː'), ('vein', 'lama1277-lewoi', 'aliˈhiŋ'), ('vein', 'alor1247-munas', 'aliŋ'), ('vein', 'keda1252-leuba', "a'li")}


Concept ‘wood’ is different between Alorese and Lamaholot.

 One or more Lamaholot dialects innovated forms not found in Alorese:
 {('wood', 'lama1277-lerek', 'kaj ˈtavan')}

  Just for clarity:
  There are also the following similarity classes shared between Alorese and LH.
  {('wood', 'lama1277-kalik', 'ˈkaʤɔr'), ('wood', 'p-cent2245-abvd', '*kayu'), ('wood', 'lama1277-kalik', 'ˈkaʤu'), ('wood', 'alor1247-pandai', 'kaʤɔ'), ('wood', 'alor1247-besar', 'kaʤɔ'), ('wood', 'lama1277-adona', 'ˈkajo'), ('wood', 'lama1277-lewoi', 'ˈkajo'), ('wood', 'alor1247-munas', 'kaʤɔ')}


Concept ‘raised_platform’ is different between Alorese and Lamaholot.

 Alorese innovated the following forms:
 {('raised_platform', 'alor1247-pandai', 'sadu')}
 Alorese shares the following forms with other AN languanges, but not Lamaholot:
 {('raised_platform', 'blag1240-warsa', 'deki'), ('raised_platform', 'blag1240-nule', 'dej'), ('raised_platform', 'alor1247-munas', 'dɛki'), ('raised_platform', 'pura1258', 'dei'), ('raised_platform', 'alor1247-pandai', 'dɛki'), ('raised_platform', 'blag1240-tuntu', 'deki'), ('raised_platform', 'alor1247-besar', 'dɛki'), ('raised_platform', 'kuii1253', 'taka'), ('raised_platform', 'teiw1235', 'dɛ:k'), ('raised_platform', 'sika1262-hewa', 'tepi'), ('raised_platform', 'kira1248', 'taka'), ('raised_platform', 'blag1240-bama', 'deki'), ('raised_platform', 'kaer1234', 'dɛ:ki'), ('raised_platform', 'kaer1234', 'dɛ:k'), ('raised_platform', 'adan1251-otvai', '’deʔ'), ('raised_platform', 'blag1240-kulij', 'dei'), ('raised_platform', 'baka1276', 'dei')}
 One or more Lamaholot dialects innovated forms not found in Alorese:
 {('raised_platform', 'lama1277-adona', 'kɜˈnatã')}
 {('raised_platform', 'lama1277-lerek', 'kəˈnepiŋ')}
 {('raised_platform', 'lama1277-kalik', 'uˈlik')}
 {('raised_platform', 'lama1277-lewoi', 'buaŋ')}


Concept ‘good’ is different between Alorese and Lamaholot.

 One or more Lamaholot dialects innovated forms not found in Alorese:
 {('good', 'lama1277-lamat', 'bəliʔa')}
 {('good', 'lama1277-mulan', 'alusɤ'), ('good', 'lama1277-lewuk', 'alus'), ('good', 'lama1277-paina', 'aluhən'), ('good', 'lama1277-kalik', 'alus'), ('good', 'lama1277-lamah', 'alus'), ('good', 'lama1277-lerek', 'aluˈhən'), ('good', 'lama1277-lewok', 'alus'), ('good', 'lama1277-merde', 'alusã'), ('good', 'lama1277-kalik', 'aˈlus')}
 {('good', 'lama1277-lewog', 'əreʔ'), ('good', 'lama1277-lamal', 'sənareŋ'), ('good', 'lama1277-watan', 'əre'), ('good', 'lama1277-lamak', 'ərʔẽ'), ('good', 'lama1277-lewoi', 'əˈrɛŋ'), ('good', 'lama1277-ileap', 'sare'), ('good', 'lama1277-ritae', 'sənare'), ('good', 'lama1277-lewob', 'sarẽ'), ('good', 'lama1277-bama', 'əre'), ('good', 'lama1277-imulo', 'sənarekən')}
 {('good', 'lama1277-pukau', 'masoʔ')}
 {('good', 'lama1277-kiwan', 'məlʔã'), ('good', 'lama1277-lewot', 'məla'), ('good', 'lama1277-dulhi', 'məlaʔ'), ('good', 'lama1277-minga', 'məla'), ('good', 'kema1243', "mo'lɔi"), ('good', 'lama1277-waiwa', 'məlã'), ('good', 'lama1277-horow', 'məlʔã'), ('good', 'lama1277-wuake', 'məlã'), ('good', 'lama1277-lewop', 'məla'), ('good', 'lama1277-adona', 'ˈmɜʔlaʔ'), ('good', 'lama1277-botun', 'məlʔã'), ('good', 'lama1277-belan', 'məla'), ('good', 'tuku1254', "bo'loi")}
 {('good', 'lama1277-waiba', 'mae'), ('good', 'p-cent2245-abvd', '*ma-pia'), ('good', 'p-mala1545-abvd', '*ma-pia'), ('good', 'lama1277-baipi', 'mae'), ('good', 'lama1277-lewom', 'mãe'), ('good', 'lama1277-tanju', 'mae')}

  Just for clarity:
  There are also the following similarity classes shared between Alorese and LH.
  {('good', 'alor1247-besar', 'dikɛ'), ('good', 'tetu1245-vique', 'diak'), ('good', 'alor1247-munas', 'dikɛk'), ('good', 'lama1277-lewoe', 'diʔe'), ('good', 'keda1252', 'diʔen'), ('good', 'alor1247-baran', 'dik:ɛ'), ('good', 'p-mala1545-acd', '*diqaq'), ('good', 'alor1247-pandai', 'dikɛk'), ('good', 'tetu1246', 'diɑk'), ('good', 'keda1252-leuwa', 'diʔεn hεrun'), ('good', 'keda1252-leuba', 'diʔən'), ('good', 'tetu1245-suai', 'diak')}


Concept ‘burden_stick’ is different between Alorese and Lamaholot.

 Alorese innovated the following forms:
 {('burden_stick', 'alor1247-pandai', "apa 'tikiŋ 'wai")}
 {('burden_stick', 'alor1247-pandai', 'rikiŋ')}

  Just for clarity:
  There are also the following similarity classes shared between Alorese and LH.
  {('burden_stick', 'alor1247-munas', "ləp'lɛpaʔ"), ('burden_stick', 'sika1262-hewa', 'leba'), ('burden_stick', 'lama1277-lerek', 'bəˈlebar'), ('burden_stick', 'lama1277-lewoi', 'bəˈlebaʔ'), ('burden_stick', 'tetu1245-vique', 'leba'), ('burden_stick', 'blag1240-warsa', 'kəlepa'), ('burden_stick', 'lama1277-kalik', 'bəleˈbar'), ('burden_stick', 'lama1277-adona', "bə'lebaʔ"), ('burden_stick', 'alor1247-besar', "kə'lapaŋ"), ('burden_stick', 'tetu1245-suai', 'leba'), ('burden_stick', 'lama1277-kalik', 'bəleˈba')}


Concept ‘to_sleep’ is different between Alorese and Lamaholot.

 One or more Lamaholot dialects innovated forms not found in Alorese:
 {('to_sleep', 'lama1277-imulo', 'gəle'), ('to_sleep', 'lama1277-lewop', 'gəle'), ('to_sleep', 'lama1277-lewot', 'gəle'), ('to_sleep', 'lama1277-minga', 'gəle'), ('to_sleep', 'lama1277-kalik', 'gəle'), ('to_sleep', 'lama1277-lewok', 'gəle'), ('to_sleep', 'lama1277-lewuk', 'gəle'), ('to_sleep', 'lama1277-paina', 'galek(a)'), ('to_sleep', 'lama1277-lerek', 'ɡəˈle'), ('to_sleep', 'lama1277-kalik', 'ɡəˈlɛ')}

  Just for clarity:
  There are also the following similarity classes shared between Alorese and LH.
  {('to_sleep', 'lama1277-bama', 'turu'), ('to_sleep', 'lama1277-tanju', 'turu'), ('to_sleep', 'lama1277-kiwan', 'turu'), ('to_sleep', 'lama1277-baipi', 'turu'), ('to_sleep', 'lama1277-lewoe', 'turu'), ('to_sleep', 'alor1247-besar', 'turu'), ('to_sleep', 'lama1277-wuake', 'turu'), ('to_sleep', 'lama1277-waiwa', 'turu'), ('to_sleep', 'lama1277-merde', 'turu'), ('to_sleep', 'lama1277-botun', 'turu'), ('to_sleep', 'lama1277-pukau', 'turu'), ('to_sleep', 'p-east2519', '*tia(r)'), ('to_sleep', 'p-timo1261', '*tia(r)'), ('to_sleep', 'lama1277-lamak', 'turu'), ('to_sleep', 'lama1277-lewob', 'turu'), ('to_sleep', 'lama1277-lewoi', 'ˈturu'), ('to_sleep', 'lama1277-horow', 'turuk'), ('to_sleep', 'lama1277-watan', 'turu'), ('to_sleep', 'alor1247-pandai', 'turu'), ('to_sleep', 'lama1277-belan', 'turu'), ('to_sleep', 'hama1240', 'tar'), ('to_sleep', 'adan1251-lawah', 'tara'), ('to_sleep', 'lama1277-lewom', 'turu'), ('to_sleep', 'lama1277-ritae', 'turu'), ('to_sleep', 'alor1247-baran', 'tur:u'), ('to_sleep', 'lama1277-lewog', 'turu'), ('to_sleep', 'lama1277-lamat', 'turʔo'), ('to_sleep', 'lama1277-dulhi', 'turu'), ('to_sleep', 'alor1247-munas', 'turu'), ('to_sleep', 'lama1277-adona', 'ˈturu'), ('to_sleep', 'lama1277-lamah', 'turu'), ('to_sleep', 'lama1277-waiba', 'turu'), ('to_sleep', 'p-mala1545-acd', '*tuduR'), ('to_sleep', 'lama1277-lamal', 'turu'), ('to_sleep', 'lama1277-ileap', 'turu'), ('to_sleep', 'lama1277-mulan', 'turu')}


Concept ‘to_ask_question’ is different between Alorese and Lamaholot.

 One or more Lamaholot dialects innovated forms not found in Alorese:
 {('to_ask_question', 'lama1277-lerek', 'ˈnəna')}
 {('to_ask_question', 'lama1277-kalik', 'ɡaˈləv')}
 {('to_ask_question', 'keda1252-leuba', "da'haŋ"), ('to_ask_question', 'lama1277-adona', 'ˈdahã'), ('to_ask_question', 'abui1241-takal', "hä.tä.'häŋ"), ('to_ask_question', 'abui1241-takal', "miŋ tä.'häŋ"), ('to_ask_question', 'atim1239', 'tatahaŋ'), ('to_ask_question', 'adan1251-otvai', 'uta’ʔain'), ('to_ask_question', 'abui1241-fuime', 'na:tahaŋ'), ('to_ask_question', 'lama1277-lerek', 'ˈdaha'), ('to_ask_question', 'kabo1247', 'hutaʔaiɲ'), ('to_ask_question', 'abui1241-ulaga', 'ɣetaɣaŋ'), ('to_ask_question', 'dein1238', 'taxan')}

  Just for clarity:
  There are also the following similarity classes shared between Alorese and LH.
  {('to_ask_question', 'alor1247-besar', 'gatɛ'), ('to_ask_question', 'alor1247-munas', "gə'tɛr"), ('to_ask_question', 'lama1277-lewoi', 'ˈɡətə'), ('to_ask_question', 'sarr1247-nule', 'gatag'), ('to_ask_question', 'rett1240', 'geteŋ'), ('to_ask_question', 'alor1247-pandai', "ga'tːer")}


Concept ‘day_before_yesterday’ is different between Alorese and Lamaholot.

 Alorese innovated the following forms:
 {('day_before_yesterday', 'alor1247-munas', "aru 'hɛlɛ"), ('day_before_yesterday', 'alor1247-munas', "ɛrua 'hɛlɛ"), ('day_before_yesterday', 'alor1247-pandai', "ɛrua 'hele")}
 {('day_before_yesterday', 'alor1247-besar', "ɛrua 'neka")}
 One or more Lamaholot dialects innovated forms not found in Alorese:
 {('day_before_yesterday', 'lama1277-kalik', 'ˈʤəma ˈʤua be'), ('day_before_yesterday', 'lama1277-lerek', 'ˈdʒ͡ama ˈdʒ͡ua be')}
 {('day_before_yesterday', 'lama1277-adona', "ʔɜrɜ rua 'ʔi"), ('day_before_yesterday', 'lama1277-lewoi', 'ˈərərəˈwia')}


Concept ‘who’ is different between Alorese and Lamaholot.

 Alorese innovated the following forms:
 {('who', 'alor1247-pandai', 'hewai'), ('who', 'alor1247-baran', 'haf:a'), ('who', 'alor1247-munas', 'hɛwai'), ('who', 'alor1247-munas', "hewai'aru")}
 {('who', 'alor1247-besar', 'fiaru')}
 Alorese shares the following forms with other AN languanges, but not Lamaholot:
 {('who', 'sika1262-tanai', 'hai'), ('who', 'sika1262-hewa', 'hai'), ('who', 'sika1262-maume', 'hai'), ('who', 'alor1247-munas', 'haru')}
 One or more Lamaholot dialects innovated forms not found in Alorese:
 {('who', 'lama1277-lewot', 'kena'), ('who', 'lama1277-lewuk', 'enak'), ('who', 'adan1251-otvai', '’a:no'), ('who', 'lama1277-kalik', 'eˈnak'), ('who', 'lama1277-lerek', 'eˈna'), ('who', 'lama1277-lewop', 'kena'), ('who', 'lama1277-lewok', 'enaku'), ('who', 'lama1277-minga', 'henaku'), ('who', 'lama1277-imulo', 'kena'), ('who', 'lama1277-paina', 'ena')}
 {('who', 'lama1277-lewom', 'hege'), ('who', 'lama1277-horow', 'heku'), ('who', 'lama1277-kalik', 'naku'), ('who', 'lama1277-ritae', 'hege'), ('who', 'lama1277-waiwa', 'hege'), ('who', 'lama1277-waiba', 'hege'), ('who', 'koto1251', 'sekau'), ('who', 'lama1277-lamak', 'heku'), ('who', 'lama1277-lamah', 'heku'), ('who', 'lama1277-baipi', 'hege'), ('who', 'lama1277-pukau', 'hege'), ('who', 'lama1277-watan', 'hege'), ('who', 'lama1277-lamat', 'he'), ('who', 'lama1277-lewoi', 'ˈheɡe'), ('who', 'lama1277-belan', 'heku'), ('who', 'lama1277-wuake', 'heku'), ('who', 'lama1277-lewoe', 'heʔe'), ('who', 'lama1277-dulhi', 'heku'), ('who', 'lama1277-tanju', 'hege'), ('who', 'lama1277-ileap', 'heku'), ('who', 'lama1277-botun', 'hege'), ('who', 'lama1277-lamal', 'heku'), ('who', 'lama1277-adona', 'ˈheku'), ('who', 'lama1277-kiwan', 'heku'), ('who', 'lama1277-lewob', 'hege'), ('who', 'lama1277-merde', 'heku'), ('who', 'lama1277-bama', 'hege'), ('who', 'lama1277-lewog', 'hege')}
 {('who', 'lama1277-mulan', 'ate'), ('who', 'idat1237', 'ʔɛta'), ('who', 'laka1255', 'ʔɛtɛ')}


Concept ‘to_bathe_a_child’ is different between Alorese and Lamaholot.

 One or more Lamaholot dialects innovated forms not found in Alorese:
 {('to_bathe_a_child', 'lama1277-kalik', 'ləˈbura')}

  Just for clarity:
  There are also the following similarity classes shared between Alorese and LH.
  {('to_bathe_a_child', 'alor1247-besar', "habɔ 'bɛka"), ('to_bathe_a_child', 'alor1247-baran', "hab:ɔ 'anaŋ"), ('to_bathe_a_child', 'alor1247-munas', "ha'bːɔ 'bai"), ('to_bathe_a_child', 'lama1277-adona', 'ˈhabo'), ('to_bathe_a_child', 'alor1247-pandai', "habːɔ 'bai"), ('to_bathe_a_child', 'lama1277-lerek', 'ləˈbo anaˈkən'), ('to_bathe_a_child', 'lama1277-lewoi', 'həˈbɔrɔ')}


Concept ‘thirsty’ is different between Alorese and Lamaholot.

 One or more Lamaholot dialects innovated forms not found in Alorese:
 {('thirsty', 'lama1277-kalik', 'ˈmai')}

  Just for clarity:
  There are also the following similarity classes shared between Alorese and LH.
  {('thirsty', 'lama1277-lerek', 'oremai'), ('thirsty', 'lama1277-adona', 'ɑnɜk ˈmara'), ('thirsty', 'lama1277-lewoi', 'ɔnə ˈmara'), ('thirsty', 'alor1247-munas', "onɔŋ 'marak"), ('thirsty', 'alor1247-besar', "onɔŋ 'mara"), ('thirsty', 'alor1247-munas', "onɔ 'marak"), ('thirsty', 'alor1247-pandai', "ɔnɔŋ 'mara")}


Concept ‘to_own’ is different between Alorese and Lamaholot.

 Alorese innovated the following forms:
 {('to_own', 'alor1247-besar', "go 'apa")}
 {('to_own', 'alor1247-pandai', "naŋ 'apa"), ('to_own', 'abui1241-ulaga', 'nei no: pa')}
 {('to_own', 'alor1247-munas', "apa 'ada")}
 Alorese shares the following forms with TAP languages:
 {('to_own', 'alor1247-besar', "go 'apa")}
 {('to_own', 'alor1247-pandai', "naŋ 'apa"), ('to_own', 'abui1241-ulaga', 'nei no: pa')}
 {('to_own', 'alor1247-munas', "apa 'ada")}
 One or more Lamaholot dialects innovated forms not found in Alorese:
 {('to_own', 'lama1277-kalik', 'ɡoˈen'), ('to_own', 'lama1277-adona', 'ɡoˈʔẽ')}
 {('to_own', 'lama1277-lerek', 'no anəˈmai')}
 {('to_own', 'lama1277-kalik', 'daˈen'), ('to_own', 'sika1262-hewa', 'noran'), ('to_own', 'lama1277-kalik', 'naˈen'), ('to_own', 'lama1277-lewoi', 'naˈʔɛŋ')}


Concept ‘mothers_brother’ is different between Alorese and Lamaholot.

 Alorese innovated the following forms:
 {('mothers_brother', 'alor1247-pandai', "pukɔŋ k'lake"), ('mothers_brother', 'alor1247-munas', "pukɔŋ k'lake"), ('mothers_brother', 'alor1247-besar', "am'pukɔŋ")}
 One or more Lamaholot dialects innovated forms not found in Alorese:
 {('mothers_brother', 'keda1252-leuba', 'epu'), ('mothers_brother', 'lama1277-adona', 'opu'), ('mothers_brother', 'lama1277-lewoi', 'epu')}


Concept ‘to_borrow’ is different between Alorese and Lamaholot.

 Alorese shares the following forms with other AN languanges, but not Lamaholot:
 {('to_borrow', 'keda1252-leuba', "bi'heʔ"), ('to_borrow', 'alor1247-munas', 'bihɛʔ'), ('to_borrow', 'alor1247-pandai', 'bihɛ'), ('to_borrow', 'alor1247-besar', 'bihɛ')}
 One or more Lamaholot dialects innovated forms not found in Alorese:
 {('to_borrow', 'lama1277-lewoi', 'məˈnaku')}
 {('to_borrow', 'lama1277-adona', 'ɡɜˈnerĩː')}


Concept ‘day_after_tomorrow’ is different between Alorese and Lamaholot.

 One or more Lamaholot dialects innovated forms not found in Alorese:
 {('day_after_tomorrow', 'sika1262-hewa', 'rema rua'), ('day_after_tomorrow', 'lama1277-kalik', 'ˈʤəma ˈʤua'), ('day_after_tomorrow', 'lama1277-lerek', 'dʒ͡əm ˈdʒ͡ua')}

  Just for clarity:
  There are also the following similarity classes shared between Alorese and LH.
  {('day_after_tomorrow', 'alor1247-munas', 'ɛrua '), ('day_after_tomorrow', 'alor1247-besar', 'ɛrua '), ('day_after_tomorrow', 'lama1277-lewoi', 'ərəˈrua'), ('day_after_tomorrow', 'alor1247-pandai', 'ɛrua '), ('day_after_tomorrow', 'lama1277-adona', "ʔɜrɜ 'rua")}


Concept ‘shoulder’ is different between Alorese and Lamaholot.

 One or more Lamaholot dialects innovated forms not found in Alorese:
 {('shoulder', 'lama1277-kalik', 'kvaˈlɛk'), ('shoulder', 'lama1277-lerek', 'ˈkwalek'), ('shoulder', 'keda1252-leuba', 'wali'), ('shoulder', 'sika1262-hewa', 'palik')}

  Just for clarity:
  There are also the following similarity classes shared between Alorese and LH.
  {('shoulder', 'alor1247-munas', 'hanaŋ'), ('shoulder', 'lama1277-lewoi', 'ˈhanaŋ'), ('shoulder', 'alor1247-pandai', 'hanaŋ'), ('shoulder', 'alor1247-besar', 'hanɛ̃'), ('shoulder', 'lama1277-adona', 'ˈhanaˈnɜt')}


Concept ‘fat’ is different between Alorese and Lamaholot.

 One or more Lamaholot dialects innovated forms not found in Alorese:
 {('fat', 'lama1277-horow', 'kənaharə'), ('fat', 'lama1277-adona', 'ˈkahare'), ('fat', 'lama1277-dulhi', 'kənaha'), ('fat', 'lama1277-waiwa', 'kənaha')}

  Just for clarity:
  There are also the following similarity classes shared between Alorese and LH.
  {('fat', 'keda1252', 'woyaʔ'), ('fat', 'lama1277-lamat', 'woraʔã'), ('fat', 'lama1277-lamah', 'worʔã'), ('fat', 'lama1277-wuake', 'worɁa'), ('fat', 'lama1277-kiwan', 'worʔã'), ('fat', 'alor1247-munas', 'wɔrak'), ('fat', 'lama1277-lamal', 'forã'), ('fat', 'lama1277-mulan', 'worɤ'), ('fat', 'lama1277-ritae', 'worã(kahal)'), ('fat', 'lama1277-botun', 'worʔã'), ('fat', 'lama1277-lewot', 'fojak'), ('fat', 'lama1277-tanju', 'worã'), ('fat', 'lama1277-lewoi', 'ˈwɔraŋ'), ('fat', 'lama1277-waiba', 'worã'), ('fat', 'sika1262-hewa', 'ʋura'), ('fat', 'lama1277-kalik', 'voˈʤak'), ('fat', 'lama1277-kalik', 'wojak'), ('fat', 'lama1277-lewom', 'worã'), ('fat', 'lama1277-merde', 'worã'), ('fat', 'lama1277-belan', 'wora'), ('fat', 'lama1277-lewok', 'wojak'), ('fat', 'alor1247-pandai', 'wɔrak'), ('fat', 'lama1277-ileap', 'worak'), ('fat', 'lama1277-imulo', 'fojakən'), ('fat', 'lama1277-lewog', 'worʔã'), ('fat', 'lama1277-lewoe', 'worõ'), ('fat', 'lama1277-minga', 'fojak'), ('fat', 'alor1247-besar', "fo'rakaŋ"), ('fat', 'lama1277-lamak', 'worʔã'), ('fat', 'lama1277-paina', 'wojak'), ('fat', 'lama1277-lewob', 'worã'), ('fat', 'lama1277-baipi', 'wora'), ('fat', 'lama1277-watan', 'worʔã'), ('fat', 'keda1252-leuba', "wɔ'jaʔ"), ('fat', 'lama1277-lerek', 'woˈjak'), ('fat', 'lama1277-lewop', 'fojak'), ('fat', 'sika1262-maume', 'vuran'), ('fat', 'sika1262-hewa', 'wura'), ('fat', 'lama1277-lewuk', 'wojak'), ('fat', 'lama1277-pukau', 'woraʔã'), ('fat', 'lama1277-bama', 'wora')}


Concept ‘to_promise’ is different between Alorese and Lamaholot.

 Alorese innovated the following forms:
 {('to_promise', 'alor1247-munas', "nuhu 'lɔdɔŋ")}
 One or more Lamaholot dialects innovated forms not found in Alorese:
 {('to_promise', 'lama1277-lewoi', 'ˈʤanʤi'), ('to_promise', 'keda1252-leuba', 'dʒɑndʒi')}
 {('to_promise', 'lama1277-adona', 'ˈbaja')}

  Just for clarity:
  There are also the following similarity classes shared between Alorese and LH.
  {('to_promise', 'lama1277-lerek', 'nəˈkaŋ'), ('to_promise', 'alor1247-pandai', 'nakːiŋ'), ('to_promise', 'alor1247-besar', 'nakiŋ'), ('to_promise', 'lamm1241-westp', 'nakiŋ')}


Concept ‘to_climb’ is different between Alorese and Lamaholot.

 One or more Lamaholot dialects innovated forms not found in Alorese:
 {('to_climb', 'p-mala1545-acd', '*dakiS'), ('to_climb', 'blag1240-warsa', '(tei) goa'), ('to_climb', 'p-aust1307-abvd', '*dakiS'), ('to_climb', 'p-mala1545-abvd', '*dakih'), ('to_climb', 'lama1277-kalik', 'tuˈkar'), ('to_climb', 'lama1277-lerek', 'ˈtuka')}

  Just for clarity:
  There are also the following similarity classes shared between Alorese and LH.
  {('to_climb', 'alor1247-munas', 'gɛrɛ'), ('to_climb', 'alor1247-pandai', 'gɛre'), ('to_climb', 'alor1247-besar', 'gɛrɛ'), ('to_climb', 'lama1277-adona', 'ˈɡere'), ('to_climb', 'lama1277-lewoi', 'ˈɡere')}


Concept ‘long’ is different between Alorese and Lamaholot.

 One or more Lamaholot dialects innovated forms not found in Alorese:
 {('long', 'lama1277-lerek', 'doˈak')}
 {('long', 'lama1277-lamat', 'doʔã'), ('long', 'lama1277-paina', 'doakən'), ('long', 'lama1277-lewuk', 'doak'), ('long', 'lama1277-lewok', 'doak'), ('long', 'lama1277-kalik', 'doˈak'), ('long', 'lama1277-kalik', 'doak')}
 {('long', 'lama1277-lewop', 'lafa'), ('long', 'lama1277-lewoe', 'bawã'), ('long', 'lama1277-imulo', 'lafakən'), ('long', 'keda1252', 'lawan')}

  Just for clarity:
  There are also the following similarity classes shared between Alorese and LH.
  {('long', 'alor1247-besar', "('kaʤo) bəla'hakaŋ"), ('long', 'alor1247-munas', "b'lahak"), ('long', 'lama1277-adona', 'bɜlahɜˈa'), ('long', 'lama1277-lewoi', 'bəˈlaha'), ('long', 'alor1247-baran', "ba'lah:a"), ('long', 'lama1277-adona', 'ˈblaha')}
  {('long', 'alor1247-munas', "b'lahak"), ('long', 'p-aust1307-abvd', '*inaduq'), ('long', 'lama1277-lewot', 'belamikən'), ('long', 'p-mala1545-abvd', '*anaduq'), ('long', 'lama1277-wuake', 'bəlakã'), ('long', 'lama1277-lewob', 'bəlahã'), ('long', 'lama1277-ileap', 'bəlahakən'), ('long', 'lama1277-botun', 'bəlahã'), ('long', 'lama1277-lamah', 'bəlahã'), ('long', 'lama1277-tanju', 'bəlaha'), ('long', 'lama1277-baipi', 'bəlahã'), ('long', 'lama1277-watan', 'bəlahã'), ('long', 'lama1277-horow', 'bəlahʔã'), ('long', 'lama1277-pukau', 'bahã'), ('long', 'lama1277-waiba', 'bəlahã'), ('long', 'alor1247-besar', "bəla'hakaŋ"), ('long', 'lama1277-ritae', 'bəlãhã'), ('long', 'lama1277-minga', 'bəlamik'), ('long', 'lama1277-lamal', 'blã'), ('long', 'lama1277-dulhi', 'bəlahã'), ('long', 'lama1277-waiwa', 'bəlaha'), ('long', 'lama1277-lamak', 'bəlahã'), ('long', 'lama1277-kiwan', 'bəlahʔã'), ('long', 'lama1277-bama', 'bəlaha'), ('long', 'lama1277-mulan', 'bələhɤ'), ('long', 'lama1277-merde', 'bəlahã'), ('long', 'alor1247-pandai', "b'lahak"), ('long', 'lama1277-lewom', 'bəlãha'), ('long', 'lama1277-belan', 'bəlawi'), ('long', 'lama1277-lewog', 'bəlaha')}


Concept ‘yesterday’ is different between Alorese and Lamaholot.

 One or more Lamaholot dialects innovated forms not found in Alorese:
 {('yesterday', 'lama1277-lerek', 'ɡaro'), ('yesterday', 'lama1277-kalik', 'nəɡəˈro')}

  Just for clarity:
  There are also the following similarity classes shared between Alorese and LH.
  {('yesterday', 'alor1247-pandai', 'wiaŋ'), ('yesterday', 'lama1277-adona', 'ˈwia'), ('yesterday', 'alor1247-munas', 'wiaŋ'), ('yesterday', 'alor1247-besar', 'fiaŋ'), ('yesterday', 'keda1252-leuba', "ʔɛ'wɪn"), ('yesterday', 'lama1277-lewoi', 'ˈwia')}


Concept ‘grandmother’ is different between Alorese and Lamaholot.

 Alorese innovated the following forms:
 {('grandmother', 'alor1247-pandai', "bapa k'wae"), ('grandmother', 'alor1247-besar', "bapaŋ ka'fae"), ('grandmother', 'alor1247-munas', "bapa k'wae")}
 One or more Lamaholot dialects innovated forms not found in Alorese:
 {('grandmother', 'lama1277-adona', 'wae')}
 {('grandmother', 'keda1252-leuba', 'ʔepu ʔarian'), ('grandmother', 'lama1277-lewoi', 'epu arian')}


Concept ‘to_hear’ is different between Alorese and Lamaholot.

 Alorese innovated the following forms:
 {('to_hear', 'alor1247-besar', 'haŋge')}
 One or more Lamaholot dialects innovated forms not found in Alorese:
 {('to_hear', 'lama1277-botun', 'bãi'), ('to_hear', 'lama1277-waiba', 'bãi'), ('to_hear', 'lama1277-lewob', 'bãi'), ('to_hear', 'lama1277-ritae', 'bãi'), ('to_hear', 'lama1277-lewom', 'bãi'), ('to_hear', 'lama1277-baipi', 'bãi'), ('to_hear', 'lama1277-pukau', 'bãi'), ('to_hear', 'lama1277-tanju', 'bãi')}

  Just for clarity:
  There are also the following similarity classes shared between Alorese and LH.
  {('to_hear', 'lama1277-dulhi', 'dəŋɤ'), ('to_hear', 'keda1252', 'deŋər'), ('to_hear', 'lama1277-lamal', 'dəŋa'), ('to_hear', 'p-mala1545-acd', '*diŋa'), ('to_hear', 'tetu1245-suai', 'rɔna'), ('to_hear', 'keda1252-leuwa', 'dæŋεr bæiŋ'), ('to_hear', 'sika1262-hewa', 'diʔin'), ('to_hear', 'lama1277-imulo', 'dəŋ'), ('to_hear', 'lama1277-lewok', 'dəŋər'), ('to_hear', 'lama1277-lewoe', 'dəŋe'), ('to_hear', 'lama1277-lewot', 'dəŋ'), ('to_hear', 'lama1277-lamat', 'dəŋe'), ('to_hear', 'sika1262-tanai', 'rəna'), ('to_hear', 'lama1277-bama', 'weŋe'), ('to_hear', 'p-mala1545-abvd', '*deŋeʀ'), ('to_hear', 'p-mala1545-acd', '*diŋeR'), ('to_hear', 'lama1277-adona', 'ˈdɜŋɜʔ'), ('to_hear', 'p-cent2245-abvd', '*dəŋəʀ'), ('to_hear', 'lama1277-merde', 'dəŋa'), ('to_hear', 'lama1277-lewuk', 'dəŋ'), ('to_hear', 'lama1277-watan', 'dəŋa'), ('to_hear', 'alor1247-munas', "də'ŋa"), ('to_hear', 'lama1277-lewog', 'weŋe'), ('to_hear', 'p-mala1545-acd', '*kiŋeR'), ('to_hear', 'lama1277-lamak', 'dəŋa'), ('to_hear', 'tetu1246', 'rɔna'), ('to_hear', 'lama1277-lewoi', 'ˈwɛŋɛ'), ('to_hear', 'keda1252-leuwa', 'baŋær'), ('to_hear', 'lama1277-wuake', 'dəŋə'), ('to_hear', 'sika1262-hewa', 'diʔiŋ'), ('to_hear', 'sika1262-hewa', 'rəna'), ('to_hear', 'tetu1245-vique', 'rɔna'), ('to_hear', 'p-mala1545-acd', '*deŋeR'), ('to_hear', 'alor1247-besar', 'daŋa'), ('to_hear', 'keda1252-leuba', 'dɛŋər'), ('to_hear', 'lama1277-lamah', 'dəŋa'), ('to_hear', 'alor1247-pandai', 'daŋa'), ('to_hear', 'alor1247-baran', 'daŋ:a'), ('to_hear', 'lama1277-kalik', 'daŋar'), ('to_hear', 'lama1277-lewop', 'dəŋ'), ('to_hear', 'lama1277-waiwa', 'weŋe'), ('to_hear', 'lama1277-mulan', 'dəŋa'), ('to_hear', 'lama1277-horow', 'dəŋɤ'), ('to_hear', 'lama1277-lerek', 'ˈdəŋə'), ('to_hear', 'lama1277-ileap', 'dəŋa'), ('to_hear', 'lama1277-minga', 'dəŋə'), ('to_hear', 'lama1277-kalik', 'ˈdəŋər'), ('to_hear', 'lama1277-kiwan', 'dəŋə'), ('to_hear', 'kema1243', 'rega'), ('to_hear', 'lama1277-paina', 'dəŋa'), ('to_hear', 'lama1277-belan', 'dəŋ')}


Concept ‘enemy’ is different between Alorese and Lamaholot.

 Alorese innovated the following forms:
 {('enemy', 'alor1247-besar', "onɔŋ 'datɛ")}
 {('enemy', 'alor1247-munas', 'nɛgɔŋ')}
 {('enemy', 'alor1247-pandai', "k'likil")}
 One or more Lamaholot dialects innovated forms not found in Alorese:
 {('enemy', 'lama1277-kalik', 'ˈoŋe')}
 {('enemy', 'lama1277-lewoi', 'bəˈliwɑŋ')}
 {('enemy', 'p-mala1545-acd', '*busuR'), ('enemy', 'lama1277-adona', 'ˈmusu'), ('enemy', 'koto1251', 'musu'), ('enemy', 'sika1262-hewa', 'musuh'), ('enemy', 'keda1252-leuba', 'musuh')}


Concept ‘to_grow’ is different between Alorese and Lamaholot.

 One or more Lamaholot dialects innovated forms not found in Alorese:
 {('to_grow', 'lama1277-kalik', 'ˈnubu')}

  Just for clarity:
  There are also the following similarity classes shared between Alorese and LH.
  {('to_grow', 'alor1247-pandai', 'tawaŋ'), ('to_grow', 'blag1240-kulij', 'tap:a'), ('to_grow', 'abui1241-fuime', 'tape'), ('to_grow', 'alor1247-munas', 'tawaŋ'), ('to_grow', 'keda1252-leuba', 'tawe'), ('to_grow', 'kula1280-lanto', 'atuba'), ('to_grow', 'lama1277-adona', 'ˈtawã'), ('to_grow', 'lama1277-lerek', 'ˈtava'), ('to_grow', 'alor1247-besar', 'tafa'), ('to_grow', 'lama1277-lewoi', 'ˈtawa')}


Concept ‘to_dig’ is different between Alorese and Lamaholot.

 One or more Lamaholot dialects innovated forms not found in Alorese:
 {('to_dig', 'lama1277-watan', 'baʔe'), ('to_dig', 'lama1277-botun', 'baʔe'), ('to_dig', 'lama1277-bama', 'pehe'), ('to_dig', 'lama1277-lamak', 'bake'), ('to_dig', 'fata1247', 'laki'), ('to_dig', 'lama1277-horow', 'baʔe'), ('to_dig', 'maka1316', 'toɁi'), ('to_dig', 'lama1277-kiwan', 'baʔe'), ('to_dig', 'lama1277-waiwa', 'baʔe'), ('to_dig', 'lama1277-wuake', 'gaɁe'), ('to_dig', 'lama1277-dulhi', 'baʔe'), ('to_dig', 'lama1277-adona', 'ˈbaʔe')}
 {('to_dig', 'lama1277-ileap', 'doka')}

  Just for clarity:
  There are also the following similarity classes shared between Alorese and LH.
  {('to_dig', 'lama1277-lamal', 'gui'), ('to_dig', 'lama1277-ritae', 'guĩ'), ('to_dig', 'lama1277-lewob', 'guʔi'), ('to_dig', 'lama1277-lewoi', 'ˈɡuʔi'), ('to_dig', 'alor1247-pandai', 'gui'), ('to_dig', 'lama1277-lerek', 'ˈɡurit'), ('to_dig', 'sika1262-tanai', 'ɡoʔi')}
  {('to_dig', 'lama1277-merde', 'gali'), ('to_dig', 'lama1277-lamat', 'gali'), ('to_dig', 'lama1277-mulan', 'gali'), ('to_dig', 'lama1277-lewuk', 'gali'), ('to_dig', 'rett1240', 'alu'), ('to_dig', 'alor1247-baran', 'gali'), ('to_dig', 'lama1277-lewog', 'gali'), ('to_dig', 'lama1277-baipi', 'gəlĩ'), ('to_dig', 'lama1277-imulo', 'gali'), ('to_dig', 'p-mala1545-acd', '*k<um>ali'), ('to_dig', 'lama1277-lewot', 'gali'), ('to_dig', 'sika1262-hewa', 'ɡali'), ('to_dig', 'lama1277-lewop', 'gali'), ('to_dig', 'sika1262-hewa', 'ʔali'), ('to_dig', 'lama1277-pukau', 'galiŋ'), ('to_dig', 'sika1262-maume', 'ʔali'), ('to_dig', 'p-aust1307-abvd', '*kalih'), ('to_dig', 'p-mala1545-abvd', '*kali'), ('to_dig', 'lama1277-lamah', 'gali'), ('to_dig', 'alor1247-munas', 'galiŋ'), ('to_dig', 'lama1277-lewok', 'gali'), ('to_dig', 'lama1277-kalik', 'galiŋ'), ('to_dig', 'keda1252-leuba', 'kaɛl'), ('to_dig', 'sika1262-hewa', 'a̤li'), ('to_dig', 'sika1262-tanai', 'ɡali'), ('to_dig', 'p-mala1545-acd', '*kali'), ('to_dig', 'lama1277-belan', 'gəli'), ('to_dig', 'lama1277-tanju', 'gəli'), ('to_dig', 'lama1277-minga', 'gali'), ('to_dig', 'lama1277-lewom', 'gəli'), ('to_dig', 'alor1247-besar', 'galiŋ'), ('to_dig', 'alor1247-pandai', 'galiŋ'), ('to_dig', 'lama1277-paina', 'gal'), ('to_dig', 'p-cent2245-abvd', '*keli'), ('to_dig', 'lama1277-lewoe', 'gɔli'), ('to_dig', 'lama1277-lerek', 'ɡal'), ('to_dig', 'p-cent2245-abvd', '*kali'), ('to_dig', 'keda1252', 'kael'), ('to_dig', 'lama1277-waiba', 'gəli'), ('to_dig', 'lama1277-kalik', 'ɡaˈli')}


Concept ‘womans_younger_sister’ is different between Alorese and Lamaholot.

 Alorese innovated the following forms:
 {('womans_younger_sister', 'alor1247-pandai', "kau k'wae"), ('womans_younger_sister', 'alor1247-pandai', "ula k'wae"), ('womans_younger_sister', 'alor1247-munas', "ulːa k'wae"), ('womans_younger_sister', 'alor1247-munas', "kau k'wae")}
 {('womans_younger_sister', 'alor1247-besar', "ariŋ ka'fae")}
 One or more Lamaholot dialects innovated forms not found in Alorese:
 {('womans_younger_sister', 'lama1277-lewoi', 'ariʔ (arian)'), ('womans_younger_sister', 'lama1277-adona', 'ʔariʔ'), ('womans_younger_sister', 'sika1262-hewa', 'ʋari'), ('womans_younger_sister', 'keda1252-leuba', 'ariʔ')}


Concept ‘thin’ is different between Alorese and Lamaholot.

 Alorese innovated the following forms:
 {('thin', 'alor1247-baran', 'kar:i')}
 One or more Lamaholot dialects innovated forms not found in Alorese:
 {('thin', 'lama1277-minga', 'bəlitis')}

  Just for clarity:
  There are also the following similarity classes shared between Alorese and LH.
  {('thin', 'lama1277-wuake', 'nipi'), ('thin', 'p-mala1545-acd', '*mipis'), ('thin', 'lama1277-lewop', 'mipi'), ('thin', 'p-cent2245-abvd', '*ma-nipis'), ('thin', 'lama1277-lewot', 'mənipifən'), ('thin', 'lama1277-lewog', 'mənipi'), ('thin', 'lama1277-baipi', 'mənipi'), ('thin', 'lama1277-kiwan', 'məniphi'), ('thin', 'lama1277-lamal', 'mənipi'), ('thin', 'alor1247-munas', 'nipi'), ('thin', 'lama1277-dulhi', 'mənipi'), ('thin', 'lama1277-pukau', 'nipi'), ('thin', 'lama1277-merde', 'bənipi'), ('thin', 'lama1277-waiba', 'mənipi'), ('thin', 'lama1277-lewom', 'mənipĩ'), ('thin', 'lama1277-ritae', 'mənipĩ'), ('thin', 'lama1277-belan', 'nipi'), ('thin', 'alor1247-besar', "ma'nipi"), ('thin', 'lama1277-botun', 'məniphĩ'), ('thin', 'lama1277-horow', 'məniphĩ'), ('thin', 'lama1277-tanju', 'nipi'), ('thin', 'alor1247-munas', 'nipi̤'), ('thin', 'lama1277-lewoi', 'məˈnipi'), ('thin', 'lama1277-watan', 'mənipi'), ('thin', 'lama1277-lewob', 'mənipi'), ('thin', 'lama1277-lamat', 'mipihi'), ('thin', 'keda1252-leuba', 'mipi'), ('thin', 'p-aust1307-abvd', '*ma-NiSepiS'), ('thin', 'p-mala1545-acd', '*tipis'), ('thin', 'lama1277-lewok', 'mipiwu'), ('thin', 'lama1277-lewuk', 'mipi'), ('thin', 'alor1247-pandai', 'nipi'), ('thin', 'lama1277-lerek', 'mipiu'), ('thin', 'p-mala1545-abvd', '*ma-nipis'), ('thin', 'alor1247-pandai', "mə'nipi"), ('thin', 'lama1277-waiwa', 'mənipi'), ('thin', 'lama1277-kalik', 'mipˈiv'), ('thin', 'lama1277-imulo', 'mipi'), ('thin', 'lama1277-ileap', 'nipi'), ('thin', 'keda1252-leuwa', 'mipi tεʔul'), ('thin', 'maka1316', 'nifihi'), ('thin', 'lama1277-lewoe', 'mipi'), ('thin', 'keda1252', 'mipi'), ('thin', 'lama1277-lamak', 'mənipi'), ('thin', 'lama1277-lamah', 'bənipi'), ('thin', 'lama1277-paina', 'mipiwən'), ('thin', 'lama1277-adona', 'mɜˈnipi'), ('thin', 'lama1277-mulan', 'bənipi'), ('thin', 'p-mala1545-acd', '*mepis'), ('thin', 'lama1277-kalik', 'mipiw'), ('thin', 'lama1277-bama', 'mənipi')}


Concept ‘door’ is different between Alorese and Lamaholot.

 One or more Lamaholot dialects innovated forms not found in Alorese:
 {('door', 'lama1277-lerek', 'ˈknaver'), ('door', 'lama1277-kalik', 'ˈknaver'), ('door', 'lama1277-lerek', 'kəˈnaver'), ('door', 'lama1277-kalik', 'knaˈvɛr'), ('door', 'lama1277-lewoi', 'kəˈnawe'), ('door', 'lama1277-kalik', 'ˈknavi')}

  Just for clarity:
  There are also the following similarity classes shared between Alorese and LH.
  {('door', 'alor1247-besar', 'pitu'), ('door', 'alor1247-munas', "pitu 'mataŋ"), ('door', 'lama1277-adona', 'ˈpita'), ('door', 'alor1247-pandai', 'pitɛ'), ('door', 'alor1247-munas', "pite 'mataŋ")}


Concept ‘small’ is different between Alorese and Lamaholot.

 Alorese shares the following forms with other AN languanges, but not Lamaholot:
 {('small', 'alor1247-munas', 'kihu'), ('small', 'kabo1247', 'kaʔai'), ('small', 'alor1247-pandai', 'kihu'), ('small', 'hama1240', 'kaʔ.i'), ('small', 'adan1251-lawah', 'kaʔai'), ('small', 'kema1243', "klɑ'ʔin"), ('small', 'adan1251-otvai', 'ka’ʔai')}
 One or more Lamaholot dialects innovated forms not found in Alorese:
 {('small', 'buna1278-suai', 'gɔl'), ('small', 'buna1278-bobon', 'gɔl'), ('small', 'buna1278-malia', ",ɛtɑŋ 'gɔl"), ('small', 'lama1277-mulan', 'kəre')}
 {('small', 'p-mala1545-abvd', '*kedi'), ('small', 'p-aust1307-abvd', '*kedi'), ('small', 'atim1239', 'kidiŋ'), ('small', 'p-mala1545-acd', '*bitiq'), ('small', 'buna1278-suai', 'ɛthan'), ('small', 'kama1365', 'kidiŋ'), ('small', 'baka1276', 'kuduk'), ('small', 'abui1241-ulaga', 'kidiŋ'), ('small', 'p-mala1545-acd', '*qitik '), ('small', 'blag1240-kulij', 'kud:u'), ('small', 'lama1277-lamak', 'kəda'), ('small', 'keda1252-leuba', "u'tuː"), ('small', 'p-mala1545-acd', '*kedi'), ('small', 'kafo1240', 'kɛdɪŋ'), ('small', 'sika1262-maume', 'kət̪ik'), ('small', 'blag1240-nule', 'kod:uk'), ('small', 'abui1241-fuime', 'kdiŋ'), ('small', 'keda1252', 'ṳtun'), ('small', 'kuii1253', 'kadín'), ('small', 'p-cent2245-abvd', '*kedi'), ('small', 'buna1278-bobon', 'ɛthan'), ('small', 'lama1277-wuake', 'kudu'), ('small', 'lama1277-watan', 'kedi'), ('small', 'abui1241-takal', "ki.'diŋ"), ('small', 'kira1248', 'kaˈdin')}
 {('small', 'blag1240-tuntu', 'kiki'), ('small', 'pura1258', 'kiki'), ('small', 'lama1277-ileap', 'uwekən'), ('small', 'blag1240-bama', 'kiki'), ('small', 'kaer1234', "ki'ki:"), ('small', 'lama1277-paina', 'kəlurəkən'), ('small', 'p-mala1545-acd', '*kikit'), ('small', 'kelo1247-bring', 'kɛkɛin'), ('small', 'lama1277-lewop', 'kəlurkən'), ('small', 'blag1240-warsa', 'kiki')}
 {('small', 'lama1277-dulhi', 'kusʔã')}
 {('small', 'lama1277-minga', 'bəluit'), ('small', 'lama1277-lewuk', 'bənut')}

  Just for clarity:
  There are also the following similarity classes shared between Alorese and LH.
  {('small', 'lama1277-merde', 'kesi'), ('small', 'lama1277-lewom', 'kəni'), ('small', 'alor1247-pandai', 'karːi'), ('small', 'lama1277-lamal', 'keni'), ('small', 'lama1277-horow', 'keni'), ('small', 'kelo1247-hopte', "kə'ni:t"), ('small', 'lama1277-botun', 'kəsĩ'), ('small', 'lama1277-kiwan', 'keni'), ('small', 'lamm1241-westp', 'kal:a'), ('small', 'lama1277-waiba', 'kəne'), ('small', 'lama1277-waiwa', 'kene'), ('small', 'alor1247-besar', 'kae'), ('small', 'lama1277-tanju', 'kəni'), ('small', 'lama1277-pukau', 'kəsiʔ'), ('small', 'lama1277-lewob', 'kərẽ'), ('small', 'lama1277-lamah', 'kesi'), ('small', 'lama1277-lewog', 'kəne'), ('small', 'lama1277-baipi', 'kəne'), ('small', 'lama1277-lewoi', 'ˈkənɛ'), ('small', 'lama1277-bama', 'kəni'), ('small', 'lama1277-adona', 'ˈkɛni')}
  {('small', 'lama1277-lewot', 'anak'), ('small', 'lama1277-belan', 'ana'), ('small', 'alor1247-baran', 'an:aŋ'), ('small', 'alor1247-munas', 'anaŋ'), ('small', 'koto1251', 'anaʔ'), ('small', 'lama1277-ritae', 'anã'), ('small', 'sika1262-hewa', 'anak'), ('small', 'lama1277-imulo', 'anakən'), ('small', 'lama1277-kalik', 'anak'), ('small', 'alor1247-pandai', 'anaŋ'), ('small', 'sika1262-tanai', 'anak'), ('small', 'lama1277-lewok', 'anak'), ('small', 'lama1277-lerek', 'anaˈkən'), ('small', 'lama1277-lewoe', 'kana'), ('small', 'lama1277-lamat', 'kanaʔã'), ('small', 'sarr1247-adiab', 'kəno:j'), ('small', 'lama1277-kalik', 'aˈnak')}


Concept ‘breadfruit’ is different between Alorese and Lamaholot.

 Alorese innovated the following forms:
 {('breadfruit', 'alor1247-pandai', "pə'loka"), ('breadfruit', 'alor1247-munas', "p'lɔka")}
 One or more Lamaholot dialects innovated forms not found in Alorese:
 {('breadfruit', 'lama1277-lewoi', 'ɡələˈweː')}

  Just for clarity:
  There are also the following similarity classes shared between Alorese and LH.
  {('breadfruit', 'lama1277-adona', 'tuˈnũ'), ('breadfruit', 'alor1247-pandai', 'tɔna')}
  {('breadfruit', 'rett1240', 'kundul'), ('breadfruit', 'lama1277-lerek', 'ˈkunur'), ('breadfruit', 'rett1240', 'kunul'), ('breadfruit', 'blag1240-warsa', 'kunur'), ('breadfruit', 'pura1258', 'undur'), ('breadfruit', 'kaer1234', "ku'nur"), ('breadfruit', 'alor1247-besar', 'kunʤu'), ('breadfruit', 'blag1240-kulij', 'undur'), ('breadfruit', 'blag1240-nule', 'undur'), ('breadfruit', 'baka1276', 'undur'), ('breadfruit', 'lama1277-kalik', 'kuˈnʊr'), ('breadfruit', 'blag1240-tuntu', "ku'nur"), ('breadfruit', 'blag1240-bama', 'kunur')}


Concept ‘to_stab’ is different between Alorese and Lamaholot.

 Alorese innovated the following forms:
 {('to_stab', 'alor1247-pandai', 'bunɔ')}
 Alorese shares the following forms with other AN languanges, but not Lamaholot:
 {('to_stab', 'p-cent2245-abvd', '*susuk'), ('to_stab', 'alor1247-pandai', 'tuhu'), ('to_stab', 'sika1262-tanai', 'rohuk'), ('to_stab', 'p-mala1545-abvd', '*suksuk'), ('to_stab', 'p-mala1545-acd', '*rusuk'), ('to_stab', 'idat1237', "tu'huk")}
 One or more Lamaholot dialects innovated forms not found in Alorese:
 {('to_stab', 'kaer1234', 'qaqo'), ('to_stab', 'lama1277-lewoi', 'ˈdəɡoʔ'), ('to_stab', 'sika1262-tanai', 'təɡu'), ('to_stab', 'lama1277-lewuk', 'rəgu')}
 {('to_stab', 'lama1277-botun', 'tubak'), ('to_stab', 'blag1240-kulij', 'ʤawa'), ('to_stab', 'p-mala1545-acd', '*tebek'), ('to_stab', 'lama1277-belan', 'tubak'), ('to_stab', 'lama1277-kiwan', 'tubak'), ('to_stab', 'sika1262-hewa', 'robak'), ('to_stab', 'lama1277-pukau', 'tubak '), ('to_stab', 'lama1277-lewog', 'tubak'), ('to_stab', 'lama1277-baipi', 'tubak'), ('to_stab', 'keda1252', 'tubaʔ'), ('to_stab', 'lama1277-kalik', 'tubak'), ('to_stab', 'sika1262-tanai', 'robak'), ('to_stab', 'lama1277-lewob', 'tuba'), ('to_stab', 'kuii1253', 'tapai'), ('to_stab', 'lama1277-lewoe', 'tuba'), ('to_stab', 'atim1239', 'tilok'), ('to_stab', 'lamm1241-westp', 'tap:aŋ'), ('to_stab', 'lama1277-imulo', 'tubak'), ('to_stab', 'lama1277-lewot', 'tubak'), ('to_stab', 'lama1277-horow', 'tubak'), ('to_stab', 'lama1277-wuake', 'tubak'), ('to_stab', 'lama1277-kalik', 'tuˈbak'), ('to_stab', 'lama1277-ileap', 'tuba'), ('to_stab', 'lama1277-minga', 'tubak'), ('to_stab', 'rett1240', 'ʤavak'), ('to_stab', 'lama1277-bama', 'tubak'), ('to_stab', 'lama1277-lewom', 'tubak'), ('to_stab', 'lama1277-ritae', 'tubak'), ('to_stab', 'lama1277-waiba', 'tubak '), ('to_stab', 'lama1277-merde', 'tubak'), ('to_stab', 'maka1316', 'tafa'), ('to_stab', 'lama1277-tanju', 'tubak'), ('to_stab', 'abui1241-takal', "tu.'lo:q"), ('to_stab', 'lama1277-mulan', 'tuba'), ('to_stab', 'lama1277-watan', 'tubak'), ('to_stab', 'lama1277-dulhi', 'tubak'), ('to_stab', 'abui1241-ulaga', 'ɣei tapɛ'), ('to_stab', 'lama1277-lamal', 'tuba'), ('to_stab', 'nede1245', 'tap:a'), ('to_stab', 'lama1277-lewop', 'tubak'), ('to_stab', 'lama1277-waiwa', 'tubak'), ('to_stab', 'p-mala1545-acd', '*t<um>ebek'), ('to_stab', 'lama1277-lamak', 'tubak'), ('to_stab', 'lama1277-lamat', 'tuba'), ('to_stab', 'lama1277-lamah', 'tubak'), ('to_stab', 'lama1277-lewok', 'tubak')}

  Just for clarity:
  There are also the following similarity classes shared between Alorese and LH.
  {('to_stab', 'lama1277-paina', 'həgat'), ('to_stab', 'alor1247-munas', 'sikaʔ'), ('to_stab', 'lama1277-adona', 'ˈsɜɡɑt'), ('to_stab', 'alor1247-pandai', 'sika'), ('to_stab', 'kafo1240', 'tɑkɑ'), ('to_stab', 'lama1277-lerek', 'həɡəˈtəŋ'), ('to_stab', 'alor1247-baran', 'sik:a'), ('to_stab', 'alor1247-besar', 'sika'), ('to_stab', 'kafo1240', 'tɑxɑ')}


Concept ‘kidney’ is different between Alorese and Lamaholot.

 Alorese innovated the following forms:
 {('kidney', 'alor1247-munas', "wɛ'huŋ")}
 Alorese shares the following forms with other AN languanges, but not Lamaholot:
 {('kidney', 'alor1247-pandai', 'gindʒal'), ('kidney', 'alor1247-besar', 'ginʤal'), ('kidney', 'sika1262-hewa', 'ginʤal')}
 One or more Lamaholot dialects innovated forms not found in Alorese:
 {('kidney', 'lama1277-adona', 'ˈwuak'), ('kidney', 'lama1277-lewoi', 'ˈwuakəŋ')}
 {('kidney', 'lama1277-lerek', 'keˈɡer'), ('kidney', 'lama1277-kalik', 'kɛk')}


Concept ‘blue’ is different between Alorese and Lamaholot.

 Alorese shares the following forms with TAP languages:
 {('blue', 'teiw1235', 'blau'), ('blue', 'alor1247-baran', "ba'lapã")}
 Alorese shares the following forms with other AN languanges, but not Lamaholot:
 {('blue', 'rett1240', 'iʤɔŋ'), ('blue', 'alor1247-pandai', 'taŋaŋ'), ('blue', 'alor1247-munas', 'idʒɔŋ'), ('blue', 'sika1262-hewa', 'daʔan')}
 One or more Lamaholot dialects innovated forms not found in Alorese:
 {('blue', 'lama1277-kalik', 'kəbəˈris')}
 {('blue', 'keda1252-leuba', 'biru'), ('blue', 'lama1277-lewoi', 'ˈbiru'), ('blue', 'koto1251', 'biru')}
 {('blue', 'lama1277-adona', 'ˈkeorɛ')}

  Just for clarity:
  There are also the following similarity classes shared between Alorese and LH.
  {('blue', 'lama1277-lerek', 'pəhəˈləm'), ('blue', 'rett1240', 'paheleŋ'), ('blue', 'alor1247-besar', "pa'helɛŋ")}


Concept ‘poor’ is different between Alorese and Lamaholot.

 Alorese innovated the following forms:
 {('poor', 'alor1247-besar', "mi'sikiŋ"), ('poor', 'pura1258', 'masikiŋ'), ('poor', 'wers1238-marit', 'miskin'), ('poor', 'abui1241-takal', "mäi.'si.kiŋ"), ('poor', 'rett1240', 'masikiŋ')}
 {('poor', 'alor1247-munas', "kuraŋ g'nasiŋ"), ('poor', 'sawi1256', "ku'rɔŋ kə'labi"), ('poor', 'kelo1247-hopte', "gunaŋ 'gab 'naŋ"), ('poor', 'abui1241-takal', "ku.räŋ kä.le̞.'bä")}
 {('poor', 'alor1247-pandai', "sukar 'tudak")}
 Alorese shares the following forms with TAP languages:
 {('poor', 'alor1247-besar', "mi'sikiŋ"), ('poor', 'pura1258', 'masikiŋ'), ('poor', 'wers1238-marit', 'miskin'), ('poor', 'abui1241-takal', "mäi.'si.kiŋ"), ('poor', 'rett1240', 'masikiŋ')}
 {('poor', 'alor1247-munas', "kuraŋ g'nasiŋ"), ('poor', 'sawi1256', "ku'rɔŋ kə'labi"), ('poor', 'kelo1247-hopte', "gunaŋ 'gab 'naŋ"), ('poor', 'abui1241-takal', "ku.räŋ kä.le̞.'bä")}
 {('poor', 'alor1247-pandai', "sukar 'tudak")}
 One or more Lamaholot dialects innovated forms not found in Alorese:
 {('poor', 'lama1277-lewoi', 'ˈkrideŋ')}
 {('poor', 'lama1277-adona', 'ɡuru ˈɡipa')}
 {('poor', 'lama1277-lerek', 'kəˈhian')}
 {('poor', 'lama1277-adona', 'ˈkuno')}
 {('poor', 'lama1277-kalik', 'snuˈsan')}


Concept ‘heart’ is different between Alorese and Lamaholot.

 Alorese shares the following forms with other AN languanges, but not Lamaholot:
 {('heart', 'wers1238-taram', 'geukabaŋ'), ('heart', 'blag1240-bama', 'kubaŋ'), ('heart', 'alor1247-munas', "tap'kubaŋ"), ('heart', 'blag1240-tuntu', "ku'baŋ"), ('heart', 'alor1247-besar', 'kubaŋ'), ('heart', 'blag1240-nule', 'kubaŋ'), ('heart', 'blag1240-kulij', 'kubaŋ'), ('heart', 'laka1255', 'khuɑ-n'), ('heart', 'maka1316', 'guɁu'), ('heart', 'pura1258', 'kubaŋ'), ('heart', 'wers1238-marit', "u'kabaŋ"), ('heart', 'alor1247-pandai', "tapɔ 'kubaŋ"), ('heart', 'blag1240-warsa', 'kubaŋ'), ('heart', 'baka1276', 'e kubaŋ')}
 One or more Lamaholot dialects innovated forms not found in Alorese:
 {('heart', 'buna1278-suai', 'huan'), ('heart', 'sika1262-hewa', 'wuan'), ('heart', 'buna1278-malia', 'huɑn'), ('heart', 'tetu1246', 'fuɑn'), ('heart', 'kema1243', 'hũar'), ('heart', 'tetu1245-vique', 'fuan'), ('heart', 'fata1247', 'uan'), ('heart', 'idat1237', 'huan'), ('heart', 'tuku1254', 'hu:'), ('heart', 'lama1277-lewom', 'wuak'), ('heart', 'tetu1245-suai', 'fuan')}
 {('heart', 'lama1277-lewoi', 'ˈʤɑntuŋ'), ('heart', 'sika1262-hewa', 'ʤantuŋ')}
 {('heart', 'lama1277-lewog', 'puhũ'), ('heart', 'lama1277-wuake', 'puho'), ('heart', 'lama1277-lewob', 'puhũ'), ('heart', 'lama1277-baipi', 'pəwuã'), ('heart', 'lama1277-ileap', 'puho'), ('heart', 'lama1277-waiba', 'puhũ'), ('heart', 'lama1277-lamah', 'puhokə'), ('heart', 'lama1277-dulhi', 'puho'), ('heart', 'lama1277-lamak', 'puho'), ('heart', 'lama1277-lerek', 'puon'), ('heart', 'lama1277-lewok', 'puo'), ('heart', 'p-mala1545-acd', '*pusuŋ'), ('heart', 'lama1277-lamal', 'puo'), ('heart', 'lama1277-imulo', 'puosa'), ('heart', 'koto1251', 'bua-f'), ('heart', 'lama1277-waiwa', 'puho'), ('heart', 'lama1277-merde', 'puho'), ('heart', 'lama1277-adona', 'ˈpuho'), ('heart', 'lama1277-tanju', 'puhũ'), ('heart', 'lama1277-lamat', 'puho'), ('heart', 'lama1277-minga', 'puo'), ('heart', 'lama1277-mulan', 'puhu'), ('heart', 'lama1277-lewop', 'puoha'), ('heart', 'lama1277-belan', 'puo'), ('heart', 'lama1277-lewoe', 'pũhũ'), ('heart', 'sika1262-tanai', 'puhu-ŋ βua-ŋ'), ('heart', 'lama1277-kiwan', 'puho'), ('heart', 'lama1277-lewuk', 'puo'), ('heart', 'lama1277-paina', 'puon'), ('heart', 'lama1277-lewot', 'puoha'), ('heart', 'lama1277-botun', 'puho'), ('heart', 'lama1277-ritae', 'puho'), ('heart', 'lama1277-bama', 'puhũ'), ('heart', 'lama1277-watan', 'puho'), ('heart', 'lama1277-kalik', 'puˈo'), ('heart', 'lama1277-kalik', 'puo'), ('heart', 'lama1277-horow', 'puho')}


Concept ‘feather’ is different between Alorese and Lamaholot.

 Alorese shares the following forms with other AN languanges, but not Lamaholot:
 {('feather', 'tuku1254', 'hulu'), ('feather', 'hama1240', 'falo'), ('feather', 'mamb1306', 'hulu'), ('feather', 'alor1247-pandai', 'wuluk'), ('feather', 'idat1237', "hu'luk"), ('feather', 'alor1247-besar', "fu'lukuŋ"), ('feather', 'koto1251', 'funu-f'), ('feather', 'kema1243', 'hulur'), ('feather', 'tetu1246', 'fulun'), ('feather', 'keda1252-leuba', "u'run"), ('feather', 'sika1262-tanai', 'βulu-ŋ'), ('feather', 'p-cent2245-abvd', '*bulu'), ('feather', 'p-mala1545-abvd', '*bulu'), ('feather', 'sika1262-hewa', 'wulu'), ('feather', 'tuku1254', 'manu hulu'), ('feather', 'sika1262-hewa', 'ʋulu'), ('feather', 'laka1255', 'hulun'), ('feather', 'alor1247-baran', "wu'luk"), ('feather', 'alor1247-munas', 'wuluk'), ('feather', 'p-mala1545-acd', '*bulu')}
 One or more Lamaholot dialects innovated forms not found in Alorese:
 {('feather', 'lama1277-paina', 'riaw')}
 {('feather', 'lama1277-lerek', 'kuˈkak ˈriav')}
 {('feather', 'lama1277-lewob', 'rawu'), ('feather', 'lama1277-bama', 'rawuk'), ('feather', 'lama1277-lamal', 'rafuk'), ('feather', 'lama1277-lewom', 'rawuk'), ('feather', 'lama1277-ritae', 'rawuk'), ('feather', 'lama1277-lewoi', 'rawuˈkəŋ'), ('feather', 'lama1277-lewuk', 'rawuk'), ('feather', 'lama1277-kiwan', 'rawuk'), ('feather', 'lama1277-merde', 'rawuk'), ('feather', 'lama1277-tanju', 'rawuk'), ('feather', 'lama1277-lewot', 'rafuk'), ('feather', 'lama1277-dulhi', 'rawuk'), ('feather', 'lama1277-baipi', 'rawuk'), ('feather', 'lama1277-imulo', 'rafukəy'), ('feather', 'lama1277-kalik', 'rawuk'), ('feather', 'lama1277-lamak', 'raʔwũ'), ('feather', 'lama1277-ileap', 'rawuk'), ('feather', 'lama1277-waiba', 'rawuk'), ('feather', 'lama1277-waiwa', 'rawuk'), ('feather', 'lama1277-lewop', 'rafuk'), ('feather', 'lama1277-adona', 'ˈrawuke'), ('feather', 'lama1277-lewok', 'rawuk'), ('feather', 'lama1277-wuake', 'rawũ'), ('feather', 'lama1277-belan', 'rawuk'), ('feather', 'lama1277-pukau', 'rawuʔũ'), ('feather', 'lama1277-lamat', 'rawuʔũ'), ('feather', 'lama1277-lewoe', 'rawũ'), ('feather', 'lama1277-lewog', 'rawuk'), ('feather', 'lama1277-minga', 'rafuk'), ('feather', 'lama1277-lamah', 'rawukə'), ('feather', 'lama1277-watan', 'rawuk'), ('feather', 'lama1277-kalik', 'ˈklie raˈvʊk'), ('feather', 'lama1277-mulan', 'rawuk'), ('feather', 'lama1277-botun', 'rawũ'), ('feather', 'lama1277-horow', 'rawuk')}


Concept ‘leg’ is different between Alorese and Lamaholot.

 One or more Lamaholot dialects innovated forms not found in Alorese:
 {('leg', 'lama1277-adona', 'ˈleikɜt')}

  Just for clarity:
  There are also the following similarity classes shared between Alorese and LH.
  {('leg', 'keda1252-leuba', 'lei'), ('leg', 'lama1277-kalik', 'li'), ('leg', 'lama1277-lewoi', 'ˈleiŋ'), ('leg', 'alor1247-baran', 'leiŋ'), ('leg', 'alor1247-pandai', 'leiŋ'), ('leg', 'alor1247-munas', 'leiʔ '), ('leg', 'lama1277-lerek', 'li'), ('leg', 'alor1247-besar', 'leiŋ'), ('leg', 'alor1247-munas', 'leiŋ')}


Concept ‘sacrifice’ is different between Alorese and Lamaholot.

 Alorese innovated the following forms:
 {('sacrifice', 'alor1247-besar', 'kaulu')}
 {('sacrifice', 'alor1247-pandai', 'lɔndɔ')}
 {('sacrifice', 'alor1247-munas', "raŋ 'mahu")}
 One or more Lamaholot dialects innovated forms not found in Alorese:
 {('sacrifice', 'keda1252-leuba', 'kɔrbɑn'), ('sacrifice', 'wers1238-marit', 'kɔrban'), ('sacrifice', 'lama1277-lewoi', 'ˈkɔrbɑn')}
 {('sacrifice', 'lama1277-kalik', 'umlaˈmak')}


Concept ‘light_not_dark’ is different between Alorese and Lamaholot.

 Alorese shares the following forms with TAP languages:
 {('light_not_dark', 'pura1258', 'ɟara'), ('light_not_dark', 'baka1276', 'ʤara'), ('light_not_dark', 'alor1247-besar', 'tarã'), ('light_not_dark', 'blag1240-nule', 'ʤara')}
 {('light_not_dark', 'alor1247-pandai', 'ɛluŋ'), ('light_not_dark', 'lamm1241-westp', 'diala'), ('light_not_dark', 'kula1280-lanto', '’dalaŋ'), ('light_not_dark', 'alor1247-munas', "ɛ'luŋ"), ('light_not_dark', 'wers1238-marit', "də'laŋ")}
 One or more Lamaholot dialects innovated forms not found in Alorese:
 {('light_not_dark', 'lama1277-kalik', 'bəneˈkən'), ('light_not_dark', 'lama1277-lerek', 'beneiˈkən')}
 {('light_not_dark', 'keda1252-leuba', 'nihɔ'), ('light_not_dark', 'lama1277-lewoi', 'ˈneʔa'), ('light_not_dark', 'lama1277-adona', 'ˈnɛʔɛk')}


Concept ‘sky’ is different between Alorese and Lamaholot.

 Alorese innovated the following forms:
 {('sky', 'alor1247-besar', 'kɔfa')}
 Alorese shares the following forms with other AN languanges, but not Lamaholot:
 {('sky', 'p-mala1545-abvd', '*laŋit'), ('sky', 'alor1247-munas', 'laŋi'), ('sky', 'p-cent2245-abvd', '*laŋit'), ('sky', 'p-aust1307-abvd', '*laŋiC'), ('sky', 'p-mala1545-acd', '*laŋit'), ('sky', 'kuii1253', 'laŋin'), ('sky', 'alor1247-baran', 'laŋi')}

  Just for clarity:
  There are also the following similarity classes shared between Alorese and LH.
  {('sky', 'lama1277-adona', 'ˈkɜlɜ'), ('sky', 'lama1277-mulan', 'kəlɤ'), ('sky', 'lama1277-imulo', 'kələm'), ('sky', 'alor1247-pandai', 'kalːeŋ'), ('sky', 'lama1277-watan', 'kəlã'), ('sky', 'lama1277-horow', 'kəlã'), ('sky', 'lama1277-lamah', 'kələm'), ('sky', 'lama1277-ileap', 'kələm'), ('sky', 'lama1277-dulhi', 'kələŋ'), ('sky', 'keda1252-leuba', "ʔɛ'leŋ"), ('sky', 'lama1277-lewot', 'kələmu'), ('sky', 'lama1277-baipi', 'kəlã'), ('sky', 'lama1277-wuake', 'kələm'), ('sky', 'lama1277-ritae', 'kəlã'), ('sky', 'keda1252-leuwa', 'ælæŋ'), ('sky', 'kama1365', 'deliŋ'), ('sky', 'lama1277-lewog', 'kəlã'), ('sky', 'lama1277-kiwan', 'kəlɤ'), ('sky', 'lama1277-kalik', 'kələm'), ('sky', 'lama1277-lewok', 'kələm'), ('sky', 'lama1277-lewop', 'kələm'), ('sky', 'lama1277-lewob', 'kəlã'), ('sky', 'lama1277-lewom', 'kəle'), ('sky', 'lama1277-kalik', 'kəˈləm'), ('sky', 'lama1277-lamal', 'kelã'), ('sky', 'lama1277-lewoe', 'əlẽ'), ('sky', 'lama1277-merde', 'kəlã'), ('sky', 'keda1252', 'eleŋ'), ('sky', 'lama1277-waiwa', 'kəlã'), ('sky', 'lama1277-lamat', 'əle'), ('sky', 'lama1277-paina', 'lewa'), ('sky', 'sika1262-hewa', 'kəleŋ'), ('sky', 'lama1277-lamak', 'kəlaŋ'), ('sky', 'lama1277-minga', 'kələm'), ('sky', 'lama1277-pukau', 'kulã'), ('sky', 'lama1277-lewoi', 'ˈkələŋ'), ('sky', 'lama1277-belan', 'kələm'), ('sky', 'lama1277-lewuk', 'kələm'), ('sky', 'lama1277-bama', 'kəlã'), ('sky', 'lama1277-botun', 'kəlã'), ('sky', 'lama1277-lerek', 'ˈkələm ˈleva'), ('sky', 'lama1277-waiba', 'kəlẽ'), ('sky', 'lama1277-tanju', 'kəlã')}


Concept ‘language’ is different between Alorese and Lamaholot.

 One or more Lamaholot dialects innovated forms not found in Alorese:
 {('language', 'lama1277-adona', 'ˈkoda')}
 {('language', 'keda1252-leuba', 'bahasa'), ('language', 'lama1277-lewoi', 'baˈhasa')}
 {('language', 'lama1277-lerek', 'makəˈtei')}

  Just for clarity:
  There are also the following similarity classes shared between Alorese and LH.
  {('language', 'lama1277-kalik', 'kniˈriŋ'), ('language', 'alor1247-pandai', "k'dirɛ"), ('language', 'alor1247-munas', "k'dirɛʔ"), ('language', 'alor1247-besar', 'kirɛ')}


Concept ‘chieftain’ is different between Alorese and Lamaholot.

 Alorese innovated the following forms:
 {('chieftain', 'alor1247-munas', "kotɔŋ 'huar")}
 {('chieftain', 'alor1247-pandai', "gam'beiŋ 'uma 'suku")}
 {('chieftain', 'alor1247-besar', "ka'pala 'lalaŋ")}
 One or more Lamaholot dialects innovated forms not found in Alorese:
 {('chieftain', 'lama1277-lewoi', 'kəˈbeləʔəŋ')}
 {('chieftain', 'lama1277-lerek', 'ˈata kaˈbelan')}
 {('chieftain', 'lama1277-adona', 'ˈsuku ˈmehene')}


Concept ‘to_hit_drum’ is different between Alorese and Lamaholot.

 Alorese innovated the following forms:
 {('to_hit_drum', 'alor1247-besar', "bɛhɛ 'bafa")}

  Just for clarity:
  There are also the following similarity classes shared between Alorese and LH.
  {('to_hit_drum', 'lama1277-adona', 'ˈdane'), ('to_hit_drum', 'lama1277-kalik', 'ˈdani'), ('to_hit_drum', 'alor1247-pandai', 'dane'), ('to_hit_drum', 'lama1277-lewoi', 'ˈdane'), ('to_hit_drum', 'alor1247-munas', "danɛ 'bawa"), ('to_hit_drum', 'lama1277-lerek', 'dan ˈbavar')}


Concept ‘hot’ is different between Alorese and Lamaholot.

 One or more Lamaholot dialects innovated forms not found in Alorese:
 {('hot', 'lama1277-kalik', 'kaˈti'), ('hot', 'lama1277-lewuk', 'kati'), ('hot', 'lama1277-imulo', 'kat'), ('hot', 'lama1277-lewot', 'kati'), ('hot', 'lama1277-lewok', 'kati'), ('hot', 'lama1277-minga', 'kati'), ('hot', 'lama1277-kalik', 'katiŋ'), ('hot', 'lama1277-lewop', 'kati'), ('hot', 'lama1277-kalik', 'knaˈtiŋ'), ('hot', 'lama1277-paina', 'katiŋən'), ('hot', 'lama1277-lerek', 'kənatiˈŋan')}

  Just for clarity:
  There are also the following similarity classes shared between Alorese and LH.
  {('hot', 'lama1277-dulhi', 'pəlate'), ('hot', 'lama1277-lamah', 'pəlate'), ('hot', 'lama1277-ileap', 'pəlate'), ('hot', 'lama1277-lamak', 'pəlate'), ('hot', 'lama1277-pukau', 'pate'), ('hot', 'lama1277-kiwan', 'pəlate'), ('hot', 'lama1277-baipi', 'pəlate'), ('hot', 'lama1277-tanju', 'pəlate'), ('hot', 'alor1247-munas', "p'latiŋ"), ('hot', 'lama1277-watan', 'pəlate'), ('hot', 'lama1277-waiwa', 'pəlate'), ('hot', 'lama1277-lewob', 'pəlate'), ('hot', 'lama1277-wuake', 'pəlate'), ('hot', 'lama1277-lamat', 'pati'), ('hot', 'lama1277-mulan', 'pəlate'), ('hot', 'lama1277-merde', 'pəlate'), ('hot', 'lama1277-bama', 'pəlate'), ('hot', 'lama1277-botun', 'pəlate'), ('hot', 'lama1277-waiba', 'pəlate'), ('hot', 'alor1247-besar', "pa'latiŋ"), ('hot', 'lama1277-lamal', 'pəlate'), ('hot', 'lama1277-lewog', 'pəlate'), ('hot', 'alor1247-baran', "pa'latiŋ"), ('hot', 'alor1247-pandai', "p'latiŋ"), ('hot', 'lama1277-lewom', 'pəlate'), ('hot', 'lama1277-ritae', 'pəlate'), ('hot', 'lama1277-belan', 'pəlate'), ('hot', 'lama1277-lewoe', 'pati'), ('hot', 'lama1277-lewoi', 'ˈpəlate'), ('hot', 'lama1277-adona', 'ˈplate'), ('hot', 'lama1277-horow', 'pəlate')}


Concept ‘chest’ is different between Alorese and Lamaholot.

 Alorese innovated the following forms:
 {('chest', 'alor1247-munas', 'kɔlɔʔ'), ('chest', 'alor1247-munas', 'kɔlɔŋ'), ('chest', 'kama1365', 'koraŋ'), ('chest', 'alor1247-pandai', 'kɔlɔŋ')}
 {('chest', 'alor1247-pandai', 'warːɛŋ'), ('chest', 'alor1247-besar', 'farɛ̃')}
 Alorese shares the following forms with TAP languages:
 {('chest', 'alor1247-munas', 'kɔlɔʔ'), ('chest', 'alor1247-munas', 'kɔlɔŋ'), ('chest', 'kama1365', 'koraŋ'), ('chest', 'alor1247-pandai', 'kɔlɔŋ')}
 {('chest', 'alor1247-pandai', 'warːɛŋ'), ('chest', 'alor1247-besar', 'farɛ̃')}
 One or more Lamaholot dialects innovated forms not found in Alorese:
 {('chest', 'lama1277-adona', 'ˈobaˈkɜt')}
 {('chest', 'lama1277-lerek', 'koˈrok'), ('chest', 'lama1277-lewoi', 'ˈkɔrɔk'), ('chest', 'keda1252-leuba', "ɔ'rɔʔ"), ('chest', 'lama1277-kalik', 'kɔˈrɔk')}


Concept ‘sole_of_foot’ is different between Alorese and Lamaholot.

 Alorese innovated the following forms:
 {('sole_of_foot', 'alor1247-munas', "lei 'ɔnɔŋ"), ('sole_of_foot', 'alor1247-munas', "leiŋ 'ɔnɔŋ"), ('sole_of_foot', 'alor1247-pandai', "leiŋ 'ɔnɔŋ"), ('sole_of_foot', 'alor1247-besar', "leiŋ 'ɔnɔŋ")}
 One or more Lamaholot dialects innovated forms not found in Alorese:
 {('sole_of_foot', 'lama1277-lewoi', 'ˈmakɑk')}
 {('sole_of_foot', 'lama1277-adona', 'leikɜt ˈʔonʔɜ')}
 {('sole_of_foot', 'lama1277-kalik', 'li əˈpak'), ('sole_of_foot', 'sika1262-hewa', 'ʋaʔi əpak'), ('sole_of_foot', 'lama1277-lerek', 'əˈpak')}


Concept ‘marriage’ is different between Alorese and Lamaholot.

 Alorese innovated the following forms:
 {('marriage', 'alor1247-besar', "haʤa 'nika")}
 {('marriage', 'alor1247-munas', "rale 'gute"), ('marriage', 'alor1247-pandai', "rale 'gute")}
 One or more Lamaholot dialects innovated forms not found in Alorese:
 {('marriage', 'sika1262-hewa', 'bɛrkɑt'), ('marriage', 'lama1277-adona', 'bɜrˈkaːt')}
 {('marriage', 'lama1277-kalik', 'kaˈvɛn'), ('marriage', 'lama1277-lerek', 'ˈkavɛn'), ('marriage', 'fata1247', 'kawe'), ('marriage', 'tetu1245-vique', 'kabɛŋ'), ('marriage', 'lama1277-lewoi', 'ˈkawɛn'), ('marriage', 'maka1316', "ka'wene")}


Concept ‘to_surrender’ is different between Alorese and Lamaholot.

 Alorese innovated the following forms:
 {('to_surrender', 'alor1247-munas', 'sɔmba'), ('to_surrender', 'alor1247-besar', 'ampɔ'), ('to_surrender', 'alor1247-pandai', 'sɔmba')}
 One or more Lamaholot dialects innovated forms not found in Alorese:
 {('to_surrender', 'lama1277-adona', 'soʔoˈtaʔ')}
 {('to_surrender', 'lama1277-lewoi', 'mənjeˈrah')}
 {('to_surrender', 'lama1277-lerek', 'ˈnotoŋ')}


Concept ‘to_split’ is different between Alorese and Lamaholot.

 One or more Lamaholot dialects innovated forms not found in Alorese:
 {('to_split', 'lama1277-kiwan', 'hiwək'), ('to_split', 'lama1277-lewom', 'sidak'), ('to_split', 'lama1277-dulhi', 'hiwək'), ('to_split', 'p-mala1545-abvd', '*silaq'), ('to_split', 'lama1277-botun', 'hiwək'), ('to_split', 'lama1277-horow', 'hiwak')}
 {('to_split', 'lama1277-waiba', 'giah'), ('to_split', 'kama1365', 'makarei'), ('to_split', 'koto1251', 'n-pora'), ('to_split', 'kama1365', 'karei'), ('to_split', 'mamb1306', 'fɛra')}
 {('to_split', 'lama1277-mulan', 'gika'), ('to_split', 'lama1277-lamah', 'gika'), ('to_split', 'lama1277-lewog', 'tika'), ('to_split', 'lama1277-bama', 'tika'), ('to_split', 'sika1262-hewa', 'hika'), ('to_split', 'lama1277-tanju', 'tika')}
 {('to_split', 'sika1262-hewa', 'plika'), ('to_split', 'lama1277-kalik', 'ləˈɡa'), ('to_split', 'lama1277-watan', 'ləka'), ('to_split', 'lama1277-lamal', 'ləka'), ('to_split', 'lama1277-adona', 'ˈlɜkɑʔ'), ('to_split', 'lama1277-lewot', 'ləgat'), ('to_split', 'lama1277-lewoi', 'ˈlikaʔ'), ('to_split', 'lama1277-lerek', 'ləˈkaŋ'), ('to_split', 'lama1277-lewok', 'ləga'), ('to_split', 'lama1277-merde', 'ləga'), ('to_split', 'sika1262-hewa', 'pleka'), ('to_split', 'lama1277-lewuk', 'ləga'), ('to_split', 'lama1277-belan', 'ləka'), ('to_split', 'lama1277-lamak', 'ləka'), ('to_split', 'lama1277-imulo', 'legata'), ('to_split', 'lama1277-wuake', 'ləka'), ('to_split', 'lama1277-lewob', 'ləga'), ('to_split', 'lama1277-lamat', 'ləgə'), ('to_split', 'lama1277-kalik', 'ləga'), ('to_split', 'lama1277-lewoe', 'ləgo'), ('to_split', 'lama1277-waiwa', 'ləka'), ('to_split', 'lama1277-minga', 'legat'), ('to_split', 'sika1262-maume', 'ləka'), ('to_split', 'lama1277-lewop', 'ləgat'), ('to_split', 'lama1277-paina', 'ləga')}

  Just for clarity:
  There are also the following similarity classes shared between Alorese and LH.
  {('to_split', 'alor1247-baran', 'bat:a'), ('to_split', 'keda1252', 'bete'), ('to_split', 'alor1247-besar', 'batːa'), ('to_split', 'keda1252-leuba', "bɛ'tɛ"), ('to_split', 'lama1277-ileap', 'bəta'), ('to_split', 'alor1247-pandai', "bə'tːa")}
  {('to_split', 'keda1252-leuwa', 'baʔ'), ('to_split', 'alor1247-munas', 'peaʔ'), ('to_split', 'buna1278-suai', 'phɛpha'), ('to_split', 'lama1277-baipi', 'bəlah'), ('to_split', 'lama1277-ritae', 'bəla'), ('to_split', 'buna1278-bobon', 'phɛpha'), ('to_split', 'alor1247-pandai', 'pea'), ('to_split', 'p-cent2245-abvd', '*bəlaq'), ('to_split', 'nede1245', 'bəlapa'), ('to_split', 'lama1277-pukau', 'bəla'), ('to_split', 'sika1262-tanai', 'kəlak'), ('to_split', 'p-mala1545-acd', '*beriq'), ('to_split', 'p-mala1545-abvd', '*belaq')}


Concept ‘bride_price’ is different between Alorese and Lamaholot.

 One or more Lamaholot dialects innovated forms not found in Alorese:
 {('bride_price', 'lama1277-lerek', 'ˈmaləhan')}

  Just for clarity:
  There are also the following similarity classes shared between Alorese and LH.
  {('bride_price', 'alor1247-besar', 'feliŋ'), ('bride_price', 'adan1251-otvai', '’fali'), ('bride_price', 'tetu1245-vique', 'fɔ:lɪŋ'), ('bride_price', 'sika1262-hewa', 'belis'), ('bride_price', 'alor1247-pandai', 'weli'), ('bride_price', 'lama1277-kalik', 'veˈli əˈlan'), ('bride_price', 'alor1247-munas', "k'wae 'wɛliŋ"), ('bride_price', 'lama1277-lerek', 'veˈlin'), ('bride_price', 'p-mala1545-acd', '*beli'), ('bride_price', 'lama1277-adona', 'weli'), ('bride_price', 'tetu1245-suai', 'belis'), ('bride_price', 'lama1277-lewoi', 'ˈweliŋ'), ('bride_price', 'alor1247-pandai', 'weliŋ'), ('bride_price', 'alor1247-besar', 'palaŋ')}


Concept ‘cold’ is different between Alorese and Lamaholot.

 Alorese innovated the following forms:
 {('cold', 'alor1247-baran', "ka'luaŋ"), ('cold', 'alor1247-pandai', "k'luaŋ"), ('cold', 'alor1247-munas', "k'luaŋ"), ('cold', 'alor1247-besar', 'kaluaŋ')}
 One or more Lamaholot dialects innovated forms not found in Alorese:
 {('cold', 'lama1277-lewob', 'loʔi')}
 {('cold', 'lama1277-lewom', 'gələtẽ'), ('cold', 'lama1277-pukau', 'gətã'), ('cold', 'lama1277-adona', 'ˈɡlɜtɜ'), ('cold', 'lama1277-lewoi', 'ˈɡələtə'), ('cold', 'lama1277-imulo', 'lətəs'), ('cold', 'lama1277-dulhi', 'gələtəŋ'), ('cold', 'lama1277-waiba', 'gələtə'), ('cold', 'lama1277-watan', 'gələtã'), ('cold', 'lama1277-wuake', 'lətəs'), ('cold', 'lama1277-lewot', 'lətəh'), ('cold', 'lama1277-kiwan', 'gələtɤ'), ('cold', 'lama1277-lewuk', 'lətəs'), ('cold', 'lama1277-mulan', 'leta'), ('cold', 'lama1277-lewok', 'lətəs'), ('cold', 'lama1277-lamah', 'ləta'), ('cold', 'lama1277-horow', 'gələtɤ̃'), ('cold', 'lama1277-lamak', 'gələtã'), ('cold', 'lama1277-belan', 'lətəs'), ('cold', 'lama1277-ritae', 'gələtã'), ('cold', 'lama1277-lewog', 'gələtə'), ('cold', 'lama1277-bama', 'gələtã'), ('cold', 'lama1277-lamat', 'lətha'), ('cold', 'lama1277-kalik', 'ləˈtəs'), ('cold', 'lama1277-botun', 'gələtã'), ('cold', 'lama1277-minga', 'lətəs'), ('cold', 'lama1277-tanju', 'gələtã'), ('cold', 'lama1277-lerek', 'lətəˈhən'), ('cold', 'lama1277-baipi', 'gələtã'), ('cold', 'lama1277-lewoe', 'lətɔ'), ('cold', 'lama1277-merde', 'lətã'), ('cold', 'lama1277-kalik', 'bələtəs'), ('cold', 'lama1277-lamal', 'gələtã'), ('cold', 'lama1277-paina', 'lətəhən'), ('cold', 'lama1277-ileap', '(gə)lətən'), ('cold', 'lama1277-waiwa', 'gələtã')}
 {('cold', 'lamm1241-westp', 'dag:a'), ('cold', 'lama1277-lewop', 'ləkə')}


Concept ‘fathers_sister’ is different between Alorese and Lamaholot.

 Alorese innovated the following forms:
 {('fathers_sister', 'alor1247-besar', "inaŋ 'pukɔŋ "), ('fathers_sister', 'alor1247-munas', "pukɔŋ k'wae"), ('fathers_sister', 'alor1247-pandai', "pukɔŋ k'wae")}
 One or more Lamaholot dialects innovated forms not found in Alorese:
 {('fathers_sister', 'lama1277-lewoi', 'binen'), ('fathers_sister', 'keda1252-leuba', 'binen')}
 {('fathers_sister', 'sika1262-hewa', 'kaka'), ('fathers_sister', 'lama1277-adona', 'kaka')}


Concept ‘now’ is different between Alorese and Lamaholot.

 Alorese innovated the following forms:
 {('now', 'alor1247-besar', 'hamuŋ')}
 {('now', 'alor1247-pandai', 'nuke'), ('now', 'lamm1241-westp', 'anaga')}
 {('now', 'alor1247-munas', 'nihu'), ('now', 'alor1247-pandai', 'nihu')}
 Alorese shares the following forms with TAP languages:
 {('now', 'alor1247-besar', 'hamuŋ')}
 {('now', 'alor1247-pandai', 'nuke'), ('now', 'lamm1241-westp', 'anaga')}
 {('now', 'alor1247-munas', 'nihu'), ('now', 'alor1247-pandai', 'nihu')}
 One or more Lamaholot dialects innovated forms not found in Alorese:
 {('now', 'lama1277-lerek', 'tea naˈbe')}
 {('now', 'lama1277-adona', 'ˈnhake ˈnhare')}
 {('now', 'lama1277-kalik', 've')}
 {('now', 'lama1277-lewoi', 'paˈliʔiŋ')}


Concept ‘to_sing’ is different between Alorese and Lamaholot.

 Alorese innovated the following forms:
 {('to_sing', 'alor1247-pandai', "kaŋ 'liaŋ"), ('to_sing', 'alor1247-besar', 'liaŋ'), ('to_sing', 'alor1247-munas', "kaŋ 'liaŋ"), ('to_sing', 'alor1247-baran', "bɔtɛ 'liaŋ")}
 One or more Lamaholot dialects innovated forms not found in Alorese:
 {('to_sing', 'sika1262-hewa', 'de:dɑŋ'), ('to_sing', 'lama1277-adona', 'ˈdedã')}

  Just for clarity:
  There are also the following similarity classes shared between Alorese and LH.
  {('to_sing', 'lama1277-mulan', 'ɲaɲi'), ('to_sing', 'alor1247-munas', "naŋ 'liaŋ"), ('to_sing', 'lama1277-bama', 'ɲaɲi'), ('to_sing', 'kema1243', "mə'nanit"), ('to_sing', 'lama1277-lewob', 'ɲaɲi'), ('to_sing', 'lama1277-lewom', 'ɲaɲi'), ('to_sing', 'lama1277-watan', 'ɲaɲi'), ('to_sing', 'lama1277-baipi', 'ɲaɲi'), ('to_sing', 'lama1277-pukau', 'ɲaɲi'), ('to_sing', 'lama1277-lamak', 'ɲaɲi'), ('to_sing', 'lama1277-lewog', 'ɲaɲi'), ('to_sing', 'lama1277-tanju', 'ɲaɲi'), ('to_sing', 'tetu1245-suai', "ha'nanu"), ('to_sing', 'lama1277-kiwan', 'ɲaɲi'), ('to_sing', 'lama1277-merde', 'ɲaɲi'), ('to_sing', 'lama1277-lewoi', 'ˈnjanji')}
  {('to_sing', 'tuku1254', 'kɑnta'), ('to_sing', 'lama1277-botun', 'kantar'), ('to_sing', 'lama1277-paina', 'kantar'), ('to_sing', 'lama1277-lamah', 'kantar'), ('to_sing', 'idat1237', 'kɑnta'), ('to_sing', 'sika1262-tanai', 'kantar'), ('to_sing', 'buna1278-bobon', 'kantar'), ('to_sing', 'lama1277-lewop', 'kantar'), ('to_sing', 'lama1277-imulo', 'kantar'), ('to_sing', 'lama1277-lewot', 'kantar'), ('to_sing', 'maka1316', 'ka:nta'), ('to_sing', 'lama1277-lerek', 'kanˈtar'), ('to_sing', 'lama1277-wuake', 'kantar'), ('to_sing', 'lama1277-waiba', 'kantar'), ('to_sing', 'lama1277-kalik', 'kantar'), ('to_sing', 'lama1277-kalik', 'kanˈtar'), ('to_sing', 'nede1245', 'pantuŋ'), ('to_sing', 'laka1255', 'kanta'), ('to_sing', 'lama1277-lamal', 'kantar'), ('to_sing', 'sika1262-hewa', 'kantar'), ('to_sing', 'lama1277-dulhi', 'kantar'), ('to_sing', 'lama1277-belan', 'kantar'), ('to_sing', 'alor1247-baran', 'pantɔ'), ('to_sing', 'lama1277-lewuk', 'kantar'), ('to_sing', 'fata1247', 'kanta'), ('to_sing', 'lama1277-lewoi', 'ˈkantar'), ('to_sing', 'buna1278-malia', 'kɑnta'), ('to_sing', 'lama1277-ritae', 'kantar'), ('to_sing', 'lama1277-lewok', 'kantar'), ('to_sing', 'tetu1245-vique', 'kanta'), ('to_sing', 'lama1277-horow', 'kantar'), ('to_sing', 'lama1277-waiwa', 'kantar'), ('to_sing', 'lama1277-lewoe', 'kata'), ('to_sing', 'lama1277-lamat', 'katərã'), ('to_sing', 'lama1277-ileap', 'kantar'), ('to_sing', 'lama1277-minga', 'katar'), ('to_sing', 'tetu1246', 'kɑnta'), ('to_sing', 'buna1278-suai', 'kantar')}


Concept ‘why’ is different between Alorese and Lamaholot.

 One or more Lamaholot dialects innovated forms not found in Alorese:
 {('why', 'teiw1235', 'taxaran'), ('why', 'adan1251-otvai', 'ta’ro ni(n)'), ('why', 'fata1247', "tɛa'nɛ"), ('why', 'lama1277-lewoi', 'dariˈaː'), ('why', 'kaer1234', 'taraŋ'), ('why', 'pura1258', "ea'talaŋ")}
 {('why', 'lama1277-lerek', 'nəroɡa')}

  Just for clarity:
  There are also the following similarity classes shared between Alorese and LH.
  {('why', 'lama1277-kalik', 'puˈkən an'), ('why', 'alor1247-baran', 'peinã:'), ('why', 'lama1277-adona', 'pukɜ ˈʔaku'), ('why', 'alor1247-pandai', "naməŋ'ga"), ('why', 'lama1277-lerek', 'naoɡa'), ('why', 'alor1247-pandai', 'naŋga'), ('why', 'alor1247-munas', "pai 'naŋ"), ('why', 'alor1247-pandai', "pai'naŋ"), ('why', 'alor1247-besar', "pa'rːnaŋ"), ('why', 'lama1277-kalik', 'bo an')}


Concept ‘to_bury’ is different between Alorese and Lamaholot.

 Alorese innovated the following forms:
 {('to_bury', 'alor1247-munas', 'nɛwaʔ')}
 {('to_bury', 'kaer1234', "tra'qo"), ('to_bury', 'alor1247-besar', 'tou'), ('to_bury', 'baka1276', 'tow'), ('to_bury', 'blag1240-bama', 'troku'), ('to_bury', 'blag1240-kulij', 'trou')}
 Alorese shares the following forms with TAP languages:
 {('to_bury', 'alor1247-munas', 'nɛwaʔ')}
 {('to_bury', 'kaer1234', "tra'qo"), ('to_bury', 'alor1247-besar', 'tou'), ('to_bury', 'baka1276', 'tow'), ('to_bury', 'blag1240-bama', 'troku'), ('to_bury', 'blag1240-kulij', 'trou')}
 One or more Lamaholot dialects innovated forms not found in Alorese:
 {('to_bury', 'lama1277-lerek', 'ˈtoman'), ('to_bury', 'lama1277-adona', 'ˈtomɜ')}
 {('to_bury', 'lama1277-lewoi', 'ˈrobok')}
 {('to_bury', 'lama1277-kalik', 'təˈni')}

  Just for clarity:
  There are also the following similarity classes shared between Alorese and LH.
  {('to_bury', 'keda1252-leuba', 'tanɛŋ'), ('to_bury', 'p-mala1545-acd', '*tanem'), ('to_bury', 'rett1240', 'tɔnɔŋ'), ('to_bury', 'alor1247-pandai', 'tɔnɔŋ'), ('to_bury', 'alor1247-munas', 'tɔnɔŋ'), ('to_bury', 'lama1277-adona', 'ˈtonɛk')}


Concept ‘round’ is different between Alorese and Lamaholot.

 Alorese shares the following forms with other AN languanges, but not Lamaholot:
 {('round', 'buna1278-malia', 'boluk'), ('round', 'alor1247-munas', 'pɔlik'), ('round', 'teiw1235', 'pug'), ('round', 'alor1247-pandai', 'pɔli'), ('round', 'buna1278-suai', 'bolugɔl'), ('round', 'keda1252-leuba', 'ʔɔpaŋ'), ('round', 'nede1245', 'puga')}
 One or more Lamaholot dialects innovated forms not found in Alorese:
 {('round', 'lama1277-kalik', 'klɔˈpɔr'), ('round', 'idat1237', "kapu'ɑr"), ('round', 'sika1262-tanai', 'ɡuər'), ('round', 'tetu1246', "kɑ'buɑr"), ('round', 'sika1262-hewa', 'ɡuer'), ('round', 'laka1255', "ka'buɑn"), ('round', 'lama1277-lerek', 'kəlopoˈrən'), ('round', 'tetu1245-vique', "ka'buar"), ('round', 'tetu1245-suai', "ka'buar")}
 {('round', 'lama1277-adona', 'kɜˈlubu')}
 {('round', 'lama1277-lewoi', 'ˈpunaŋ')}

  Just for clarity:
  There are also the following similarity classes shared between Alorese and LH.
  {('round', 'adan1251-lawah', 'koloŋ'), ('round', 'alor1247-baran', "ga'lɔkɔ"), ('round', 'alor1247-besar', "ga'lɔkɔ"), ('round', 'lama1277-kalik', 'kˈpɔlɔt'), ('round', 'adan1251-otvai', 'ka’dɔlaŋ'), ('round', 'sika1262-tanai', 'ɡulo')}


Concept ‘bird’ is different between Alorese and Lamaholot.

 One or more Lamaholot dialects innovated forms not found in Alorese:
 {('bird', 'lama1277-minga', 'apuanak')}
 {('bird', 'lama1277-adona', 'kɜˈsui')}
 {('bird', 'lama1277-lewob', 'kəpã')}
 {('bird', 'adan1251-otvai', 'bu’raŋ ’hiuw'), ('bird', 'kabo1247', 'siu'), ('bird', 'adan1251-otvai', '’hiuw'), ('bird', 'keda1252-leuba', "bu'ruŋ"), ('bird', 'lama1277-dulhi', 'burõ'), ('bird', 'hama1240', 'hif')}
 {('bird', 'lama1277-ileap', 'kukak'), ('bird', 'lama1277-lerek', 'kuˈkak')}
 {('bird', 'laka1255', 'manu'), ('bird', 'tetu1245-vique', "manu 'semo"), ('bird', 'tetu1245-suai', "manu 'semo"), ('bird', 'p-mala1545-acd', '*manu(k)-manuk'), ('bird', 'lama1277-lewoe', 'manuʔotõ'), ('bird', 'tetu1246', 'mɑnu'), ('bird', 'lama1277-watan', 'manuk utã'), ('bird', 'mamb1306', 'man hui'), ('bird', 'tuku1254', 'manu'), ('bird', 'idat1237', "manu ɑhu'ʔin"), ('bird', 'kema1243', 'manu'), ('bird', 'mamb1306', 'man'), ('bird', 'p-cent2245-abvd', '*manuk'), ('bird', 'lama1277-horow', 'manuk utã'), ('bird', 'p-mala1545-abvd', '*manuk'), ('bird', 'kema1243', "manu 'hui")}

  Just for clarity:
  There are also the following similarity classes shared between Alorese and LH.
  {('bird', 'lama1277-lamak', 'kolõ'), ('bird', 'maka1316', 'olo'), ('bird', 'lama1277-tanju', 'kolõ'), ('bird', 'lama1277-pukau', 'kolõ'), ('bird', 'lama1277-imulo', 'koloŋ'), ('bird', 'lama1277-lewom', 'kolõ'), ('bird', 'lama1277-ritae', 'kolõ'), ('bird', 'lama1277-lewuk', 'kəlieŋ'), ('bird', 'alor1247-pandai', 'kɔlɔŋ'), ('bird', 'abui1241-ulaga', 'kila'), ('bird', 'lama1277-merde', 'kolo'), ('bird', 'lama1277-baipi', 'kolõ'), ('bird', 'sika1262-maume', 'olon'), ('bird', 'lama1277-waiwa', 'kolõ'), ('bird', 'lama1277-lewop', 'koloŋ'), ('bird', 'keda1252', 'udaŋ oloŋ'), ('bird', 'lama1277-mulan', 'kolo'), ('bird', 'lama1277-lewog', 'kolõ'), ('bird', 'lama1277-kiwan', 'kolõ'), ('bird', 'lama1277-belan', 'kolo'), ('bird', 'alor1247-munas', 'kɔlɔŋ'), ('bird', 'lama1277-lamat', 'olõ'), ('bird', 'lama1277-kalik', 'kliˈɛŋ'), ('bird', 'lama1277-lewoi', 'ˈkɔlɔŋ'), ('bird', 'lama1277-paina', 'kəlieŋ'), ('bird', 'lama1277-waiba', 'kolõ'), ('bird', 'sika1262-hewa', 'olon'), ('bird', 'fata1247', 'ɔlɔ'), ('bird', 'lama1277-lamal', 'kolo'), ('bird', 'lama1277-lewot', 'koloŋu'), ('bird', 'lama1277-kalik', 'ˈklie'), ('bird', 'lama1277-botun', 'kolõ'), ('bird', 'koto1251', 'koro'), ('bird', 'alor1247-besar', 'koloŋ'), ('bird', 'lama1277-bama', 'kolõ'), ('bird', 'lama1277-kalik', 'kəlieŋ'), ('bird', 'lama1277-wuake', 'kolo'), ('bird', 'lama1277-lamah', 'kolo'), ('bird', 'alor1247-baran', 'koloŋ'), ('bird', 'lama1277-lewok', 'koloŋ')}


Concept ‘to_shoot_with_slingshot’ is different between Alorese and Lamaholot.

 Alorese innovated the following forms:
 {('to_shoot_with_slingshot', 'alor1247-pandai', 'fiti')}
 {('to_shoot_with_slingshot', 'alor1247-besar', "pasa nɔŋ kar'tapel"), ('to_shoot_with_slingshot', 'blag1240-nule', 'kartapel')}
 Alorese shares the following forms with TAP languages:
 {('to_shoot_with_slingshot', 'alor1247-pandai', 'fiti')}
 {('to_shoot_with_slingshot', 'alor1247-besar', "pasa nɔŋ kar'tapel"), ('to_shoot_with_slingshot', 'blag1240-nule', 'kartapel')}
 One or more Lamaholot dialects innovated forms not found in Alorese:
 {('to_shoot_with_slingshot', 'lama1277-lerek', 'pahaˈkəŋ no kataˈpel')}
 {('to_shoot_with_slingshot', 'lamm1241-westp', 'pasak:uŋ'), ('to_shoot_with_slingshot', 'dein1238', 'psaq'), ('to_shoot_with_slingshot', 'lama1277-kalik', 'pəˈsik'), ('to_shoot_with_slingshot', 'sika1262-hewa', 'pasɑk'), ('to_shoot_with_slingshot', 'sarr1247-nule', 'pəsa:k'), ('to_shoot_with_slingshot', 'sarr1247-adiab', "pə'sak"), ('to_shoot_with_slingshot', 'lama1277-lewoi', 'ˈpasɑk'), ('to_shoot_with_slingshot', 'lama1277-adona', 'ˈpɜsi'), ('to_shoot_with_slingshot', 'nede1245', 'pasaq:o')}


Concept ‘and’ is different between Alorese and Lamaholot.

 One or more Lamaholot dialects innovated forms not found in Alorese:
 {('and', 'lama1277-lewuk', 'tal')}
 {('and', 'lama1277-adona', 'ˈtaliʔ')}
 {('and', 'lama1277-kalik', 'pa')}
 {('and', 'lama1277-minga', 'we'), ('and', 'lama1277-lewot', 'fe')}
 {('and', 'adan1251-otvai', '’he'), ('and', 'lama1277-lamat', 'ʔo'), ('and', 'tetu1246', 'hɔ:'), ('and', 'buna1278-suai', 'hɔ'), ('and', 'buna1278-bobon', 'hɔ')}
 {('and', 'lama1277-merde', 'ko'), ('and', 'lama1277-lamah', 'kã'), ('and', 'p-mala1545-abvd', '*ka'), ('and', 'p-mala1545-acd', '*a'), ('and', 'p-aust1307-abvd', '*ka'), ('and', 'lama1277-kalik', 'ko'), ('and', 'p-mala1545-acd', '*ka')}
 {('and', 'lama1277-lamat', 'mo'), ('and', 'maka1316', 'mai'), ('and', 'p-mala1545-abvd', '*ma'), ('and', 'lama1277-kalik', 'mo'), ('and', 'sika1262-tanai', 'mole'), ('and', 'p-aust1307-abvd', '*mah'), ('and', 'koto1251', '#RIF!'), ('and', 'p-cent2245-abvd', '*ma'), ('and', 'p-mala1545-acd', '*mai'), ('and', 'lama1277-lamah', 'mã'), ('and', 'lama1277-merde', 'mo')}

  Just for clarity:
  There are also the following similarity classes shared between Alorese and LH.
  {('and', 'alor1247-baran', 'nɔ̃:'), ('and', 'lama1277-bama', 'no'), ('and', 'lama1277-tanju', 'noʔo'), ('and', 'lama1277-horow', 'noʔõ'), ('and', 'lama1277-ileap', 'no'), ('and', 'lama1277-waiwa', 'noʔõ'), ('and', 'alor1247-besar', 'nɔŋ'), ('and', 'lama1277-lewog', 'noʔõ'), ('and', 'lama1277-mulan', 'nɤ'), ('and', 'alor1247-pandai', 'naŋ'), ('and', 'sika1262-maume', 'nora'), ('and', 'sika1262-hewa', 'nora'), ('and', 'lama1277-kiwan', 'noʔõ'), ('and', 'lama1277-watan', 'noʔõ'), ('and', 'lama1277-adona', 'nɔʔ'), ('and', 'lama1277-lewob', 'noʔõ'), ('and', 'idat1237', 'nɔrɔ:'), ('and', 'kema1243', 'nɔ:'), ('and', 'lama1277-belan', 'no'), ('and', 'lama1277-botun', 'noʔõ'), ('and', 'tetu1245-suai', 'nɔ:'), ('and', 'lama1277-lewom', 'noʔõ'), ('and', 'lama1277-ritae', 'noʔõ'), ('and', 'lama1277-waiba', 'nã'), ('and', 'lama1277-baipi', 'nõ'), ('and', 'lama1277-lamal', 'nã'), ('and', 'lama1277-lewok', 'no'), ('and', 'lama1277-lerek', 'no naˈne'), ('and', 'keda1252-leuba', "no're"), ('and', 'lama1277-imulo', 'na'), ('and', 'lama1277-lewoi', 'ˈnɔʔɔŋ'), ('and', 'alor1247-munas', 'nəŋ'), ('and', 'lama1277-lewop', 'no'), ('and', 'tetu1245-vique', 'nɔ:'), ('and', 'lama1277-lamak', 'noʔõ'), ('and', 'keda1252', 'nore'), ('and', 'lama1277-dulhi', 'noʔo'), ('and', 'alor1247-munas', 'naŋ'), ('and', 'laka1255', 'nɔrɔ'), ('and', 'lama1277-lewoe', 'nu'), ('and', 'lama1277-wuake', 'no'), ('and', 'lama1277-kalik', 'no'), ('and', 'lama1277-paina', 'no'), ('and', 'lama1277-pukau', 'noʔõ'), ('and', 'mamb1306', 'nɔr')}


Concept ‘dark’ is different between Alorese and Lamaholot.

 Alorese shares the following forms with TAP languages:
 {('dark', 'teiw1235', "qa'Ɂan"), ('dark', 'sarr1247-nule', 'iqaʔan'), ('dark', 'alor1247-baran', 'kuiŋ'), ('dark', 'rett1240', 'kakua'), ('dark', 'alor1247-besar', 'kui'), ('dark', 'alor1247-munas', 'kuiŋ'), ('dark', 'abui1241-takal', "ä.'qun"), ('dark', 'alor1247-pandai', 'kuiŋ'), ('dark', 'pura1258', 'kua')}
 One or more Lamaholot dialects innovated forms not found in Alorese:
 {('dark', 'wers1238-taram', 'wudiŋ'), ('dark', 'lama1277-kalik', 'ˈmitəm'), ('dark', 'lama1277-lerek', 'eka mitəˈman'), ('dark', 'maka1316', "me'tana"), ('dark', 'lama1277-lewoi', 'ˈmitəŋ'), ('dark', 'wers1238-marit', "u'diŋ"), ('dark', 'kema1243', ",mɛta 'ma:n"), ('dark', 'keda1252-leuba', 'mitɛŋ')}
 {('dark', 'lama1277-kalik', 'rəˈmak'), ('dark', 'sika1262-hewa', 'rumaŋ')}
 {('dark', 'lama1277-adona', 'pɜˈroɡɜ')}


Concept ‘grandfather’ is different between Alorese and Lamaholot.

 Alorese innovated the following forms:
 {('grandfather', 'alor1247-besar', "bapaŋ ka'lake"), ('grandfather', 'alor1247-pandai', "bapa k'lake"), ('grandfather', 'alor1247-munas', "bapa k'lake")}
 One or more Lamaholot dialects innovated forms not found in Alorese:
 {('grandfather', 'lama1277-lewoi', 'epu'), ('grandfather', 'lama1277-adona', 'opu'), ('grandfather', 'maka1316', 'abo')}


Concept ‘big’ is different between Alorese and Lamaholot.

 One or more Lamaholot dialects innovated forms not found in Alorese:
 {('big', 'lama1277-lewop', 'duaŋən'), ('big', 'mamb1306', 'tu:'), ('big', 'idat1237', "ɑ'tun")}
 {('big', 'p-cent2245-abvd', '*ɾaya'), ('big', 'p-aust1307-abvd', '*ma-ʀaya'), ('big', 'lama1277-lerek', 'raˈjan'), ('big', 'lama1277-imulo', 'rajan'), ('big', 'p-mala1545-acd', '*Raya'), ('big', 'keda1252-leuba', "ri'aː"), ('big', 'p-mala1545-abvd', '*ma-ɾaya'), ('big', 'keda1252', 'rian')}
 {('big', 'lama1277-kalik', 'kedak'), ('big', 'lama1277-minga', 'kedak'), ('big', 'sika1262-maume', 'gət̪e'), ('big', 'sika1262-hewa', 'gəte'), ('big', 'lama1277-lewot', 'keda'), ('big', 'sika1262-tanai', 'ɡəte'), ('big', 'lama1277-lerek', 'keˈdak'), ('big', 'lama1277-paina', 'kedakən'), ('big', 'sika1262-hewa', 'gəteʔ'), ('big', 'lama1277-lewuk', 'keda'), ('big', 'lama1277-lewok', 'kedak'), ('big', 'lama1277-kalik', 'keˈdak')}

  Just for clarity:
  There are also the following similarity classes shared between Alorese and LH.
  {('big', 'rett1240', 'ɓal'), ('big', 'lama1277-merde', 'belã'), ('big', 'lama1277-baipi', 'beleʔ'), ('big', 'lama1277-botun', 'belʔã'), ('big', 'lama1277-horow', 'belʔã'), ('big', 'lama1277-lewob', 'belã'), ('big', 'lama1277-lamak', 'belʔã'), ('big', 'lama1277-tanju', 'belə'), ('big', 'alor1247-besar', 'beiŋ'), ('big', 'lama1277-lamah', 'belã'), ('big', 'lama1277-adona', 'ˈbele'), ('big', 'alor1247-munas', 'beaʔ'), ('big', 'lama1277-waiba', 'belã'), ('big', 'lama1277-kiwan', 'belʔã'), ('big', 'alor1247-baran', 'bĩ:'), ('big', 'lama1277-lamal', 'belã'), ('big', 'lama1277-lamat', 'bəlaʔã'), ('big', 'alor1247-munas', 'peiŋ'), ('big', 'rett1240', 'ɓa:l'), ('big', 'lama1277-waiwa', 'bele'), ('big', 'lama1277-wuake', 'belɁẽ'), ('big', 'lama1277-lewom', 'belẽ'), ('big', 'lama1277-pukau', 'bəlʔã'), ('big', 'lama1277-lewog', 'belə̃'), ('big', 'lama1277-belan', 'bel (duaŋ)'), ('big', 'lama1277-bama', 'belə'), ('big', 'lama1277-lewoi', 'ˈbelə'), ('big', 'lama1277-lewoe', 'bəle'), ('big', 'lama1277-mulan', 'belɤ'), ('big', 'alor1247-pandai', 'bea'), ('big', 'lama1277-ritae', 'belã'), ('big', 'lama1277-dulhi', 'belã'), ('big', 'lama1277-ileap', 'belən'), ('big', 'lama1277-watan', 'belʔã')}


Concept ‘to_bite’ is different between Alorese and Lamaholot.

 One or more Lamaholot dialects innovated forms not found in Alorese:
 {('to_bite', 'sika1262-hewa', 'baka'), ('to_bite', 'lama1277-lewoi', 'ˈbɑkɑ'), ('to_bite', 'sika1262-hewa', 'waka')}

  Just for clarity:
  There are also the following similarity classes shared between Alorese and LH.
  {('to_bite', 'lama1277-adona', 'ˈɡike'), ('to_bite', 'sika1262-maume', 'giʔi'), ('to_bite', 'fata1247', "kiki'Ɂɛ"), ('to_bite', 'lama1277-lewok', 'giki'), ('to_bite', 'kaer1234', "ki'ko"), ('to_bite', 'lama1277-belan', 'gike'), ('to_bite', 'keda1252-leuba', "ki'ʔi"), ('to_bite', 'keda1252', 'kiʔi'), ('to_bite', 'lama1277-lewog', 'gike'), ('to_bite', 'lama1277-imulo', 'gik'), ('to_bite', 'maka1316', "ga'ele"), ('to_bite', 'buna1278-suai', 'gi:'), ('to_bite', 'lama1277-mulan', 'gike'), ('to_bite', 'lama1277-lamah', 'gike'), ('to_bite', 'alor1247-pandai', 'gaki'), ('to_bite', 'alor1247-baran', 'gaki'), ('to_bite', 'lama1277-minga', 'giki'), ('to_bite', 'alor1247-besar', 'gaki'), ('to_bite', 'lama1277-horow', 'gike'), ('to_bite', 'lama1277-botun', 'gike'), ('to_bite', 'buna1278-malia', 'gi:'), ('to_bite', 'lama1277-lewop', 'giki'), ('to_bite', 'rett1240', "ki'ki"), ('to_bite', 'lama1277-lamal', 'goki'), ('to_bite', 'lama1277-watan', 'gike'), ('to_bite', 'lama1277-lewom', 'gike'), ('to_bite', 'lama1277-kalik', 'ˈɡike'), ('to_bite', 'lama1277-lamat', 'giʔi'), ('to_bite', 'lama1277-paina', 'gik'), ('to_bite', 'blag1240-kulij', 'gaki'), ('to_bite', 'lama1277-lewoe', 'giʔi'), ('to_bite', 'lama1277-ritae', 'gike'), ('to_bite', 'lama1277-lamak', 'gike'), ('to_bite', 'tuku1254', 'ka:'), ('to_bite', 'lama1277-kalik', 'ˈɡiki'), ('to_bite', 'lama1277-pukau', 'gike'), ('to_bite', 'lama1277-dulhi', 'gike'), ('to_bite', 'lama1277-lerek', '(ˈaor) ˈɡike'), ('to_bite', 'lama1277-kiwan', 'gike'), ('to_bite', 'lama1277-lewot', 'gike'), ('to_bite', 'lama1277-tanju', 'gike'), ('to_bite', 'lamm1241-westp', 'kak:uŋ'), ('to_bite', 'p-timo1261', '*ki(l)'), ('to_bite', 'lama1277-bama', 'gike'), ('to_bite', 'lama1277-lewob', 'gori'), ('to_bite', 'p-alor1249', '*(ta)ki'), ('to_bite', 'baka1276', 'ga:kik'), ('to_bite', 'blag1240-nule', 'gaki'), ('to_bite', 'p-east2519', '*(ga)gel'), ('to_bite', 'lama1277-baipi', 'gike'), ('to_bite', 'lama1277-merde', 'gike'), ('to_bite', 'lama1277-lewuk', 'giki'), ('to_bite', 'lama1277-lewoi', 'ˈɡike'), ('to_bite', 'lama1277-waiwa', 'gike'), ('to_bite', 'buna1278-bobon', 'gi:'), ('to_bite', 'keda1252-leuwa', 'kiʔi ka'), ('to_bite', 'lama1277-kalik', 'giki'), ('to_bite', 'lama1277-wuake', 'gike'), ('to_bite', 'rett1240', 'kiki'), ('to_bite', 'alor1247-munas', 'gakiʔ'), ('to_bite', 'lama1277-waiba', 'gike'), ('to_bite', 'lama1277-ileap', 'gike')}


Concept ‘ridge’ is different between Alorese and Lamaholot.

 Alorese innovated the following forms:
 {('ridge', 'alor1247-munas', "b'lɔkɔŋ"), ('ridge', 'lamm1241-westp', 'bla gaʔuŋ'), ('ridge', 'alor1247-pandai', "b'lokɔŋ")}
 {('ridge', 'alor1247-pandai', 'nuka')}
 Alorese shares the following forms with TAP languages:
 {('ridge', 'alor1247-munas', "b'lɔkɔŋ"), ('ridge', 'lamm1241-westp', 'bla gaʔuŋ'), ('ridge', 'alor1247-pandai', "b'lokɔŋ")}
 {('ridge', 'alor1247-pandai', 'nuka')}
 One or more Lamaholot dialects innovated forms not found in Alorese:
 {('ridge', 'lama1277-adona', 'ˈwolaʔ'), ('ridge', 'lama1277-lerek', 'ˈvolar'), ('ridge', 'keda1252-leuba', 'wɔlar')}

  Just for clarity:
  There are also the following similarity classes shared between Alorese and LH.
  {('ridge', 'p-mala1545-acd', '*bubuŋ'), ('ridge', 'lama1277-lerek', 'ˈuvuŋ'), ('ridge', 'kama1365', 'aifaŋ'), ('ridge', 'p-mala1545-acd', '*bubuŋ-en'), ('ridge', 'lama1277-lewoi', 'ˈuwuŋ'), ('ridge', 'sika1262-hewa', 'puʋun'), ('ridge', 'koto1251', 'ʔpupu-n'), ('ridge', 'p-mala1545-acd', '*bubuŋ-an'), ('ridge', 'lamm1241-westp', 'bibis'), ('ridge', 'lama1277-kalik', 'uˈvʊŋ'), ('ridge', 'alor1247-besar', "mou'fufuŋ")}


Concept ‘knee’ is different between Alorese and Lamaholot.

 Alorese innovated the following forms:
 {('knee', 'alor1247-besar', "kana'duluŋ")}

  Just for clarity:
  There are also the following similarity classes shared between Alorese and LH.
  {('knee', 'lama1277-kalik', 'loˈtɔr'), ('knee', 'sika1262-tanai', 'tur'), ('knee', 'sika1262-tanai', 'βaʔi-ŋ tur'), ('knee', 'tetu1245-vique', "ai'tur"), ('knee', 'alor1247-munas', "leiŋ 'kudul"), ('knee', 'fata1247', 'tɕul'), ('knee', 'alor1247-munas', "lei 'kudul"), ('knee', 'sika1262-hewa', 'tur'), ('knee', 'koto1251', 'tu:-f'), ('knee', 'keda1252-leuwa', 'udul'), ('knee', 'lama1277-adona', 'ˈlotorɜt'), ('knee', 'alor1247-baran', ",leiŋ 'kudul"), ('knee', 'tetu1246', 'ʔɑin tur'), ('knee', 'tetu1245-suai', "ai'tur"), ('knee', 'idat1237', "wein 'tur"), ('knee', 'alor1247-pandai', 'kudul'), ('knee', 'lama1277-lerek', 'loˈtor'), ('knee', 'lama1277-lewoi', 'ˈlɔtɔr'), ('knee', 'keda1252-leuba', 'ʔudul'), ('knee', 'alor1247-munas', 'kudul')}


Concept ‘to_close’ is different between Alorese and Lamaholot.

 Alorese innovated the following forms:
 {('to_close', 'alor1247-pandai', 'likɔ')}
 {('to_close', 'alor1247-pandai', 'hɔbɔ')}
 One or more Lamaholot dialects innovated forms not found in Alorese:
 {('to_close', 'lama1277-kalik', 'ˈtuŋən')}
 {('to_close', 'keda1252-leuba', "lɛ'tuʔ"), ('to_close', 'lama1277-adona', 'ˈlɜːtuʔ'), ('to_close', 'lama1277-lewoi', 'ˈlətuʔ')}

  Just for clarity:
  There are also the following similarity classes shared between Alorese and LH.
  {('to_close', 'alor1247-besar', 'tɛra'), ('to_close', 'tetu1245-suai', 'taka'), ('to_close', 'alor1247-pandai', 'tɛra'), ('to_close', 'lamm1241-westp', 'dauruŋ'), ('to_close', 'alor1247-munas', 'tɛraʔ'), ('to_close', 'dein1238', 'tiar'), ('to_close', 'lamm1241-westp', 'tiariŋ'), ('to_close', 'lama1277-lerek', 'ihɛˈrəŋ'), ('to_close', 'tetu1245-vique', 'taka'), ('to_close', 'p-alor1249', '*-tiari(n)'), ('to_close', 'kula1280-lanto', 'tira')}


Concept ‘head’ is different between Alorese and Lamaholot.

 One or more Lamaholot dialects innovated forms not found in Alorese:
 {('head', 'kabo1247', 'lo:ŋ'), ('head', 'p-aust1307-abvd', '*quluh'), ('head', 'mamb1306', "ʔulu 'hɑtu"), ('head', 'laka1255', "ulu 'hatu"), ('head', 'lama1277-imulo', 'ulusa'), ('head', 'sika1262-maume', 'alan'), ('head', 'lama1277-kalik', 'ulun'), ('head', 'lama1277-minga', 'ulu'), ('head', 'tetu1245-suai', 'ulun'), ('head', 'lama1277-paina', 'ulug'), ('head', 'lama1277-lerek', 'uˈlu'), ('head', 'lama1277-lewot', 'uluha'), ('head', 'lama1277-lewop', 'ulu'), ('head', 'p-mala1545-abvd', '*qulu'), ('head', 'adan1251-lawah', 'loŋ'), ('head', 'sika1262-tanai', 'ala-ŋ'), ('head', 'lama1277-kalik', 'uˈlu'), ('head', 'p-cent2245-abvd', '*qulu'), ('head', 'lama1277-lewuk', 'ulu'), ('head', 'tetu1246', 'ʔulun'), ('head', 'tetu1245-vique', 'ulun'), ('head', 'lama1277-lewok', 'uluga'), ('head', 'p-mala1545-acd', '*qulu')}

  Just for clarity:
  There are also the following similarity classes shared between Alorese and LH.
  {('head', 'alor1247-munas', 'kɔtɔʔ'), ('head', 'lama1277-lewob', 'kotə'), ('head', 'lama1277-lamah', 'kotəkə'), ('head', 'lama1277-mulan', 'kotə'), ('head', 'lama1277-ritae', 'kotã'), ('head', 'alor1247-besar', 'kɔtɔŋ'), ('head', 'lama1277-lewoi', 'ˈkɔtəʔ'), ('head', 'kelo1247-hopte', 'gitu'), ('head', 'lama1277-lewog', 'kotã'), ('head', 'lama1277-wuake', 'kotẽ'), ('head', 'lama1277-horow', 'kotək'), ('head', 'lama1277-adona', 'kotɜm'), ('head', 'lama1277-lamak', 'kotʔã'), ('head', 'lama1277-pukau', 'kotã'), ('head', 'alor1247-munas', 'kɔtɔŋ'), ('head', 'lama1277-baipi', 'kotət'), ('head', 'lama1277-merde', 'kotəkət'), ('head', 'lama1277-watan', 'kotʔã'), ('head', 'lama1277-botun', 'kotək'), ('head', 'lama1277-tanju', 'kotək'), ('head', 'lama1277-lamat', 'əte'), ('head', 'lama1277-waiba', 'kotə'), ('head', 'lama1277-lewoe', 'əte'), ('head', 'lama1277-adona', 'ˈkotɜk'), ('head', 'lama1277-belan', 'kotək'), ('head', 'alor1247-baran', 'kɔtɔŋ'), ('head', 'alor1247-pandai', 'kɔtɔŋ'), ('head', 'lama1277-ileap', 'kotɤ'), ('head', 'lama1277-bama', 'kote'), ('head', 'lama1277-lamal', 'kotãte'), ('head', 'lama1277-lewom', 'kotə'), ('head', 'lama1277-dulhi', 'kotəŋ'), ('head', 'kelo1247-bring', 'kədɛh'), ('head', 'lama1277-kiwan', 'kotək'), ('head', 'lama1277-waiwa', 'kotã')}


Concept ‘meat’ is different between Alorese and Lamaholot.

 One or more Lamaholot dialects innovated forms not found in Alorese:
 {('meat', 'lama1277-mulan', 'nawək')}
 {('meat', 'lama1277-lewob', 'mətã'), ('meat', 'sika1262-tanai', 'mei-ŋ ʔəta-ŋ'), ('meat', 'sika1262-maume', 'ʔət̪an'), ('meat', 'lama1277-waiwa', 'mətã'), ('meat', 'lama1277-pukau', 'mətã'), ('meat', 'sika1262-hewa', 'ətan')}
 {('meat', 'lama1277-minga', 'eləm'), ('meat', 'lama1277-lerek', 'eˈlam'), ('meat', 'lama1277-imulo', 'elam'), ('meat', 'lama1277-lamat', 'elã'), ('meat', 'lama1277-lewot', 'elam'), ('meat', 'lama1277-lewop', 'elam'), ('meat', 'lama1277-kalik', 'eˈlam'), ('meat', 'lama1277-wuake', 'elã'), ('meat', 'lama1277-lamal', 'elã'), ('meat', 'lama1277-lewuk', 'elam'), ('meat', 'lama1277-merde', 'elã'), ('meat', 'lama1277-paina', 'elam'), ('meat', 'lama1277-kalik', 'elam'), ('meat', 'lama1277-lamah', 'ela'), ('meat', 'lama1277-lewoe', 'əlã'), ('meat', 'lama1277-lewok', 'elaŋ')}
 {('meat', 'lama1277-belan', 'bənək')}
 {('meat', 'lama1277-dulhi', 'əwã')}
 {('meat', 'lama1277-botun', 'umã'), ('meat', 'lama1277-horow', 'umã'), ('meat', 'lama1277-adona', 'ˈʔumɜ̃')}
 {('meat', 'tetu1246', 'na:n'), ('meat', 'keda1252-leuba', "na'ʔɛn"), ('meat', 'tetu1245-vique', 'naɁan'), ('meat', 'lama1277-lewoi', 'ˈmənakəŋ'), ('meat', 'tetu1245-suai', 'naɁan'), ('meat', 'lama1277-baipi', 'mənakẽ')}

  Just for clarity:
  There are also the following similarity classes shared between Alorese and LH.
  {('meat', 'alor1247-pandai', "dagiŋ 'ihi"), ('meat', 'lama1277-lamak', 'dagiŋ'), ('meat', 'lama1277-waiba', 'dagiŋ'), ('meat', 'blag1240-nule', 'dagiŋ hi:')}
  {('meat', 'lama1277-lewom', 'ihik'), ('meat', 'lama1277-ritae', 'ihik'), ('meat', 'lama1277-watan', 'ihik'), ('meat', 'alor1247-baran', 'ʔihik'), ('meat', 'lama1277-lamal', 'hik'), ('meat', 'buna1278-bobon', 'si:'), ('meat', 'koto1251', 'sisi'), ('meat', 'p-east2519', '*seor'), ('meat', 'sika1262-tanai', 'ihi-ŋ'), ('meat', 'p-mala1545-abvd', '*hesi'), ('meat', 'laka1255', 'sisi'), ('meat', 'p-aust1307-abvd', '*Sesi'), ('meat', 'lama1277-lewog', 'ihik'), ('meat', 'keda1252', 'ihin'), ('meat', 'lama1277-ileap', 'ihin'), ('meat', 'p-mala1545-abvd', '*isi'), ('meat', 'tuku1254', 'sisi'), ('meat', 'buna1278-malia', 'si:'), ('meat', 'p-timo1261', '*isor'), ('meat', 'buna1278-suai', 'si:'), ('meat', 'p-aust1307-abvd', '*isi'), ('meat', 'keda1252-leuwa', 'i̘hiŋ'), ('meat', 'alor1247-munas', 'hik'), ('meat', 'wers1238-marit', 'sis'), ('meat', 'kema1243', 'si:'), ('meat', 'blag1240-kulij', 'ehi'), ('meat', 'mamb1306', 'sɪs'), ('meat', 'p-cent2245-abvd', '*isi'), ('meat', 'idat1237', 'sisi'), ('meat', 'lama1277-tanju', 'ihik'), ('meat', 'lama1277-kiwan', 'ihik'), ('meat', 'maka1316', 'seu'), ('meat', 'p-mala1545-acd', '*hesi'), ('meat', 'alor1247-besar', "i'hikiŋ"), ('meat', 'lama1277-bama', 'ihik')}


Concept ‘chin’ is different between Alorese and Lamaholot.

 Alorese innovated the following forms:
 {('chin', 'alor1247-munas', 'bamɛʔ'), ('chin', 'alor1247-pandai', 'bamaŋ'), ('chin', 'alor1247-besar', 'bamɛŋ'), ('chin', 'alor1247-munas', 'bamɛŋ')}
 One or more Lamaholot dialects innovated forms not found in Alorese:
 {('chin', 'lama1277-adona', 'ˈsaroˈnɜt')}
 {('chin', 'lama1277-lewoi', 'ˈkɔrɛŋ')}
 {('chin', 'lama1277-kalik', 'ɡrim'), ('chin', 'lama1277-lerek', 'ɡəˈrimə')}


Concept ‘hill_ridge’ is different between Alorese and Lamaholot.

 Alorese shares the following forms with TAP languages:
 {('hill_ridge', 'alor1247-munas', 'dɔlu'), ('hill_ridge', 'alor1247-besar', 'dɔla'), ('hill_ridge', 'wers1238-marit', 'dɔl'), ('hill_ridge', 'kelo1247-hopte', "də'dɔl")}
 One or more Lamaholot dialects innovated forms not found in Alorese:
 {('hill_ridge', 'lama1277-adona', 'woka'), ('hill_ridge', 'lama1277-lewoi', 'wɔka')}
 {('hill_ridge', 'lama1277-lerek', 'ˈwuhun')}

  Just for clarity:
  There are also the following similarity classes shared between Alorese and LH.
  {('hill_ridge', 'sika1262-hewa', 'ʋolon'), ('hill_ridge', 'alor1247-pandai', 'wɔtɔ'), ('hill_ridge', 'lama1277-kalik', 'ˈvolo'), ('hill_ridge', 'alor1247-munas', "wɔtɔ 'wɛrɔʔ"), ('hill_ridge', 'lama1277-kalik', 'ˈvoloŋ')}


Concept ‘frog’ is different between Alorese and Lamaholot.

 One or more Lamaholot dialects innovated forms not found in Alorese:
 {('frog', 'lama1277-lerek', 'ɡurˈɡeŋ')}
 {('frog', 'lama1277-kalik', 'tuˈtəŋ')}
 {('frog', 'lama1277-kalik', 'ˈtulɔr'), ('frog', 'fata1247', 'cila'), ('frog', 'maka1316', 'dile'), ('frog', 'lama1277-kalik', 'tulu')}

  Just for clarity:
  There are also the following similarity classes shared between Alorese and LH.
  {('frog', 'lama1277-adona', 'ˈmɜto'), ('frog', 'alor1247-besar', 'matɔ'), ('frog', 'lama1277-kalik', 'kəməˈtɔr'), ('frog', 'alor1247-pandai', "ma'tːɔ"), ('frog', 'blag1240-nule', "mo'to"), ('frog', 'baka1276', 'moto'), ('frog', 'lama1277-lewoi', 'ˈmətɔ'), ('frog', 'alor1247-munas', "mə'tːɔ")}


Concept ‘older_sibling’ is different between Alorese and Lamaholot.

 One or more Lamaholot dialects innovated forms not found in Alorese:
 {('older_sibling', 'lama1277-lewoe', 'ʔaʔa'), ('older_sibling', 'lama1277-lamat', 'ʔaʔã'), ('older_sibling', 'keda1252', 'ʔaʔe')}

  Just for clarity:
  There are also the following similarity classes shared between Alorese and LH.
  {('older_sibling', 'lama1277-lewuk', 'tata'), ('older_sibling', 'blag1240-nule', "neta'ta"), ('older_sibling', 'alor1247-munas', 'tata'), ('older_sibling', 'keda1252-leuba', 'tata'), ('older_sibling', 'lama1277-lamal', 'tata'), ('older_sibling', 'lama1277-imulo', 'tata'), ('older_sibling', 'alor1247-besar', 'tata'), ('older_sibling', 'lama1277-lewog', 'tata'), ('older_sibling', 'alor1247-baran', 'tataŋ'), ('older_sibling', 'sika1262-hewa', 'tata'), ('older_sibling', 'lama1277-bama', 'tataʔ'), ('older_sibling', 'lama1277-kalik', 'taˈta'), ('older_sibling', 'blag1240-kulij', 'tata'), ('older_sibling', 'alor1247-pandai', 'tata'), ('older_sibling', 'koto1251', 'tataf')}
  {('older_sibling', 'lama1277-adona', 'kaka'), ('older_sibling', 'lama1277-lewot', 'kaka'), ('older_sibling', 'fata1247', 'kaka'), ('older_sibling', 'lama1277-pukau', 'kakaʔ'), ('older_sibling', 'idat1237', "ka'ak"), ('older_sibling', 'buna1278-suai', 'khaɁa'), ('older_sibling', 'lama1277-lamak', 'kaka'), ('older_sibling', 'alor1247-pandai', 'kakaŋ'), ('older_sibling', 'lama1277-mulan', 'kaka'), ('older_sibling', 'lama1277-minga', 'kaka'), ('older_sibling', 'lama1277-paina', 'kakay'), ('older_sibling', 'lama1277-lamah', 'kakakə'), ('older_sibling', 'mamb1306', 'ka:'), ('older_sibling', 'lama1277-ileap', 'kaka'), ('older_sibling', 'lama1277-lerek', 'kaka'), ('older_sibling', 'lama1277-wuake', 'kaka'), ('older_sibling', 'lama1277-lewop', 'kaka'), ('older_sibling', 'lama1277-kalik', 'kaka'), ('older_sibling', 'rett1240', 'gakaku'), ('older_sibling', 'maka1316', 'kaka'), ('older_sibling', 'lama1277-waiba', 'kaka'), ('older_sibling', 'lama1277-adona', 'kakãm'), ('older_sibling', 'lama1277-tanju', 'kaka'), ('older_sibling', 'p-mala1545-acd', '*kaka'), ('older_sibling', 'lama1277-baipi', 'kakaʔ'), ('older_sibling', 'lama1277-kiwan', 'kaka'), ('older_sibling', 'lama1277-lewok', 'kaka'), ('older_sibling', 'lama1277-dulhi', 'kaka'), ('older_sibling', 'lama1277-ritae', 'kaka'), ('older_sibling', 'lama1277-belan', 'kaka'), ('older_sibling', 'lama1277-waiwa', 'kakaʔ'), ('older_sibling', 'lama1277-lewoi', 'ˈkakɑŋ'), ('older_sibling', 'lama1277-merde', 'kaka'), ('older_sibling', 'lama1277-horow', 'kaka'), ('older_sibling', 'alor1247-munas', 'kakaŋ'), ('older_sibling', 'lama1277-botun', 'kaka'), ('older_sibling', 'lama1277-lewob', 'kaka'), ('older_sibling', 'lama1277-watan', 'kaka'), ('older_sibling', 'buna1278-bobon', 'khaɁa'), ('older_sibling', 'lama1277-lewom', 'kaka')}


Concept ‘earthquake’ is different between Alorese and Lamaholot.

 Alorese innovated the following forms:
 {('earthquake', 'alor1247-pandai', "tana 'otaŋ"), ('earthquake', 'alor1247-munas', "ɛkaŋ 'ɔtaŋ"), ('earthquake', 'alor1247-besar', "tana 'ɔʤaŋ"), ('earthquake', 'alor1247-pandai', "ɛkaŋ 'otaŋ")}
 One or more Lamaholot dialects innovated forms not found in Alorese:
 {('earthquake', 'lama1277-lerek', 'ˈbredoŋ'), ('earthquake', 'lama1277-kalik', 'ˈblero ɛˈkok'), ('earthquake', 'lama1277-adona', 'brero'), ('earthquake', 'lama1277-lewoi', 'bəˈlero ˈredɔʔ')}


Concept ‘house’ is different between Alorese and Lamaholot.

 Alorese shares the following forms with other AN languanges, but not Lamaholot:
 {('house', 'alor1247-besar', 'uma'), ('house', 'koto1251', 'ume'), ('house', 'maka1316', 'oma'), ('house', 'alor1247-munas', 'uma'), ('house', 'p-aust1307-abvd', '*ʀumaq'), ('house', 'p-cent2245-abvd', '*ʀumaq'), ('house', 'tetu1245-suai', 'uma'), ('house', 'alor1247-pandai', 'uma'), ('house', 'p-mala1545-abvd', '*ʀumaq'), ('house', 'p-mala1545-acd', '*Rumaq'), ('house', 'tetu1246', 'ʔuma'), ('house', 'tetu1245-vique', 'uma'), ('house', 'mamb1306', 'ʔum'), ('house', 'kema1243', 'ʔuma'), ('house', 'alor1247-baran', 'ʔuma')}
 One or more Lamaholot dialects innovated forms not found in Alorese:
 {('house', 'lama1277-lewoi', 'ˈlaŋɔʔ'), ('house', 'lama1277-adona', 'ˈlaŋɔ')}
 {('house', 'lama1277-kalik', 'uˈnan'), ('house', 'lama1277-kalik', 'ˈuna'), ('house', 'lama1277-lerek', 'ˈunan')}


Concept ‘wind’ is different between Alorese and Lamaholot.

 One or more Lamaholot dialects innovated forms not found in Alorese:
 {('wind', 'blag1240-kulij', 'enar'), ('wind', 'blag1240-bama', 'enar'), ('wind', 'blag1240-nule', 'enar'), ('wind', 'lama1277-pukau', 'ləmur'), ('wind', 'blag1240-warsa', 'enar'), ('wind', 'kelo1247-hopte', "nə'mɛr"), ('wind', 'kelo1247-bring', 'nəmɛr'), ('wind', 'laka1255', 'kamɑr'), ('wind', 'baka1276', 'enar'), ('wind', 'dein1238', 'enir')}

  Just for clarity:
  There are also the following similarity classes shared between Alorese and LH.
  {('wind', 'lama1277-botun', 'aŋi'), ('wind', 'lama1277-lamat', 'aŋi'), ('wind', 'lama1277-lewoe', 'aŋin'), ('wind', 'lama1277-adona', 'ˈʔaŋĩ'), ('wind', 'lama1277-waiwa', 'aŋi'), ('wind', 'sika1262-tanai', 'ani-n'), ('wind', 'lama1277-belan', 'aŋi'), ('wind', 'sika1262-hewa', 'a̤nin'), ('wind', 'sika1262-hewa', 'anin'), ('wind', 'tetu1246', 'ʔɑnin'), ('wind', 'p-mala1545-abvd', '*haŋin'), ('wind', 'lama1277-tanju', 'aŋi'), ('wind', 'lama1277-kalik', 'aŋin'), ('wind', 'lama1277-lamah', 'aŋi'), ('wind', 'lama1277-dulhi', 'aŋi'), ('wind', 'alor1247-munas', 'aŋiŋ'), ('wind', 'p-cent2245-abvd', '*haŋin'), ('wind', 'sika1262-maume', 'anin'), ('wind', 'lama1277-kalik', 'aˈŋin'), ('wind', 'lama1277-lamak', 'aŋi'), ('wind', 'lama1277-lewom', 'aŋi'), ('wind', 'tetu1245-suai', 'aniŋ'), ('wind', 'lama1277-ritae', 'aŋi'), ('wind', 'lama1277-waiba', 'aŋi'), ('wind', 'lama1277-mulan', 'əŋi'), ('wind', 'lama1277-lamal', 'aŋi'), ('wind', 'lama1277-lewop', 'aŋin'), ('wind', 'lama1277-horow', 'aŋi'), ('wind', 'keda1252-leuwa', 'a̘ŋin vεŋin'), ('wind', 'alor1247-baran', 'aŋi'), ('wind', 'lama1277-watan', 'aŋi'), ('wind', 'lama1277-wuake', 'aŋi'), ('wind', 'lama1277-paina', 'aŋin'), ('wind', 'koto1251', 'anin'), ('wind', 'lama1277-merde', 'aŋi'), ('wind', 'lama1277-bama', 'aŋi'), ('wind', 'alor1247-besar', 'aŋĩ'), ('wind', 'keda1252', 'aŋin'), ('wind', 'lama1277-lewog', 'aŋi'), ('wind', 'lama1277-minga', 'aŋi'), ('wind', 'lama1277-ileap', 'aŋin'), ('wind', 'lama1277-imulo', 'aŋi'), ('wind', 'lama1277-kiwan', 'aŋi'), ('wind', 'lama1277-lerek', 'ˈaŋin'), ('wind', 'keda1252-leuba', "aː'ŋin"), ('wind', 'lama1277-lewok', 'aŋinu'), ('wind', 'lama1277-lewuk', 'aŋin'), ('wind', 'lama1277-kalik', 'aˈŋi'), ('wind', 'lama1277-lewob', 'aŋi'), ('wind', 'alor1247-pandai', 'aŋiŋ'), ('wind', 'lama1277-lewoi', 'ˈaŋin'), ('wind', 'kema1243', 'ʔɑgi'), ('wind', 'tetu1245-vique', 'aniŋ'), ('wind', 'lama1277-lewot', 'aŋinu'), ('wind', 'nede1245', 'aŋi'), ('wind', 'p-mala1545-acd', '*haŋin'), ('wind', 'lama1277-baipi', 'aŋi')}


Concept ‘straight’ is different between Alorese and Lamaholot.

 One or more Lamaholot dialects innovated forms not found in Alorese:
 {('straight', 'lama1277-horow', 'kəloho'), ('straight', 'lama1277-botun', 'kəlohok'), ('straight', 'lama1277-waiwa', 'kəloho'), ('straight', 'lama1277-watan', 'kəloho'), ('straight', 'lama1277-tanju', 'kəlo'), ('straight', 'lama1277-adona', 'kɜˈlɔhɔ'), ('straight', 'lama1277-kiwan', 'kəlohʔã'), ('straight', 'lama1277-lewob', 'kəlodo'), ('straight', 'lama1277-dulhi', 'kəloho'), ('straight', 'laka1255', 'klɔ:n'), ('straight', 'lama1277-lamak', 'kəloho')}
 {('straight', 'lama1277-bama', 'mopa'), ('straight', 'lama1277-baipi', 'mopa'), ('straight', 'lama1277-lewoi', 'ˈmopaʔ'), ('straight', 'lama1277-ritae', 'mopã'), ('straight', 'lama1277-waiba', 'mopa'), ('straight', 'lama1277-lewoe', 'mohpa'), ('straight', 'lama1277-lewog', 'mopaʔ'), ('straight', 'lama1277-lewom', 'mopa')}
 {('straight', 'p-mala1545-acd', '*lurus'), ('straight', 'lama1277-lewuk', 'lurus'), ('straight', 'kula1280-lanto', '’lura'), ('straight', 'wers1238-marit', 'lɔr'), ('straight', 'kula1280-lanto', 'lura'), ('straight', 'lama1277-pukau', 'lorʔã'), ('straight', 'sawi1256', 'lura'), ('straight', 'wers1238-taram', 'lor'), ('straight', 'tuku1254', 'lɔba')}

  Just for clarity:
  There are also the following similarity classes shared between Alorese and LH.
  {('straight', 'kaer1234', "u'quruŋ"), ('straight', 'abui1241-fuime', 'mulaŋ'), ('straight', 'lama1277-ileap', 'mūr'), ('straight', 'alor1247-pandai', 'moloŋ'), ('straight', 'lama1277-imulo', 'mulurən'), ('straight', 'lama1277-mulan', 'mulu'), ('straight', 'blag1240-bama', 'molos'), ('straight', 'lama1277-minga', 'mulur'), ('straight', 'lama1277-belan', 'mulu'), ('straight', 'lama1277-paina', 'mulurən'), ('straight', 'blag1240-warsa', 'omolo'), ('straight', 'pura1258', 'molo'), ('straight', 'lama1277-lamat', 'mulũlũ'), ('straight', 'atim1239', 'mulaŋ'), ('straight', 'lama1277-lamal', 'mūlu'), ('straight', 'rett1240', 'mɔlɔ'), ('straight', 'adan1251-otvai', '’mulaŋ'), ('straight', 'lama1277-lewot', 'mulu'), ('straight', 'lama1277-merde', 'murã'), ('straight', 'lama1277-lamah', 'murã'), ('straight', 'abui1241-takal', "mu.'läŋ"), ('straight', 'lama1277-kalik', 'mulur'), ('straight', 'alor1247-besar', 'molo'), ('straight', 'blag1240-nule', 'moloŋ'), ('straight', 'lama1277-lerek', 'muluˈrən'), ('straight', 'lama1277-lewok', 'mulur'), ('straight', 'lama1277-kalik', 'moˈlur'), ('straight', 'lama1277-wuake', 'mulu'), ('straight', 'rett1240', 'molo'), ('straight', 'baka1276', 'moloŋ'), ('straight', 'alor1247-baran', 'mallɔŋ'), ('straight', 'lama1277-lewop', 'mulurən'), ('straight', 'blag1240-tuntu', "mo'lus"), ('straight', 'keda1252', 'potoŋ'), ('straight', 'alor1247-munas', 'mɔlɔŋ')}


Concept ‘to_accuse’ is different between Alorese and Lamaholot.

 Alorese innovated the following forms:
 {('to_accuse', 'alor1247-pandai', "turuŋ 'tumaŋ"), ('to_accuse', 'alor1247-besar', 'turuŋ'), ('to_accuse', 'alor1247-munas', "turuŋ 'apa")}
 One or more Lamaholot dialects innovated forms not found in Alorese:
 {('to_accuse', 'lama1277-lerek', 'ˈmanaŋ')}
 {('to_accuse', 'lama1277-adona', 'ʔɜˈwaʔ'), ('to_accuse', 'lama1277-kalik', 'əˈvan')}
 {('to_accuse', 'lama1277-lewoi', 'pəˈlaiŋ')}


Concept ‘to_chew’ is different between Alorese and Lamaholot.

 One or more Lamaholot dialects innovated forms not found in Alorese:
 {('to_chew', 'lama1277-lerek', 'maˈhap')}
 {('to_chew', 'lama1277-lewoi', 'ˈbaha'), ('to_chew', 'lama1277-adona', 'ˈbɑhɑk')}

  Just for clarity:
  There are also the following similarity classes shared between Alorese and LH.
  {('to_chew', 'alor1247-pandai', 'mame'), ('to_chew', 'alor1247-besar', 'mamɛ'), ('to_chew', 'sika1262-hewa', 'maʔmaʔ'), ('to_chew', 'blag1240-nule', 'mame'), ('to_chew', 'alor1247-munas', 'mamɛ'), ('to_chew', 'lama1277-kalik', 'maˈmɔt'), ('to_chew', 'koto1251', 'n-mama'), ('to_chew', 'p-mala1545-abvd', '*mamaq'), ('to_chew', 'baka1276', 'mame'), ('to_chew', 'keda1252-leuba', "ma'mɛʔ"), ('to_chew', 'blag1240-kulij', 'mameʔ'), ('to_chew', 'p-cent2245-abvd', '*mamaq')}


Concept ‘to_fall_over’ is different between Alorese and Lamaholot.

 Alorese shares the following forms with TAP languages:
 {('to_fall_over', 'lamm1241-westp', 'bauluŋ'), ('to_fall_over', 'alor1247-pandai', 'bakuŋ')}
 One or more Lamaholot dialects innovated forms not found in Alorese:
 {('to_fall_over', 'lama1277-kalik', 'ɡoˈkal')}

  Just for clarity:
  There are also the following similarity classes shared between Alorese and LH.
  {('to_fall_over', 'alor1247-pandai', 'hɔbak'), ('to_fall_over', 'lama1277-adona', 'ˈtobaŋ'), ('to_fall_over', 'lama1277-adona', 'ˈhobat'), ('to_fall_over', 'alor1247-besar', 'hoba'), ('to_fall_over', 'alor1247-munas', 'hobak'), ('to_fall_over', 'lama1277-lerek', 'ˈtobaŋ'), ('to_fall_over', 'lama1277-kalik', 'tuˈbal'), ('to_fall_over', 'lama1277-lewoi', 'ˈtɔbɑŋ'), ('to_fall_over', 'keda1252-leuba', 'hubaʔ')}


Concept ‘firewood’ is different between Alorese and Lamaholot.

 One or more Lamaholot dialects innovated forms not found in Alorese:
 {('firewood', 'lama1277-kalik', 'ˈkaʤɔr ta doˈsɔm')}

  Just for clarity:
  There are also the following similarity classes shared between Alorese and LH.
  {('firewood', 'lama1277-lewoi', 'kajo ˈape'), ('firewood', 'alor1247-pandai', "kaʤɔ 'ape"), ('firewood', 'alor1247-besar', "kaʤɔ 'ape"), ('firewood', 'keda1252-leuba', "ʔai 'japi"), ('firewood', 'lama1277-lerek', 'kaj ˈapi'), ('firewood', 'alor1247-munas', "kaʤɔ 'ape"), ('firewood', 'lama1277-adona', 'kajo ˈʔape')}


Concept ‘sand’ is different between Alorese and Lamaholot.

 Alorese innovated the following forms:
 {('sand', 'alor1247-munas', "tə'nːi"), ('sand', 'alor1247-besar', "ta'nːiŋ"), ('sand', 'alor1247-munas', "ə'nːi")}
 Alorese shares the following forms with other AN languanges, but not Lamaholot:
 {('sand', 'keda1252', 'e̤ne'), ('sand', 'sika1262-tanai', 'ne'), ('sand', 'alor1247-munas', "a'nːe"), ('sand', 'sika1262-hewa', 'nen'), ('sand', 'keda1252-leuwa', 'ε̘nε'), ('sand', 'sika1262-maume', 'ne'), ('sand', 'alor1247-pandai', 'ane'), ('sand', 'alor1247-pandai', 'anːe')}
 One or more Lamaholot dialects innovated forms not found in Alorese:
 {('sand', 'lama1277-lewuk', 'botan'), ('sand', 'p-aust1307-abvd', '*bunaj'), ('sand', 'lama1277-lewop', 'bota'), ('sand', 'sika1262-hewa', 'əda'), ('sand', 'lama1277-kalik', 'botan'), ('sand', 'kabo1247', 'woitʲo'), ('sand', 'lama1277-lamal', 'botã'), ('sand', 'lama1277-mulan', 'botɤ'), ('sand', 'lama1277-kalik', 'ˈbota'), ('sand', 'lama1277-kalik', 'ˈbotan'), ('sand', 'lama1277-minga', 'bota'), ('sand', 'lama1277-lewoe', 'botã'), ('sand', 'lama1277-paina', 'botan'), ('sand', 'keda1252-leuwa', 'buta'), ('sand', 'lama1277-lamat', 'botã'), ('sand', 'sika1262-hewa', 'ʔəda'), ('sand', 'lama1277-lerek', 'ˈbotan')}
 {('sand', 'lama1277-baipi', 'wəra'), ('sand', 'lama1277-lewok', 'wəraru'), ('sand', 'lama1277-botun', 'wəra'), ('sand', 'lama1277-lamah', 'wəra'), ('sand', 'lama1277-imulo', 'fərar'), ('sand', 'lama1277-belan', 'wəra'), ('sand', 'lama1277-tanju', 'wəra'), ('sand', 'lama1277-lewob', 'wəra'), ('sand', 'lama1277-pukau', 'wəra'), ('sand', 'lama1277-merde', 'wəra'), ('sand', 'lama1277-wuake', 'wəra'), ('sand', 'lama1277-lewoi', 'ˈwəra'), ('sand', 'lama1277-lewot', 'fəraja'), ('sand', 'lama1277-bama', 'wəra'), ('sand', 'sika1262-tanai', 'ne βura'), ('sand', 'lama1277-kiwan', 'wəra'), ('sand', 'lama1277-lamak', 'wəra'), ('sand', 'lama1277-lewog', 'wəra'), ('sand', 'lama1277-dulhi', 'wəra'), ('sand', 'lama1277-adona', 'ˈwɜra'), ('sand', 'lama1277-lewom', 'wəra'), ('sand', 'lama1277-ritae', 'wəra'), ('sand', 'lama1277-watan', 'wəra'), ('sand', 'lama1277-waiba', 'wəra'), ('sand', 'lama1277-ileap', 'wəra'), ('sand', 'lama1277-waiwa', 'wəra'), ('sand', 'lama1277-horow', 'wəra')}


Concept ‘nephew_niece’ is different between Alorese and Lamaholot.

 Alorese innovated the following forms:
 {('nephew_niece', 'alor1247-pandai', 'nula'), ('nephew_niece', 'alor1247-pandai', "m'nulaŋ"), ('nephew_niece', 'alor1247-besar', "ma'nulaŋ"), ('nephew_niece', 'alor1247-munas', 'nulaʔ'), ('nephew_niece', 'alor1247-munas', "mə'nulaŋ")}
 One or more Lamaholot dialects innovated forms not found in Alorese:
 {('nephew_niece', 'keda1252-leuba', 'anaʔ'), ('nephew_niece', 'lama1277-lewoi', 'anaʔ'), ('nephew_niece', 'lama1277-adona', 'ʔanaʔ')}


Concept ‘if’ is different between Alorese and Lamaholot.

 Alorese innovated the following forms:
 {('if', 'alor1247-munas', "nəm kə'te kə'ne")}
 One or more Lamaholot dialects innovated forms not found in Alorese:
 {('if', 'lama1277-kalik', 'ma'), ('if', 'lama1277-lerek', 'məˈki'), ('if', 'p-cent2245-abvd', '*ma'), ('if', 'abui1241-ulaga', 'mei'), ('if', 'lama1277-adona', 'ˈmia'), ('if', 'abui1241-takal', 'mɞɪ')}

  Just for clarity:
  There are also the following similarity classes shared between Alorese and LH.
  {('if', 'teiw1235', 'kalau'), ('if', 'p-aust1307-abvd', '*ka'), ('if', 'lama1277-lamak', 'kalu'), ('if', 'lama1277-waiwa', 'kalu'), ('if', 'lama1277-imulo', 'kalu'), ('if', 'lama1277-lewot', 'kalu'), ('if', 'p-mala1545-abvd', '*ka'), ('if', 'lama1277-lewop', 'kalu'), ('if', 'lama1277-mulan', 'kalu'), ('if', 'lama1277-waiba', 'kalu'), ('if', 'lama1277-lewob', 'kalu'), ('if', 'lama1277-lamal', 'kalu'), ('if', 'lama1277-ritae', 'kalu'), ('if', 'lama1277-lewog', 'kalu'), ('if', 'alor1247-baran', 'kalau'), ('if', 'lama1277-kiwan', 'kalu'), ('if', 'alor1247-besar', 'kalɔ'), ('if', 'lama1277-bama', 'kalu'), ('if', 'lama1277-minga', 'kalu'), ('if', 'lama1277-kalik', 'kalu ma'), ('if', 'lamm1241-westp', 'kalo'), ('if', 'lama1277-lamah', 'kalu'), ('if', 'p-mala1545-acd', '*ka'), ('if', 'lama1277-lewoi', 'ˈkalɔ'), ('if', 'lama1277-wuake', 'kalu'), ('if', 'lama1277-lamat', 'kalu'), ('if', 'lama1277-lewuk', 'kalu ma'), ('if', 'lama1277-watan', 'kalu'), ('if', 'lama1277-pukau', 'kalu'), ('if', 'lama1277-baipi', 'kalu'), ('if', 'lama1277-tanju', 'kalu'), ('if', 'keda1252-leuba', "ka'lau"), ('if', 'lama1277-merde', 'kalu'), ('if', 'lama1277-belan', 'kalu'), ('if', 'idat1237', 'kalɑu'), ('if', 'rett1240', 'ka:lu'), ('if', 'alor1247-pandai', 'kalɔ'), ('if', 'lama1277-lewom', 'kalu')}


Concept ‘sharp’ is different between Alorese and Lamaholot.

 One or more Lamaholot dialects innovated forms not found in Alorese:
 {('sharp', 'lama1277-lamat', 'raʔe'), ('sharp', 'lama1277-lewoe', 'reʔõ')}
 {('sharp', 'lama1277-pukau', 'gã'), ('sharp', 'lama1277-lewob', 'gã')}
 {('sharp', 'lama1277-dulhi', 'wəreʔe')}
 {('sharp', 'abui1241-fuime', 'bula'), ('sharp', 'abui1241-takal', "bu.'lä"), ('sharp', 'kafo1240', 'bulɑ'), ('sharp', 'kama1365', 'piːla'), ('sharp', 'lama1277-adona', 'bɜˈleu'), ('sharp', 'abui1241-ulaga', 'bula'), ('sharp', 'atim1239', 'bula')}

  Just for clarity:
  There are also the following similarity classes shared between Alorese and LH.
  {('sharp', 'lama1277-watan', 'dəkət'), ('sharp', 'lama1277-merde', 'bərəkət'), ('sharp', 'lama1277-horow', 'bərəkət'), ('sharp', 'lama1277-lamak', 'bərəkət'), ('sharp', 'lama1277-lerek', 'rəkəˈtən'), ('sharp', 'lama1277-lewuk', 'bərəkət'), ('sharp', 'lama1277-kalik', 'rəˈkət'), ('sharp', 'lama1277-kiwan', 'dəkət'), ('sharp', 'lama1277-lewoi', 'ˈbərəkə'), ('sharp', 'lama1277-tanju', 'dəkət'), ('sharp', 'lama1277-waiwa', 'bərəkət'), ('sharp', 'alor1247-munas', "də'kɛk"), ('sharp', 'lama1277-botun', 'dəkət'), ('sharp', 'lama1277-waiba', 'dəkət'), ('sharp', 'lama1277-belan', 'rəkət'), ('sharp', 'lama1277-mulan', 'rəkə'), ('sharp', 'lama1277-lewog', 'bərekə'), ('sharp', 'lama1277-imulo', 'bərək'), ('sharp', 'alor1247-besar', 'dakːɛ'), ('sharp', 'alor1247-baran', 'dak:ɛ'), ('sharp', 'alor1247-munas', "də'kɛ 'tɔrak"), ('sharp', 'lama1277-ileap', 'dəkət'), ('sharp', 'lama1277-lewok', 'rəkət'), ('sharp', 'alor1247-pandai', "də'kɛk"), ('sharp', 'lama1277-adona', 'ˈdɜkɜt'), ('sharp', 'lama1277-lamah', 'rəkətə'), ('sharp', 'lama1277-bama', 'bərəkət'), ('sharp', 'lama1277-minga', 'rəkət'), ('sharp', 'lama1277-lewom', 'dəkət'), ('sharp', 'lama1277-ritae', 'dəkət'), ('sharp', 'lama1277-wuake', 'rəket'), ('sharp', 'lama1277-paina', 'rəkət'), ('sharp', 'lama1277-lewot', 'bərəkət'), ('sharp', 'lama1277-kalik', 'rəkət'), ('sharp', 'lama1277-lewop', 'bərəkət'), ('sharp', 'lama1277-baipi', 'dəkət'), ('sharp', 'lama1277-lamal', 'bərəkət')}


Concept ‘easy’ is different between Alorese and Lamaholot.

 Alorese innovated the following forms:
 {('easy', 'alor1247-munas', "pə'duk")}
 {('easy', 'alor1247-pandai', "sə'sadʒa"), ('easy', 'alor1247-munas', "sɛ'sadʒa")}
 {('easy', 'alor1247-besar', 'lohi')}
 One or more Lamaholot dialects innovated forms not found in Alorese:
 {('easy', 'lama1277-kalik', 'klɔˈsʊm')}
 {('easy', 'lama1277-adona', 'ˈpəlelɛt')}
 {('easy', 'teiw1235', 'gampaŋ'), ('easy', 'lama1277-lewoi', 'ˈɡɑmpɑŋ'), ('easy', 'keda1252-leuba', 'ɡampaŋ')}


Concept ‘neck’ is different between Alorese and Lamaholot.

 Alorese shares the following forms with other AN languanges, but not Lamaholot:
 {('neck', 'idat1237', "kakɔ'rɔ:k"), ('neck', 'tetu1245-vique', "ka'kɔrɔk"), ('neck', 'tetu1245-suai', "ka'kɔrɔk"), ('neck', 'tetu1246', "kɑkɔ'rɔk"), ('neck', 'alor1247-munas', "k'bukɔr")}

  Just for clarity:
  There are also the following similarity classes shared between Alorese and LH.
  {('neck', 'lama1277-lewom', 'wuli'), ('neck', 'lama1277-kalik', 'wulen'), ('neck', 'lama1277-lamak', 'wulĩ'), ('neck', 'lama1277-merde', 'wulik'), ('neck', 'alor1247-pandai', 'wuliŋ'), ('neck', 'lama1277-belan', 'wulik'), ('neck', 'lama1277-botun', 'wuli'), ('neck', 'lama1277-imulo', 'fulesa'), ('neck', 'lama1277-lewoi', 'ˈwuliŋ'), ('neck', 'keda1252-leuwa', 'u̘li'), ('neck', 'lama1277-horow', 'wulĩ'), ('neck', 'lama1277-lamal', 'fuli'), ('neck', 'atim1239', 'rola'), ('neck', 'lama1277-waiba', 'wulik'), ('neck', 'lama1277-mulan', 'fuli'), ('neck', 'alor1247-baran', 'fuliŋ'), ('neck', 'lama1277-ritae', 'wulĩ'), ('neck', 'alor1247-besar', 'fuliŋ'), ('neck', 'lama1277-kalik', 'vuˈle'), ('neck', 'lama1277-lamat', 'wuli'), ('neck', 'lama1277-waiwa', 'wuli'), ('neck', 'lama1277-lewog', 'wulĩ'), ('neck', 'lama1277-lewuk', 'wule'), ('neck', 'lama1277-lewok', 'wule'), ('neck', 'lama1277-watan', 'wulĩ'), ('neck', 'lama1277-lewot', 'fuleha'), ('neck', 'lama1277-kiwan', 'wulik'), ('neck', 'lama1277-tanju', 'wuli'), ('neck', 'lama1277-paina', 'wuleg'), ('neck', 'lama1277-lerek', 'ˈwule'), ('neck', 'lama1277-baipi', 'wuli'), ('neck', 'alor1247-munas', 'wuliŋ'), ('neck', 'lama1277-lewop', 'fule'), ('neck', 'lama1277-lewoe', 'wull̃'), ('neck', 'lama1277-ileap', 'wuli'), ('neck', 'lama1277-lewob', 'wuli'), ('neck', 'lama1277-adona', 'ˈwuliˈkɜt'), ('neck', 'lama1277-lamah', 'wulikə'), ('neck', 'lama1277-dulhi', 'wuliŋ'), ('neck', 'lama1277-wuake', 'wuli'), ('neck', 'lama1277-pukau', 'wulĩ'), ('neck', 'lama1277-minga', 'fule'), ('neck', 'lama1277-bama', 'wuli'), ('neck', 'kuii1253', 'wot plok')}


Concept ‘sister’ is different between Alorese and Lamaholot.

 One or more Lamaholot dialects innovated forms not found in Alorese:
 {('sister', 'lama1277-lewoi', 'aʔe'), ('sister', 'keda1252-leuba', 'aʔe')}
 {('sister', 'keda1252-leuba', 'ariʔ'), ('sister', 'lama1277-lewoi', 'ariʔ (arian)')}

  Just for clarity:
  There are also the following similarity classes shared between Alorese and LH.
  {('sister', 'alor1247-munas', 'binɛŋ'), ('sister', 'alor1247-pandai', 'binɛŋ '), ('sister', 'lama1277-adona', 'bine-k'), ('sister', 'alor1247-munas', 'binɛʔ'), ('sister', 'alor1247-besar', "binɛŋ ka'fae")}


Concept ‘tomorrow’ is different between Alorese and Lamaholot.

 Alorese innovated the following forms:
 {('tomorrow', 'alor1247-munas', 'bouʔ '), ('tomorrow', 'alor1247-besar', 'bou')}
 One or more Lamaholot dialects innovated forms not found in Alorese:
 {('tomorrow', 'lama1277-kalik', 'bamˈbe')}
 {('tomorrow', 'blag1240-warsa', 'tobaŋ'), ('tomorrow', 'rett1240', 'piŋ'), ('tomorrow', 'lama1277-lerek', 'baˈen'), ('tomorrow', 'rett1240', 'pe:ŋ'), ('tomorrow', 'kama1365', 'paisan'), ('tomorrow', 'blag1240-kulij', 'tobaŋ'), ('tomorrow', 'dein1238', 'bas:aŋ'), ('tomorrow', 'blag1240-bama', 'tobaŋ'), ('tomorrow', 'blag1240-tuntu', 'tobaŋ'), ('tomorrow', 'baka1276', 'tobaŋ'), ('tomorrow', 'blag1240-nule', 'tobaŋ')}

  Just for clarity:
  There are also the following similarity classes shared between Alorese and LH.
  {('tomorrow', 'sarr1247-nule', 'bat'), ('tomorrow', 'lama1277-adona', 'ˈbauk'), ('tomorrow', 'alor1247-pandai', 'bau'), ('tomorrow', 'teiw1235', 'bas'), ('tomorrow', 'lama1277-lewoi', 'ˈbəta'), ('tomorrow', 'sarr1247-adiab', 'bat')}


Concept ‘sweet’ is different between Alorese and Lamaholot.

 Alorese shares the following forms with TAP languages:
 {('sweet', 'alor1247-pandai', "ta'gːɛ"), ('sweet', 'alor1247-pandai', "taŋ'gɛ"), ('sweet', 'alor1247-munas', "tə'gɛ"), ('sweet', 'blag1240-kulij', 'teŋge'), ('sweet', 'blag1240-nule', 'teŋge'), ('sweet', 'baka1276', 'teŋge'), ('sweet', 'alor1247-besar', 'taŋge')}
 One or more Lamaholot dialects innovated forms not found in Alorese:
 {('sweet', 'lama1277-lewoi', 'ˈhərə'), ('sweet', 'sika1262-hewa', 'hureʔ')}
 {('sweet', 'lama1277-lewoi', 'ˈmai')}
 {('sweet', 'lama1277-lerek', 'hər-hər')}
 {('sweet', 'lama1277-adona', 'kɜˈseʔe')}
 {('sweet', 'lama1277-lerek', 'hənəruˈkən'), ('sweet', 'lama1277-kalik', 'snəˈruk')}


Concept ‘finished’ is different between Alorese and Lamaholot.

 Alorese innovated the following forms:
 {('finished', 'alor1247-besar', "amuŋ 'neka ")}
 {('finished', 'alor1247-munas', 'hapuʔ')}
 Alorese shares the following forms with other AN languanges, but not Lamaholot:
 {('finished', 'p-mala1545-acd', '*qubus'), ('finished', 'p-mala1545-acd', '*upus'), ('finished', 'alor1247-pandai', "ka'pːo")}
 One or more Lamaholot dialects innovated forms not found in Alorese:
 {('finished', 'lama1277-kalik', 'vaka'), ('finished', 'lama1277-adona', 'ˈwahak(a)'), ('finished', 'lama1277-kalik', 'vak'), ('finished', 'lama1277-lewoi', 'ˈwaha')}
 {('finished', 'lama1277-lerek', 'ˈipok')}


Concept ‘to_wipe’ is different between Alorese and Lamaholot.

 One or more Lamaholot dialects innovated forms not found in Alorese:
 {('to_wipe', 'lama1277-belan', 'bəjak')}
 {('to_wipe', 'keda1252-leuwa', 'pɔhɔʔ'), ('to_wipe', 'p-mala1545-acd', '*usuq'), ('to_wipe', 'lama1277-botun', 'bohok'), ('to_wipe', 'lama1277-kalik', 'sɔk'), ('to_wipe', 'keda1252', 'poho'), ('to_wipe', 'lama1277-lerek', 'pohoˈkəŋ'), ('to_wipe', 'keda1252-leuba', "pɔ'hɔʔ"), ('to_wipe', 'lama1277-watan', 'oso'), ('to_wipe', 'lama1277-lamak', 'oso'), ('to_wipe', 'lama1277-lewop', 'hok'), ('to_wipe', 'lama1277-ileap', 'boho'), ('to_wipe', 'sika1262-tanai', 'bloso'), ('to_wipe', 'lama1277-horow', 'bohok'), ('to_wipe', 'lama1277-kiwan', 'bohok'), ('to_wipe', 'lama1277-kalik', 'soka'), ('to_wipe', 'lama1277-ritae', 'goho'), ('to_wipe', 'lama1277-merde', 'tohok'), ('to_wipe', 'lama1277-paina', 'pohok'), ('to_wipe', 'lama1277-adona', 'ˈbɔhɔʔ'), ('to_wipe', 'lama1277-dulhi', 'bohok')}
 {('to_wipe', 'buna1278-suai', 'gamɔs'), ('to_wipe', 'lama1277-waiba', 'hamu'), ('to_wipe', 'lama1277-lewob', 'hamu'), ('to_wipe', 'lama1277-lewom', 'hamu'), ('to_wipe', 'buna1278-bobon', 'gamɔs'), ('to_wipe', 'tetu1245-vique', "ha'moɁos"), ('to_wipe', 'tetu1245-suai', "ha'moɁos"), ('to_wipe', 'lama1277-tanju', 'hamu'), ('to_wipe', 'lama1277-bama', 'hamu'), ('to_wipe', 'lama1277-lewog', 'hamu'), ('to_wipe', 'lama1277-waiwa', 'hamo')}
 {('to_wipe', 'lama1277-lewoi', 'tɛˈruʔ'), ('to_wipe', 'rett1240', 'doro'), ('to_wipe', 'rett1240', 'dɔrɔ'), ('to_wipe', 'keda1252-leuwa', 'dɔru')}

  Just for clarity:
  There are also the following similarity classes shared between Alorese and LH.
  {('to_wipe', 'lama1277-lewok', 'apus'), ('to_wipe', 'lama1277-pukau', 'hapũ'), ('to_wipe', 'rett1240', 'hapɔ'), ('to_wipe', 'lama1277-lamat', 'hapu'), ('to_wipe', 'lama1277-lewoe', 'hapu'), ('to_wipe', 'baka1276', 'hapo'), ('to_wipe', 'alor1247-munas', 'hapɔ'), ('to_wipe', 'lama1277-lewot', 'apuja'), ('to_wipe', 'lama1277-imulo', 'hapus'), ('to_wipe', 'lama1277-lamah', 'hapu'), ('to_wipe', 'idat1237', 'sɑpu'), ('to_wipe', 'blag1240-nule', 'hapo'), ('to_wipe', 'alor1247-pandai', 'hapo'), ('to_wipe', 'p-mala1545-acd', '*punas'), ('to_wipe', 'lama1277-lamal', 'hapu'), ('to_wipe', 'lama1277-lewuk', 'apus'), ('to_wipe', 'alor1247-besar', 'hapɔ'), ('to_wipe', 'lama1277-wuake', 'hapus'), ('to_wipe', 'lama1277-mulan', 'hapu'), ('to_wipe', 'p-mala1545-acd', '*sapu'), ('to_wipe', 'alor1247-baran', 'hapɔ'), ('to_wipe', 'blag1240-kulij', 'hapo'), ('to_wipe', 'sika1262-hewa', 'hapus'), ('to_wipe', 'lama1277-minga', 'apujɤ')}
  {('to_wipe', 'buna1278-malia', 'dɑsɑh'), ('to_wipe', 'lama1277-baipi', 'teʔu'), ('to_wipe', 'mamb1306', 'das'), ('to_wipe', 'kema1243', 'ra:s'), ('to_wipe', 'laka1255', 'dasa'), ('to_wipe', 'tetu1246', 'dɑsa'), ('to_wipe', 'alor1247-pandai', 'dɔhɔ')}


Concept ‘lungs’ is different between Alorese and Lamaholot.

 One or more Lamaholot dialects innovated forms not found in Alorese:
 {('lungs', 'lama1277-lewoi', 'ˈparu-ˈparu')}

  Just for clarity:
  There are also the following similarity classes shared between Alorese and LH.
  {('lungs', 'blag1240-kulij', 'buraŋ'), ('lungs', 'lama1277-lerek', 'ˈore wuˈdʒ͡an'), ('lungs', 'blag1240-nule', "bu'raŋ"), ('lungs', 'alor1247-munas', 'wuraŋ'), ('lungs', 'blag1240-warsa', 'guburaŋ'), ('lungs', 'baka1276', 'buraŋ'), ('lungs', 'alor1247-besar', 'furaŋ'), ('lungs', 'lama1277-kalik', 'vuˈʤa'), ('lungs', 'lama1277-adona', 'ˈwurhã'), ('lungs', 'alor1247-pandai', 'wuraŋ')}


Concept ‘to_yawn’ is different between Alorese and Lamaholot.

 Alorese innovated the following forms:
 {('to_yawn', 'alor1247-munas', "p'noek"), ('to_yawn', 'alor1247-besar', 'panoe'), ('to_yawn', 'alor1247-pandai', "p'noe")}
 One or more Lamaholot dialects innovated forms not found in Alorese:
 {('to_yawn', 'lama1277-adona', 'pɜˈmoaː'), ('to_yawn', 'keda1252-leuba', 'moa'), ('to_yawn', 'lama1277-lewoi', 'pəˈmoa')}
 {('to_yawn', 'lama1277-kalik', 'kəmuˈav'), ('to_yawn', 'lama1277-lerek', 'kəˈmoav'), ('to_yawn', 'kira1248', 'agup')}


Concept ‘to_hide’ is different between Alorese and Lamaholot.

 Alorese innovated the following forms:
 {('to_hide', 'alor1247-pandai', 'dawu'), ('to_hide', 'alor1247-besar', 'dafu'), ('to_hide', 'alor1247-munas', "də'wuk")}
 One or more Lamaholot dialects innovated forms not found in Alorese:
 {('to_hide', 'lama1277-adona', 'ˈhorõ'), ('to_hide', 'lama1277-kalik', 'soˈrɔ'), ('to_hide', 'lama1277-lerek', 'hoˈroŋ'), ('to_hide', 'lama1277-lewoi', 'ˈhɔrɔŋ')}


Concept ‘deaf’ is different between Alorese and Lamaholot.

 Alorese shares the following forms with TAP languages:
 {('deaf', 'alor1247-munas', 'mukɛk'), ('deaf', 'alor1247-besar', "ka'mukɛ"), ('deaf', 'kula1280-lanto', 'moki'), ('deaf', 'blag1240-nule', 'kəmuke'), ('deaf', 'blag1240-nule', 'kmuke'), ('deaf', 'alor1247-pandai', 'muke'), ('deaf', 'alor1247-baran', "ka'muk:ɛ"), ('deaf', 'blag1240-kulij', 'klus'), ('deaf', 'alor1247-pandai', "k'muke"), ('deaf', 'sawi1256', 'muko')}
 One or more Lamaholot dialects innovated forms not found in Alorese:
 {('deaf', 'lama1277-lewoi', 'kəˈbekɛŋ'), ('deaf', 'lama1277-adona', 'kɜˈbekʔẽ')}
 {('deaf', 'lama1277-lerek', 'kəˈlebitən'), ('deaf', 'lama1277-kalik', 'kleˈbit')}


Concept ‘to_cry’ is different between Alorese and Lamaholot.

 One or more Lamaholot dialects innovated forms not found in Alorese:
 {('to_cry', 'lama1277-lewob', 'gaka')}
 {('to_cry', 'idat1237', 'sɛro'), ('to_cry', 'tuku1254', 'se:ɔ'), ('to_cry', 'lama1277-imulo', 'əra'), ('to_cry', 'lama1277-lewot', 'əra'), ('to_cry', 'lama1277-kalik', 'əˈra'), ('to_cry', 'kema1243', 'siɔ'), ('to_cry', 'lama1277-kalik', 'əraw'), ('to_cry', 'maka1316', 'iara'), ('to_cry', 'lama1277-lewuk', 'əra'), ('to_cry', 'mamb1306', 'sɛro'), ('to_cry', 'laka1255', 'sɛrɔ')}

  Just for clarity:
  There are also the following similarity classes shared between Alorese and LH.
  {('to_cry', 'kafo1240', 'kɑriɑ'), ('to_cry', 'kuii1253', 'bir'), ('to_cry', 'lama1277-lewop', 'giləŋ'), ('to_cry', 'sawi1256', 'kiri'), ('to_cry', 'lama1277-paina', 'giar'), ('to_cry', 'alor1247-besar', 'garɛŋ'), ('to_cry', 'lama1277-minga', 'gilɤ'), ('to_cry', 'lama1277-lewok', 'giar'), ('to_cry', 'nede1245', 'kiaru'), ('to_cry', 'lama1277-lerek', 'ˈɡiar')}
  {('to_cry', 'alor1247-pandai', 'taniŋ'), ('to_cry', 'p-mala1545-acd', '*ma-naŋis'), ('to_cry', 'lama1277-watan', 'tani'), ('to_cry', 'lama1277-lamak', 'tani'), ('to_cry', 'tetu1245-vique', 'tanɪs'), ('to_cry', 'p-cent2245-abvd', '*taŋis'), ('to_cry', 'sika1262-maume', 't̪ani'), ('to_cry', 'alor1247-baran', 'tanĩ'), ('to_cry', 'sika1262-hewa', 'dani'), ('to_cry', 'lama1277-lewog', 'tani'), ('to_cry', 'tetu1246', 'tɑnis'), ('to_cry', 'p-aust1307-abvd', '*Caŋis'), ('to_cry', 'lama1277-bama', 'tani'), ('to_cry', 'lama1277-baipi', 'tani'), ('to_cry', 'p-mala1545-acd', '*taŋis'), ('to_cry', 'lama1277-waiwa', 'tani'), ('to_cry', 'lama1277-wuake', 'tani'), ('to_cry', 'lama1277-lewoi', 'ˈtaniŋ'), ('to_cry', 'lama1277-waiba', 'tani'), ('to_cry', 'lama1277-pukau', 'taniŋ'), ('to_cry', 'lama1277-lamal', 'tani'), ('to_cry', 'lama1277-merde', 'tani'), ('to_cry', 'sika1262-hewa', 'tani'), ('to_cry', 'lama1277-horow', 'tani'), ('to_cry', 'lama1277-botun', 'tani'), ('to_cry', 'p-mala1545-abvd', '*taŋis'), ('to_cry', 'alor1247-munas', 'taniʔ'), ('to_cry', 'lama1277-mulan', 'tani'), ('to_cry', 'alor1247-pandai', 'tani'), ('to_cry', 'lama1277-belan', 'tani'), ('to_cry', 'lama1277-lewom', 'tani'), ('to_cry', 'lama1277-ritae', 'tani'), ('to_cry', 'lama1277-tanju', 'tani'), ('to_cry', 'lama1277-adona', 'ˈtani'), ('to_cry', 'lama1277-lewoe', 'tani'), ('to_cry', 'lama1277-kiwan', 'tani'), ('to_cry', 'lama1277-lamah', 'tani'), ('to_cry', 'lama1277-lamat', 'tani'), ('to_cry', 'p-mala1545-acd', '*C<um>aŋis'), ('to_cry', 'alor1247-munas', 'taniŋ'), ('to_cry', 'tetu1245-suai', 'tanɪs'), ('to_cry', 'kama1365', 'tanei'), ('to_cry', 'lama1277-ileap', 'tani'), ('to_cry', 'lama1277-dulhi', 'tani')}


Concept ‘to_know’ is different between Alorese and Lamaholot.

 One or more Lamaholot dialects innovated forms not found in Alorese:
 {('to_know', 'p-mala1545-acd', '*taqu'), ('to_know', 'lama1277-lewoe', 'təgo'), ('to_know', 'p-mala1545-abvd', '*taqu')}
 {('to_know', 'lama1277-paina', 'ketan'), ('to_know', 'lama1277-kalik', 'ketən'), ('to_know', 'lama1277-paina', 'metan'), ('to_know', 'baka1276', 'teiŋ'), ('to_know', 'blag1240-tuntu', 'gadaŋ'), ('to_know', 'lama1277-lewok', 'ketən'), ('to_know', 'lama1277-lerek', 'meˈtən'), ('to_know', 'sawi1256', "lite'maina"), ('to_know', 'pura1258', "a'teiŋ"), ('to_know', 'wers1238-taram', 'leteŋ'), ('to_know', 'blag1240-bama', 'gadaŋ')}
 {('to_know', 'kabo1247', 'ʔel (we)'), ('to_know', 'lama1277-kalik', '-etən'), ('to_know', 'adan1251-otvai', 'a’hul'), ('to_know', 'adan1251-lawah', 'ahøl'), ('to_know', 'hama1240', 'ahʊl')}
 {('to_know', 'lama1277-imulo', 'kin'), ('to_know', 'kama1365', 'kaŋ'), ('to_know', 'kafo1240', 'gɛn'), ('to_know', 'lama1277-lewop', 'kin')}
 {('to_know', 'lama1277-ileap', 'toi'), ('to_know', 'lama1277-mulan', 'toi'), ('to_know', 'lama1277-belan', 'toi'), ('to_know', 'blag1240-kulij', 'tei'), ('to_know', 'lama1277-lewoi', 'tɔi'), ('to_know', 'lama1277-lamal', 'toi'), ('to_know', 'lama1277-lewot', 'toi'), ('to_know', 'lama1277-lamat', 'ʔoi')}
 {('to_know', 'tetu1246', "hɑ'tɛnɛ"), ('to_know', 'tetu1245-vique', "ha'tɛnɛ"), ('to_know', 'lama1277-minga', 'tina'), ('to_know', 'tetu1245-suai', "ha'tɛnɛ"), ('to_know', 'lama1277-lewuk', 'metəna')}

  Just for clarity:
  There are also the following similarity classes shared between Alorese and LH.
  {('to_know', 'lama1277-lewop', 'min'), ('to_know', 'lama1277-waiba', 'moi'), ('to_know', 'lama1277-wuake', 'moi'), ('to_know', 'lama1277-lewob', 'moi'), ('to_know', 'lama1277-lamak', 'moi'), ('to_know', 'lama1277-waiwa', 'moi'), ('to_know', 'lama1277-watan', 'moi'), ('to_know', 'lama1277-lewom', 'moi'), ('to_know', 'lama1277-dulhi', 'moi'), ('to_know', 'lama1277-botun', 'moi'), ('to_know', 'lama1277-ritae', 'moi'), ('to_know', 'lama1277-kiwan', 'moi'), ('to_know', 'lama1277-pukau', 'moi'), ('to_know', 'lama1277-baipi', 'moi'), ('to_know', 'lama1277-lamat', 'moi'), ('to_know', 'lama1277-bama', 'moi'), ('to_know', 'lama1277-lewog', 'moi'), ('to_know', 'lama1277-tanju', 'moi'), ('to_know', 'lama1277-imulo', 'min'), ('to_know', 'lama1277-horow', 'moi'), ('to_know', 'alor1247-pandai', 'moiŋ')}
  {('to_know', 'lama1277-lewoi', 'nɔi'), ('to_know', 'lama1277-adona', 'ˈnoi'), ('to_know', 'alor1247-pandai', 'noiŋ'), ('to_know', 'keda1252', 'nuan'), ('to_know', 'keda1252-leuba', 'nuan')}
  {('to_know', 'lama1277-botun', 'koi'), ('to_know', 'lama1277-waiwa', 'koi'), ('to_know', 'lama1277-lamak', 'koi'), ('to_know', 'lama1277-lewob', 'koi'), ('to_know', 'alor1247-munas', 'koiŋ'), ('to_know', 'lama1277-baipi', 'koi'), ('to_know', 'alor1247-besar', 'koiŋ'), ('to_know', 'lama1277-pukau', 'koi'), ('to_know', 'lama1277-kiwan', 'koi'), ('to_know', 'lama1277-merde', 'koi'), ('to_know', 'lama1277-lewog', 'koi'), ('to_know', 'lama1277-adona', 'ˈkoi'), ('to_know', 'lama1277-watan', 'koi'), ('to_know', 'lama1277-wuake', 'koi'), ('to_know', 'lama1277-horow', 'koi'), ('to_know', 'lama1277-bama', 'koi'), ('to_know', 'lama1277-dulhi', 'koi'), ('to_know', 'lama1277-lewom', 'koi'), ('to_know', 'lama1277-ritae', 'koi'), ('to_know', 'lama1277-lamah', 'koi'), ('to_know', 'lama1277-waiba', 'koi'), ('to_know', 'alor1247-pandai', 'koiŋ'), ('to_know', 'lama1277-tanju', 'koi')}


Concept ‘to_crouch’ is different between Alorese and Lamaholot.

 Alorese innovated the following forms:
 {('to_crouch', 'alor1247-munas', "tɔbɔ 'tɛlɛng"), ('to_crouch', 'alor1247-besar', "tɔbɔ 'tae")}
 {('to_crouch', 'alor1247-pandai', "sɔrɔ'bɔkɔŋ")}
 {('to_crouch', 'alor1247-besar', "tɔbɔ 'tae"), ('to_crouch', 'alor1247-munas', "tɔbɔ 'tɛlɛŋ")}
 One or more Lamaholot dialects innovated forms not found in Alorese:
 {('to_crouch', 'lama1277-adona', 'ˈpraɡa')}
 {('to_crouch', 'lama1277-lerek', 'həvaˈkat'), ('to_crouch', 'lama1277-kalik', 'svaˈkat')}

  Just for clarity:
  There are also the following similarity classes shared between Alorese and LH.
  {('to_crouch', 'alor1247-pandai', 'lugu'), ('to_crouch', 'atim1239', 'luk ˈbo'), ('to_crouch', 'p-alor1249', '*luk(V)'), ('to_crouch', 'wers1238-taram', 'lukuk'), ('to_crouch', 'sawi1256', "a'luku"), ('to_crouch', 'lama1277-lewoi', 'ˈluɡuŋ'), ('to_crouch', 'buna1278-suai', 'thouk'), ('to_crouch', 'p-timo1261', '*luk(V)'), ('to_crouch', 'abui1241-fuime', 'luke'), ('to_crouch', 'kula1280-lanto', 'aluku'), ('to_crouch', 'p-east2519', '*luk'), ('to_crouch', 'kelo1247-hopte', "lu'luk"), ('to_crouch', 'buna1278-bobon', 'thouk'), ('to_crouch', 'kuii1253', 'luk'), ('to_crouch', 'abui1241-ulaga', 'luka'), ('to_crouch', 'lamm1241-westp', 'luk:iŋ'), ('to_crouch', 'wers1238-marit', "lu'kuk")}


Concept ‘to_wake_someone_up’ is different between Alorese and Lamaholot.

 Alorese innovated the following forms:
 {('to_wake_someone_up', 'alor1247-pandai', "guo 'bauŋ"), ('to_wake_someone_up', 'alor1247-pandai', 'pui'), ('to_wake_someone_up', 'alor1247-pandai', "pui 'bauŋ"), ('to_wake_someone_up', 'alor1247-munas', 'pui'), ('to_wake_someone_up', 'alor1247-baran', "guɔ 'baũ")}
 {('to_wake_someone_up', 'alor1247-besar', "bakuŋ 'tidɛ")}
 One or more Lamaholot dialects innovated forms not found in Alorese:
 {('to_wake_someone_up', 'lama1277-lewoi', 'soɡo ˈhoɡo')}
 {('to_wake_someone_up', 'lama1277-adona', 'ˈʔio'), ('to_wake_someone_up', 'lama1277-kalik', 'ˈriɔ'), ('to_wake_someone_up', 'lama1277-lerek', 'ˈriɔ')}
 {('to_wake_someone_up', 'lama1277-lewoi', 'neiŋ ˈhoɡo')}


Concept ‘conjunctivitis’ is different between Alorese and Lamaholot.

 One or more Lamaholot dialects innovated forms not found in Alorese:
 {('conjunctivitis', 'lama1277-adona', 'kɜˈrara')}

  Just for clarity:
  There are also the following similarity classes shared between Alorese and LH.
  {('conjunctivitis', 'lama1277-lewoi', 'mata ˈmeʔa'), ('conjunctivitis', 'sika1262-hewa', 'mata taʔin'), ('conjunctivitis', 'tetu1245-vique', "mata 'moras"), ('conjunctivitis', 'tetu1245-suai', "mata 'moras"), ('conjunctivitis', 'alor1247-munas', "mataŋ 'meaŋ"), ('conjunctivitis', 'alor1247-munas', "mataʔ 'meaŋ")}
  {('conjunctivitis', 'alor1247-pandai', "mataŋ b'lara"), ('conjunctivitis', 'alor1247-besar', "mataŋ 'lara"), ('conjunctivitis', 'lama1277-kalik', 'maˈta ˈklara')}


Concept ‘flea’ is different between Alorese and Lamaholot.

 Alorese innovated the following forms:
 {('flea', 'alor1247-munas', 'bɛgal'), ('flea', 'alor1247-pandai', 'begal')}
 One or more Lamaholot dialects innovated forms not found in Alorese:
 {('flea', 'blag1240-kulij', 'ʤavar kubaŋ'), ('flea', 'lama1277-lewoi', 'ˈkəpi'), ('flea', 'baka1276', 'kubaŋ'), ('flea', 'rett1240', '(ʤobal) gaɓaŋ')}

  Just for clarity:
  There are also the following similarity classes shared between Alorese and LH.
  {('flea', 'alor1247-munas', "mə'rːɛ"), ('flea', 'kabo1247', "teme'le"), ('flea', 'lama1277-kalik', 'təməˈlar'), ('flea', 'lama1277-lerek', 'təməˈlar'), ('flea', 'blag1240-nule', "tə'mela"), ('flea', 'sika1262-hewa', 'məla'), ('flea', 'p-mala1545-acd', '*qatimela'), ('flea', 'pura1258', "tata 'mɛra"), ('flea', 'alor1247-besar', "aho ta'mela"), ('flea', 'lama1277-kalik', 'təməˈla'), ('flea', 'lama1277-adona', 'tɜˈmɜla'), ('flea', 'blag1240-bama', 'tepela'), ('flea', 'keda1252-leuba', 'mɛlɛ')}


Concept ‘to_answer’ is different between Alorese and Lamaholot.

 Alorese innovated the following forms:
 {('to_answer', 'alor1247-munas', "hodɛ k'dirɛʔ"), ('to_answer', 'alor1247-pandai', 'hɔde')}
 {('to_answer', 'alor1247-besar', 'taliŋ')}
 One or more Lamaholot dialects innovated forms not found in Alorese:
 {('to_answer', 'buna1278-suai', 'thaban'), ('to_answer', 'lama1277-adona', 'ˈtapã'), ('to_answer', 'lama1277-lewoi', 'ˈtapɑŋ'), ('to_answer', 'lama1277-kalik', 'taˈpan'), ('to_answer', 'buna1278-bobon', 'tha:n')}
 {('to_answer', 'keda1252-leuba', 'hɔlɔ'), ('to_answer', 'lama1277-lerek', 'ˈholoi')}
 {('to_answer', 'lama1277-lerek', 'pəˈnua')}


Concept ‘sorcerer’ is different between Alorese and Lamaholot.

 Alorese innovated the following forms:
 {('sorcerer', 'alor1247-pandai', 'dɔti')}
 {('sorcerer', 'alor1247-munas', "m'nakaʔ"), ('sorcerer', 'alor1247-pandai', "m'naka"), ('sorcerer', 'alor1247-besar', "ma'naka")}
 {('sorcerer', 'alor1247-pandai', "sə'her")}
 One or more Lamaholot dialects innovated forms not found in Alorese:
 {('sorcerer', 'lama1277-lewoi', 'ˈmolaŋ'), ('sorcerer', 'keda1252-leuba', 'mɔlan')}
 {('sorcerer', 'lama1277-adona', 'sɜˈnɜmu')}
 {('sorcerer', 'lama1277-lerek', 'ˈeoŋ')}
 {('sorcerer', 'lama1277-kalik', 'ˈmakap  ˈtaŋən')}

  Just for clarity:
  There are also the following similarity classes shared between Alorese and LH.
  {('sorcerer', 'alor1247-pandai', "su'waŋgi"), ('sorcerer', 'lama1277-lerek', 'ˈsvaŋɡi')}


Concept ‘child’ is different between Alorese and Lamaholot.

 Alorese innovated the following forms:
 {('child', 'alor1247-pandai', 'bai')}
 {('child', 'pura1258', "bal ka'kari"), ('child', 'pura1258', 'wal kakari'), ('child', 'alor1247-baran', "bi're ka'ri:")}
 Alorese shares the following forms with TAP languages:
 {('child', 'alor1247-pandai', 'bai')}
 {('child', 'pura1258', "bal ka'kari"), ('child', 'pura1258', 'wal kakari'), ('child', 'alor1247-baran', "bi're ka'ri:")}
 One or more Lamaholot dialects innovated forms not found in Alorese:
 {('child', 'lama1277-pukau', 'kəreŋ')}
 {('child', 'lama1277-kalik', 'kopoŋ'), ('child', 'lama1277-kalik', 'kɔˈpɔ'), ('child', 'lama1277-kalik', 'kɔˈpɔŋ'), ('child', 'lama1277-lewuk', 'kopoŋ')}

  Just for clarity:
  There are also the following similarity classes shared between Alorese and LH.
  {('child', 'keda1252', 'anaʔ'), ('child', 'lama1277-tanju', 'ana'), ('child', 'lama1277-lerek', 'anaˈkən'), ('child', 'koto1251', 'anah'), ('child', 'lama1277-botun', 'ana'), ('child', 'idat1237', "wa'nɑn"), ('child', 'lama1277-wuake', 'ana'), ('child', 'koto1251', 'riʔanaʔ'), ('child', 'lama1277-lamah', 'ana'), ('child', 'lama1277-lewom', 'ana'), ('child', 'lama1277-ritae', 'ana'), ('child', 'lama1277-kiwan', 'anaʔ'), ('child', 'lama1277-lewot', 'anakja'), ('child', 'laka1255', 'ʔanɑn'), ('child', 'lama1277-adona', 'ˈʔanaʔ'), ('child', 'lama1277-lewok', 'nuya'), ('child', 'lama1277-ileap', 'ana'), ('child', 'lama1277-baipi', 'ana'), ('child', 'lama1277-watan', 'ana'), ('child', 'laka1255', 'au anak'), ('child', 'lama1277-belan', 'ana'), ('child', 'lama1277-horow', 'anaʔ'), ('child', 'lama1277-lamat', 'ana ranã'), ('child', 'lama1277-merde', 'ana'), ('child', 'lama1277-lewoi', 'ˈanaʔ'), ('child', 'kema1243', 'ʔɑnɑr'), ('child', 'p-mala1545-acd', '*anak'), ('child', 'lama1277-lamak', 'ana'), ('child', 'lama1277-waiwa', 'ana'), ('child', 'lama1277-lewog', 'anaʔ'), ('child', 'alor1247-munas', 'anaʔ'), ('child', 'tuku1254', 'ʔana'), ('child', 'p-aust1307-abvd', '*aNak'), ('child', 'keda1252-leuwa', 'a̘naʔ'), ('child', 'lama1277-lewop', 'ana'), ('child', 'lama1277-mulan', 'ana'), ('child', 'mamb1306', 'ʔana'), ('child', 'lama1277-waiba', 'ana'), ('child', 'lama1277-lewob', 'anaʔ'), ('child', 'lama1277-imulo', 'ana'), ('child', 'lama1277-paina', 'anaken'), ('child', 'p-mala1545-abvd', '*anak'), ('child', 'alor1247-baran', 'ʔanaŋ'), ('child', 'p-cent2245-abvd', '*anak'), ('child', 'lama1277-lamal', 'ana'), ('child', 'lama1277-minga', 'anuja'), ('child', 'alor1247-munas', 'ana'), ('child', 'lama1277-lewoe', 'ana'), ('child', 'lama1277-bama', 'anaʔ'), ('child', 'alor1247-munas', 'anaŋ'), ('child', 'keda1252-leuba', 'anaʔ'), ('child', 'lama1277-dulhi', 'anaʔ'), ('child', 'alor1247-besar', 'anaŋ')}


Concept ‘to_kill’ is different between Alorese and Lamaholot.

 Alorese shares the following forms with other AN languanges, but not Lamaholot:
 {('to_kill', 'p-mala1545-abvd', '*bunuq'), ('to_kill', 'p-cent2245-abvd', '*bunuq'), ('to_kill', 'alor1247-pandai', 'bunɔ'), ('to_kill', 'p-mala1545-acd', '*bunuq'), ('to_kill', 'tetu1245-vique', 'funu'), ('to_kill', 'alor1247-besar', 'bunɔ'), ('to_kill', 'alor1247-munas', 'bunɔ'), ('to_kill', 'alor1247-baran', 'bun:ɔ')}
 One or more Lamaholot dialects innovated forms not found in Alorese:
 {('to_kill', 'lama1277-bama', 'belo'), ('to_kill', 'lama1277-mulan', 'səbəle'), ('to_kill', 'baka1276', 'buj'), ('to_kill', 'lama1277-lewoe', 'belu'), ('to_kill', 'lama1277-lamat', 'belu'), ('to_kill', 'lama1277-lamak', 'belo'), ('to_kill', 'keda1252', 'beloʔ'), ('to_kill', 'lama1277-paina', 'həbal'), ('to_kill', 'lama1277-watan', 'belo'), ('to_kill', 'lama1277-kalik', 'belu'), ('to_kill', 'lama1277-waiba', 'belo'), ('to_kill', 'lama1277-kiwan', 'belo'), ('to_kill', 'lama1277-botun', 'belo'), ('to_kill', 'lama1277-lewog', 'belo'), ('to_kill', 'lama1277-baipi', 'belo'), ('to_kill', 'lama1277-dulhi', 'belo'), ('to_kill', 'lama1277-lewok', 'belu'), ('to_kill', 'lama1277-lewom', 'belo'), ('to_kill', 'lama1277-ritae', 'belo'), ('to_kill', 'lama1277-lamah', 'belo'), ('to_kill', 'lama1277-adona', 'ˈbelo'), ('to_kill', 'lama1277-lewoi', 'ˈbelo'), ('to_kill', 'lama1277-ileap', 'belo'), ('to_kill', 'lama1277-waiwa', 'belo'), ('to_kill', 'lama1277-horow', 'belo'), ('to_kill', 'keda1252-leuba', 'bel'), ('to_kill', 'lama1277-lewop', 'belu'), ('to_kill', 'lama1277-lewob', 'belo'), ('to_kill', 'lama1277-tanju', 'belo'), ('to_kill', 'lama1277-pukau', 'belo'), ('to_kill', 'lama1277-imulo', 'bel')}
 {('to_kill', 'lama1277-lerek', 'məˈraŋ na ˈmatei')}
 {('to_kill', 'lama1277-lewot', 'təbajak'), ('to_kill', 'lama1277-minga', 'təbajak'), ('to_kill', 'lama1277-lamal', 'təbajak'), ('to_kill', 'lama1277-belan', 'təbajak'), ('to_kill', 'lama1277-lewuk', 'təbajak'), ('to_kill', 'lama1277-wuake', 'təbajak')}
 {('to_kill', 'lama1277-kalik', '-ar maˈtaʤa')}


Concept ‘blind’ is different between Alorese and Lamaholot.

 Alorese innovated the following forms:
 {('blind', 'alor1247-munas', 'kɔmak')}
 Alorese shares the following forms with other AN languanges, but not Lamaholot:
 {('blind', 'alor1247-pandai', "mataŋ 'date"), ('blind', 'tetu1245-suai', 'mata at'), ('blind', 'tetu1245-vique', 'mata at'), ('blind', 'alor1247-besar', "mataŋ 'date")}
 One or more Lamaholot dialects innovated forms not found in Alorese:
 {('blind', 'lama1277-kalik', 'kniˈpɛk'), ('blind', 'lama1277-lerek', 'maˈta kiˈpɛk')}
 {('blind', 'lama1277-adona', 'kɜˈnisũ')}
 {('blind', 'lama1277-adona', 'kɜˈbuʔlɔ̃')}
 {('blind', 'lamm1241-westp', 'bad:aŋ'), ('blind', 'kama1365', 'bita'), ('blind', 'lama1277-lewoi', 'ˈbuta'), ('blind', 'lamm1241-westp', 'taiti bad:aŋ'), ('blind', 'p-mala1545-acd', '*buta')}


Concept ‘cloud’ is different between Alorese and Lamaholot.

 Alorese shares the following forms with TAP languages:
 {('cloud', 'hama1240', 'tafɛʔɪŋ'), ('cloud', 'alor1247-pandai', 'awaŋ'), ('cloud', 'kafo1240', 'tɑbɛkɛl'), ('cloud', 'kira1248', 'tabes'), ('cloud', 'kuii1253', 'tebes'), ('cloud', 'abui1241-takal', "tä.be̞ä.'kil"), ('cloud', 'adan1251-otvai', 'ta’fiʔiŋ')}
 Alorese shares the following forms with other AN languanges, but not Lamaholot:
 {('cloud', 'mamb1306', "sɑ'bɑi"), ('cloud', 'alor1247-pandai', 'sapːu'), ('cloud', 'laka1255', "sa'bɑi"), ('cloud', 'tuku1254', "sɑ'bɑi 'tɛʔ")}
 One or more Lamaholot dialects innovated forms not found in Alorese:
 {('cloud', 'lama1277-mulan', 'kəkə')}

  Just for clarity:
  There are also the following similarity classes shared between Alorese and LH.
  {('cloud', 'lama1277-paina', 'kowar'), ('cloud', 'keda1252', 'owa'), ('cloud', 'lama1277-lewoe', 'ʔowa'), ('cloud', 'lama1277-lewog', 'kowa'), ('cloud', 'lama1277-lewuk', 'kowar'), ('cloud', 'keda1252-leuba', "ʔɔ'wa"), ('cloud', 'lama1277-lamah', 'kowa'), ('cloud', 'lama1277-botun', 'kowa'), ('cloud', 'lama1277-lewoi', 'kɔwa'), ('cloud', 'alor1247-besar', 'kɔfa'), ('cloud', 'lama1277-pukau', 'kowa'), ('cloud', 'sika1262-hewa', 'koʋa'), ('cloud', 'lama1277-imulo', 'kofar'), ('cloud', 'lama1277-watan', 'kowa'), ('cloud', 'lama1277-kalik', 'koˈva'), ('cloud', 'lama1277-belan', 'kowa'), ('cloud', 'lama1277-kalik', 'koˈvar'), ('cloud', 'lama1277-lewop', 'kofaru'), ('cloud', 'lama1277-tanju', 'kowa'), ('cloud', 'lama1277-baipi', 'kowa'), ('cloud', 'lama1277-lamal', 'kofa'), ('cloud', 'lama1277-waiba', 'kowa'), ('cloud', 'lama1277-lewok', 'kowaru'), ('cloud', 'lama1277-lamak', 'kowa'), ('cloud', 'lama1277-ileap', 'kowa'), ('cloud', 'lama1277-merde', 'kowa'), ('cloud', 'lama1277-dulhi', 'kowa'), ('cloud', 'sika1262-hewa', 'kowa'), ('cloud', 'lama1277-lamat', 'ʔowa'), ('cloud', 'lama1277-lewom', 'kowa'), ('cloud', 'lama1277-bama', 'kowa'), ('cloud', 'lama1277-lewob', 'kowa'), ('cloud', 'lama1277-ritae', 'kowa'), ('cloud', 'lama1277-lerek', 'ˈkovar'), ('cloud', 'lama1277-kalik', 'kowa'), ('cloud', 'lama1277-lewot', 'kofaru'), ('cloud', 'lama1277-minga', 'kowa'), ('cloud', 'lama1277-adona', 'ˈkɔːwa'), ('cloud', 'lama1277-wuake', 'kowa'), ('cloud', 'lama1277-kiwan', 'kowa')}
  {('cloud', 'alor1247-munas', 'nabaʔ'), ('cloud', 'lama1277-horow', 'abak'), ('cloud', 'lama1277-waiwa', 'abak'), ('cloud', 'abui1241-ulaga', 'tabok')}


Concept ‘bed’ is different between Alorese and Lamaholot.

 Alorese innovated the following forms:
 {('bed', 'alor1247-munas', "dɛki 'turu")}
 {('bed', 'alor1247-pandai', "sə'ligaŋ"), ('bed', 'alor1247-besar', "sa'ligaŋ")}
 One or more Lamaholot dialects innovated forms not found in Alorese:
 {('bed', 'lama1277-lewoi', 'ˈbuaŋ')}
 {('bed', 'lama1277-kalik', 'uˈlik')}
 {('bed', 'lama1277-adona', 'kɜˈnatã')}
 {('bed', 'lama1277-lerek', 'kəneˈpiŋ')}


Concept ‘to_wait’ is different between Alorese and Lamaholot.

 Alorese shares the following forms with TAP languages:
 {('to_wait', 'blag1240-warsa', 'gadiŋ'), ('to_wait', 'baka1276', 'adiŋ'), ('to_wait', 'blag1240-bama', 'gadiŋ'), ('to_wait', 'alor1247-pandai', 'tiaŋ'), ('to_wait', 'blag1240-tuntu', 'tadiŋ'), ('to_wait', 'blag1240-kulij', 'adiŋ'), ('to_wait', 'blag1240-nule', 'ʔadiŋ'), ('to_wait', 'kaer1234', "ga'riŋ"), ('to_wait', 'pura1258', 'adiŋ')}
 {('to_wait', 'alor1247-munas', 'baiŋ'), ('to_wait', 'alor1247-besar', 'baiŋ'), ('to_wait', 'alor1247-pandai', 'baiŋ'), ('to_wait', 'kira1248', 'bana')}
 One or more Lamaholot dialects innovated forms not found in Alorese:
 {('to_wait', 'lama1277-kalik', 'kide'), ('to_wait', 'lama1277-kalik', 'kliˈde')}
 {('to_wait', 'lama1277-lerek', 'baˈlɛŋ')}

  Just for clarity:
  There are also the following similarity classes shared between Alorese and LH.
  {('to_wait', 'lama1277-lewoi', 'ˈniaŋ'), ('to_wait', 'lama1277-adona', 'ˈmĩa'), ('to_wait', 'alor1247-pandai', 'riaŋ'), ('to_wait', 'alor1247-munas', 'miaŋ'), ('to_wait', 'alor1247-pandai', 'kiaŋ'), ('to_wait', 'alor1247-pandai', 'miaŋ')}


Concept ‘brother’ is different between Alorese and Lamaholot.

 Alorese innovated the following forms:
 {('brother', 'alor1247-pandai', 'tata')}
 {('brother', 'alor1247-besar', "naŋ ka'lake")}
 {('brother', 'alor1247-pandai', 'ula')}
 Alorese shares the following forms with other AN languanges, but not Lamaholot:
 {('brother', 'alor1247-pandai', 'naŋ'), ('brother', 'alor1247-munas', 'naʔ'), ('brother', 'sika1262-hewa', 'nara'), ('brother', 'alor1247-munas', 'naŋ ')}
 One or more Lamaholot dialects innovated forms not found in Alorese:
 {('brother', 'buna1278-malia', "khɑu 'kha'a"), ('brother', 'nede1245', 'geŋ'), ('brother', 'maka1316', 'kaka'), ('brother', 'fata1247', 'kaka'), ('brother', 'lama1277-adona', 'kaka'), ('brother', 'teiw1235', 'gian qai')}
 {('brother', 'lama1277-adona', 'ʔariʔ'), ('brother', 'lama1277-lewoi', 'ariʔ'), ('brother', 'keda1252-leuba', 'ariʔ')}
 {('brother', 'keda1252-leuba', 'aʔe'), ('brother', 'lama1277-lewoi', 'aʔe')}


Concept ‘wound’ is different between Alorese and Lamaholot.

 Alorese shares the following forms with other AN languanges, but not Lamaholot:
 {('wound', 'p-mala1545-acd', '*nuka'), ('wound', 'alor1247-munas', 'ukak'), ('wound', 'alor1247-munas', 'ugar'), ('wound', 'alor1247-besar', 'uka'), ('wound', 'alor1247-pandai', 'ugar'), ('wound', 'alor1247-pandai', 'uka')}
 One or more Lamaholot dialects innovated forms not found in Alorese:
 {('wound', 'lama1277-lerek', 'ɡaˈjak'), ('wound', 'lama1277-kalik', 'ɡaˈʤak')}
 {('wound', 'lama1277-adona', 'ˈʔuŋarɜt')}
 {('wound', 'lama1277-lewoi', 'bəˈlaraŋ')}


Concept ‘difficult’ is different between Alorese and Lamaholot.

 Alorese innovated the following forms:
 {('difficult', 'adan1251-otvai', 'ka’rak'), ('difficult', 'alor1247-besar', "ka'raki")}
 {('difficult', 'alor1247-pandai', 'padʒa')}
 Alorese shares the following forms with TAP languages:
 {('difficult', 'adan1251-otvai', 'ka’rak'), ('difficult', 'alor1247-besar', "ka'raki")}
 {('difficult', 'alor1247-pandai', 'padʒa')}
 One or more Lamaholot dialects innovated forms not found in Alorese:
 {('difficult', 'lama1277-lewoi', 'ˈsulit'), ('difficult', 'keda1252-leuba', 'sulit'), ('difficult', 'sika1262-hewa', 'sulit')}
 {('difficult', 'lama1277-kalik', 'snuˈkar')}
 {('difficult', 'lama1277-lerek', 'ɡəˈnukə')}
 {('difficult', 'kama1365', '-0'), ('3sg', 'lama1277-lerek', '(mɔˈne)'), ('tree', 'p-aust1307-abvd', '*(kayu)'), ('Earth', 'wers1238-taram', '(dunia)'), ('thatch_for_roofing', 'kama1365', '-0'), ('ice', 'rett1240', '-0'), ('field', 'kama1365', '-0'), ('sister', 'p-mala1545-acd', '-0'), ('to_refuse', 'kama1365', '-0'), ('to_lie_down', 'p-mala1545-acd', '-0'), ('turtle_saltwater', 'rett1240', '-0'), ('betel_nut_areca', 'kama1365', '-0'), ('rice_plant', 'kama1365', '-0'), ('snow', 'rett1240', '-0'), ('3sg', 'lama1277-lerek', '(mɔn)'), ('initiation_ceremony', 'rett1240', '-0'), ('deer', 'p-mala1545-acd', '-0'), ('ant', 'p-mala1545-acd', '-0'), ('fly', 'p-mala1545-acd', '-0'), ('to_bless', 'rett1240', '-0'), ('brother', 'p-mala1545-acd', '-0'), ('to_accuse', 'rett1240', '-0'), ('necklace', 'p-mala1545-acd', '-0'), ('cuscus', 'rett1240', '-0'), ('priest_modern', 'p-mala1545-acd', '-0'), ('short', 'kama1365', '-0')}

  Just for clarity:
  There are also the following similarity classes shared between Alorese and LH.
  {('difficult', 'alor1247-munas', 'tudaʔ'), ('difficult', 'lama1277-adona', 'ˈtudak')}


Concept ‘to_defend’ is different between Alorese and Lamaholot.

 Alorese innovated the following forms:
 {('to_defend', 'alor1247-besar', 'ʤaga')}
 {('to_defend', 'alor1247-munas', 'rɛmba')}
 Alorese shares the following forms with other AN languanges, but not Lamaholot:
 {('to_defend', 'alor1247-pandai', 'likɔ'), ('to_defend', 'keda1252-leuba', "li'kɔʔ")}
 One or more Lamaholot dialects innovated forms not found in Alorese:
 {('to_defend', 'lama1277-adona', 'ˈnɜtĩ')}
 {('to_defend', 'lama1277-kalik', 'məˈtiŋa liˈti')}
 {('to_defend', 'adan1251-otvai', 'ʔei’taʔ'), ('to_defend', 'lama1277-lewoi', 'ˈhipaʔ')}


Concept ‘taro’ is different between Alorese and Lamaholot.

 Alorese shares the following forms with TAP languages:
 {('taro', 'blag1240-nule', 'awgol'), ('taro', 'alor1247-besar', "ag'ɔl"), ('taro', 'blag1240-kulij', 'awgol'), ('taro', 'hama1240', 'akol'), ('taro', 'rett1240', 'aigol'), ('taro', 'adan1251-otvai', 'a’gɔl'), ('taro', 'baka1276', 'awgol'), ('taro', 'pura1258', 'au gol')}
 One or more Lamaholot dialects innovated forms not found in Alorese:
 {('taro', 'lama1277-lerek', 'keˈvok')}

  Just for clarity:
  There are also the following similarity classes shared between Alorese and LH.
  {('taro', 'kula1280-lanto', 'a’lata'), ('taro', 'alor1247-munas', "kwə'lːaːʔ"), ('taro', 'lama1277-lewoi', 'ˈkəladi'), ('taro', 'kaer1234', 'kɛladi'), ('taro', 'kula1280-lanto', 'alata'), ('taro', 'alor1247-pandai', "kə'walːa"), ('taro', 'sawi1256', "a'lata"), ('taro', 'lama1277-adona', 'kəˈlɑdi'), ('taro', 'fata1247', "ka'lahi")}


Concept ‘stick_pole’ is different between Alorese and Lamaholot.

 Alorese shares the following forms with TAP languages:
 {('stick_pole', 'alor1247-besar', 'dɔba'), ('stick_pole', 'adan1251-lawah', 'dopoŋ'), ('stick_pole', 'kabo1247', 'ti bo'), ('stick_pole', 'adan1251-otvai', '’dɔpaŋ'), ('stick_pole', 'pura1258', 'doba'), ('stick_pole', 'baka1276', 'doba'), ('stick_pole', 'kelo1247-hopte', 'dɔb')}
 One or more Lamaholot dialects innovated forms not found in Alorese:
 {('stick_pole', 'lama1277-adona', "brɜ'dɔ̃")}
 {('stick_pole', 'lama1277-kalik', 'nɔˈdɔk'), ('stick_pole', 'blag1240-kulij', 'hudok'), ('stick_pole', 'lama1277-lerek', 'ˈnodok')}

  Just for clarity:
  There are also the following similarity classes shared between Alorese and LH.
  {('stick_pole', 'rett1240', 'tei kaŋ'), ('stick_pole', 'wers1238-taram', 'dekeŋ'), ('stick_pole', 'kama1365', 'teːkaŋ'), ('stick_pole', 'alor1247-pandai', "tə'nɛkaŋ"), ('stick_pole', 'sawi1256', "dika'na"), ('stick_pole', 'lama1277-lewoi', 'ˈnəkəŋ'), ('stick_pole', 'alor1247-pandai', 'nɛkaŋ'), ('stick_pole', 'wers1238-marit', 'dɛkɛŋ'), ('stick_pole', 'rett1240', 'te:kaŋ'), ('stick_pole', 'alor1247-munas', "nə'kaŋ")}


Concept ‘tall’ is different between Alorese and Lamaholot.

 One or more Lamaholot dialects innovated forms not found in Alorese:
 {('tall', 'lama1277-lerek', 'ataˈdikə ɡoloˈkən')}

  Just for clarity:
  There are also the following similarity classes shared between Alorese and LH.
  {('tall', 'alor1247-munas', "b'lɔlɔk"), ('tall', 'alor1247-besar', "ba'lɔlɔ"), ('tall', 'baka1276', 'blolu'), ('tall', 'blag1240-nule', 'məlolu'), ('tall', 'blag1240-tuntu', 'bololu'), ('tall', 'lama1277-lewoi', 'bəˈlola'), ('tall', 'rett1240', 'balo:lu'), ('tall', 'rett1240', "ba'lolu"), ('tall', 'blag1240-warsa', 'bəlolu'), ('tall', 'lama1277-adona', 'blɔʔˈlɔ̃'), ('tall', 'blag1240-kulij', 'blolu'), ('tall', 'blag1240-bama', 'blolu'), ('tall', 'pura1258', "nehe ba'lolu")}
  {('tall', 'alor1247-munas', "b'lɔlɔk"), ('tall', 'alor1247-besar', "ba'lɔlɔ"), ('tall', 'alor1247-pandai', "b'lolɔk"), ('tall', 'lama1277-kalik', 'gɔˈlɔk')}


Concept ‘to_see’ is different between Alorese and Lamaholot.

 One or more Lamaholot dialects innovated forms not found in Alorese:
 {('to_see', 'lama1277-lewom', 'tədə'), ('to_see', 'lama1277-lewop', 'tene'), ('to_see', 'lama1277-ritae', 'tədə'), ('to_see', 'hama1240', 'dun'), ('to_see', 'sika1262-hewa', 'təŋer'), ('to_see', 'kabo1247', 'edunu'), ('to_see', 'lama1277-lewog', 'tədə'), ('to_see', 'lama1277-lamak', 'təda'), ('to_see', 'adan1251-lawah', 'dunuʔ'), ('to_see', 'sika1262-hewa', 'teŋer'), ('to_see', 'lama1277-bama', 'təda'), ('to_see', 'lama1277-lewoi', 'ˈtəŋə'), ('to_see', 'lama1277-lewoe', 'todo'), ('to_see', 'lama1277-lewot', 'tene'), ('to_see', 'lama1277-watan', 'tədaʔ'), ('to_see', 'lama1277-baipi', 'tədə'), ('to_see', 'fata1247', 'tɔtɔ')}
 {('to_see', 'lama1277-dulhi', 'hulã'), ('to_see', 'rett1240', 'ʔuliŋ'), ('to_see', 'lama1277-botun', 'hulã'), ('to_see', 'lama1277-horow', 'hulã'), ('to_see', 'lama1277-lamah', 'hulã'), ('to_see', 'lama1277-wuake', 'hule'), ('to_see', 'lama1277-waiwa', 'hulã'), ('to_see', 'kama1365', 'ilai'), ('to_see', 'lama1277-adona', 'ˈhulɜ'), ('to_see', 'lama1277-kiwan', 'hulə')}
 {('to_see', 'lama1277-lamal', 'təgəl')}
 {('to_see', 'lama1277-kalik', 'du'), ('to_see', 'lama1277-lerek', 'ˈtulu'), ('to_see', 'lama1277-lewuk', 'du')}
 {('to_see', 'wers1238-marit', "gə'dir"), ('to_see', 'lama1277-belan', 'koi'), ('to_see', 'kelo1247-bring', 'gəur'), ('to_see', 'lama1277-waiba', 'koi'), ('to_see', 'lama1277-tanju', 'koi'), ('to_see', 'kelo1247-hopte', "ge'ʔur")}
 {('to_see', 'p-mala1545-abvd', '*kita'), ('to_see', 'lama1277-lewob', 'notõ'), ('to_see', 'kira1248', 'gatik'), ('to_see', 'kama1365', '-tak'), ('to_see', 'sika1262-hewa', 'ʔitaʔ'), ('to_see', 'p-cent2245-abvd', '*kita'), ('to_see', 'p-aust1307-abvd', '*kita'), ('to_see', 'koto1251', 'n-ita'), ('to_see', 'p-mala1545-acd', '*kita'), ('to_see', 'sika1262-tanai', 'ʔita'), ('to_see', 'kama1365', 'gatɛk')}

  Just for clarity:
  There are also the following similarity classes shared between Alorese and LH.
  {('to_see', 'alor1247-baran', 'sɛru'), ('to_see', 'alor1247-munas', 'sɛruʔ'), ('to_see', 'lama1277-imulo', 'sure'), ('to_see', 'alor1247-besar', 'sɛru'), ('to_see', 'alor1247-pandai', 'sɛru')}
  {('to_see', 'lama1277-ileap', 'niku'), ('to_see', 'lamm1241-westp', '-niaka'), ('to_see', 'sika1262-tanai', 'niʔa'), ('to_see', 'koto1251', 'n-kisu'), ('to_see', 'sika1262-maume', 'niʔa'), ('to_see', 'alor1247-baran', 'hik:i')}
  {('to_see', 'lama1277-lamat', 'bəlele'), ('to_see', 'dein1238', 'bil'), ('to_see', 'blag1240-warsa', 'lala'), ('to_see', 'sarr1247-nule', 'bil'), ('to_see', 'lama1277-lewok', 'bele'), ('to_see', 'kaer1234', "la'lo"), ('to_see', 'lama1277-paina', 'bəlele'), ('to_see', 'blag1240-tuntu', 'lala'), ('to_see', 'lama1277-minga', 'bəlele'), ('to_see', 'nede1245', 'bili'), ('to_see', 'blag1240-bama', 'lala'), ('to_see', 'lama1277-mulan', 'bele'), ('to_see', 'lama1277-pukau', 'bələr'), ('to_see', 'alor1247-besar', "ka'lalaŋ"), ('to_see', 'teiw1235', "bə'li"), ('to_see', 'sarr1247-adiab', 'bil'), ('to_see', 'lama1277-merde', 'bəlele')}


Concept ‘to_swell’ is different between Alorese and Lamaholot.

 Alorese innovated the following forms:
 {('to_swell', 'alor1247-pandai', 'ihiŋ'), ('to_swell', 'alor1247-munas', 'hiŋ'), ('to_swell', 'alor1247-besar', 'ihĩ'), ('to_swell', 'alor1247-munas', 'ihiŋ')}
 One or more Lamaholot dialects innovated forms not found in Alorese:
 {('to_swell', 'lama1277-kalik', 'snoˈbaʤ')}
 {('to_swell', 'lama1277-wuake', 'kəbəte'), ('to_swell', 'lama1277-minga', 'kəbetɤ')}
 {('to_swell', 'lama1277-ileap', 'bā'), ('to_swell', 'lama1277-kalik', 'sənobaj'), ('to_swell', 'lama1277-belan', 'hobaj'), ('to_swell', 'lama1277-dulhi', 'baʔa'), ('to_swell', 'lama1277-lewog', 'baʔa'), ('to_swell', 'lama1277-lewob', 'baʔa'), ('to_swell', 'lama1277-watan', 'baʔa'), ('to_swell', 'lama1277-horow', 'baʔa'), ('to_swell', 'lama1277-lamak', 'baʔa'), ('to_swell', 'lama1277-lewuk', 'sobaj'), ('to_swell', 'lama1277-lewok', 'sobaj'), ('to_swell', 'keda1252', 'babaŋ'), ('to_swell', 'lama1277-ritae', 'baʔa'), ('to_swell', 'lama1277-lewom', 'baʔa'), ('to_swell', 'lama1277-bama', 'baʔa'), ('to_swell', 'lama1277-baipi', 'baʔa'), ('to_swell', 'lama1277-paina', 'kəhobay'), ('to_swell', 'lama1277-waiwa', 'baʔ'), ('to_swell', 'lama1277-botun', 'baʔa'), ('to_swell', 'lama1277-kiwan', 'baʔa'), ('to_swell', 'lama1277-tanju', 'baʔa'), ('to_swell', 'lama1277-waiba', 'baʔa')}
 {('to_swell', 'sika1262-hewa', 'bowo'), ('to_swell', 'lama1277-pukau', 'bowo')}
 {('to_swell', 'lama1277-lewoe', 'mawa')}
 {('to_swell', 'lama1277-lerek', 'kəˈhobɔi')}
 {('to_swell', 'lama1277-lamah', 'hogor'), ('to_swell', 'lama1277-lamat', 'hogo'), ('to_swell', 'lama1277-merde', 'nogor')}
 {('to_swell', 'lama1277-lewop', 'kəbaraha'), ('to_swell', 'lama1277-mulan', 'kebaras'), ('to_swell', 'lama1277-lamal', 'kəbaras'), ('to_swell', 'lama1277-lewot', 'kəbarah'), ('to_swell', 'lama1277-imulo', 'kəbarasa')}

  Just for clarity:
  There are also the following similarity classes shared between Alorese and LH.
  {('to_swell', 'buna1278-bobon', 'phɛ'), ('to_swell', 'alor1247-besar', 'bao'), ('to_swell', 'lama1277-adona', 'ˈbaʔa'), ('to_swell', 'buna1278-malia', 'phe:'), ('to_swell', 'keda1252-leuba', 'babaŋ'), ('to_swell', 'alor1247-baran', 'baɔ'), ('to_swell', 'buna1278-suai', 'phɛ'), ('to_swell', 'lama1277-lewoi', 'ˈbaʔa'), ('to_swell', 'alor1247-pandai', 'baŋ')}


Concept ‘wide’ is different between Alorese and Lamaholot.

 Alorese shares the following forms with TAP languages:
 {('wide', 'kula1280-lanto', 'lowana'), ('wide', 'alor1247-pandai', "p'lawaʔ"), ('wide', 'kula1280-lanto', 'la’wana')}
 One or more Lamaholot dialects innovated forms not found in Alorese:
 {('wide', 'lama1277-lewob', 'luas')}
 {('wide', 'abui1241-petle', 'foka'), ('wide', 'tetu1246', "lu'ɑn"), ('wide', 'lama1277-lamak', 'loŋar'), ('wide', 'kuii1253', 'aka'), ('wide', 'laka1255', 'mlua:r'), ('wide', 'mamb1306', "m'lua"), ('wide', 'abui1241-petle', 'fo.ko'), ('wide', 'kema1243', "mɔ'lɔɑ"), ('wide', 'idat1237', "ma'luɑr"), ('wide', 'lama1277-watan', 'longar'), ('wide', 'tuku1254', "bu'luɑr"), ('wide', 'lama1277-lewok', 'loŋgar'), ('wide', 'tetu1245-suai', 'luan'), ('wide', 'tetu1245-vique', 'luaŋ')}
 {('wide', 'lamm1241-westp', 'salala'), ('wide', 'lama1277-lamah', 'kəlarã'), ('wide', 'p-mala1545-acd', '*lawa'), ('wide', 'blag1240-warsa', 'sarara'), ('wide', 'blag1240-nule', 'lara'), ('wide', 'blag1240-kulij', 'lara'), ('wide', 'baka1276', 'lara'), ('wide', 'blag1240-warsa', 'sararaʤli'), ('wide', 'p-aust1307-abvd', '*ma-lawas'), ('wide', 'p-mala1545-acd', '*lawas'), ('wide', 'kira1248', 'silara')}
 {('wide', 'lama1277-lerek', 'kedaˈkən'), ('wide', 'lama1277-kalik', 'kedak'), ('wide', 'keda1252-leuwa', 'kεdεŋ kεnaʔ'), ('wide', 'lama1277-lewuk', 'kedak'), ('wide', 'lama1277-paina', 'kedakən')}

  Just for clarity:
  There are also the following similarity classes shared between Alorese and LH.
  {('wide', 'lama1277-lamat', 'pəloʔe'), ('wide', 'lama1277-mulan', 'belɤ'), ('wide', 'lama1277-waiba', 'bəlã'), ('wide', 'alor1247-besar', "pa'leŋa"), ('wide', 'lama1277-botun', 'pəlelet'), ('wide', 'lama1277-dulhi', 'belʔã'), ('wide', 'lama1277-lewog', 'belə'), ('wide', 'lama1277-bama', 'belã'), ('wide', 'lama1277-lewop', 'pelafatən'), ('wide', 'lama1277-ileap', 'pəloen'), ('wide', 'lama1277-lewot', 'pəlafa'), ('wide', 'lama1277-baipi', 'bəlapa'), ('wide', 'lama1277-kalik', 'pleˈdan'), ('wide', 'lama1277-merde', 'belã'), ('wide', 'lama1277-minga', 'pəlawat'), ('wide', 'alor1247-pandai', "p'lengaŋ"), ('wide', 'lama1277-ritae', 'pəloʔẽ'), ('wide', 'alor1247-baran', 'bɛa'), ('wide', 'lama1277-adona', 'ˈbeleʔ'), ('wide', 'lama1277-wuake', 'belɁẽ'), ('wide', 'lama1277-kiwan', 'pəlelet'), ('wide', 'lama1277-lewoi', 'ˈbelə'), ('wide', 'sika1262-hewa', 'pəloeŋ'), ('wide', 'lama1277-pukau', 'belʔã'), ('wide', 'lama1277-lamal', 'pelafat'), ('wide', 'lama1277-belan', 'pəlagaj'), ('wide', 'lama1277-waiwa', 'palelet'), ('wide', 'lama1277-tanju', 'belə'), ('wide', 'lama1277-lewoe', 'bəle'), ('wide', 'lama1277-lewom', 'belẽ'), ('wide', 'lama1277-horow', 'pəlelet'), ('wide', 'lama1277-imulo', 'pəlafat'), ('wide', 'alor1247-munas', "p'lɛŋaŋ")}


Concept ‘stomach_belly’ is different between Alorese and Lamaholot.

 One or more Lamaholot dialects innovated forms not found in Alorese:
 {('stomach_belly', 'lama1277-imulo', 'tukasa usər')}
 {('stomach_belly', 'lama1277-ileap', 'tain onən')}
 {('stomach_belly', 'lama1277-lewoe', 'ũhãlau')}
 {('stomach_belly', 'lama1277-lewok', 'taik'), ('stomach_belly', 'lama1277-lewog', 'tãi')}
 {('stomach_belly', 'lama1277-mulan', 'kəboti'), ('stomach_belly', 'lama1277-waiwa', 'tãikəboti'), ('stomach_belly', 'lama1277-lamat', 'lau boti'), ('stomach_belly', 'lama1277-dulhi', 'kəboti'), ('stomach_belly', 'lama1277-ritae', 'kəboti'), ('stomach_belly', 'lama1277-watan', 'kəbotik'), ('stomach_belly', 'lama1277-lamak', 'kəboti'), ('stomach_belly', 'lama1277-waiba', 'tãikəboti'), ('stomach_belly', 'lama1277-lewop', 'tai kəboti'), ('stomach_belly', 'lama1277-baipi', 'tãikəboti'), ('stomach_belly', 'lama1277-lewot', 'tukaha kebotiha'), ('stomach_belly', 'lama1277-lamal', 'kəboti'), ('stomach_belly', 'lama1277-kiwan', 'kəbotik'), ('stomach_belly', 'lama1277-lewom', 'taikəboti'), ('stomach_belly', 'lama1277-tanju', 'tãi kəboti'), ('stomach_belly', 'lama1277-bama', 'kəboti'), ('stomach_belly', 'lama1277-kalik', 'tai kəbotin'), ('stomach_belly', 'lama1277-minga', 'kəbotisa')}
 {('stomach_belly', 'lama1277-belan', 'taik kaluŋ'), ('stomach_belly', 'lama1277-merde', 'taik kalunã'), ('stomach_belly', 'lama1277-wuake', 'taik kaluŋ'), ('stomach_belly', 'lama1277-lewob', 'tãi kənaluŋ')}
 {('stomach_belly', 'lama1277-paina', 'tai oray'), ('stomach_belly', 'lama1277-lewuk', 'taisa orasa'), ('stomach_belly', 'lama1277-horow', 'lorã'), ('stomach_belly', 'lama1277-botun', 'taik lorã')}
 {('stomach_belly', 'lama1277-lewob', 'luwu'), ('stomach_belly', 'lama1277-lewog', 'luwu'), ('stomach_belly', 'lama1277-lewoi', 'ˈluwuŋ'), ('stomach_belly', 'lama1277-belan', 'luwuk'), ('stomach_belly', 'lama1277-lamal', 'lufu')}
 {('stomach_belly', 'lama1277-pukau', 'dulak onoʔo'), ('stomach_belly', 'sika1262-hewa', 'tai dula'), ('stomach_belly', 'lama1277-lamah', 'taikət onã')}
 {('stomach_belly', 'lama1277-ritae', 'ale'), ('stomach_belly', 'lama1277-tanju', 'ale'), ('stomach_belly', 'lama1277-baipi', 'ale'), ('stomach_belly', 'lama1277-lewom', 'ale'), ('stomach_belly', 'lama1277-waiba', 'alek')}

  Just for clarity:
  There are also the following similarity classes shared between Alorese and LH.
  {('stomach_belly', 'alor1247-munas', 'tukaʔ'), ('stomach_belly', 'alor1247-pandai', 'tukaŋ'), ('stomach_belly', 'alor1247-munas', 'tukaŋ'), ('stomach_belly', 'p-mala1545-acd', '*bituka'), ('stomach_belly', 'lama1277-lewot', 'tukaha'), ('stomach_belly', 'lama1277-imulo', 'tukasa'), ('stomach_belly', 'lama1277-lewop', 'tuka')}
  {('stomach_belly', 'lama1277-horow', 'taik'), ('stomach_belly', 'lama1277-lewok', 'taiga'), ('stomach_belly', 'lama1277-botun', 'taik'), ('stomach_belly', 'lama1277-kiwan', 'taik'), ('stomach_belly', 'lama1277-merde', 'taik'), ('stomach_belly', 'sika1262-hewa', 'dula'), ('stomach_belly', 'lama1277-dulhi', 'taol'), ('stomach_belly', 'lama1277-kalik', 'tai'), ('stomach_belly', 'lama1277-bama', 'dulak'), ('stomach_belly', 'lama1277-lamak', 'taĩ'), ('stomach_belly', 'lama1277-ileap', 'taik'), ('stomach_belly', 'lama1277-adona', 'taĩ'), ('stomach_belly', 'koto1251', 'tai-f'), ('stomach_belly', 'lama1277-lamah', 'taik'), ('stomach_belly', 'alor1247-pandai', 'taiŋ'), ('stomach_belly', 'lama1277-adona', 'taim'), ('stomach_belly', 'lama1277-pukau', 'dulak'), ('stomach_belly', 'lama1277-minga', 'taisa'), ('stomach_belly', 'lama1277-wuake', 'taik'), ('stomach_belly', 'lama1277-watan', 'taĩ'), ('stomach_belly', 'lama1277-lewuk', 'taisa'), ('stomach_belly', 'rett1240', 'ɔtɔk'), ('stomach_belly', 'lama1277-paina', 'taih')}
  {('stomach_belly', 'lama1277-lamat', 'boti'), ('stomach_belly', 'keda1252-leuba', "bɔ'tin"), ('stomach_belly', 'blag1240-kulij', 'tow'), ('stomach_belly', 'lama1277-lerek', 'kəboˈtin'), ('stomach_belly', 'p-mala1545-abvd', '*tian'), ('stomach_belly', 'lama1277-waiwa', 'tãiŋ'), ('stomach_belly', 'lama1277-mulan', 'tɤi'), ('stomach_belly', 'alor1247-baran', 'teiŋ'), ('stomach_belly', 'keda1252', 'botin'), ('stomach_belly', 'p-cent2245-abvd', '*tian'), ('stomach_belly', 'lama1277-lewoe', 'botih'), ('stomach_belly', 'alor1247-besar', 'teiŋ'), ('stomach_belly', 'sika1262-tanai', 'taʔi-ŋ'), ('stomach_belly', 'sika1262-maume', 't̪aʔin'), ('stomach_belly', 'keda1252-leuwa', 'bɔtin')}


Concept ‘friend’ is different between Alorese and Lamaholot.

 Alorese shares the following forms with other AN languanges, but not Lamaholot:
 {('friend', 'keda1252-leuba', 'niruŋ'), ('friend', 'alor1247-munas', 'niru')}

  Just for clarity:
  There are also the following similarity classes shared between Alorese and LH.
  {('friend', 'alor1247-besar', "ba'rːe-ba'rːe"), ('friend', 'lama1277-lewoi', 'ˈbreuŋ'), ('friend', 'alor1247-pandai', "ba'rːe"), ('friend', 'lama1277-kalik', 'kəˈneu'), ('friend', 'lama1277-lerek', 'kəneˈbreuŋ'), ('friend', 'lama1277-adona', 'ˈrɛu')}


Concept ‘to_think’ is different between Alorese and Lamaholot.

 Alorese innovated the following forms:
 {('to_think', 'alor1247-besar', 'iŋa')}
 Alorese shares the following forms with other AN languanges, but not Lamaholot:
 {('to_think', 'blag1240-nule', 'ukur'), ('to_think', 'blag1240-tuntu', 'veŋpikir'), ('to_think', 'blag1240-kulij', 'ukur'), ('to_think', 'kira1248', 'ukar'), ('to_think', 'pura1258', "veŋ 'ukur"), ('to_think', 'blag1240-warsa', 'veŋ-okur'), ('to_think', 'baka1276', 'ukur'), ('to_think', 'alor1247-baran', 'pikir'), ('to_think', 'keda1252-leuba', 'pikir')}
 One or more Lamaholot dialects innovated forms not found in Alorese:
 {('to_think', 'lama1277-lewog', 'nəkət'), ('to_think', 'lama1277-bama', 'nəkət'), ('to_think', 'lama1277-pukau', 'nəkət'), ('to_think', 'lama1277-lewoi', 'ˈnəkə'), ('to_think', 'tuku1254', 'nurat'), ('to_think', 'kema1243', 'nurɑt')}
 {('to_think', 'tetu1245-suai', "ha'noin"), ('to_think', 'buna1278-bobon', 'ganoin'), ('to_think', 'laka1255', "ʔa'nuin"), ('to_think', 'buna1278-suai', 'ganoin'), ('to_think', 'tetu1246', "hɑ'noin"), ('to_think', 'lama1277-paina', 'gənuan'), ('to_think', 'idat1237', "ano'in"), ('to_think', 'tetu1245-vique', "ha'nɔjɪŋ")}
 {('to_think', 'lama1277-lewob', 'hu'), ('to_think', 'kama1365', 'suk pak'), ('to_think', 'sika1262-hewa', 'sugo'), ('to_think', 'lama1277-lamat', 'huʔə'), ('to_think', 'sika1262-hewa', 'suɡo'), ('to_think', 'sika1262-tanai', 'huk'), ('to_think', 'lama1277-lewoe', 'huã'), ('to_think', 'kama1365', 'suk'), ('to_think', 'sika1262-maume', 'huk'), ('to_think', 'lama1277-lerek', 'uˈkoi')}
 {('to_think', 'lama1277-lewuk', 'petən'), ('to_think', 'lama1277-minga', 'petə'), ('to_think', 'lama1277-lamal', 'petã'), ('to_think', 'lama1277-horow', 'petən'), ('to_think', 'lama1277-merde', 'petã'), ('to_think', 'lama1277-ileap', 'petən'), ('to_think', 'lama1277-lamak', 'petã'), ('to_think', 'lama1277-lamah', 'petən'), ('to_think', 'lama1277-mulan', 'petɤ'), ('to_think', 'lama1277-wuake', 'petã'), ('to_think', 'lama1277-botun', 'petən'), ('to_think', 'lama1277-watan', 'petã'), ('to_think', 'lama1277-kiwan', 'petə'), ('to_think', 'lama1277-kalik', 'petən'), ('to_think', 'lama1277-dulhi', 'petəŋ'), ('to_think', 'lama1277-belan', 'pet'), ('to_think', 'lama1277-waiwa', 'petən'), ('to_think', 'lama1277-ritae', 'petə'), ('to_think', 'lama1277-adona', 'ˈpetɜ'), ('to_think', 'lama1277-imulo', 'petən'), ('to_think', 'lama1277-lewot', 'petən'), ('to_think', 'lama1277-lewop', 'hedəp'), ('to_think', 'lama1277-kalik', 'ˈpetən'), ('to_think', 'lama1277-lewok', 'petən')}

  Just for clarity:
  There are also the following similarity classes shared between Alorese and LH.
  {('to_think', 'alor1247-pandai', 'guaŋ'), ('to_think', 'rett1240', 'ganukuŋ'), ('to_think', 'lama1277-lewom', 'hukut'), ('to_think', 'lama1277-waiba', 'ukut'), ('to_think', 'alor1247-pandai', "g'nuku"), ('to_think', 'lama1277-tanju', 'ukut'), ('to_think', 'lama1277-baipi', 'hukut'), ('to_think', 'alor1247-munas', "g'nukuʔ"), ('to_think', 'alor1247-besar', "ga'nokuŋ")}


Concept ‘drizzle’ is different between Alorese and Lamaholot.

 Alorese shares the following forms with TAP languages:
 {('drizzle', 'alor1247-munas', "uraŋ sə'rːik"), ('drizzle', 'alor1247-pandai', "uraŋ 'rɛrɛ"), ('drizzle', 'alor1247-pandai', "uraŋ 'sarːik"), ('drizzle', 'alor1247-besar', "uraŋ fa'rɛha-fa'rɛha"), ('drizzle', 'blag1240-kulij', 'sirik-sirik'), ('drizzle', 'alor1247-munas', "uraŋ 'brɛhak")}
 One or more Lamaholot dialects innovated forms not found in Alorese:
 {('drizzle', 'lama1277-lewoi', 'kiteˈritɛŋ')}
 {('drizzle', 'lama1277-kalik', 'uˈʤan ɡrəˈtik'), ('drizzle', 'lama1277-lerek', 'ˈudʒ͡a krenik')}
 {('drizzle', 'lama1277-adona', "ɡri'ʔɛna")}


Concept ‘older_sister’ is different between Alorese and Lamaholot.

 Alorese innovated the following forms:
 {('older_sister', 'alor1247-besar', "kakaŋ ka'fae")}
 {('older_sister', 'alor1247-munas', "tata k'wae "), ('older_sister', 'alor1247-pandai', "tata k'wae")}
 One or more Lamaholot dialects innovated forms not found in Alorese:
 {('older_sister', 'keda1252-leuba', 'aʔe'), ('older_sister', 'lama1277-lewoi', 'aʔe (arian)'), ('older_sister', 'lama1277-adona', 'ʔariʔ')}
 {('older_sister', 'lama1277-adona', 'kaka')}


Concept ‘rack_above_hearth’ is different between Alorese and Lamaholot.

 Alorese innovated the following forms:
 {('rack_above_hearth', 'rett1240', 'hɛrɛ'), ('rack_above_hearth', 'alor1247-pandai', 'hɛrɛ'), ('rack_above_hearth', 'alor1247-munas', 'hɛrɛʔ')}
 {('rack_above_hearth', 'adan1251-otvai', '’huruŋ'), ('rack_above_hearth', 'alor1247-pandai', 'huruŋ'), ('rack_above_hearth', 'alor1247-besar', 'huruŋ')}
 {('rack_above_hearth', 'alor1247-pandai', 'kɛlɛr')}
 Alorese shares the following forms with TAP languages:
 {('rack_above_hearth', 'rett1240', 'hɛrɛ'), ('rack_above_hearth', 'alor1247-pandai', 'hɛrɛ'), ('rack_above_hearth', 'alor1247-munas', 'hɛrɛʔ')}
 {('rack_above_hearth', 'adan1251-otvai', '’huruŋ'), ('rack_above_hearth', 'alor1247-pandai', 'huruŋ'), ('rack_above_hearth', 'alor1247-besar', 'huruŋ')}
 {('rack_above_hearth', 'alor1247-pandai', 'kɛlɛr')}
 One or more Lamaholot dialects innovated forms not found in Alorese:
 {('rack_above_hearth', 'lama1277-kalik', 'maˈgar'), ('rack_above_hearth', 'lama1277-adona', 'maɡa')}
 {('rack_above_hearth', 'lama1277-adona', 'kɜˈnala')}
 {('rack_above_hearth', 'abui1241-ulaga', 'parik'), ('rack_above_hearth', 'lama1277-lewoi', 'ˈraːk')}
 {('rack_above_hearth', 'lama1277-lerek', 'nɛˈkɛt')}


Concept ‘road’ is different between Alorese and Lamaholot.

 Alorese shares the following forms with TAP languages:
 {('road', 'dein1238', 'tor'), ('road', 'alor1247-munas', 'tɔr'), ('road', 'alor1247-baran', 'tɔ:r'), ('road', 'dein1238', 'wutor')}

  Just for clarity:
  There are also the following similarity classes shared between Alorese and LH.
  {('road', 'lama1277-bama', 'larã'), ('road', 'lama1277-lewob', 'larã'), ('road', 'lama1277-lewuk', 'lalan'), ('road', 'lama1277-botun', 'rarã'), ('road', 'kema1243', 'sala'), ('road', 'laka1255', 'luron'), ('road', 'tetu1245-vique', 'dalaŋ'), ('road', 'lama1277-lamal', 'larã'), ('road', 'p-cent2245-abvd', '*zalan'), ('road', 'keda1252', 'lala'), ('road', 'lama1277-belan', 'lara'), ('road', 'sika1262-maume', 'lalan'), ('road', 'idat1237', "lɑla i'na:k"), ('road', 'sika1262-tanai', 'lala-ŋ'), ('road', 'alor1247-pandai', 'laraŋ'), ('road', 'alor1247-besar', 'laraŋ'), ('road', 'tetu1246', 'dɑlɑn'), ('road', 'lama1277-lewog', 'laran'), ('road', 'lama1277-mulan', 'larã'), ('road', 'lama1277-ileap', 'laran'), ('road', 'lama1277-paina', 'lalan'), ('road', 'lama1277-waiba', 'larã'), ('road', 'lama1277-lerek', 'ˈlalan'), ('road', 'lama1277-lewop', 'lalan'), ('road', 'lama1277-lewoe', 'laran'), ('road', 'sika1262-hewa', 'laran'), ('road', 'lama1277-lewom', 'rarã'), ('road', 'sika1262-hewa', 'larã'), ('road', 'lama1277-tanju', 'larã'), ('road', 'lama1277-dulhi', 'rarã'), ('road', 'lama1277-imulo', 'lala'), ('road', 'lama1277-lewoi', 'ˈlaraŋ'), ('road', 'lama1277-kalik', 'laˈla'), ('road', 'lama1277-horow', 'rarã'), ('road', 'lama1277-lamak', 'rarã'), ('road', 'tetu1245-suai', 'dalaŋ'), ('road', 'lama1277-lewok', 'lalanu'), ('road', 'lama1277-kalik', 'lalan'), ('road', 'alor1247-munas', 'laraŋ'), ('road', 'koto1251', 'ranan'), ('road', 'lama1277-adona', 'rarã'), ('road', 'lama1277-waiwa', 'larã'), ('road', 'lama1277-ritae', 'larã'), ('road', 'lama1277-baipi', 'rarã'), ('road', 'mamb1306', "sa'lolo"), ('road', 'p-aust1307-abvd', '*zalan'), ('road', 'p-mala1545-acd', '*zalan'), ('road', 'lama1277-minga', 'lalan'), ('road', 'p-mala1545-abvd', '*zalan'), ('road', 'lama1277-kiwan', 'rarã'), ('road', 'keda1252-leuba', 'lala'), ('road', 'lama1277-lewot', 'lalanu'), ('road', 'lama1277-lamat', 'larã'), ('road', 'lama1277-watan', 'rarã'), ('road', 'lama1277-pukau', 'larã'), ('road', 'lama1277-merde', 'larã'), ('road', 'lama1277-wuake', 'lara'), ('road', 'lama1277-lamah', 'laran'), ('road', 'lama1277-kalik', 'laˈlan')}


Concept ‘rotten’ is different between Alorese and Lamaholot.

 Alorese innovated the following forms:
 {('rotten', 'alor1247-munas', "ulɔr 'mirɔʔ")}
 {('rotten', 'alor1247-besar', 'nihiŋ')}
 One or more Lamaholot dialects innovated forms not found in Alorese:
 {('rotten', 'lama1277-lewuk', 'suŋaw'), ('rotten', 'buna1278-malia', 'gunɑs'), ('rotten', 'buna1278-malia', 'nuɑs')}
 {('rotten', 'rett1240', 'manisa'), ('rotten', 'pura1258', "ehi ma'nisa"), ('rotten', 'baka1276', "mi'nisa"), ('rotten', 'blag1240-warsa', 'menisa'), ('rotten', 'blag1240-tuntu', 'minisa'), ('rotten', 'blag1240-kulij', 'misa'), ('rotten', 'blag1240-nule', 'misa'), ('rotten', 'lama1277-horow', 'mənutã'), ('rotten', 'blag1240-bama', 'minisa')}
 {('rotten', 'keda1252-leuba', "bu'ruʔ"), ('rotten', 'p-cent2245-abvd', '*buʀuk'), ('rotten', 'keda1252-leuwa', 'buruʔ balaŋ'), ('rotten', 'p-mala1545-abvd', '*ma-buɾuk'), ('rotten', 'p-mala1545-acd', '*buRuk'), ('rotten', 'p-mala1545-abvd', '*ma-busuk'), ('rotten', 'buna1278-suai', 'busuɁ'), ('rotten', 'buna1278-bobon', 'busuɁ'), ('rotten', 'keda1252', 'buruʔ'), ('rotten', 'lama1277-lewot', 'buru'), ('rotten', 'p-mala1545-acd', '*busuk'), ('rotten', 'p-aust1307-abvd', '*ma-buʀaq'), ('rotten', 'kema1243', 'busu'), ('rotten', 'p-cent2245-abvd', '*busuk')}
 {('rotten', 'lama1277-lerek', 'dəvəˈkən')}
 {('rotten', 'lama1277-lerek', 'ɡuˈtun')}

  Just for clarity:
  There are also the following similarity classes shared between Alorese and LH.
  {('rotten', 'lama1277-kalik', 'kədaˈtək'), ('rotten', 'alor1247-baran', 'datɛ'), ('rotten', 'tetu1246', "dɔ'dɔk"), ('rotten', 'tetu1245-vique', 'dɔdɔk'), ('rotten', 'lama1277-adona', 'ˈdatɜ'), ('rotten', 'tetu1245-suai', 'dɔdɔk')}
  {('rotten', 'alor1247-munas', 'wouʔ'), ('rotten', 'lama1277-lewop', 'fuk'), ('rotten', 'alor1247-besar', 'fou'), ('rotten', 'lama1277-imulo', 'fuk'), ('rotten', 'lama1277-minga', 'fukɤ')}
  {('rotten', 'lama1277-watan', 'wãu'), ('rotten', 'lama1277-lewoe', 'weu'), ('rotten', 'lama1277-lewom', 'wãu'), ('rotten', 'lama1277-ritae', 'wãu'), ('rotten', 'lama1277-lewob', 'wuhu'), ('rotten', 'lama1277-botun', 'wãu'), ('rotten', 'lama1277-lamal', 'fãu'), ('rotten', 'lama1277-kiwan', 'wãu'), ('rotten', 'lama1277-tanju', 'wãu'), ('rotten', 'sika1262-hewa', 'ʋauk'), ('rotten', 'lama1277-pukau', 'wãu'), ('rotten', 'sika1262-tanai', 'βauk'), ('rotten', 'lama1277-dulhi', 'wãu'), ('rotten', 'lama1277-lewok', 'wauk'), ('rotten', 'lama1277-ileap', 'waun'), ('rotten', 'lama1277-lamah', 'waũ'), ('rotten', 'lama1277-wuake', 'wau'), ('rotten', 'alor1247-pandai', 'wau'), ('rotten', 'lama1277-lamak', 'waũ'), ('rotten', 'sika1262-tanai', 'βau-ŋ'), ('rotten', 'lama1277-waiwa', 'wãu'), ('rotten', 'lama1277-lamat', 'weʔu'), ('rotten', 'lama1277-bama', 'wãu'), ('rotten', 'sika1262-hewa', 'wauk'), ('rotten', 'lama1277-baipi', 'wãu'), ('rotten', 'lama1277-kalik', 'wauk'), ('rotten', 'lama1277-belan', 'wau'), ('rotten', 'lama1277-lewog', 'waũ'), ('rotten', 'lama1277-merde', 'wãu'), ('rotten', 'lama1277-paina', 'wauk'), ('rotten', 'lama1277-waiba', 'wãu'), ('rotten', 'lama1277-mulan', 'wɤu'), ('rotten', 'lama1277-lewoi', 'ˈwauŋ')}


Concept ‘to_bark’ is different between Alorese and Lamaholot.

 Alorese innovated the following forms:
 {('to_bark', 'alor1247-besar', 'kabɔŋ')}
 One or more Lamaholot dialects innovated forms not found in Alorese:
 {('to_bark', 'lama1277-adona', 'ˈhobo')}
 {('to_bark', 'lama1277-lewom', 'kokar')}
 {('to_bark', 'lama1277-lamal', 'kajo kãmã'), ('to_bark', 'lama1277-tanju', 'kayokamã'), ('to_bark', 'lama1277-botun', 'kamã'), ('to_bark', 'lama1277-kalik', 'kajukamak'), ('to_bark', 'lama1277-mulan', 'kajo kɤmɤ'), ('to_bark', 'lama1277-imulo', 'kajokamak'), ('to_bark', 'lama1277-lamah', 'kayo kamʔã'), ('to_bark', 'lama1277-ritae', 'kayo kama'), ('to_bark', 'lama1277-minga', 'kajukamak'), ('to_bark', 'lama1277-lewuk', 'kajukamak'), ('to_bark', 'lama1277-wuake', 'kajokama'), ('to_bark', 'lama1277-lamak', 'kajokamʔã'), ('to_bark', 'lama1277-waiba', 'kajo kamak'), ('to_bark', 'lama1277-watan', 'karokaʔma'), ('to_bark', 'lama1277-merde', 'kayo kamʔã'), ('to_bark', 'keda1252', 'ayi amaʔ'), ('to_bark', 'lama1277-lewoe', 'əso ʔamã'), ('to_bark', 'lama1277-belan', 'kajo kama'), ('to_bark', 'lama1277-lewog', 'kayokamã'), ('to_bark', 'lama1277-kiwan', 'kayokamʔã'), ('to_bark', 'lama1277-lewop', 'kajukamak'), ('to_bark', 'lama1277-waiwa', 'kayo kama'), ('to_bark', 'lama1277-dulhi', 'kajokamʔa'), ('to_bark', 'lama1277-horow', 'kayokamã'), ('to_bark', 'lama1277-lewot', 'kaju kamakən'), ('to_bark', 'lama1277-lewok', 'kamak'), ('to_bark', 'lama1277-paina', 'kayo kamakən'), ('to_bark', 'lama1277-ileap', 'kayo kamakən')}
 {('to_bark', 'lama1277-lerek', 'ˈaor ˈtodo')}
 {('to_bark', 'lama1277-lewob', 'kajokəloka'), ('to_bark', 'lama1277-baipi', 'kajo kulit'), ('to_bark', 'sika1262-hewa', 'ulit ai'), ('to_bark', 'lama1277-lamat', 'əso ulitã'), ('to_bark', 'lama1277-pukau', 'kayokulit'), ('to_bark', 'lama1277-bama', 'kayokulit')}
 {('to_bark', 'lama1277-lewoi', 'ˈniu')}

  Just for clarity:
  There are also the following similarity classes shared between Alorese and LH.
  {('to_bark', 'alor1247-pandai', 'bɔwɔŋ'), ('to_bark', 'lama1277-lewoi', 'ˈbowo'), ('to_bark', 'keda1252-leuba', "bɔ'wɔŋ"), ('to_bark', 'lama1277-kalik', 'boˈvo'), ('to_bark', 'alor1247-munas', 'bowɔŋ')}


Concept ‘starfish’ is different between Alorese and Lamaholot.

 Alorese innovated the following forms:
 {('starfish', 'alor1247-besar', "ta'mala 'tahi")}
 {('starfish', 'alor1247-munas', "ta'pinaʔ"), ('starfish', 'alor1247-pandai', 'tapina')}
 One or more Lamaholot dialects innovated forms not found in Alorese:
 {('starfish', 'lama1277-adona', 'boˈwojõ'), ('starfish', 'lama1277-lewoi', 'bəˈwɔjɔŋ')}
 {('starfish', 'lama1277-lerek', 'toˈna loyoˈran')}


Concept ‘to_hold’ is different between Alorese and Lamaholot.

 One or more Lamaholot dialects innovated forms not found in Alorese:
 {('to_hold', 'abui1241-petle', "ho.'pu.na"), ('to_hold', 'lama1277-kalik', 'sogan'), ('to_hold', 'lama1277-lewuk', 'soga(ŋ)'), ('to_hold', 'lama1277-belan', 'soga'), ('to_hold', 'lama1277-lewok', 'soga'), ('to_hold', 'lama1277-kalik', 'soˈɡa')}
 {('to_hold', 'teiw1235', 'wanpin'), ('to_hold', 'dein1238', '(wan) pi:n'), ('to_hold', 'lama1277-merde', 'wetet'), ('to_hold', 'lama1277-paina', 'wetenəŋ'), ('to_hold', 'sarr1247-nule', 'wompin'), ('to_hold', 'sarr1247-adiab', '(wam)pin')}
 {('to_hold', 'adan1251-otvai', 'a’puin'), ('to_hold', 'kafo1240', 'gopun'), ('to_hold', 'lama1277-lewop', 'pida'), ('to_hold', 'kaer1234', "pi'no"), ('to_hold', 'keda1252-leuwa', 'pænæ'), ('to_hold', 'abui1241-takal', "pu.'nä"), ('to_hold', 'rett1240', 'puni'), ('to_hold', 'lama1277-lewot', 'pid'), ('to_hold', 'nede1245', 'pin:i'), ('to_hold', 'lama1277-minga', 'pidɤ'), ('to_hold', 'lamm1241-westp', 'pin:i'), ('to_hold', 'blag1240-bama', 'pina'), ('to_hold', 'p-timo1261', '*p(i,u)nV'), ('to_hold', 'lama1277-imulo', 'pida'), ('to_hold', 'dein1238', '(wan) pi:n'), ('to_hold', 'abui1241-ulaga', 'go:puna'), ('to_hold', 'baka1276', 'pina'), ('to_hold', 'kelo1247-bring', 'gopʊɪn'), ('to_hold', 'blag1240-kulij', 'weŋ pina'), ('to_hold', 'adan1251-lawah', 'ʔapun'), ('to_hold', 'kabo1247', 'oʔopuiɲ'), ('to_hold', 'kelo1247-hopte', "gɔ'puɲ"), ('to_hold', 'kira1248', 'gapuna'), ('to_hold', 'hama1240', 'opʊin'), ('to_hold', 'sarr1247-adiab', '(wam)pin'), ('to_hold', 'kuii1253', 'gapúna'), ('to_hold', 'blag1240-warsa', 'pina'), ('to_hold', 'abui1241-fuime', 'ho-puna'), ('to_hold', 'kama1365', 'fun'), ('to_hold', 'atim1239', 'puna'), ('to_hold', 'p-alor1249', '*p{i, u}nV'), ('to_hold', 'pura1258', 'pina'), ('to_hold', 'blag1240-tuntu', 'pina'), ('to_hold', 'blag1240-nule', "pempi'na")}
 {('to_hold', 'lama1277-pukau', 'data')}

  Just for clarity:
  There are also the following similarity classes shared between Alorese and LH.
  {('to_hold', 'lama1277-tanju', 'pehe'), ('to_hold', 'alor1247-munas', 'paha'), ('to_hold', 'lama1277-waiba', 'pehe'), ('to_hold', 'lama1277-watan', 'pehẽ'), ('to_hold', 'lama1277-lamat', 'pehe'), ('to_hold', 'lama1277-ileap', 'pehe'), ('to_hold', 'lama1277-adona', 'ˈpehẽ'), ('to_hold', 'lama1277-lamal', 'pe'), ('to_hold', 'lama1277-lewom', 'pehẽ'), ('to_hold', 'lama1277-ritae', 'pehẽ'), ('to_hold', 'lama1277-lerek', 'peheˈnaŋ'), ('to_hold', 'lama1277-lewoe', 'pehe'), ('to_hold', 'lama1277-lamak', 'pẽhe'), ('to_hold', 'alor1247-besar', 'paha'), ('to_hold', 'lama1277-waiwa', 'pehẽ'), ('to_hold', 'alor1247-baran', "pa'ha:"), ('to_hold', 'lama1277-lewob', 'pehẽ'), ('to_hold', 'lama1277-wuake', 'pehe'), ('to_hold', 'lama1277-baipi', 'pehe'), ('to_hold', 'lama1277-lamah', 'pehe'), ('to_hold', 'lama1277-lewog', 'pehẽ'), ('to_hold', 'alor1247-pandai', 'paha'), ('to_hold', 'lama1277-horow', 'pehe'), ('to_hold', 'lama1277-bama', 'pehe'), ('to_hold', 'lama1277-kiwan', 'pehẽ'), ('to_hold', 'lama1277-dulhi', 'pehẽ'), ('to_hold', 'lama1277-mulan', 'pehe'), ('to_hold', 'lama1277-botun', 'pehẽ'), ('to_hold', 'lama1277-lewoi', 'ˈpɛhɛŋ')}


Concept ‘narrow’ is different between Alorese and Lamaholot.

 Alorese shares the following forms with other AN languanges, but not Lamaholot:
 {('narrow', 'blag1240-nule', "kə'rubo"), ('narrow', 'rett1240', 'kipe'), ('narrow', 'p-mala1545-acd', '*kiput'), ('narrow', 'blag1240-warsa', 'kropok'), ('narrow', 'p-mala1545-acd', '*kipit'), ('narrow', 'kabo1247', "ke'puhu"), ('narrow', 'adan1251-otvai', 'ka’puh'), ('narrow', 'keda1252-leuba', "i'peʔ"), ('narrow', 'hama1240', 'kapuh'), ('narrow', 'alor1247-baran', "ki'p:ɛ"), ('narrow', 'p-mala1545-abvd', '*kepit'), ('narrow', 'baka1276', 'krobok'), ('narrow', 'blag1240-bama', 'kopok'), ('narrow', 'p-mala1545-acd', '*gipit'), ('narrow', 'blag1240-kulij', 'krubo'), ('narrow', 'kaer1234', 'kopok'), ('narrow', 'pura1258', "ka'puhiŋ"), ('narrow', 'rett1240', 'kipɛ'), ('narrow', 'alor1247-munas', 'kupɔʔ'), ('narrow', 'alor1247-besar', 'kipɛ')}
 One or more Lamaholot dialects innovated forms not found in Alorese:
 {('narrow', 'lama1277-kiwan', 'tudək'), ('narrow', 'lama1277-botun', 'tudak'), ('narrow', 'lama1277-waiwa', 'tudak'), ('narrow', 'lama1277-lamah', 'tudak'), ('narrow', 'lama1277-watan', 'tudak'), ('narrow', 'lama1277-mulan', 'tudak'), ('narrow', 'lama1277-tanju', 'tudək'), ('narrow', 'lama1277-lamak', 'kətudak')}
 {('narrow', 'lama1277-horow', 'tənudʔã')}
 {('narrow', 'lama1277-lerek', 'kənipoˈtən'), ('narrow', 'lama1277-lewoi', 'ˈkənɛʔ'), ('narrow', 'lama1277-adona', 'ˈkɛniʔ'), ('narrow', 'lama1277-lerek', 'hənipoˈtən')}
 {('narrow', 'lama1277-pukau', 'təkə'), ('narrow', 'lama1277-ritae', 'kətəkə'), ('narrow', 'sika1262-hewa', 'təker'), ('narrow', 'lama1277-lewob', 'kətəkə'), ('narrow', 'lama1277-ileap', 'təkəl'), ('narrow', 'lama1277-waiba', 'kətəkə'), ('narrow', 'lama1277-lewom', 'kətəker')}
 {('narrow', 'lama1277-lewoe', 'ʔuhu'), ('narrow', 'lama1277-lamat', 'uhu')}

  Just for clarity:
  There are also the following similarity classes shared between Alorese and LH.
  {('narrow', 'lama1277-belan', 'hipet'), ('narrow', 'lama1277-bama', 'kəhipuk'), ('narrow', 'lama1277-minga', 'kipo'), ('narrow', 'lama1277-wuake', 'hipet'), ('narrow', 'lama1277-lewot', 'hipet'), ('narrow', 'lama1277-dulhi', 'kipuʔ'), ('narrow', 'lama1277-merde', 'kənipo'), ('narrow', 'lama1277-paina', 'kənipotən'), ('narrow', 'lama1277-kalik', 'kniˈpɔt'), ('narrow', 'lama1277-lewog', 'kəripu'), ('narrow', 'lama1277-baipi', 'gələgipət'), ('narrow', 'lama1277-lamal', 'hipet'), ('narrow', 'lama1277-lewuk', 'kənipot'), ('narrow', 'alor1247-pandai', 'kupo'), ('narrow', 'lama1277-imulo', 'kənipotən'), ('narrow', 'keda1252', 'i̤pe'), ('narrow', 'lama1277-lewop', 'hipe'), ('narrow', 'lama1277-lewok', 'kənipot'), ('narrow', 'lama1277-kalik', 'kənipot'), ('narrow', 'alor1247-munas', 'kupɔʔ'), ('narrow', 'alor1247-besar', 'kipɛ')}


Concept ‘whale’ is different between Alorese and Lamaholot.

 Alorese shares the following forms with TAP languages:
 {('whale', 'alor1247-pandai', "k'laru"), ('whale', 'blag1240-tuntu', "qa'laru"), ('whale', 'kelo1247-hopte', "kə'lar"), ('whale', 'blag1240-kulij', 'klaru'), ('whale', 'baka1276', 'klaru'), ('whale', 'kira1248', 'kaˈlar'), ('whale', 'blag1240-warsa', 'xalaru'), ('whale', 'blag1240-bama', "ka'laru"), ('whale', 'alor1247-munas', "k'laruʔ"), ('whale', 'pura1258', "ka'laru"), ('whale', 'alor1247-besar', "ikaŋ ka'laru"), ('whale', 'blag1240-nule', "kə'laru")}
 One or more Lamaholot dialects innovated forms not found in Alorese:
 {('whale', 'lama1277-kalik', 'təˈmus'), ('whale', 'lama1277-lerek', 'təmˈhu')}
 {('whale', 'sika1262-hewa', 'iʔan paus'), ('whale', 'lama1277-lewoi', 'ˈpaus')}
 {('whale', 'lama1277-adona', 'bɜˈlɛlã')}


Concept ‘testicles’ is different between Alorese and Lamaholot.

 One or more Lamaholot dialects innovated forms not found in Alorese:
 {('testicles', 'lama1277-lerek', 'ˈleke ˈteluk')}
 {('testicles', 'lama1277-kalik', 'lɛk')}
 {('testicles', 'lama1277-lewoi', 'kuluˈʔuŋ')}

  Just for clarity:
  There are also the following similarity classes shared between Alorese and LH.
  {('testicles', 'alor1247-munas', "lahak 'kuluŋ"), ('testicles', 'tetu1245-suai', "lasa'tolun"), ('testicles', 'tetu1245-vique', "lasa'tolun"), ('testicles', 'alor1247-besar', "la'hakaŋ ku'lukuŋ"), ('testicles', 'alor1247-pandai', "lahak 'kuluŋ"), ('testicles', 'p-mala1545-acd', '*laseR'), ('testicles', 'alor1247-munas', 'lahak'), ('testicles', 'lama1277-adona', 'ˈlahak'), ('testicles', 'keda1252-leuba', "la'hɛʔ")}


Concept ‘coral_rock’ is different between Alorese and Lamaholot.

 Alorese innovated the following forms:
 {('coral_rock', 'alor1247-pandai', 'aŋgɛ')}
 {('coral_rock', 'baka1276', 'koka'), ('coral_rock', 'blag1240-bama', 'koqas'), ('coral_rock', 'kaer1234', "qo'qis"), ('coral_rock', 'alor1247-munas', 'kɔka'), ('coral_rock', 'teiw1235', "qo'qas")}
 {('coral_rock', 'alor1247-besar', "fatɔ ma'napa"), ('coral_rock', 'blag1240-nule', "mə'napa")}
 Alorese shares the following forms with TAP languages:
 {('coral_rock', 'alor1247-pandai', 'aŋgɛ')}
 {('coral_rock', 'baka1276', 'koka'), ('coral_rock', 'blag1240-bama', 'koqas'), ('coral_rock', 'kaer1234', "qo'qis"), ('coral_rock', 'alor1247-munas', 'kɔka'), ('coral_rock', 'teiw1235', "qo'qas")}
 {('coral_rock', 'alor1247-besar', "fatɔ ma'napa"), ('coral_rock', 'blag1240-nule', "mə'napa")}
 One or more Lamaholot dialects innovated forms not found in Alorese:
 {('coral_rock', 'lama1277-kalik', 'ˈvatu kaˈraŋ'), ('coral_rock', 'sika1262-hewa', 'ʋatu ruhɔ'), ('coral_rock', 'tetu1245-suai', 'fatuk ruiŋ'), ('coral_rock', 'tetu1245-vique', 'fatuk ruiŋ'), ('coral_rock', 'lama1277-lerek', 'wat uˈhor')}

  Just for clarity:
  There are also the following similarity classes shared between Alorese and LH.
  {('coral_rock', 'alor1247-pandai', 'uhuŋ'), ('coral_rock', 'lama1277-lewoi', 'ˈuhɔ'), ('coral_rock', 'lama1277-adona', 'ʔaʔɔ')}


Concept ‘liver’ is different between Alorese and Lamaholot.

 One or more Lamaholot dialects innovated forms not found in Alorese:
 {('liver', 'lama1277-belan', 'onək'), ('liver', 'lama1277-lamah', 'onəkə'), ('liver', 'lama1277-lamal', 'onã'), ('liver', 'lama1277-merde', 'onəkət'), ('liver', 'lama1277-wuake', 'one')}
 {('liver', 'lama1277-lewok', 'kəbotik ate')}
 {('liver', 'lama1277-lamat', 'huʔa'), ('liver', 'lama1277-lewoe', 'ũhã')}
 {('liver', 'lama1277-kalik', 'ɔr'), ('liver', 'lama1277-lewop', 'oraha'), ('liver', 'lama1277-paina', 'oray'), ('liver', 'lama1277-minga', 'orasa'), ('liver', 'lama1277-kalik', 'oren'), ('liver', 'lama1277-lewot', 'oraha'), ('liver', 'lama1277-lerek', 'orən'), ('liver', 'lama1277-lewuk', 'or'), ('liver', 'lama1277-imulo', 'orasa')}
 {('liver', 'lama1277-kiwan', 'mehĩ'), ('liver', 'lama1277-adona', 'ˈmehi'), ('liver', 'lama1277-dulhi', 'mehĩ')}

  Just for clarity:
  There are also the following similarity classes shared between Alorese and LH.
  {('liver', 'keda1252-leuba', "ha'tɛn"), ('liver', 'tetu1245-vique', 'aten'), ('liver', 'lama1277-baipi', 'atẽ'), ('liver', 'alor1247-pandai', 'ateŋ'), ('liver', 'lama1277-tanju', 'ate'), ('liver', 'lama1277-bama', 'atẽ'), ('liver', 'kabo1247', 'ʔom atoʔo'), ('liver', 'lama1277-lamak', 'ate'), ('liver', 'kema1243', "a'tɛr"), ('liver', 'sika1262-tanai', 'ʔβate'), ('liver', 'alor1247-baran', 'hati'), ('liver', 'lama1277-waiwa', 'ate'), ('liver', 'lama1277-watan', 'hati'), ('liver', 'laka1255', 'ʔatɛn'), ('liver', 'koto1251', 'ate-f'), ('liver', 'lama1277-horow', 'atek'), ('liver', 'sika1262-hewa', 'waten'), ('liver', 'tuku1254', 'ʔate'), ('liver', 'lama1277-lewob', 'atẽ'), ('liver', 'lama1277-pukau', 'ate'), ('liver', 'lama1277-lewom', 'atẽ'), ('liver', 'lama1277-lewog', 'ate'), ('liver', 'alor1247-besar', "a'tɛ̃"), ('liver', 'tetu1245-suai', 'aten'), ('liver', 'sika1262-hewa', 'ʋaten'), ('liver', 'lama1277-ritae', 'ate'), ('liver', 'alor1247-munas', "a'tɛŋ"), ('liver', 'lama1277-lewoi', 'ˈatɛŋ'), ('liver', 'lama1277-ileap', 'ate'), ('liver', 'lama1277-botun', 'atek'), ('liver', 'lama1277-mulan', 'ate'), ('liver', 'lama1277-waiba', 'ate'), ('liver', 'tetu1246', 'ʔatɛn'), ('liver', 'sika1262-maume', 'vat̪en'), ('liver', 'mamb1306', 'ʔɑte')}


Concept ‘skinny’ is different between Alorese and Lamaholot.

 Alorese innovated the following forms:
 {('skinny', 'alor1247-pandai', 'kɛlaŋ')}
 {('skinny', 'alor1247-besar', "ku'rusu")}
 {('skinny', 'alor1247-pandai', 'kipɛk'), ('skinny', 'alor1247-munas', 'kipɛk')}
 {('skinny', 'alor1247-munas', 'kahaŋ')}
 One or more Lamaholot dialects innovated forms not found in Alorese:
 {('skinny', 'lama1277-lerek', 'kənoˈjoŋ')}
 {('skinny', 'lama1277-lewoi', 'kəˈroɡɔŋ'), ('skinny', 'lama1277-kalik', 'krɔˈɡɔŋ')}
 {('skinny', 'lama1277-adona', 'pɜˈriɡo')}


Concept ‘to_cook’ is different between Alorese and Lamaholot.

 Alorese shares the following forms with other AN languanges, but not Lamaholot:
 {('to_cook', 'p-mala1545-abvd', '*zakan'), ('to_cook', 'p-cent2245-abvd', '*zakan'), ('to_cook', 'alor1247-besar', "dakaŋ 'apa"), ('to_cook', 'alor1247-munas', 'dakaŋ '), ('to_cook', 'alor1247-baran', 'dakaŋ'), ('to_cook', 'alor1247-pandai', 'dakaŋ ')}
 One or more Lamaholot dialects innovated forms not found in Alorese:
 {('to_cook', 'lama1277-lerek', 'ˈlolu'), ('to_cook', 'lama1277-kalik', 'loˈlu'), ('to_cook', 'lama1277-kalik', 'loluŋ')}
 {('to_cook', 'lama1277-merde', 'biho'), ('to_cook', 'lama1277-ritae', 'biso'), ('to_cook', 'lama1277-lewoi', 'ˈbiho'), ('to_cook', 'lama1277-lewob', 'biho'), ('to_cook', 'lama1277-mulan', 'biho'), ('to_cook', 'lama1277-tanju', 'biho'), ('to_cook', 'lama1277-wuake', 'biho'), ('to_cook', 'lama1277-baipi', 'biho'), ('to_cook', 'lama1277-lamah', 'biho'), ('to_cook', 'lama1277-botun', 'biho'), ('to_cook', 'lama1277-ileap', 'biho'), ('to_cook', 'lama1277-waiba', 'biho'), ('to_cook', 'lama1277-lamak', 'biho'), ('to_cook', 'lama1277-pukau', 'biho'), ('to_cook', 'lama1277-horow', 'biho'), ('to_cook', 'lama1277-lewoe', 'bihu'), ('to_cook', 'lama1277-bama', 'biho'), ('to_cook', 'lama1277-lamat', 'bihu'), ('to_cook', 'lama1277-lewom', 'biho'), ('to_cook', 'lama1277-waiwa', 'biho'), ('to_cook', 'lama1277-lewog', 'biho'), ('to_cook', 'lama1277-belan', 'bio')}
 {('to_cook', 'lama1277-lamal', 'denã'), ('to_cook', 'lama1277-watan', 'dena'), ('to_cook', 'lama1277-adona', 'ˈdena'), ('to_cook', 'buna1278-malia', 'ʧinik'), ('to_cook', 'buna1278-bobon', 'tʃinik'), ('to_cook', 'p-cent2245-abvd', '*tanək'), ('to_cook', 'p-mala1545-acd', '*tanek'), ('to_cook', 'buna1278-suai', 'tʃinik'), ('to_cook', 'p-aust1307-abvd', '*tanek'), ('to_cook', 'lama1277-kiwan', 'dena'), ('to_cook', 'p-mala1545-abvd', '*tanek'), ('to_cook', 'lama1277-dulhi', 'dena'), ('to_cook', 'abui1241-petle', 'sεn'), ('to_cook', 'maka1316', 'tina'), ('to_cook', 'abui1241-petle', 'sen'), ('to_cook', 'tetu1246', 'tein')}
 {('to_cook', 'keda1252-leuba', "iu 'daʔa"), ('to_cook', 'lama1277-lewuk', 'giu'), ('to_cook', 'lama1277-paina', 'giu'), ('to_cook', 'lama1277-lewop', 'giu'), ('to_cook', 'keda1252', 'iu'), ('to_cook', 'keda1252-leuba', 'iu'), ('to_cook', 'lama1277-imulo', 'giu'), ('to_cook', 'lama1277-lewot', 'giu'), ('to_cook', 'lama1277-lewok', 'giu'), ('to_cook', 'lama1277-minga', 'giu')}


Concept ‘gravel’ is different between Alorese and Lamaholot.

 Alorese innovated the following forms:
 {('gravel', 'blag1240-kulij', 'gədowar'), ('gravel', 'baka1276', "gə'dobar"), ('gravel', 'blag1240-nule', "gə'novar"), ('gravel', 'alor1247-munas', "g'lowar")}
 {('gravel', 'alor1247-besar', "ba'lɔfa")}
 Alorese shares the following forms with TAP languages:
 {('gravel', 'blag1240-kulij', 'gədowar'), ('gravel', 'baka1276', "gə'dobar"), ('gravel', 'blag1240-nule', "gə'novar"), ('gravel', 'alor1247-munas', "g'lowar")}
 {('gravel', 'alor1247-besar', "ba'lɔfa")}
 One or more Lamaholot dialects innovated forms not found in Alorese:
 {('gravel', 'lama1277-lewoi', 'kəˈlɛka'), ('gravel', 'lama1277-adona', 'kəˈlɛhɛk'), ('gravel', 'kula1280-lanto', 'gwalakɨkki'), ('gravel', 'lama1277-adona', 'kəˈlɛkɛk'), ('gravel', 'kula1280-lanto', '’gwala ’kək')}

  Just for clarity:
  There are also the following similarity classes shared between Alorese and LH.
  {('gravel', 'lama1277-kalik', 'ˈvatu vuˈrɛk'), ('gravel', 'lama1277-kalik', 'vuˈrɛs-a'), ('gravel', 'lama1277-lerek', 'ˈvurek'), ('gravel', 'alor1247-pandai', "watɔ 'karːik")}


Concept ‘foot’ is different between Alorese and Lamaholot.

 Alorese innovated the following forms:
 {('foot', 'alor1247-baran', "leiŋ ka'lumak")}
 One or more Lamaholot dialects innovated forms not found in Alorese:
 {('foot', 'lama1277-adona', 'ˈleikɜt tɜˈnɑpʔã')}
 {('foot', 'lama1277-lewoi', 'kɔan ˈleiŋ')}

  Just for clarity:
  There are also the following similarity classes shared between Alorese and LH.
  {('foot', 'lama1277-lewot', 'linaha'), ('foot', 'lama1277-lerek', 'li'), ('foot', 'lama1277-dulhi', 'leĩ'), ('foot', 'keda1252-leuba', 'lei'), ('foot', 'alor1247-besar', 'leiŋ'), ('foot', 'lama1277-minga', 'lijesa'), ('foot', 'lama1277-horow', 'leik'), ('foot', 'lama1277-lewok', 'liga'), ('foot', 'lama1277-lewog', 'lei'), ('foot', 'lama1277-bama', 'lei'), ('foot', 'lama1277-lamal', 'leite'), ('foot', 'lama1277-waiwa', 'lẽi'), ('foot', 'lama1277-watan', 'lei'), ('foot', 'lama1277-kalik', 'li'), ('foot', 'lama1277-lewob', 'lei'), ('foot', 'lama1277-baipi', 'lei'), ('foot', 'lama1277-wuake', 'leik'), ('foot', 'lama1277-lamak', 'leik'), ('foot', 'lama1277-waiba', 'lei'), ('foot', 'lama1277-mulan', 'lei'), ('foot', 'lama1277-lamah', 'leik'), ('foot', 'lama1277-lewom', 'lei'), ('foot', 'lama1277-ritae', 'lei'), ('foot', 'lama1277-lewop', 'leiha'), ('foot', 'lama1277-paina', 'lig(a)'), ('foot', 'lama1277-botun', 'lei'), ('foot', 'lama1277-tanju', 'lei'), ('foot', 'lama1277-belan', 'leik'), ('foot', 'lama1277-ileap', 'leik'), ('foot', 'alor1247-pandai', 'leiŋ'), ('foot', 'alor1247-munas', 'leiʔ '), ('foot', 'lama1277-lewuk', 'linəga'), ('foot', 'lama1277-imulo', 'linəsa'), ('foot', 'lama1277-kalik', 'liga'), ('foot', 'keda1252', 'lei'), ('foot', 'alor1247-munas', 'leiŋ'), ('foot', 'lama1277-lamat', 'lei'), ('foot', 'lama1277-lewoe', 'lei'), ('foot', 'lama1277-pukau', 'lei'), ('foot', 'lama1277-merde', 'leik'), ('foot', 'lama1277-kiwan', 'leik'), ('foot', 'keda1252-leuwa', 'læi')}


Concept ‘louse_clothing’ is different between Alorese and Lamaholot.

 Alorese innovated the following forms:
 {('louse_clothing', 'alor1247-munas', "knə'pɛr")}
 {('louse_clothing', 'alor1247-pandai', "mamə'lapuŋ")}
 {('louse_clothing', 'alor1247-besar', "aho ka'mela")}

  Just for clarity:
  There are also the following similarity classes shared between Alorese and LH.
  {('louse_clothing', 'keda1252-leuwa', 'utu'), ('louse_clothing', 'sika1262-maume', 'ʔut̪u'), ('louse_clothing', 'keda1252', 'utuʔ'), ('louse_clothing', 'p-mala1545-abvd', '*kutu'), ('louse_in_hair', 'alor1247-munas', 'kutɔ'), ('louse_clothing', 'buna1278-suai', 'katuma'), ('louse_clothing', 'lama1277-pukau', 'kuto'), ('louse_in_hair', 'lama1277-lewoi', 'ˈkuto'), ('louse_in_hair', 'laka1255', 'utu'), ('louse_clothing', 'lama1277-tanju', 'kuto'), ('louse_clothing', 'sika1262-hewa', 'ʔutu'), ('louse_clothing', 'lama1277-lerek', 'kətuˈmau'), ('louse_clothing', 'lama1277-lamah', 'kuto'), ('louse_clothing', 'lama1277-lewoe', 'uto'), ('louse_clothing', 'lama1277-lewok', 'kutoru'), ('louse_in_hair', 'keda1252-leuba', 'ʔutu'), ('louse_clothing', 'lama1277-kalik', 'kətuˈmaw'), ('louse_in_hair', 'alor1247-besar', 'kuto'), ('louse_clothing', 'lama1277-botun', 'kuto'), ('louse_clothing', 'lama1277-lewot', 'kutoja'), ('louse_clothing', 'p-aust1307-abvd', '*kuCu'), ('louse_clothing', 'p-cent2245-abvd', '*kutu'), ('louse_in_hair', 'tetu1246', 'ʔutu'), ('louse_clothing', 'lama1277-merde', 'kuto'), ('louse_clothing', 'buna1278-bobon', 'katuma'), ('louse_clothing', 'alor1247-baran', "ku't:ɔ"), ('louse_in_hair', 'lama1277-adona', 'ˈkutɔ'), ('louse_in_hair', 'lama1277-kalik', 'kuˈtɔr'), ('louse_clothing', 'lama1277-lewop', 'kutoji'), ('louse_clothing', 'lama1277-kalik', 'kutu'), ('louse_clothing', 'lama1277-horow', 'kuto'), ('louse_clothing', 'lama1277-waiwa', 'kuto'), ('louse_clothing', 'lama1277-watan', 'kuto'), ('louse_clothing', 'lama1277-belan', 'kuto'), ('louse_in_hair', 'mamb1306', 'ʔut'), ('louse_clothing', 'lama1277-ileap', 'kuto'), ('louse_clothing', 'sika1262-tanai', 'ʔutu'), ('louse_clothing', 'p-timo1261', '*kVt'), ('louse_clothing', 'lama1277-adona', 'keˈtumã'), ('louse_in_hair', 'kema1243', 'ʔutu'), ('louse_clothing', 'lama1277-lamat', 'uto'), ('louse_clothing', 'lama1277-lewom', 'kuto'), ('louse_clothing', 'lama1277-ritae', 'kuto'), ('louse_in_hair', 'lama1277-kalik', 'ˈkutu'), ('louse_clothing', 'lama1277-dulhi', 'kuto'), ('louse_clothing', 'lama1277-baipi', 'kuto'), ('louse_in_hair', 'lama1277-lerek', 'kuˈtor'), ('louse_clothing', 'lama1277-lewuk', 'kutu'), ('louse_clothing', 'lama1277-lewog', 'kuto'), ('louse_clothing', 'lama1277-lewoi', 'kəˈtuma'), ('louse_clothing', 'sika1262-hewa', 'ṳtu'), ('louse_clothing', 'lama1277-kiwan', 'kuto'), ('louse_in_hair', 'alor1247-pandai', 'kuto'), ('louse_clothing', 'lama1277-minga', 'kutoru'), ('louse_clothing', 'lama1277-waiba', 'kuto'), ('louse_clothing', 'lama1277-mulan', 'kuto'), ('louse_in_hair', 'p-mala1545-acd', '*kutu'), ('louse_clothing', 'lama1277-lewob', 'kuto'), ('louse_clothing', 'lama1277-lamal', 'kuto'), ('louse_in_hair', 'tetu1245-suai', 'utu'), ('louse_in_hair', 'tetu1245-vique', 'utu'), ('louse_clothing', 'lama1277-paina', 'kutoy'), ('louse_in_hair', 'sika1262-hewa', 'ʔutu'), ('louse_in_hair', 'tuku1254', 'kutu'), ('louse_in_hair', 'koto1251', 'hutu'), ('louse_clothing', 'lama1277-lamak', 'kuto'), ('louse_in_hair', 'idat1237', 'utu'), ('louse_clothing', 'lama1277-wuake', 'kuto'), ('louse_clothing', 'lama1277-imulo', 'kutor'), ('louse_clothing', 'lama1277-kalik', 'kətuˈma'), ('louse_clothing', 'lama1277-bama', 'kuto')}


Concept ‘flat’ is different between Alorese and Lamaholot.

 Alorese shares the following forms with other AN languanges, but not Lamaholot:
 {('flat', 'keda1252', 'napoʔ'), ('flat', 'alor1247-pandai', 'napuk'), ('flat', 'keda1252-leuba', "na'poʔ"), ('flat', 'alor1247-besar', "na'pukuŋ")}
 One or more Lamaholot dialects innovated forms not found in Alorese:
 {('flat', 'lama1277-paina', 'nədəkən'), ('flat', 'lama1277-kalik', 'lerek'), ('flat', 'lama1277-lamal', 'lere'), ('flat', 'lama1277-lewuk', 'lerek'), ('flat', 'lama1277-merde', 'lere'), ('flat', 'lama1277-lamat', 'lerʔẽ'), ('flat', 'lama1277-lamah', 'lerʔe'), ('flat', 'lama1277-lewop', 'lereken'), ('flat', 'lama1277-kalik', 'leˈrɛk'), ('flat', 'lama1277-mulan', 'lere'), ('flat', 'lama1277-belan', 'lere'), ('flat', 'lama1277-lerek', 'lereˈkən'), ('flat', 'lama1277-minga', 'lerek'), ('flat', 'lama1277-lerek', 'ɡənəˈkən'), ('flat', 'lama1277-lewok', 'lerek'), ('flat', 'lama1277-wuake', 'lere'), ('flat', 'lama1277-imulo', 'lerekən'), ('flat', 'lama1277-lewot', 'lerekən')}
 {('flat', 'lama1277-baipi', 'əbʔã'), ('flat', 'lama1277-lewog', 'əbʔã'), ('flat', 'lama1277-tanju', 'əbʔã'), ('flat', 'lama1277-bama', 'əbʔã'), ('flat', 'lama1277-horow', 'əbʔã'), ('flat', 'lama1277-kiwan', 'əbʔã'), ('flat', 'lama1277-botun', 'əbʔã'), ('flat', 'lama1277-ileap', 'nəpa'), ('flat', 'lama1277-adona', 'ˈmepe'), ('flat', 'lama1277-ritae', 'əbã'), ('flat', 'lama1277-lewoi', 'ˈəbaʔaŋ'), ('flat', 'sarr1247-adiab', "bi'ʔa"), ('flat', 'lama1277-lewom', 'əbʔã'), ('flat', 'lama1277-waiwa', 'əbʔã'), ('flat', 'lama1277-dulhi', 'əbʔã'), ('flat', 'lama1277-waiba', 'əbaʔ')}
 {('flat', 'lama1277-lewoe', 'mənete')}

  Just for clarity:
  There are also the following similarity classes shared between Alorese and LH.
  {('flat', 'mamb1306', 'rɛma'), ('flat', 'lama1277-lamak', 'hama'), ('flat', 'alor1247-baran', 'hama')}
  {('flat', 'kama1365', 'tatok'), ('flat', 'sika1262-hewa', 'dətu'), ('flat', 'laka1255', 'natɑr'), ('flat', 'alor1247-pandai', 'nadak'), ('flat', 'sika1262-hewa', 'rata'), ('flat', 'p-mala1545-acd', '*pa(n)tar'), ('flat', 'sika1262-tanai', 'dətu-ŋ'), ('flat', 'p-alor1249', '*tatok'), ('flat', 'p-timo1261', '*tatok'), ('flat', 'lama1277-pukau', 'rata'), ('flat', 'sika1262-tanai', 'desak'), ('flat', 'lama1277-watan', 'rata'), ('flat', 'p-mala1545-acd', '*dataR'), ('flat', 'maka1316', "te'tuɁu"), ('flat', 'p-east2519', '*tetok'), ('flat', 'lama1277-lewob', 'rata'), ('flat', 'alor1247-munas', 'nadak')}


Concept ‘how’ is different between Alorese and Lamaholot.

 Alorese innovated the following forms:
 {('how', 'alor1247-munas', "nəm'ga")}
 {('how', 'alor1247-pandai', "pai'naŋ")}
 One or more Lamaholot dialects innovated forms not found in Alorese:
 {('how', 'lama1277-kalik', 'nao ɡa'), ('how', 'lama1277-adona', 'nɜˈɡaku'), ('how', 'lama1277-lerek', 'naboɡa')}

  Just for clarity:
  There are also the following similarity classes shared between Alorese and LH.
  {('how', 'alor1247-munas', 'nəmən;ga'), ('how', 'lama1277-lewoi', 'naʔaŋ ˈɡaʔe'), ('how', 'alor1247-pandai', "naməŋ'ga"), ('how', 'alor1247-baran', "na'mɔnaŋga"), ('how', 'alor1247-besar', "namɔ'naŋga")}


Concept ‘night’ is different between Alorese and Lamaholot.

 Alorese shares the following forms with TAP languages:
 {('night', 'alor1247-munas', "mə'rːɛŋ"), ('night', 'wers1238-taram', "ma'leŋ"), ('night', 'wers1238-marit', "m'lɛng"), ('night', 'alor1247-baran', "ma'reŋ"), ('night', 'alor1247-besar', "ma'rɛŋ"), ('night', 'alor1247-pandai', "ma'rːeŋ")}
 One or more Lamaholot dialects innovated forms not found in Alorese:
 {('night', 'lama1277-mulan', 'rəma'), ('night', 'lama1277-lamah', 'rəma'), ('night', 'lama1277-dulhi', 'rəmã'), ('night', 'lama1277-botun', 'rəmʔã'), ('night', 'lama1277-horow', 'rəmã'), ('night', 'lama1277-belan', 'rəma'), ('night', 'lama1277-ileap', 'rəma'), ('night', 'lama1277-bama', 'rəma'), ('night', 'lama1277-lamal', 'remã'), ('night', 'lama1277-lamat', 'rəma'), ('night', 'lama1277-kiwan', 'rəmãʔ'), ('night', 'lama1277-lamak', 'rəmã'), ('night', 'lama1277-lewog', 'rəmã'), ('night', 'lama1277-waiwa', 'rəmã'), ('night', 'lama1277-lewoi', 'rəˈmaʔaŋ'), ('night', 'lama1277-adona', 'ˈrɜmaʔ'), ('night', 'lama1277-lewok', 'rəma'), ('night', 'lama1277-watan', 'rəmã'), ('night', 'lama1277-merde', 'rəma')}
 {('night', 'lama1277-wuake', 'baɁu')}
 {('night', 'lama1277-lewop', 'buk'), ('night', 'lama1277-kalik', 'buk'), ('night', 'lama1277-lerek', 'buˈkənən'), ('night', 'lama1277-paina', 'bukən'), ('night', 'lama1277-lewuk', 'buk'), ('night', 'lama1277-lewot', 'buk'), ('night', 'lama1277-imulo', 'buk'), ('night', 'lama1277-minga', 'bukɤ'), ('night', 'lama1277-kalik', 'bukən')}
 {('night', 'fata1247', 'kounɛ'), ('night', 'kafo1240', 'ɑkʊ:n'), ('night', 'kelo1247-hopte', "ʔa'ka:n"), ('night', 'rett1240', 'kakua'), ('night', 'lama1277-lewoe', 'ṳgũ'), ('night', 'blag1240-bama', 'ilkana'), ('night', 'blag1240-warsa', 'ilxana'), ('night', 'blag1240-bama', 'ilqana'), ('night', 'sarr1247-nule', 'iqaʔan'), ('night', 'rett1240', 'ʔil kakua'), ('night', 'blag1240-tuntu', 'ilqana'), ('night', 'rett1240', 'ilkakua'), ('night', 'abui1241-petle', "ak.'un"), ('night', 'abui1241-ulaga', 'akuni'), ('night', 'kaer1234', 'iqano'), ('night', 'nede1245', 'ilaqana'), ('night', 'kelo1247-bring', 'əkɑn')}
 {('night', 'lama1277-ritae', 'noko'), ('night', 'lama1277-lewob', 'nokõ'), ('night', 'lama1277-lewom', 'nokõ'), ('night', 'lama1277-waiba', 'nokõ'), ('night', 'lama1277-tanju', 'noko'), ('night', 'lama1277-pukau', 'noko'), ('night', 'lama1277-baipi', 'noko')}


Concept ‘to_suck’ is different between Alorese and Lamaholot.

 Alorese innovated the following forms:
 {('to_suck', 'alor1247-besar', 'hamuŋ')}
 {('to_suck', 'alor1247-munas', 'hiuʔ')}
 Alorese shares the following forms with other AN languanges, but not Lamaholot:
 {('to_suck', 'blag1240-bama', "ku'dumis"), ('to_suck', 'kula1280-lanto', 'duma'), ('to_suck', 'alor1247-pandai', "da'mːe"), ('to_suck', 'keda1252-leuwa', 'dumεʔ'), ('to_suck', 'blag1240-nule', "kə'numu"), ('to_suck', 'kafo1240', 'dumɑ'), ('to_suck', 'rett1240', 'kadumi'), ('to_suck', 'nede1245', 'duma'), ('to_suck', 'blag1240-tuntu', 'kudumis'), ('to_suck', 'blag1240-warsa', 'kudumis'), ('to_suck', 'pura1258', "ka'dumiŋ"), ('to_suck', 'lamm1241-westp', 'dimis'), ('to_suck', 'keda1252-leuba', "du'mɛʔ"), ('to_suck', 'baka1276', "kə'dumu"), ('to_suck', 'kula1280-lanto', '’dumua'), ('to_suck', 'hama1240', 'dum')}
 One or more Lamaholot dialects innovated forms not found in Alorese:
 {('to_suck', 'lama1277-lerek', 'məmiˈhəŋ')}
 {('to_suck', 'lama1277-lerek', 'məmˈhi')}
 {('to_suck', 'p-mala1545-acd', '*sepsep'), ('to_suck', 'p-aust1307-abvd', '*sepsep'), ('to_suck', 'lama1277-adona', 'ˈʔisɜp'), ('to_suck', 'p-mala1545-abvd', '*sepsep'), ('to_suck', 'p-mala1545-acd', '*qisep'), ('to_suck', 'lama1277-lewoi', 'ˈisɜ'), ('to_suck', 'p-mala1545-acd', '*hisep')}

  Just for clarity:
  There are also the following similarity classes shared between Alorese and LH.
  {('to_suck', 'lama1277-kiwan', 'tuhu'), ('to_suck', 'lama1277-lewuk', 'tusu'), ('to_suck', 'lama1277-waiba', 'tuho'), ('to_suck', 'lama1277-ileap', 'tuho'), ('to_suck', 'lama1277-waiwa', 'tuho'), ('to_suck', 'lama1277-lamah', 'tuho'), ('to_suck', 'lama1277-wuake', 'tuho'), ('to_suck', 'lama1277-kalik', 'tusu'), ('to_suck', 'lama1277-mulan', 'tuho'), ('to_suck', 'p-cent2245-abvd', '*susu'), ('to_suck', 'lama1277-lewoe', 'tuhu'), ('to_suck', 'lama1277-lewok', 'tusu'), ('to_suck', 'lama1277-lamat', 'tuhu'), ('to_suck', 'lama1277-lewog', 'tuho'), ('to_suck', 'lama1277-lewot', 'tuho'), ('to_suck', 'lama1277-lamak', 'tuho'), ('to_suck', 'lama1277-lewom', 'tuho'), ('to_suck', 'lama1277-ritae', 'tuho'), ('to_suck', 'lama1277-dulhi', 'tuhu'), ('to_suck', 'lama1277-watan', 'tuho'), ('to_suck', 'lama1277-belan', 'tuo'), ('to_suck', 'alor1247-besar', 'dumɔ'), ('to_suck', 'lama1277-lewob', 'tuho'), ('to_suck', 'lama1277-lewop', 'tuhu'), ('to_suck', 'lama1277-imulo', 'tus'), ('to_suck', 'lama1277-horow', 'tuho'), ('to_suck', 'blag1240-kulij', 'dusaŋ'), ('to_suck', 'lama1277-merde', 'tuho'), ('to_suck', 'lama1277-lamal', 'tu (ana)'), ('to_suck', 'lama1277-kalik', 'ˈdusəp'), ('to_suck', 'lama1277-baipi', 'tuho'), ('to_suck', 'lama1277-botun', 'tuho'), ('to_suck', 'keda1252', 'tu'), ('to_suck', 'lama1277-minga', 'tuso'), ('to_suck', 'lama1277-bama', 'tuho'), ('to_suck', 'lama1277-paina', 'tuhuŋ'), ('to_suck', 'keda1252-leuwa', 'dupεŋ'), ('to_suck', 'lama1277-tanju', 'tuho'), ('to_suck', 'lama1277-pukau', 'tuho'), ('to_suck', 'sika1262-hewa', 'wuhu'), ('to_suck', 'keda1252-leuwa', 'duhu'), ('to_suck', 'alor1247-pandai', 'dumɔ'), ('to_suck', 'alor1247-baran', 'dum:ɔ')}


Concept ‘to_play’ is different between Alorese and Lamaholot.

 One or more Lamaholot dialects innovated forms not found in Alorese:
 {('to_play', 'lama1277-lewoe', 'pule')}
 {('to_play', 'lama1277-merde', 'raʔa'), ('to_play', 'lama1277-lamat', 'urã')}
 {('to_play', 'lama1277-kalik', 'ˈglasa'), ('to_play', 'lama1277-kalik', 'gəlasa'), ('to_play', 'lamm1241-westp', 'ikaluŋ'), ('to_play', 'lama1277-dulhi', 'gəlaha'), ('to_play', 'lama1277-watan', 'gəlaha'), ('to_play', 'kafo1240', 'kɪrɑ'), ('to_play', 'lama1277-botun', 'gəlaha'), ('to_play', 'lama1277-imulo', 'gəlaŋ'), ('to_play', 'lama1277-waiwa', 'gəlaha'), ('to_play', 'lama1277-wuake', 'gəlaha'), ('to_play', 'lama1277-lewok', 'gəlasa'), ('to_play', 'lama1277-horow', 'gəlaha'), ('to_play', 'lama1277-mulan', 'gəlaha'), ('to_play', 'lama1277-belan', 'gəla'), ('to_play', 'lama1277-kiwan', 'gəlaha'), ('to_play', 'lama1277-lewuk', 'gəlasa'), ('to_play', 'lama1277-paina', 'kəraŋa'), ('to_play', 'lama1277-lewot', 'gəlah'), ('to_play', 'lama1277-adona', 'ɡ(ɜ)ˈlahaʔ'), ('to_play', 'lama1277-lewop', 'gəlahaŋ'), ('to_play', 'lama1277-lerek', 'kəˈraŋa'), ('to_play', 'lama1277-lamak', 'gəlaha'), ('to_play', 'lama1277-lamal', 'gəla'), ('to_play', 'lama1277-lamah', 'gəlaha')}
 {('to_play', 'lama1277-ileap', 'irek')}
 {('to_play', 'lama1277-pukau', 'vəŋa')}

  Just for clarity:
  There are also the following similarity classes shared between Alorese and LH.
  {('to_play', 'lama1277-bama', 'gənəku'), ('to_play', 'lama1277-baipi', 'gənəku'), ('to_play', 'alor1247-pandai', "k'nakːu"), ('to_play', 'alor1247-munas', "knə'ku"), ('to_play', 'lama1277-ritae', 'gənəkuk'), ('to_play', 'alor1247-besar', "ka'naku"), ('to_play', 'lama1277-lewog', 'gənəku'), ('to_play', 'lama1277-lewom', 'gənəku'), ('to_play', 'lama1277-tanju', 'gənəku'), ('to_play', 'lama1277-minga', 'kənebo'), ('to_play', 'lama1277-lewoi', 'ɡənəˈku'), ('to_play', 'alor1247-baran', "ka'nɔku"), ('to_play', 'lama1277-waiba', 'gənəku'), ('to_play', 'lama1277-lewob', 'məŋə')}


Concept ‘slave’ is different between Alorese and Lamaholot.

 Alorese innovated the following forms:
 {('slave', 'alor1247-pandai', "k'rumoŋ "), ('slave', 'alor1247-besar', "ma'nɔle"), ('slave', 'alor1247-pandai', "k'rumoŋ 'mənɔle"), ('slave', 'alor1247-pandai', 'mənɔle'), ('slave', 'alor1247-besar', "ka'rumoŋ ")}
 {('slave', 'alor1247-munas', "b'lɛnta ")}
 One or more Lamaholot dialects innovated forms not found in Alorese:
 {('slave', 'keda1252-leuba', "ʔu'nan"), ('slave', 'lama1277-lewoi', 'ˈkunaŋ')}
 {('slave', 'lama1277-adona', 'ˈʔariˈʔanaʔ')}
 {('slave', 'lama1277-lerek', 'ˈdʒ͡ɔŋɔs')}
 {('slave', 'lama1277-kalik', 'sənuˈdaŋ')}


Concept ‘to_squeeze’ is different between Alorese and Lamaholot.

 Alorese shares the following forms with other AN languanges, but not Lamaholot:
 {('to_squeeze', 'alor1247-pandai', 'bulɛ'), ('to_squeeze', 'keda1252', 'buleŋ')}
 {('to_squeeze', 'blag1240-bama', 'pemex'), ('to_squeeze', 'sika1262-maume', 'pəme'), ('to_squeeze', 'blag1240-nule', 'pemek'), ('to_squeeze', 'alor1247-pandai', 'pɛmɛ'), ('to_squeeze', 'alor1247-besar', 'pɛmɛ'), ('to_squeeze', 'alor1247-munas', 'pɛmɛʔ')}
 One or more Lamaholot dialects innovated forms not found in Alorese:
 {('to_squeeze', 'lama1277-tanju', 'ikut'), ('to_squeeze', 'lama1277-baipi', 'ikut'), ('to_squeeze', 'keda1252-leuba', 'ikuʔ'), ('to_squeeze', 'lama1277-minga', 'gəmikut'), ('to_squeeze', 'lama1277-lamak', 'iku'), ('to_squeeze', 'lama1277-lewop', 'ikut'), ('to_squeeze', 'lama1277-lewom', 'ikut')}
 {('to_squeeze', 'kula1280-lanto', 'la’mas'), ('to_squeeze', 'p-mala1545-acd', '*mesmes'), ('to_squeeze', 'p-mala1545-acd', '*Rames'), ('to_squeeze', 'kula1280-lanto', 'laməs'), ('to_squeeze', 'kula1280-lanto', 'lamis'), ('to_squeeze', 'p-mala1545-acd', '*kemes'), ('to_squeeze', 'lama1277-watan', 'pərəs'), ('to_squeeze', 'p-cent2245-abvd', '*ʀaməs'), ('to_squeeze', 'wers1238-taram', 'lumusi')}

  Just for clarity:
  There are also the following similarity classes shared between Alorese and LH.
  {('to_squeeze', 'lama1277-belan', 'piuk'), ('to_squeeze', 'lama1277-lerek', 'ˈpiuŋ'), ('to_squeeze', 'lama1277-kiwan', 'piʔuk'), ('to_squeeze', 'lama1277-ritae', 'piʔuk'), ('to_squeeze', 'lama1277-paina', 'piuŋ'), ('to_squeeze', 'lama1277-lewoi', 'ˈpiʔu'), ('to_squeeze', 'lama1277-mulan', 'piu'), ('to_squeeze', 'lama1277-bama', 'piʔuk'), ('to_squeeze', 'lama1277-lamal', 'piuk'), ('to_squeeze', 'lama1277-adona', 'ˈpiʔuk'), ('to_squeeze', 'lama1277-lewok', 'piuk'), ('to_squeeze', 'lama1277-merde', 'piuk'), ('to_squeeze', 'lama1277-kalik', 'ˈpiuk'), ('to_squeeze', 'lama1277-ileap', 'piuk'), ('to_squeeze', 'lama1277-lewob', 'piʔu'), ('to_squeeze', 'lama1277-lewuk', 'piuk'), ('to_squeeze', 'lama1277-lamat', 'piuʔ'), ('to_squeeze', 'lama1277-wuake', 'piɁuk'), ('to_squeeze', 'baka1276', 'paha'), ('to_squeeze', 'lama1277-lewot', 'piuk'), ('to_squeeze', 'lama1277-pukau', 'piʔuk'), ('to_squeeze', 'lama1277-dulhi', 'piʔuk'), ('to_squeeze', 'lama1277-imulo', 'piuka'), ('to_squeeze', 'lama1277-waiba', 'piʔuk'), ('to_squeeze', 'rett1240', 'paha'), ('to_squeeze', 'lama1277-kalik', 'piuk'), ('to_squeeze', 'atim1239', 'ajak'), ('to_squeeze', 'lama1277-lewog', 'piʔu'), ('to_squeeze', 'lama1277-horow', 'piʔuk'), ('to_squeeze', 'lama1277-waiwa', 'piʔuk'), ('to_squeeze', 'lama1277-lewoe', 'piu'), ('to_squeeze', 'lama1277-lamah', 'piuk'), ('to_squeeze', 'blag1240-kulij', 'paha'), ('to_squeeze', 'lama1277-botun', 'peʔut'), ('to_squeeze', 'alor1247-besar', 'pihi')}


Concept ‘blunt’ is different between Alorese and Lamaholot.

 Alorese innovated the following forms:
 {('blunt', 'alor1247-pandai', 'punak')}
 Alorese shares the following forms with other AN languanges, but not Lamaholot:
 {('blunt', 'kema1243', 'bo:n'), ('blunt', 'buna1278-bobon', 'bou'), ('blunt', 'buna1278-malia', 'bou'), ('blunt', 'sika1262-tanai', 'bou-ŋ'), ('blunt', 'buna1278-suai', 'bou'), ('blunt', 'alor1247-munas', 'pouŋ'), ('blunt', 'mamb1306', 'bou')}
 {('blunt', 'alor1247-pandai', 'kumbuk'), ('blunt', 'blag1240-nule', 'kumbul'), ('blunt', 'blag1240-kulij', 'kumbul'), ('blunt', 'p-cent2245-abvd', '*dumpul'), ('blunt', 'p-mala1545-acd', '*dumpul'), ('blunt', 'p-mala1545-acd', '*tumpel'), ('blunt', 'alor1247-baran', 'kumbu'), ('blunt', 'p-mala1545-abvd', '*dumpul'), ('blunt', 'alor1247-besar', 'kumbu'), ('blunt', 'baka1276', 'kumbul'), ('blunt', 'p-mala1545-acd', '*tumpul')}
 One or more Lamaholot dialects innovated forms not found in Alorese:
 {('blunt', 'lama1277-tanju', 'dat'), ('blunt', 'lama1277-lewog', 'dã'), ('blunt', 'nede1245', 'it:a'), ('blunt', 'lama1277-pukau', 'da'), ('blunt', 'lama1277-lewoi', 'daː'), ('blunt', 'lama1277-bama', 'dat'), ('blunt', 'lama1277-waiba', 'dāt')}
 {('blunt', 'lama1277-lewoe', 'milu'), ('blunt', 'lama1277-lamat', 'milu')}
 {('blunt', 'sika1262-hewa', 'bohuk'), ('blunt', 'lama1277-horow', 'kəbusu'), ('blunt', 'sika1262-maume', 'bohok'), ('blunt', 'sika1262-hewa', 'bohu'), ('blunt', 'lama1277-lewob', 'gəbohu')}
 {('blunt', 'lama1277-lewot', 'motəl'), ('blunt', 'lama1277-lewop', 'motəl'), ('blunt', 'tuku1254', "kɔ'mɔte"), ('blunt', 'sika1262-tanai', 'mumuk'), ('blunt', 'keda1252', 'mamel'), ('blunt', 'lama1277-lerek', 'kəmoˈtəl'), ('blunt', 'lama1277-minga', 'motəl'), ('blunt', 'lama1277-wuake', 'motel'), ('blunt', 'lama1277-imulo', 'motəl'), ('blunt', 'lama1277-mulan', 'kəmotol')}
 {('blunt', 'lama1277-kiwan', 'gã hala'), ('blunt', 'lama1277-ileap', 'dəkət hala'), ('blunt', 'lama1277-waiwa', 'gãhala'), ('blunt', 'lama1277-watan', 'gã hala'), ('blunt', 'lama1277-baipi', 'gãhala'), ('blunt', 'lama1277-botun', 'gã hala'), ('blunt', 'lama1277-lewom', 'gãhala')}
 {('blunt', 'lama1277-lewuk', 'mosok'), ('blunt', 'lama1277-dulhi', 'kəmoŋo'), ('blunt', 'lama1277-lewok', 'kəmoso'), ('blunt', 'lama1277-merde', 'kəmoŋo'), ('blunt', 'lama1277-lamak', 'kələŋo'), ('blunt', 'lama1277-kalik', 'kəmoson'), ('blunt', 'lama1277-lerek', 'kəmoˈhok'), ('blunt', 'lama1277-lamah', 'moŋo'), ('blunt', 'lama1277-adona', 'kɜˈmɔŋɔ'), ('blunt', 'lama1277-paina', 'kəmohokə'), ('blunt', 'lama1277-ritae', 'kəwiŋo'), ('blunt', 'lama1277-belan', 'moso'), ('blunt', 'lama1277-kalik', 'kəmɔˈsɔn'), ('blunt', 'lama1277-lamal', 'moso')}


Concept ‘to_dance’ is different between Alorese and Lamaholot.

 Alorese innovated the following forms:
 {('to_dance', 'rett1240', 'sara sumba'), ('to_dance', 'pura1258', 'sara sumba'), ('to_dance', 'alor1247-besar', "sar'sumba"), ('to_dance', 'blag1240-warsa', 'sara sumba'), ('to_dance', 'blag1240-kulij', 'sarosumba'), ('to_dance', 'blag1240-nule', 'sarsumba')}
 {('to_dance', 'kaer1234', 'roŋgeŋ'), ('to_dance', 'alor1247-pandai', "rɔŋ'ge")}
 {('to_dance', 'alor1247-baran', 'tam:ɔ')}
 Alorese shares the following forms with TAP languages:
 {('to_dance', 'rett1240', 'sara sumba'), ('to_dance', 'pura1258', 'sara sumba'), ('to_dance', 'alor1247-besar', "sar'sumba"), ('to_dance', 'blag1240-warsa', 'sara sumba'), ('to_dance', 'blag1240-kulij', 'sarosumba'), ('to_dance', 'blag1240-nule', 'sarsumba')}
 {('to_dance', 'kaer1234', 'roŋgeŋ'), ('to_dance', 'alor1247-pandai', "rɔŋ'ge")}
 {('to_dance', 'alor1247-baran', 'tam:ɔ')}
 One or more Lamaholot dialects innovated forms not found in Alorese:
 {('to_dance', 'lama1277-paina', 'hedu'), ('to_dance', 'mamb1306', 'hudi'), ('to_dance', 'lama1277-lerek', 'hɛˈdu'), ('to_dance', 'abui1241-ulaga', 'soyaŋ'), ('to_dance', 'keda1252-leuba', "he'duŋ"), ('to_dance', 'kafo1240', 'sʊʤɑŋ')}
 {('to_dance', 'lama1277-adona', 'ˈniːɡɜ'), ('to_dance', 'adan1251-lawah', 'neikøli:'), ('to_dance', 'lama1277-horow', 'nigə'), ('to_dance', 'lama1277-kiwan', 'nigə')}
 {('to_dance', 'lama1277-dulhi', 'enən')}

  Just for clarity:
  There are also the following similarity classes shared between Alorese and LH.
  {('to_dance', 'lama1277-lewog', 'soka'), ('to_dance', 'sarr1247-nule', 'sohai '), ('to_dance', 'lama1277-lewoi', 'ˈsoka'), ('to_dance', 'lama1277-pukau', 'soka'), ('to_dance', 'sarr1247-adiab', 'sohal '), ('to_dance', 'lama1277-lewok', 'soka'), ('to_dance', 'lama1277-lewom', 'soka'), ('to_dance', 'lama1277-ritae', 'soka'), ('to_dance', 'blag1240-tuntu', 'sokil'), ('to_dance', 'blag1240-warsa', 'sokil'), ('to_dance', 'lama1277-lewop', 'soka'), ('to_dance', 'lama1277-lamah', 'soka'), ('to_dance', 'lama1277-waiwa', 'soka'), ('to_dance', 'lama1277-wuake', 'soka'), ('to_dance', 'lama1277-baipi', 'soka'), ('to_dance', 'lama1277-bama', 'soka'), ('to_dance', 'buna1278-bobon', 'soe'), ('to_dance', 'lama1277-mulan', 'soka'), ('to_dance', 'lama1277-watan', 'soka'), ('to_dance', 'lama1277-imulo', 'soka'), ('to_dance', 'alor1247-munas', 'tɔdʒa'), ('to_dance', 'lama1277-lewot', 'soka'), ('to_dance', 'lama1277-tanju', 'soka'), ('to_dance', 'sika1262-hewa', 'soka'), ('to_dance', 'lama1277-merde', 'soka'), ('to_dance', 'lama1277-lamal', 'soka'), ('to_dance', 'lama1277-lewob', 'soka'), ('to_dance', 'lama1277-waiba', 'soka'), ('to_dance', 'lama1277-botun', 'soka'), ('to_dance', 'keda1252', 'soka'), ('to_dance', 'lama1277-ileap', 'soka'), ('to_dance', 'teiw1235', 'soxai'), ('to_dance', 'lama1277-lewuk', 'soka'), ('to_dance', 'kaer1234', 'soqai'), ('to_dance', 'nede1245', 'soi'), ('to_dance', 'blag1240-bama', 'sokil'), ('to_dance', 'lama1277-lewoe', 'sokə'), ('to_dance', 'lama1277-minga', 'soka'), ('to_dance', 'lama1277-lamat', 'sokə'), ('to_dance', 'lama1277-kalik', 'ˈsoka'), ('to_dance', 'lama1277-belan', 'soka'), ('to_dance', 'lama1277-kalik', 'soka'), ('to_dance', 'lama1277-lamak', 'soka')}


Concept ‘butterfly’ is different between Alorese and Lamaholot.

 Alorese innovated the following forms:
 {('butterfly', 'alor1247-munas', "krə'bɛŋ")}
 {('butterfly', 'alor1247-besar', "fulu-'fulu")}
 {('butterfly', 'baka1276', 'kaliliŋ'), ('butterfly', 'blag1240-nule', 'kaililiŋ'), ('butterfly', 'blag1240-kulij', 'kaliliŋ'), ('butterfly', 'blag1240-tuntu', 'kaliliŋ'), ('butterfly', 'alor1247-pandai', "kakə'lili")}
 Alorese shares the following forms with TAP languages:
 {('butterfly', 'alor1247-munas', "krə'bɛŋ")}
 {('butterfly', 'alor1247-besar', "fulu-'fulu")}
 {('butterfly', 'baka1276', 'kaliliŋ'), ('butterfly', 'blag1240-nule', 'kaililiŋ'), ('butterfly', 'blag1240-kulij', 'kaliliŋ'), ('butterfly', 'blag1240-tuntu', 'kaliliŋ'), ('butterfly', 'alor1247-pandai', "kakə'lili")}
 One or more Lamaholot dialects innovated forms not found in Alorese:
 {('butterfly', 'lama1277-lerek', 'kəbuˈkal'), ('butterfly', 'lama1277-adona', 'kɜˈbukare'), ('butterfly', 'lama1277-lewoi', 'bukaˈlaka'), ('butterfly', 'keda1252-leuba', 'ʔebal'), ('butterfly', 'lama1277-kalik', 'kəbuˈkal')}


Concept ‘to_cut_down’ is different between Alorese and Lamaholot.

 Alorese innovated the following forms:
 {('to_cut_down', 'alor1247-pandai', 'hɔdaŋ')}
 {('to_cut_down', 'alor1247-besar', 'lɔlɔ')}
 {('to_cut_down', 'alor1247-pandai', "ta'rːi"), ('to_cut_down', 'alor1247-munas', "tə'rːi")}
 One or more Lamaholot dialects innovated forms not found in Alorese:
 {('to_cut_down', 'lama1277-kalik', 'baˈkat'), ('to_cut_down', 'lama1277-lerek', 'bakəˈtəŋ')}
 {('to_cut_down', 'lama1277-lewoi', 'ˈɡəto')}
 {('to_cut_down', 'lama1277-adona', 'ˈhobat'), ('to_cut_down', 'lama1277-kalik', 'ˈtoba')}


Concept ‘to_spit’ is different between Alorese and Lamaholot.

 Alorese innovated the following forms:
 {('to_spit', 'alor1247-pandai', "pitɔŋ 'ilu"), ('to_spit', 'alor1247-munas', 'pitɔŋ'), ('to_spit', 'alor1247-pandai', 'pitɔŋ')}
 {('to_spit', 'alor1247-besar', 'uli')}
 {('to_spit', 'alor1247-baran', "buh:u 'ill:u"), ('to_spit', 'alor1247-pandai', 'buhu')}
 One or more Lamaholot dialects innovated forms not found in Alorese:
 {('to_spit', 'abui1241-ulaga', 'tipuoŋ'), ('to_spit', 'lamm1241-westp', 'tup:uŋ'), ('to_spit', 'lama1277-dulhi', 'pərino'), ('to_spit', 'p-alor1249', '*purVn'), ('to_spit', 'kira1248', 'upuran'), ('to_spit', 'lama1277-watan', 'pərino'), ('to_spit', 'blag1240-tuntu', 'puruŋ'), ('to_spit', 'kaer1234', "pu'raŋ"), ('to_spit', 'maka1316', "ulu'ira"), ('to_spit', 'kuii1253', 'puriŋ'), ('to_spit', 'abui1241-takal', "pu.'jü"), ('to_spit', 'lama1277-lewoe', 'piru'), ('to_spit', 'lama1277-pukau', 'pino'), ('to_spit', 'blag1240-nule', 'puruŋ'), ('to_spit', 'baka1276', 'puruŋ'), ('to_spit', 'teiw1235', "pu'ran"), ('to_spit', 'blag1240-kulij', 'puruŋ'), ('to_spit', 'lama1277-mulan', 'pərino'), ('to_spit', 'lama1277-kiwan', 'pərino'), ('to_spit', 'idat1237', "bu'rit"), ('to_spit', 'lama1277-lamak', 'pərino'), ('to_spit', 'lama1277-lewom', 'pərino'), ('to_spit', 'lama1277-ritae', 'pərino'), ('to_spit', 'blag1240-bama', 'puruŋ'), ('to_spit', 'blag1240-warsa', 'puruŋ'), ('to_spit', 'p-east2519', '*fulu(k, n)'), ('to_spit', 'fata1247', 'fulun'), ('to_spit', 'lama1277-waiba', 'pərino'), ('to_spit', 'p-timo1261', '*puRV(n)'), ('to_spit', 'koto1251', 'na-rono'), ('to_spit', 'lama1277-merde', 'pərino'), ('to_spit', 'pura1258', 'puruŋ'), ('to_spit', 'kelo1247-bring', 'pərʊin'), ('to_spit', 'lama1277-botun', 'pərino'), ('to_spit', 'lama1277-ileap', 'pino'), ('to_spit', 'lama1277-horow', 'pərino'), ('to_spit', 'lama1277-waiwa', 'pərino'), ('to_spit', 'atim1239', 'puina'), ('to_spit', 'lama1277-baipi', 'pərino'), ('to_spit', 'lama1277-bama', 'pərino'), ('to_spit', 'lama1277-lamah', 'prino'), ('to_spit', 'lama1277-adona', 'ˈprino'), ('to_spit', 'rett1240', 'puruŋ'), ('to_spit', 'lama1277-lewoi', 'pəˈrino'), ('to_spit', 'kabo1247', 'paraŋ puju'), ('to_spit', 'lama1277-lewog', 'pərino'), ('to_spit', 'lama1277-lewob', 'pino')}
 {('to_spit', 'lama1277-lerek', 'təˈmidʒ͡ɔ'), ('to_spit', 'lama1277-lewuk', 'təmiju'), ('to_spit', 'lama1277-wuake', 'təmijo'), ('to_spit', 'lama1277-lewok', 'təmiju'), ('to_spit', 'lama1277-minga', 'təmiju'), ('to_spit', 'lama1277-belan', 'təmijo'), ('to_spit', 'lama1277-kalik', 'təˈmiʤu'), ('to_spit', 'lama1277-lewot', 'təmiju'), ('to_spit', 'lama1277-lewop', 'təmiju'), ('to_spit', 'adan1251-lawah', 'atamaj'), ('to_spit', 'lama1277-lamal', 'təmiro'), ('to_spit', 'lama1277-paina', 'təmij'), ('to_spit', 'lama1277-kalik', 'təmiju'), ('to_spit', 'lama1277-imulo', 'təmi')}
 {('to_spit', 'keda1252-leuwa', 'mijuʔ'), ('to_spit', 'keda1252-leuwa', 'pɔtaʔ mijuʔ'), ('to_spit', 'lama1277-tanju', 'putã'), ('to_spit', 'keda1252-leuwa', 'i̘juʔ'), ('to_spit', 'keda1252-leuba', "pɔ'taʔ"), ('to_spit', 'keda1252', 'potaʔ'), ('to_spit', 'sika1262-tanai', 'bota-ŋ')}

  Just for clarity:
  There are also the following similarity classes shared between Alorese and LH.
  {('to_spit', 'alor1247-munas', 'ilu'), ('to_spit', 'sika1262-tanai', 'ilur'), ('to_spit', 'sika1262-maume', 'niru'), ('to_spit', 'sika1262-hewa', 'niru'), ('to_spit', 'sika1262-tanai', 'nilur'), ('to_spit', 'lama1277-lamat', 'piru')}


Concept ‘garden’ is different between Alorese and Lamaholot.

 One or more Lamaholot dialects innovated forms not found in Alorese:
 {('garden', 'lama1277-adona', 'mãː'), ('garden', 'sika1262-hewa', 'uma'), ('garden', 'maka1316', 'ama'), ('garden', 'lama1277-lewoi', 'maːŋ')}
 {('garden', 'lama1277-lerek', 'ˈoleŋ')}

  Just for clarity:
  There are also the following similarity classes shared between Alorese and LH.
  {('garden', 'lama1277-kalik', 'ekan'), ('garden', 'alor1247-munas', 'ɛkaŋ '), ('garden', 'lama1277-kalik', 'eka'), ('garden', 'alor1247-pandai', 'ɛkaŋ '), ('garden', 'alor1247-munas', "ɛkaŋ 'butaʔ"), ('garden', 'alor1247-besar', 'ɛkaŋ ')}


Concept ‘scared’ is different between Alorese and Lamaholot.

 One or more Lamaholot dialects innovated forms not found in Alorese:
 {('scared', 'lama1277-kalik', 'têmêkon')}
 {('scared', 'lama1277-lamak', 'soʔo'), ('scared', 'lama1277-dulhi', 'soʔot'), ('scared', 'lama1277-kiwan', 'soʔot'), ('scared', 'lama1277-horow', 'soʔot'), ('scared', 'lama1277-waiwa', 'soʔot'), ('scared', 'lama1277-tanju', 'soʔot'), ('scared', 'lama1277-watan', 'soʔo'), ('scared', 'lama1277-ileap', 'soʔot'), ('scared', 'lama1277-adona', 'ˈsoʔot'), ('scared', 'lama1277-ritae', 'soʔot'), ('scared', 'lama1277-botun', 'soʔot')}
 {('scared', 'lama1277-lewoe', 'goʔə')}
 {('scared', 'lama1277-wuake', 'kərui'), ('scared', 'lama1277-imulo', 'kəruitəs'), ('scared', 'lama1277-minga', 'kərui'), ('scared', 'lama1277-lewot', 'kəruit'), ('scared', 'lama1277-lamal', 'kəruit'), ('scared', 'lama1277-belan', 'kərui'), ('scared', 'lama1277-lewop', 'keruit')}

  Just for clarity:
  There are also the following similarity classes shared between Alorese and LH.
  {('scared', 'alor1247-munas', 'takuʔ'), ('scared', 'lama1277-kalik', 'takut'), ('scared', 'maka1316', 'agaɁaga'), ('scared', 'lama1277-lewob', 'takuʔ'), ('scared', 'tetu1246', 'tɑuk'), ('scared', 'lama1277-lamat', 'taʔu'), ('scared', 'lama1277-lewok', 'takutga'), ('scared', 'lama1277-lamah', 'takut'), ('scared', 'kema1243', "ta'maʔut"), ('scared', 'lama1277-lewog', 'taku'), ('scared', 'lama1277-lewoi', 'ˈtaku'), ('scared', 'lama1277-bama', 'taku'), ('scared', 'p-aust1307-abvd', '*ma-takut'), ('scared', 'keda1252', 'taiʔ'), ('scared', 'lama1277-pukau', 'tapuk'), ('scared', 'alor1247-besar', 'taku'), ('scared', 'lama1277-baipi', 'takut'), ('scared', 'alor1247-pandai', 'takuk'), ('scared', 'lama1277-lewuk', 'takut'), ('scared', 'tetu1245-vique', 'taɁuk'), ('scared', 'lama1277-merde', 'takut'), ('scared', 'lama1277-mulan', 'taku'), ('scared', 'p-mala1545-acd', '*takut'), ('scared', 'lama1277-paina', 'takut'), ('scared', 'lama1277-lewom', 'takut'), ('scared', 'laka1255', "ta'ʔuk"), ('scared', 'lama1277-lerek', 'təˈnakutən'), ('scared', 'tuku1254', "ma'taku"), ('scared', 'alor1247-baran', 'taku'), ('scared', 'keda1252-leuba', 'tauʔ'), ('scared', 'tetu1245-suai', 'hataɁuk'), ('scared', 'nede1245', 'teʔi'), ('scared', 'lama1277-waiba', 'takut')}


Concept ‘wave’ is different between Alorese and Lamaholot.

 Alorese innovated the following forms:
 {('wave', 'alor1247-pandai', "bə'notɛŋ")}
 {('wave', 'alor1247-besar', 'umba'), ('wave', 'alor1247-munas', 'ɔmbaʔ')}
 {('wave', 'alor1247-munas', "tahi k'lakɔl")}
 One or more Lamaholot dialects innovated forms not found in Alorese:
 {('wave', 'lama1277-adona', "mɜ'naʔlũː")}
 {('wave', 'blag1240-warsa', 'kolaŋ'), ('wave', 'rett1240', 'ko:laŋ'), ('wave', 'lama1277-lerek', 'ˈknolhan'), ('wave', 'p-mala1545-acd', '*qalun')}

  Just for clarity:
  There are also the following similarity classes shared between Alorese and LH.
  {('wave', 'lama1277-kalik', 'ɔˈʤɔk'), ('wave', 'alor1247-pandai', 'ɔdʒɔ'), ('wave', 'lama1277-lewoi', 'ojo'), ('wave', 'alor1247-besar', 'ɔʤɔ'), ('wave', 'alor1247-munas', 'ɔdʒɔʔ')}


Concept ‘here’ is different between Alorese and Lamaholot.

 Alorese innovated the following forms:
 {('here', 'alor1247-baran', "hanʤa'fa")}
 {('here', 'alor1247-besar', "ha 'ɔnɔŋ")}
 {('here', 'alor1247-pandai', "ɔrɔ 'kia"), ('here', 'alor1247-pandai', 'ɔrkɛ'), ('here', 'alor1247-munas', "r'kɛ"), ('here', 'alor1247-pandai', "ɔrɔ 'kɛ")}
 One or more Lamaholot dialects innovated forms not found in Alorese:
 {('here', 'lama1277-pukau', 'ite'), ('here', 'sika1262-hewa', 'muʔe ʔete'), ('here', 'sika1262-hewa', 'muʔe'), ('here', 'sika1262-hewa', 'ete')}
 {('here', 'p-mala1545-acd', '*ia'), ('here', 'p-mala1545-acd', '*ia-ni'), ('here', 'lama1277-lewoe', 'noʔo'), ('here', 'keda1252-leuwa', 'di nɔʔ'), ('here', 'lama1277-dulhi', 'siniʔ'), ('here', 'laka1255', "deʔi 'nia"), ('here', 'keda1252', 'dinoʔo'), ('here', 'p-mala1545-acd', '*i-ni'), ('here', 'keda1252-leuba', "diʔ 'nɔʔɔ"), ('here', 'idat1237', "hɛ'nia"), ('here', 'kema1243', ",dia 'nɔgɔ")}
 {('here', 'lama1277-botun', 'dəpi'), ('here', 'lama1277-horow', 'təpi'), ('here', 'lama1277-adona', 'dɜˈhirɜ'), ('here', 'lama1277-ritae', 'dipi'), ('here', 'lama1277-lamal', 'dipi'), ('here', 'lama1277-ileap', 'təpi'), ('here', 'lama1277-waiba', 'təpi'), ('here', 'lama1277-lewom', 'tiʔi'), ('here', 'lama1277-mulan', 'dipi'), ('here', 'lama1277-lewop', 'kətəri'), ('here', 'lama1277-lewot', 'keteri'), ('here', 'lama1277-watan', 'dipi'), ('here', 'lama1277-lamah', 'təpi'), ('here', 'lama1277-lewob', 'pi'), ('here', 'mamb1306', "nei 'rɑi"), ('here', 'lama1277-kiwan', 'pəhia'), ('here', 'lama1277-bama', 'pia'), ('here', 'lama1277-wuake', 'dəpi'), ('here', 'lama1277-lamat', 'dabi'), ('here', 'lama1277-waiwa', 'təpi'), ('here', 'lama1277-lewok', 'bədi'), ('here', 'lama1277-belan', 'nəpi'), ('here', 'lama1277-merde', 'təpi'), ('here', 'koto1251', 'ia'), ('here', 'lama1277-lewog', 'pi'), ('here', 'lama1277-imulo', 'kətəri'), ('here', 'lama1277-tanju', 'pia'), ('here', 'lama1277-baipi', 'tiwi'), ('here', 'lama1277-lewoi', 'ˈpiʔiŋ'), ('here', 'lama1277-lamak', 'dəwi'), ('here', 'keda1252-leuwa', 'bæʔ')}
 {('here', 'lama1277-lewuk', 'bene'), ('here', 'lama1277-kalik', 're'), ('here', 'lama1277-kalik', 'rene'), ('here', 'lama1277-lerek', 'rəˈbe'), ('here', 'lama1277-paina', 'rəbe')}
 {('here', 'lama1277-minga', 'rori')}


Concept ‘dowry’ is different between Alorese and Lamaholot.

 Alorese innovated the following forms:
 {('dowry', 'alor1247-besar', "hodaŋ 'balɛ")}
 One or more Lamaholot dialects innovated forms not found in Alorese:
 {('dowry', 'lama1277-adona', 'ˈʔohɛ')}
 {('dowry', 'lama1277-kalik', 'ˈɔi  vəˈrat')}

  Just for clarity:
  There are also the following similarity classes shared between Alorese and LH.
  {('dowry', 'alor1247-pandai', "weli 'pala"), ('dowry', 'lama1277-lewoi', 'ˈweliŋ ˈəlɑŋ'), ('dowry', 'alor1247-munas', "weli 'pala"), ('dowry', 'lama1277-lerek', 'ˈvelin')}


Concept ‘to_chase_away_to_run_after’ is different between Alorese and Lamaholot.

 Alorese innovated the following forms:
 {('to_chase_away_to_run_after', 'alor1247-munas', 'lisoʔ'), ('to_chase_away_to_run_after', 'alor1247-pandai', 'liso')}
 {('to_chase_away_to_run_after', 'alor1247-besar', 'tutɛ')}
 {('to_chase_away_to_run_after', 'alor1247-pandai', 'ladːɔ')}
 One or more Lamaholot dialects innovated forms not found in Alorese:
 {('to_chase_away_to_run_after', 'lama1277-adona', 'ˈdore'), ('to_chase_away_to_run_after', 'sarr1247-adiab', 'gatijas'), ('to_chase_away_to_run_after', 'blag1240-tuntu', "ge'teri"), ('to_chase_away_to_run_after', 'rett1240', 'gatiari'), ('to_chase_away_to_run_after', 'kaer1234', "gɛ'tɛro"), ('to_chase_away_to_run_after', 'dein1238', 'gatias'), ('to_chase_away_to_run_after', 'kuii1253', 'gapateri'), ('to_chase_away_to_run_after', 'baka1276', 'teri'), ('to_chase_away_to_run_after', 'rett1240', 'gatieri'), ('to_chase_away_to_run_after', 'sarr1247-nule', 'getiris'), ('to_chase_away_to_run_after', 'blag1240-nule', 'teri'), ('to_chase_away_to_run_after', 'blag1240-bama', 'geteriŋ'), ('to_chase_away_to_run_after', 'pura1258', "a'teri"), ('to_chase_away_to_run_after', 'lama1277-lerek', 'dəɡuˈrəŋ'), ('to_chase_away_to_run_after', 'rett1240', 'tiari'), ('to_chase_away_to_run_after', 'lama1277-lewoi', 'ˈtəruŋ'), ('to_chase_away_to_run_after', 'teiw1235', 'gətiar')}
 {('to_chase_away_to_run_after', 'lama1277-kalik', 'oˈkap')}


Concept ‘to_hit’ is different between Alorese and Lamaholot.

 Alorese innovated the following forms:
 {('to_hit', 'alor1247-munas', 'boaʔ'), ('to_hit', 'alor1247-pandai', 'boa')}
 {('to_hit', 'adan1251-lawah', 'bihiʔ'), ('to_hit', 'hama1240', 'bɛh'), ('to_hit', 'alor1247-baran', 'bɛh:ɛ'), ('to_hit', 'alor1247-pandai', 'bɛhɛ'), ('to_hit', 'kabo1247', "bi'siʔ"), ('to_hit', 'kuii1253', 'bles'), ('to_hit', 'fata1247', 'paha'), ('to_hit', 'adan1251-otvai', '’bɛh'), ('to_hit', 'alor1247-besar', 'bɛhɛ')}
 Alorese shares the following forms with TAP languages:
 {('to_hit', 'alor1247-munas', 'boaʔ'), ('to_hit', 'alor1247-pandai', 'boa')}
 {('to_hit', 'adan1251-lawah', 'bihiʔ'), ('to_hit', 'hama1240', 'bɛh'), ('to_hit', 'alor1247-baran', 'bɛh:ɛ'), ('to_hit', 'alor1247-pandai', 'bɛhɛ'), ('to_hit', 'kabo1247', "bi'siʔ"), ('to_hit', 'kuii1253', 'bles'), ('to_hit', 'fata1247', 'paha'), ('to_hit', 'adan1251-otvai', '’bɛh'), ('to_hit', 'alor1247-besar', 'bɛhɛ')}
 Alorese shares the following forms with other AN languanges, but not Lamaholot:
 {('to_hit', 'sika1262-maume', 't̪au'), ('to_hit', 'alor1247-munas', 'danɛ'), ('to_hit', 'mamb1306', 'dɑe'), ('to_hit', 'alor1247-besar', 'danɛ')}
 {('to_hit', 'koto1251', 'n-tufu'), ('to_hit', 'idat1237', "tu'u"), ('to_hit', 'tetu1246', 'tuku'), ('to_hit', 'laka1255', 'tuku'), ('to_hit', 'alor1247-munas', 'tubuʔ')}
 One or more Lamaholot dialects innovated forms not found in Alorese:
 {('to_hit', 'lama1277-adona', 'ˈbɜteːk')}
 {('to_hit', 'lama1277-lewoi', 'ˈbəŋɔ'), ('to_hit', 'koto1251', 'n-bana'), ('to_hit', 'lama1277-lerek', 'bəŋoˈlaŋ'), ('to_hit', 'tuku1254', 'bɔnu'), ('to_hit', 'kama1365', 'boʔna')}
 {('to_hit', 'p-cent2245-abvd', '*palu'), ('to_hit', 'kema1243', 'para'), ('to_hit', 'abui1241-takal', 'bo:l'), ('to_hit', 'atim1239', 'bol'), ('to_hit', 'abui1241-ulaga', 'bol'), ('to_hit', 'keda1252-leuwa', 'paluʔ'), ('to_hit', 'lama1277-kalik', 'paˈluk'), ('to_hit', 'kafo1240', 'bɑlɑi'), ('to_hit', 'keda1252-leuba', "pa'luʔ"), ('to_hit', 'abui1241-fuime', 'tebol'), ('to_hit', 'p-mala1545-abvd', '*palu'), ('to_hit', 'lama1277-adona', 'ˈplei'), ('to_hit', 'kira1248', 'baˈlak'), ('to_hit', 'atim1239', 'ne-bol'), ('to_hit', 'p-aust1307-abvd', '*palu')}


Concept ‘heel’ is different between Alorese and Lamaholot.

 Alorese innovated the following forms:
 {('heel', 'alor1247-besar', "leiŋ 'ubɔŋ")}

  Just for clarity:
  There are also the following similarity classes shared between Alorese and LH.
  {('heel', 'alor1247-pandai', "leiŋ 'udek"), ('heel', 'lama1277-lewoi', 'ˈude'), ('heel', 'lama1277-kalik', 'nuˈdəp'), ('heel', 'alor1247-munas', "lei 'udɛk"), ('heel', 'lama1277-lerek', 'ˈudəm'), ('heel', 'alor1247-munas', "leiŋ 'udɛk"), ('heel', 'lama1277-adona', 'ˈnudɜrɜt')}


Concept ‘rattan’ is different between Alorese and Lamaholot.

 Alorese shares the following forms with TAP languages:
 {('rattan', 'blag1240-tuntu', 'leg'), ('rattan', 'blag1240-warsa', 'le:g'), ('rattan', 'teiw1235', 'lijag'), ('rattan', 'blag1240-kulij', 'lia'), ('rattan', 'rett1240', 'lijag'), ('rattan', 'blag1240-nule', 'lija'), ('rattan', 'adan1251-lawah', 'lɛʔ'), ('rattan', 'pura1258', 'lia'), ('rattan', 'kaer1234', 'lɛ:g'), ('rattan', 'alor1247-munas', 'lua'), ('rattan', 'adan1251-otvai', '’le'), ('rattan', 'kuii1253', 'le'), ('rattan', 'dein1238', 'liax'), ('rattan', 'blag1240-bama', 'leg'), ('rattan', 'baka1276', 'lija'), ('rattan', 'rett1240', 'liag'), ('rattan', 'kabo1247', 'lojoʔ'), ('rattan', 'alor1247-pandai', 'lua'), ('rattan', 'sarr1247-nule', 'lijag'), ('rattan', 'sarr1247-adiab', 'lijah')}
 Alorese shares the following forms with other AN languanges, but not Lamaholot:
 {('rattan', 'sika1262-hewa', 'ue'), ('rattan', 'alor1247-pandai', 'ue'), ('rattan', 'alor1247-munas', 'uwe')}
 One or more Lamaholot dialects innovated forms not found in Alorese:
 {('rattan', 'lama1277-kalik', 'uˈa'), ('rattan', 'lama1277-kalik', 'uˈaʤ')}
 {('rattan', 'lama1277-lerek', 'ˈuar')}
 {('rattan', 'lama1277-adona', 'ˈʔua')}

  Just for clarity:
  There are also the following similarity classes shared between Alorese and LH.
  {('rattan', 'alor1247-besar', 'ufi'), ('rattan', 'lama1277-lewoi', 'uˈwajəŋ')}


Concept ‘to_come’ is different between Alorese and Lamaholot.

 Alorese innovated the following forms:
 {('to_come', 'alor1247-baran', 'nɔu')}
 One or more Lamaholot dialects innovated forms not found in Alorese:
 {('to_come', 'lama1277-lewop', 'məfi'), ('to_come', 'lama1277-lewop', 'kəfi')}
 {('to_come', 'lama1277-paina', 'oliŋ'), ('to_come', 'lama1277-lewom', 'oli'), ('to_come', 'lama1277-kalik', 'ɔˈli'), ('to_come', 'lama1277-kalik', 'oli')}
 {('to_come', 'lama1277-merde', 'lohe'), ('to_come', 'lama1277-lewoe', 'hoʔe'), ('to_come', 'lama1277-lamah', 'lohe')}
 {('to_come', 'lama1277-lewoi', 'ˈhəwɔ')}
 {('to_come', 'lama1277-lewok', 'haka')}
 {('to_come', 'lama1277-lewoi', 'ˈsəɡa'), ('to_come', 'lama1277-ritae', 'səga'), ('to_come', 'lama1277-lewob', 'səga'), ('to_come', 'lama1277-pukau', 'səgat'), ('to_come', 'sika1262-hewa', 'səga'), ('to_come', 'lama1277-baipi', 'səga'), ('to_come', 'lama1277-lewog', 'səga'), ('to_come', 'lama1277-bama', 'səga'), ('to_come', 'p-mala1545-acd', '*teka'), ('to_come', 'lama1277-tanju', 'səga')}
 {('to_come', 'kira1248', 'mai'), ('to_come', 'wers1238-taram', 'amai'), ('to_come', 'abui1241-fuime', 'me'), ('to_come', 'sawi1256', "a'me"), ('to_come', 'p-mala1545-abvd', '*maʀi'), ('to_come', 'baka1276', 'ma'), ('to_come', 'tuku1254', 'ma:i'), ('to_come', 'p-alor1249', '*mai'), ('to_come', 'sarr1247-nule', 'ma:'), ('to_come', 'abui1241-takal', 'me̞'), ('to_come', 'kafo1240', 'mɑi'), ('to_come', 'tetu1246', 'mɑi'), ('to_come', 'p-timo1261', '*mai'), ('to_come', 'lamm1241-westp', 'ma'), ('to_come', 'blag1240-warsa', 'ma'), ('to_come', 'kaer1234', 'ma'), ('to_come', 'buna1278-malia', 'ma:n'), ('to_come', 'abui1241-ulaga', 'mia.'), ('to_come', 'hama1240', 'maʔ'), ('to_come', 'blag1240-tuntu', 'ma'), ('to_come', 'sika1262-hewa', 'main'), ('to_come', 'kula1280-lanto', 'yame'), ('to_come', 'lama1277-lewuk', 'mene'), ('to_come', 'wers1238-marit', "a'mai"), ('to_come', 'kema1243', 'mɑi'), ('to_come', 'kabo1247', 'maʔ'), ('to_come', 'sika1262-maume', 'main'), ('to_come', 'tetu1245-suai', 'mai'), ('to_come', 'p-cent2245-abvd', '*mai'), ('to_come', 'sika1262-tanai', 'mai'), ('to_come', 'buna1278-suai', 'man'), ('to_come', 'dein1238', 'ma'), ('to_come', 'tetu1245-vique', 'mai'), ('to_come', 'sika1262-hewa', 'mai'), ('to_come', 'blag1240-nule', 'ma'), ('to_come', 'koto1251', 'nema'), ('to_come', 'blag1240-bama', 'ma'), ('to_come', 'sarr1247-adiab', 'ma'), ('to_come', 'atim1239', 'me'), ('to_come', 'idat1237', 'ma:'), ('to_come', 'buna1278-bobon', 'man'), ('to_come', 'laka1255', 'ma:n'), ('to_come', 'kama1365', 'me'), ('to_come', 'mamb1306', 'ma:'), ('to_come', 'kuii1253', 'marán'), ('to_come', 'kelo1247-hopte', 'ma:'), ('to_come', 'rett1240', 'ma')}

  Just for clarity:
  There are also the following similarity classes shared between Alorese and LH.
  {('to_come', 'alor1247-munas', 'bɛta'), ('to_come', 'alor1247-baran', 'bɛta'), ('to_come', 'lama1277-belan', 'bəsol'), ('to_come', 'sika1262-tanai', 'boʔu'), ('to_come', 'lama1277-lamal', 'bəso'), ('to_come', 'lama1277-minga', 'bəsol'), ('to_come', 'lama1277-watan', 'beto'), ('to_come', 'lama1277-lerek', 'ˈbedʒ͡ɔ'), ('to_come', 'lama1277-mulan', 'bəso'), ('to_come', 'alor1247-pandai', 'beta'), ('to_come', 'lama1277-lamat', 'bəso'), ('to_come', 'lama1277-botun', 'beto'), ('to_come', 'lama1277-lewot', 'bəsol'), ('to_come', 'lama1277-imulo', 'bəsol'), ('to_come', 'lama1277-adona', 'ˈbeto'), ('to_come', 'lama1277-kiwan', 'beto'), ('to_come', 'lama1277-lamak', 'beto'), ('to_come', 'alor1247-besar', 'bɛta'), ('to_come', 'lama1277-wuake', 'bəsol'), ('to_come', 'lama1277-dulhi', 'beto'), ('to_come', 'lama1277-waiba', 'beto'), ('to_come', 'lama1277-ileap', 'beto'), ('to_come', 'alor1247-munas', 'bɛtaŋ'), ('to_come', 'lama1277-waiwa', 'beto'), ('to_come', 'lama1277-horow', 'beto')}


Concept ‘fish_trap’ is different between Alorese and Lamaholot.

 Alorese innovated the following forms:
 {('fish_trap', 'alor1247-munas', 'wuwɔ'), ('fish_trap', 'alor1247-besar', 'fufɔ'), ('fish_trap', 'alor1247-pandai', 'wuwo')}
 {('fish_trap', 'alor1247-pandai', 'knopal'), ('fish_trap', 'wers1238-taram', 'knopoŋ'), ('fish_trap', 'alor1247-munas', 'nɔpal'), ('fish_trap', 'alor1247-besar', "ka'nopaŋ")}
 {('fish_trap', 'blag1240-bama', 'wer'), ('fish_trap', 'alor1247-pandai', 'kɛr'), ('fish_trap', 'baka1276', 'ver'), ('fish_trap', 'teiw1235', 'kɛ:r'), ('fish_trap', 'blag1240-tuntu', 'ver'), ('fish_trap', 'wers1238-marit', 'ar'), ('fish_trap', 'blag1240-nule', 'ker'), ('fish_trap', 'kula1280-lanto', '’gar')}
 Alorese shares the following forms with TAP languages:
 {('fish_trap', 'alor1247-munas', 'wuwɔ'), ('fish_trap', 'alor1247-besar', 'fufɔ'), ('fish_trap', 'alor1247-pandai', 'wuwo')}
 {('fish_trap', 'alor1247-pandai', 'knopal'), ('fish_trap', 'wers1238-taram', 'knopoŋ'), ('fish_trap', 'alor1247-munas', 'nɔpal'), ('fish_trap', 'alor1247-besar', "ka'nopaŋ")}
 {('fish_trap', 'blag1240-bama', 'wer'), ('fish_trap', 'alor1247-pandai', 'kɛr'), ('fish_trap', 'baka1276', 'ver'), ('fish_trap', 'teiw1235', 'kɛ:r'), ('fish_trap', 'blag1240-tuntu', 'ver'), ('fish_trap', 'wers1238-marit', 'ar'), ('fish_trap', 'blag1240-nule', 'ker'), ('fish_trap', 'kula1280-lanto', '’gar')}
 One or more Lamaholot dialects innovated forms not found in Alorese:
 {('fish_trap', 'lama1277-adona', 'namaʔ')}
 {('fish_trap', 'lama1277-lewoi', 'bɜˈlutuʔ')}

  Just for clarity:
  There are also the following similarity classes shared between Alorese and LH.
  {('fish_trap', 'lama1277-kalik', 'puˈkət'), ('fish_trap', 'lama1277-lerek', 'ˈpukat'), ('fish_trap', 'alor1247-pandai', 'pukɔ')}


Concept ‘word’ is different between Alorese and Lamaholot.

 Alorese innovated the following forms:
 {('word', 'alor1247-munas', "k'dirɛʔ"), ('word', 'alor1247-besar', 'kirɛ'), ('word', 'alor1247-pandai', "k'dirɛ")}
 One or more Lamaholot dialects innovated forms not found in Alorese:
 {('word', 'lama1277-lerek', 'ɡəpəˈraŋ')}
 {('word', 'lama1277-lerek', 'ˈkaiŋ')}
 {('word', 'lama1277-adona', 'ˈkoda'), ('word', 'sika1262-hewa', 'kata'), ('word', 'lama1277-lewoi', 'ˈkoda')}
 {('word', 'lama1277-kalik', 'maˈkət')}


Concept ‘to_guard’ is different between Alorese and Lamaholot.

 Alorese innovated the following forms:
 {('to_guard', 'alor1247-pandai', 'sɛru')}
 {('to_guard', 'alor1247-besar', "sa'daha")}
 {('to_guard', 'blag1240-nule', "gə'rejaŋ"), ('to_guard', 'alor1247-munas', 'kwaraŋ')}
 Alorese shares the following forms with TAP languages:
 {('to_guard', 'alor1247-pandai', 'sɛru')}
 {('to_guard', 'alor1247-besar', "sa'daha")}
 {('to_guard', 'blag1240-nule', "gə'rejaŋ"), ('to_guard', 'alor1247-munas', 'kwaraŋ')}
 One or more Lamaholot dialects innovated forms not found in Alorese:
 {('to_guard', 'lama1277-lewoi', 'likɔ ˈlapa')}
 {('to_guard', 'lama1277-lerek', 'ˈbavat')}

  Just for clarity:
  There are also the following similarity classes shared between Alorese and LH.
  {('to_guard', 'kira1248', 'uʤaga'), ('to_guard', 'sika1262-hewa', 'ʤaga'), ('to_guard', 'lama1277-adona', 'dʒaɡa'), ('to_guard', 'alor1247-pandai', 'dʒaga')}


Concept ‘ceremonial_place’ is different between Alorese and Lamaholot.

 Alorese innovated the following forms:
 {('ceremonial_place', 'alor1247-besar', "dolu 'nɔŋ 'ula 'naga")}
 {('ceremonial_place', 'alor1247-munas', "lɛlaŋ 'ɔnɔŋ")}
 {('ceremonial_place', 'alor1247-pandai', 'dɔru')}
 One or more Lamaholot dialects innovated forms not found in Alorese:
 {('ceremonial_place', 'lama1277-kalik', 'ˈnuba')}
 {('ceremonial_place', 'lama1277-kalik', 'ˈnɔbɛŋ'), ('ceremonial_place', 'lama1277-lerek', 'uˈlik noˈbeŋ')}
 {('ceremonial_place', 'lama1277-lewoi', 'ˈmɛraŋ')}
 {('ceremonial_place', 'lama1277-adona', 'basa')}


Concept ‘to_be_silent’ is different between Alorese and Lamaholot.

 Alorese innovated the following forms:
 {('to_be_silent', 'alor1247-besar', "man'nehe")}
 {('to_be_silent', 'abui1241-ulaga', 'rama'), ('to_be_silent', 'alor1247-munas', 'ramaʔ'), ('to_be_silent', 'alor1247-pandai', 'rama'), ('to_be_silent', 'abui1241-takal', 'rä.mä')}
 Alorese shares the following forms with TAP languages:
 {('to_be_silent', 'alor1247-besar', "man'nehe")}
 {('to_be_silent', 'abui1241-ulaga', 'rama'), ('to_be_silent', 'alor1247-munas', 'ramaʔ'), ('to_be_silent', 'alor1247-pandai', 'rama'), ('to_be_silent', 'abui1241-takal', 'rä.mä')}
 One or more Lamaholot dialects innovated forms not found in Alorese:
 {('to_be_silent', 'lama1277-adona', 'ˈpaha')}
 {('to_be_silent', 'lama1277-adona', 'ˈpiʔe')}
 {('to_be_silent', 'lama1277-lerek', 'məl-məl')}
 {('to_be_silent', 'lama1277-kalik', 'kəˈpae'), ('to_be_silent', 'lama1277-lewoi', 'ˈkəpərə')}
 {('to_be_silent', 'lama1277-kalik', 'məˈli')}


Concept ‘to_throw’ is different between Alorese and Lamaholot.

 Alorese shares the following forms with other AN languanges, but not Lamaholot:
 {('to_throw', 'kema1243', 'tiɑr'), ('to_throw', 'alor1247-munas', 'deiʔ'), ('to_throw', 'mamb1306', 'tia'), ('to_throw', 'alor1247-besar', 'deiŋ'), ('to_throw', 'alor1247-baran', 'dɛĩ:'), ('to_throw', 'idat1237', 'tɛ:r'), ('to_throw', 'tuku1254', 'tia'), ('to_throw', 'laka1255', 'tɛ:r'), ('to_throw', 'alor1247-pandai', 'dei')}
 One or more Lamaholot dialects innovated forms not found in Alorese:
 {('to_throw', 'lama1277-horow', 'paluk'), ('to_throw', 'sika1262-tanai', 'roga'), ('to_throw', 'sika1262-maume', 'loga')}
 {('to_throw', 'lama1277-minga', 'pədi'), ('to_throw', 'lama1277-lewot', 'pədi'), ('to_throw', 'lama1277-imulo', 'ped'), ('to_throw', 'lama1277-lewop', 'pədi')}
 {('to_throw', 'lama1277-lewog', 'wərã'), ('to_throw', 'koto1251', 'n-firin'), ('to_throw', 'lama1277-lewoi', 'ˈwərəŋ'), ('to_throw', 'lama1277-lewoi', 'ˈwalɛŋ'), ('to_throw', 'lama1277-bama', 'wərə')}
 {('to_throw', 'lama1277-kalik', 'batu'), ('to_throw', 'p-mala1545-acd', '*batu'), ('to_throw', 'lama1277-lerek', 'ˈbato'), ('to_throw', 'lama1277-lewuk', 'batu'), ('to_throw', 'lama1277-kalik', 'ˈbatu')}
 {('to_throw', 'lama1277-waiwa', 'gahak'), ('to_throw', 'lama1277-dulhi', 'gahak'), ('to_throw', 'lama1277-watan', 'gahak'), ('to_throw', 'lamm1241-westp', 'guʔa'), ('to_throw', 'lama1277-adona', 'ɡɑˈhɑk'), ('to_throw', 'lama1277-ileap', 'gaha'), ('to_throw', 'lama1277-lamak', 'gahak'), ('to_throw', 'sika1262-tanai', 'βehak')}
 {('to_throw', 'lama1277-lewok', 'dəka'), ('to_throw', 'sika1262-hewa', 'təgu'), ('to_throw', 'lama1277-lerek', 'dəkaŋ'), ('to_throw', 'lama1277-tanju', 'dəka'), ('to_throw', 'koto1251', 'n-tokar'), ('to_throw', 'lama1277-mulan', 'dəka'), ('to_throw', 'sika1262-hewa', 'dəgu'), ('to_throw', 'tetu1245-vique', 'daka'), ('to_throw', 'tetu1245-suai', 'daka'), ('to_throw', 'lama1277-paina', 'dəkaŋ')}
 {('to_throw', 'lama1277-lewoe', 'geba'), ('to_throw', 'lama1277-lewom', 'geba'), ('to_throw', 'lama1277-ritae', 'geba'), ('to_throw', 'lama1277-adona', 'ɡeˈbaʔ'), ('to_throw', 'fata1247', 'kalɛ'), ('to_throw', 'buna1278-bobon', 'gɛbɛl'), ('to_throw', 'lama1277-wuake', 'geba'), ('to_throw', 'lama1277-lamah', 'geba'), ('to_throw', 'lama1277-waiba', 'geba'), ('to_throw', 'lama1277-belan', 'geba'), ('to_throw', 'lama1277-kiwan', 'geba'), ('to_throw', 'lama1277-lamal', 'geba'), ('to_throw', 'lama1277-botun', 'geba'), ('to_throw', 'lama1277-lamat', 'gebə'), ('to_throw', 'lama1277-merde', 'geba'), ('to_throw', 'sika1262-tanai', 'ɡeba'), ('to_throw', 'buna1278-suai', 'gɛbɛl'), ('to_throw', 'lama1277-baipi', 'geba'), ('to_throw', 'lama1277-lewob', 'geba')}
 {('to_throw', 'p-mala1545-acd', '*tudaq'), ('to_throw', 'lama1277-pukau', 'tubak '), ('to_throw', 'p-cent2245-abvd', '*tudaq'), ('to_throw', 'p-mala1545-abvd', '*tudaq')}


Concept ‘rainbow’ is different between Alorese and Lamaholot.

 Alorese innovated the following forms:
 {('rainbow', 'alor1247-munas', "tat 'beiŋ")}
 One or more Lamaholot dialects innovated forms not found in Alorese:
 {('rainbow', 'lama1277-lewoi', 'patuŋ ˈtawa'), ('rainbow', 'lama1277-adona', 'ˈpatũ'), ('rainbow', 'lama1277-kalik', 'tokˈbaʤu')}

  Just for clarity:
  There are also the following similarity classes shared between Alorese and LH.
  {('rainbow', 'alor1247-besar', 'tata bei-bei'), ('rainbow', 'alor1247-pandai', "tata 'bai"), ('rainbow', 'lama1277-lerek', 'tat ˈbajuŋ')}


Concept ‘this’ is different between Alorese and Lamaholot.

 Alorese innovated the following forms:
 {('this', 'alor1247-besar', 'hã'), ('this', 'alor1247-baran', ",ha'ʔã")}
 {('this', 'alor1247-pandai', 'kɛ'), ('this', 'alor1247-munas', 'ke')}
 One or more Lamaholot dialects innovated forms not found in Alorese:
 {('this', 'lama1277-mulan', 'nəpi'), ('this', 'lama1277-wuake', 'nəpi'), ('this', 'lama1277-lamal', 'pi'), ('this', 'lama1277-lewog', 'piʔĩ'), ('this', 'lama1277-bama', 'piʔĩ'), ('this', 'lama1277-tanju', 'piʔĩ'), ('this', 'lama1277-lewom', 'piʔi'), ('this', 'lama1277-watan', 'pi'), ('this', 'lama1277-lamah', 'napi'), ('this', 'lama1277-ileap', 'pi'), ('this', 'lama1277-baipi', 'pi'), ('this', 'lama1277-botun', 'pi'), ('this', 'lama1277-lerek', 'naˈbe'), ('this', 'koto1251', '#RIF!'), ('this', 'lama1277-lewoi', 'ˈpiː'), ('this', 'lama1277-paina', 'nəbe'), ('this', 'lama1277-lewok', 'bədi'), ('this', 'lama1277-belan', 'nəpi'), ('this', 'lama1277-waiba', 'pi')}
 {('this', 'keda1252-leuwa', 'nɔʔ'), ('this', 'idat1237', 'nia'), ('this', 'lama1277-horow', 'ni'), ('this', 'p-aust1307-abvd', '*i-ni'), ('this', 'p-cent2245-abvd', '*-ni'), ('this', 'lama1277-lamat', 'noni'), ('this', 'sawi1256', 'uŋanu'), ('this', 'fata1247', "ɛ'niɁi"), ('this', 'kema1243', 'nɔgɔ'), ('this', 'lama1277-merde', 'niʔi'), ('this', 'keda1252-leuba', 'nɔʔɔ'), ('this', 'keda1252', 'noʔo'), ('this', 'pura1258', 'aŋa'), ('this', 'tetu1246', 'ne:'), ('this', 'lama1277-waiwa', 'ni'), ('this', 'lama1277-adona', 'ˈnhiː'), ('this', 'tetu1245-vique', 'neʔe'), ('this', 'p-mala1545-abvd', '*i ni'), ('this', 'lama1277-dulhi', 'ni'), ('this', 'p-mala1545-acd', '*i-ni'), ('this', 'tetu1245-suai', 'nɛ:'), ('this', 'lama1277-lamak', 'nəwi'), ('this', 'lama1277-kiwan', 'ni')}
 {('this', 'lama1277-lewoe', 'bobo')}
 {('this', 'lama1277-lewop', 'nakri'), ('this', 'lama1277-imulo', 'nakri'), ('this', 'lama1277-minga', 'nakri'), ('this', 'mamb1306', "ɑ'rɑi"), ('this', 'lama1277-lewot', 'nakuri')}
 {('this', 'lama1277-lewuk', 'me')}
 {('this', 'abui1241-petle', "i.'ti"), ('this', 'sika1262-hewa', 'ete'), ('this', 'abui1241-takal', 'dɔ'), ('this', 'sika1262-hewa', 'ʔete'), ('this', 'abui1241-ulaga', 'do'), ('this', 'lama1277-pukau', 'iteʔẽ'), ('this', 'lama1277-lewob', 'teʔẽ')}
 {('this', 'lama1277-kalik', 'rene'), ('this', 'lama1277-kalik', 've'), ('this', 'lama1277-kalik', 'vene')}
 {('this', 'lama1277-ritae', 'tokowe')}


Concept ‘ringworm’ is different between Alorese and Lamaholot.

 Alorese innovated the following forms:
 {('ringworm', 'alor1247-besar', "ha'rukaŋ")}
 One or more Lamaholot dialects innovated forms not found in Alorese:
 {('ringworm', 'lama1277-lewoi', 'ˈaho iluŋ')}
 {('ringworm', 'sika1262-hewa', 'ʋəne'), ('ringworm', 'lama1277-lerek', 'wəˈner')}
 {('ringworm', 'lama1277-kalik', 'kənəˈtək')}

  Just for clarity:
  There are also the following similarity classes shared between Alorese and LH.
  {('ringworm', 'blag1240-warsa', 'xot'), ('ringworm', 'blag1240-kulij', 'kot'), ('ringworm', 'baka1276', 'katu'), ('ringworm', 'lamm1241-westp', 'kat:ur'), ('ringworm', 'rett1240', 'katu'), ('ringworm', 'adan1251-otvai', '’a:t'), ('ringworm', 'rett1240', "ka't:u"), ('ringworm', 'alor1247-munas', "katu 'datɛʔ"), ('ringworm', 'blag1240-warsa', 'qot'), ('ringworm', 'nede1245', 'katu'), ('ringworm', 'kelo1247-hopte', 'kat'), ('ringworm', 'kaer1234', 'qatu'), ('ringworm', 'blag1240-tuntu', 'qot'), ('ringworm', 'alor1247-pandai', 'katu'), ('ringworm', 'adan1251-lawah', 'ot'), ('ringworm', 'lama1277-adona', 'kəˈbuʔu'), ('ringworm', 'kabo1247', 'kaʧ')}


Concept ‘cold_influenza’ is different between Alorese and Lamaholot.

 Alorese innovated the following forms:
 {('cold_influenza', 'alor1247-pandai', "iruŋ 'matːɛ"), ('cold_influenza', 'alor1247-munas', "iru'ɔnɔŋ mə'tːɛʔ"), ('cold_influenza', 'alor1247-besar', "niruŋ 'matɛ")}
 {('cold_influenza', 'alor1247-munas', "ha'nːuŋ")}
 One or more Lamaholot dialects innovated forms not found in Alorese:
 {('cold_influenza', 'lama1277-kalik', 'nurən'), ('cold_influenza', 'lama1277-kalik', 'nur')}
 {('cold_influenza', 'lama1277-lewoi', 'dapu ˈmekə')}
 {('cold_influenza', 'lama1277-lerek', 'niˈdʒ͡or')}
 {('cold_influenza', 'lama1277-adona', 'pɜˈlate-ɡɜˈlɜtɜ')}


Concept ‘to_eat’ is different between Alorese and Lamaholot.

 One or more Lamaholot dialects innovated forms not found in Alorese:
 {('to_eat', 'lama1277-lewot', 'pa')}
 {('to_eat', 'lama1277-lewoi', 'ˈbuʔa')}

  Just for clarity:
  There are also the following similarity classes shared between Alorese and LH.
  {('to_eat', 'lama1277-paina', 'ka'), ('to_eat', 'alor1247-pandai', 'gaŋ'), ('to_eat', 'lama1277-lerek', 'ka'), ('to_eat', 'lama1277-pukau', 'go'), ('to_eat', 'alor1247-pandai', 'kaŋ '), ('to_eat', 'lama1277-lewop', 'ka'), ('to_eat', 'keda1252-leuwa', 'a'), ('to_eat', 'lama1277-tanju', 'təkã'), ('to_eat', 'lama1277-lamah', 'kã'), ('to_eat', 'lama1277-wuake', 'ka'), ('to_eat', 'keda1252-leuba', 'kaː'), ('to_eat', 'lama1277-horow', 'ka'), ('to_eat', 'lama1277-minga', 'ka'), ('to_eat', 'p-mala1545-abvd', '*kaen'), ('to_eat', 'lama1277-dulhi', 'təka'), ('to_eat', 'p-aust1307-abvd', '*kaen'), ('to_eat', 'lama1277-lewok', 'ga'), ('to_eat', 'lama1277-bama', 'təkã'), ('to_eat', 'lama1277-lewop', 'ga'), ('to_eat', 'lama1277-belan', 'ka'), ('to_eat', 'p-cent2245-abvd', '*kan'), ('to_eat', 'lama1277-mulan', 'kɤ'), ('to_eat', 'lama1277-lewoe', 'a'), ('to_eat', 'lama1277-adona', 'ˈgoe kãː'), ('to_eat', 'keda1252', 'a'), ('to_eat', 'lama1277-lamat', 'a'), ('to_eat', 'lama1277-mulan', 'go'), ('to_eat', 'lama1277-pukau', 'kã'), ('to_eat', 'lama1277-merde', 'kã'), ('to_eat', 'lama1277-merde', 'go'), ('to_eat', 'lama1277-baipi', 'kã'), ('to_eat', 'alor1247-besar', "kaŋ 'apa"), ('to_eat', 'lama1277-kalik', 'ka'), ('to_eat', 'lama1277-ileap', 'ka'), ('to_eat', 'lama1277-botun', 'ka'), ('to_eat', 'keda1252', 'ka'), ('to_eat', 'lama1277-lewuk', 'ga'), ('to_eat', 'lama1277-lamat', 'ga'), ('to_eat', 'lama1277-lewog', 'təkã'), ('to_eat', 'lama1277-lewuk', 'ka'), ('to_eat', 'lama1277-wuake', 'ga'), ('to_eat', 'lama1277-lewom', 'go'), ('to_eat', 'lama1277-ritae', 'go'), ('to_eat', 'lama1277-lewob', 'təkã'), ('to_eat', 'lama1277-waiba', 'kã'), ('to_eat', 'lama1277-lamal', 'kã'), ('to_eat', 'lama1277-ileap', 'go'), ('to_eat', 'lama1277-lamak', 'go'), ('to_eat', 'lama1277-lewoi', 'ˈtəkɑŋ'), ('to_eat', 'lama1277-horow', 'go'), ('to_eat', 'lama1277-waiwa', 'go'), ('to_eat', 'lama1277-kalik', 'ga'), ('to_eat', 'lama1277-waiwa', 'kã'), ('to_eat', 'lama1277-lamak', 'kã'), ('to_eat', 'lama1277-paina', 'ga'), ('to_eat', 'p-mala1545-acd', '*kaen'), ('to_eat', 'lama1277-lamal', 'go'), ('to_eat', 'lama1277-lewoe', 'ga'), ('to_eat', 'lama1277-imulo', 'ga'), ('to_eat', 'alor1247-munas', 'kaŋ '), ('to_eat', 'lama1277-imulo', 'ka'), ('to_eat', 'lama1277-lewom', 'kã'), ('to_eat', 'lama1277-kiwan', 'ka'), ('to_eat', 'lama1277-lewot', 'ga'), ('to_eat', 'lama1277-ritae', 'kã'), ('to_eat', 'alor1247-pandai', 'gouŋ'), ('to_eat', 'lama1277-waiba', 'go'), ('to_eat', 'lama1277-watan', 'go'), ('to_eat', 'lama1277-botun', 'go'), ('to_eat', 'lama1277-lewok', 'ka'), ('to_eat', 'lama1277-lerek', 'ɡa'), ('to_eat', 'lama1277-baipi', 'go')}


Concept ‘bad’ is different between Alorese and Lamaholot.

 Alorese innovated the following forms:
 {('bad', 'alor1247-munas', "rupaŋ 'lahɛ")}
 One or more Lamaholot dialects innovated forms not found in Alorese:
 {('bad', 'lama1277-lewom', 'afadã')}
 {('bad', 'lama1277-lerek', 'ɡuˈtun'), ('bad', 'lama1277-paina', 'gutun'), ('bad', 'p-aust1307-abvd', '*kuya')}
 {('bad', 'lama1277-lewoi', 'ˈməkɔʔ'), ('bad', 'lama1277-lewop', 'məkənən'), ('bad', 'lama1277-mulan', 'mədo'), ('bad', 'lama1277-botun', 'mədõ'), ('bad', 'lama1277-lewog', 'məko'), ('bad', 'lama1277-horow', 'mədõ'), ('bad', 'lama1277-minga', 'məkɤ'), ('bad', 'lama1277-kiwan', 'mədho'), ('bad', 'lama1277-adona', 'maˈdoː'), ('bad', 'lama1277-bama', 'məko'), ('bad', 'lama1277-pukau', 'məko'), ('bad', 'lama1277-waiwa', 'madõ'), ('bad', 'lama1277-watan', 'mədõʔ'), ('bad', 'lama1277-wuake', 'məkeŋ'), ('bad', 'lama1277-dulhi', 'mədõ'), ('bad', 'lama1277-lewob', 'məko'), ('bad', 'lama1277-lamak', 'mədõ')}
 {('bad', 'lama1277-baipi', 'maehala')}

  Just for clarity:
  There are also the following similarity classes shared between Alorese and LH.
  {('bad', 'keda1252', 'daten'), ('bad', 'lama1277-lewoe', 'datẽ'), ('bad', 'alor1247-pandai', 'datɛ'), ('bad', 'alor1247-besar', 'datɛ'), ('bad', 'lama1277-lewot', 'kədatək'), ('bad', 'lama1277-lamah', 'daʔa'), ('bad', 'lama1277-imulo', 'kədatekən'), ('bad', 'lama1277-belan', 'kədatək'), ('bad', 'lama1277-waiba', 'datə'), ('bad', 'lama1277-lamat', 'datʔẽ'), ('bad', 'lama1277-lewuk', 'kədatək'), ('bad', 'lama1277-merde', 'datã'), ('bad', 'lama1277-ritae', 'datã'), ('bad', 'alor1247-baran', 'datɛ'), ('bad', 'lama1277-lewok', 'kədatək'), ('bad', 'lama1277-ileap', 'fedatən'), ('bad', 'lama1277-tanju', 'datã'), ('bad', 'lama1277-kalik', 'kədatək'), ('bad', 'keda1252-leuwa', 'datεn ɔhaʔ'), ('bad', 'lama1277-lamal', 'afadatəŋ'), ('bad', 'keda1252-leuba', "da'tɛn"), ('bad', 'lama1277-kalik', 'kəˈdatək')}


Concept ‘meeting_house’ is different between Alorese and Lamaholot.

 Alorese innovated the following forms:
 {('meeting_house', 'alor1247-pandai', 'lada')}
 {('meeting_house', 'alor1247-besar', "pusuŋ 'rɛbɔŋ")}
 {('meeting_house', 'alor1247-besar', "uma 'hada")}
 {('meeting_house', 'alor1247-pandai', "m'nɔhi")}
 Alorese shares the following forms with other AN languanges, but not Lamaholot:
 {('meeting_house', 'tetu1245-suai', "uma 'lulik"), ('meeting_house', 'alor1247-munas', "uma 'lɛlaŋ ")}
 One or more Lamaholot dialects innovated forms not found in Alorese:
 {('meeting_house', 'lama1277-kalik', 'ˈuna keˈdak')}
 {('meeting_house', 'lama1277-lerek', 'ˈunə ˈrajan')}

  Just for clarity:
  There are also the following similarity classes shared between Alorese and LH.
  {('meeting_house', 'rett1240', 'kɔkɔr'), ('meeting_house', 'lama1277-lewoi', 'ˈkɔkə ˈbale'), ('meeting_house', 'alor1247-pandai', 'kɔkɔr'), ('meeting_house', 'lama1277-adona', 'ˈkokɜ')}


Concept ‘to_pull’ is different between Alorese and Lamaholot.

 Alorese innovated the following forms:
 {('to_pull', 'kira1248', 'gabɪk'), ('to_pull', 'blag1240-kulij', 'awak'), ('to_pull', 'alor1247-pandai', 'wak'), ('to_pull', 'kelo1247-bring', 'gəbɪk'), ('to_pull', 'abui1241-takal', "hä.'fik"), ('to_pull', 'kelo1247-hopte', "gə'bik"), ('to_pull', 'abui1241-ulaga', 'gavik'), ('to_pull', 'blag1240-nule', 'avak'), ('to_pull', 'abui1241-ulaga', 'gafik'), ('to_pull', 'kafo1240', 'gɑfɪki'), ('to_pull', 'atim1239', 'ha-fik'), ('to_pull', 'abui1241-fuime', 'ha-fik'), ('to_pull', 'kuii1253', 'gabik'), ('to_pull', 'baka1276', "a'vak")}
 {('to_pull', 'alor1247-pandai', "ta'rːe"), ('to_pull', 'alor1247-baran', 'tar:ɛ'), ('to_pull', 'alor1247-munas', "tɛ'rːɛʔ"), ('to_pull', 'alor1247-besar', 'tarːɛ')}
 Alorese shares the following forms with TAP languages:
 {('to_pull', 'kira1248', 'gabɪk'), ('to_pull', 'blag1240-kulij', 'awak'), ('to_pull', 'alor1247-pandai', 'wak'), ('to_pull', 'kelo1247-bring', 'gəbɪk'), ('to_pull', 'abui1241-takal', "hä.'fik"), ('to_pull', 'kelo1247-hopte', "gə'bik"), ('to_pull', 'abui1241-ulaga', 'gavik'), ('to_pull', 'blag1240-nule', 'avak'), ('to_pull', 'abui1241-ulaga', 'gafik'), ('to_pull', 'kafo1240', 'gɑfɪki'), ('to_pull', 'atim1239', 'ha-fik'), ('to_pull', 'abui1241-fuime', 'ha-fik'), ('to_pull', 'kuii1253', 'gabik'), ('to_pull', 'baka1276', "a'vak")}
 {('to_pull', 'alor1247-pandai', "ta'rːe"), ('to_pull', 'alor1247-baran', 'tar:ɛ'), ('to_pull', 'alor1247-munas', "tɛ'rːɛʔ"), ('to_pull', 'alor1247-besar', 'tarːɛ')}
 One or more Lamaholot dialects innovated forms not found in Alorese:
 {('to_pull', 'idat1237', "a'dada"), ('to_pull', 'tetu1245-vique', 'dada'), ('to_pull', 'mamb1306', 'dad'), ('to_pull', 'tetu1246', 'dada'), ('to_pull', 'blag1240-bama', 'tedo'), ('to_pull', 'laka1255', 'dada'), ('to_pull', 'tetu1245-suai', 'dada'), ('to_pull', 'lama1277-lewom', 'eta'), ('to_pull', 'blag1240-warsa', 'tedo'), ('to_pull', 'blag1240-tuntu', 'tedoh')}
 {('to_pull', 'lama1277-lewot', 'gəfea'), ('to_pull', 'lama1277-merde', 'gihã'), ('to_pull', 'lama1277-lewob', 'gehaʔ'), ('to_pull', 'lama1277-tanju', 'gehe'), ('to_pull', 'lama1277-pukau', 'gehã'), ('to_pull', 'lama1277-adona', 'ˈɡehã'), ('to_pull', 'lama1277-baipi', 'gehã'), ('to_pull', 'lama1277-lamal', 'gəfea'), ('to_pull', 'lama1277-lewog', 'gehã'), ('to_pull', 'lama1277-horow', 'gehã'), ('to_pull', 'lama1277-waiwa', 'geã'), ('to_pull', 'lama1277-lewoi', 'ˈɡehɑŋ'), ('to_pull', 'lama1277-waiba', 'gehã'), ('to_pull', 'lama1277-bama', 'gehẽ')}
 {('to_pull', 'lama1277-lewok', 'tugu'), ('to_pull', 'lama1277-kalik', 'tugu'), ('to_pull', 'lama1277-watan', 'tubo'), ('to_pull', 'lama1277-minga', 'tubu'), ('to_pull', 'lama1277-kiwan', 'tubo'), ('to_pull', 'lama1277-lamah', 'tubo'), ('to_pull', 'lama1277-belan', 'tubo'), ('to_pull', 'lama1277-adona', 'ˈtubo'), ('to_pull', 'lama1277-mulan', 'tubo'), ('to_pull', 'lama1277-wuake', 'tubo'), ('to_pull', 'lama1277-lamak', 'tubo'), ('to_pull', 'lama1277-lewuk', 'tugu'), ('to_pull', 'lama1277-paina', 'tugo'), ('to_pull', 'lama1277-lerek', 'ˈtuɡo'), ('to_pull', 'lama1277-botun', 'tubo'), ('to_pull', 'lama1277-ileap', 'tubo')}

  Just for clarity:
  There are also the following similarity classes shared between Alorese and LH.
  {('to_pull', 'alor1247-pandai', 'wider'), ('to_pull', 'lama1277-dulhi', 'wəda'), ('to_pull', 'lama1277-kalik', 'ˈvidu'), ('to_pull', 'lamm1241-westp', 'bid:i'), ('to_pull', 'lama1277-lamat', 'gidə'), ('to_pull', 'lama1277-ritae', 'widu'), ('to_pull', 'sika1262-tanai', 'ɡide'), ('to_pull', 'lama1277-lewoe', 'idɔ'), ('to_pull', 'lama1277-lewop', 'fidu'), ('to_pull', 'lama1277-imulo', 'fido'), ('to_pull', 'sika1262-hewa', 'ide')}


Concept ‘spine_lower_back’ is different between Alorese and Lamaholot.

 Alorese innovated the following forms:
 {('spine_lower_back', 'alor1247-pandai', "punuŋ 'kajuŋ"), ('spine_lower_back', 'alor1247-besar', "punuŋ 'kajɔŋ")}
 {('spine_lower_back', 'pura1258', 'taha waŋ'), ('spine_lower_back', 'abui1241-ulaga', 'tapiaŋ'), ('spine_lower_back', 'pura1258', 'taha baŋ'), ('spine_lower_back', 'alor1247-munas', "hie 'beiŋ"), ('spine_lower_back', 'abui1241-ulaga', 'tapiaŋ tapai')}
 Alorese shares the following forms with TAP languages:
 {('spine_lower_back', 'alor1247-pandai', "punuŋ 'kajuŋ"), ('spine_lower_back', 'alor1247-besar', "punuŋ 'kajɔŋ")}
 {('spine_lower_back', 'pura1258', 'taha waŋ'), ('spine_lower_back', 'abui1241-ulaga', 'tapiaŋ'), ('spine_lower_back', 'pura1258', 'taha baŋ'), ('spine_lower_back', 'alor1247-munas', "hie 'beiŋ"), ('spine_lower_back', 'abui1241-ulaga', 'tapiaŋ tapai')}
 One or more Lamaholot dialects innovated forms not found in Alorese:
 {('spine_lower_back', 'lama1277-lerek', 'wuˈhuk kaˈjuŋ')}
 {('spine_lower_back', 'lama1277-adona', 'ˈhɜlɜrɜt')}
 {('spine_lower_back', 'lama1277-kalik', 'kəˈnaru riˈuk')}

  Just for clarity:
  There are also the following similarity classes shared between Alorese and LH.
  {('spine_lower_back', 'alor1247-besar', 'alɛ̃'), ('spine_lower_back', 'lama1277-lewoi', 'ˈalɛŋ')}


Concept ‘seed’ is different between Alorese and Lamaholot.

 One or more Lamaholot dialects innovated forms not found in Alorese:
 {('seed', 'lama1277-lerek', 'ˈriuk'), ('seed', 'lama1277-lewot', 'riuk'), ('seed', 'lama1277-kalik', 'riˈuk')}
 {('seed', 'lama1277-lewom', 'ərã')}
 {('seed', 'lama1277-ritae', 'lõhõ')}
 {('seed', 'lama1277-lamal', 'upu'), ('seed', 'lamm1241-westp', 'up:ur'), ('seed', 'lama1277-lamat', 'upu')}

  Just for clarity:
  There are also the following similarity classes shared between Alorese and LH.
  {('seed', 'lama1277-lamak', 'kulʔũ'), ('seed', 'blag1240-kulij', 'kulu'), ('seed', 'lama1277-adona', 'ˈkulʔũ'), ('seed', 'lama1277-botun', 'kulʔũ'), ('seed', 'keda1252-leuba', "ʔu'luʔ"), ('seed', 'keda1252', 'ulu?'), ('seed', 'blag1240-nule', 'te kulu'), ('seed', 'lama1277-ileap', 'kuluk'), ('seed', 'lama1277-merde', 'kulʔũ'), ('seed', 'lama1277-paina', 'kulukən'), ('seed', 'alor1247-munas', 'kuluŋ'), ('seed', 'lama1277-lewok', 'kuluk'), ('seed', 'lama1277-lewob', 'kulu'), ('seed', 'alor1247-besar', "ku'lukuŋ"), ('seed', 'lama1277-kiwan', 'kulʔũ'), ('seed', 'lama1277-waiwa', 'kəluʔũ'), ('seed', 'lama1277-lamah', 'kulũ'), ('seed', 'lama1277-lewog', 'kulʔũ'), ('seed', 'lama1277-dulhi', 'kulʔũ'), ('seed', 'lama1277-lewoe', 'ʔulũ'), ('seed', 'lama1277-mulan', 'kənuluk'), ('seed', 'lama1277-bama', 'kuluʔ'), ('seed', 'lama1277-tanju', 'kuluʔ'), ('seed', 'lama1277-baipi', 'kuluk'), ('seed', 'lama1277-horow', 'kəlulũ'), ('seed', 'alor1247-pandai', 'kuluŋ'), ('seed', 'lama1277-lewoi', 'ˈkuluŋ'), ('seed', 'lama1277-lewuk', 'kuluk'), ('seed', 'lama1277-wuake', 'kulɁu'), ('seed', 'lama1277-watan', 'kulʔũ'), ('seed', 'lama1277-kalik', 'kuluk'), ('seed', 'lama1277-lewop', 'kuluk'), ('seed', 'alor1247-baran', 'kuluŋ'), ('seed', 'lama1277-waiba', 'kənuluʔ'), ('seed', 'lama1277-imulo', 'kuluk'), ('seed', 'keda1252-leuwa', 'uluʔ'), ('seed', 'lama1277-belan', 'kulu'), ('seed', 'lama1277-minga', 'kuluk'), ('seed', 'lama1277-pukau', 'kuluʔũ')}


Concept ‘river_stream’ is different between Alorese and Lamaholot.

 Alorese shares the following forms with TAP languages:
 {('river_stream', 'kafo1240', 'kulɑ'), ('river_stream', 'alor1247-baran', 'kali')}
 One or more Lamaholot dialects innovated forms not found in Alorese:
 {('river_stream', 'lama1277-lerek', 'ˈwei raˈjan')}
 {('river_stream', 'lama1277-watan', 'suŋe'), ('river_stream', 'lama1277-lewom', 'suŋe'), ('river_stream', 'lama1277-ritae', 'suŋe'), ('river_stream', 'lama1277-pukau', 'suŋe'), ('river_stream', 'lama1277-lewog', 'suŋe'), ('river_stream', 'lama1277-adona', 'suŋe'), ('river_stream', 'lama1277-bama', 'suŋe'), ('river_stream', 'lama1277-lamal', 'suŋe')}
 {('river_stream', 'keda1252-leuba', 'wɛi'), ('river_stream', 'lama1277-horow', 'wai'), ('river_stream', 'lama1277-dulhi', 'wai'), ('river_stream', 'kaer1234', 'ir boi'), ('river_stream', 'lama1277-paina', 'wai'), ('river_stream', 'lama1277-lewop', 'fai'), ('river_stream', 'kelo1247-bring', 'wɛr'), ('river_stream', 'lama1277-waiwa', 'wai'), ('river_stream', 'kelo1247-hopte', 'wɛr'), ('river_stream', 'lama1277-minga', 'wai'), ('river_stream', 'lama1277-mulan', 'wai'), ('river_stream', 'lama1277-lewot', 'fai mənaŋaken'), ('river_stream', 'maka1316', 'ira wair'), ('river_stream', 'lama1277-kiwan', 'wai maʔã'), ('river_stream', 'lama1277-baipi', 'wai ba'), ('river_stream', 'fata1247', 'ira wɛr'), ('river_stream', 'sika1262-hewa', 'wair ba'), ('river_stream', 'lama1277-waiba', 'lũ wãi'), ('river_stream', 'lama1277-kalik', 'waer'), ('river_stream', 'lama1277-lewuk', 'waer'), ('river_stream', 'sika1262-tanai', 'βair ba'), ('river_stream', 'lama1277-ileap', 'wai nahan'), ('river_stream', 'lama1277-botun', 'wai maʔã'), ('river_stream', 'lama1277-imulo', 'fai'), ('river_stream', 'p-mala1545-acd', '*wahiR')}
 {('river_stream', 'blag1240-tuntu', 'bola'), ('river_stream', 'blag1240-warsa', 'bolaʔ'), ('river_stream', 'nede1245', 'balu'), ('river_stream', 'blag1240-bama', 'bola'), ('river_stream', 'lama1277-lewok', 'baku')}
 {('river_stream', 'lama1277-kalik', 'əˈbak'), ('river_stream', 'p-mala1545-acd', '*sapaq')}
 {('river_stream', 'sika1262-hewa', 'napun'), ('river_stream', 'sika1262-tanai', 'naŋa ba'), ('river_stream', 'lama1277-lamak', 'waimənaʔaŋ')}

  Just for clarity:
  There are also the following similarity classes shared between Alorese and LH.
  {('river_stream', 'alor1247-munas', 'aluk'), ('river_stream', 'lama1277-lamah', 'luŋu'), ('river_stream', 'lama1277-belan', 'luŋu'), ('river_stream', 'lama1277-lamat', 'luŋu'), ('river_stream', 'lama1277-lewoe', 'luŋu'), ('river_stream', 'alor1247-pandai', 'alu'), ('river_stream', 'lama1277-tanju', 'luŋu'), ('river_stream', 'lama1277-wuake', 'luŋu'), ('river_stream', 'lama1277-lewoi', 'ˈluŋuŋ'), ('river_stream', 'lama1277-merde', 'luŋu'), ('river_stream', 'alor1247-besar', 'alu'), ('river_stream', 'lama1277-lewob', 'luŋu')}


Concept ‘thunder’ is different between Alorese and Lamaholot.

 Alorese shares the following forms with other AN languanges, but not Lamaholot:
 {('thunder', 'alor1247-munas', "hilaʔ brə'kaŋ"), ('thunder', 'alor1247-pandai', 'hila'), ('rainbow', 'keda1252-leuba', 'tado'), ('rainbow', 'keda1252-leuba', 'nado'), ('thunder', 'alor1247-besar', 'hila')}
 One or more Lamaholot dialects innovated forms not found in Alorese:
 {('thunder', 'sarr1247-adiab', 'gragar'), ('thunder', 'lama1277-adona', "kɜ'lɜka"), ('thunder', 'sarr1247-nule', 'gregaj'), ('thunder', 'lama1277-kalik', 'kələˈɡor'), ('thunder', 'blag1240-bama', "ge'lagar"), ('thunder', 'rett1240', 'galagal'), ('thunder', 'lama1277-lerek', 'ˈkləkə'), ('thunder', 'blag1240-kulij', 'lal'), ('thunder', 'blag1240-nule', 'lal'), ('thunder', 'blag1240-tuntu', "gə'lagar"), ('thunder', 'rett1240', "ga'lagal"), ('thunder', 'kira1248', 'paka kal'), ('thunder', 'kuii1253', 'galagal'), ('thunder', 'sika1262-hewa', 'kleka liar'), ('thunder', 'baka1276', 'lal')}
 {('thunder', 'lama1277-lewoi', 'pələˈdə')}


Concept ‘stranger’ is different between Alorese and Lamaholot.

 Alorese innovated the following forms:
 {('stranger', 'alor1247-pandai', 'mesia mata puna'), ('stranger', 'alor1247-besar', "ata mata'puna"), ('stranger', 'alor1247-pandai', "ata 'mata 'puna")}
 {('stranger', 'alor1247-munas', "ata 'laraŋ 'doaŋ")}
 One or more Lamaholot dialects innovated forms not found in Alorese:
 {('stranger', 'lama1277-lewoi', 'ˈata bəʔaˈhɑŋ'), ('stranger', 'lama1277-kalik', 'ˈata  ɡeˈsak'), ('stranger', 'lama1277-lerek', 'ˈata ɡehaˈkən')}
 {('stranger', 'lama1277-adona', 'muriˈke')}


Concept ‘to_vomit’ is different between Alorese and Lamaholot.

 Alorese shares the following forms with TAP languages:
 {('to_vomit', 'rett1240', 'igir'), ('to_vomit', 'kaer1234', "g-i'gir"), ('to_vomit', 'teiw1235', "ji'gar"), ('to_vomit', 'buna1278-bobon', 'khakhal'), ('to_vomit', 'dein1238', 'jig:it'), ('to_vomit', 'rett1240', 'ʔigir'), ('to_vomit', 'kuii1253', 'karí'), ('to_vomit', 'sarr1247-nule', 'ʤigit'), ('to_vomit', 'sarr1247-adiab', 'ʤigit'), ('to_vomit', 'blag1240-tuntu', 'jigeda'), ('to_vomit', 'alor1247-munas', "k'bɔr"), ('to_vomit', 'buna1278-malia', 'khɑkhɑl')}
 {('to_vomit', 'blag1240-bama', 'boras'), ('to_vomit', 'alor1247-munas', 'bɔras')}
 One or more Lamaholot dialects innovated forms not found in Alorese:
 {('to_vomit', 'lama1277-lewuk', 'lua'), ('to_vomit', 'lama1277-paina', 'luag'), ('to_vomit', 'lama1277-kalik', 'luaŋ'), ('to_vomit', 'lama1277-belan', 'lua'), ('to_vomit', 'lama1277-lamat', 'luã'), ('to_vomit', 'lama1277-lewop', 'lua'), ('to_vomit', 'lama1277-wuake', 'lua'), ('to_vomit', 'lama1277-imulo', 'luaŋa'), ('to_vomit', 'lama1277-lewot', 'luaŋa'), ('to_vomit', 'lama1277-lewok', 'lua'), ('to_vomit', 'lama1277-lerek', 'ˈluaŋ'), ('to_vomit', 'lama1277-mulan', 'luə'), ('to_vomit', 'lama1277-horow', 'luʔuk'), ('to_vomit', 'lama1277-minga', 'lua'), ('to_vomit', 'lama1277-kalik', 'luˈa'), ('to_vomit', 'lama1277-merde', 'luã')}
 {('to_vomit', 'sika1262-tanai', 'doʔuk'), ('to_vomit', 'lama1277-adona', 'kɜˈdɔʔuk')}

  Just for clarity:
  There are also the following similarity classes shared between Alorese and LH.
  {('to_vomit', 'sika1262-hewa', 'mutaʔ'), ('to_vomit', 'lama1277-botun', 'muta'), ('to_vomit', 'laka1255', 'muta'), ('to_vomit', 'tetu1245-suai', 'muta'), ('to_vomit', 'kema1243', 'muta'), ('to_vomit', 'p-mala1545-acd', '*um-utaq'), ('to_vomit', 'lama1277-watan', 'muta'), ('to_vomit', 'keda1252', 'mute'), ('to_vomit', 'lama1277-bama', 'tani'), ('to_vomit', 'lama1277-dulhi', 'muta'), ('to_vomit', 'alor1247-baran', 'mut:a'), ('to_vomit', 'lama1277-ileap', 'muta'), ('to_vomit', 'tetu1246', 'muta'), ('to_vomit', 'p-mala1545-acd', '*utaq'), ('to_vomit', 'lama1277-lamah', 'mutakə'), ('to_vomit', 'alor1247-pandai', 'muta'), ('to_vomit', 'lama1277-lewom', 'muta'), ('to_vomit', 'lama1277-ritae', 'muta'), ('to_vomit', 'alor1247-munas', 'mutaʔ'), ('to_vomit', 'alor1247-munas', 'mutaŋ'), ('to_vomit', 'lama1277-lewog', 'muta'), ('to_vomit', 'lama1277-kiwan', 'muta'), ('to_vomit', 'lama1277-lewoe', 'mu'), ('to_vomit', 'p-aust1307-abvd', '*utaq'), ('to_vomit', 'adan1251-lawah', 'wudi'), ('to_vomit', 'alor1247-besar', 'muta'), ('to_vomit', 'lama1277-lewob', 'muta'), ('to_vomit', 'lama1277-pukau', 'muta'), ('to_vomit', 'lama1277-baipi', 'muta'), ('to_vomit', 'hama1240', 'ʊid'), ('to_vomit', 'alor1247-pandai', 'mutaŋ'), ('to_vomit', 'p-mala1545-abvd', '*utaq'), ('to_vomit', 'p-cent2245-abvd', '*mutaq'), ('to_vomit', 'lama1277-lewoi', 'ˈmutaʔ'), ('to_vomit', 'lama1277-waiba', 'muta'), ('to_vomit', 'mamb1306', 'mu:t'), ('to_vomit', 'sika1262-maume', 'mut̪a'), ('to_vomit', 'sika1262-tanai', 'muta'), ('to_vomit', 'tuku1254', 'mu:t'), ('to_vomit', 'lama1277-lamal', 'muta'), ('to_vomit', 'tetu1245-vique', 'muta'), ('to_vomit', 'lama1277-tanju', 'muta'), ('to_vomit', 'lama1277-waiwa', 'muta'), ('to_vomit', 'lama1277-lamak', 'muta')}


Concept ‘uncooked_rice’ is different between Alorese and Lamaholot.

 Alorese innovated the following forms:
 {('uncooked_rice', 'alor1247-munas', "wata 'pare"), ('uncooked_rice', 'alor1247-munas', 'pare'), ('uncooked_rice', 'alor1247-besar', 'pari'), ('uncooked_rice', 'alor1247-pandai', 'parɛ')}
 {('uncooked_rice', 'alor1247-baran', 'apa')}
 One or more Lamaholot dialects innovated forms not found in Alorese:
 {('uncooked_rice', 'lama1277-adona', 'ˈwahaʔ')}
 {('uncooked_rice', 'lama1277-kalik', 'ˈlisɔ daˈnen')}
 {('uncooked_rice', 'lama1277-lewoi', 'ˈtahaŋ ˈlɔhɔŋ')}


Concept cooked_rice is attested only in Lamaholot


Concept ‘because’ is different between Alorese and Lamaholot.

 Alorese innovated the following forms:
 {('because', 'alor1247-pandai', 'karna'), ('because', 'alor1247-baran', 'karɛna')}
 {('because', 'alor1247-besar', "ɛ'tɛmuŋ")}
 One or more Lamaholot dialects innovated forms not found in Alorese:
 {('because', 'lama1277-lerek', 'məˈki')}
 {('because', 'lama1277-lewom', 'pukẽ'), ('because', 'lama1277-lamah', 'pukã'), ('because', 'lama1277-dulhi', 'pukã'), ('because', 'lama1277-baipi', 'pukã'), ('because', 'lama1277-lewoi', 'ˈpukəŋ'), ('because', 'lama1277-lamat', 'puʔẽ'), ('because', 'lama1277-lewuk', 'pukən'), ('because', 'lama1277-watan', 'pukã'), ('because', 'lama1277-lewog', 'pukən'), ('because', 'lama1277-lewot', 'puk'), ('because', 'lama1277-waiba', 'pukã'), ('because', 'lama1277-paina', 'pukən'), ('because', 'lama1277-imulo', 'puka'), ('because', 'lama1277-lewop', 'pukã'), ('because', 'lama1277-lamak', 'pukã'), ('because', 'lama1277-adona', 'ˈpukɜ'), ('because', 'lama1277-belan', 'puk'), ('because', 'lama1277-lamal', 'pukãŋ'), ('because', 'lama1277-minga', 'pukɤ'), ('because', 'sika1262-hewa', 'puʔan poi'), ('because', 'lama1277-bama', 'pukã'), ('because', 'lama1277-merde', 'pukã'), ('because', 'lama1277-tanju', 'pukã'), ('because', 'lama1277-kalik', 'pukən'), ('because', 'lama1277-lewok', 'puk'), ('because', 'lama1277-mulan', 'pukə'), ('because', 'lama1277-lewob', 'pukã'), ('because', 'lama1277-horow', 'pukã'), ('because', 'lama1277-kalik', 'ˈpukən'), ('because', 'lama1277-ileap', 'pukən'), ('because', 'lama1277-wuake', 'puka'), ('because', 'lama1277-pukau', 'pukə̃'), ('because', 'lama1277-botun', 'pukã'), ('because', 'lama1277-waiwa', 'pukã'), ('because', 'lama1277-ritae', 'pukã')}

  Just for clarity:
  There are also the following similarity classes shared between Alorese and LH.
  {('because', 'lama1277-kiwan', 'səbab'), ('because', 'alor1247-baran', "sɛ'bab")}


Concept ‘to_drop’ is different between Alorese and Lamaholot.

 One or more Lamaholot dialects innovated forms not found in Alorese:
 {('to_drop', 'lama1277-lerek', 'ˈlokaŋ')}
 {('to_drop', 'lama1277-lewoi', 'ləˈsaː')}
 {('to_drop', 'lama1277-kalik', 'məˈra ləˈŋata')}
 {('to_drop', 'lama1277-lerek', 'paˈhoˈləŋ'), ('to_drop', 'lama1277-kalik', 'pəˈsola')}

  Just for clarity:
  There are also the following similarity classes shared between Alorese and LH.
  {('to_drop', 'koto1251', 'na-kua'), ('to_drop', 'alor1247-pandai', "lɛlaŋ 'gɔkal"), ('to_drop', 'alor1247-besar', "naŋ 'gɔka"), ('to_drop', 'alor1247-baran', "nĩ: 'gɔka"), ('to_drop', 'lama1277-adona', 'na ˈɡoka'), ('to_drop', 'alor1247-munas', "lelaŋ 'gɔkal")}


Concept ‘rice_plant’ is different between Alorese and Lamaholot.

 Alorese innovated the following forms:
 {('rice_plant', 'alor1247-munas', "pare 'nawaŋ"), ('rice_plant', 'alor1247-pandai', 'parɛ'), ('rice_plant', 'alor1247-besar', 'pari')}
 One or more Lamaholot dialects innovated forms not found in Alorese:
 {('rice_plant', 'lama1277-kalik', 'liˈsɔ'), ('rice_plant', 'lama1277-kalik', 'liˈsɔr')}
 {('rice_plant', 'lama1277-lewoi', 'ˈtahaʔɑŋ')}
 {('rice_plant', 'lama1277-adona', 'ˈwahaʔ')}
 {('rice_plant', 'lama1277-lerek', 'kəˈluok kaˈmak')}


Concept ‘grasshopper’ is different between Alorese and Lamaholot.

 One or more Lamaholot dialects innovated forms not found in Alorese:
 {('grasshopper', 'lama1277-lewoi', 'kəˈtoko')}

  Just for clarity:
  There are also the following similarity classes shared between Alorese and LH.
  {('grasshopper', 'lama1277-kalik', 'maˈʤu'), ('grasshopper', 'alor1247-besar', "ka'marɔ"), ('grasshopper', 'lama1277-lerek', 'maˈyor'), ('grasshopper', 'alor1247-munas', 'marɔʔ'), ('grasshopper', 'alor1247-pandai', 'marɔ'), ('grasshopper', 'lama1277-kalik', 'maˈʤɔr'), ('grasshopper', 'lama1277-adona', 'kɜˈmaroʔ'), ('grasshopper', 'alor1247-pandai', "k'marɔ")}


Concept ‘dust’ is different between Alorese and Lamaholot.

 One or more Lamaholot dialects innovated forms not found in Alorese:
 {('dust', 'lama1277-baipi', 'nuhũ')}
 {('dust', 'lama1277-kalik', 'əmut'), ('dust', 'lama1277-adona', 'ʔɜˈmut'), ('dust', 'lama1277-kalik', 'əˈmʊt'), ('dust', 'lama1277-belan', 'əmut'), ('dust', 'lama1277-ileap', 'əmut'), ('dust', 'lama1277-lewuk', 'əmut'), ('dust', 'lama1277-lewok', 'amutu'), ('dust', 'lama1277-lewot', 'əmutu'), ('dust', 'lama1277-kiwan', 'əmut'), ('dust', 'lama1277-merde', 'əmut'), ('dust', 'lama1277-botun', 'əmut'), ('dust', 'lama1277-lerek', 'ˈəmut'), ('dust', 'lama1277-lamak', 'əmu'), ('dust', 'lama1277-lamal', 'əmut'), ('dust', 'lama1277-watan', 'əmut'), ('dust', 'lama1277-wuake', 'əmut'), ('dust', 'lama1277-imulo', 'əmut'), ('dust', 'lama1277-horow', 'əmut'), ('dust', 'lama1277-waiwa', 'əmut'), ('dust', 'lama1277-paina', 'əmut'), ('dust', 'lama1277-lamat', 'əmu'), ('dust', 'lama1277-minga', 'əmutu'), ('dust', 'lama1277-lamah', 'əmut'), ('dust', 'lama1277-dulhi', 'əmut'), ('dust', 'lama1277-lewop', 'əmut'), ('dust', 'lama1277-mulan', 'əmu')}
 {('dust', 'lama1277-lamat', 'oʔɔ')}

  Just for clarity:
  There are also the following similarity classes shared between Alorese and LH.
  {('dust', 'lama1277-lewom', 'kəawuk'), ('dust', 'sika1262-hewa', 'awu'), ('dust', 'lama1277-dulhi', 'kawuk'), ('dust', 'lama1277-baipi', 'kəawuk'), ('dust', 'lama1277-pukau', 'kəwuk'), ('dust', 'lama1277-ritae', 'kawuk'), ('dust', 'lama1277-minga', 'kawok'), ('dust', 'sika1262-hewa', 'aʋu'), ('dust', 'keda1252', 'ewol'), ('dust', 'lama1277-lewob', 'kəwu'), ('dust', 'lama1277-tanju', 'kəawuk'), ('dust', 'lama1277-lewob', 'əwu'), ('dust', 'alor1247-baran', "a'fo:"), ('dust', 'lama1277-lamak', 'kaʔwuk'), ('dust', 'p-mala1545-acd', '*ebuk'), ('dust', 'p-mala1545-acd', '*habuk'), ('dust', 'lama1277-merde', 'kawu'), ('dust', 'lama1277-lewoe', 'ʔawɔ'), ('dust', 'lama1277-kiwan', 'kəʔawuk'), ('dust', 'lama1277-ileap', 'awo'), ('dust', 'lama1277-lewog', 'kəʔawuk'), ('dust', 'lama1277-lewok', 'kawok'), ('dust', 'lama1277-bama', 'kəawuk'), ('dust', 'lama1277-lewot', 'kafoku'), ('dust', 'lama1277-imulo', 'kafok'), ('dust', 'lama1277-lewoi', 'kəˈʔawu'), ('dust', 'lama1277-lewoe', 'awo'), ('dust', 'lama1277-lewog', 'keawuk'), ('dust', 'sika1262-tanai', 'ʔreβu'), ('dust', 'p-mala1545-abvd', '*qapuk'), ('dust', 'lama1277-botun', 'kəawuk'), ('dust', 'lama1277-horow', 'kəʔawuk'), ('dust', 'lama1277-lewuk', 'kawok'), ('dust', 'lama1277-mulan', 'kawu'), ('dust', 'lama1277-waiwa', 'kəʔawuk'), ('dust', 'alor1247-besar', 'afɔ'), ('dust', 'lama1277-lamah', 'kawu'), ('dust', 'p-mala1545-abvd', '*qabuk'), ('dust', 'lama1277-bama', 'kʔawuk'), ('dust', 'tuku1254', 'ʔahu'), ('dust', 'lama1277-lamal', 'kafu'), ('dust', 'lama1277-waiba', 'kəawuk'), ('dust', 'lama1277-ritae', 'awo'), ('dust', 'lama1277-kalik', 'kawok'), ('dust', 'sika1262-hewa', 'pua'), ('dust', 'lama1277-watan', 'kəʔawuk'), ('dust', 'p-cent2245-abvd', '*qabu'), ('dust', 'alor1247-munas', 'krawuʔ'), ('dust', 'alor1247-pandai', "kə'rawu"), ('dust', 'p-mala1545-acd', '*qabuk'), ('dust', 'lama1277-paina', 'kawok'), ('dust', 'lama1277-belan', 'kawuk'), ('dust', 'lama1277-wuake', 'kawok'), ('dust', 'lama1277-lewop', 'kafoku')}


Concept ‘storage_house’ is different between Alorese and Lamaholot.

 Alorese innovated the following forms:
 {('storage_house', 'alor1247-pandai', 'lada'), ('storage_house', 'alor1247-munas', 'ladaʔ')}
 {('storage_house', 'alor1247-pandai', 'nɔhi'), ('storage_house', 'alor1247-pandai', "mə'nɔhi")}
 {('storage_house', 'alor1247-besar', "uma 'lapuŋ")}
 One or more Lamaholot dialects innovated forms not found in Alorese:
 {('storage_house', 'lamm1241-westp', 'kebaŋ'), ('storage_house', 'keda1252-leuba', 'ʔebaŋ'), ('storage_house', 'dein1238', 'qebaŋ'), ('storage_house', 'lama1277-lewoi', 'ˈkɛbɑŋ')}
 {('storage_house', 'lama1277-kalik', 'veˈtak'), ('storage_house', 'lama1277-lerek', 'ˈwetak')}
 {('storage_house', 'lama1277-adona', 'ˈɡudã'), ('storage_house', 'abui1241-fuime', 'kadaŋ'), ('storage_house', 'blag1240-warsa', 'kuruŋ'), ('storage_house', 'blag1240-tuntu', 'kuruŋ'), ('storage_house', 'atim1239', 'kudaŋ'), ('storage_house', 'kaer1234', "ku'ruŋ"), ('storage_house', 'kira1248', 'gudan'), ('storage_house', 'abui1241-ulaga', 'gudaŋ'), ('storage_house', 'atim1239', 'kadaŋ'), ('storage_house', 'blag1240-bama', 'kuru')}
 {('storage_house', 'pura1258', 'mɛraŋ'), ('storage_house', 'sika1262-hewa', 'orin'), ('storage_house', 'rett1240', 'melaŋ'), ('storage_house', 'lama1277-lewoi', 'ˈɔriŋ'), ('storage_house', 'blag1240-kulij', 'meraŋ'), ('storage_house', 'rett1240', 'me:laŋ')}


Concept ‘to_open’ is different between Alorese and Lamaholot.

 Alorese innovated the following forms:
 {('to_open', 'alor1247-besar', 'hioka')}
 {('to_open', 'alor1247-munas', 'loe')}
 Alorese shares the following forms with other AN languanges, but not Lamaholot:
 {('to_open', 'kula1280-lanto', 'lə’ka'), ('to_open', 'maka1316', 'lɔke'), ('to_open', 'tetu1245-suai', 'lɔke'), ('to_open', 'alor1247-pandai', 'lɛka'), ('to_open', 'sawi1256', 'laka'), ('to_open', 'alor1247-munas', 'leka'), ('to_open', 'wers1238-taram', 'lak'), ('to_open', 'blag1240-warsa', 'legi'), ('to_open', 'tetu1245-vique', 'lɔke'), ('to_open', 'wers1238-marit', 'lak')}
 One or more Lamaholot dialects innovated forms not found in Alorese:
 {('to_open', 'lama1277-kalik', 'biˈtɔl')}

  Just for clarity:
  There are also the following similarity classes shared between Alorese and LH.
  {('to_open', 'lama1277-lewoi', 'ˈbukaʔ'), ('to_open', 'p-mala1545-acd', '*buka'), ('to_open', 'blag1240-nule', 'bakuŋ'), ('to_open', 'blag1240-kulij', 'bakuŋ'), ('to_open', 'keda1252-leuba', "bu'kaʔ"), ('to_open', 'alor1247-besar', 'buka'), ('to_open', 'p-cent2245-abvd', '*buka'), ('to_open', 'p-mala1545-abvd', '*buka'), ('to_open', 'p-mala1545-acd', '*bu(ŋ)kas'), ('to_open', 'lama1277-lerek', 'bukəˈtəŋ'), ('to_open', 'kama1365', 'pilah'), ('to_open', 'lama1277-adona', 'ˈbuka')}


Concept ‘to_breathe’ is different between Alorese and Lamaholot.

 Alorese shares the following forms with TAP languages:
 {('to_breathe', 'alor1247-munas', 'hɔpaŋ'), ('to_breathe', 'lamm1241-westp', 'hopaŋ'), ('to_breathe', 'rett1240', 'hupaŋ'), ('to_breathe', 'kaer1234', "su'paŋ"), ('to_breathe', 'blag1240-bama', 'sopaŋ'), ('to_breathe', 'blag1240-nule', 'hopaŋ'), ('to_breathe', 'blag1240-kulij', 'hopaŋ'), ('to_breathe', 'dein1238', 'opaŋ'), ('to_breathe', 'rett1240', 'ho:paŋ'), ('to_breathe', 'pura1258', 'hopaŋ'), ('to_breathe', 'adan1251-lawah', 'haʔan')}
 One or more Lamaholot dialects innovated forms not found in Alorese:
 {('to_breathe', 'lama1277-lewoe', 'hədu')}
 {('to_breathe', 'lama1277-kalik', 'nai'), ('to_breathe', 'lama1277-imulo', 'ər naisa'), ('to_breathe', 'lama1277-lewot', 'ər naiha'), ('to_breathe', 'lama1277-minga', 'ərə nai'), ('to_breathe', 'lama1277-lewop', 'ernaj'), ('to_breathe', 'lama1277-ritae', 'ər nãi')}
 {('to_breathe', 'lama1277-lamah', 'eha naikə'), ('to_breathe', 'lama1277-paina', 'ehe naig'), ('to_breathe', 'maka1316', "hau'neɁe"), ('to_breathe', 'lama1277-lerek', 'ˈehe ˈnai'), ('to_breathe', 'lama1277-lewok', 'sənaiga'), ('to_breathe', 'lama1277-belan', 'hea naik'), ('to_breathe', 'lama1277-merde', 'eha naikə'), ('to_breathe', 'lama1277-ileap', 'həŋə naikə'), ('to_breathe', 'lama1277-lamat', 'ha nei'), ('to_breathe', 'lama1277-kalik', 'esa nain'), ('to_breathe', 'lama1277-mulan', 'eha nai'), ('to_breathe', 'lama1277-lewuk', 'esa nai')}
 {('to_breathe', 'lama1277-pukau', 'boʔ'), ('to_breathe', 'sika1262-hewa', 'boʔ')}

  Just for clarity:
  There are also the following similarity classes shared between Alorese and LH.
  {('to_breathe', 'lama1277-botun', 'nəri nahiŋ'), ('to_breathe', 'lama1277-tanju', 'hari nãi'), ('to_breathe', 'alor1247-besar', "nami 'nihiŋ "), ('to_breathe', 'lama1277-waiba', 'harinãi'), ('to_breathe', 'lama1277-lewom', 'harinãi'), ('to_breathe', 'lama1277-baipi', 'harinaĩ'), ('to_breathe', 'lama1277-bama', 'harĩ'), ('to_breathe', 'lama1277-lewoi', 'ˈhariŋ'), ('to_breathe', 'lama1277-adona', 'ˈnɜriˈnɑĩ'), ('to_breathe', 'lama1277-waiwa', 'nərinãi'), ('to_breathe', 'lama1277-lewog', 'hari nait'), ('to_breathe', 'lama1277-horow', 'nəri nahiŋ'), ('to_breathe', 'alor1247-pandai', "naŋ 'nahiŋ"), ('to_breathe', 'alor1247-baran', "tar:ɛ 'nah:ĩ:"), ('to_breathe', 'lama1277-wuake', 'gidã nãi'), ('to_breathe', 'lama1277-watan', 'nəri nahiŋ'), ('to_breathe', 'lama1277-lamal', 'ərã nãi'), ('to_breathe', 'lama1277-lewob', 'hari nãi'), ('to_breathe', 'lama1277-adona', 'ˈnɜriˈnɑĩk'), ('to_breathe', 'lama1277-kiwan', 'nəri naik'), ('to_breathe', 'lama1277-dulhi', 'nəri nahiŋ'), ('to_breathe', 'lama1277-lamak', 'nərinãi')}


Concept ‘to_speak’ is different between Alorese and Lamaholot.

 One or more Lamaholot dialects innovated forms not found in Alorese:
 {('to_speak', 'hama1240', 'luruŋ'), ('to_speak', 'lama1277-adona', 'pɜˈʔulũ'), ('to_speak', 'sika1262-hewa', 'ulun')}
 {('to_speak', 'lama1277-lerek', 'pəˈnua')}
 {('to_speak', 'lama1277-kalik', 'pətuˈtosa')}
 {('to_speak', 'lama1277-lerek', 'ˈkoiŋ')}
 {('to_speak', 'lama1277-lewoi', 'ˈkɔda')}

  Just for clarity:
  There are also the following similarity classes shared between Alorese and LH.
  {('to_speak', 'alor1247-besar', 'tutu'), ('to_speak', 'lama1277-lewoi', 'ˈtutuʔ'), ('to_speak', 'alor1247-baran', 'tut:u'), ('to_speak', 'blag1240-bama', 'tutuk'), ('to_speak', 'alor1247-munas', "tutu 'apa"), ('to_speak', 'rett1240', 'tutuk'), ('to_speak', 'blag1240-kulij', 'tutuk'), ('to_speak', 'blag1240-nule', 'tutuk'), ('to_speak', 'keda1252-leuba', "tutuʔ 'nanaŋ"), ('to_speak', 'blag1240-warsa', 'tutuk'), ('to_speak', 'keda1252-leuba', 'tutuʔ'), ('to_speak', 'kaer1234', "tu'tuk"), ('to_speak', 'blag1240-tuntu', 'tutuk'), ('to_speak', 'buna1278-malia', 'thɔɛk'), ('to_speak', 'baka1276', 'tutuk'), ('to_speak', 'alor1247-pandai', 'tutu'), ('to_speak', 'pura1258', "vɛŋ 'tutuk"), ('to_speak', 'rett1240', 'ututuk'), ('to_speak', 'alor1247-munas', 'tutu')}


Concept ‘thatched_roof’ is different between Alorese and Lamaholot.

 One or more Lamaholot dialects innovated forms not found in Alorese:
 {('thatched_roof', 'lama1277-lewoi', 'luʔɔ')}
 {('thatched_roof', 'lama1277-kalik', 'uˈvu'), ('thatched_roof', 'lama1277-kalik', 'uˈvuŋ')}

  Just for clarity:
  There are also the following similarity classes shared between Alorese and LH.
  {('thatched_roof', 'lama1277-adona', 'ˈnakɜ'), ('thatched_roof', 'alor1247-munas', 'takar'), ('thatched_roof', 'lama1277-kalik', 'naˈkər'), ('thatched_roof', 'lama1277-lewoi', 'nuki'), ('thatched_roof', 'alor1247-pandai', "tə'nakar"), ('thatched_roof', 'alor1247-pandai', 'takar'), ('thatched_roof', 'alor1247-besar', "luo 'taka"), ('thatched_roof', 'lama1277-lerek', 'ˈnakar')}


Concept ‘morning’ is different between Alorese and Lamaholot.

 Alorese innovated the following forms:
 {('morning', 'alor1247-pandai', "kə'ramaŋ")}
 {('morning', 'alor1247-munas', 'wuraʔ'), ('morning', 'alor1247-pandai', 'wura'), ('morning', 'alor1247-besar', "fu'ra-fu'ra")}
 One or more Lamaholot dialects innovated forms not found in Alorese:
 {('morning', 'lama1277-lerek', 'baeˈnan'), ('morning', 'lama1277-kalik', 'baˈɛn')}
 {('morning', 'lama1277-adona', "ɡ(ə)'hula"), ('morning', 'lama1277-lewoi', 'ˈɡuləŋ'), ('morning', 'lama1277-adona', "hə'ɡula")}


Concept ‘to_smell’ is different between Alorese and Lamaholot.

 Alorese innovated the following forms:
 {('to_smell', 'alor1247-munas', 'hugoʔ')}
 {('to_smell', 'alor1247-pandai', "wun'gɔ")}
 One or more Lamaholot dialects innovated forms not found in Alorese:
 {('to_smell', 'lama1277-paina', 'hodam'), ('to_smell', 'lama1277-lewuk', 'sodam'), ('to_smell', 'lama1277-belan', 'hodam'), ('to_smell', 'lama1277-lewop', 'hodam'), ('to_smell', 'lama1277-lewok', 'sodam'), ('to_smell', 'lama1277-mulan', 'hodɤ'), ('to_smell', 'lama1277-minga', 'sodam'), ('to_smell', 'lama1277-merde', 'hodã'), ('to_smell', 'lama1277-lewot', 'hodam'), ('to_smell', 'lama1277-wuake', 'hodã'), ('to_smell', 'lama1277-kalik', 'sɔˈdam'), ('to_smell', 'lama1277-lewoe', 'hodã'), ('to_smell', 'lama1277-kalik', 'sodam'), ('to_smell', 'lama1277-lamah', 'hodã'), ('to_smell', 'lama1277-imulo', 'sodam'), ('to_smell', 'lama1277-lerek', 'hoˈdam (vaˈuk)')}
 {('to_smell', 'lama1277-botun', 'pənuk'), ('to_smell', 'lama1277-lamal', 'penukã'), ('to_smell', 'lama1277-adona', 'pɜnuˈhuk'), ('to_smell', 'lama1277-kiwan', 'penuhuk'), ('to_smell', 'lama1277-ileap', 'nuhuk'), ('to_smell', 'mamb1306', 'penel'), ('to_smell', 'lama1277-horow', 'pənuhuk'), ('to_smell', 'lama1277-dulhi', 'pənuhuk')}

  Just for clarity:
  There are also the following similarity classes shared between Alorese and LH.
  {('to_smell', 'lama1277-watan', 'sioro'), ('to_smell', 'sika1262-tanai', 'sino-ŋ'), ('to_smell', 'lama1277-bama', 'siõ'), ('to_smell', 'lama1277-lamak', 'sio'), ('to_smell', 'lama1277-tanju', 'soba'), ('to_smell', 'lama1277-lewog', 'siõ'), ('to_smell', 'lama1277-baipi', 'siõ'), ('to_smell', 'lama1277-waiwa', 'sio'), ('to_smell', 'lama1277-pukau', 'siõ'), ('to_smell', 'alor1247-baran', 'siɔ̃:'), ('to_smell', 'lama1277-lewom', 'siõ'), ('to_smell', 'lama1277-ritae', 'siõ'), ('to_smell', 'lama1277-lewoi', 'ˈsiɔŋ'), ('to_smell', 'lama1277-waiba', 'siõ'), ('to_smell', 'lama1277-lewob', 'siõ')}
  {('to_smell', 'lama1277-lamat', 'are'), ('to_smell', 'alor1247-besar', 'mare'), ('to_smell', 'alor1247-pandai', 'marɛ')}


Concept ‘clothing’ is different between Alorese and Lamaholot.

 Alorese shares the following forms with TAP languages:
 {('clothing', 'abui1241-ulaga', 'konde'), ('clothing', 'alor1247-pandai', 'kɔndɔ'), ('clothing', 'pura1258', 'kondo'), ('clothing', 'blag1240-kulij', 'kondo'), ('clothing', 'alor1247-baran', 'konʤo'), ('clothing', 'blag1240-warsa', 'kondo')}
 One or more Lamaholot dialects innovated forms not found in Alorese:
 {('clothing', 'lama1277-lewok', 'anampunak')}
 {('clothing', 'lama1277-adona', 'aleˈlolo'), ('clothing', 'lama1277-kalik', 'ˈale ˈlɔlɔ'), ('clothing', 'lama1277-imulo', 'alolo'), ('clothing', 'lama1277-horow', 'alelolo'), ('clothing', 'lama1277-waiwa', 'alelolo'), ('clothing', 'lama1277-ileap', 'alelolo'), ('clothing', 'lama1277-lerek', 'ˈale ˈlolo'), ('clothing', 'lama1277-ritae', 'alelolõ'), ('clothing', 'lama1277-lewuk', 'alelolo'), ('clothing', 'lama1277-watan', 'alelolo'), ('clothing', 'lama1277-tanju', 'alelolo'), ('clothing', 'lama1277-botun', 'alelolo'), ('clothing', 'lama1277-paina', 'alelolo'), ('clothing', 'lama1277-minga', 'alelolo'), ('clothing', 'lama1277-kalik', 'ˈale ˈlolo'), ('clothing', 'lama1277-lamah', 'alelolo'), ('clothing', 'lama1277-wuake', 'alelolo'), ('clothing', 'lama1277-lamak', 'alelolo'), ('clothing', 'lama1277-mulan', 'alelolo'), ('clothing', 'lama1277-lamal', 'alelolo'), ('clothing', 'lama1277-dulhi', 'alelolo'), ('clothing', 'lama1277-kalik', 'alelolo'), ('clothing', 'lama1277-lewot', 'aleloloja'), ('clothing', 'lama1277-kiwan', 'alelolo'), ('clothing', 'lama1277-lewop', 'alelol'), ('clothing', 'lama1277-lamat', 'alalolo'), ('clothing', 'lama1277-merde', 'alelolo'), ('clothing', 'lama1277-belan', 'alelolo')}
 {('clothing', 'keda1252', 'ulialen'), ('clothing', 'lama1277-lewoe', 'uluale')}
 {('clothing', 'lama1277-baipi', 'agopake'), ('clothing', 'lama1277-bama', 'agopake'), ('clothing', 'lama1277-pukau', 'akopake'), ('clothing', 'lama1277-lewom', 'agopake'), ('clothing', 'lama1277-lewog', 'akəpake'), ('clothing', 'lama1277-waiba', 'agopake')}

  Just for clarity:
  There are also the following similarity classes shared between Alorese and LH.
  {('clothing', 'keda1252-leuba', "pa'kejan"), ('clothing', 'kelo1247-hopte', "pə'kar"), ('clothing', 'lama1277-lewoi', 'əˈkaŋ'), ('clothing', 'sika1262-hewa', 'pakian'), ('clothing', 'lama1277-kalik', 'məˈkaŋ-a'), ('clothing', 'alor1247-munas', "pə'kiaŋ"), ('clothing', 'sika1262-hewa', 'pakaian'), ('clothing', 'alor1247-besar', "pa'kiãŋ"), ('clothing', 'koto1251', 'pake'), ('clothing', 'kelo1247-bring', 'pəkɑr'), ('clothing', 'lama1277-lewob', 'pakaian'), ('clothing', 'blag1240-warsa', 'ke:ŋ')}


Concept ‘to_fall_from_above’ is different between Alorese and Lamaholot.

 One or more Lamaholot dialects innovated forms not found in Alorese:
 {('to_fall_from_above', 'lama1277-lewom', 'lubak')}
 {('to_fall_from_above', 'lama1277-kalik', 'ləˈŋat'), ('to_fall_from_above', 'lama1277-merde', 'ləŋat'), ('to_fall_from_above', 'lama1277-lamah', 'ləŋa'), ('to_fall_from_above', 'lama1277-lewuk', 'ləŋat'), ('to_fall_from_above', 'lama1277-belan', 'ləŋat'), ('to_fall_from_above', 'lama1277-mulan', 'ləŋa'), ('to_fall_from_above', 'lama1277-lewok', 'ləŋat'), ('to_fall_from_above', 'lama1277-imulo', 'ləŋata'), ('to_fall_from_above', 'lama1277-lamak', 'ləŋa')}
 {('to_fall_from_above', 'lama1277-tanju', 'dəkak'), ('to_fall_from_above', 'lama1277-pukau', 'dəkak'), ('to_fall_from_above', 'lama1277-lewog', 'dəka'), ('to_fall_from_above', 'abui1241-fuime', 'takuwila'), ('to_fall_from_above', 'lama1277-bama', 'dəkak'), ('to_fall_from_above', 'lama1277-ileap', 'dəka'), ('to_fall_from_above', 'lama1277-waiba', 'dəkak'), ('to_fall_from_above', 'lama1277-lewoi', 'ˈdəka'), ('to_fall_from_above', 'lama1277-ritae', 'dəka'), ('to_fall_from_above', 'lama1277-wuake', 'gətak'), ('to_fall_from_above', 'lama1277-minga', 'gəta'), ('to_fall_from_above', 'lama1277-botun', 'dəkak'), ('to_fall_from_above', 'atim1239', 'da-kuila')}
 {('to_fall_from_above', 'lama1277-baipi', 'loʔu')}
 {('to_fall_from_above', 'sarr1247-adiab', 'baʔ'), ('to_fall_from_above', 'lama1277-kalik', 'peˈal'), ('to_fall_from_above', 'nede1245', 'baʔa'), ('to_fall_from_above', 'lama1277-lerek', 'pəˈhol dʒe ˈlodo'), ('to_fall_from_above', 'teiw1235', 'baɁ'), ('to_fall_from_above', 'lama1277-paina', 'pahol'), ('to_fall_from_above', 'sarr1247-nule', 'baʔ')}
 {('to_fall_from_above', 'tetu1246', 'monu'), ('to_fall_from_above', 'p-cent2245-abvd', '*ma-nabuq'), ('to_fall_from_above', 'p-cent2245-abvd', '*ka-nabuq'), ('to_fall_from_above', 'p-mala1545-acd', '*nabuq'), ('to_fall_from_above', 'p-mala1545-abvd', '*nabuq'), ('to_fall_from_above', 'lama1277-lewop', 'gəpu'), ('to_fall_from_above', 'kema1243', "ko'moku"), ('to_fall_from_above', 'tetu1245-vique', 'monu'), ('to_fall_from_above', 'idat1237', "mɑ'nahu"), ('to_fall_from_above', 'kema1243', "ma'nahu"), ('to_fall_from_above', 'tetu1245-suai', 'monu')}
 {('to_fall_from_above', 'keda1252-leuba', "mo'ruʔ"), ('to_fall_from_above', 'keda1252-leuwa', 'hubaʔ hɔkɔ'), ('to_fall_from_above', 'keda1252-leuwa', 'hubaʔ kɔliʔ'), ('to_fall_from_above', 'koto1251', 'n-mofu'), ('to_fall_from_above', 'sika1262-tanai', 'ɡoru'), ('to_fall_from_above', 'keda1252-leuwa', 'mɔruʔ hɔkɔ'), ('to_fall_from_above', 'lama1277-lewoe', 'moru'), ('to_fall_from_above', 'keda1252', 'moruʔ'), ('to_fall_from_above', 'sika1262-hewa', 'goru')}

  Just for clarity:
  There are also the following similarity classes shared between Alorese and LH.
  {('to_fall_from_above', 'alor1247-pandai', "gɔkal 'lɔdɔŋ"), ('to_fall_from_above', 'alor1247-pandai', 'gɔkal'), ('to_fall_from_above', 'lama1277-lamat', 'goli'), ('to_fall_from_above', 'alor1247-munas', "gɔkal 'lɔdɔŋ"), ('to_fall_from_above', 'lama1277-lewob', 'goka'), ('to_fall_from_above', 'keda1252-leuwa', 'kɔʔal'), ('to_fall_from_above', 'lama1277-waiwa', 'goka'), ('to_fall_from_above', 'lama1277-watan', 'goka'), ('to_fall_from_above', 'lama1277-kiwan', 'goka'), ('to_fall_from_above', 'lama1277-lewot', 'gokala'), ('to_fall_from_above', 'alor1247-baran', 'gɔka'), ('to_fall_from_above', 'alor1247-besar', "gɔka 'iti 'loloŋ"), ('to_fall_from_above', 'lama1277-kalik', 'gokal'), ('to_fall_from_above', 'lama1277-horow', 'goka'), ('to_fall_from_above', 'lama1277-dulhi', 'goka'), ('to_fall_from_above', 'alor1247-besar', 'gɔka'), ('to_fall_from_above', 'lama1277-lamal', 'goka'), ('to_fall_from_above', 'kama1365', 'kok'), ('to_fall_from_above', 'lama1277-adona', 'ˈɡɔka')}


Concept ‘there’ is different between Alorese and Lamaholot.

 Alorese shares the following forms with other AN languanges, but not Lamaholot:
 {('there', 'alor1247-munas', "kə'tːi"), ('there', 'alor1247-pandai', "ɔrkə'tːe"), ('there', 'alor1247-munas', "kə'tːi  'lɔlɔŋ"), ('there', 'p-mala1545-acd', '*i-di'), ('there', 'alor1247-pandai', "ɔrka'tːi")}
 One or more Lamaholot dialects innovated forms not found in Alorese:
 {('there', 'lama1277-kalik', 'ˈlau')}
 {('there', 'kema1243', 'dia nua'), ('there', 'kema1243', ",dia lɑ'nua"), ('there', 'keda1252-leuwa', 'nɔbε'), ('there', 'keda1252', 'dinobeʔ'), ('there', 'lama1277-paina', 'nəbo'), ('there', 'keda1252-leuwa', 'di nɔbε'), ('there', 'lama1277-dulhi', 'sene')}
 {('there', 'lama1277-lamal', 'depe'), ('there', 'lama1277-lewob', 'pehe'), ('there', 'lama1277-kiwan', 'dəpe'), ('there', 'lama1277-lewuk', 'bete'), ('there', 'lama1277-imulo', 'kətəre'), ('there', 'lama1277-lewom', 'pe'), ('there', 'lama1277-kalik', 'bete'), ('there', 'lama1277-lewoi', 'ˈpeʔɛŋ'), ('there', 'lama1277-ritae', 'dipe'), ('there', 'lama1277-lewot', 'ketere'), ('there', 'lama1277-pukau', 'tete'), ('there', 'lama1277-ileap', 'təpe'), ('there', 'lama1277-botun', 'dəpe'), ('there', 'lama1277-lewop', 'kətəre'), ('there', 'lama1277-baipi', 'tewe'), ('there', 'lama1277-lewog', 'pe'), ('there', 'lama1277-adona', 'dɜˈhɛrɜ'), ('there', 'lama1277-lamat', 'dabe'), ('there', 'lama1277-lamak', 'dəwe'), ('there', 'lama1277-kalik', 'ˈʤae'), ('there', 'lama1277-mulan', 'depe'), ('there', 'lama1277-waiwa', 'təpi'), ('there', 'lama1277-belan', 'nəpe'), ('there', 'lama1277-wuake', 'dəpe'), ('there', 'lama1277-horow', 'təpe')}
 {('there', 'lama1277-minga', 'nakre')}

  Just for clarity:
  There are also the following similarity classes shared between Alorese and LH.
  {('there', 'lama1277-lewoe', 'weli'), ('there', 'alor1247-munas', "kə'lːi"), ('there', 'alor1247-besar', "fali 'kali"), ('there', 'alor1247-pandai', "ɔrka'li"), ('there', 'lama1277-waiba', 'pewəli'), ('there', 'alor1247-munas', "rkwə'lːi"), ('there', 'alor1247-munas', "kə'lːi 'lauŋ"), ('there', 'alor1247-baran', "alika'le:"), ('there', 'lama1277-lamah', 'na wəli'), ('there', 'lama1277-lewok', 'wəli'), ('there', 'lama1277-tanju', 'pəwəli'), ('there', 'alor1247-munas', "rvə'lːi"), ('there', 'lama1277-bama', 'pewəli'), ('there', 'lama1277-lerek', 'rəˈvəl'), ('there', 'abui1241-petle', 'ʋe'), ('there', 'lama1277-merde', 'na wəli'), ('there', 'abui1241-petle', 'we'), ('there', 'lama1277-kalik', 'vəˈli'), ('there', 'lama1277-watan', 'pəwəli')}


Concept ‘above’ is different between Alorese and Lamaholot.

 One or more Lamaholot dialects innovated forms not found in Alorese:
 {('above', 'koto1251', 'ata'), ('above', 'lama1277-lerek', 'rəˈdʒ͡e'), ('above', 'lama1277-kalik', 'ʤene'), ('above', 'lama1277-kalik', 'ʤe'), ('above', 'kama1365', 'taː')}

  Just for clarity:
  There are also the following similarity classes shared between Alorese and LH.
  {('above', 'alor1247-munas', 'lɔlɔŋ'), ('above', 'alor1247-besar', "iti 'lɔlɔŋ"), ('above', 'lama1277-kalik', 'ˈɡolo'), ('above', 'alor1247-baran', 'lɔlɔŋ'), ('above', 'alor1247-besar', "ɔrɔ 'lɔlɔŋ"), ('above', 'lama1277-adona', 'lolõ'), ('above', 'alor1247-pandai', 'lɔlɔŋ'), ('above', 'lama1277-kalik', 'ˈlolo'), ('above', 'lama1277-adona', 'ˈbloʔlõ'), ('above', 'lama1277-lewoi', 'tɛti ˈlɔlɔŋ'), ('above', 'sika1262-hewa', 'lala nan')}


Concept ‘wet’ is different between Alorese and Lamaholot.

 Alorese innovated the following forms:
 {('wet', 'alor1247-besar', 'ɛlɛ'), ('wet', 'alor1247-munas', 'ɛlɛk'), ('wet', 'alor1247-baran', 'ɛlɛ'), ('wet', 'alor1247-pandai', 'ɛlek')}
 One or more Lamaholot dialects innovated forms not found in Alorese:
 {('wet', 'lama1277-kiwan', 'nəm’e'), ('wet', 'lama1277-lewoi', 'dəˈmɛŋ'), ('wet', 'lama1277-merde', 'nəmʔe'), ('wet', 'lama1277-tanju', 'dəma'), ('wet', 'tuku1254', 'tita'), ('wet', 'lama1277-lewog', 'dəmã'), ('wet', 'lama1277-pukau', 'deman'), ('wet', 'lama1277-ritae', 'dəme'), ('wet', 'lama1277-baipi', 'kədəmu'), ('wet', 'lama1277-waiba', 'kədəmuk'), ('wet', 'lama1277-horow', 'nəme'), ('wet', 'lama1277-ileap', 'nəmaŋ'), ('wet', 'lama1277-bama', 'dəme'), ('wet', 'lama1277-lamak', 'nəməŋ'), ('wet', 'lama1277-lewom', 'kədəmu'), ('wet', 'lama1277-watan', 'nəmʔe'), ('wet', 'lama1277-lamah', 'nəme'), ('wet', 'lama1277-waiwa', 'nəme'), ('wet', 'lama1277-botun', 'nəme'), ('wet', 'lama1277-dulhi', 'nəme')}
 {('wet', 'blag1240-bama', 'kolo'), ('wet', 'blag1240-warsa', 'xolo'), ('wet', 'sarr1247-nule', "ka'loʔ"), ('wet', 'lama1277-lewoe', 'ilo'), ('wet', 'fata1247', 'culu'), ('wet', 'blag1240-tuntu', 'qolo'), ('wet', 'lama1277-lewob', 'kəwaʔi'), ('wet', 'sarr1247-adiab', "ka'loʔ"), ('wet', 'kaer1234', "qo'lo"), ('wet', 'teiw1235', "qa'loɁ")}
 {('wet', 'lama1277-adona', 'ˈnamʔɛ̃')}
 {('wet', 'tetu1245-vique', 'wek'), ('wet', 'tetu1245-suai', 'werɔk'), ('wet', 'lama1277-lamat', 'weĩ'), ('wet', 'kuii1253', 'rik')}
 {('wet', 'lama1277-lerek', 'hənəbaˈrən'), ('wet', 'lama1277-lamal', 'sənəbe'), ('wet', 'lama1277-mulan', 'sənəbe'), ('wet', 'lama1277-lewuk', 'səbar'), ('wet', 'lamm1241-westp', 'hub:a'), ('wet', 'lama1277-lewok', 'sənəbar'), ('wet', 'lama1277-kalik', 'sənəˈbar'), ('wet', 'lama1277-minga', 'sənəbar'), ('wet', 'lama1277-kalik', 'sənəbar'), ('wet', 'lama1277-lewot', 'həba'), ('wet', 'lama1277-paina', 'hənəbarən'), ('wet', 'lama1277-belan', 'səba'), ('wet', 'mamb1306', 'ʔɛra'), ('wet', 'lama1277-wuake', 'səba'), ('wet', 'lama1277-lewop', 'həba'), ('wet', 'lama1277-imulo', 'sənəbar')}


Concept ‘claw’ is different between Alorese and Lamaholot.

 Alorese innovated the following forms:
 {('claw', 'alor1247-baran', "limɑŋ ta'nuŋgul")}
 {('claw', 'alor1247-munas', "kɛla 'leiŋ")}
 {('claw', 'alor1247-besar', "ga'raku"), ('claw', 'buna1278-bobon', 'galakha'), ('claw', 'alor1247-pandai', "k'rako"), ('claw', 'sawi1256', "kə'riki")}
 Alorese shares the following forms with TAP languages:
 {('claw', 'alor1247-baran', "limɑŋ ta'nuŋgul")}
 {('claw', 'alor1247-munas', "kɛla 'leiŋ")}
 {('claw', 'alor1247-besar', "ga'raku"), ('claw', 'buna1278-bobon', 'galakha'), ('claw', 'alor1247-pandai', "k'rako"), ('claw', 'sawi1256', "kə'riki")}
 One or more Lamaholot dialects innovated forms not found in Alorese:
 {('claw', 'lama1277-adona', 'kɜˈmukure'), ('claw', 'kelo1247-bring', 'gɛkuh'), ('claw', 'p-mala1545-acd', '*kuhkuh')}
 {('claw', 'lama1277-lewoi', 'təˈnuʔi')}
 {('claw', 'lama1277-lerek', 'kabəˈlən')}


Concept ‘woman’ is different between Alorese and Lamaholot.

 One or more Lamaholot dialects innovated forms not found in Alorese:
 {('woman', 'lama1277-paina', 'ronen'), ('woman', 'lama1277-lerek', 'ronen')}
 {('woman', 'lama1277-lewop', 'gərapoja'), ('woman', 'lama1277-minga', 'gəraporu'), ('woman', 'lama1277-imulo', 'gərapor'), ('woman', 'lama1277-lewot', 'gərapoja')}
 {('woman', 'p-cent2245-abvd', '*b-in-ay'), ('woman', 'keda1252-leuwa', 'inaʔ'), ('woman', 'buna1278-bobon', 'phana'), ('woman', 'keda1252-leuwa', 'inε'), ('woman', 'laka1255', 'hina'), ('woman', 'buna1278-suai', 'phana'), ('woman', 'idat1237', "mahi'nak"), ('woman', 'lama1277-lewuk', 'bineja'), ('woman', 'lama1277-kalik', 'binen'), ('woman', 'lama1277-lewok', 'bine'), ('woman', 'buna1278-malia', "ʔɛn 'phana"), ('woman', 'keda1252-leuwa', 'i̘nɔ'), ('woman', 'lama1277-kalik', 'biˈnɛn')}
 {('woman', 'lama1277-bama', 'inawae'), ('woman', 'lama1277-lewom', 'inawae'), ('woman', 'lama1277-dulhi', 'inawae'), ('woman', 'lama1277-horow', 'inawae'), ('woman', 'lama1277-adona', 'ˈʔina wae'), ('woman', 'lama1277-kiwan', 'inawae'), ('woman', 'lama1277-lewog', 'inawae'), ('woman', 'lama1277-tanju', 'inawae'), ('woman', 'lama1277-ileap', 'inawae'), ('woman', 'lama1277-lewoi', 'ina ˈwai')}

  Just for clarity:
  There are also the following similarity classes shared between Alorese and LH.
  {('woman', 'alor1247-baran', "ka'fae"), ('woman', 'lama1277-waiwa', 'kawae'), ('woman', 'lama1277-belan', 'kəbarawae'), ('woman', 'alor1247-pandai', "k'wae"), ('woman', 'alor1247-besar', "ka'fae"), ('woman', 'lama1277-ritae', 'bərkəwãe'), ('woman', 'lama1277-lamal', 'kəbarafae'), ('woman', 'lama1277-wuake', 'kəwae'), ('woman', 'lama1277-watan', 'bərkəwae'), ('woman', 'alor1247-munas', "k'wae"), ('woman', 'lama1277-lamak', 'bərkəwae'), ('woman', 'lama1277-merde', 'bərwae'), ('woman', 'lama1277-botun', 'bərwae'), ('woman', 'lama1277-mulan', 'kəbarawae'), ('woman', 'lama1277-waiba', 'bərkawãe'), ('woman', 'kafo1240', 'kofɑ'), ('woman', 'lama1277-pukau', 'vae'), ('woman', 'lama1277-lewob', 'kəbarawãi'), ('woman', 'lama1277-lamah', 'bərwae'), ('woman', 'lama1277-lamat', 'bare'), ('woman', 'lama1277-lewoe', 'bare'), ('woman', 'lama1277-baipi', 'bərwãi')}


Concept ‘coral_reef’ is different between Alorese and Lamaholot.

 Alorese innovated the following forms:
 {('coral_reef', 'alor1247-munas', 'aŋgu')}
 {('coral_reef', 'alor1247-besar', "ta'ga")}
 One or more Lamaholot dialects innovated forms not found in Alorese:
 {('coral_reef', 'lama1277-lewoi', 'aha')}
 {('coral_reef', 'lama1277-adona', 'rubu')}

  Just for clarity:
  There are also the following similarity classes shared between Alorese and LH.
  {('coral_reef', 'lama1277-lerek', 'ˈuhor'), ('coral_reef', 'alor1247-munas', 'uhuŋ'), ('coral_reef', 'alor1247-munas', 'tuhu'), ('coral_reef', 'alor1247-besar', 'umuŋ'), ('coral_reef', 'alor1247-besar', 'uho'), ('coral_reef', 'alor1247-munas', 'huŋ'), ('coral_reef', 'alor1247-pandai', 'uhuŋ'), ('coral_reef', 'sika1262-hewa', 'ruhɔ'), ('coral_reef', 'keda1252-leuba', 'ruhu')}


Concept ‘rib’ is different between Alorese and Lamaholot.

 Alorese innovated the following forms:
 {('rib', 'alor1247-besar', "punuŋ 'kaʤɔŋ")}
 {('rib', 'alor1247-pandai', 'hakaŋ'), ('rib', 'alor1247-munas', 'hakaŋ'), ('rib', 'alor1247-besar', 'hakaŋ')}
 One or more Lamaholot dialects innovated forms not found in Alorese:
 {('rib', 'lama1277-lerek', 'hake riuk')}
 {('rib', 'lama1277-adona', 'kɜˈnawaˈne'), ('rib', 'lama1277-kalik', 'kənaˈvan'), ('rib', 'lama1277-lewoi', 'ˈriʔu kəˈnawaŋ')}


Concept ‘clay’ is different between Alorese and Lamaholot.

 Alorese innovated the following forms:
 {('clay', 'alor1247-besar', "fa'raha 'mɛaŋ")}
 One or more Lamaholot dialects innovated forms not found in Alorese:
 {('clay', 'lama1277-lerek', 'ˈanei kəˈlubaŋ')}
 {('clay', 'lama1277-kalik', 'əˈna vaˈkɔt')}

  Just for clarity:
  There are also the following similarity classes shared between Alorese and LH.
  {('clay', 'alor1247-pandai', "tana 'meaŋ"), ('clay', 'alor1247-munas', "tana 'meaŋ"), ('clay', 'lama1277-lewoi', 'tana ˈmɛʔɑŋ')}


Concept ‘grass’ is different between Alorese and Lamaholot.

 Alorese innovated the following forms:
 {('grass', 'alor1247-pandai', 'laduŋ'), ('grass', 'alor1247-besar', "la'duŋ"), ('grass', 'alor1247-munas', "lə'duŋ"), ('grass', 'alor1247-baran', "la'duŋ")}
 One or more Lamaholot dialects innovated forms not found in Alorese:
 {('grass', 'lama1277-belan', 'kərəkak'), ('grass', 'lama1277-kalik', 'kərəkak'), ('grass', 'lama1277-lewop', 'kərəka'), ('grass', 'lama1277-lewuk', 'kərəkak'), ('grass', 'lama1277-imulo', 'kərəkasa'), ('grass', 'lama1277-kalik', 'krəˈkak'), ('grass', 'lama1277-lewot', 'kərəka'), ('grass', 'lama1277-watan', 'kədəku'), ('grass', 'lama1277-kiwan', 'kəduku'), ('grass', 'lama1277-adona', 'kəˈdəku')}
 {('grass', 'lama1277-merde', 'kərəmət'), ('grass', 'lama1277-horow', 'kərəmət'), ('grass', 'lama1277-lamal', 'kərəmet'), ('grass', 'lama1277-lewoi', 'kərəmə'), ('grass', 'lama1277-baipi', 'kərəmət'), ('grass', 'lama1277-botun', 'kərəmət'), ('grass', 'lama1277-lewog', 'kərəmə'), ('grass', 'lama1277-ileap', 'kərəmət'), ('grass', 'lama1277-minga', 'rearu'), ('grass', 'lama1277-lamah', 'kərəmət'), ('grass', 'lama1277-wuake', 'kəruɁu'), ('grass', 'lama1277-lewom', 'kərəmət'), ('grass', 'lama1277-waiwa', 'kərəmət'), ('grass', 'lama1277-mulan', 'kərəmə'), ('grass', 'lama1277-ritae', 'kərəmə')}
 {('grass', 'atim1239', 'kupe'), ('grass', 'abui1241-fuime', 'kupei'), ('grass', 'lama1277-lamat', 'gəpe'), ('grass', 'lama1277-lewoe', 'gəpe')}
 {('grass', 'lama1277-lewok', 'mewak'), ('grass', 'lama1277-lerek', 'ˈmevau'), ('grass', 'lama1277-paina', 'mewaw')}
 {('grass', 'kabo1247', 'luju'), ('grass', 'lama1277-bama', 'luʔo'), ('grass', 'lama1277-pukau', 'luʔo'), ('grass', 'lama1277-dulhi', 'luʔo'), ('grass', 'lama1277-lamak', 'luʔo'), ('grass', 'lama1277-lewob', 'luʔo'), ('grass', 'lama1277-waiba', 'luʔo')}
 {('grass', 'tetu1245-suai', 'duɁut'), ('grass', 'lama1277-tanju', 'hokə̃'), ('grass', 'p-mala1545-acd', '*zukut'), ('grass', 'p-mala1545-acd', '*dukut'), ('grass', 'blag1240-bama', 'ut'), ('grass', 'buna1278-malia', 'hu:t'), ('grass', 'blag1240-tuntu', 'u:t'), ('grass', 'tetu1245-vique', 'duɁut')}


Concept ‘to_lie_down’ is different between Alorese and Lamaholot.

 One or more Lamaholot dialects innovated forms not found in Alorese:
 {('to_lie_down', 'lama1277-waiwa', 'pədenək'), ('to_lie_down', 'lama1277-dulhi', 'pədenək')}
 {('to_lie_down', 'lama1277-lerek', 'ˈeva'), ('to_lie_down', 'lama1277-lamah', 'ewa'), ('to_lie_down', 'lama1277-lamal', 'pafã'), ('to_lie_down', 'lama1277-belan', 'pawaŋ'), ('to_lie_down', 'lama1277-mulan', 'pawaŋ'), ('to_lie_down', 'lama1277-kalik', 'paˈva'), ('to_lie_down', 'lama1277-lewuk', 'pawa'), ('to_lie_down', 'lama1277-kalik', 'pawan'), ('to_lie_down', 'lama1277-imulo', 'pafaŋa'), ('to_lie_down', 'lama1277-lewot', 'pafaŋa'), ('to_lie_down', 'lama1277-lewoe', 'ewa'), ('to_lie_down', 'lama1277-ileap', 'ewan'), ('to_lie_down', 'lama1277-lamat', 'ewã')}
 {('to_lie_down', 'lama1277-tanju', 'epa gola'), ('to_lie_down', 'lama1277-pukau', 'gokal'), ('to_lie_down', 'lama1277-ritae', 'gola'), ('to_lie_down', 'lama1277-paina', 'galek(a)'), ('to_lie_down', 'lama1277-lewog', 'polar'), ('to_lie_down', 'lama1277-bama', 'pola'), ('to_lie_down', 'lama1277-kiwan', 'pola'), ('to_lie_down', 'lama1277-lewok', 'gəle')}
 {('to_lie_down', 'koto1251', 'n-tupa'), ('to_lie_down', 'maka1316', "tefu'nei"), ('to_lie_down', 'lama1277-wuake', 'toba'), ('to_lie_down', 'lama1277-lewop', 'toba'), ('to_lie_down', 'lama1277-minga', 'tobana')}
 {('to_lie_down', 'lama1277-lewob', 'ledãnə'), ('to_lie_down', 'lama1277-lamak', 'ledã')}
 {('to_lie_down', 'p-cent2245-abvd', '*qinəp'), ('to_lie_down', 'p-cent2245-abvd', '*qenəp'), ('to_lie_down', 'lama1277-horow', 'linək'), ('to_lie_down', 'p-mala1545-abvd', '*qinep')}
 {('to_lie_down', 'lama1277-adona', 'ˈʔolɛʔ')}

  Just for clarity:
  There are also the following similarity classes shared between Alorese and LH.
  {('to_lie_down', 'lama1277-botun', 'pənaiŋ'), ('to_lie_down', 'blag1240-kulij', 'bena'), ('to_lie_down', 'alor1247-pandai', "pɔna 'alɛŋ"), ('to_lie_down', 'alor1247-besar', "pɔna-'pɔna"), ('to_lie_down', 'alor1247-munas', 'pɔna')}
  {('to_lie_down', 'lama1277-merde', 'turu'), ('to_lie_down', 'lama1277-waiba', 'tobãturu'), ('to_lie_down', 'alor1247-baran', 'tur:u'), ('to_lie_down', 'alor1247-pandai', 'turu'), ('to_lie_down', 'lama1277-lewoi', 'ˈturu')}


Concept ‘to_curse’ is different between Alorese and Lamaholot.

 Alorese innovated the following forms:
 {('to_curse', 'alor1247-pandai', "sa'lːɛ")}
 {('to_curse', 'alor1247-munas', "noe 'puwaŋ")}
 {('to_curse', 'alor1247-pandai', 'hɔi')}
 {('to_curse', 'alor1247-pandai', 'hina')}
 {('to_curse', 'alor1247-munas', "hoi 'hari 'biru")}
 {('to_curse', 'alor1247-besar', "noe 'gama"), ('to_curse', 'pura1258', "nehe'gamar")}
 Alorese shares the following forms with TAP languages:
 {('to_curse', 'alor1247-pandai', "sa'lːɛ")}
 {('to_curse', 'alor1247-munas', "noe 'puwaŋ")}
 {('to_curse', 'alor1247-pandai', 'hɔi')}
 {('to_curse', 'alor1247-pandai', 'hina')}
 {('to_curse', 'alor1247-munas', "hoi 'hari 'biru")}
 {('to_curse', 'alor1247-besar', "noe 'gama"), ('to_curse', 'pura1258', "nehe'gamar")}
 One or more Lamaholot dialects innovated forms not found in Alorese:
 {('to_curse', 'lama1277-kalik', 'ˈpsata')}
 {('to_curse', 'lama1277-lewoi', 'ˈtumaŋ'), ('to_curse', 'lama1277-adona', 'ˈtuma')}


Concept ‘grandchild’ is different between Alorese and Lamaholot.

 Alorese innovated the following forms:
 {('grandchild', 'alor1247-besar', 'bapaŋ'), ('grandchild', 'alor1247-munas', 'bapa'), ('grandchild', 'alor1247-pandai', "ana 'bapa")}
 One or more Lamaholot dialects innovated forms not found in Alorese:
 {('grandchild', 'maka1316', 'dada'), ('grandchild', 'sika1262-hewa', 'ʧuʧu'), ('grandchild', 'lama1277-adona', 'tʃutʃu'), ('grandchild', 'kelo1247-hopte', "dat'ja:"), ('grandchild', 'kelo1247-hopte', 'dat'), ('grandchild', 'kelo1247-bring', 'dɑt'), ('grandchild', 'blag1240-warsa', 'pidat')}


Concept ‘cross_cousin’ is different between Alorese and Lamaholot.

 Alorese innovated the following forms:
 {('cross_cousin', 'alor1247-besar', "ka'fae 'uma"), ('cross_cousin', 'alor1247-besar', "ka'lake 'uma")}
 {('cross_cousin', 'alor1247-pandai', "pukɔŋ 'anaŋ")}
 {('cross_cousin', 'alor1247-munas', "higu 'nuka")}
 {('cross_cousin', 'alor1247-munas', "wai kɔ'lːuʔ")}
 One or more Lamaholot dialects innovated forms not found in Alorese:
 {('cross_cousin', 'keda1252-leuba', 'ine ame'), ('cross_cousin', 'lama1277-lewoi', 'ine ame')}
 {('cross_cousin', 'lama1277-adona', 'breũ')}
 {('cross_cousin', 'lama1277-adona', 'opu')}
 {('cross_cousin', 'lama1277-lewoi', 'mɑhɑn'), ('cross_cousin', 'keda1252-leuba', 'mahan')}


Concept ‘to_tie’ is different between Alorese and Lamaholot.

 Alorese innovated the following forms:
 {('to_tie', 'alor1247-pandai', 'wela')}
 One or more Lamaholot dialects innovated forms not found in Alorese:
 {('to_tie', 'abui1241-ulaga', 'kor'), ('to_tie', 'lama1277-lewoe', 'gui'), ('to_tie', 'abui1241-takal', "hä.'kɔl"), ('to_tie', 'abui1241-fuime', 'ha-kode'), ('to_tie', 'abui1241-petle', 'kol'), ('to_tie', 'atim1239', 'kode')}
 {('to_tie', 'lama1277-watan', 'wido'), ('to_tie', 'lama1277-kiwan', 'wido'), ('to_tie', 'lama1277-horow', 'puĩ (wido)'), ('to_tie', 'lama1277-lamak', 'wido')}

  Just for clarity:
  There are also the following similarity classes shared between Alorese and LH.
  {('to_tie', 'lama1277-lewob', 'pũi'), ('to_tie', 'lama1277-wuake', 'pui'), ('to_tie', 'lama1277-lewog', 'puĩ'), ('to_tie', 'lama1277-baipi', 'puĩ'), ('to_tie', 'lama1277-waiwa', 'pũi'), ('to_tie', 'alor1247-besar', 'pɔhi'), ('to_tie', 'lama1277-paina', 'puinəŋ'), ('to_tie', 'lama1277-bama', 'puĩ'), ('to_tie', 'alor1247-pandai', 'pɔhi'), ('to_tie', 'lama1277-horow', 'puĩ (wido)'), ('to_tie', 'lama1277-waiba', 'pũĩ'), ('to_tie', 'lama1277-tanju', 'pui'), ('to_tie', 'lama1277-lewok', 'pui'), ('to_tie', 'lama1277-pukau', 'pũi'), ('to_tie', 'lama1277-belan', 'pui'), ('to_tie', 'alor1247-munas', 'pɔhi'), ('to_tie', 'lama1277-ileap', 'pui'), ('to_tie', 'lama1277-botun', 'puĩ'), ('to_tie', 'lama1277-adona', 'ˈpuĩ'), ('to_tie', 'lama1277-kalik', 'puˈʤu'), ('to_tie', 'lama1277-dulhi', 'puiŋ'), ('to_tie', 'lama1277-imulo', 'pui'), ('to_tie', 'lama1277-mulan', 'pui'), ('to_tie', 'lama1277-lewom', 'pũĩ'), ('to_tie', 'lama1277-ritae', 'pũĩ'), ('to_tie', 'alor1247-baran', "pɔ'hi:"), ('to_tie', 'lama1277-lewoi', 'ˈpuiŋ'), ('to_tie', 'lama1277-minga', 'pui'), ('to_tie', 'lama1277-lerek', 'ˈpui'), ('to_tie', 'lama1277-lamah', 'pui')}
  {('to_tie', 'lama1277-lewop', 'hoŋ'), ('to_tie', 'lama1277-kalik', 'soŋ'), ('to_tie', 'alor1247-pandai', 'hɔŋɔŋ'), ('to_tie', 'alor1247-pandai', 'hɔwɔ'), ('to_tie', 'keda1252-leuba', "ho'ŋɛn"), ('to_tie', 'keda1252-leuwa', 'hɔŋεn'), ('to_tie', 'lama1277-lewuk', 'soŋ'), ('to_tie', 'lama1277-merde', 'hoŋã'), ('to_tie', 'lama1277-lamat', 'hoŋe'), ('to_tie', 'lama1277-lewot', 'hoŋ'), ('to_tie', 'lama1277-lamal', 'hoŋã')}


Concept ‘to_cut’ is different between Alorese and Lamaholot.

 Alorese innovated the following forms:
 {('to_cut', 'alor1247-munas', 'hɔdaŋ')}
 Alorese shares the following forms with other AN languanges, but not Lamaholot:
 {('to_cut', 'alor1247-baran', 'pak:u'), ('to_cut', 'sika1262-tanai', 'poʔok'), ('to_cut', 'sarr1247-nule', 'bah'), ('to_cut', 'alor1247-pandai', "pə'kːu"), ('to_cut', 'baka1276', 'boj'), ('to_cut', 'alor1247-besar', 'pakːu'), ('to_cut', 'blag1240-tuntu', 'boki'), ('to_cut', 'lamm1241-westp', 'pai'), ('to_cut', 'blag1240-kulij', 'boj'), ('to_cut', 'pura1258', 'boi'), ('to_cut', 'dein1238', 'ba:')}
 One or more Lamaholot dialects innovated forms not found in Alorese:
 {('to_cut', 'sika1262-hewa', 'pati'), ('to_cut', 'kama1365', 'batat'), ('to_cut', 'blag1240-bama', 'pataki'), ('to_cut', 'kaer1234', "pa'tako"), ('to_cut', 'rett1240', 'pataki'), ('to_cut', 'sika1262-hewa', 'bati'), ('to_cut', 'sika1262-maume', 'pat̪i'), ('to_cut', 'blag1240-warsa', 'paʤ'), ('to_cut', 'lama1277-lewoi', 'ˈpətə'), ('to_cut', 'abui1241-takal', "bä.'te̞k"), ('to_cut', 'blag1240-warsa', 'pataki'), ('to_cut', 'sika1262-tanai', 'patar pati')}
 {('to_cut', 'lama1277-paina', 'bel(o)'), ('to_cut', 'lama1277-baipi', 'belo'), ('to_cut', 'lama1277-lerek', 'ˈbelo'), ('to_cut', 'lamm1241-westp', 'luʔuŋ'), ('to_cut', 'lama1277-lewoe', 'belu'), ('to_cut', 'lama1277-tanju', 'belo'), ('to_cut', 'keda1252-leuwa', 'bεl baʔ'), ('to_cut', 'keda1252-leuwa', 'bεluŋ'), ('to_cut', 'lama1277-kiwan', 'belo'), ('to_cut', 'keda1252', 'bel'), ('to_cut', 'lama1277-lewoi', 'ˈbelo'), ('to_cut', 'sika1262-tanai', 'poro'), ('to_cut', 'lama1277-imulo', 'bel'), ('to_cut', 'lama1277-lamak', 'belo'), ('to_cut', 'lama1277-merde', 'belo'), ('to_cut', 'lama1277-horow', 'belo'), ('to_cut', 'lama1277-botun', 'belo'), ('to_cut', 'lama1277-belan', 'belo'), ('to_cut', 'lama1277-adona', 'ˈbelo'), ('to_cut', 'lama1277-lewob', 'belo'), ('to_cut', 'lama1277-mulan', 'belo'), ('to_cut', 'lama1277-watan', 'belo'), ('to_cut', 'lama1277-lewom', 'belo'), ('to_cut', 'lama1277-ritae', 'belo'), ('to_cut', 'lama1277-minga', 'belu'), ('to_cut', 'lama1277-lewot', 'belu'), ('to_cut', 'lama1277-lewog', 'belo'), ('to_cut', 'lama1277-wuake', 'belo'), ('to_cut', 'lama1277-waiwa', 'belo'), ('to_cut', 'lama1277-waiba', 'belo'), ('to_cut', 'lama1277-lamah', 'belo'), ('to_cut', 'lama1277-lamal', 'belo'), ('to_cut', 'lama1277-lewop', 'belu'), ('to_cut', 'lama1277-dulhi', 'belo'), ('to_cut', 'lama1277-lewuk', 'belu'), ('to_cut', 'lama1277-pukau', 'belo'), ('to_cut', 'lama1277-lewok', 'belu'), ('to_cut', 'lamm1241-westp', 'bailaŋ'), ('to_cut', 'lama1277-ileap', 'belo'), ('to_cut', 'lama1277-kalik', 'belu'), ('to_cut', 'lama1277-lamat', 'belo'), ('to_cut', 'lama1277-kalik', 'ˈbelu'), ('to_cut', 'lama1277-bama', 'belo')}
 {('to_cut', 'keda1252-leuba', "pɛ'riʔ"), ('to_cut', 'lama1277-adona', 'ˈporoʔ'), ('to_cut', 'blag1240-nule', "pa'ras"), ('to_cut', 'keda1252-leuwa', 'pæriʔ piʔ')}


Concept ‘spider’ is different between Alorese and Lamaholot.

 Alorese innovated the following forms:
 {('spider', 'alor1247-munas', "kaŋ'ga 'rukaʔ")}
 Alorese shares the following forms with other AN languanges, but not Lamaholot:
 {('spider', 'blag1240-warsa', 'kakaraŋa'), ('spider', 'blag1240-bama', "kaka'raŋa"), ('spider', 'sika1262-hewa', 'kuku raka'), ('spider', 'alor1247-besar', "ka'raŋga"), ('spider', 'pura1258', "kaka'randa"), ('spider', 'baka1276', 'kraŋga'), ('spider', 'kaer1234', "ka'karaŋa"), ('spider', 'blag1240-nule', "kə'raŋa"), ('spider', 'blag1240-tuntu', "kaka'raŋa"), ('spider', 'alor1247-pandai', "k'lake")}
 One or more Lamaholot dialects innovated forms not found in Alorese:
 {('spider', 'lama1277-lerek', 'ˈlaka boˈrit'), ('spider', 'lama1277-kalik', 'lakboˈrit')}
 {('spider', 'lama1277-lewoi', 'ˈkɔbɔ')}
 {('spider', 'lama1277-adona', 'kabaˈrabat')}


Concept ‘correct’ is different between Alorese and Lamaholot.

 Alorese innovated the following forms:
 {('correct', 'alor1247-pandai', 'murok'), ('correct', 'alor1247-besar', 'muro'), ('correct', 'alor1247-munas', 'murɔk')}
 Alorese shares the following forms with other AN languanges, but not Lamaholot:
 {('correct', 'blag1240-kulij', 'molo'), ('correct', 'rett1240', 'molo'), ('correct', 'alor1247-pandai', 'mɔlɔŋ'), ('correct', 'blag1240-bama', 'molo'), ('correct', 'alor1247-baran', 'mallɔŋ'), ('correct', 'mamb1306', "do'lo:"), ('correct', 'rett1240', 'mɔlɔ'), ('correct', 'kaer1234', "mo'lo"), ('correct', 'baka1276', 'molo'), ('correct', 'blag1240-tuntu', 'molo')}
 One or more Lamaholot dialects innovated forms not found in Alorese:
 {('correct', 'lama1277-lamat', 'toʔa'), ('correct', 'lama1277-pukau', 'tokeʔẽ')}
 {('correct', 'lama1277-lerek', 'neˈmoi')}
 {('correct', 'lama1277-lewot', 'mui'), ('correct', 'lama1277-mulan', 'murɤ'), ('correct', 'lama1277-ritae', 'nə murã'), ('correct', 'lama1277-wuake', 'nã mure'), ('correct', 'lama1277-waiwa', 'murõ'), ('correct', 'lama1277-horow', 'murʔã'), ('correct', 'lama1277-kiwan', 'murʔã'), ('correct', 'lama1277-botun', 'murã'), ('correct', 'keda1252', 'muar'), ('correct', 'lama1277-lamak', 'murʔã'), ('correct', 'lama1277-belan', 'mur'), ('correct', 'lama1277-watan', 'murʔã'), ('correct', 'lama1277-bama', 'murə'), ('correct', 'lama1277-baipi', 'mu'), ('correct', 'lama1277-dulhi', 'murʔã'), ('correct', 'lama1277-tanju', 'murə'), ('correct', 'lama1277-merde', 'murã'), ('correct', 'lama1277-lamal', 'murã'), ('correct', 'lama1277-lamah', 'nã murã'), ('correct', 'lama1277-waiba', 'nə murə')}
 {('correct', 'lama1277-kalik', 'muˈʤək'), ('correct', 'keda1252-leuba', 'muar'), ('correct', 'lama1277-adona', 'muʔˈrɜ̃'), ('correct', 'lama1277-lewoi', 'ˈmurəŋ')}
 {('correct', 'lama1277-ileap', 'apadikən'), ('correct', 'lama1277-lewog', 'naʔandikã'), ('correct', 'lama1277-lewoe', 'na toʔõ'), ('correct', 'lama1277-lewob', 'nãdikã'), ('correct', 'lama1277-lewom', 'apadiken')}
 {('correct', 'lama1277-kalik', 'nər moi'), ('correct', 'lama1277-lewuk', 'nər moi'), ('correct', 'lama1277-imulo', 'na moi'), ('correct', 'lama1277-lewop', 'nara moi'), ('correct', 'lama1277-paina', 'nəmoi'), ('correct', 'lama1277-minga', 'namui'), ('correct', 'lama1277-lewok', 'na moi')}


Concept ‘to_push’ is different between Alorese and Lamaholot.

 Alorese innovated the following forms:
 {('to_push', 'alor1247-besar', 'natoŋ')}
 {('to_push', 'alor1247-munas', "gə'rɛng")}
 Alorese shares the following forms with other AN languanges, but not Lamaholot:
 {('to_push', 'keda1252-leuba', 'obaŋ'), ('to_push', 'blag1240-nule', 'tobaŋ'), ('to_push', 'blag1240-kulij', 'tobaŋ'), ('to_push', 'alor1247-munas', 'tobaŋ'), ('to_push', 'pura1258', "a'tobaŋ"), ('to_push', 'nede1245', 'doba'), ('to_push', 'alor1247-besar', 'tɔbaŋ'), ('to_push', 'alor1247-baran', 'tɔbaŋ'), ('to_push', 'blag1240-tuntu', "go'tobaŋ"), ('to_push', 'keda1252-leuwa', 'ɔ̘baŋ'), ('to_push', 'blag1240-bama', 'gotobaŋ'), ('to_push', 'rett1240', 'tɔmba'), ('to_push', 'alor1247-pandai', 'tɔbaŋ'), ('to_push', 'keda1252', 'obaŋ'), ('to_push', 'baka1276', 'tobaŋ'), ('to_push', 'alor1247-munas', 'ebaŋ'), ('to_push', 'blag1240-warsa', 'gotobaŋ'), ('to_push', 'kaer1234', "go'tobuŋ")}
 One or more Lamaholot dialects innovated forms not found in Alorese:
 {('to_push', 'lama1277-ileap', 'sokol'), ('to_push', 'sika1262-hewa', 'sogor'), ('to_push', 'lama1277-pukau', 'sogor'), ('to_push', 'lama1277-lewop', 'ogor'), ('to_push', 'lama1277-ritae', 'bogõ'), ('to_push', 'sika1262-hewa', 'soɡor'), ('to_push', 'lama1277-lamak', 'pogõ'), ('to_push', 'lama1277-lewot', 'bogor')}

  Just for clarity:
  There are also the following similarity classes shared between Alorese and LH.
  {('to_push', 'lama1277-minga', 'udur'), ('to_push', 'lama1277-adona', 'ˈtojɔ̃'), ('to_push', 'alor1247-besar', 'uruŋ'), ('to_push', 'mamb1306', 'dud'), ('to_push', 'lama1277-lewom', 'tojõ'), ('to_push', 'keda1252-leuwa', 'u̘ruʔ'), ('to_push', 'lama1277-lewob', 'odo'), ('to_push', 'fata1247', 'tuturɛ'), ('to_push', 'lama1277-watan', 'osõ'), ('to_push', 'lama1277-lamah', 'uduk'), ('to_push', 'lama1277-lamat', 'udũ'), ('to_push', 'tetu1245-vique', 'dudu'), ('to_push', 'lama1277-lewog', 'odo'), ('to_push', 'lama1277-dulhi', 'osõ'), ('to_push', 'tetu1246', 'dudu'), ('to_push', 'laka1255', 'dudu'), ('to_push', 'lama1277-mulan', 'udu'), ('to_push', 'lama1277-lamal', 'odo'), ('to_push', 'lama1277-lerek', 'ˈuduk'), ('to_push', 'maka1316', "du'dulu"), ('to_push', 'lama1277-kalik', 'uduk'), ('to_push', 'tuku1254', 'dudu'), ('to_push', 'lama1277-lewoe', 'udu'), ('to_push', 'lama1277-belan', 'uduk'), ('to_push', 'lama1277-waiba', 'tojõ'), ('to_push', 'lama1277-lewok', 'uduk'), ('to_push', 'lama1277-kalik', 'uˈdʊk'), ('to_push', 'lama1277-bama', 'odo'), ('to_push', 'idat1237', "a'dudu"), ('to_push', 'kema1243', 'tuka'), ('to_push', 'lama1277-kiwan', 'osõ'), ('to_push', 'adan1251-otvai', 'a’dodo'), ('to_push', 'lama1277-horow', 'toyõ'), ('to_push', 'hama1240', 'adʊdʊ'), ('to_push', 'adan1251-lawah', 'adoʔo'), ('to_push', 'lama1277-tanju', 'odo'), ('to_push', 'lama1277-lamal', 'uruk'), ('to_push', 'lama1277-merde', 'uduk'), ('to_push', 'lama1277-waiwa', 'toyõ'), ('to_push', 'kabo1247', 'odoʔo'), ('to_push', 'lama1277-baipi', 'odo'), ('to_push', 'lama1277-paina', 'udukaŋ'), ('to_push', 'lama1277-lewuk', 'uduk'), ('to_push', 'lama1277-botun', 'toyõ'), ('to_push', 'lama1277-wuake', 'udur'), ('to_push', 'lama1277-lewoi', 'ˈtojo'), ('to_push', 'rett1240', 'ɔrɔk'), ('to_push', 'tetu1245-suai', 'dudu'), ('to_push', 'lama1277-imulo', 'uduka')}


Concept ‘dirty’ is different between Alorese and Lamaholot.

 One or more Lamaholot dialects innovated forms not found in Alorese:
 {('dirty', 'lama1277-lewoe', 'raʔe')}
 {('dirty', 'lama1277-waiwa', 'milã'), ('dirty', 'sika1262-tanai', 'miʔak'), ('dirty', 'lama1277-lewuk', 'mila'), ('dirty', 'lama1277-minga', 'mila'), ('dirty', 'lama1277-ileap', 'milan'), ('dirty', 'lama1277-botun', 'milã'), ('dirty', 'lama1277-lerek', 'bəmiˈlan'), ('dirty', 'lama1277-watan', 'milã'), ('dirty', 'lama1277-kalik', 'miˈlan'), ('dirty', 'lama1277-lewok', 'milan'), ('dirty', 'lama1277-kiwan', 'milã'), ('dirty', 'lama1277-lamah', 'milã'), ('dirty', 'lama1277-lewog', 'milã'), ('dirty', 'lama1277-pukau', 'milã'), ('dirty', 'lama1277-paina', 'milan'), ('dirty', 'lama1277-lewom', 'milã'), ('dirty', 'lama1277-ritae', 'milã'), ('dirty', 'lama1277-bama', 'milã'), ('dirty', 'sika1262-hewa', 'milan'), ('dirty', 'lama1277-lewoi', 'ˈmilaŋ'), ('dirty', 'lama1277-horow', 'milã'), ('dirty', 'lama1277-lamak', 'milã'), ('dirty', 'lama1277-lewob', 'milã'), ('dirty', 'lama1277-merde', 'milã'), ('dirty', 'lama1277-lamal', 'milã'), ('dirty', 'lama1277-mulan', 'milɤ'), ('dirty', 'lama1277-waiba', 'milã'), ('dirty', 'lama1277-wuake', 'mila'), ('dirty', 'lama1277-lewot', 'mila'), ('dirty', 'lama1277-tanju', 'milã'), ('dirty', 'lama1277-kalik', 'milan'), ('dirty', 'lama1277-adona', 'miˈlã'), ('dirty', 'lama1277-baipi', 'milã'), ('dirty', 'lama1277-dulhi', 'milã'), ('dirty', 'lama1277-lewop', 'milan'), ('dirty', 'lama1277-imulo', 'milana')}
 {('dirty', 'lama1277-belan', 'wala')}

  Just for clarity:
  There are also the following similarity classes shared between Alorese and LH.
  {('dirty', 'alor1247-besar', "ka'lita"), ('dirty', 'rett1240', 'karita'), ('dirty', 'pura1258', "ka'rita"), ('dirty', 'blag1240-nule', "kə'ritak"), ('dirty', 'teiw1235', "kli'taɁ"), ('dirty', 'alor1247-pandai', "k'lita"), ('dirty', 'alor1247-munas', "k'litaʔ"), ('dirty', 'baka1276', 'klitak'), ('dirty', 'lama1277-lamat', 'prita'), ('dirty', 'blag1240-kulij', 'kəlitah'), ('dirty', 'alor1247-baran', "ka'lit:a")}


Concept ‘that’ is different between Alorese and Lamaholot.

 Alorese innovated the following forms:
 {('that', 'alor1247-pandai', 'klou')}
 {('that', 'alor1247-pandai', 'kalːi'), ('that', 'alor1247-munas', "kwə'lːi"), ('that', 'alor1247-pandai', "k'walːi")}
 One or more Lamaholot dialects innovated forms not found in Alorese:
 {('that', 'abui1241-takal', "('i.ti) nu:"), ('that', 'kema1243', 'nua'), ('that', 'p-cent2245-abvd', '*-na'), ('that', 'abui1241-petle', "i.'ti mu:"), ('that', 'p-mala1545-acd', '*i-na'), ('that', 'abui1241-takal', "ɔ.'rɔ nu:"), ('that', 'sawi1256', 'aŋanu'), ('that', 'pura1258', 'aŋu'), ('that', 'lama1277-dulhi', 'ne'), ('that', 'lama1277-adona', 'ˈnhẽː'), ('that', 'lama1277-lewoe', 'nene'), ('that', 'p-mala1545-abvd', '*i na')}
 {('that', 'lama1277-lewuk', 'məre')}
 {('that', 'lama1277-kalik', 'vo'), ('that', 'lama1277-kalik', 'vone')}
 {('that', 'lama1277-lamal', 'pe'), ('that', 'lama1277-lewom', 'peʔẽ'), ('that', 'lama1277-lewoi', 'ˈpeː'), ('that', 'lama1277-waiwa', 'pe'), ('that', 'lama1277-mulan', 'nəpe'), ('that', 'keda1252-leuba', "no'beʔ"), ('that', 'lama1277-baipi', 'pe'), ('that', 'lama1277-lewog', 'peʔẽ'), ('that', 'lama1277-wuake', 'nəpe'), ('that', 'lama1277-botun', 'pe'), ('that', 'lama1277-lamat', 'nəbe'), ('that', 'tetu1246', "nə'ba:"), ('that', 'lama1277-lerek', 'naˈbo'), ('that', 'lama1277-lamah', 'nəpe'), ('that', 'tetu1245-suai', "nɛ'ba:"), ('that', 'lama1277-horow', 'pe'), ('that', 'tetu1245-vique', "nɛ'ba:"), ('that', 'keda1252', 'nobeʔ'), ('that', 'lama1277-lamak', 'nəwe'), ('that', 'lama1277-ritae', 'pe'), ('that', 'lama1277-kiwan', 'nəpe'), ('that', 'lama1277-paina', 'nəbo'), ('that', 'lama1277-belan', 'pəpe'), ('that', 'lama1277-tanju', 'peʔẽ'), ('that', 'lama1277-bama', 'peʔẽ'), ('that', 'lama1277-ileap', 'pe '), ('that', 'lama1277-waiba', 'pe'), ('that', 'lama1277-watan', 'pe'), ('that', 'lama1277-merde', 'nəpe'), ('that', 'lama1277-kalik', 'bete')}

  Just for clarity:
  There are also the following similarity classes shared between Alorese and LH.
  {('that', 'lama1277-lewot', 'nakure'), ('that', 'lama1277-minga', 'nakre'), ('that', 'alor1247-pandai', 'kre'), ('that', 'lama1277-imulo', 'nakre'), ('that', 'lama1277-lewop', 'nakre')}
  {('that', 'alor1247-pandai', "kə'te"), ('that', 'alor1247-baran', ",ka'te"), ('that', 'alor1247-munas', "kə'tːe"), ('that', 'lama1277-lewok', 'dede'), ('that', 'lama1277-pukau', 'teteʔẽ'), ('that', 'alor1247-besar', "kə'te"), ('that', 'abui1241-takal', "('i.ti) tɔ"), ('that', 'wers1238-marit', 'gadi'), ('that', 'lama1277-lewob', 'teʔẽ'), ('that', 'p-mala1545-acd', '*i-di')}


Concept ‘rice_grains’ is different between Alorese and Lamaholot.

 Alorese innovated the following forms:
 {('rice_grains', 'alor1247-munas', "pare 'wuliŋ"), ('rice_grains', 'alor1247-pandai', "parɛ 'wuliŋ"), ('rice_grains', 'alor1247-besar', "pari mu'lɛŋ")}
 One or more Lamaholot dialects innovated forms not found in Alorese:
 {('rice_grains', 'lama1277-kalik', 'ˈlisɔ kaˈraʤ')}
 {('rice_grains', 'lama1277-lerek', 'kəˈluok ˈvuliran')}
 {('rice_grains', 'lama1277-lewoi', 'ˈtahaŋ ˈwuliŋ')}
 {('rice_grains', 'lama1277-adona', 'ˈwahaʔ')}


Concept ‘sick_painful’ is different between Alorese and Lamaholot.

 Alorese innovated the following forms:
 {('sick_painful', 'alor1247-pandai', "wə'kːiŋ p'latiŋ"), ('sick_painful', 'alor1247-besar', "pa'latiŋ")}
 One or more Lamaholot dialects innovated forms not found in Alorese:
 {('sick_painful', 'lama1277-lewoi', 'bəˈriŋi')}
 {('sick_painful', 'lama1277-kalik', 'ˈɡai')}
 {('sick_painful', 'lama1277-lerek', 'huˈhan')}

  Just for clarity:
  There are also the following similarity classes shared between Alorese and LH.
  {('sick_painful', 'alor1247-munas', "b'laraŋ"), ('sick_painful', 'sika1262-hewa', 'blaraʔ'), ('sick_painful', 'lama1277-adona', 'ˈbrara'), ('sick_painful', 'lama1277-adona', 'bɜˈrarʔa'), ('sick_painful', 'alor1247-besar', "bə'lara"), ('sick_painful', 'alor1247-pandai', "b'lara")}


Concept ‘to_wash’ is different between Alorese and Lamaholot.

 Alorese innovated the following forms:
 {('to_wash', 'alor1247-pandai', 'bema'), ('to_wash', 'alor1247-munas', 'bema'), ('to_wash', 'alor1247-besar', "bɛmɛ 'apa"), ('to_wash', 'alor1247-baran', 'bɛmɛ')}
 {('to_wash', 'alor1247-baran', 'huɛ'), ('to_wash', 'alor1247-munas', 'hue'), ('to_wash', 'alor1247-pandai', 'hue')}
 {('to_wash', 'dein1238', 'lanaŋ'), ('to_wash', 'lamm1241-westp', 'lamiŋ'), ('to_wash', 'hama1240', 'nalam'), ('to_wash', 'kabo1247', '(na)lam'), ('to_wash', 'adan1251-otvai', '’lam'), ('to_wash', 'adan1251-lawah', 'la:m'), ('to_wash', 'kafo1240', 'ulɑm'), ('to_wash', 'alor1247-munas', 'lamiŋ'), ('to_wash', 'alor1247-baran', 'lamiŋ'), ('to_wash', 'alor1247-pandai', 'lamiŋ')}
 Alorese shares the following forms with TAP languages:
 {('to_wash', 'alor1247-pandai', 'bema'), ('to_wash', 'alor1247-munas', 'bema'), ('to_wash', 'alor1247-besar', "bɛmɛ 'apa"), ('to_wash', 'alor1247-baran', 'bɛmɛ')}
 {('to_wash', 'alor1247-baran', 'huɛ'), ('to_wash', 'alor1247-munas', 'hue'), ('to_wash', 'alor1247-pandai', 'hue')}
 {('to_wash', 'dein1238', 'lanaŋ'), ('to_wash', 'lamm1241-westp', 'lamiŋ'), ('to_wash', 'hama1240', 'nalam'), ('to_wash', 'kabo1247', '(na)lam'), ('to_wash', 'adan1251-otvai', '’lam'), ('to_wash', 'adan1251-lawah', 'la:m'), ('to_wash', 'kafo1240', 'ulɑm'), ('to_wash', 'alor1247-munas', 'lamiŋ'), ('to_wash', 'alor1247-baran', 'lamiŋ'), ('to_wash', 'alor1247-pandai', 'lamiŋ')}
 One or more Lamaholot dialects innovated forms not found in Alorese:
 {('to_wash', 'lama1277-ileap', 'baha'), ('to_wash', 'sika1262-tanai', 'rasi'), ('to_wash', 'lama1277-pukau', 'baha'), ('to_wash', 'lama1277-paina', 'baha'), ('to_wash', 'tetu1245-suai', 'fasi'), ('to_wash', 'lama1277-lerek', 'ˈbahaŋ'), ('to_wash', 'lama1277-lamak', 'baha'), ('to_wash', 'tetu1246', 'fɑse'), ('to_wash', 'keda1252-leuba', "ba'hiŋ"), ('to_wash', 'lama1277-lewog', 'baha'), ('to_wash', 'keda1252', 'bahiŋ'), ('to_wash', 'lama1277-lewop', 'baha'), ('to_wash', 'lama1277-mulan', 'baha'), ('to_wash', 'lama1277-lewoi', 'ˈbaha'), ('to_wash', 'lama1277-adona', 'ˈbaha'), ('to_wash', 'lama1277-lamah', 'baha'), ('to_wash', 'lama1277-lamal', 'ba'), ('to_wash', 'fata1247', 'pahalɛ'), ('to_wash', 'lama1277-ritae', 'baha'), ('to_wash', 'lama1277-waiwa', 'bak'), ('to_wash', 'lama1277-baipi', 'baha'), ('to_wash', 'lama1277-horow', 'baha'), ('to_wash', 'p-mala1545-acd', '*basuq'), ('to_wash', 'lama1277-lewot', 'baha'), ('to_wash', 'lama1277-bama', 'baha'), ('to_wash', 'lama1277-watan', 'baha'), ('to_wash', 'lama1277-lewob', 'baha'), ('to_wash', 'lama1277-imulo', 'basa'), ('to_wash', 'lama1277-botun', 'baha'), ('to_wash', 'lama1277-merde', 'baha'), ('to_wash', 'lama1277-kalik', 'basa'), ('to_wash', 'lama1277-kiwan', 'baha'), ('to_wash', 'lama1277-tanju', 'baha'), ('to_wash', 'tetu1245-vique', 'fasi'), ('to_wash', 'lama1277-waiba', 'baha'), ('to_wash', 'lama1277-lewuk', 'basa'), ('to_wash', 'lama1277-kalik', 'baˈsa'), ('to_wash', 'lama1277-dulhi', 'baha'), ('to_wash', 'lama1277-lewok', 'basa'), ('to_wash', 'lama1277-lewoe', 'baha'), ('to_wash', 'lama1277-lamat', 'baha')}
 {('to_wash', 'lama1277-kalik', 'ˈreup')}
 {('to_wash', 'mamb1306', 'has'), ('to_wash', 'lama1277-wuake', 'sesa'), ('to_wash', 'tuku1254', "kɑ'hɑsa"), ('to_wash', 'kelo1247-hopte', "ʔu'han"), ('to_wash', 'lama1277-belan', 'səsa'), ('to_wash', 'idat1237', 'hase'), ('to_wash', 'kema1243', 'hɑsa'), ('to_wash', 'laka1255', 'hasɛ')}
 {('to_wash', 'lama1277-minga', 'pusɤ'), ('to_wash', 'lama1277-lewoi', 'ˈpuʔu'), ('to_wash', 'sika1262-hewa', 'puʔu'), ('to_wash', 'nede1245', 'bugi'), ('to_wash', 'lama1277-lamal', 'pu'), ('to_wash', 'lama1277-kalik', 'pus'), ('to_wash', 'lama1277-lewom', 'puʔũ')}


Concept ‘to_say’ is different between Alorese and Lamaholot.

 One or more Lamaholot dialects innovated forms not found in Alorese:
 {('to_say', 'lama1277-merde', 'gasip'), ('to_say', 'lama1277-minga', 'gasi'), ('to_say', 'lama1277-imulo', 'gas'), ('to_say', 'kira1248', 'gapi')}
 {('to_say', 'lama1277-lerek', 'pəˈnua'), ('to_say', 'lama1277-kalik', 'pəˈnua')}
 {('to_say', 'lama1277-paina', 'gəpar')}
 {('to_say', 'lama1277-adona', 'pɜˈʔulũ')}
 {('to_say', 'laka1255', 'da:'), ('to_say', 'lama1277-lewoi', 'ˈkɔda'), ('to_say', 'lama1277-ileap', 'koda'), ('to_say', 'mamb1306', 'tou'), ('to_say', 'fata1247', 'taɁa'), ('to_say', 'tetu1245-suai', 'katak')}
 {('to_say', 'sika1262-hewa', 'həron'), ('to_say', 'sika1262-tanai', 'həro-ŋ'), ('to_say', 'lama1277-pukau', 'hərõ'), ('to_say', 'nede1245', 'biraŋ'), ('to_say', 'sika1262-tanai', 'kiri-ŋ')}

  Just for clarity:
  There are also the following similarity classes shared between Alorese and LH.
  {('to_say', 'lama1277-lerek', 'təˈdək'), ('to_say', 'alor1247-besar', 'tutu'), ('to_say', 'lama1277-wuake', 'tutu'), ('to_say', 'buna1278-bobon', 'tetʃu'), ('to_say', 'sika1262-maume', 't̪ut̪ur'), ('to_say', 'tetu1245-vique', "ha'tɛtɛŋ"), ('to_say', 'blag1240-tuntu', 'tutuk'), ('to_say', 'keda1252-leuba', "tutuʔ 'nanaŋ"), ('to_say', 'rett1240', 'tutuk'), ('to_say', 'kaer1234', "tu'tuk"), ('to_say', 'p-mala1545-abvd', '*tutur'), ('to_say', 'lama1277-lewop', 'tədək'), ('to_say', 'lama1277-lewoe', 'təde'), ('to_say', 'lama1277-lamat', 'təde'), ('to_say', 'lama1277-lewoi', 'ˈtutuʔ'), ('to_say', 'lama1277-kalik', 'tədək'), ('to_say', 'buna1278-malia', 'tɛtʃuh'), ('to_say', 'p-cent2245-abvd', '*tutur'), ('to_say', 'sika1262-tanai', 'tutur bəta'), ('to_say', 'blag1240-nule', 'tutuktutuk'), ('to_say', 'tetu1246', 'hateten'), ('to_say', 'keda1252-leuba', 'tutuʔ')}
  {('to_say', 'lama1277-mulan', 'mɤi'), ('to_say', 'sika1262-hewa', 'marĩ'), ('to_say', 'lama1277-lewom', 'marĩ'), ('to_say', 'p-mala1545-abvd', '*kaɾi'), ('to_say', 'lama1277-ritae', 'marĩ'), ('to_say', 'lama1277-lewuk', 'maik'), ('to_say', 'lama1277-lewot', 'maji'), ('to_say', 'lama1277-bama', 'marĩ'), ('to_say', 'lama1277-horow', 'marĩ'), ('to_say', 'lama1277-belan', 'mari'), ('to_say', 'alor1247-pandai', 'mariŋ'), ('to_say', 'lama1277-lamal', 'mari'), ('to_say', 'hama1240', 'matɛŋ'), ('to_say', 'lama1277-watan', 'marĩ'), ('to_say', 'lama1277-tanju', 'mari'), ('to_say', 'lama1277-kiwan', 'mari'), ('to_say', 'lama1277-lewog', 'marĩ'), ('to_say', 'lama1277-lewob', 'mari'), ('to_say', 'alor1247-baran', "ma'rĩ:"), ('to_say', 'lama1277-baipi', 'mari'), ('to_say', 'sika1262-hewa', 'marin'), ('to_say', 'lama1277-lewok', 'maik'), ('to_say', 'lama1277-lamah', 'mari'), ('to_say', 'kuii1253', 'lakari'), ('to_say', 'lama1277-waiwa', 'marĩ'), ('to_say', 'lama1277-lamak', 'mari'), ('to_say', 'lama1277-waiba', 'marĩ'), ('to_say', 'alor1247-munas', 'mariŋ'), ('to_say', 'lama1277-botun', 'marĩ'), ('to_say', 'lama1277-dulhi', 'marĩ')}


Concept ‘lazy’ is different between Alorese and Lamaholot.

 Alorese shares the following forms with TAP languages:
 {('lazy', 'adan1251-lawah', 'lahunu'), ('lazy', 'alor1247-pandai', "p'lihaŋ"), ('lazy', 'alor1247-munas', "p'lihaŋ"), ('lazy', 'adan1251-otvai', 'la’hun')}
 One or more Lamaholot dialects innovated forms not found in Alorese:
 {('lazy', 'lama1277-kalik', 'bəˈnɔɡəl')}
 {('lazy', 'lama1277-lerek', 'meˈtəl')}
 {('lazy', 'lama1277-lewoi', 'kəˈmoɡə')}
 {('lazy', 'lama1277-adona', 'ˈhadɜ')}

  Just for clarity:
  There are also the following similarity classes shared between Alorese and LH.
  {('lazy', 'alor1247-besar', "pəma'lasa"), ('lazy', 'lama1277-lerek', 'pemalˈha')}


Concept ‘old_people’ is different between Alorese and Lamaholot.

 Alorese innovated the following forms:
 {('old_people', 'alor1247-pandai', "ɔraŋ 'tua")}
 {('old_people', 'alor1247-baran', "gambɛ-'gambɛ"), ('old_people', 'alor1247-pandai', 'gambeiŋ')}
 {('old_people', 'alor1247-pandai', 'wabeiŋ')}
 {('old_people', 'alor1247-munas', "mə'sia 'tua")}
 {('old_people', 'alor1247-baran', "ina-'ina")}
 One or more Lamaholot dialects innovated forms not found in Alorese:
 {('old_people', 'lama1277-lamah', 'magũ'), ('old_people', 'lama1277-lamal', 'magu'), ('old_people', 'lama1277-lewot', 'maguja'), ('old_people', 'lama1277-lewok', 'magu'), ('old_people', 'lama1277-lewuk', 'magun'), ('old_people', 'lama1277-mulan', 'magu'), ('old_people', 'lama1277-kalik', 'magun'), ('old_people', 'lama1277-wuake', 'magu'), ('old_people', 'lama1277-imulo', 'magun'), ('old_people', 'lama1277-belan', 'magu'), ('old_people', 'lama1277-merde', 'magũ'), ('old_people', 'lama1277-lamat', 'magũ'), ('old_people', 'lama1277-lewoe', 'magũ')}
 {('old_people', 'tuku1254', 'mekei hine'), ('old_people', 'lama1277-kalik', 'maˈɡuna'), ('old_people', 'tuku1254', "mə'kei 'mane"), ('old_people', 'lama1277-lerek', 'ˈtua ˈmaɡu')}
 {('old_people', 'lama1277-lewob', 'tua'), ('old_people', 'lama1277-pukau', 'duʔã'), ('old_people', 'sika1262-hewa', 'duan')}
 {('old_people', 'lama1277-adona', 'tɜˈnuʔe'), ('old_people', 'lama1277-lewoi', 'ˈtənueŋ')}
 {('old_people', 'lama1277-baipi', 'tənue'), ('old_people', 'lama1277-dulhi', 'tənue'), ('old_people', 'lama1277-bama', 'tənue'), ('old_people', 'lama1277-lewog', 'tənue'), ('old_people', 'lama1277-tanju', 'tənue'), ('old_people', 'lama1277-horow', 'tənuʔe'), ('old_people', 'lama1277-kiwan', 'tənuʔe'), ('old_people', 'lama1277-paina', 'tuanakən'), ('old_people', 'lama1277-lewom', 'tənuẽ'), ('old_people', 'lama1277-minga', 'tənəras'), ('old_people', 'lama1277-waiba', 'tənue'), ('old_people', 'lama1277-lamak', 'tənuʔe'), ('old_people', 'lama1277-ileap', 'nuhen'), ('old_people', 'lama1277-lewop', 'tənərahən'), ('old_people', 'lama1277-waiwa', 'tənuʔe'), ('old_people', 'lama1277-botun', 'tənuʔe'), ('old_people', 'lama1277-ritae', 'tənuʔe'), ('old_people', 'lama1277-watan', 'tənuʔe')}

  Just for clarity:
  There are also the following similarity classes shared between Alorese and LH.
  {('old_people', 'p-aust1307-abvd', '*ma-tuqaS'), ('old_people', 'p-mala1545-acd', '*tuqah'), ('old_people', 'p-cent2245-abvd', '*tuqa'), ('old_people', 'alor1247-pandai', 'tua'), ('old_people', 'lama1277-kalik', 'tuˈana'), ('old_people', 'p-mala1545-abvd', '*ma-tuqah')}


Concept ‘rough’ is different between Alorese and Lamaholot.

 Alorese shares the following forms with TAP languages:
 {('rough', 'sarr1247-adiab', 'qa:l'), ('rough', 'alor1247-pandai', 'bɔgal'), ('rough', 'alor1247-munas', 'bogal')}
 Alorese shares the following forms with other AN languanges, but not Lamaholot:
 {('rough', 'kaer1234', "ko'taŋ"), ('rough', 'p-mala1545-acd', '*garaŋ'), ('rough', 'blag1240-tuntu', "ka'rataŋ"), ('rough', 'keda1252-leuba', "ka'raŋ"), ('rough', 'alor1247-besar', 'karɔ'), ('rough', 'lamm1241-westp', 'kora'), ('rough', 'nede1245', 'arad:a')}
 One or more Lamaholot dialects innovated forms not found in Alorese:
 {('rough', 'lama1277-lewoi', 'podoˈrodo')}
 {('rough', 'lama1277-kalik', 'ɡroˈsiŋ')}
 {('rough', 'lama1277-adona', 'ˈɡaʔak')}


Concept ‘field’ is different between Alorese and Lamaholot.

 Alorese shares the following forms with TAP languages:
 {('field', 'alor1247-munas', "ɛkaŋ 'lata"), ('field', 'alor1247-besar', 'lata'), ('field', 'kuii1253', 'lat'), ('field', 'alor1247-pandai', 'lata')}
 One or more Lamaholot dialects innovated forms not found in Alorese:
 {('field', 'lama1277-lerek', 'ˈkurum oˈran')}
 {('field', 'lama1277-adona', 'nɜphã'), ('field', 'lama1277-kalik', 'nəˈpan')}
 {('field', 'lama1277-lewoi', 'ˈhɔkɛŋ')}


Concept ‘few’ is different between Alorese and Lamaholot.

 One or more Lamaholot dialects innovated forms not found in Alorese:
 {('few', 'lama1277-belan', 'ue'), ('few', 'lama1277-ileap', 'buhe'), ('few', 'lama1277-mulan', 'ue'), ('few', 'lama1277-lamah', 'uhe'), ('few', 'lama1277-lerek', 'ˈue'), ('few', 'lama1277-merde', 'uhe')}
 {('few', 'tetu1245-suai', "uan 'ida"), ('few', 'tuku1254', "sə'niʔit"), ('few', 'lama1277-pukau', 'niutou'), ('few', 'tuku1254', "se'niʔit nana"), ('few', 'tetu1245-vique', "uan 'ida")}
 {('few', 'lama1277-lewoi', 'bəˈrua'), ('few', 'lama1277-lewog', 'bərua'), ('few', 'lama1277-lamat', 'ərahue'), ('few', 'lama1277-bama', 'bərua'), ('few', 'lama1277-ritae', 'bəruna')}
 {('few', 'lama1277-adona', 'kuluk')}
 {('few', 'keda1252-leuwa', 'uraŋ'), ('few', 'lama1277-tanju', 'kurã'), ('few', 'keda1252', 'uraŋ')}
 {('few', 'lama1277-baipi', 'sələ'), ('few', 'lama1277-waiba', 'sələ'), ('few', 'lama1277-lewom', 'səle')}
 {('few', 'teiw1235', "ga'ne:g"), ('few', 'lama1277-kalik', 'kənako'), ('few', 'lama1277-lewop', 'kənakofai'), ('few', 'lama1277-lewot', 'kənako'), ('few', 'sika1262-hewa', 'anak ha'), ('few', 'lama1277-imulo', 'kənakofai'), ('few', 'lama1277-lewok', 'kənəko'), ('few', 'sika1262-hewa', 'anakha'), ('few', 'kelo1247-hopte', "kənit 'a:k"), ('few', 'lama1277-kalik', 'kənaˈkɔt'), ('few', 'lama1277-lewoe', 'kana'), ('few', 'lama1277-lewuk', 'kənako')}

  Just for clarity:
  There are also the following similarity classes shared between Alorese and LH.
  {('few', 'lama1277-adona', 'ˈʔɜsiʔ'), ('few', 'lama1277-lamal', 'usi'), ('few', 'alor1247-besar', 'usu'), ('few', 'alor1247-munas', 'wisuʔ'), ('few', 'alor1247-baran', "ʔata 'ʔusu"), ('few', 'lama1277-lamak', 'əsi'), ('few', 'lama1277-lewob', 'kəsə'), ('few', 'lama1277-paina', 'ətihi'), ('few', 'lama1277-kiwan', 'əsi'), ('few', 'lama1277-waiwa', 'əsiʔ'), ('few', 'lama1277-watan', 'əsi'), ('few', 'lama1277-wuake', 'usi'), ('few', 'alor1247-pandai', 'isu'), ('few', 'lama1277-botun', 'usi'), ('few', 'lama1277-horow', 'əsiʔ'), ('few', 'lama1277-dulhi', 'əsi'), ('few', 'alor1247-pandai', 'wisu'), ('few', 'lama1277-minga', 'bosi'), ('few', 'lama1277-lerek', 'ətˈhi'), ('few', 'sika1262-tanai', 'kəsik ha')}


Concept ‘twilight_dawn’ is different between Alorese and Lamaholot.

 Alorese innovated the following forms:
 {('twilight_dawn', 'alor1247-pandai', "manu 'kɔkɔŋ")}
 {('twilight_dawn', 'alor1247-pandai', 'subu')}
 One or more Lamaholot dialects innovated forms not found in Alorese:
 {('twilight_dawn', 'lama1277-kalik', 'ˈeka buˈʤak'), ('twilight_dawn', 'lama1277-lerek', 'ekan buˈjak')}

  Just for clarity:
  There are also the following similarity classes shared between Alorese and LH.
  {('twilight_dawn', 'alor1247-besar', "ɛkaŋ 'dahɛ ta'ranaŋ"), ('twilight_dawn', 'lama1277-adona', "ʔekã 'dahe"), ('twilight_dawn', 'alor1247-munas', "ɛkaŋ 'dahɛk"), ('twilight_dawn', 'alor1247-pandai', "ɛkaŋ 'dahek"), ('twilight_dawn', 'lama1277-lewoi', 'ɛkaŋˈdahɛʔ')}


Concept ‘ant’ is different between Alorese and Lamaholot.

 One or more Lamaholot dialects innovated forms not found in Alorese:
 {('ant', 'lama1277-kalik', 'təmiˈsi'), ('ant', 'lama1277-kalik', 'təmiˈsɛr'), ('ant', 'lama1277-lewoi', 'təˈmihe'), ('ant', 'lama1277-lerek', 'temiˈher'), ('ant', 'sika1262-hewa', 'mihe')}

  Just for clarity:
  There are also the following similarity classes shared between Alorese and LH.
  {('ant', 'lamm1241-westp', 'karé'), ('ant', 'alor1247-pandai', "ka'rːe"), ('ant', 'alor1247-munas', "kə'rːek"), ('ant', 'lama1277-adona', 'kɜˈmɜrɜk'), ('ant', 'alor1247-besar', "ka'rːe"), ('ant', 'dein1238', 'kre'), ('ant', 'blag1240-nule', "kə're"), ('ant', 'baka1276', 'kere'), ('ant', 'blag1240-kulij', 'ker:e')}


Concept ‘fever’ is different between Alorese and Lamaholot.

 Alorese innovated the following forms:
 {('fever', 'alor1247-besar', "ta'fefa")}
 One or more Lamaholot dialects innovated forms not found in Alorese:
 {('fever', 'lama1277-kalik', 'kaˈti ɡeˈɡet')}
 {('fever', 'blag1240-warsa', 'biriŋ'), ('fever', 'blag1240-bama', 'biriŋ'), ('fever', 'pura1258', "wa'wiriŋ"), ('fever', 'lama1277-lewoi', 'bəˈriŋin'), ('fever', 'baka1276', 'bi biriŋ'), ('fever', 'blag1240-kulij', 'bibiriŋ'), ('fever', 'blag1240-nule', 'viviriŋ'), ('fever', 'blag1240-tuntu', 'biriŋ')}
 {('fever', 'lama1277-lerek', 'puˈjuk')}

  Just for clarity:
  There are also the following similarity classes shared between Alorese and LH.
  {('fever', 'alor1247-pandai', "ɔnɔŋ 'platiŋ"), ('fever', 'lama1277-adona', 'pɜˈlatekɜt'), ('fever', 'alor1247-pandai', "wə'kːiŋ 'platiŋ"), ('fever', 'alor1247-munas', "p'latiŋ ə'kɛr")}


Concept ‘at’ is different between Alorese and Lamaholot.

 Alorese innovated the following forms:
 {('at', 'alor1247-baran', 'ʔunuŋ')}
 {('at', 'alor1247-pandai', 'ɔrɔ'), ('at', 'alor1247-besar', 'ɔro')}
 {('at', 'alor1247-munas', "go 'ke 'laraŋ 'ɔnɔŋ")}
 {('at', 'alor1247-pandai', 'kɛ')}
 {('at', 'alor1247-besar', 'ali'), ('at', 'sawi1256', 'nuli'), ('at', 'alor1247-munas', "və'lːi")}
 Alorese shares the following forms with TAP languages:
 {('at', 'alor1247-baran', 'ʔunuŋ')}
 {('at', 'alor1247-pandai', 'ɔrɔ'), ('at', 'alor1247-besar', 'ɔro')}
 {('at', 'alor1247-munas', "go 'ke 'laraŋ 'ɔnɔŋ")}
 {('at', 'alor1247-pandai', 'kɛ')}
 {('at', 'alor1247-besar', 'ali'), ('at', 'sawi1256', 'nuli'), ('at', 'alor1247-munas', "və'lːi")}
 One or more Lamaholot dialects innovated forms not found in Alorese:
 {('at', 'lama1277-lerek', 'nəvəl')}
 {('at', 'lama1277-lamal', 'lau'), ('at', 'lama1277-lewot', 'lau'), ('at', 'lama1277-lewob', 'lau'), ('at', 'lama1277-mulan', 'lau')}
 {('at', 'lama1277-lamat', 'tubə')}
 {('at', 'lama1277-botun', 'di'), ('at', 'lama1277-merde', 'tə'), ('at', 'lama1277-watan', 'di'), ('at', 'lama1277-belan', 'di'), ('at', 'keda1252', 'di'), ('at', 'lama1277-imulo', 'di'), ('at', 'lama1277-ileap', 'tɤ'), ('at', 'lama1277-lamak', 'di'), ('at', 'lama1277-lamah', 'di'), ('at', 'lama1277-dulhi', 'si'), ('at', 'lama1277-baipi', 'di'), ('at', 'lama1277-waiwa', 'tə'), ('at', 'lama1277-horow', 'tə'), ('at', 'lama1277-kiwan', 'də'), ('at', 'lama1277-wuake', 'de')}
 {('at', 'lama1277-lewop', 'je'), ('at', 'lama1277-paina', 'je'), ('at', 'lama1277-lewot', 'ju'), ('at', 'lama1277-lewok', 'je'), ('at', 'lama1277-kalik', 'je')}
 {('at', 'lama1277-lerek', 'nədʒ͡u'), ('at', 'lama1277-lerek', 'nədʒ͡e')}
 {('at', 'lama1277-lewoi', 'ˈpi'), ('at', 'p-mala1545-acd', '*ba'), ('at', 'lama1277-adona', 'pi'), ('at', 'koto1251', 'n-bi'), ('at', 'keda1252-leuba', 'bɛʔ')}
 {('at', 'lama1277-tanju', 'pi'), ('at', 'lama1277-lewog', 'pi'), ('at', 'lama1277-ritae', 'pi'), ('at', 'lama1277-bama', 'pi')}
 {('at', 'lama1277-pukau', 'wəli'), ('at', 'lama1277-minga', 'fəli'), ('at', 'lama1277-lewuk', 'wəli'), ('at', 'lama1277-waiba', 'wəli'), ('at', 'lama1277-lewom', 'wəli'), ('at', 'lama1277-lewoe', 'wəli')}
 {('at', 'keda1252-leuba', 'diʔ'), ('at', 'p-mala1545-abvd', '*di'), ('at', 'lama1277-adona', 'dɜˈhi'), ('at', 'p-aust1307-abvd', '*i'), ('at', 'p-mala1545-abvd', '*i'), ('at', 'buna1278-malia', "a'ʧih"), ('at', 'laka1255', 'de:i'), ('at', 'p-mala1545-acd', '*di'), ('at', 'lama1277-adona', 'dɜ'), ('at', 'kema1243', 'dia'), ('at', 'p-cent2245-abvd', '*di'), ('at', 'p-aust1307-abvd', '*di'), ('at', 'rett1240', 'ʤia')}
 {('at', 'lama1277-kalik', 'lane'), ('at', 'lama1277-kalik', 'la')}
 {('at', 'lama1277-lewom', 'rae'), ('at', 'lama1277-lamal', 'rae'), ('at', 'lama1277-lewob', 'rae'), ('at', 'lama1277-waiba', 'rae'), ('at', 'lama1277-mulan', 'rae')}


Concept ‘to_hunt’ is different between Alorese and Lamaholot.

 Alorese innovated the following forms:
 {('to_hunt', 'alor1247-besar', 'kɔri'), ('to_hunt', 'kelo1247-hopte', 'ku:r'), ('to_hunt', 'rett1240', 'kɔli'), ('to_hunt', 'rett1240', 'koli')}
 {('to_hunt', 'alor1247-munas', "gɛna 'ɛkaŋ"), ('to_hunt', 'alor1247-pandai', "gɛna 'ɛkaŋ")}
 Alorese shares the following forms with TAP languages:
 {('to_hunt', 'alor1247-besar', 'kɔri'), ('to_hunt', 'kelo1247-hopte', 'ku:r'), ('to_hunt', 'rett1240', 'kɔli'), ('to_hunt', 'rett1240', 'koli')}
 {('to_hunt', 'alor1247-munas', "gɛna 'ɛkaŋ"), ('to_hunt', 'alor1247-pandai', "gɛna 'ɛkaŋ")}
 One or more Lamaholot dialects innovated forms not found in Alorese:
 {('to_hunt', 'lama1277-lewoi', 'pəˈrehaʔ'), ('to_hunt', 'lama1277-lewog', 'pəreha'), ('to_hunt', 'mamb1306', "pɑɛ 'ʔas"), ('to_hunt', 'lama1277-pukau', 'ləraʔ'), ('to_hunt', 'lama1277-bama', 'pəreha'), ('to_hunt', 'lama1277-lewob', 'lərakə')}
 {('to_hunt', 'lama1277-tanju', 'hewa'), ('to_hunt', 'lama1277-waiba', 'hewã'), ('to_hunt', 'lama1277-lewom', 'hewa')}
 {('to_hunt', 'keda1252', 'deruŋ'), ('to_hunt', 'tuku1254', 'tiru'), ('to_hunt', 'kuii1253', 'peteri'), ('to_hunt', 'keda1252-leuwa', 'dεruŋ dæŋ'), ('to_hunt', 'lama1277-lewoe', 'deru'), ('to_hunt', 'keda1252-leuba', "de'ruŋ"), ('to_hunt', 'pura1258', "a'teri")}
 {('to_hunt', 'lama1277-merde', 'welok'), ('to_hunt', 'lama1277-mulan', 'welo'), ('to_hunt', 'teiw1235', "i wa'rak"), ('to_hunt', 'lama1277-paina', 'welok'), ('to_hunt', 'lama1277-lamat', 'welo')}
 {('to_hunt', 'lama1277-baipi', 'hewãbati'), ('to_hunt', 'lama1277-ritae', 'batĩ'), ('to_hunt', 'lama1277-lamah', 'batĩ'), ('to_hunt', 'lama1277-horow', 'batĩ'), ('to_hunt', 'lama1277-lamal', 'bati'), ('to_hunt', 'lama1277-lerek', 'baˈti'), ('to_hunt', 'lama1277-belan', 'bati'), ('to_hunt', 'tuku1254', 'pa:m'), ('to_hunt', 'lama1277-kalik', 'baˈti'), ('to_hunt', 'lama1277-adona', 'ˈbatĩ'), ('to_hunt', 'lama1277-lewot', 'bati'), ('to_hunt', 'lama1277-imulo', 'bati'), ('to_hunt', 'lama1277-botun', 'batĩ'), ('to_hunt', 'lama1277-lewop', 'bati'), ('to_hunt', 'lama1277-dulhi', 'batiŋ'), ('to_hunt', 'lama1277-watan', 'batʔ'), ('to_hunt', 'lama1277-kiwan', 'bati'), ('to_hunt', 'lama1277-lewuk', 'bati'), ('to_hunt', 'lama1277-ileap', 'batiŋən'), ('to_hunt', 'lama1277-waiwa', 'batĩ'), ('to_hunt', 'lama1277-lewok', 'bati'), ('to_hunt', 'lama1277-kalik', 'batin')}
 {('to_hunt', 'lama1277-wuake', 'hapõ'), ('to_hunt', 'lama1277-minga', 'apu')}

  Just for clarity:
  There are also the following similarity classes shared between Alorese and LH.
  {('to_hunt', 'alor1247-baran', 'tut:ɛ'), ('to_hunt', 'lama1277-lamak', 'dore'), ('to_hunt', 'tetu1245-vique', 'tuda'), ('to_hunt', 'tetu1245-suai', 'tuda')}


Concept ‘to_burn_clear_land’ is different between Alorese and Lamaholot.

 One or more Lamaholot dialects innovated forms not found in Alorese:
 {('to_burn_clear_land', 'lama1277-minga', 'buaf')}
 {('to_burn_clear_land', 'teiw1235', '(maxar) de:Ɂ'), ('to_burn_clear_land', 'lama1277-lamat', 'daʔe'), ('to_burn_clear_land', 'lama1277-lewoe', 'deʔe')}

  Just for clarity:
  There are also the following similarity classes shared between Alorese and LH.
  {('to_burn_clear_land', 'lamm1241-westp', 'toŋ'), ('to_burn_clear_land', 'lama1277-ritae', 'tuno'), ('to_burn_clear_land', 'lama1277-lamak', 'tuno'), ('to_burn_clear_land', 'lama1277-lewob', 'tuno'), ('to_burn_clear_land', 'lama1277-pukau', 'tuno'), ('to_burn_clear_land', 'alor1247-baran', 'tun:ɔ'), ('to_burn_clear_land', 'lama1277-waiwa', 'tuno'), ('to_burn_clear_land', 'lama1277-merde', 'tuno'), ('to_burn_clear_land', 'p-mala1545-abvd', '*tunu'), ('to_burn_clear_land', 'lama1277-horow', 'tuno'), ('to_burn_clear_land', 'lama1277-mulan', 'tuno'), ('to_burn_clear_land', 'lama1277-tanju', 'tuno'), ('to_burn_clear_land', 'p-cent2245-abvd', '*tunu'), ('to_burn_clear_land', 'lama1277-kalik', 'ˈtunɔ'), ('to_burn_clear_land', 'keda1252', 'tun')}
  {('to_burn_clear_land', 'lama1277-lewok', 'papi'), ('to_burn_clear_land', 'lama1277-lerek', 'ˈpapi ˈole'), ('to_burn_clear_land', 'rett1240', 'gapia'), ('to_burn_clear_land', 'lama1277-lamal', 'papi'), ('to_burn_clear_land', 'lama1277-imulo', 'papi'), ('to_burn_clear_land', 'rett1240', 'ilgapia'), ('to_burn_clear_land', 'lama1277-lewot', 'papi'), ('to_burn_clear_land', 'keda1252-leuba', 'paʔ (lumar)'), ('to_burn_clear_land', 'lama1277-lewop', 'papi'), ('to_burn_clear_land', 'lama1277-kalik', 'paˈpi'), ('to_burn_clear_land', 'alor1247-pandai', 'papiŋ'), ('to_burn_clear_land', 'lama1277-kalik', 'papi'), ('to_burn_clear_land', 'keda1252-leuwa', 'paʔ bεɔʔ'), ('to_burn_clear_land', 'lama1277-lewuk', 'papi'), ('to_burn_clear_land', 'alor1247-munas', 'papiŋ')}
  {('to_burn_clear_land', 'lama1277-waiba', 'səru'), ('to_burn_clear_land', 'lama1277-lewom', 'səru'), ('to_burn_clear_land', 'lama1277-wuake', 'səru'), ('to_burn_clear_land', 'lama1277-lamah', 'səru'), ('to_burn_clear_land', 'lama1277-watan', 'səru'), ('to_burn_clear_land', 'lama1277-ileap', 'səru'), ('to_burn_clear_land', 'lama1277-botun', 'səru'), ('to_burn_clear_land', 'lama1277-adona', 'ˈsɜruʔ'), ('to_burn_clear_land', 'lama1277-dulhi', 'səru'), ('to_burn_clear_land', 'alor1247-besar', "saru 'ɛkaŋ"), ('to_burn_clear_land', 'lama1277-baipi', 'səru'), ('to_burn_clear_land', 'lama1277-lewog', 'səru'), ('to_burn_clear_land', 'lama1277-kiwan', 'səru'), ('to_burn_clear_land', 'alor1247-pandai', "sa'rːu"), ('to_burn_clear_land', 'lama1277-bama', 'səru'), ('to_burn_clear_land', 'alor1247-pandai', "sa'rːu 'ɛkaŋ"), ('to_burn_clear_land', 'lama1277-paina', 'talekaŋ'), ('to_burn_clear_land', 'lama1277-lewoi', 'seˈruʔ'), ('to_burn_clear_land', 'lama1277-belan', 'səru')}


Concept ‘old_worn_house’ is different between Alorese and Lamaholot.

 Alorese innovated the following forms:
 {('old_worn_house', 'alor1247-pandai', "uma 'oki")}
 {('old_worn_house', 'alor1247-munas', "uma 'basaŋ"), ('old_worn_house', 'alor1247-besar', "uma 'basaŋ")}
 Alorese shares the following forms with other AN languanges, but not Lamaholot:
 {('old_worn_house', 'tetu1245-vique', "uma 'tuan"), ('old_worn_house', 'tetu1245-suai', "uma 'tuan"), ('old_worn_house', 'keda1252-leuwa', 'tua'), ('old_worn_house', 'p-mala1545-acd', '*dadan'), ('old_worn_house', 'blag1240-bama', 'tunaŋge'), ('old_worn_house', 'keda1252-leuba', "tu'an"), ('old_worn_house', 'idat1237', "matu'ʔan"), ('old_worn_house', 'alor1247-pandai', 'tua'), ('old_worn_house', 'blag1240-tuntu', 'tunaŋ'), ('old_worn_house', 'tetu1246', "tu'ɑn")}
 One or more Lamaholot dialects innovated forms not found in Alorese:
 {('old_worn_house', 'lama1277-lewoi', 'laŋo ˈokiŋ')}
 {('old_worn_house', 'lama1277-adona', 'tɜˈnuʔe')}

  Just for clarity:
  There are also the following similarity classes shared between Alorese and LH.
  {('old_worn_house', 'alor1247-baran', 'nɔlɔ'), ('old_worn_house', 'lama1277-kalik', 'noˈlʊŋ'), ('old_worn_house', 'alor1247-pandai', "uma 'nɔlo"), ('old_worn_house', 'lama1277-lerek', 'una moˈlun')}


Concept ‘wife’ is different between Alorese and Lamaholot.

 One or more Lamaholot dialects innovated forms not found in Alorese:
 {('wife', 'lama1277-kiwan', 'hak (kawae)'), ('wife', 'tuku1254', 'he:'), ('wife', 'lama1277-belan', 'ha'), ('wife', 'lama1277-lamah', 'hakã'), ('wife', 'kema1243', 'hɛr'), ('wife', 'laka1255', 'hɛ:n'), ('wife', 'lama1277-adona', 'hãː'), ('wife', 'lama1277-ileap', 'hān'), ('wife', 'lama1277-watan', 'hã')}
 {('wife', 'lama1277-lewop', 'gərapu'), ('wife', 'lama1277-minga', 'gərapu'), ('wife', 'lama1277-lewot', 'gerapu')}
 {('wife', 'blag1240-tuntu', 'piparas'), ('wife', 'blag1240-bama', 'tepras'), ('wife', 'blag1240-kulij', 'epra'), ('wife', 'blag1240-nule', "pi'pra"), ('wife', 'lama1277-lewoe', 'bare'), ('wife', 'baka1276', 'epraha')}
 {('wife', 'p-mala1545-acd', '*bahi'), ('wife', 'lama1277-kalik', 'aˈvan biˈne'), ('wife', 'buna1278-malia', 'phanɑ'), ('wife', 'lama1277-lewok', 'awaga bine'), ('wife', 'buna1278-suai', 'giɛ phana'), ('wife', 'buna1278-bobon', 'giɛ phana')}
 {('wife', 'lama1277-paina', 'ronen')}
 {('wife', 'tetu1245-suai', 'fɛn'), ('wife', 'koto1251', 'fe:'), ('wife', 'tetu1246', 'fɛn'), ('wife', 'sika1262-tanai', 'βai'), ('wife', 'lama1277-pukau', 'vahe'), ('wife', 'lama1277-lewuk', 'awan'), ('wife', 'tetu1245-vique', 'fɛn')}
 {('wife', 'lama1277-lamat', 'hawã'), ('wife', 'p-cent2245-abvd', '*qasawa'), ('wife', 'p-mala1545-abvd', '*qasawa'), ('wife', 'lama1277-kalik', 'awan')}

  Just for clarity:
  There are also the following similarity classes shared between Alorese and LH.
  {('wife', 'lama1277-kiwan', 'hak (kawae)'), ('wife', 'alor1247-munas', "k'wae"), ('wife', 'lama1277-lamak', 'bərkəwahe'), ('wife', 'alor1247-pandai', "k'wae"), ('wife', 'alor1247-besar', "ka'fae"), ('wife', 'kafo1240', 'kofɑ'), ('wife', 'lama1277-botun', 'kəwae'), ('wife', 'alor1247-baran', "ka'fae"), ('wife', 'lama1277-lerek', 'kəweinan'), ('wife', 'lama1277-imulo', 'kəfae'), ('wife', 'lama1277-lewog', 'kəwae'), ('wife', 'lama1277-lamal', 'kəfae'), ('wife', 'lama1277-horow', 'kəwahe'), ('wife', 'lama1277-wuake', 'kəwae'), ('wife', 'alor1247-munas', "k'wae 'nara"), ('wife', 'lama1277-mulan', 'kəwae'), ('wife', 'lama1277-lewoi', 'kəˈwae'), ('wife', 'lama1277-lewob', 'kəwae'), ('wife', 'lama1277-bama', 'kəwae'), ('wife', 'lama1277-lewom', 'kəwae'), ('wife', 'lama1277-dulhi', 'kəwae'), ('wife', 'lama1277-merde', 'kəwae'), ('wife', 'lama1277-waiba', 'kəwae'), ('wife', 'lama1277-ritae', 'kəwãe'), ('wife', 'lama1277-baipi', 'kəwae'), ('wife', 'lama1277-tanju', 'kəwae'), ('wife', 'lama1277-waiwa', 'kəwae')}


Concept ‘all’ is different between Alorese and Lamaholot.

 Alorese innovated the following forms:
 {('all', 'alor1247-pandai', 'skali'), ('all', 'alor1247-baran', "ʔata sa'kali"), ('all', 'alor1247-munas', "sə'kali"), ('all', 'alor1247-munas', "s'kaliʔ")}
 {('all', 'alor1247-besar', "pu'namuŋ")}
 One or more Lamaholot dialects innovated forms not found in Alorese:
 {('all', 'lama1277-lewoe', 'rowao'), ('all', 'lama1277-lamat', 'rowao')}
 {('all', 'lama1277-kalik', 'dərwaki')}
 {('all', 'lama1277-lewot', 'pua'), ('all', 'lama1277-minga', 'puari'), ('all', 'lama1277-belan', 'pua'), ('all', 'lama1277-lewop', 'pua'), ('all', 'lama1277-imulo', 'pua')}
 {('all', 'lama1277-lewok', 'səmua')}
 {('all', 'lama1277-pukau', 'pətika')}
 {('all', 'lama1277-lamak', 'wahok'), ('all', 'lama1277-waiba', 'wakaŋkae'), ('all', 'lama1277-adona', 'ˈwahã ˈkaeʔ'), ('all', 'lama1277-watan', 'wahaŋkae'), ('all', 'lama1277-lamal', 'fakahae'), ('all', 'lama1277-dulhi', 'wahaŋkae'), ('all', 'lama1277-kiwan', 'wahaŋkae'), ('all', 'lama1277-ileap', 'wəkae'), ('all', 'lama1277-lewoi', 'wau ˈkaʔɛŋ'), ('all', 'lama1277-merde', 'wahakae'), ('all', 'lama1277-waiwa', 'wahaŋkae'), ('all', 'lama1277-botun', 'wahaŋkae'), ('all', 'lama1277-horow', 'wahaŋkae'), ('all', 'lama1277-bama', 'waokae'), ('all', 'lama1277-tanju', 'wahak'), ('all', 'lama1277-lewom', 'wakaŋkae'), ('all', 'lama1277-lewog', 'wəkãe'), ('all', 'lama1277-ritae', 'wokae'), ('all', 'lama1277-lewob', 'waokãe'), ('all', 'lama1277-mulan', 'fəka'), ('all', 'lama1277-baipi', 'wakaŋkae'), ('all', 'lama1277-wuake', 'wakae')}
 {('all', 'lama1277-lamah', 'waol'), ('all', 'lama1277-lerek', 'vaˈɔl'), ('all', 'lama1277-lewuk', 'səwaol'), ('all', 'lama1277-kalik', 'svaˈɔl'), ('all', 'lama1277-paina', 'waol')}


Concept ‘sweet_potato’ is different between Alorese and Lamaholot.

 Alorese innovated the following forms:
 {('sweet_potato', 'alor1247-baran', ",kur:a: 'ʔutaŋ")}
 {('sweet_potato', 'alor1247-munas', "ue'sela"), ('sweet_potato', 'alor1247-pandai', "uwe 'sɛla")}
 One or more Lamaholot dialects innovated forms not found in Alorese:
 {('sweet_potato', 'lama1277-lerek', 'ˈhure ˈdʒ͡avan'), ('sweet_potato', 'lama1277-kalik', 'saˈva'), ('sweet_potato', 'lama1277-lewoi', 'uwe ˈʤawa'), ('sweet_potato', 'lama1277-kalik', 'saˈvan'), ('sweet_potato', 'sika1262-hewa', 'ʔohu ʤaʋɑn')}

  Just for clarity:
  There are also the following similarity classes shared between Alorese and LH.
  {('sweet_potato', 'lama1277-adona', 'kəˈwoit'), ('sweet_potato', 'alor1247-besar', "ka'fɔi")}


Concept ‘lake’ is different between Alorese and Lamaholot.

 Alorese shares the following forms with other AN languanges, but not Lamaholot:
 {('lake', 'rett1240', 'kɔlaŋ ɓa:l'), ('lake', 'blag1240-kulij', 'kolaŋ'), ('lake', 'idat1237', "kɔ'lan"), ('lake', 'alor1247-pandai', 'kɔlaŋ'), ('lake', 'rett1240', 'jel kolaŋ'), ('lake', 'alor1247-munas', "wai 'nɛbɔk"), ('lake', 'alor1247-besar', 'kɔlãŋ beiŋ'), ('lake', 'alor1247-munas', "kɔlaŋ 'nɛbɔk")}
 One or more Lamaholot dialects innovated forms not found in Alorese:
 {('lake', 'keda1252', 'o̤pa'), ('lake', 'lama1277-lewoe', 'opa'), ('lake', 'lama1277-lamat', 'opa')}
 {('lake', 'lama1277-lewob', 'walã'), ('lake', 'lama1277-merde', 'walã')}
 {('lake', 'lama1277-mulan', 'liwo'), ('lake', 'lama1277-lewop', 'lifor'), ('lake', 'lama1277-lamah', 'liwo'), ('lake', 'lama1277-lewot', 'liforu'), ('lake', 'lama1277-bama', 'liwo'), ('lake', 'lama1277-paina', 'liwuŋən'), ('lake', 'lama1277-imulo', 'lifor'), ('lake', 'lama1277-lewuk', 'liwuŋ'), ('lake', 'lama1277-wuake', 'liwo'), ('lake', 'koto1251', 'nefo'), ('lake', 'lama1277-lamak', 'liwo'), ('lake', 'lama1277-waiwa', 'liwo'), ('lake', 'lama1277-kiwan', 'liwo'), ('lake', 'lama1277-botun', 'liwo'), ('lake', 'lama1277-kalik', 'liwu'), ('lake', 'lama1277-ritae', 'liwu'), ('lake', 'lama1277-lamal', 'lifo'), ('lake', 'lama1277-tanju', 'liwo'), ('lake', 'lama1277-kalik', 'ˈvai liˈvuŋ'), ('lake', 'lama1277-adona', 'liwo'), ('lake', 'lama1277-lewom', 'liwo'), ('lake', 'lama1277-lewok', 'liworu'), ('lake', 'idat1237', 'lihu'), ('lake', 'lama1277-horow', 'liwo'), ('lake', 'lama1277-dulhi', 'liwo'), ('lake', 'lama1277-pukau', 'liwo'), ('lake', 'sika1262-tanai', 'liβu-n'), ('lake', 'tuku1254', "ʔɛ: 'lihu"), ('lake', 'lama1277-lewog', 'liwũ'), ('lake', 'lama1277-belan', 'liwo'), ('lake', 'lama1277-waiba', 'liwuk'), ('lake', 'lama1277-watan', 'liwo'), ('lake', 'lama1277-lerek', 'ˈwai liˈvunan')}
 {('lake', 'lama1277-ileap', 'bərebot')}
 {('lake', 'lama1277-baipi', 'suŋe'), ('lake', 'hama1240', 'sipuʔɛ')}

  Just for clarity:
  There are also the following similarity classes shared between Alorese and LH.
  {('lake', 'sika1262-tanai', 'rano'), ('lake', 'lama1277-lewoi', 'danau'), ('lake', 'sika1262-maume', 'naŋa'), ('lake', 'alor1247-baran', 'danɑu'), ('lake', 'keda1252-leuba', "da'nɑu"), ('lake', 'p-mala1545-acd', '*danaw'), ('lake', 'p-aust1307-abvd', '*danaw'), ('lake', 'p-cent2245-abvd', '*danaw'), ('lake', 'dein1238', 'bana:q'), ('lake', 'p-mala1545-abvd', '*danaw'), ('lake', 'p-mala1545-acd', '*banaw')}


Concept ‘to_call_out’ is different between Alorese and Lamaholot.

 Alorese shares the following forms with TAP languages:
 {('to_call_out', 'teiw1235', 'puan'), ('to_call_out', 'teiw1235', 'punan'), ('to_call_out', 'sarr1247-nule', 'po:n'), ('to_call_out', 'sarr1247-adiab', 'pon '), ('to_call_out', 'alor1247-besar', 'boaŋ')}
 {('to_call_out', 'atim1239', 'ir'), ('to_call_out', 'kira1248', 'ir'), ('to_call_out', 'alor1247-munas', "g'par"), ('to_call_out', 'abui1241-fuime', 'ir'), ('to_call_out', 'abui1241-ulaga', 'ir'), ('to_call_out', 'alor1247-pandai', "gə'par")}
 {('to_call_out', 'alor1247-pandai', 'ɔrɔŋ'), ('to_call_out', 'pura1258', 'moriŋ'), ('to_call_out', 'rett1240', 'mo:riŋ'), ('to_call_out', 'blag1240-warsa', 'gə-mʊriŋ')}
 One or more Lamaholot dialects innovated forms not found in Alorese:
 {('to_call_out', 'lama1277-lewoi', 'ˈwoeʔ')}
 {('to_call_out', 'lama1277-lewoi', 'ɡəˈmokɑŋ')}
 {('to_call_out', 'lama1277-kalik', 'iˈrak'), ('to_call_out', 'lama1277-lerek', 'ˈirak')}

  Just for clarity:
  There are also the following similarity classes shared between Alorese and LH.
  {('to_call_out', 'kama1365', 'kai'), ('to_call_out', 'alor1247-besar', 'gɛkɛ'), ('to_call_out', 'abui1241-takal', 'kä.qäi'), ('to_call_out', 'lama1277-adona', 'ɡiˈake')}


Concept ‘fog’ is different between Alorese and Lamaholot.

 Alorese innovated the following forms:
 {('fog', 'alor1247-pandai', "ɛkaŋ 'lu")}
 {('fog', 'adan1251-lawah', 'lowo taweʔɛŋ'), ('fog', 'alor1247-besar', "ta'fɛuŋ"), ('fog', 'hama1240', 'tafiʔiŋ'), ('fog', 'adan1251-otvai', 'tafi’ʔiŋ')}
 Alorese shares the following forms with TAP languages:
 {('fog', 'alor1247-pandai', "ɛkaŋ 'lu")}
 {('fog', 'adan1251-lawah', 'lowo taweʔɛŋ'), ('fog', 'alor1247-besar', "ta'fɛuŋ"), ('fog', 'hama1240', 'tafiʔiŋ'), ('fog', 'adan1251-otvai', 'tafi’ʔiŋ')}
 Alorese shares the following forms with other AN languanges, but not Lamaholot:
 {('fog', 'sika1262-tanai', 'səbu sak'), ('fog', 'sika1262-tanai', 'səbur'), ('fog', 'alor1247-munas', "sə'pu"), ('fog', 'kama1365', 'safuː')}
 One or more Lamaholot dialects innovated forms not found in Alorese:
 {('fog', 'lama1277-lerek', 'ˈkəkan ˈkova')}
 {('fog', 'sika1262-tanai', 'koβa'), ('fog', 'lama1277-lewoi', 'kɔwa lɔdɔʔ'), ('fog', 'lama1277-adona', 'kowa koː(k)'), ('rainbow', 'sika1262-hewa', "labi 'ʤaʋɑn")}
 {('fog', 'lama1277-kalik', 'koˈvaru bənəˈbət')}


Concept ‘young_people’ is different between Alorese and Lamaholot.

 Alorese innovated the following forms:
 {('young_people', 'alor1247-munas', "mə'sia 'muda")}
 {('young_people', 'alor1247-pandai', "bik'wae"), ('young_people', 'alor1247-baran', 'kafaɛ'), ('young_people', 'alor1247-besar', "bei'kafae"), ('young_people', 'alor1247-munas', "bik'wae")}
 Alorese shares the following forms with other AN languanges, but not Lamaholot:
 {('young_people', 'alor1247-munas', "bi'lamɛŋ"), ('old_people', 'sika1262-hewa', 'blupur'), ('young_people', 'alor1247-pandai', "bi'lamiŋ"), ('old_people', 'sika1262-maume', 'blupur'), ('old_people', 'alor1247-besar', "bua 'bapaŋ"), ('young_people', 'alor1247-baran', "bɛ 'lamɛŋ"), ('young_people', 'alor1247-besar', "bei'lamɛ̃")}
 One or more Lamaholot dialects innovated forms not found in Alorese:
 {('young_people', 'lama1277-kalik', 'ɡraˈnak')}
 {('young_people', 'lama1277-lerek', 'ɡəˈrəp blaˈmun'), ('young_people', 'lama1277-kalik', 'ɡəˈrəp')}
 {('young_people', 'lama1277-lewoi', 'məˈnureŋ'), ('young_people', 'lama1277-adona', 'muˈrine')}


Concept ‘below’ is different between Alorese and Lamaholot.

 One or more Lamaholot dialects innovated forms not found in Alorese:
 {('below', 'lama1277-lerek', 'rəˈdʒ͡u')}
 {('below', 'lama1277-adona', 'leˈrerˈʔẽ'), ('below', 'lama1277-kalik', 'ˈlere')}

  Just for clarity:
  There are also the following similarity classes shared between Alorese and LH.
  {('below', 'alor1247-pandai', 'lauŋ'), ('below', 'sika1262-hewa', 'ləʋun'), ('below', 'pura1258', "a'lolaŋ"), ('below', 'alor1247-besar', "ɔrɔ 'laŋ"), ('below', 'rett1240', 'galɔlaŋ'), ('below', 'alor1247-baran', 'la:uŋ'), ('below', 'alor1247-besar', "ali 'laŋ"), ('below', 'alor1247-munas', 'lauŋ'), ('below', 'lama1277-adona', 'lali laŋũ'), ('below', 'lama1277-lewoi', 'ˈlali')}


Concept ‘hole’ is different between Alorese and Lamaholot.

 Alorese innovated the following forms:
 {('hole', 'alor1247-pandai', 'waŋɡɔ'), ('hole', 'alor1247-besar', 'faŋɡɔ'), ('hole', 'alor1247-munas', 'waŋɔʔ')}
 Alorese shares the following forms with other AN languanges, but not Lamaholot:
 {('hole', 'keda1252-leuba', 'uɔʔ'), ('hole', 'alor1247-munas', 'nuɔʔ')}
 One or more Lamaholot dialects innovated forms not found in Alorese:
 {('hole', 'lama1277-lerek', 'ˈroboŋ')}
 {('hole', 'lama1277-kalik', 'uˈma'), ('hole', 'lama1277-kalik', 'uˈmaŋ'), ('hole', 'lama1277-lewoi', 'ˈumaŋ')}
 {('hole', 'lama1277-adona', 'rɔɑ̃')}
 {('hole', 'lama1277-lerek', 'ˈvuok'), ('hole', 'tetu1245-vique', 'kuak'), ('hole', 'tetu1245-suai', 'kuak')}


Concept ‘forest’ is different between Alorese and Lamaholot.

 Alorese innovated the following forms:
 {('forest', 'alor1247-munas', 'dɛhɛk'), ('forest', 'alor1247-pandai', "də'hɛk")}
 One or more Lamaholot dialects innovated forms not found in Alorese:
 {('forest', 'sarr1247-nule', 'imra:g'), ('forest', 'lama1277-imulo', 'ekamaŋan'), ('forest', 'sarr1247-adiab', "mə'rag"), ('forest', 'kama1365', 'atakai'), ('forest', 'lama1277-kalik', 'maŋak'), ('forest', 'lama1277-lewop', 'ekamaŋan'), ('forest', 'dein1238', 'mata:g'), ('forest', 'lama1277-lewuk', 'maŋak'), ('forest', 'lama1277-lewot', 'eka maŋan'), ('forest', 'lama1277-lewok', 'maŋak'), ('forest', 'lama1277-belan', 'eka maŋan'), ('forest', 'lama1277-kalik', 'maˈŋak')}
 {('forest', 'lama1277-bama', 'woʔonã'), ('forest', 'lama1277-lamah', 'bənewa'), ('forest', 'lama1277-lewom', 'newa onã'), ('forest', 'lama1277-baipi', 'newa onã'), ('forest', 'lama1277-merde', 'bənewa'), ('forest', 'lama1277-lewog', 'nəwoʔona')}
 {('forest', 'lama1277-lewob', 'kərawo')}
 {('forest', 'lama1277-lamat', 'musʔã')}
 {('forest', 'lama1277-lerek', 'ˈknovot'), ('forest', 'kuii1253', 'awotan'), ('forest', 'lama1277-paina', 'kowoten')}
 {('forest', 'lama1277-mulan', 'məgə'), ('forest', 'lama1277-ileap', 'məgən'), ('forest', 'lama1277-wuake', 'əka məge'), ('forest', 'kaer1234', "qa'qui"), ('forest', 'lama1277-horow', 'məgã')}
 {('forest', 'lama1277-adona', 'ruːkʔãː'), ('forest', 'lama1277-dulhi', 'rukʔã'), ('forest', 'lama1277-watan', 'rukʔã'), ('forest', 'lama1277-waiwa', 'rukã'), ('forest', 'lama1277-pukau', 'ruka onã'), ('forest', 'lama1277-botun', 'rukʔã'), ('forest', 'lama1277-waiba', 'kajo onã')}
 {('forest', 'lama1277-lewoe', 'əso ʔuho')}

  Just for clarity:
  There are also the following similarity classes shared between Alorese and LH.
  {('forest', 'alor1247-baran', "u'tɑŋ"), ('forest', 'atim1239', 'atiŋ'), ('forest', 'keda1252', 'tuen'), ('forest', 'lama1277-kiwan', 'dua'), ('forest', 'keda1252-leuwa', 'u̘taŋ'), ('forest', 'lama1277-lewoi', 'ˈhutaŋ'), ('forest', 'alor1247-munas', 'duaŋ'), ('forest', 'alor1247-pandai', 'utaŋ'), ('forest', 'sika1262-maume', 't̪uʔan'), ('forest', 'p-mala1545-acd', '*tuqan'), ('forest', 'keda1252-leuba', "tu'wɛn"), ('forest', 'lama1277-lamak', 'utan'), ('forest', 'lama1277-kalik', 'ˈduan'), ('forest', 'p-mala1545-acd', '*talun'), ('forest', 'lama1277-tanju', 'duã'), ('forest', 'sika1262-hewa', 'tuʔan')}
  {('forest', 'lama1277-ritae', 'pənɤ'), ('forest', 'adan1251-lawah', 'bana'), ('forest', 'adan1251-otvai', '’bana'), ('forest', 'pura1258', "ba'naha"), ('forest', 'rett1240', 'bana:'), ('forest', 'lama1277-lamal', 'eka pənət'), ('forest', 'kula1280-lanto', 'banan'), ('forest', 'rett1240', "va'na"), ('forest', 'kula1280-lanto', '’banaŋ'), ('forest', 'alor1247-besar', "ba'nːa"), ('forest', 'lama1277-minga', 'ekapənət')}


Concept ‘earthworm’ is different between Alorese and Lamaholot.

 One or more Lamaholot dialects innovated forms not found in Alorese:
 {('earthworm', 'lama1277-lewop', 'ekoj'), ('earthworm', 'lama1277-merde', 'kodəl'), ('earthworm', 'lama1277-waiba', 'gego'), ('earthworm', 'lama1277-mulan', 'kodɤ'), ('earthworm', 'lama1277-pukau', 'mekã'), ('earthworm', 'lama1277-belan', 'eko'), ('earthworm', 'lama1277-lamah', 'kodəl'), ('earthworm', 'lama1277-paina', 'egoy'), ('earthworm', 'lama1277-kalik', 'ekoj')}
 {('earthworm', 'lama1277-lerek', 'helvaˈlak'), ('earthworm', 'lama1277-kalik', 'səlvaˈlak')}
 {('earthworm', 'lama1277-watan', 'kəbeŋər'), ('earthworm', 'lama1277-waiwa', 'kəbeŋe'), ('earthworm', 'lama1277-lewob', 'kəbeŋə̃'), ('earthworm', 'lama1277-ritae', 'kəbeŋã'), ('earthworm', 'lama1277-botun', 'kəbeŋə'), ('earthworm', 'sika1262-hewa', 'taibener'), ('earthworm', 'lama1277-baipi', 'kəbeŋə'), ('earthworm', 'lama1277-lewom', 'kəbeŋer'), ('earthworm', 'lama1277-dulhi', 'kəbeŋə'), ('earthworm', 'lama1277-kiwan', 'kəbeŋã'), ('earthworm', 'lama1277-bama', 'kəbeŋə'), ('earthworm', 'lama1277-lewog', 'kəbeŋə̃'), ('earthworm', 'lama1277-tanju', 'kəbeŋə'), ('earthworm', 'lama1277-horow', 'kəbeŋə'), ('earthworm', 'lama1277-lamak', 'kəbeŋer')}
 {('earthworm', 'lama1277-ileap', 'keləwalo'), ('earthworm', 'lama1277-wuake', 'salawalak'), ('earthworm', 'lama1277-lewok', 'səwalaku'), ('earthworm', 'lama1277-lewoe', 'səwalo'), ('earthworm', 'lama1277-lamat', 'səwalo'), ('earthworm', 'lama1277-lewuk', 'salwaja'), ('earthworm', 'lama1277-minga', 'salafalak'), ('earthworm', 'lama1277-lewot', 'salafalaku'), ('earthworm', 'lama1277-lamal', 'salafalã'), ('earthworm', 'lama1277-imulo', 'salafalak')}

  Just for clarity:
  There are also the following similarity classes shared between Alorese and LH.
  {('earthworm', 'alor1247-munas', "ula 'wali"), ('earthworm', 'lama1277-adona', 'kalaˈwala'), ('earthworm', 'alor1247-besar', "fali-'fali"), ('earthworm', 'lama1277-lewoi', 'ulaˈwala'), ('earthworm', 'kira1248', 'biliwali'), ('earthworm', 'alor1247-pandai', "ula 'wali"), ('earthworm', 'alor1247-baran', ",ʔula 'fale")}


Concept ‘short’ is different between Alorese and Lamaholot.

 Alorese shares the following forms with other AN languanges, but not Lamaholot:
 {('short', 'alor1247-pandai', 'wɛro'), ('short', 'alor1247-munas', 'wɛrɔʔ'), ('short', 'alor1247-besar', 'fero'), ('short', 'keda1252-leuba', "wɛ'hɛʔ")}
 One or more Lamaholot dialects innovated forms not found in Alorese:
 {('short', 'lama1277-lewoe', 'boʔõ'), ('short', 'abui1241-fuime', 'bui'), ('short', 'sika1262-hewa', 'buluk'), ('short', 'abui1241-takal', 'bui'), ('short', 'abui1241-ulaga', 'bui'), ('short', 'sika1262-hewa', 'puʔur'), ('short', 'atim1239', 'bui'), ('short', 'sika1262-hewa', 'buʔu')}
 {('short', 'lama1277-lamat', 'waʔã')}
 {('short', 'lama1277-lewoi', 'ˈlɛrɛʔ'), ('short', 'lama1277-kalik', '(aˈdik) leˈrɛk'), ('short', 'lama1277-lerek', 'ataˈdikə lereˈkən')}
 {('short', 'lama1277-lewoi', 'ˈkənu'), ('short', 'lama1277-adona', 'kɜʔˈrũ')}
 {('short', 'lama1277-pukau', 'lerʔẽ'), ('short', 'lama1277-bama', 'lere')}
 {('short', 'lama1277-mulan', 'kənəpo')}
 {('short', 'lama1277-lewom', 'kəru'), ('short', 'lama1277-baipi', 'kərũ'), ('short', 'lama1277-ritae', 'kəru'), ('short', 'lama1277-tanju', 'ətu'), ('short', 'lama1277-belan', 'kəru'), ('short', 'lama1277-wuake', 'kəsɁu'), ('short', 'lama1277-lamah', 'kərʔu'), ('short', 'lama1277-dulhi', 'kəsũ'), ('short', 'lama1277-lewog', 'kəsu'), ('short', 'lama1277-waiba', 'karu'), ('short', 'lama1277-botun', 'kərʔũ'), ('short', 'lama1277-waiwa', 'kəru'), ('short', 'lama1277-watan', 'kəsʔũ'), ('short', 'lama1277-lamak', 'kəsũ'), ('short', 'lama1277-ileap', 'kərukən'), ('short', 'lama1277-horow', 'kərʔũ'), ('short', 'lama1277-kiwan', 'kərʔũ'), ('short', 'lama1277-merde', 'kəsʔu'), ('short', 'lama1277-lamal', 'kəru'), ('short', 'lama1277-lewob', 'pəsu')}
 {('short', 'buna1278-suai', 'barak'), ('short', 'p-mala1545-acd', '*babaq'), ('short', 'laka1255', 'badɑk'), ('short', 'buna1278-malia', 'barɑk'), ('short', 'buna1278-bobon', 'barak'), ('short', 'tetu1246', "ba'dak"), ('short', 'p-cent2245-abvd', '*babaq'), ('short', 'tetu1245-vique', '(ai) badak'), ('short', 'mamb1306', 'bada'), ('short', 'sika1262-maume', 'buluk'), ('short', 'tetu1245-suai', '(ai) badak'), ('short', 'idat1237', "pa'dak"), ('short', 'sika1262-tanai', 'buluk'), ('short', 'lama1277-lerek', 'paˈdʒ͡ok'), ('short', 'tuku1254', "bɔ'lɔ:kɔ"), ('short', 'koto1251', 'paraʔ'), ('short', 'p-mala1545-abvd', '*ma-babaq')}
 {('short', 'lama1277-adona', 'rɜʔˈrẽ')}
 {('short', 'lama1277-minga', 'pəjok'), ('short', 'lama1277-paina', 'pəjokən'), ('short', 'lama1277-lewot', 'pəjokan'), ('short', 'lama1277-lewop', 'pəjokən'), ('short', 'lama1277-imulo', 'pəjokən')}

  Just for clarity:
  There are also the following similarity classes shared between Alorese and LH.
  {('short', 'keda1252-leuba', "mɛ'kuʔ"), ('short', 'alor1247-baran', 'mak:u'), ('short', 'alor1247-besar', "('kaʤo) 'maku"), ('short', 'alor1247-munas', "mə'ku"), ('short', 'lama1277-lerek', 'məˈkul')}
  {('short', 'lama1277-kalik', 'məgok'), ('short', 'alor1247-besar', 'maku'), ('short', 'lama1277-lewuk', 'məgok'), ('short', 'lama1277-kalik', 'məˈɡɔk'), ('short', 'keda1252', 'mekuʔ'), ('short', 'lama1277-lewok', 'məgok'), ('short', 'alor1247-munas', "mə'ku"), ('short', 'alor1247-pandai', "ma'kːu")}


Concept ‘traditional_house’ is different between Alorese and Lamaholot.

 Alorese innovated the following forms:
 {('traditional_house', 'alor1247-besar', "uma 'hada"), ('traditional_house', 'alor1247-munas', "uma 'adat"), ('traditional_house', 'alor1247-pandai', "uma 'adat")}
 {('traditional_house', 'alor1247-munas', "uma 'lɛlaŋ")}
 {('traditional_house', 'rett1240', 'kokor'), ('traditional_house', 'alor1247-pandai', 'kɔkɔr')}
 Alorese shares the following forms with TAP languages:
 {('traditional_house', 'alor1247-besar', "uma 'hada"), ('traditional_house', 'alor1247-munas', "uma 'adat"), ('traditional_house', 'alor1247-pandai', "uma 'adat")}
 {('traditional_house', 'alor1247-munas', "uma 'lɛlaŋ")}
 {('traditional_house', 'rett1240', 'kokor'), ('traditional_house', 'alor1247-pandai', 'kɔkɔr')}
 One or more Lamaholot dialects innovated forms not found in Alorese:
 {('traditional_house', 'lama1277-kalik', 'ˈuna keˈdak')}
 {('traditional_house', 'lama1277-adona', 'laŋɔ ˈbɛʔlẽ'), ('traditional_house', 'lama1277-lewoi', 'ˈlaŋɔ ˈbeləŋ')}
 {('traditional_house', 'lama1277-lerek', 'ˈuna ˈrajan')}
 {('traditional_house', 'lama1277-lewoi', 'ˈkɔrkɛ')}


Concept ‘angry’ is different between Alorese and Lamaholot.

 Alorese innovated the following forms:
 {('angry', 'alor1247-pandai', "k'badʒa")}
 {('angry', 'alor1247-besar', "ka'laki"), ('angry', 'alor1247-munas', "ɔnɔ k'likil"), ('angry', 'rett1240', 'numi kalila'), ('angry', 'alor1247-munas', "ɔnɔŋ k'likil"), ('angry', 'blag1240-kulij', 'lil'), ('angry', 'alor1247-pandai', "k'likil"), ('angry', 'pura1258', "a'lil"), ('angry', 'rett1240', 'go:mi kalila'), ('angry', 'kaer1234', "ke'likil"), ('angry', 'blag1240-warsa', '(om mi) kilikil'), ('angry', 'baka1276', 'om ilil'), ('angry', 'blag1240-nule', 'tomilil')}
 Alorese shares the following forms with TAP languages:
 {('angry', 'alor1247-pandai', "k'badʒa")}
 {('angry', 'alor1247-besar', "ka'laki"), ('angry', 'alor1247-munas', "ɔnɔ k'likil"), ('angry', 'rett1240', 'numi kalila'), ('angry', 'alor1247-munas', "ɔnɔŋ k'likil"), ('angry', 'blag1240-kulij', 'lil'), ('angry', 'alor1247-pandai', "k'likil"), ('angry', 'pura1258', "a'lil"), ('angry', 'rett1240', 'go:mi kalila'), ('angry', 'kaer1234', "ke'likil"), ('angry', 'blag1240-warsa', '(om mi) kilikil'), ('angry', 'baka1276', 'om ilil'), ('angry', 'blag1240-nule', 'tomilil')}
 One or more Lamaholot dialects innovated forms not found in Alorese:
 {('angry', 'lama1277-kalik', 'ɡrɛˈdi')}
 {('angry', 'lama1277-lewoi', 'məˈŋɛʔ')}
 {('angry', 'lama1277-lerek', 'ˈunor')}
 {('angry', 'lama1277-adona', 'ˈbɜkɜ')}


Concept ‘to_fight’ is different between Alorese and Lamaholot.

 Alorese innovated the following forms:
 {('to_fight', 'alor1247-baran', "ka'laĩ 'fak:ĩ"), ('to_fight', 'alor1247-munas', "boa wə'ki"), ('to_fight', 'alor1247-pandai', "boa wə'kːi"), ('to_fight', 'alor1247-pandai', "k'laiŋ wə'kːi"), ('to_fight', 'alor1247-pandai', "ula wə'kːi")}
 One or more Lamaholot dialects innovated forms not found in Alorese:
 {('to_fight', 'lama1277-lamah', 'uno'), ('to_fight', 'lama1277-minga', 'puno'), ('to_fight', 'lama1277-lewuk', 'punu'), ('to_fight', 'lama1277-lewok', 'puno'), ('to_fight', 'lama1277-lamak', 'pəwuno'), ('to_fight', 'lama1277-lamal', 'uno'), ('to_fight', 'lama1277-lewob', 'kəwuno'), ('to_fight', 'lama1277-paina', 'punu'), ('to_fight', 'lama1277-wuake', 'unho'), ('to_fight', 'sika1262-tanai', 'punu βiʔi-ŋ'), ('to_fight', 'lama1277-lerek', 'ˈpunu vakˈhi'), ('to_fight', 'lama1277-dulhi', 'pəwuno'), ('to_fight', 'lama1277-kalik', 'ˈpunu'), ('to_fight', 'lama1277-belan', 'uno'), ('to_fight', 'lama1277-lewop', 'pun'), ('to_fight', 'lama1277-watan', 'pəwuno'), ('to_fight', 'lama1277-lewot', 'punoj'), ('to_fight', 'keda1252-leuwa', 'avε u̘nu'), ('to_fight', 'lama1277-horow', 'pəwuno'), ('to_fight', 'lama1277-waiwa', 'pəwuno'), ('to_fight', 'lama1277-mulan', 'pəwuno'), ('to_fight', 'lama1277-botun', 'pəwuno'), ('to_fight', 'lama1277-imulo', 'puno'), ('to_fight', 'lama1277-adona', 'pɜˈwuno'), ('to_fight', 'lama1277-merde', 'uno (geni)'), ('to_fight', 'lama1277-kiwan', 'pəwuno'), ('to_fight', 'lama1277-ileap', 'uno'), ('to_fight', 'lama1277-kalik', 'puno'), ('to_fight', 'sika1262-hewa', 'punu ʋiʔin')}
 {('to_fight', 'lama1277-lewoe', 'ladu(woʔi)')}

  Just for clarity:
  There are also the following similarity classes shared between Alorese and LH.
  {('to_fight', 'lama1277-lewog', 'gəni'), ('to_fight', 'lama1277-tanju', 'gəni'), ('to_fight', 'lama1277-lewom', 'gəni(wəki)'), ('to_fight', 'lama1277-lamat', 'gəni (waʔi)'), ('to_fight', 'lama1277-merde', 'uno (geni)'), ('to_fight', 'lama1277-waiba', 'gəni'), ('to_fight', 'lama1277-ritae', 'gəni'), ('to_fight', 'lama1277-baipi', 'gəni(wəki)'), ('to_fight', 'alor1247-besar', "bɛhɛ 'fakiŋ"), ('to_fight', 'lama1277-bama', 'gəni'), ('to_fight', 'lama1277-pukau', 'konik'), ('to_fight', 'lama1277-lewoi', 'ˈɡəniŋ')}


Concept ‘many’ is different between Alorese and Lamaholot.

 Alorese innovated the following forms:
 {('many', 'alor1247-baran', "ʔata la'bi:"), ('many', 'alor1247-pandai', 'labːiŋ'), ('many', 'alor1247-pandai', 'labːi')}
 {('many', 'alor1247-besar', 'mafa')}
 One or more Lamaholot dialects innovated forms not found in Alorese:
 {('many', 'lama1277-adona', 'ˈʔaːjʔa')}
 {('many', 'lama1277-baipi', 'aja'), ('many', 'lama1277-watan', 'ajaʔ'), ('many', 'lama1277-waiba', 'ajã'), ('many', 'lama1277-lamal', 'ajã'), ('many', 'lama1277-wuake', 'ajã'), ('many', 'lama1277-lewob', 'ajã'), ('many', 'lama1277-mulan', 'aja'), ('many', 'lama1277-horow', 'ayʔã'), ('many', 'lama1277-botun', 'ayʔã'), ('many', 'lama1277-lamak', 'ajã'), ('many', 'lama1277-lewog', 'ayaʔ'), ('many', 'lama1277-pukau', 'ayaʔ'), ('many', 'lama1277-kiwan', 'ayã'), ('many', 'lama1277-merde', 'ayã'), ('many', 'lama1277-ritae', 'aya'), ('many', 'lama1277-bama', 'aya'), ('many', 'lama1277-lewoi', 'ˈajaʔ'), ('many', 'lama1277-lamah', 'ayʔã'), ('many', 'lama1277-belan', 'aja'), ('many', 'lama1277-waiwa', 'ayaʔ'), ('many', 'lama1277-tanju', 'ajã'), ('many', 'lama1277-ileap', 'aja')}
 {('many', 'lama1277-lamat', 'wəda')}
 {('many', 'lama1277-lewuk', 'rai'), ('many', 'tetu1246', 'barɑk'), ('many', 'lama1277-lewot', 'rai'), ('many', 'lama1277-lewop', 'rai'), ('many', 'lama1277-imulo', 'raiŋa'), ('many', 'lama1277-minga', 'rai'), ('many', 'keda1252-leuba', 'rai'), ('many', 'lama1277-lewok', 'rai'), ('many', 'lama1277-lerek', 'ˈrɔi'), ('many', 'lama1277-kalik', 'raik'), ('many', 'keda1252', 'rai'), ('many', 'lama1277-kalik', 'ˈraik')}
 {('many', 'buna1278-malia', "bɑ'ʔɪs"), ('many', 'buna1278-bobon', 'baɁis'), ('many', 'lama1277-lewoe', 'boʔu'), ('many', 'buna1278-suai', 'baɁis')}
 {('many', 'lama1277-paina', 'waikəy')}
 {('many', 'lama1277-lewom', 'dəne')}

  Just for clarity:
  There are also the following similarity classes shared between Alorese and LH.
  {('many', 'alor1247-pandai', 'rasa'), ('many', 'lama1277-dulhi', 'arʔã'), ('many', 'alor1247-munas', 'rasa')}


Concept ‘back’ is different between Alorese and Lamaholot.

 Alorese innovated the following forms:
 {('back', 'alor1247-baran', 'ʔalɛŋ')}
 One or more Lamaholot dialects innovated forms not found in Alorese:
 {('back', 'kelo1247-bring', 'gɛhɑk'), ('back', 'lama1277-belan', 'usuk'), ('back', 'lama1277-lewog', 'uhuk'), ('back', 'lama1277-bama', 'uhuk'), ('back', 'lama1277-merde', 'uhukət'), ('back', 'lama1277-dulhi', 'uhuk'), ('back', 'lama1277-ileap', 'uhuk'), ('back', 'lama1277-paina', 'wuhuk'), ('back', 'keda1252-leuwa', 'u̘hur'), ('back', 'lama1277-lerek', 'wuˈhuk'), ('back', 'lama1277-ritae', 'uhuk'), ('back', 'lama1277-waiwa', 'uhuk'), ('back', 'lama1277-wuake', 'uhuk'), ('back', 'lama1277-mulan', 'uhuk')}
 {('back', 'lama1277-lewuk', 'wurəsa')}
 {('back', 'lama1277-adona', 'ˈwohʔoˈkɜt'), ('back', 'lama1277-lewoe', 'woho'), ('back', 'lama1277-kiwan', 'woho'), ('back', 'lama1277-lamak', 'woho'), ('back', 'mamb1306', 'hɔhɔ'), ('back', 'lama1277-watan', 'wohõ')}
 {('back', 'lama1277-botun', 'kolak'), ('back', 'lama1277-tanju', 'kolaʔ'), ('back', 'lama1277-lewoi', 'ˈkɔlaŋ'), ('back', 'lama1277-lewom', 'kolaʔ'), ('back', 'lama1277-waiba', 'kolaʔ')}
 {('back', 'p-mala1545-acd', '*burit'), ('back', 'lama1277-baipi', 'hurut'), ('back', 'p-mala1545-acd', '*buRit')}
 {('back', 'lama1277-lewob', 'keko')}
 {('back', 'keda1252-leuwa', 'ɔbi'), ('back', 'lama1277-pukau', 'nobe'), ('back', 'keda1252', 'obi'), ('back', 'keda1252-leuba', 'ʔobi')}
 {('back', 'lama1277-horow', 'kənuwənək'), ('back', 'lama1277-kalik', 'kənaˈru'), ('back', 'lama1277-kalik', 'kənarun'), ('back', 'lama1277-lewok', 'kənaruga'), ('back', 'lama1277-lamah', 'kənaruk')}
 {('back', 'lama1277-lewop', 'kətubəŋ'), ('back', 'lama1277-lewot', 'kətubəŋha'), ('back', 'lama1277-lamal', 'kətubəŋ'), ('back', 'lama1277-imulo', 'kətubəŋəs'), ('back', 'lama1277-minga', 'kətubəsa')}

  Just for clarity:
  There are also the following similarity classes shared between Alorese and LH.
  {('back', 'alor1247-munas', 'punuŋ'), ('back', 'lama1277-lamat', 'punuʔu'), ('back', 'alor1247-munas', 'punuʔ'), ('back', 'alor1247-pandai', 'punuŋ'), ('back', 'alor1247-besar', 'punuŋ'), ('back', 'buna1278-malia', 'nun')}


Concept ‘to_tell_untruth’ is different between Alorese and Lamaholot.

 Alorese innovated the following forms:
 {('to_tell_untruth', 'alor1247-pandai', "putɔr'bale")}
 {('to_tell_untruth', 'baka1276', 'leko'), ('to_tell_untruth', 'maka1316', 'lɔgɔ'), ('to_tell_untruth', 'rett1240', 'leko'), ('to_tell_untruth', 'blag1240-nule', 'leko'), ('to_tell_untruth', 'alor1247-munas', 'lɛkɔ'), ('to_tell_untruth', 'rett1240', 'lɛko'), ('to_tell_untruth', 'blag1240-kulij', 'leko')}
 {('to_tell_untruth', 'alor1247-besar', "ki'biri"), ('to_tell_untruth', 'alor1247-munas', 'kibir')}
 Alorese shares the following forms with TAP languages:
 {('to_tell_untruth', 'alor1247-pandai', "putɔr'bale")}
 {('to_tell_untruth', 'baka1276', 'leko'), ('to_tell_untruth', 'maka1316', 'lɔgɔ'), ('to_tell_untruth', 'rett1240', 'leko'), ('to_tell_untruth', 'blag1240-nule', 'leko'), ('to_tell_untruth', 'alor1247-munas', 'lɛkɔ'), ('to_tell_untruth', 'rett1240', 'lɛko'), ('to_tell_untruth', 'blag1240-kulij', 'leko')}
 {('to_tell_untruth', 'alor1247-besar', "ki'biri"), ('to_tell_untruth', 'alor1247-munas', 'kibir')}
 One or more Lamaholot dialects innovated forms not found in Alorese:
 {('to_tell_untruth', 'lama1277-kalik', 'ˈkvait')}
 {('to_tell_untruth', 'lama1277-lerek', 'kəˈropən')}
 {('to_tell_untruth', 'lama1277-lerek', 'kəˈvaɡor')}
 {('to_tell_untruth', 'lama1277-lewoi', 'opɑˈʔɑkɑl')}
 {('to_tell_untruth', 'lama1277-adona', 'ˈʔopɜ')}


Concept ‘to_shoo_to_chase_away_to_send_away’ is different between Alorese and Lamaholot.

 Alorese innovated the following forms:
 {('to_shoo_to_chase_away_to_send_away', 'alor1247-besar', 'sɛ')}
 {('to_shoo_to_chase_away_to_send_away', 'alor1247-besar', 'tutɛ')}
 {('to_shoo_to_chase_away_to_send_away', 'alor1247-munas', 'tutɛ'), ('to_shoo_to_chase_away_to_send_away', 'alor1247-pandai', 'tutɛ')}
 One or more Lamaholot dialects innovated forms not found in Alorese:
 {('to_shoo_to_chase_away_to_send_away', 'lama1277-lerek', 'ˈdəɡur'), ('to_shoo_to_chase_away_to_send_away', 'tetu1245-suai', 'teɁur'), ('to_shoo_to_chase_away_to_send_away', 'tetu1245-vique', 'teɁur')}
 {('to_shoo_to_chase_away_to_send_away', 'teiw1235', 'gətiar'), ('to_shoo_to_chase_away_to_send_away', 'p-alor1249', '*tiara'), ('to_shoo_to_chase_away_to_send_away', 'lamm1241-westp', '-but:ir'), ('to_shoo_to_chase_away_to_send_away', 'lama1277-lewoi', 'ˈtəruŋ'), ('to_shoo_to_chase_away_to_send_away', 'baka1276', 'teri'), ('to_shoo_to_chase_away_to_send_away', 'nede1245', 'getiala'), ('to_shoo_to_chase_away_to_send_away', 'blag1240-tuntu', "ge'teri"), ('to_shoo_to_chase_away_to_send_away', 'rett1240', 'gatiari'), ('to_shoo_to_chase_away_to_send_away', 'blag1240-bama', 'geteriŋ'), ('to_shoo_to_chase_away_to_send_away', 'rett1240', 'gatieri'), ('to_shoo_to_chase_away_to_send_away', 'blag1240-kulij', 'teri'), ('to_shoo_to_chase_away_to_send_away', 'blag1240-warsa', 'geteri')}
 {('to_shoo_to_chase_away_to_send_away', 'lama1277-kalik', 'siˈka'), ('to_shoo_to_chase_away_to_send_away', 'sarr1247-adiab', "si'kah"), ('to_shoo_to_chase_away_to_send_away', 'lama1277-adona', 'ˈsika'), ('to_shoo_to_chase_away_to_send_away', 'blag1240-nule', 'sika')}


Concept ‘digging_stick’ is different between Alorese and Lamaholot.

 Alorese innovated the following forms:
 {('digging_stick', 'alor1247-munas', 'biŋku'), ('digging_stick', 'alor1247-besar', 'biŋku')}
 {('digging_stick', 'alor1247-pandai', "ka'hawulu")}
 One or more Lamaholot dialects innovated forms not found in Alorese:
 {('digging_stick', 'lama1277-lewoi', 'ˈnuaŋ')}
 {('digging_stick', 'lama1277-lewoi', 'ˈnəpa'), ('digging_stick', 'lama1277-adona', "nu'bɑk")}
 {('digging_stick', 'lama1277-kalik', 'snɔˈkar'), ('digging_stick', 'lama1277-lerek', 'hənoˈkar')}


Concept ‘to_turn’ is different between Alorese and Lamaholot.

 Alorese innovated the following forms:
 {('to_turn', 'alor1247-besar', 'heŋko')}
 {('to_turn', 'alor1247-munas', 'bɛhɔʔ')}
 {('to_turn', 'maka1316', "ka'ili"), ('to_turn', 'alor1247-pandai', 'gɔlɔ'), ('to_turn', 'fata1247', 'kailɛ')}
 Alorese shares the following forms with TAP languages:
 {('to_turn', 'alor1247-besar', 'heŋko')}
 {('to_turn', 'alor1247-munas', 'bɛhɔʔ')}
 {('to_turn', 'maka1316', "ka'ili"), ('to_turn', 'alor1247-pandai', 'gɔlɔ'), ('to_turn', 'fata1247', 'kailɛ')}
 Alorese shares the following forms with other AN languanges, but not Lamaholot:
 {('to_turn', 'kama1365', 'miakiː'), ('to_turn', 'kama1365', '-kiː'), ('to_turn', 'nede1245', 'magija'), ('to_turn', 'kama1365', 'mi- -kiː'), ('to_turn', 'p-aust1307-abvd', '*Niku'), ('to_turn', 'alor1247-pandai', 'mikuŋ')}
 {('to_turn', 'keda1252-leuwa', 'batiŋ'), ('to_turn', 'p-mala1545-acd', '*ileŋ'), ('to_turn', 'sawi1256', "ba'liba"), ('to_turn', 'sika1262-hewa', 'belok'), ('to_turn', 'blag1240-warsa', 'kepelok'), ('to_turn', 'alor1247-baran', 'lakɔŋ'), ('to_turn', 'kula1280-lanto', 'belok'), ('to_turn', 'p-mala1545-acd', '*bali'), ('to_turn', 'p-mala1545-acd', '*biliŋ'), ('to_turn', 'p-mala1545-acd', '*balik'), ('to_turn', 'p-mala1545-acd', '*puliŋ'), ('to_turn', 'kema1243', 'hali'), ('to_turn', 'kaer1234', 'beliŋ'), ('to_turn', 'p-mala1545-abvd', '*biliŋ'), ('to_turn', 'sika1262-maume', 'baloŋ')}
 One or more Lamaholot dialects innovated forms not found in Alorese:
 {('to_turn', 'lama1277-kalik', 'ɛˈɡɔl'), ('to_turn', 'lama1277-adona', 'ˈpɛkɔt')}
 {('to_turn', 'lama1277-lerek', 'peŋoˈhaŋ')}
 {('to_turn', 'lama1277-lewoi', 'ˈtuweʔ')}


Concept ‘guts’ is different between Alorese and Lamaholot.

 Alorese innovated the following forms:
 {('guts', 'alor1247-munas', "taiŋ 'beiŋ")}
 Alorese shares the following forms with other AN languanges, but not Lamaholot:
 {('guts', 'alor1247-pandai', "taiŋ 'karːik"), ('guts', 'p-mala1545-abvd', '*tinaqi'), ('guts', 'alor1247-munas', "taiŋ kə'rik"), ('guts', 'tetu1246', 'ten kik'), ('guts', 'p-aust1307-abvd', '*Cinaqi'), ('guts', 'alor1247-besar', "teiŋ ka'rikiŋ"), ('guts', 'p-cent2245-abvd', '*taqi')}
 One or more Lamaholot dialects innovated forms not found in Alorese:
 {('guts', 'lama1277-kalik', 'tai kaˈluŋ')}
 {('guts', 'lama1277-lerek', 'kəbotin'), ('guts', 'blag1240-tuntu', 'tebetar'), ('guts', 'mamb1306', 'te buti'), ('guts', 'tuku1254', "te: 'bɔte"), ('guts', 'lama1277-adona', 'kɜˈbotĩ'), ('guts', 'sarr1247-nule', 'betar'), ('guts', 'lama1277-kalik', 'kəbɔˈti')}

  Just for clarity:
  There are also the following similarity classes shared between Alorese and LH.
  {('guts', 'lama1277-lewoi', 'ˈtaiŋ ˈonəʔəŋ'), ('guts', 'alor1247-baran', "teiŋ 'ɔnɔŋ")}


Concept ‘to_shine’ is different between Alorese and Lamaholot.

 Alorese innovated the following forms:
 {('to_shine', 'kama1365', 'saːra'), ('to_shine', 'alor1247-besar', 'saru '), ('to_shine', 'alor1247-munas', "sə'ruʔ ")}
 {('to_shine', 'alor1247-munas', 'tutuŋ'), ('to_shine', 'alor1247-pandai', 'tutuŋ')}
 {('to_shine', 'alor1247-munas', 'ɔbaŋ')}
 Alorese shares the following forms with TAP languages:
 {('to_shine', 'kama1365', 'saːra'), ('to_shine', 'alor1247-besar', 'saru '), ('to_shine', 'alor1247-munas', "sə'ruʔ ")}
 {('to_shine', 'alor1247-munas', 'tutuŋ'), ('to_shine', 'alor1247-pandai', 'tutuŋ')}
 {('to_shine', 'alor1247-munas', 'ɔbaŋ')}
 Alorese shares the following forms with other AN languanges, but not Lamaholot:
 {('to_shine', 'blag1240-warsa', 'bolol'), ('to_shine', 'alor1247-munas', 'bɔlɔr'), ('to_shine', 'blag1240-nule', 'bolor'), ('to_shine', 'blag1240-tuntu', 'bolol'), ('to_shine', 'alor1247-besar', 'bɔlo'), ('to_shine', 'blag1240-kulij', 'bolor'), ('to_shine', 'blag1240-bama', 'bolo'), ('to_shine', 'baka1276', "bo'lor"), ('to_shine', 'alor1247-pandai', 'bolɔr'), ('to_shine', 'keda1252-leuba', 'ɔlɔr'), ('to_shine', 'p-mala1545-acd', '*bilak')}
 One or more Lamaholot dialects innovated forms not found in Alorese:
 {('to_shine', 'lama1277-lerek', 'waləˈkən'), ('to_shine', 'lama1277-kalik', 'vaˈləka'), ('to_shine', 'tetu1245-suai', 'lakan'), ('to_shine', 'tetu1245-vique', 'lakan')}
 {('to_shine', 'lama1277-lewoi', 'ˈhode neʔa')}
 {('to_shine', 'lama1277-lerek', 'ˈonikənɡ')}
 {('to_shine', 'lama1277-adona', 'ˈmorit')}
 {('to_shine', 'lama1277-lewoi', 'ˈhode'), ('to_shine', 'lama1277-lerek', 'ˈhodiŋ')}
 {('to_shine', 'lama1277-lerek', 'ˈtalɛk ˈkay'), ('to_shine', 'lama1277-lerek', 'ˈtalɛk')}
 {('to_shine', 'sika1262-hewa', 'baraʔ'), ('to_shine', 'lama1277-kalik', 'vaˈra')}
 {('to_shine', 'lama1277-adona', 'ˈɡanɜ')}
 {('to_shine', 'lama1277-adona', 'ˈmorit')}
```
